# Supplementary material for: Enantioselective Cu(I)-catalyzed borylative cyclization of enone-tethered cyclohexadienones and mechanistic insights
Source: Nat Commun. 2022 Feb 14;13:854. doi: 10.1038/s41467-022-28288-7 (PMC8844005; doi:10.1038/s41467-022-28288-7)
Supplement: Supplementary file 1 — Supplementary Information [file 41467_2022_28288_MOESM1_ESM.pdf]

Supplementary Information

Enantioselective Cu(I)-Catalyzed Borylative Cyclization of Enone-Tethered Cyclohexadienones and Mechanistic Insights

Sandip B. Jadhav, Soumya Ranjan Dash, Sundaram Maurya, Jagadeesh Babu Nanubolu, Kumar Vanka and Rambabu Chegondi*

E-mail: rchegondi@iict.res.in

Pages

1. Supplementary methods	S-02
2. Supplementary discussion	S-02 to S-99
2a. Preparation of phosphoranes	S-02
2b. Synthesis of enone-tethered cyclohexadienones	S-03 to S-11
2c. Enantioselective borylative cyclization reaction	S-11 to S-57
2d. Sequential borylative cyclization/oxidation reaction with base	S-57 to S-83
2e. Sequential borylative cyclization/oxidation reaction without base:	S-84 to S-93
2f. Gram-scale synthesis of 2a	S-94 to S-95
2g. Synthetic utility	S-95 to S-99
2h. X-ray crystallographic data	S-100 to S-104
2i Computational studies	S-105 to S-107
2j NMR spectra	S-108 to S-185
3. Supplementary reference	S-186 to S-187

1. Supplementary Methods

General information: Unless otherwise noted, all reagents, catalysts and ligands were purchased from Sigma-Aldrich and TCI Chemicals and used without further purification. All reactions were performed under nitrogen atmosphere and in a flame-dried or oven-dried glassware with magnetic stirring. All solvents were dried before use following the standard procedures. Reactions were monitored using thin-layer chromatography (SiO_2). TLC plates were visualized with UV light (254 nm), iodine treatment or using *p*-anisaldehyde stain or *β*-naphthol stain. Column chromatography was carried out using 100-200 mesh silica gel packed in glass columns. NMR spectra were recorded at 300, 400, 500 MHz (H) and at 75, 101, 126 MHz (C), respectively. Chemical shifts (δ) are reported in ppm, using the residual solvent peak in $CDCl_3$ (H: δ = 7.26 and C: δ = 77.16 ppm) as internal standard. Data are reported as follows: chemical shift, multiplicity (s = singlet, d = doublet, t = triplet, dd = doublet of doublets, m = multiplet), coupling constants (Hz) and integration. HRMS were recorded using ESI-TOF techniques. Enantiomeric ratio (*er*) values were determined by chiral HPLC of the purified product and diastereomer ratio (*dr*) values were determined by ^1H NMR analysis.

2. Supplementary Discussion

2a. General Procedure for the Preparation of Phosphoranes:[1]

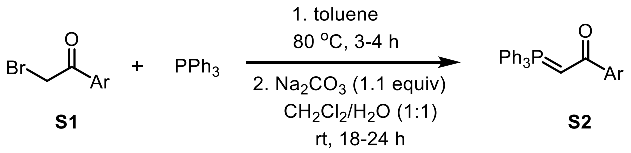

To a solution of 2-bromoacetophenone **S1** (10 mmol) in toluene (0.3 M) was added PPh_3 (11 mmol) at room temperature. The reaction mixture was stirred at 80 °C for 3-4 h. Then the resulting precipitate was filtered, washed with more Et_2O, dried and concentrated in *vacuo* to give the phosphonium salt. To a solution of phosphonium salt in CH_2Cl_2 and was added Na_2CO_3 (11 mmol) in H_2O (1 M) and the resulting biphasic solution was stirred vigorously at room temperature for 18-24 h. The layers were separated and the aqueous layers was extracted with CH_2Cl_2. The combined organic layers were dried (Na_2SO_4), filtered, and concentrated in *vacuo* to give the phosphorene **S2**. The crude Wittig reagent **S2** was used for next reaction without further purification.

2b. General procedure for the synthesis of enone-tethered cyclohexadienones 1:

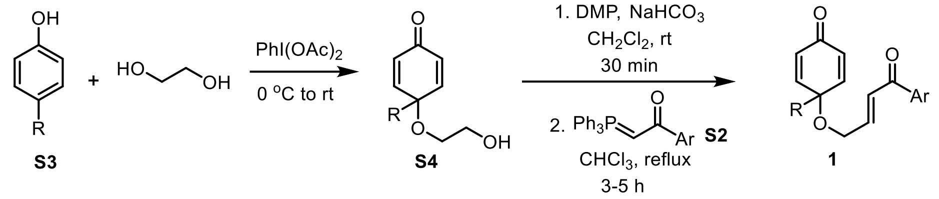

To a stirred solution of phenol **S3** (10 mmol) in CH_2Cl_2 (2 mL) and ethylene glycol (16.7 mL, 300 mmol) was added $PhI(OAc)_2$ (4.84g, 15 mmol, dissolved in 40 mL CH_2Cl_2) dropwise over 2 hours at room temperature under inert atmosphere. After completion of addition, the reaction mixture was stirred for another 30 minutes and then concentrated in *vacuo*. The crude residue was purified by column chromatography (EtOAc/hexane) to give the desired alcohol **S4**.[2]

To a stirred solution of pure alcohol **S4** (10 mmol) in CH_2Cl_2 (0.1 M) was added Dess Martin periodinane (5.9 g, 12 mmol) in one portion at room-temperature and stirred the reaction mixture for 30 minutes to 1 hour under nitrogen atmosphere. The reaction mixture was diluted with hexanes (30 mL) and filtered through Celite and then concentrated in *vacuo*. The crude product was purified by column chromatography (EtOAc/hexane) to give aldehyde in excellent yields.

To the solution of aldehyde in $CHCl_3$ (0.3 M) was added desired phosphorene **S2** (12 mmol, 1.2 equiv) in one portion at room temperature under nitrogen atmosphere. The reaction mixture stirred at 65 °C for 3 to 5 h and then concentrated in *vacuo*. The crude reaction mixture was purified by column chromatography (EtOAc/petroleum ether) to give enone tethered-cyclohexadienones **1** in good yields with excellent diastereoselectivity (*dr* = >20:1). All enone tethered-cyclohexadienones **1** were prepared according to a previously reported procedure unless otherwise mentioned below.[1]

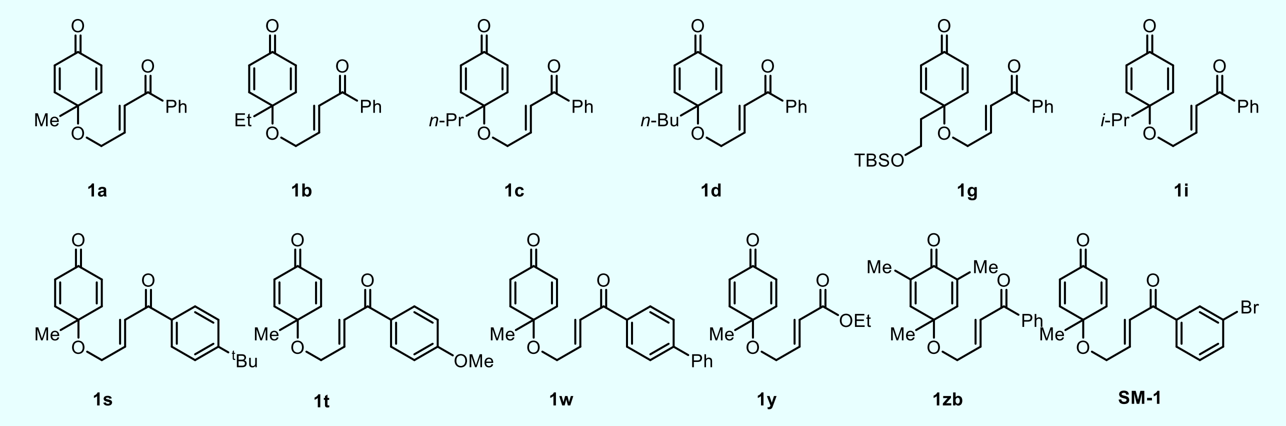

Enone-tethered cyclohexadienones **1a, 1b, 1g, 1i, 1s, 1w, 1y** and **SM-1** were prepared according to a previously reported procedure.[3]

Compounds **1c, 1d** and **1zb** was prepared according to a previously reported procedure.[4]

Compounds **1t** was prepared according to a previously reported procedure.[5]

(*E*)-4-((4-Oxo-4-phenylbut-2-en-1-yl)oxy)-4-pentylcyclohexa-2,5-dien-1-one (1e):

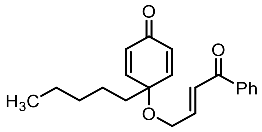

Prepared according to the general procedure as described above in 62% yield (905 mg). It was purified by flash chromatography (20% EtOAc/hexanes; R_f = 0.5) to afford a pale yellow oil; ^{1}H NMR (500 MHz, $CDCl_3$) δ 7.93 (dd, *J* = 8.2, 1.0 Hz, 2H), 7.66 – 7.51 (m, 1H), 7.47 (t, *J* = 7.6 Hz, 2H), 7.15 (dt, *J* = 15.4, 2.0 Hz, 1H), 6.98 (dt, *J* = 15.4, 4.1 Hz, 1H), 6.77 (d, *J* = 10.2 Hz, 2H), 6.36 (d, *J* = 10.2 Hz, 2H), 4.12 (dd, *J* = 4.1, 2.0 Hz, 2H), 1.88 – 1.74 (m, 2H), 1.39 – 1.16 (m, 6H), 0.87 (t, *J* = 6.8 Hz, 3H); ^{13}C NMR (101 MHz, $CDCl_3$) δ 190.3, 185.3, 150.6, 144.5, 137.7, 133.0, 131.5, 128.7, 124.9, 76.1, 64.6, 39.5, 32.0, 23.2, 22.5, 14.0; HRMS (ESI) calcd for $C_{21}H_{24}O_3Na$ [M+Na]$^+$: 347.1623; found: 347.1621.

(*E*)-4-(2-Methoxyethyl)-4-((4-oxo-4-phenylbut-2-en-1-yl)oxy)cyclohexa-2,5-dien-1-one (1f):

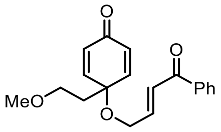

Prepared according to the general procedure as described above in 47% yield (698 mg). It was purified by flash chromatography (30% EtOAc/hexanes; R_f = 0.4) to afford as a pale yellow oil (*dr* = >10:1 ratio of inseparable *E/Z* diastereomers); ^{1}H NMR (500 MHz, $CDCl_3$) δ 7.94 (dd, *J* = 8.3, 1.2 Hz, 2H), 7.59 – 7.54 (m, 1H), 7.48 (t, *J* = 7.6 Hz, 2H), 7.15 (dt, *J* = 15.4, 2.0 Hz, 1H), 6.98 (dt, *J* = 15.5, 4.1 Hz, 1H), 6.84 (d, *J* = 10.3 Hz, 2H), 6.36 (d, *J* = 10.2 Hz, 2H), 4.13 (dd, *J* = 4.1, 2.0 Hz, 2H), 3.50 (t, *J* = 6.2 Hz, 2H), 3.28 (s, 3H), 2.10 (t, *J* = 6.2 Hz, 2H); ^{13}C NMR (101 MHz, $CDCl_3$) δ 190.4, 185.2, 150.2, 144.3, 137.7, 133.1, 131.1, 128.7, 128.7, 125.0, 74.6, 67.5, 64.5, 58.7, 39.7; HRMS (ESI) calcd for $C_{19}H_{21}O_4$ [M+H]$^+$: 313.1440; found: 313.1435.

(*E*)-1-((4-Oxo-4-phenylbut-2-en-1-yl)oxy)-4'-pentyl-[1,1'-bi(cyclohexane)]-2,5-dien-4-one (1j):

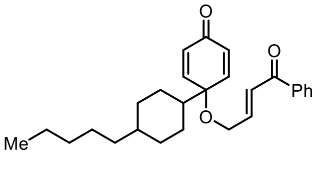

Prepared according to the general procedure as described above in 61% yield (817 mg). It was purified by flash chromatography (20% EtOAc/hexanes; R_f = 0.5) to afford as a yellow semi solid (*dr* = >10:1 ratio of inseparable *E/Z* diastereomers); ^{1}H NMR (500 MHz, $CDCl_3$) δ 7.93 (dt, *J* = 8.5, 1.6 Hz, 2H), 7.59 – 7.54 (m, 1H), 7.48 (t, *J* = 7.6 Hz, 2H), 7.14 (dt, *J* = 15.4, 2.0 Hz, 1H), 6.97 (dt, *J* = 15.5, 4.1 Hz, 1H), 6.77 (d, *J* = 10.3 Hz, 2H), 6.38 (d, *J* = 10.3 Hz, 2H), 4.11 (dd, *J* = 4.0, 2.0 Hz, 2H), 1.94 (d, *J* = 11.9 Hz, 2H), 1.80 (d, *J* = 11.4 Hz, 2H), 1.74 (tt, *J* = 12.2, 3.1 Hz, 2H), 1.34 – 1.19 (m, 7H), 1.19 –

1.08 (m, 3H), 1.03 (ddd, J = 25.0, 12.7, 2.9 Hz, 2H), 0.87 (t, J = 7.1 Hz, 3H); ^{13}C NMR (101 MHz, $CDCl_3$) δ 190.5, 185.6, 149.9, 144.8, 137.7, 133.0, 132.1, 128.7, 128.7, 125.0, 78.3, 64.4, 46.8, 37.7, 37.2, 33.1, 32.2, 27.3, 26.7, 22.8, 14.2; HRMS (ESI) calcd for $C_{27}H_{35}O_3$ $[M+H]^+$: 407.2586; found: 407.2584.

(*E*)-4-(*sec*-Butyl)-4-((4-oxo-4-phenylbut-2-en-1-yl)oxy)cyclohexa-2,5-dien-1-one (1k):

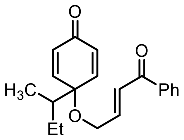

Prepared according to the general procedure as described above in 59% yield (879 mg). It was purified by flash chromatography (20% EtOAc/hexanes; R_f = 0.5) to afford as a pale yellow oil (*dr* = >20:1); ^{1}H NMR (500 MHz, $CDCl_3$) δ 7.93 (dd, J = 8.1, 1.0 Hz, 2H), 7.62 – 7.55 (m, 1H), 7.48 (t, J = 7.6 Hz, 2H), 7.15 (dt, J = 15.5, 2.0 Hz, 1H), 6.99 (dt, J = 15.5, 4.1 Hz, 1H), 6.75 (ddd, J = 10.7, 5.1, 2.8 Hz, 2H), 6.45 – 6.35 (m, 2H), 4.12 (dd, J = 4.0, 2.0 Hz, 2H), 1.91 – 1.72 (m, 2H), 1.04 – 0.90 (m, 7H); ^{13}C NMR (101 MHz, $CDCl_3$) δ 190.5, 185.6, 149.7, 149.5, 144.7, 137.8, 133.0, 132.6, 132.2, 128.7, 128.7, 124.9, 78.7, 64.5, 43.7, 24.0, 13.6, 12.6; HRMS (ESI) calcd for $C_{20}H_{22}O_3Na$ $[M+Na]^+$: 333.1467; found: 333.1466.

(*E*)-4'-Bromo-1-((4-oxo-4-phenylbut-2-en-1-yl)oxy)-[1,1'-biphenyl]-4(1*H*)-one (1n):

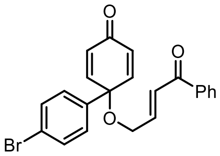

Prepared according to the general procedure as described above in 58% yield (777 mg). It was purified by flash chromatography (20% EtOAc/hexanes; R_f = 0.4) to afford as a yellow semi solid (*dr* = >20:1); ^{1}H NMR (400 MHz, $CDCl_3$) δ 7.93 (dd, J = 8.2, 1.1 Hz, 2H), 7.59 – 7.52 (m, 1H), 7.52 – 7.42 (m, 4H), 7.37 (d, J = 8.7 Hz, 2H), 7.22 (dt, J = 15.5, 1.8 Hz, 1H), 7.05 (dt, J = 15.5, 4.1 Hz, 1H), 6.79 (d, J = 10.1 Hz, 2H), 6.40 (d, J = 10.1 Hz, 2H), 4.33 (dd, J = 4.0, 1.8 Hz, 2H); ^{13}C NMR (101 MHz, $CDCl_3$) δ 190.0, 184.8, 149.0, 143.7, 137.4, 137.0, 133.0, 132.0, 130.3, 128.6, 128.5, 127.5, 125.1, 122.6, 76.2, 64.4; HRMS (ESI) calcd for $C_{22}H_{18}O_3Br$ $[M+H]^+$: 409.0439; found: 409.0442.

(*E*)-4-Methoxy-4-((4-oxo-4-phenylbut-2-en-1-yl)oxy)cyclohexa-2,5-dien-1-one (1o):

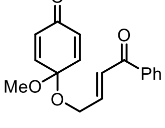

Prepared according to the general procedure as described above in 60% yield (936 mg). It was purified by flash chromatography (30% EtOAc/hexanes; R_f = 0.4) to afford as a pale yellow oil (*dr* = >20:1);

^{1}H NMR (400 MHz, $CDCl_3$) δ 7.93 (d, *J* = 7.4 Hz, 2H), 7.57 (t, *J* = 7.3 Hz, 1H), 7.47 (t, *J* = 7.7 Hz, 2H), 7.17 (dt, *J* = 15.4, 1.9 Hz, 1H), 7.04 (dt, *J* = 15.4, 3.9 Hz, 1H), 6.86 (d, *J* = 10.3 Hz, 2H), 6.31 (d, *J* = 10.4 Hz, 2H), 4.43 (dd, *J* = 3.9, 1.9 Hz, 2H), 3.42 (s, 3H); ^{13}C NMR (101 MHz, $CDCl_3$) δ 190.2, 185.0, 143.4, 142.9, 137.6, 133.1, 130.2, 128.8, 128.7, 125.2, 92.8, 62.0, 50.9; HRMS (ESI) calcd for $C_{17}H_{17}O_4$ $[M+H]^+$: 285.1127; found: 285.1126.

(*E*)-4-(Benzyloxy)-4-((4-oxo-4-phenylbut-2-en-1-yl)oxy)cyclohexa-2,5-dien-1-one (1q):

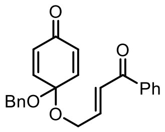

Prepared according to the general procedure as described above in 45% yield (628 mg). It was purified by flash chromatography (30% EtOAc/hexanes; R_f = 0.5) to afford as a yellow semi solid (*dr* = >20:1); ^{1}H NMR (400 MHz, $CDCl_3$) δ 7.93 (dd, *J* = 8.3, 1.3 Hz, 2H), 7.62 – 7.53 (m, 1H), 7.51 – 7.44 (m, 2H), 7.42 – 7.29 (m, 5H), 7.18 (dt, *J* = 15.4, 1.9 Hz, 1H), 7.05 (dt, *J* = 15.4, 4.0 Hz, 1H), 6.94 (d, *J* = 10.3 Hz, 2H), 6.31 (d, *J* = 10.3 Hz, 2H), 4.71 (s, 2H), 4.48 (dd, *J* = 4.0, 1.9 Hz, 2H); ^{13}C NMR (101 MHz, $CDCl_3$) δ 190.1, 185.1, 143.3, 143.1, 137.5, 137.3, 133.1, 130.0, 128.7, 128.7, 128.1, 127.7, 125.3, 93.0, 65.4, 62.2; HRMS (ESI) calcd for $C_{23}H_{20}O_4Na$ $[M+Na]^+$: 383.1259; found: 383.1257.

(*E*)-4-Methyl-4-((4-oxo-4-(*p*-tolyl)but-2-en-1-yl)oxy)cyclohexa-2,5-dien-1-one (1r):

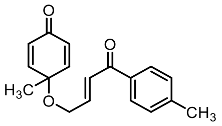

Prepared according to the general procedure as described above in 63% yield (1070 mg). It was purified by flash chromatography (20% EtOAc/hexanes; R_f = 0.4) to afford as a yellow semi solid (*dr* = >20:1); ^{1}H NMR (400 MHz, $CDCl_3$) δ 7.86 (d, *J* = 8.2 Hz, 2H), 7.28 (d, *J* = 8.0 Hz, 2H), 7.15 (dt, *J* = 15.4, 1.9 Hz, 1H), 6.97 (dt, *J* = 15.4, 4.2 Hz, 1H), 6.82 (d, *J* = 10.2 Hz, 2H), 6.33 (d, *J* = 10.2 Hz, 2H), 4.12 (dd, *J* = 4.2, 1.9 Hz, 2H), 2.42 (s, 3H), 1.53 (s, 3H); ^{13}C NMR (101 MHz, $CDCl_3$) δ 189.8, 185.0, 151.2, 143.9, 143.7, 135.1, 130.6, 129.4, 128.8, 125.1, 72.9, 64.9, 26.4, 21.8; HRMS (ESI) calcd for $C_{18}H_{19}O_3$ $[M+H]^+$: 283.1329; found: 283.1315.

(*E*)-4-((4-(4-Chlorophenyl)-4-oxobut-2-en-1-yl)oxy)-4-methylcyclohexa-2,5-dien-1-one (1u):

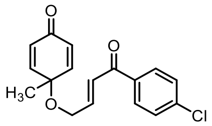

Prepared according to the general procedure as described above in 60% yield (1092 mg). It was purified by flash chromatography (20% EtOAc/hexanes; R_f = 0.4) to afford as a pale yellow oil (*dr* = >20:1); ^{1}H NMR (500 MHz, $CDCl_3$) δ 7.88 (d, *J* = 8.6 Hz, 2H), 7.44 (d, *J* = 8.6 Hz, 2H), 7.10 (dt, *J* =

15.4, 2.0 Hz, 1H), 6.98 (dt, J = 15.4, 4.0 Hz, 1H), 6.80 (d, J = 10.2 Hz, 2H), 6.32 (d, J = 10.2 Hz, 2H), 4.11 (dd, J = 4.0, 2.0 Hz, 2H), 1.52 (s, 3H); ^{13}C NMR (101 MHz, $CDCl_3$) δ 189.0, 184.9, 151.0, 144.8, 139.5, 136.0, 130.7, 130.1, 129.0, 124.5, 73.0, 64.8, 26.4; HRMS (ESI) calcd for $C_{17}H_{16}O_3Cl$ $[M+H]^+$: 303.0783; found: 303.0771.

(*E*)-4-((4-(4-Bromophenyl)-4-oxobut-2-en-1-yl)oxy)-4-methylcyclohexa-2,5-dien-1-one (1v):

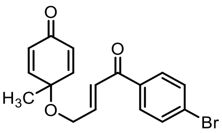

Prepared according to the general procedure as described above in 62% yield (1296 mg). It was purified by flash chromatography (20% EtOAc/hexanes; R_f = 0.4) to afford as a yellow semi solid (*dr* = >20:1); ^{1}H NMR (400 MHz, $CDCl_3$) δ 7.79 (dd, J = 8.4, 1.6 Hz, 2H), 7.59 (dd, J = 8.6, 2.0 Hz, 2H), 7.08 (dd, J = 15.4, 1.3 Hz, 1H), 6.97 (dtd, J = 15.4, 3.9, 1.5 Hz, 1H), 6.79 (d, J = 10.1 Hz, 2H), 6.30 (dd, J = 10.0, 1.6 Hz, 2H), 4.10 (dd, J = 3.7, 1.8 Hz, 2H), 1.51 (s, 3H); ^{13}C NMR (101 MHz, $CDCl_3$) δ 189.1, 184.9, 150.9, 144.8, 136.4, 132.0, 130.6, 130.2, 128.1, 124.4, 72.9, 64.8, 26.4; HRMS (ESI) calcd for $C_{17}H_{16}BrO_3$ $[M+H]^+$: 347.0277; found: 347.0268.

(*E*)-4-Methyl-4-((4-(4-nitrophenyl)-4-oxobut-2-en-1-yl)oxy)cyclohexa-2,5-dien-1-one (SM-2):

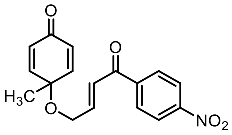

Prepared according to the general procedure as described above in 48% yield (905 mg). It was purified by flash chromatography (30% EtOAc/hexanes; R_f = 0.3) to afford as a pale yellow oil (*dr* = >20:1); ^{1}H NMR (500 MHz, $CDCl_3$) δ 8.31 (dd, J = 8.7, 1.8 Hz, 2H), 8.07 (dd, J = 8.7, 1.4 Hz, 2H), 7.13 (dt, J = 15.5, 1.4 Hz, 1H), 7.04 (dtd, J = 15.5, 3.8, 1.4 Hz, 1H), 6.80 (d, J = 10.2 Hz, 2H), 6.33 (dd, J = 10.0, 1.5 Hz, 2H), 4.14 (dd, J = 3.3, 1.8 Hz, 2H), 1.54 (s, 3H); ^{13}C NMR (101 MHz, $CDCl_3$) δ 188.8, 184.9, 150.8, 150.2, 146.5, 142.4, 130.8, 129.6, 124.3, 123.9, 73.0, 64.7, 26.3; HRMS (ESI) calcd for $C_{17}H_{16}O_5N$ $[M+H]^+$: 314.1023; found: 314.1014.

(*E*)-4-(4-((1-Methyl-4-oxocyclohexa-2,5-dien-1-yl)oxy)but-2-enoyl)benzonitrile (1x):

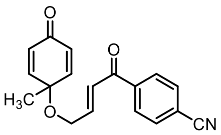

Prepared according to the general procedure as described above in 50% yield (883 mg). It was purified by flash chromatography (30% EtOAc/hexanes; R_f = 0.3) to afford as a yellow semi solid (*dr* = >20:1); ^{1}H NMR (500 MHz, $CDCl_3$) δ 7.99 (d, J = 8.0 Hz, 2H), 7.76 (d, J = 8.4 Hz, 2H), 7.09 (dd, J = 15.5, 1.7 Hz, 1H), 7.04 – 6.96 (m, 1H), 6.78 (d, J = 10.1 Hz, 2H), 6.30 (dd, J = 10.2, 2.5 Hz, 2H), 4.11 (dd,

J = 5.6, 3.7 Hz, 2H), 1.51 (s, 3H); ^{13}C NMR (101 MHz, $CDCl_3$) δ 188.9, 184.8, 150.8, 146.2, 140.8, 132.5, 130.7, 129.0, 124.1, 118.0, 116.1, 72.9, 64.6, 26.3; HRMS (ESI) calcd for $C_{18}H_{16}O_3N$ $[M+H]^+$: 294.1125; found: 294.1115.

(*E*)-4-Methyl-4-((4-oxopent-2-en-1-yl)oxy)cyclohexa-2,5-dien-1-one (1za):

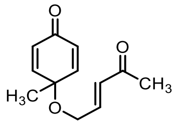

Prepared according to the general procedure as described above in 66% yield (819 mg). It was purified by flash chromatography (20% EtOAc/hexanes; R_f = 0.5) to afford as a pale yellow oil (*dr* = >20:1); ^{1}H NMR (500 MHz, $CDCl_3$) δ 6.85 (d, J = 10.2 Hz, 2H), 6.77 (dt, J = 16.1, 4.3 Hz, 1H), 6.39 – 6.22 (m, 3H), 4.07 (dd, J = 4.4, 2.1 Hz, 2H), 2.27 (s, 3H), 1.51 (s, 3H); ^{13}C NMR (75 MHz, $CDCl_3$) δ 197.2, 184.1, 150.5, 142.6, 129.9, 129.5, 72.2, 63.7, 26.6, 25.6; HRMS (ESI) calcd for $C_{12}H_{14}O_3Na$ $[M+Na]^+$: 229.0841; found: 229.0838.

(*E*)-1-Methoxy-2'-(3-oxo-3-phenylprop-1-en-1-yl)-[1,1'-biphenyl]-4(1*H*)-one (SM-3):

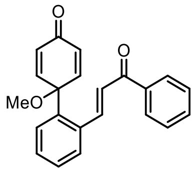

Prepared according to the general procedure as described above in 48% yield (695 mg). It was purified by flash chromatography (20% EtOAc/hexanes; R_f = 0.3) to afford as a yellow semi solid (*dr* = >20:1); ^{1}H NMR (500 MHz, $CDCl_3$) δ 8.60 (d, J = 15.5 Hz, 1H), 8.07 – 7.99 (m, 2H), 7.67 (dd, J = 7.4, 1.5 Hz, 1H), 7.59 (t, J = 7.4 Hz, 1H), 7.53 – 7.48 (m, 3H), 7.43 – 7.34 (m, 2H), 7.26 (d, J = 15.5 Hz, 1H), 6.93 (d, J = 9.9 Hz, 2H), 6.44 (d, J = 10.0 Hz, 2H), 3.37 (s, 3H); ^{13}C NMR (101 MHz, $CDCl_3$) δ 190.5, 185.0, 148.4, 144.7, 138.0, 137.9, 135.4, 132.9, 130.8, 130.1, 129.2, 128.7, 128.6, 126.8, 124.4, 76.9, 52.2; HRMS (ESI) calcd for $C_{22}H_{19}O_3$ $[M+H]^+$: 331.1329; found: 331.1323.

(*E*)-4-Methoxy-4-(5-oxo-5-phenylpent-3-en-1-yl)cyclohexa-2,5-dien-1-one (4):

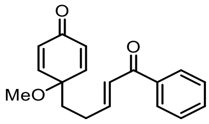

Prepared according to the general procedure as described above in 70% yield (1135 mg). It was purified by flash chromatography (20% EtOAc/hexanes; R_f = 0.3) to afford as a orange semi solid (*dr* = >20:1); ^{1}H NMR (400 MHz, $CDCl_3$) δ 7.93 – 7.87 (m, 2H), 7.60 – 7.51 (m, 1H), 7.49 – 7.38 (m, 2H), 6.98 (dt, J = 15.4, 6.5 Hz, 1H), 6.86 (dt, J = 15.4, 1.3 Hz, 1H), 6.76 (d, J = 10.3 Hz, 2H), 6.40 (d, J = 10.3 Hz, 2H), 3.22 (s, 3H), 2.40 – 2.24 (m, 2H), 2.00 – 1.88 (m, 2H); ^{13}C NMR (101 MHz, $CDCl_3$)

δ 190.5, 185.2, 150.5, 147.8, 137.8, 132.9, 132.0, 128.7, 128.6, 126.3, 75.3, 53.3, 37.8, 27.1; HRMS (ESI) calcd for $C_{18}H_{19}O_3$ $[M+H]^+$: 283.1329; found: 283.1322.

(*E*)-3,4-Dimethyl-4-((4-oxo-4-phenylbut-2-en-1-yl)oxy)cyclohexa-2,5-dien-1-one (7):

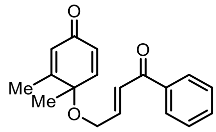

Prepared according to the general procedure as described above in 46% yield (721 mg). It was purified by flash chromatography (20% EtOAc/hexanes; R_f = 0.5) to afford as a yellow semi solid (*dr* = >20:1); ^{1}H NMR (500 MHz, $CDCl_3$) δ 7.94 (dd, *J* = 8.2, 1.0 Hz, 2H), 7.60 – 7.53 (m, 1H), 7.51 – 7.45 (m, 2H), 7.17 (dt, *J* = 15.4, 2.0 Hz, 1H), 6.98 (dt, *J* = 11.4, 4.1 Hz, 1H), 6.80 (d, *J* = 10.0 Hz, 1H), 6.30 (dd, *J* = 10.0, 1.6 Hz, 1H), 6.20 (s, 1H), 4.04 (ddd, *J* = 16.2, 4.1, 2.0 Hz, 1H), 3.87 (ddd, *J* = 16.2, 4.1, 2.0 Hz, 1H), 2.00 (s, 3H), 1.50 (s, 3H); ^{13}C NMR (101 MHz, $CDCl_3$) δ 190.3, 185.4, 160.0, 151.5, 143.9, 137.7, 133.1, 130.3, 129.3, 128.7, 128.7, 125.0, 74.8, 64.4, 25.6, 18.0; HRMS (ESI) calcd for $C_{18}H_{19}O_3$ $[M+H]^+$: 283.1329; found: 283.1321.

2c′. General procedure for the preparation of *N*- tethered enones:[6]

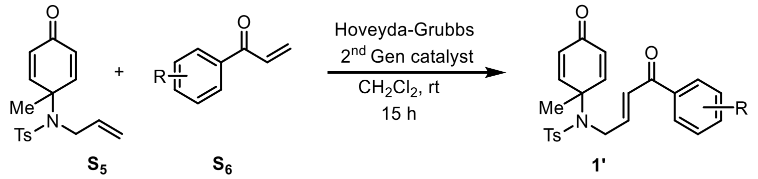

Second generation Hoveyda-Grubbs catalyst (11.3 mg, 0.018 mmol, 3 mol%) was added to the mixture of *p*-quinamine **S5** (0.6 mmol, 1 equiv) and enone **S6** (1.8 mmol, 3 equiv) in degassed CH_2Cl_2 (15 mL). The reaction was stirred at rt for 3 h and another 3.0 equiv of enone (1.8 mmol) was added to the reaction mixture and stirring was continued for 12 h. After completion of the reaction (monitored by TLC), the solution was evaporated and the crude product was subjected to flash column chromatography on silica gel (EtOAc/Hexanes) to give *N*-tethered enone.

(*E*)-4-Methyl-*N*-(1-methyl-4-oxocyclohexa-2,5-dien-1-yl)-*N*-(4-oxo-4-phenylbut-2-en-1-yl)benzenesulfonamide (1o′):

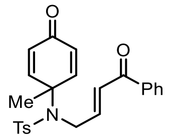

Prepared according to the general procedure as described above in 62% yield (157 mg). It was purified by flash chromatography (30% EtOAc/hexanes; R_f = 0.3) to afford as a white solid; mp = 201–203°C;

(dr = >20:1); ^{1}H NMR (500 MHz, $CDCl_3$) δ 7.92 (dd, J = 8.3, 1.2 Hz, 2H), 7.74 – 7.69 (m, 2H), 7.62 – 7.55 (m, 1H), 7.52 – 7.46 (m, 2H), 7.30 (d, J = 8.0 Hz, 2H), 7.08 (dt, J = 15.4, 1.3 Hz, 1H), 7.01 – 6.94 (m, 1H), 6.89 (d, J = 10.2 Hz, 2H), 6.17 (d, J = 10.2 Hz, 2H), 4.19 (dd, J = 5.5, 1.3 Hz, 2H), 2.42 (s, 3H), 1.58 (s, 3H); ^{13}C NMR (101 MHz, $CDCl_3$) δ 189.9, 184.2, 150.6, 144.5, 144.4, 138.9, 137.4, 133.3, 130.1, 128.8, 128.7, 128.6, 127.6, 127.6, 60.5, 48.7, 26.1, 21.7; HRMS (ESI) calcd for $C_{24}H_{24}NO_4S$ [M+H]$^+$: 422.1426; found: 422.1420.

(*E*)-4-Methyl-*N*-(1-methyl-4-oxocyclohexa-2,5-dien-1-yl)-*N*-(4-oxo-4-(p-tolyl)but-2-en-1-yl)benzenesulfonamide (1p′):

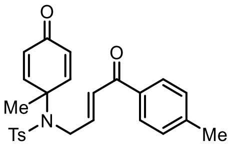

Prepared according to the general procedure as described above in 57% yield (149 mg). It was purified by flash chromatography (30% EtOAc/hexanes; R_f = 0.3) to afford as a white solid; mp = 193–195°C; (dr = >20:1); ^{1}H NMR (400 MHz, $CDCl_3$) δ 7.84 (d, J = 8.2 Hz, 2H), 7.72 (d, J = 8.4 Hz, 2H), 7.34 – 7.26 (m, 4H), 7.08 (dt, J = 15.4, 1.3 Hz, 1H), 7.00 – 6.92 (m, 1H), 6.90 (d, J = 10.2 Hz, 2H), 6.18 (d, J = 10.2 Hz, 2H), 4.18 (dd, J = 5.5, 1.3 Hz, 2H), 2.43 (s, 3H), 2.42 (s, 3H), 1.58 (s, 3H); ^{13}C NMR (101 MHz, $CDCl_3$) δ 189.5, 184.3, 150.7, 144.3, 143.9, 139.0, 134.9, 130.1, 129.9, 129.6, 129.1, 128.9, 128.6, 127.7, 127.6, 127.4, 60.6, 48.7, 26.1, 21.9, 21.7; HRMS (ESI) calcd for $C_{25}H_{26}NO_4S$ [M+H]$^+$: 436.1583; found: 436.1575.

(*E*)-*N*-(4-(4-Fluorophenyl)-4-oxobut-2-en-1-yl)-4-methyl-*N*-(1-methyl-4-oxocyclohexa-2,5-dien-1-yl)benzenesulfonamide (1q′):

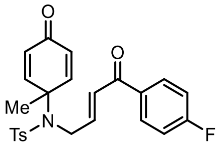

Prepared according to the general procedure as described above in 73% yield (193 mg). It was purified by flash chromatography (30% EtOAc/hexanes; R_f = 0.3) to afford as a white solid; mp = 221–223°C; (dr = >20:1); ^{1}H NMR (400 MHz, $CDCl_3$) δ 8.00 – 7.91 (m, 2H), 7.71 (d, J = 8.3 Hz, 2H), 7.31 (d, J = 8.0 Hz, 2H), 7.21 – 7.12 (m, 2H), 7.10 – 7.03 (m, 1H), 7.03 – 6.94 (m, 1H), 6.88 (d, J = 10.2 Hz, 2H), 6.17 (d, J = 10.2 Hz, 2H), 4.18 (dd, J = 5.2, 1.0 Hz, 2H), 2.43 (s, 3H), 1.58 (s, 3H); ^{13}C NMR (101 MHz, $CDCl_3$) δ 188.3, 184.2, 166.0 (d, J_{CF} = 255.2 Hz), 150.6, 144.7, 144.4, 138.9, 133.8 (d, J_{CF} = 2.3 Hz), 131.4 (d, J_{CF} = 9.3 Hz), 130.1, 128.6, 127.6, 127.3, 116.0 (d, J_{CF} = 21.9 Hz), 60.5, 48.7, 26.0, 21.7; ^{19}F NMR (376 MHz, $CDCl_3$) δ -104.70 (s, 1F); HRMS (ESI) calcd for $C_{24}H_{23}FNO_4S$ [M+H]$^+$: 440.1332; found: 440.1333.

(*E*)-*N*-(4-(2-Methoxyphenyl)-4-oxobut-2-en-1-yl)-4-methyl-*N*-(1-methyl-4-oxocyclohexa-2,5-dien-1-yl)benzenesulfonamide (1r′):

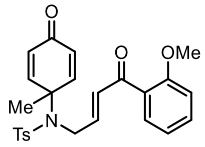

Prepared according to the general procedure as described above in 66% yield (179 mg). It was purified by flash chromatography (30% EtOAc/hexanes; R_f = 0.2) to afford as a white solid; mp = 206–208°C; (*dr* = >20:1); ^{1}H NMR (400 MHz, $CDCl_3$) δ 7.70 (d, *J* = 8.3 Hz, 2H), 7.61 (dd, *J* = 7.6, 1.8 Hz, 1H), 7.48 (ddd, *J* = 8.4, 7.4, 1.8 Hz, 1H), 7.28 (d, *J* = 8.0 Hz, 2H), 7.07 – 6.94 (m, 3H), 6.91 – 6.86 (m, 2H), 6.86 – 6.79 (m, 1H), 6.15 (d, *J* = 10.2 Hz, 2H), 4.16 (dd, *J* = 5.4, 1.4 Hz, 2H), 3.88 (s, 3H), 2.41 (s, 3H), 1.58 (s, 3H); ^{13}C NMR (151 MHz, $CDCl_3$) δ 191.9, 184.3, 158.6, 150.8, 144.2, 142.1, 139.0, 133.7, 132.3, 130.8, 130.0, 128.5, 128.4, 127.6, 120.9, 111.7, 60.4, 55.8, 48.5, 26.1, 21.7; HRMS (ESI) calcd for $C_{25}H_{26}NO_5S$ $[M+H]^+$: 452.1532; found: 452.1549.

2c. General procedure for the borylative cyclization reaction

A solution of $Cu(CH_3CN)_4PF_6$ (2.8 mg, 2.5 mol%), (*S*)-SEGPHOS (9 mg, 5 mol%), $B_2(pin)_2$ (84 mg, 0.33 mmol) and ^{t}BuOLi (53 µl, 0.6 mmol, 1 M in THF) in dry THF (2.0 mL) was stirred at room temperature for 15 min and then maintained at -78 ^{o}C. A solution of enone **1** (0.3 mmol) in dry THF (1.0 mL) was added via syringe and the resulting mixture was stirred at -78 ^{o}C for 3 h. The reaction mixture was quenched with saturated NH_4Cl (10 mL) solution and extracted with EtOAc (3 × 15 mL) and dried over anhydrous Na_2SO_4, filtered, and concentrated in *vacuo*. The resultant crude product was purified by column chromatography (hexanes/EtOAc).

(3S,4R,4aS,8aR)-4-Benzoyl-8a-methyl-3-(4,4,5,5-tetramethyl-1,3,2-dioxaborolan-2-yl)-3,4,4a,8a-tetrahydro-2*H*-chromen-6(5*H*)-one (2a):

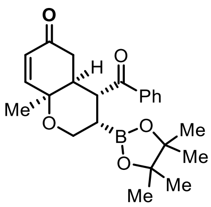

Prepared according to the general procedure as described above in 92% yield (109 mg) with 12:1*dr*. It was purified by flash chromatography (20% EtOAc/hexanes; R_f = 0.3) to afford a brown semi solid; ^{1}H NMR (400 MHz, $CDCl_3$) δ 7.75 (dd, *J* = 5.2, 3.4 Hz, 2H), 7.56 – 7.48 (m, 1H), 7.46 – 7.36 (m, 2H), 6.67 (dd, *J* = 10.3, 1.4 Hz, 1H), 6.04 (d, *J* = 10.3 Hz, 1H), 4.01 (dd, *J* = 11.6, 4.1 Hz, 1H), 3.85 (dd, *J* = 11.6, 3.3 Hz, 1H), 3.53 (dd, *J* = 9.2, 4.8 Hz, 1H), 2.91 (dt, *J* = 8.8, 4.4 Hz, 1H), 2.70 – 2.58

(m, 2H), 1.53 (dd, *J* = 8.0, 4.0 Hz, 1H), 1.44 (s, 3H), 1.21 (s, 6H), 1.19 (s, 6H); ^{13}C NMR (101 MHz, $CDCl_3$) δ 201.6, 198.6, 155.3, 136.8, 133.0, 130.0, 128.8, 128.3, 83.6, 73.2, 64.4, 45.9, 40.2, 39.5, 25.7, 24.8, 24.7; HRMS (ESI) calcd for $C_{23}H_{30}BO_5$ $[M+H]^+$: 397.2181; found: 397.2179; $[\alpha]^{20}_D$ = -92.77° (*c* 2.23, $CHCl_3$); 96:04 *er*; Chiral HPLC analysis of the product: Daicel Chiralpak IA 250X4.6 mm 5μ column; hexane/2-propanol = 80/20, detected at 240 nm, Flow rate = 1 mL/min, Retention times: 5.297 min (major), 4.551 min (minor).

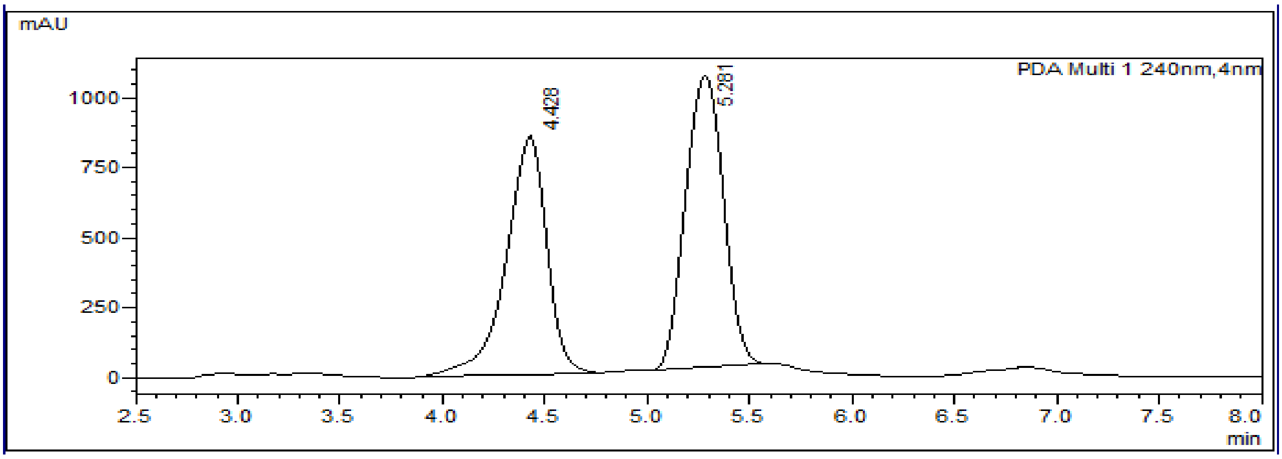

<Peak Table>

PDA Ch1 240nm

Peak#	Ret. Time	Area	Height	Area%	Height%
1	4.428	12192756	855463	47.746	45.130
2	5.281	13343731	1040081	52.254	54.870
Total		25536488	1895545	100.000	100.000

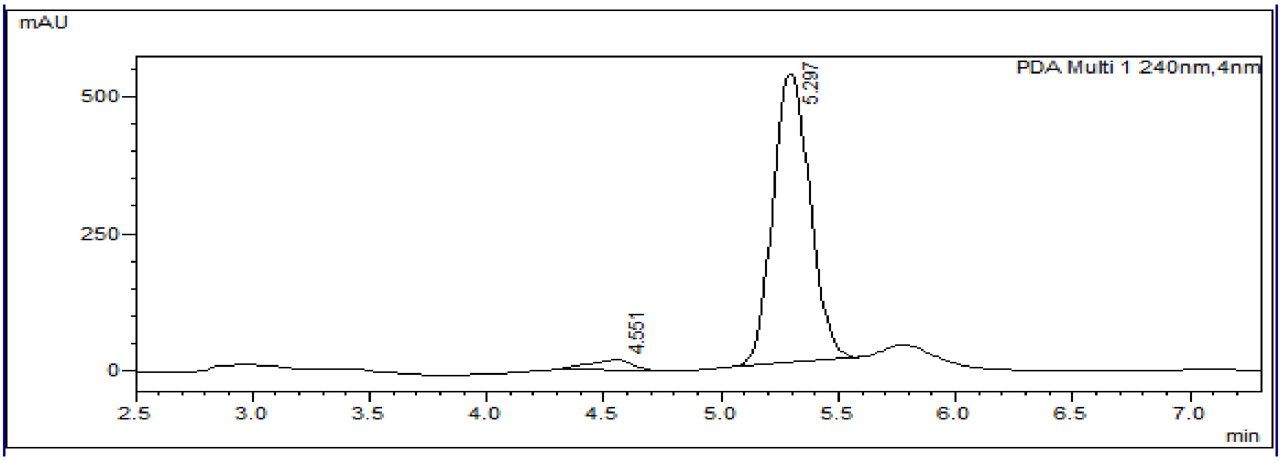

<Peak Table>

PDA Ch1 240nm

Peak#	Ret. Time	Area	Height	Area%	Height%
1	4.551	242132	18454	4.023	3.402
2	5.297	5776196	524001	95.977	96.598
Total		6018328	542455	100.000	100.000

(3S,4R,4aS,8aR)-4-Benzoyl-8a-ethyl-3-(4,4,5,5-tetramethyl-1,3,2-dioxaborolan-2-yl)-3,4,4a,8a-tetrahydro-2H-chromen-6(5*H*)-one (2b):

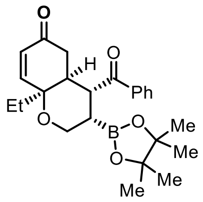

Prepared according to the general procedure as described above in 92% yield (113 mg) with >20:1*dr*. It was purified by flash chromatography (20% EtOAc/hexanes; R_f = 0.3) to afford a brown semi solid; ^{1}H NMR (400 MHz, $CDCl_3$) δ 7.74 (dd, *J* = 5.2, 3.3 Hz, 2H), 7.58 – 7.47 (m, 1H), 7.46 – 7.36 (m, 2H), 6.74 (dd, *J* = 10.4, 1.7 Hz, 1H), 6.07 (d, *J* = 10.3 Hz, 1H), 3.99 (dd, *J* = 11.5, 3.4 Hz, 1H), 3.86 (dd, *J* = 11.5, 3.2 Hz, 1H), 3.57 (dd, *J* = 9.8, 4.8 Hz, 1H), 3.16 – 3.02 (m, 1H), 2.62 (dd, *J* = 17.6, 5.0 Hz, 1H), 2.54 (dd, *J* = 17.6, 4.0 Hz, 1H), 1.84 – 1.67 (m, 2H), 1.50 (dd, *J* = 7.8, 3.3 Hz, 1H), 1.20 (s, 6H), 1.19 (s, 6H), 1.00 (t, *J* = 7.4 Hz, 3H); ^{13}C NMR (101 MHz, $CDCl_3$) δ 201.4, 198.6, 155.8, 136.9, 133.0, 130.3, 128.8, 128.3, 83.5, 75.5, 64.5, 45.6, 39.9, 35.8, 31.1, 25.0, 24.6, 7.8; HRMS (ESI) calcd for $C_{24}H_{31}BO_5Na$ $[M+Na]^+$: 433.2162; found: 433.2178; $[\alpha]^{20}_D$ = -103.60° (*c* 2.20, $CHCl_3$); 93:07 *er*; Chiral HPLC analysis of the product: Daicel Chiralpak IA 250X4.6 mm 5µ column; hexane/2-propanol = 95/05, detected at 254 nm, Flow rate = 1 mL/min, Retention times: 9.057 min (major), 6.678 min (minor).

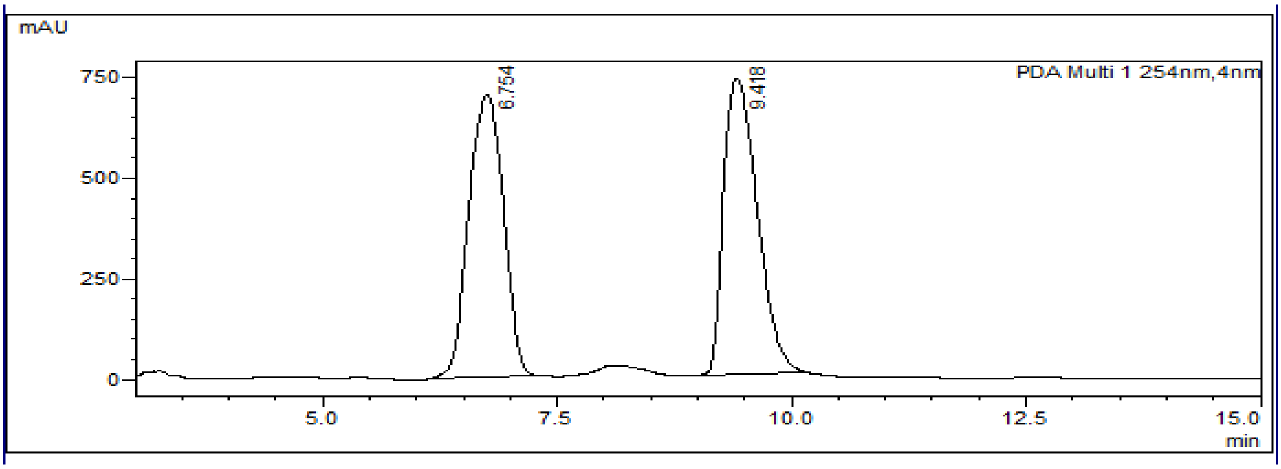

<Peak Table>

PDA Ch1 254nm

Peak#	Ret. Time	Area	Height	Area%	Height%
1	6.754	18162350	701799	49.746	48.927
2	9.418	18348067	732588	50.254	51.073
Total		36510417	1434387	100.000	100.000

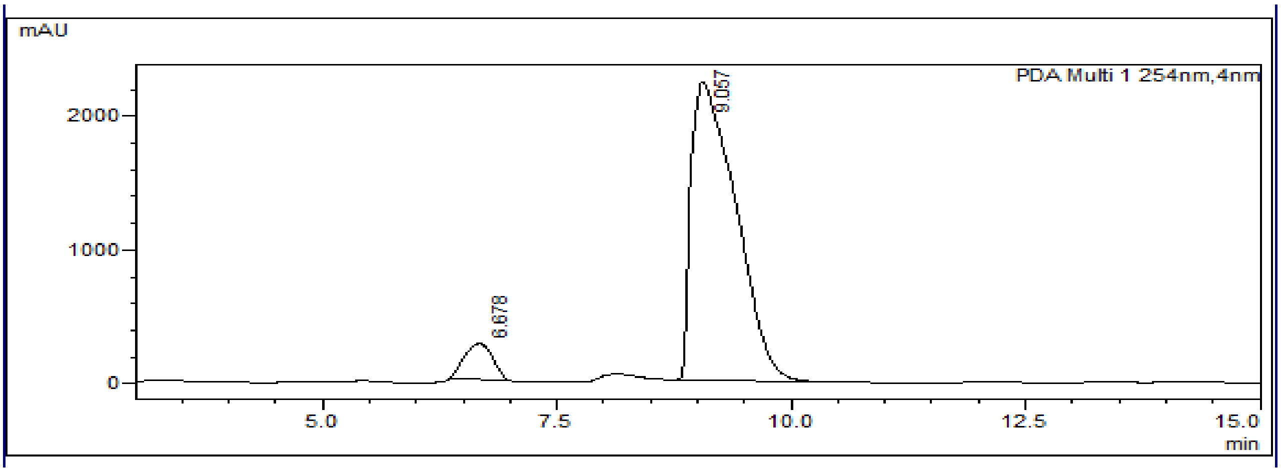

<Peak Table>

PDA Ch1 254nm

Peak#	Ret. Time	Area	Height	Area%	Height%
1	6.678	5757742	271718	7.194	10.868
2	9.057	74276096	2228549	92.806	89.132
Total		80033838	2500267	100.000	100.000

(3S,4R,4aS,8aR)-4-Benzoyl-8a-propyl-3-(4,4,5,5-tetramethyl-1,3,2-dioxaborolan-2-yl)-3,4,4a,8a-tetrahydro-2*H*-chromen-6(5*H*)-one (2c):

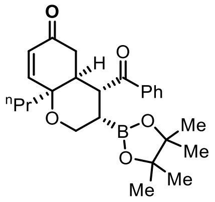

Prepared according to the general procedure as described above in 91% yield (116 mg) with >20:1*dr*. It was purified by flash chromatography (20% EtOAc/hexanes; R_f = 0.4) to afford a brown semi solid; ^{1}H NMR (400 MHz, $CDCl_3$) δ 7.74 (dd, *J* = 5.2, 3.3 Hz, 2H), 7.55 – 7.44 (m, 1H), 7.46 – 7.36 (m, 2H), 6.73 (dd, *J* = 10.3, 1.7 Hz, 1H), 6.06 (d, *J* = 10.3 Hz, 1H), 3.98 (dd, *J* = 11.5, 3.4 Hz, 1H), 3.85 (dd, *J* = 11.5, 3.2 Hz, 1H), 3.56 (dd, *J* = 9.8, 4.8 Hz, 1H), 3.19 – 2.99 (m, 1H), 2.63 (dd, *J* = 17.6, 5.0 Hz, 1H), 2.54 (dd, *J* = 17.6, 3.9 Hz, 1H), 1.79 – 1.63 (m, 2H), 1.63 – 1.47 (m, 2H), 1.40 (ddd, J = 21.2, 13.5, 6.6 Hz, 1H)., 1.20 (s, 6H), 1.19 (s, 6H), 0.90 (t, *J* = 7.3 Hz, 3H); ^{13}C NMR (101 MHz, $CDCl_3$) δ 201.4, 198.6, 155.9, 136.9, 133.0, 130.2, 128.8, 128.3, 83.5, 75.5, 64.6, 45.7, 40.7, 39.9, 36.3, 25.0, 24.6, 16.8, 14.6; HRMS (ESI) calcd for $C_{25}H_{33}BO_5Na$ $[M+Na]^+$: 447.2319; found: 447.2327; $[\alpha]^{20}_D$ = -109.99° (*c* 2.38, $CHCl_3$); 92:08 *er*; Chiral HPLC analysis of the product: Daicel Chiralpak IA 250X4.6 mm 5μ column; hexane/2-propanol = 90/10, detected at 220 nm, Flow rate = 1 mL/min, Retention times: 7.397 min (major), 5.498 min (minor).

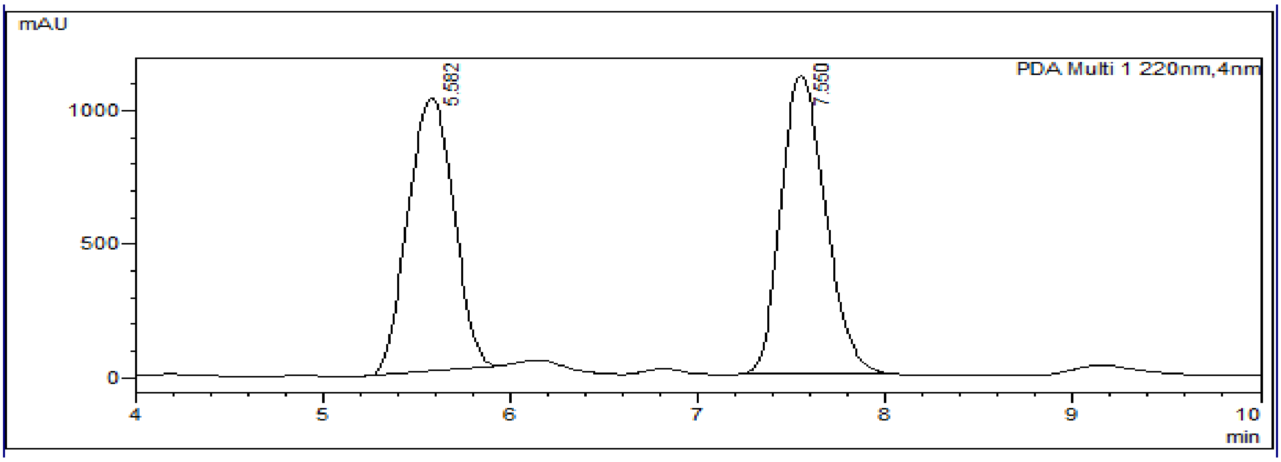

<Peak Table>

PDA Ch1 220nm

Peak#	Ret. Time	Area	Height	Area%	Height%
1	5.582	17438335	1020272	48.403	47.693
2	7.550	18589168	1118977	51.597	52.307
Total		36027503	2139249	100.000	100.000

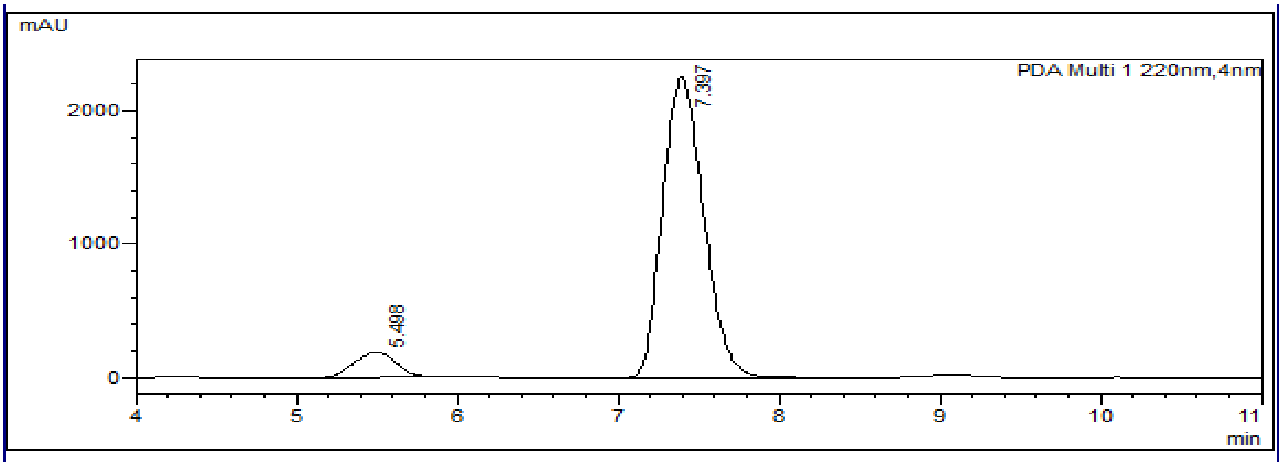

<Peak Table>

PDA Ch1 220nm

Peak#	Ret. Time	Area	Height	Area%	Height%
1	5.498	3284090	183985	7.611	7.562
2	7.397	39862376	2249105	92.389	92.438
Total		43146465	2433090	100.000	100.000

(3S,4R,4aS,8aR)-4-Benzoyl-8a-butyl-3-(4,4,5,5-tetramethyl-1,3,2-dioxaborolan-2-yl)-3,4,4a,8a-tetrahydro-2*H*-chromen-6(5*H*)-one (2d):

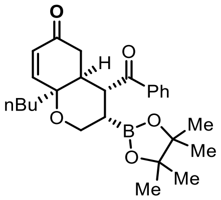

Prepared according to the general procedure as described above in 89% yield (117 mg) with >20:1*dr*. It was purified by flash chromatography (20% EtOAc/hexanes; R_f = 0.4) to afford a brown semi solid; ^{1}H NMR (400 MHz, $CDCl_3$) δ 7.75 (dd, *J* = 5.2, 3.3 Hz, 2H), 7.56 – 7.47 (m, 1H), 7.46 – 7.36 (m, 2H), 6.73 (dd, *J* = 10.3, 1.7 Hz, 1H), 6.07 (d, *J* = 10.3 Hz, 1H), 3.99 (dd, *J* = 11.5, 3.4 Hz, 1H), 3.86 (dd, *J* = 11.5, 3.1 Hz, 1H), 3.57 (dd, *J* = 9.9, 4.8 Hz, 1H), 3.14 – 3.04 (m, 1H), 2.63 (dd, *J* = 17.6, 5.0 Hz, 1H), 2.54 (dd, *J* = 17.6, 3.8 Hz, 1H), 1.79 – 1.65 (m, 2H), 1.56 – 1.41 (m, 2H), 1.35 – 1.22 (m, 3H), 1.21 (s, 6H), 1.20 (s, 6H), 0.88 (t, *J* = 7.2 Hz, 3H); ^{13}C NMR (126 MHz, $CDCl_3$) δ 201.4, 198.6, 156.1, 136.9, 133.0, 130.2, 128.8, 128.3, 83.6, 75.5, 64.6, 45.7, 39.9, 38.2, 36.1, 25.7, 25.0, 24.7, 23.4, 14.2; HRMS (ESI) calcd for $C_{26}H_{35}BO_5Na$ $[M+Na]^+$: 461.2475; found: 461.2484; $[\alpha]^{20}_D$ = -97.80° (*c* 2.34, $CHCl_3$); 97:03 *er*; Chiral HPLC analysis of the product: Daicel Chiralpak IA 250X4.6 mm 5μ column; hexane/2-propanol = 80/20, detected at 244 nm, Flow rate = 1 mL/min, Retention times: 5.073 min (major), 4.165 min (minor).

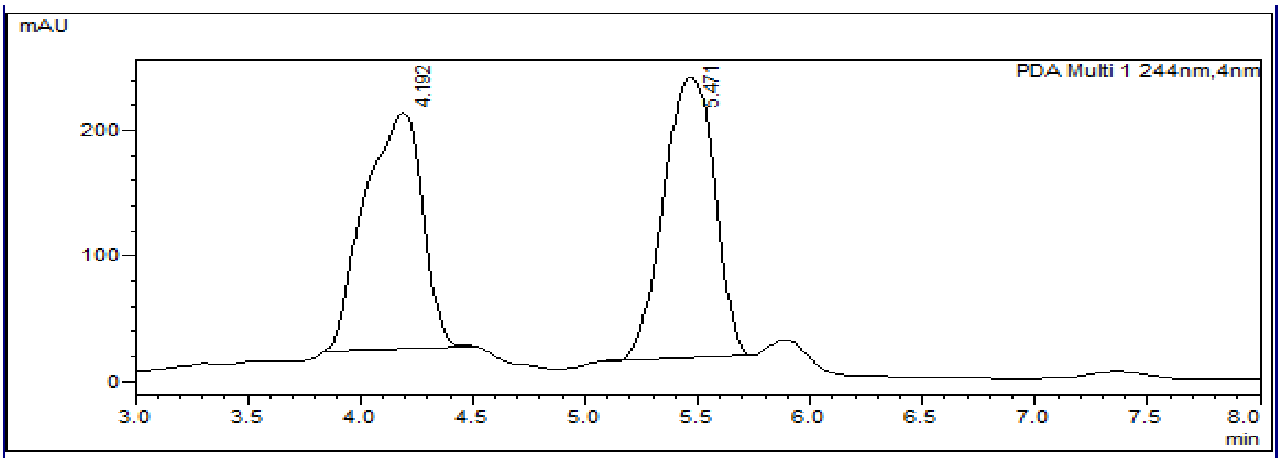

<Peak Table>

PDA Ch1 244nm

Peak#	Ret. Time	Area	Height	Area%	Height%
1	4.192	3369494	188443	48.815	45.788
2	5.471	3533067	223112	51.185	54.212
Total		6902560	411556	100.000	100.000

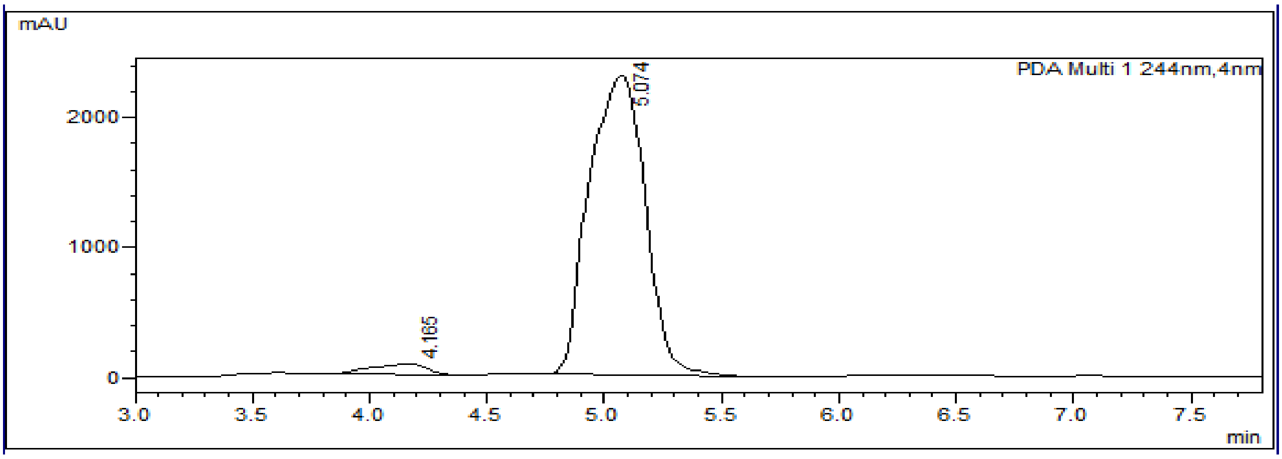

<Peak Table>

PDA Ch1 240nm

Peak#	Ret. Time	Area	Height	Area%	Height%
1	4.165	1497508	92719	2.995	2.736
2	5.073	48503496	3295709	97.005	97.264
Total		50001004	3388428	100.000	100.000

(3S,4R,4aS,8aR)-4-Benzoyl-8a-pentyl-3-(4,4,5,5-tetramethyl-1,3,2-dioxaborolan-2-yl)-3,4,4a,8a-tetrahydro-2*H*-chromen-6(5*H*)-one (2e):

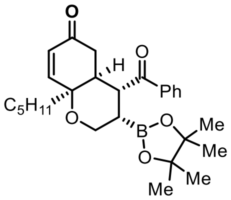

Prepared according to the general procedure as described above in 90% yield (122 mg) with >20:1*dr*. It was purified by flash chromatography (20% EtOAc/hexanes; R_f = 0.5) to afford a brown semi solid; ^{1}H NMR (500 MHz, $CDCl_3$) δ 7.75 (dd, *J* = 8.2, 0.9 Hz, 2H), 7.56 – 7.48 (m, 1H), 7.45 – 7.25 (m, 2H), 6.72 (dd, *J* = 10.3, 1.5 Hz, 1H), 6.06 (d, *J* = 10.3 Hz, 1H), 3.98 (dd, *J* = 11.5, 3.3 Hz, 1H), 3.86 (dd, *J* = 11.5, 3.1 Hz, 1H), 3.57 (dd, *J* = 9.9, 4.8 Hz, 1H), 3.17 – 3.03 (m, 1H), 2.62 (dd, *J* = 17.6, 5.0 Hz, 1H), 2.54 (dd, *J* = 17.6, 3.8 Hz, 1H), 1.79 – 1.65 (m, 2H), 1.57 – 1.47 (m, 2H), 1.46 – 1.37 (m, 1H), 1.33 – 1.23 (m, 4H), 1.21 (s, 6H), 1.19 (s, 6H), 0.87 (t, *J* = 7.0 Hz, 3H); ^{13}C NMR (101 MHz, $CDCl_3$) δ 201.4, 198.6, 156.1, 136.9, 133.0, 130.1, 128.8, 128.3, 83.5, 75.5, 64.6, 45.7, 39.9, 38.5, 36.1, 32.5, 25.0, 24.7, 23.2, 22.8, 14.2; HRMS (ESI) calcd for $C_{27}H_{37}BO_5Na$ $[M+Na]^+$: 475.2632; found: 475.2640; $[\alpha]^{20}_D$ = -94.57° (*c* 2.56, $CHCl_3$); 93:07 *er*; Chiral HPLC analysis of the product: Daicel Chiralpak IA 250X4.6 mm 5μ column; hexane/2-propanol = 95/05, detected at 254 nm, Flow rate = 1 mL/min, Retention times: 8.628 min (major), 5.936 min (minor).

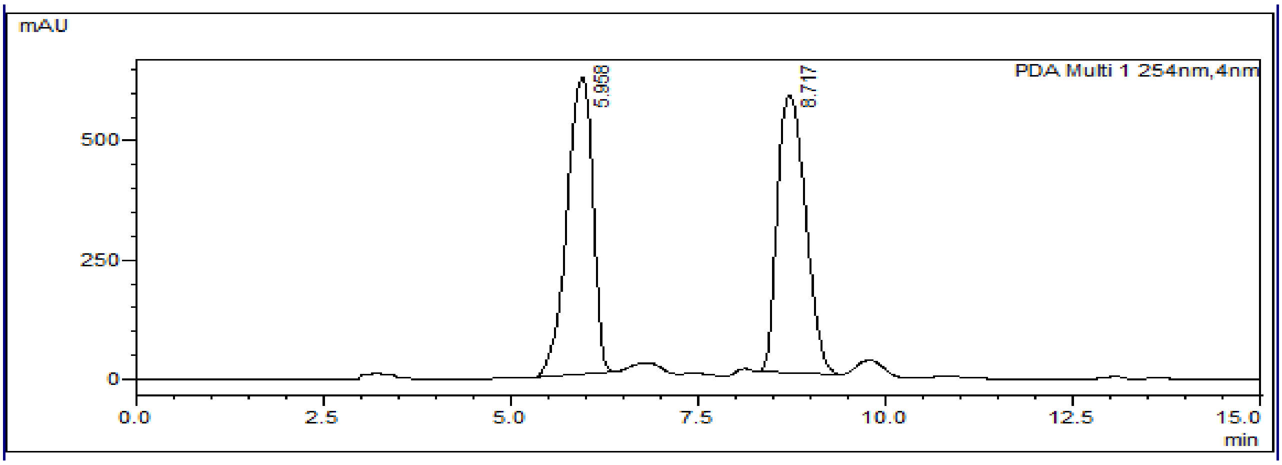

<Peak Table>

PDA Ch1 254nm

Peak#	Ret. Time	Area	Height	Area%	Height%
1	5.958	14977465	621898	49.528	51.610
2	8.717	15262976	583105	50.472	48.390
Total		30240442	1205002	100.000	100.000

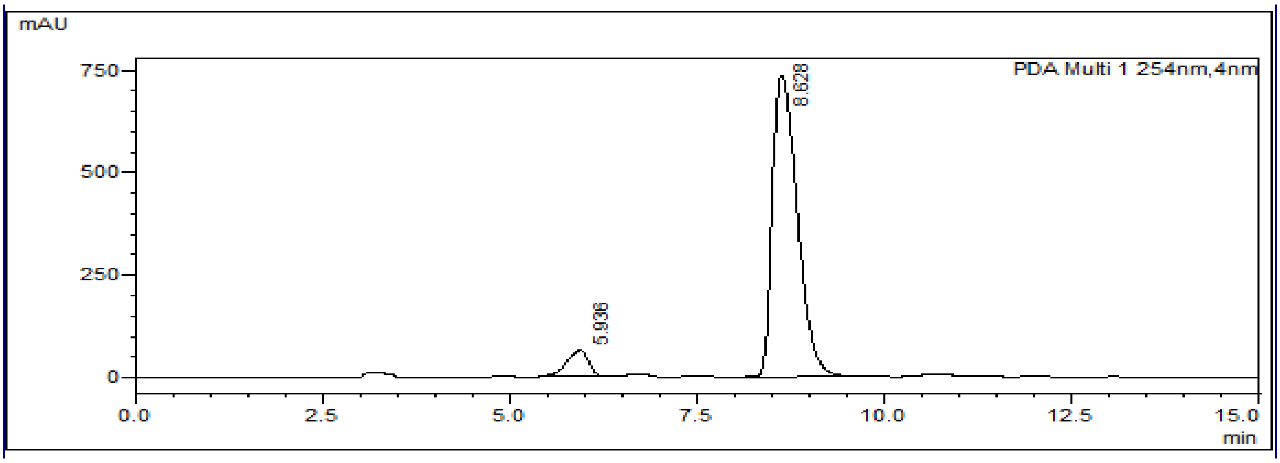

<Peak Table>

PDA Ch1 254nm

Peak#	Ret. Time	Area	Height	Area%	Height%
1	5.936	1258992	62225	6.862	7.805
2	8.628	17088006	735036	93.138	92.195
Total		18346998	797261	100.000	100.000

(3S,4R,4aS,8aR)-4-Benzoyl-8a-(2-methoxyethyl)-3-(4,4,5,5-tetramethyl-1,3,2-dioxaborolan-2-yl)-3,4,4a,8a-tetrahydro-2*H*-chromen-6(5*H*)-one (2f):

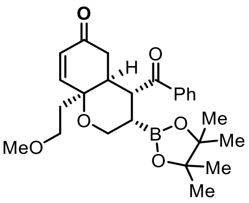

Prepared according to the general procedure as described above in 72% yield (95 mg) with 5:1*dr*. It was purified by flash chromatography (20% EtOAc/hexanes; R_f = 0.2) to afford a brown semi solid;

^{1}H NMR (400 MHz, $CDCl_3$) δ 7.76 (dd, *J* = 8.2, 1.1 Hz, 2H), 7.56 – 7.49 (m, 1H), 7.45 – 7.37 (m, 2H), 6.77 (dd, *J* = 10.4, 1.9 Hz, 1H), 6.09 (dd, *J* = 10.3, 0.9 Hz, 1H), 3.96 (dd, *J* = 11.5, 2.6 Hz, 1H), 3.86 (dd, *J* = 11.6, 3.0 Hz, 1H), 3.71 (dd, *J* = 6.7, 2.9 Hz, 1H), 3.67 – 3.56 (m, 2H), 3.32 (s, 3H), 3.14 – 3.06 (m, 1H), 2.70 (dd, *J* = 17.8, 5.1 Hz, 1H), 2.47 (dd, *J* = 17.8, 2.4 Hz, 1H), 2.12 – 2.00 (m, 2H), 1.48 (dt, *J* = 5.0, 2.7 Hz, 1H), 1.23 (s, 6H), 1.22 (s, 6H); ^{13}C NMR (101 MHz, $CDCl_3$) δ 201.3, 198.4, 155.4, 136.9, 133.2, 130.4, 128.8, 128.3, 83.6, 75.1, 68.0, 64.8, 58.7, 45.3, 39.9, 38.5, 37.2, 25.0, 24.7; HRMS (ESI) calcd for $C_{25}H_{34}BO_6$ $[M+H]^+$: 441.2448; found:441.2457; $[\alpha]^{20}_D$ = -56.92° (*c* 1.72, $CHCl_3$); 93:07 *er*; Chiral HPLC analysis of the product: Daicel Chiralpak OD-H 250X4.6 mm 5μ column; hexane/2-propanol = 95/05, detected at 230 nm, Flow rate = 1 mL/min, Retention times: 10.087 min (major), 8.248 min (minor).

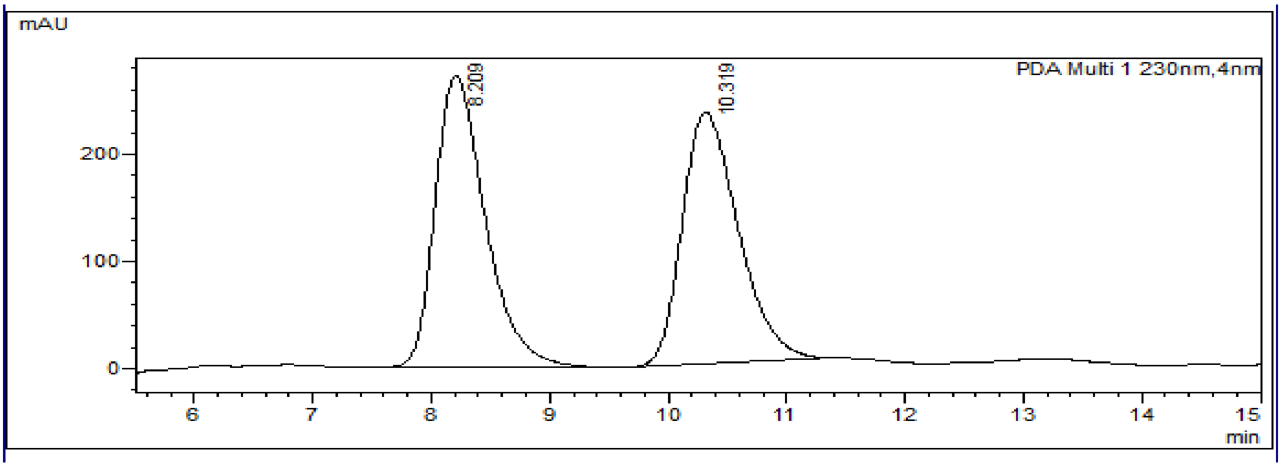

<Peak Table>

PDA Ch1 230nm

Peak#	Ret. Time	Area	Height	Area%	Height%
1	8.209	7993633	271296	50.770	53.653
2	10.319	7751054	234350	49.230	46.347
Total		15744687	505646	100.000	100.000

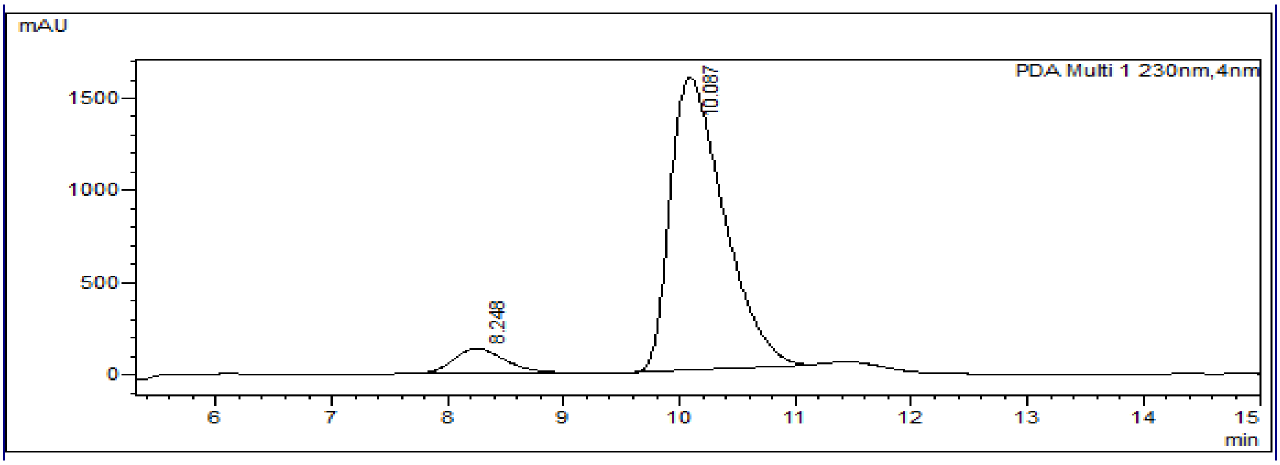

<Peak Table>

PDA Ch1 230nm

Peak#	Ret. Time	Area	Height	Area%	Height%
1	8.248	3957886	132622	7.183	7.725
2	10.087	51139866	1584265	92.817	92.275
Total		55097752	1716887	100.000	100.000

(3S,4R,4aS,8aR)-4-Benzoyl-8a-(2-((tert-butyldimethylsilyl)oxy)ethyl)-3-(4,4,5,5-tetramethyl-1,3,2-dioxaborolan-2-yl)-3,4,4a,8a-tetrahydro-2*H*-chromen-6(5*H*)-one (2g):

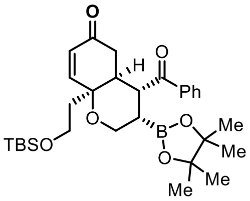

Prepared according to the general procedure as described above in 94% yield (152 mg) with >20:1*dr*. It was purified by flash chromatography (20% EtOAc/hexanes; R_f = 0.5) to afford a white solid; mp = 128–130°C; ^{1}H NMR (400 MHz, $CDCl_3$) δ 7.78 – 7.70 (m, 2H), 7.51 (t, *J* = 7.4 Hz, 1H), 7.40 (t, *J* = 7.7 Hz, 2H), 6.80 (dd, *J* = 10.4, 1.8 Hz, 1H), 6.07 (d, *J* = 10.4 Hz, 1H), 3.95 (dd, *J* = 11.5, 2.5 Hz, 1H), 3.93 – 3.80 (m, 3H), 3.60 (dd, *J* = 10.6, 4.8 Hz, 1H), 3.14 – 3.05 (m, 1H), 2.72 (dd, *J* = 17.7, 5.1 Hz, 1H), 2.48 (dd, *J* = 17.7, 2.4 Hz, 1H), 2.08 (dt, *J* = 13.9, 6.9 Hz, 1H), 2.04 – 1.95 (m, 1H), 1.47 (dt, *J* = 4.9, 2.6 Hz, 1H), 1.21 (s, 6H), 1.20 (s, 6H), 0.88 (s, 9H), 0.04 (s, 3H), 0.04 (s, 3H); ^{13}C NMR (101 MHz, $CDCl_3$) δ 201.2, 198.5, 155.8, 137.0, 133.1, 130.1, 128.8, 128.3, 83.5, 75.1, 64.8, 58.5, 45.3, 42.1, 39.9, 37.5, 26.0, 24.9, 24.7, 18.3, -5.1, -5.1; HRMS (ESI) calcd for $C_{30}H_{46}O_6SiB$ $[M+H]^+$: 541.3151; found: 541.3132; $[\alpha]^{20}_D$ = -27.90° (*c* 2.23, $CHCl_3$); 97:03 *er*; Chiral HPLC analysis of the product: Daicel Chiralpak IA 250X4.6 mm 5µ column; hexane/2-propanol = 98/02, detected at 254 nm, Flow rate = 1 mL/min, Retention times: 9.765 min (major), 6.422 min (minor).

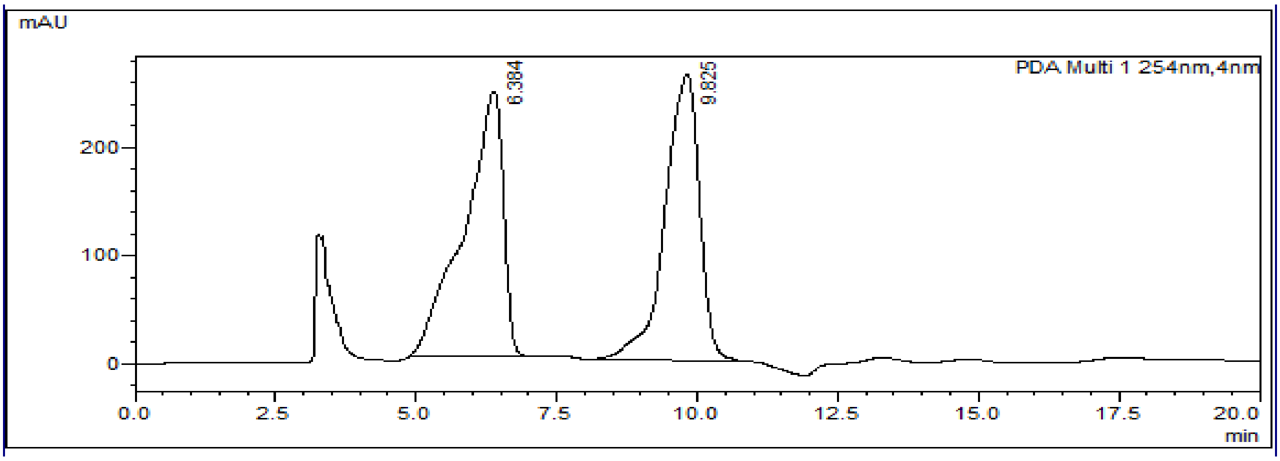

<Peak Table>

PDA Ch1 254nm

Peak#	Ret. Time	Area	Height	Area%	Height%
1	6.384	11843610	245846	50.983	48.067
2	9.825	11386904	265616	49.017	51.933
Total		23230514	511462	100.000	100.000

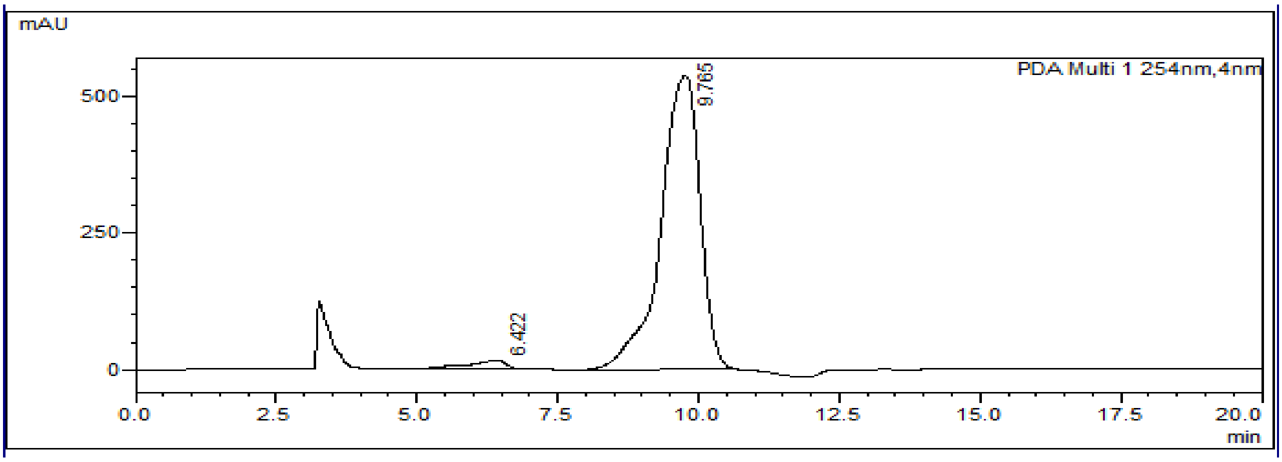

<Peak Table>

PDA Ch1 254nm

Peak#	Ret. Time	Area	Height	Area%	Height%
1	6.422	798466	15941	2.951	2.873
2	9.765	26255283	538940	97.049	97.127
Total		27053750	554881	100.000	100.000

(3S,4R,4aS,8aR)-4-Benzoyl-8a-benzyl-3-(4,4,5,5-tetramethyl-1,3,2-dioxaborolan-2-yl)-3,4,4a,8a-tetrahydro-2*H*-chromen-6(5*H*)-one (2h):

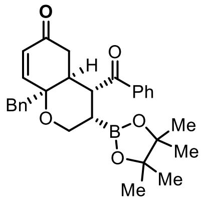

Prepared according to the general procedure as described above in 89% yield (126 mg) with 13:1*dr*. It was purified by flash chromatography (30% EtOAc/hexanes; R_f = 0.4) to afford a brown solid; mp = 138–140°C; ^{1}H NMR (400 MHz, $CDCl_3$) δ 7.74 (dd, *J* = 8.3, 1.2 Hz, 2H), 7.57 – 7.47 (m, 1H), 7.45 – 7.36 (m, 2H), 7.32 – 7.20 (m, 5H), 6.55 (dd, *J* = 10.4, 1.9 Hz, 1H), 6.12 (dd, *J* = 10.4, 1.0 Hz, 1H), 4.03 (dd, *J* = 11.5, 2.6 Hz, 1H), 3.80 (dd, *J* = 11.5, 3.0 Hz, 1H), 3.56 (dd, *J* = 10.5, 4.8 Hz, 1H), 3.13 – 3.02 (m, 1H), 3.11 (s, 2H), 2.77 (dd, *J* = 17.8, 5.2 Hz, 1H), 2.59 (ddd, *J* = 17.8, 3.0, 1.0 Hz, 1H), 1.50 (dt, *J* = 5.1, 2.7 Hz, 1H), 1.16 (s, 6H), 1.14 (s, 6H); ^{13}C NMR (101 MHz, $CDCl_3$) δ 200.9, 198.4, 155.4, 137.0, 136.0, 132.9, 131.0, 130.8, 128.7, 128.3, 128.1, 126.8, 83.5, 75.8, 65.0, 45.6, 45.4, 40.1, 38.0, 24.9, 24.6; HRMS (ESI) calcd for $C_{29}H_{34}O_5B$ $[M+H]^+$: 473.2494; found: 473.2472; $[\alpha]^{20}_D$ =

-83.23° (c 1.34, $CHCl_3$); 89:11 *er*; Chiral HPLC analysis of the product: Daicel Chiralpak IA 250X4.6 mm 5µ column; hexane/2-propanol = 97/03, detected at 254 nm, Flow rate = 1 mL/min, Retention times: 18.398 min (major), 16.309 min (minor).

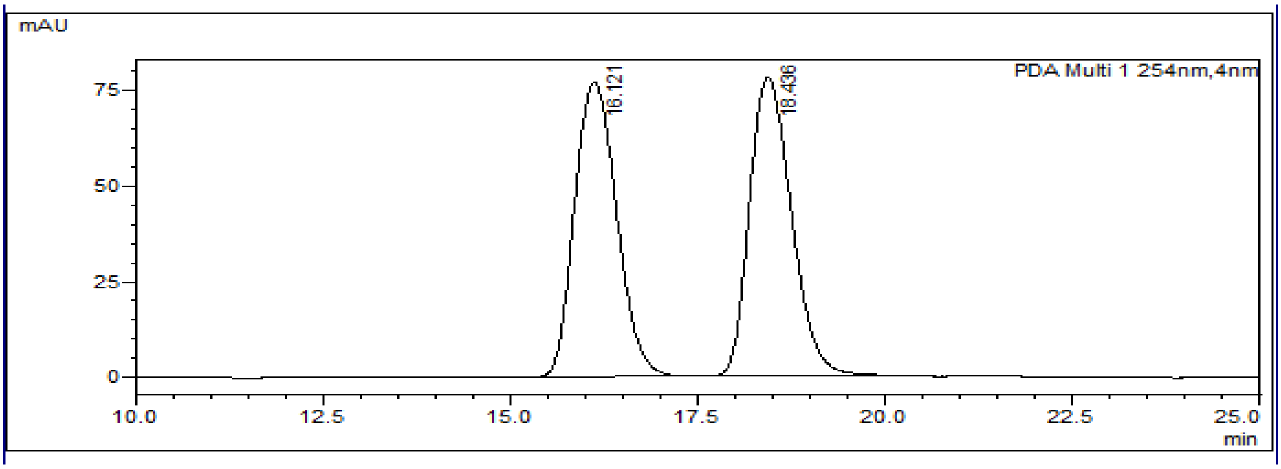

<Peak Table>

PDA Ch1 254nm

Peak#	Ret. Time	Area	Height	Area%	Height%
1	16.121	3012002	76894	49.675	49.643
2	18.436	3051365	78001	50.325	50.357
Total		6063368	154895	100.000	100.000

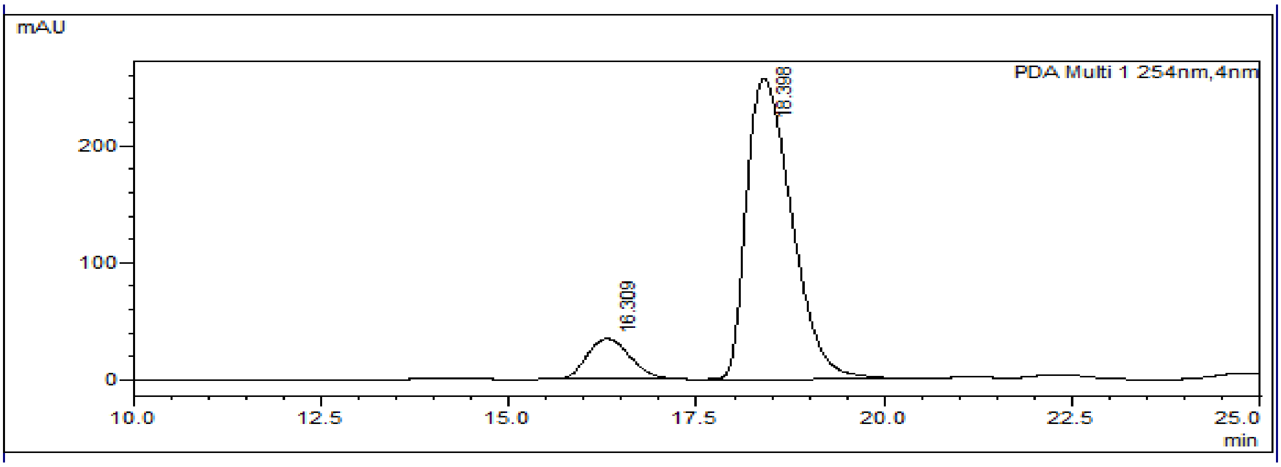

<Peak Table>

PDA Ch1 254nm

Peak#	Ret. Time	Area	Height	Area%	Height%
1	16.309	1297656	33514	10.772	11.568
2	18.398	10748928	256201	89.228	88.432
Total		12046584	289714	100.000	100.000

(3S,4R,4aS,8aR)-4-Benzoyl-8a-isopropyl-3-(4,4,5,5-tetramethyl-1,3,2-dioxaborolan-2-yl)-3,4,4a,8a-tetrahydro-2*H*-chromen-6(5*H*)-one (2i):

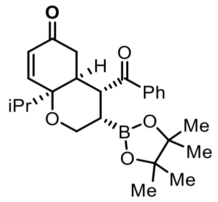

Prepared according to the general procedure as described above in 84% yield (107 mg) with 20:1*dr*. It was purified by flash chromatography (20% EtOAc/hexanes; R_f = 0.4) to afford a yellow semi solid; ^{1}H NMR (400 MHz, $CDCl_3$) δ 7.75 (dd, *J* = 9.2, 2.2 Hz, 2H), 7.59 – 7.47 (m, 1H), 7.47 – 7.35 (m, 2H), 6.86 (dd, *J* = 10.5, 1.6 Hz, 1H), 6.11 (d, *J* = 10.5 Hz, 1H), 3.97 (dd, *J* = 11.6, 3.7 Hz, 1H), 3.85 (dd, *J* = 11.6, 3.4 Hz, 1H), 3.58 (dd, *J* = 9.4, 4.9 Hz, 1H), 3.27 – 3.12 (m, 1H), 2.66 (dd, *J* = 17.8, 5.0 Hz, 1H), 2.55 (dd, *J* = 17.9, 4.0 Hz, 1H), 2.25 – 2.09 (m, 1H), 1.50 (dd, *J* = 8.1, 3.6 Hz, 1H), 1.21 (s, 6H), 1.19 (s, 6H), 1.11 (d, *J* = 6.7 Hz, 3H), 0.86 (d, *J* = 6.7 Hz, 3H); ^{13}C NMR (101 MHz, $CDCl_3$) δ 201.6, 198.5, 156.0, 136.9, 133.0, 130.4, 128.8, 128.3, 83.5, 64.1, 46.1, 39.6, 35.3, 34.6, 25.0, 24.6, 17.3, 17.1; HRMS (ESI) calcd for $C_{25}H_{33}BO_5Na$ $[M+Na]^+$: 447.2319; found: 447.2328; $[\alpha]^{20}_D$ = -116.91° (*c* 0.5, $CHCl_3$); 91:09 *er*; Chiral HPLC analysis of the product: Daicel Chiralpak IA 250X4.6 mm 5µ column; hexane/2-propanol = 95/05, detected at 254 nm, Flow rate = 1 mL/min, Retention times: 8.857 min (major), 6.199 min (minor).

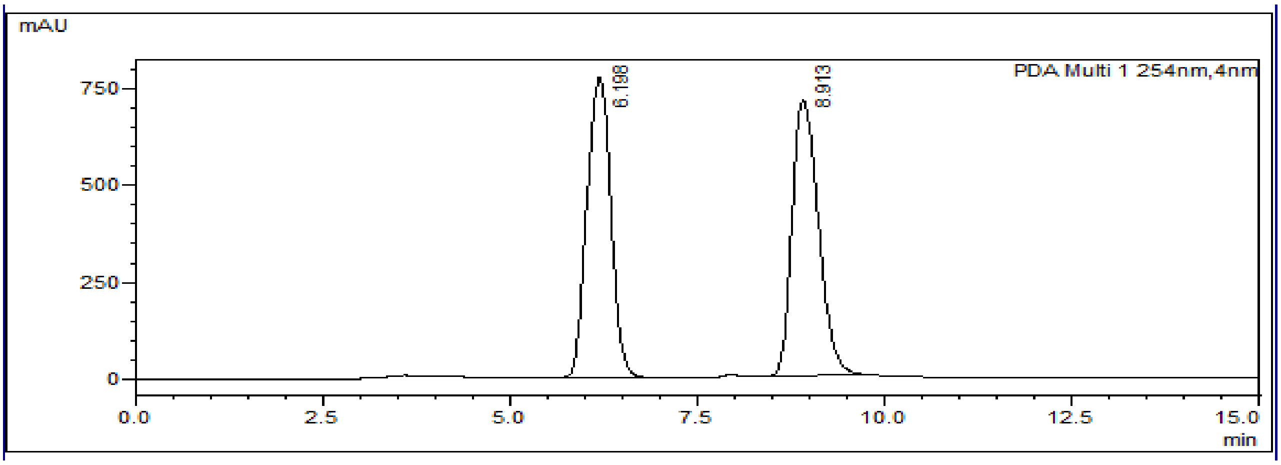

<Peak Table>

PDA Ch1 254nm

Peak#	Ret. Time	Area	Height	Area%	Height%
1	6.198	17269116	775152	49.726	52.210
2	8.913	17459560	709535	50.274	47.790
Total		34728675	1484687	100.000	100.000

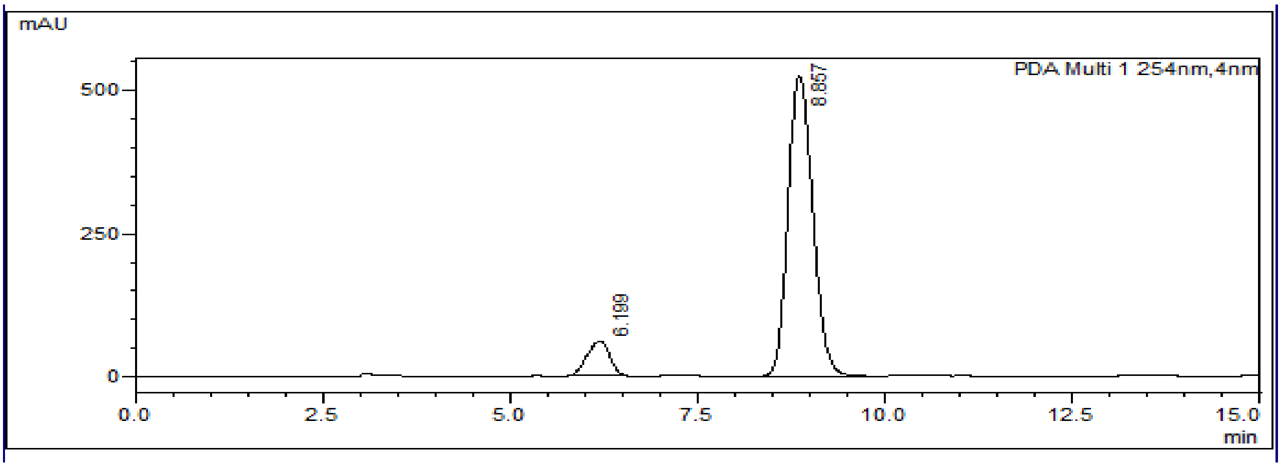

<Peak Table>

PDA Ch1 254nm

Peak#	Ret. Time	Area	Height	Area%	Height%
1	6.199	1240147	60244	9.298	10.285
2	8.857	12096962	525479	90.702	89.715
Total		13337109	585724	100.000	100.000

(3*S*,4R,4aS,8aR)-4-Benzoyl-8a-(4-pentylcyclohexyl)-3-(4,4,5,5-tetramethyl-1,3,2-dioxaborolan-2-yl)-3,4,4a,8a-tetrahydro-2*H*-chromen-6(5*H*)-one (2j):

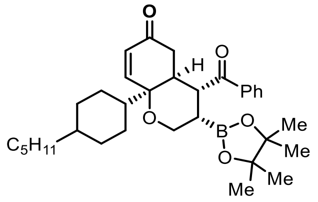

Prepared according to the general procedure as described above in 87% yield (140 mg) with >20:1*dr*. It was purified by flash chromatography (20% EtOAc/hexanes; R_f = 0.5) to afford a white solid; mp = 114–116°C; ^{1}H NMR (400 MHz, $CDCl_3$) δ 7.74 (dd, *J* = 5.2, 3.3 Hz, 2H), 7.58 – 7.47 (m, 1H), 7.47 – 7.34 (m, 2H), 6.85 (dd, *J* = 10.5, 1.3 Hz, 1H), 6.09 (d, *J* = 10.5 Hz, 1H), 3.95 (dd, *J* = 11.6, 4.0 Hz, 1H), 3.83 (dd, *J* = 11.6, 3.4 Hz, 1H), 3.56 (dd, *J* = 9.1, 4.8 Hz, 1H), 3.21 (dt, *J* = 8.5, 4.3 Hz, 1H), 2.66 (dd, *J* = 17.7, 5.0 Hz, 1H), 2.56 (dd, *J* = 17.7, 4.3 Hz, 1H), 2.00 (dt, *J* = 21.5, 8.7 Hz, 1H), 1.86 – 1.65 (m, 3H), 1.50 (dd, *J* = 8.1, 3.8 Hz, 1H), 1.46 – 1.07 (m, 14H), 1.21 (s, 6H), 1.19 (s, 6H), 0.87 (t, *J* = 7.0 Hz, 3H); ^{13}C NMR (101 MHz, $CDCl_3$) δ 201.6, 198.7, 156.0, 136.8, 133.0, 130.1, 128.8, 128.3, 83.5, 76.8, 63.9, 46.1, 44.6, 39.7, 37.5, 37.4, 34.6, 33.3, 32.3, 27.1, 26.7, 26.3, 25.0, 24.7, 22.8, 14.2; HRMS (ESI) calcd for $C_{33}H_{48}BO_5$ $[M+H]^+$: 535.3595; found: 535.3594; $[\alpha]^{20}_D$ = -107.23° (*c* 1.71, $CHCl_3$); 91:09 *er*; Chiral HPLC analysis of the product: Daicel Chiralpak IA 250X4.6 mm 5μ column; hexane/2-propanol = 98/02, detected at 254 nm, Flow rate = 1 mL/min, Retention times: 11.657 min (major), 7.548 min (minor).

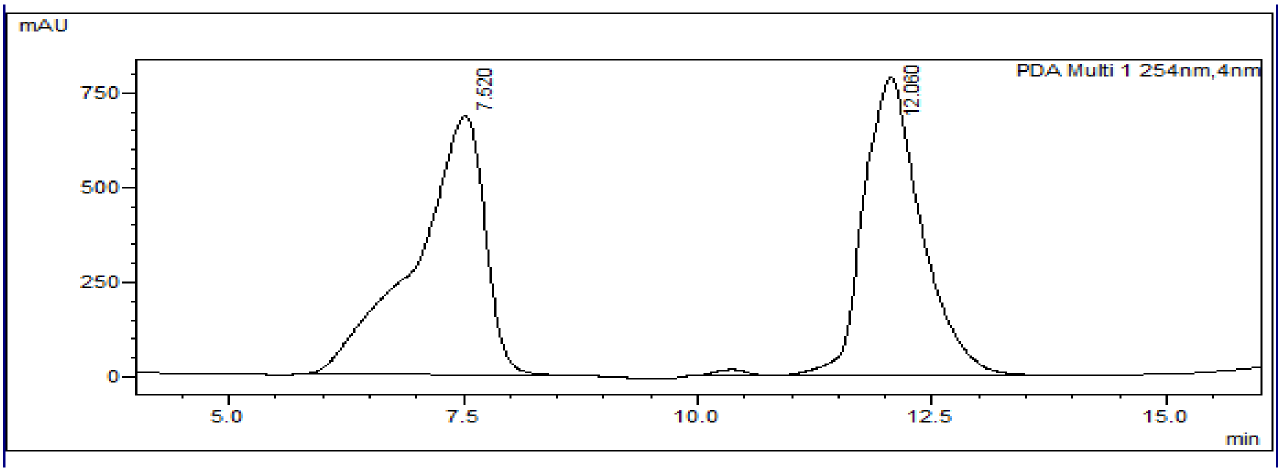

<Peak Table>

PDA Ch1 254nm

Peak#	Ret. Time	Area	Height	Area%	Height%
1	7.520	35375860	684167	50.989	46.482
2	12.060	34003191	787716	49.011	53.518
Total		69379051	1471883	100.000	100.000

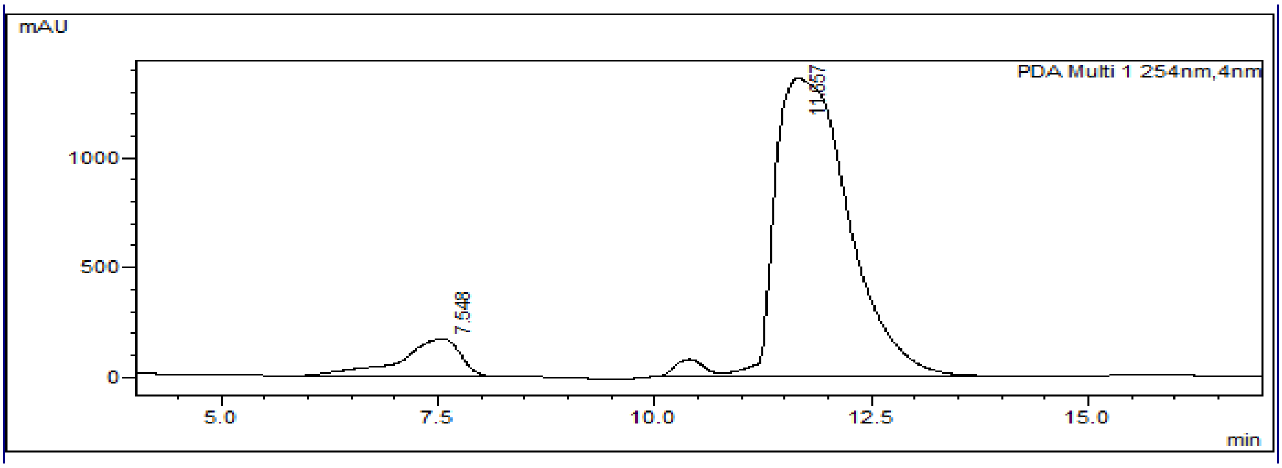

<Peak Table>

PDA Ch1 254nm

Peak#	Ret. Time	Area	Height	Area%	Height%
1	7.548	8188969	168457	9.096	11.014
2	11.657	81843477	1361004	90.904	88.986
Total		90032446	1529461	100.000	100.000

(3S,4R,4aS,8aR)-4-Benzoyl-8a-(*sec*-butyl)-3-(4,4,5,5-tetramethyl-1,3,2-dioxaborolan-2-yl)-3,4,4a,8a-tetrahydro-2*H*-chromen-6(5*H*)-one (2k):

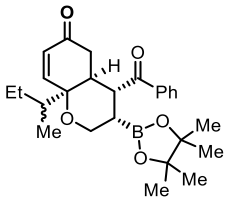

Prepared according to the general procedure as described above in 89% yield (117 mg) with 1:1*dr* with respect to methyl center at secondary butyl group. It was purified by flash chromatography (20% EtOAc/hexanes; R_f = 0.4) to afford a brown semi solid; ^{1}H NMR (400 MHz, $CDCl_3$) δ 7.79 – 7.72 (m, 2H), 7.56 – 7.49 (m, 1H), 7.45 – 7.38 (m, 2H), 6.87 (dd, *J* = 10.5, 1.2 Hz, 0.5 H), 6.83 (dd, *J* = 10.5, 1.6 Hz, 0.5 H), 6.12 (d, *J* = 4.7 Hz, 0.5 H), 6.09 (d, *J* = 4.7 Hz, 0.5 H), 3.96 (ddd, *J* = 11.6, 4.0, 2.3 Hz, 1H), 3.85 (ddd, *J* = 11.6, 4.6, 3.5 Hz, 1H), 3.61 – 3.53 (m, 1H), 3.29 – 3.14 (m, 1H), 2.70 – 2.51 (m, 2H), 2.06 – 1.74 (m, 2H), 1.74 – 1.55 (m, 1H), 1.54 – 1.46 (m, 1H), 1.22 (s, 3H), 1.21 (s, 3H), 1.19 (s, 6H), 1.08 (d, *J* = 6.7 Hz, 1.5 H), 0.93 (t, *J* = 7.5 Hz, 1.5 H), 0.85 (d, *J* = 6.7 Hz, 1.5 H), 0.79 (t, *J* = 7.4 Hz, 1.5 H); ^{13}C NMR (101 MHz, $CDCl_3$) δ 201.71, 201.57, 198.70, 198.67, 155.97, 155.61, 136.94, 136.72, 133.10, 132.98, 130.34, 130.23, 128.80, 128.34, 128.29, 83.55, 77.65, 64.12, 63.82, 46.17, 46.13, 41.54, 41.43, 39.78, 39.71, 35.27, 34.86, 25.08, 25.03, 24.67, 24.59, 24.00, 23.35, 13.60, 12.76, 12.70; HRMS (ESI) calcd for $C_{26}H_{36}BO_5$ $[M+H]^+$: 439.2656; found: 439.2678; $[\alpha]^{20}_{D}$ = -111.26° (*c* 0.78, $CHCl_3$); 94:06 *er*; Chiral HPLC analysis of the product: Daicel Chiralpak IA 250X4.6 mm 5μ column; hexane/2-propanol = 95/05, detected at 254 nm, Flow rate = 1 mL/min, Retention times: 8.450 min (major), 6.191 min (minor).

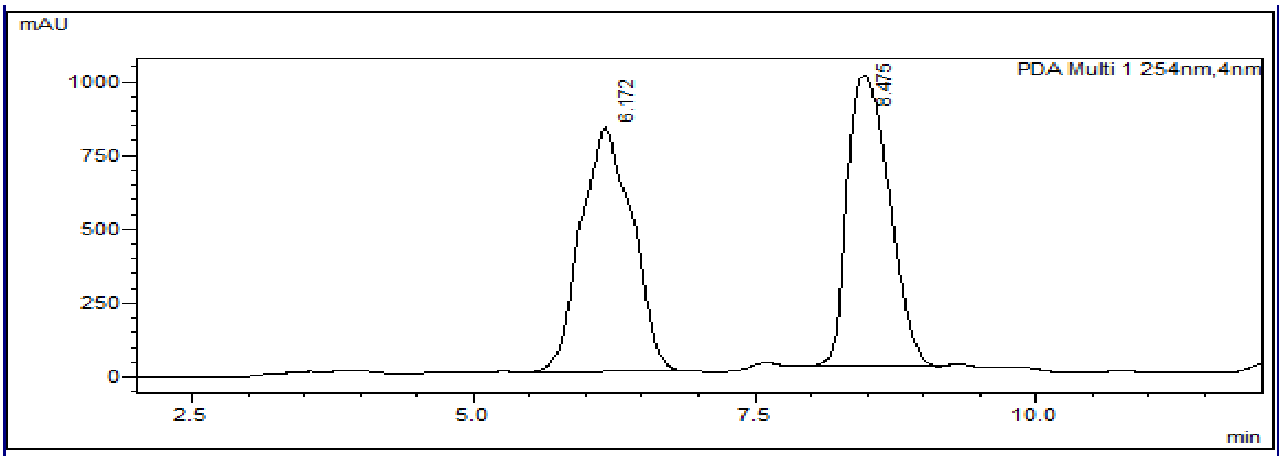

<Peak Table>

PDA Ch1 254nm

Peak#	Ret. Time	Area	Height	Area%	Height%
1	6.172	26230884	823466	50.453	45.564
2	8.475	25759629	983816	49.547	54.436
Total		51990513	1807282	100.000	100.000

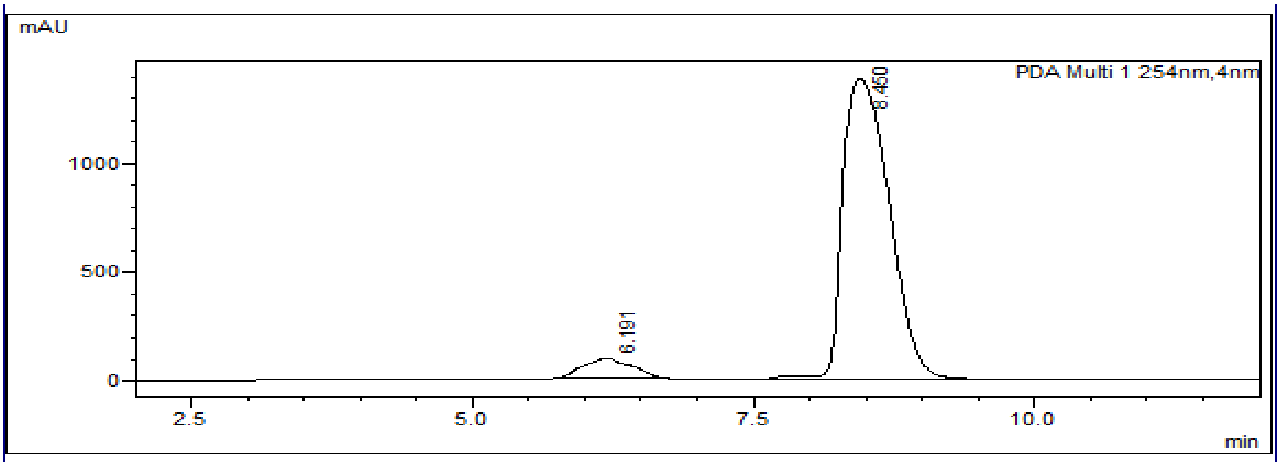

<Peak Table>

PDA Ch1 254nm

Peak#	Ret. Time	Area	Height	Area%	Height%
1	6.191	2651469	91545	6.210	6.213
2	8.450	40046250	1381906	93.790	93.787
Total		42697719	1473451	100.000	100.000

(3S,4R,4aS,8aR)-4-Benzoyl-8a-(*tert*-butyl)-3-(4,4,5,5-tetramethyl-1,3,2-dioxaborolan-2-yl)-3,4,4a,8a-tetrahydro-2*H*-chromen-6(5*H*)-one (2l):

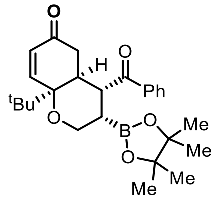

Prepared according to the general procedure as described above in 74% yield (97 mg) with >20:1*dr*. It was purified by flash chromatography (20% EtOAc/hexanes; R_f = 0.4) to afford an orange semi solid; ^{1}H NMR (500 MHz, $CDCl_3$) δ 7.74 (dd, *J* = 8.3, 1.2 Hz, 2H), 7.52 – 7.48 (m, 1H), 7.44 – 7.38 (m, 2H), 6.77 (dd, *J* = 10.6, 2.0 Hz, 1H), 6.29 (dd, *J* = 10.6, 1.0 Hz, 1H), 3.99 (dd, *J* = 11.2, 2.0 Hz, 1H), 3.80 (dd, *J* = 11.2, 3.1 Hz, 1H), 3.49 (dd, *J* = 10.7, 5.0 Hz, 1H), 3.40 – 3.29 (m, 1H), 2.91 (dd, *J* = 19.0, 6.0 Hz, 1H), 2.27 (dt, *J* = 19.1, 1.2 Hz, 1H), 1.49 – 1.42 (m, 1H), 1.17 (s, 12H), 1.10 (s, 9H); ^{13}C NMR (101 MHz, $CDCl_3$) δ 200.9, 198.8, 154.5, 137.3, 132.8, 132.5, 128.7, 128.3, 83.6, 79.2, 64.7, 48.4, 41.9, 41.7, 33.1, 26.8, 25.3, 24.5; HRMS (ESI) calcd for $C_{26}H_{35}BO_5Na$ $[M+Na]^+$: 461.2475;

found: 461.2483; $[\alpha]^{20}_{D}$ = -50.32° (*c* 0.5, $CHCl_3$); 78:22 *er*; Chiral HPLC analysis of the product: Daicel Chiralpak IA 250X4.6 mm 5μ column; hexane/2-propanol = 85/15, detected at 240 nm, Flow rate = 1 mL/min, Retention times: 6.193 min (major), 4.368 min (minor).

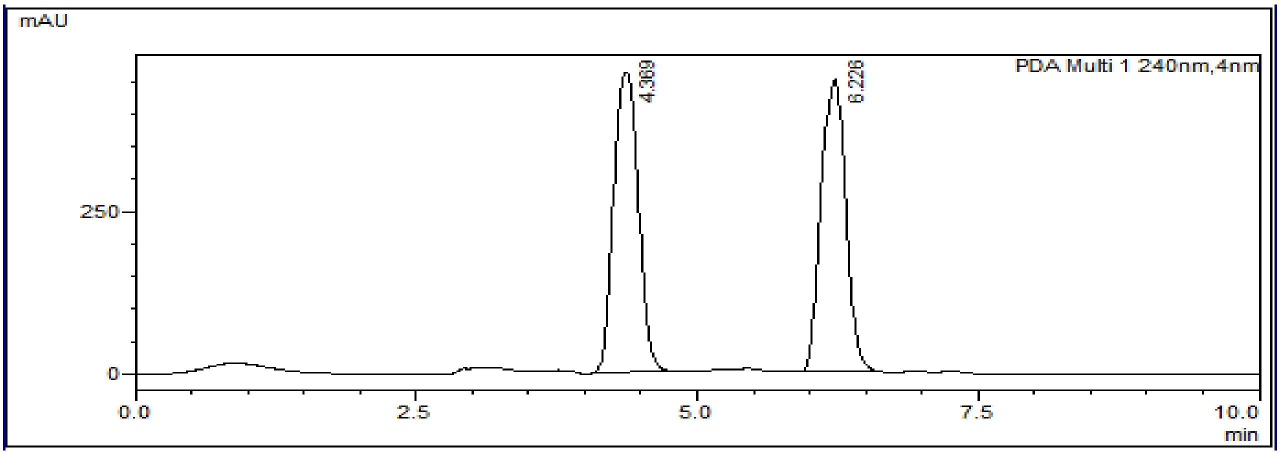

<Peak Table>

PDA Ch1 240nm

Peak#	Ret. Time	Area	Height	Area%	Height%
1	4.369	7061288	460654	50.650	50.719
2	6.226	6880098	447602	49.350	49.281
Total		13941386	908255	100.000	100.000

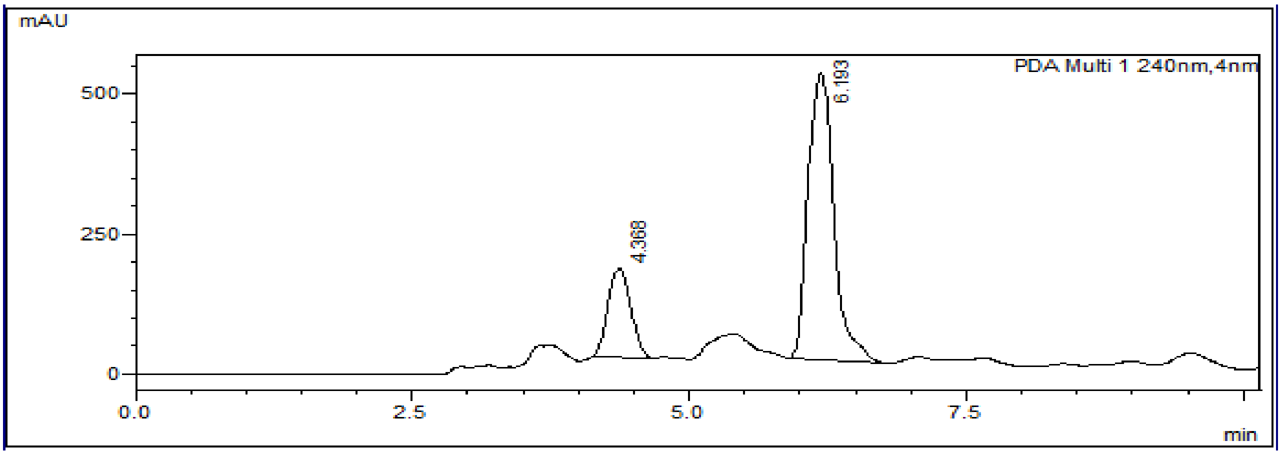

<Peak Table>

PDA Ch1 240nm

Peak#	Ret. Time	Area	Height	Area%	Height%
1	4.368	2278177	157963	21.543	23.578
2	6.193	8296881	512010	78.457	76.422
Total		10575059	669973	100.000	100.000

(3S,4R,4aS,8aR)-4-Benzoyl-8a-phenyl-3-(4,4,5,5-tetramethyl-1,3,2-dioxaborolan-2-yl)-3,4,4a,8a-tetrahydro-2*H*-chromen-6(5*H*)-one (2m):

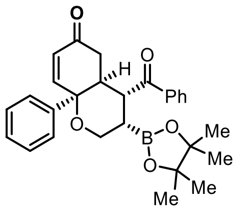

Prepared according to the general procedure as described above in 96% yield (132 mg) with >20:1*dr*. It was purified by flash chromatography (20% EtOAc/hexanes; R_f = 0.3) to afford a white semi solid; ^{1}H NMR (400 MHz, $CDCl_3$) δ 7.77 (dd, *J* = 8.3, 1.2 Hz, 2H), 7.68 (dd, *J* = 8.0, 1.6 Hz, 2H), 7.58 – 7.52 (m, 1H), 7.48 – 7.39 (m, 2H), 7.38 – 7.30 (m, 3H), 6.86 (dd, *J* = 10.3, 2.0 Hz, 1H), 6.44 (dd, *J* = 10.3, 0.7 Hz, 1H), 4.17 (dd, *J* = 11.7, 2.6 Hz, 1H), 4.07 (dd, *J* = 11.7, 3.0 Hz, 1H), 3.78 (dd, *J* = 10.6, 4.7 Hz, 1H), 3.31 (dtd, *J* = 10.4, 4.2, 2.0 Hz, 1H), 2.35 (qd, *J* = 17.6, 4.7 Hz, 2H), 1.61 (dt, *J* = 5.0, 2.7 Hz, 1H), 1.31 (s, 12H); ^{13}C NMR (101 MHz, $CDCl_3$) δ 200.9, 198.7, 152.6, 141.4, 136.8, 133.3, 132.3, 128.8, 128.4, 128.4, 127.0, 83.7, 78.7, 65.2, 46.0, 41.4, 39.4, 25.1, 24.8; HRMS (ESI) calcd for $C_{28}H_{32}BO_5$ $[M+H]^+$: 459.2343; found: 459.2349; $[\alpha]^{20}_D$ = -55.72° (*c* 2.74, $CHCl_3$); 93:07 *er*; Chiral HPLC analysis of the product: Daicel Chiralpak IA 250X4.6 mm 5μ column; hexane/2-propanol = 95/05, detected at 254 nm, Flow rate = 1 mL/min, Retention times: 11.742 min (major), 8.120 min (minor).

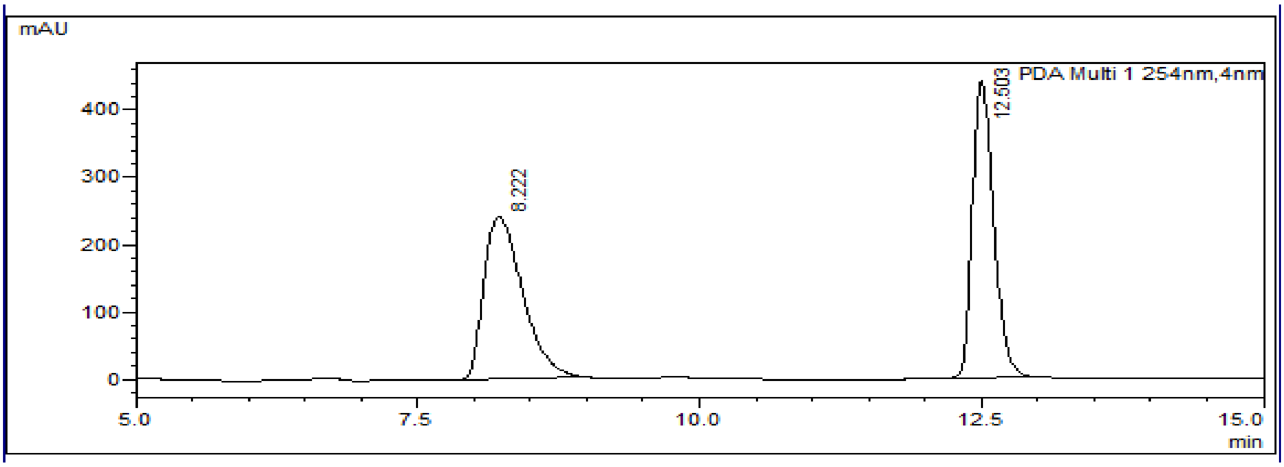

<Peak Table>

PDA Ch1 254nm

Peak#	Ret. Time	Area	Height	Area%	Height%
1	8.222	5798362	241457	49.858	35.308
2	12.503	5831299	442410	50.142	64.692
Total		11629661	683868	100.000	100.000

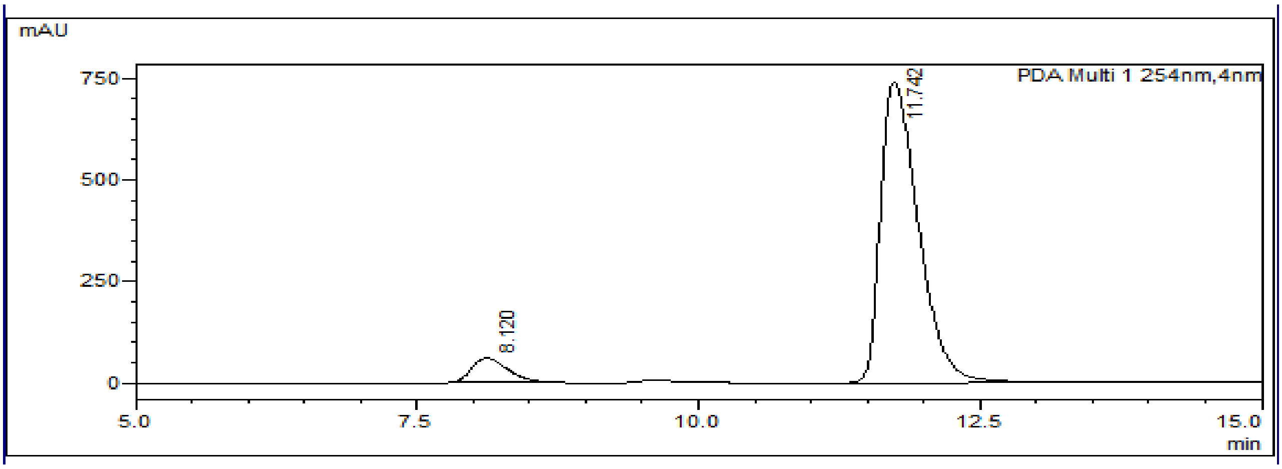

<Peak Table>

PDA Ch1 254nm

Peak#	Ret. Time	Area	Height	Area%	Height%
1	8.120	1290295	59022	6.919	7.385
2	11.742	17358732	740153	93.081	92.615
Total		18649027	799175	100.000	100.000

(3S,4R,4aS,8aR)-4-Benzoyl-8a-(4-bromophenyl)-3-(4,4,5,5-tetramethyl-1,3,2-dioxaborolan-2-yl)-3,4,4a,8a-tetrahydro-2*H*-chromen-6(5*H*)-one (2n):

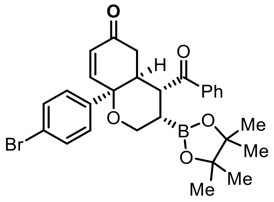

Prepared according to the general procedure as described above in 89% yield (143 mg) with 10:1*dr*. It was purified by flash chromatography (20% EtOAc/hexanes; R_f = 0.3) to afford a orange solid; mp = 136–138°C; ^{1}H NMR (400 MHz, $CDCl_3$) δ 7.76 (dd, *J* = 5.2, 3.3 Hz, 2H), 7.63 – 7.52 (m, 3H), 7.50 – 7.39 (m, 4H), 6.81 (dd, *J* = 10.3, 2.0 Hz, 1H), 6.43 (d, *J* = 10.3 Hz, 1H), 4.13 (dd, *J* = 11.7, 2.5 Hz, 1H), 4.04 (dd, *J* = 11.7, 2.9 Hz, 1H), 3.76 (dd, *J* = 10.7, 4.7 Hz, 1H), 3.23 (dtd, *J* = 10.3, 4.0, 2.0 Hz, 1H), 2.32 (dd, *J* = 3.7, 1.9 Hz, 2H), 1.59 (dt, *J* = 4.8, 2.6 Hz, 1H), 1.30 (s, 12H); ^{13}C NMR (101 MHz, $CDCl_3$) δ 200.8, 198.3, 151.9, 140.7, 136.6, 133.4, 132.6, 131.5, 128.8, 128.4, 122.5, 83.8, 78.3, 65.1, 46.0, 41.4, 39.3, 25.1, 24.8; HRMS (ESI) calcd for $C_{28}H_{31}BO_5Br$ $[M+H]^+$: 537.1448; found: 537.1465; $[\alpha]^{20}_D$ = -226.80° (*c* 0.82, $CHCl_3$); 93:07 *er*; Chiral HPLC analysis of the product: Daicel Chiralpak IA 250X4.6 mm 5μ column; hexane/2-propanol = 95/05, detected at 254 nm, Flow rate = 1 mL/min, Retention times: 10.624 min (major), 8.613 min (minor).

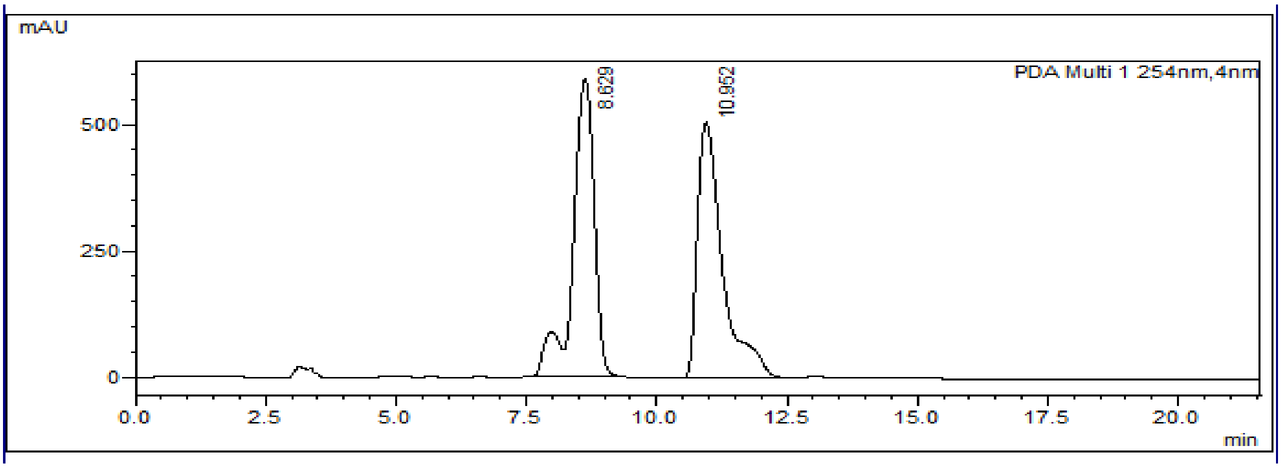

<Peak Table>

PDA Ch1 254nm

Peak#	Ret. Time	Area	Height	Area%	Height%
1	8.629	17084652	589446	50.036	53.826
2	10.952	17059844	505647	49.964	46.174
Total		34144496	1095093	100.000	100.000

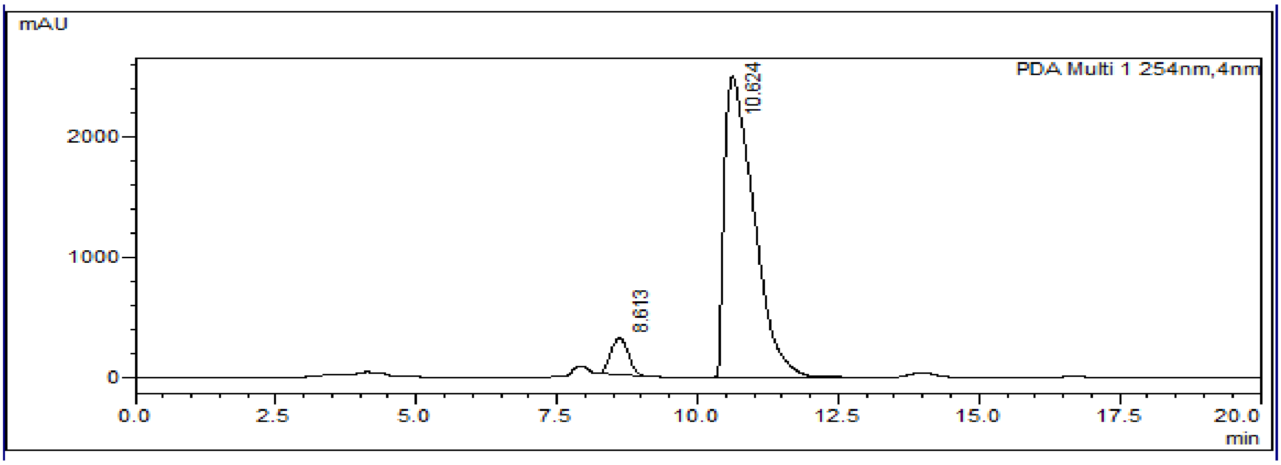

<Peak Table>

PDA Ch1 254nm

Peak#	Ret. Time	Area	Height	Area%	Height%
1	8.613	6609231	305171	6.757	10.877
2	10.624	91203884	2500497	93.243	89.123
Total		97813115	2805668	100.000	100.000

(3S,4R,4aS,8aR)-4-Benzoyl-8a-methoxy-3-(4,4,5,5-tetramethyl-1,3,2-dioxaborolan-2-yl)-3,4,4a,8a-tetrahydro-2*H*-chromen-6(5*H*)-one (2o):

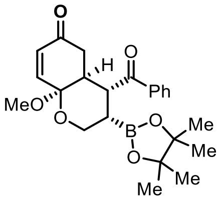

Prepared according to the general procedure as described above in 79% yield (98 mg) with 16:1*dr*. It was purified by flash chromatography (20% EtOAc/hexanes; R_f = 0.2) to afford a brown semi solid; ^{1}H NMR (500 MHz, $CDCl_3$) δ 7.66 (dd, *J* = 5.2, 3.3 Hz, 2H), 7.55 – 7.48 (m, 1H), 7.47 – 7.38 (m, 2H), 6.72 (d, *J* = 10.3 Hz, 1H), 5.94 (dd, *J* = 10.3, 1.0 Hz, 1H), 4.03 (t, *J* = 12.2 Hz, 1H), 3.90 (dd, *J* = 11.8, 5.3 Hz, 1H), 3.37 (d, *J* = 4.4 Hz, 1H), 3.19 (s, 3H), 3.00 (dd, *J* = 16.1, 13.9 Hz, 1H), 2.68 (ddd, *J* = 13.7, 3.8, 0.9 Hz, 1H), 2.57 (ddd, *J* = 16.1, 4.0, 1.0 Hz, 1H), 1.67 (dt, *J* = 12.6, 4.9 Hz, 1H), 1.28 (s, 6H), 1.18 (s, 6H); ^{13}C NMR (101 MHz, $CDCl_3$) δ 202.0, 199.1, 143.4, 137.3, 132.2, 129.6, 128.7, 127.9, 94.8, 83.5, 60.8, 47.6, 46.4, 41.1, 39.5, 25.0, 24.7; HRMS (ESI) calcd for $C_{23}H_{29}BO_6Na$ $[M+Na]^+$: 435.1955; found: 435.1963; $[\alpha]^{20}_D$ = -53.64° (*c* 0.59, $CHCl_3$); 72:28 *er*; Chiral HPLC analysis of the product: Daicel Chiralpak OD-H 250X4.6 mm 5μ column; hexane/2-propanol = 95/05, detected at 240 nm, Flow rate = 1 mL/min, Retention times: 15.231 min (major), 12.503 min (minor).

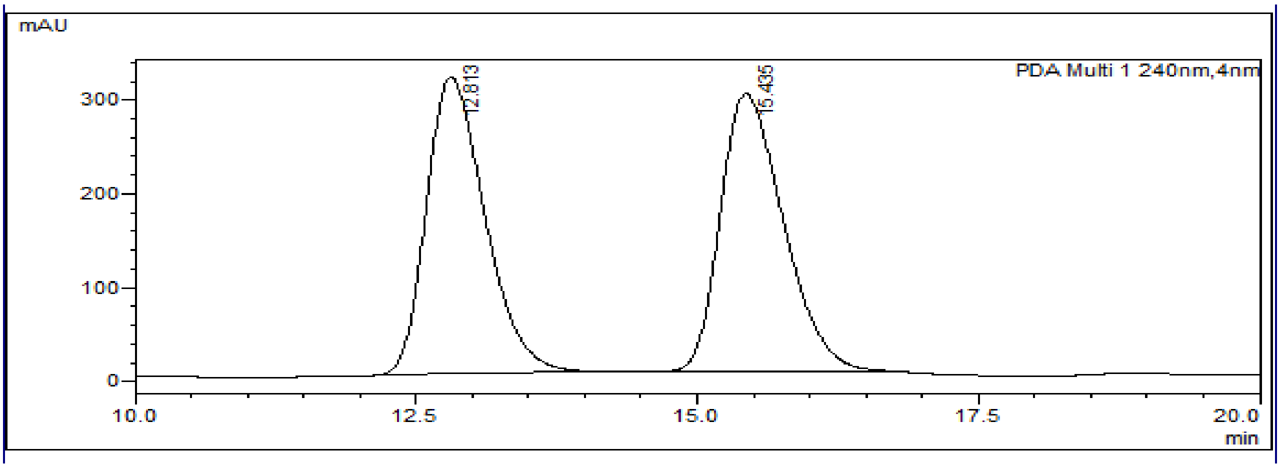

<Peak Table>

PDA Ch1 240nm

Peak#	Ret. Time	Area	Height	Area%	Height%
1	12.813	11484156	316394	49.807	51.614
2	15.435	11573077	296607	50.193	48.386
Total		23057233	613001	100.000	100.000

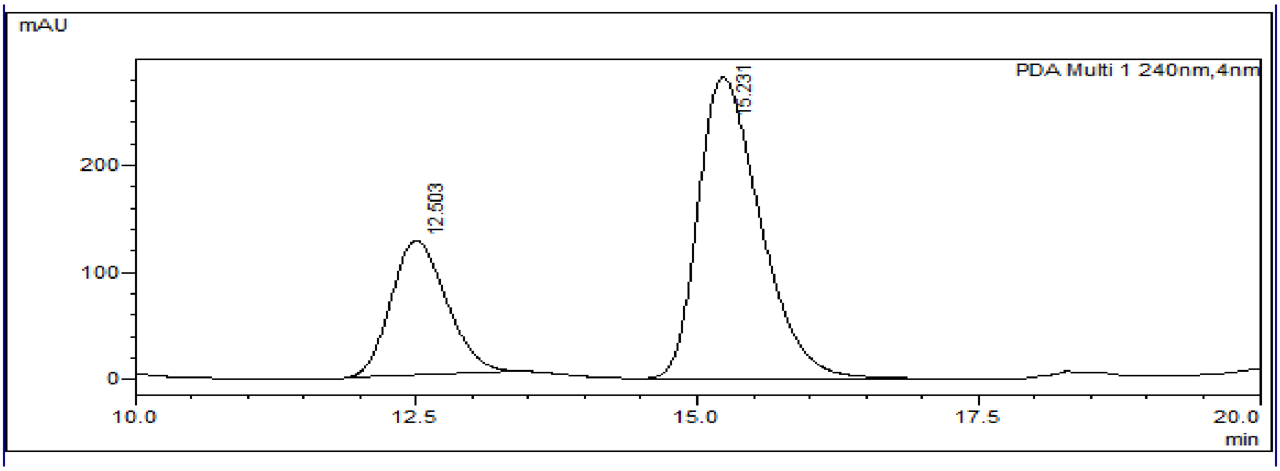

<Peak Table>

PDA Ch1 240nm

Peak#	Ret. Time	Area	Height	Area%	Height%
1	12.503	4308535	125601	28.440	30.858
2	15.231	10841001	281432	71.560	69.142
Total		15149536	407033	100.000	100.000

(3S,4R,4aS,8aR)-4-Benzoyl-8a-ethoxy-3-(4,4,5,5-tetramethyl-1,3,2-dioxaborolan-2-yl)-3,4,4a,8a-tetrahydro-2*H*-chromen-6(5*H*)-one (2p):

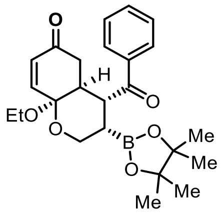

Prepared according to the general procedure as described above in 83% yield (106 mg) with >20:1*dr*. It was purified by flash chromatography (30% EtOAc/hexanes; R_f = 0.3) to afford a brown semi solid; ^{1}H NMR (400 MHz, $CDCl_3$) δ 7.68 (dd, *J* = 8.3, 1.3 Hz, 2H), 7.54 – 7.47 (m, 1H), 7.47 – 7.38 (m, 2H), 6.74 (d, *J* = 10.3 Hz, 1H), 5.93 (dd, *J* = 10.3, 1.0 Hz, 1H), 4.07 (dd, *J* = 22.1, 10.1 Hz, 1H), 3.88 (dd, *J* = 11.7, 5.2 Hz, 1H), 3.56 (dq, *J* = 8.9, 7.1 Hz, 1H), 3.46 (dq, *J* = 9.0, 7.0 Hz, 1H), 3.37 (d, *J* = 4.4 Hz, 1H), 2.99 (dd, *J* = 16.0, 13.9 Hz, 1H), 2.69 (ddd, *J* = 10.2, 3.7 0.9 Hz, 1H), 2.56 (ddd, *J* = 16.0, 4.0, 1.1 Hz, 1H), 1.66 (dt, *J* = 12.6, 4.9 Hz, 1H), 1.28 (s, 6H), 1.18 (s, 6H), 1.10 (t, *J* = 7.0 Hz, 3H); ^{13}C NMR (101 MHz, $CDCl_3$) δ 201.8, 199.1, 143.9, 137.2, 132.3, 129.4, 128.7, 128.2, 94.7, 83.5, 60.7, 55.8, 46.4, 41.1, 39.3, 25.0, 24.7, 14.8; HRMS (ESI) calcd for $C_{24}H_{32}BO_6$ $[M+H]^+$: 427.2286; found: 427.2284; $[\alpha]^{20}_D$ = -73.43° (*c* 0.96, $CHCl_3$); 91:09 *er*; Chiral HPLC analysis of the product: Daicel Chiralpak IA 250X4.6 mm 5µ column; hexane/2-propanol = 96/04, detected at 254 nm, Flow rate = 1 mL/min, Retention times: 6.351 min (major), 8.135 min (minor).

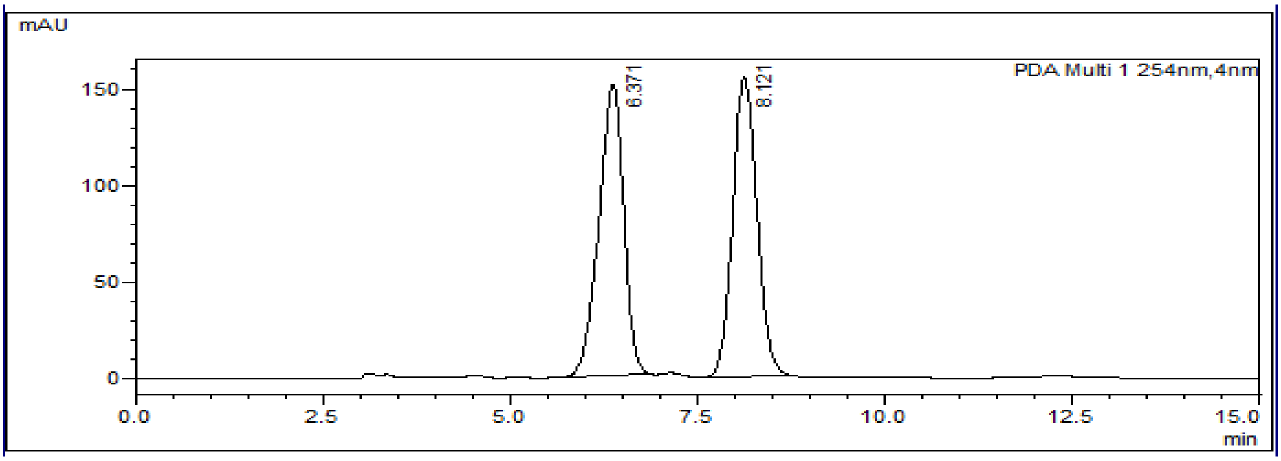

<Peak Table>

PDA Ch1 254nm

Peak#	Ret. Time	Area	Height	Area%	Height%
1	6.371	3458407	150317	49.729	49.209
2	8.121	3496032	155150	50.271	50.791
Total		6954439	305468	100.000	100.000

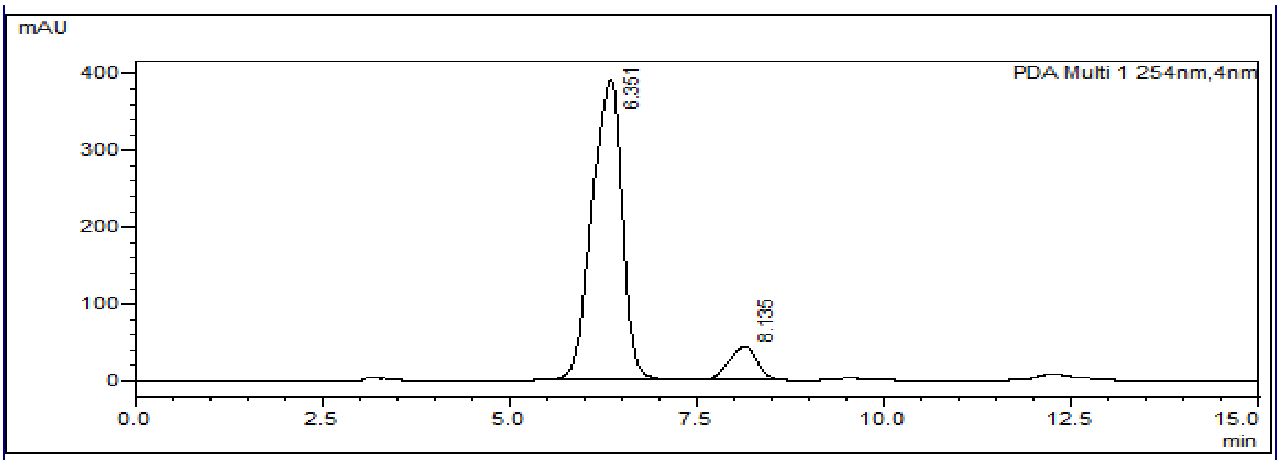

<Peak Table>

PDA Ch1 254nm

Peak#	Ret. Time	Area	Height	Area%	Height%
1	6.351	10838163	390138	90.598	89.950
2	8.135	1124705	43592	9.402	10.050
Total		11962869	433730	100.000	100.000

(3S,4R,4aS,8aS)-4-Benzoyl-8a-(benzyloxy)-3-(4,4,5,5-tetramethyl-1,3,2-dioxaborolan-2-yl)-3,4,4a,8a-tetrahydro-2*H*-chromen-6(5*H*)-one (2q):

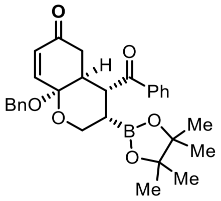

Prepared according to the general procedure as described above in 87% yield (127 mg) with >20:1*dr*. It was purified by flash chromatography (20% EtOAc/hexanes; R_f = 0.2) to afford a brown semi solid; ^{1}H NMR (500 MHz, $CDCl_3$) δ 7.48 – 7.39 (m, 3H), 7.36 – 7.27 (m, 5H), 7.12 (dd, *J* = 7.7, 1.7 Hz, 2H), 6.74 (d, *J* = 10.3 Hz, 1H), 5.92 (dd, *J* = 10.3, 1.0 Hz, 1H), 4.59 – 4.53 (m, 2H), 4.22 (t, *J* = 12.2 Hz, 1H), 3.99 (dd, *J* = 11.8, 5.2 Hz, 1H), 3.39 (d, *J* = 4.3 Hz, 1H), 3.01 (dd, *J* = 16.1, 13.9 Hz, 1H), 2.78 (ddd, *J* = 13.8, 3.8, 1.1 Hz, 1H), 2.59 (ddd, *J* = 16.1, 4.0, 1.0 Hz, 1H), 1.71 (dt, *J* = 12.6, 4.9 Hz, 1H), 1.29 (s, 6H), 1.19 (s, 6H); ^{13}C NMR (101 MHz, $CDCl_3$) δ 201.7, 198.9, 143.9, 137.7, 137.1, 132.0, 129.5, 128.5, 128.4, 128.1, 127.8, 127.7, 95.5, 83.5, 62.7, 61.2, 46.5, 41.2, 39.4, 25.0, 24.7; HRMS (ESI) calcd for $C_{29}H_{33}BO_6Na$ $[M+Na]^+$: 511.2268; found: 511.2286; $[\alpha]^{20}_D$ = -23.62° (*c* 0.92,

$CHCl_3$); 87:13 *er*; Chiral HPLC analysis of the product: Daicel Chiralpak OD-H 250X4.6 mm 5μ column; hexane/2-propanol = 95/05, detected at 254 nm, Flow rate = 1 mL/min, Retention times: 10.776 min (major), 15.007 min (minor).

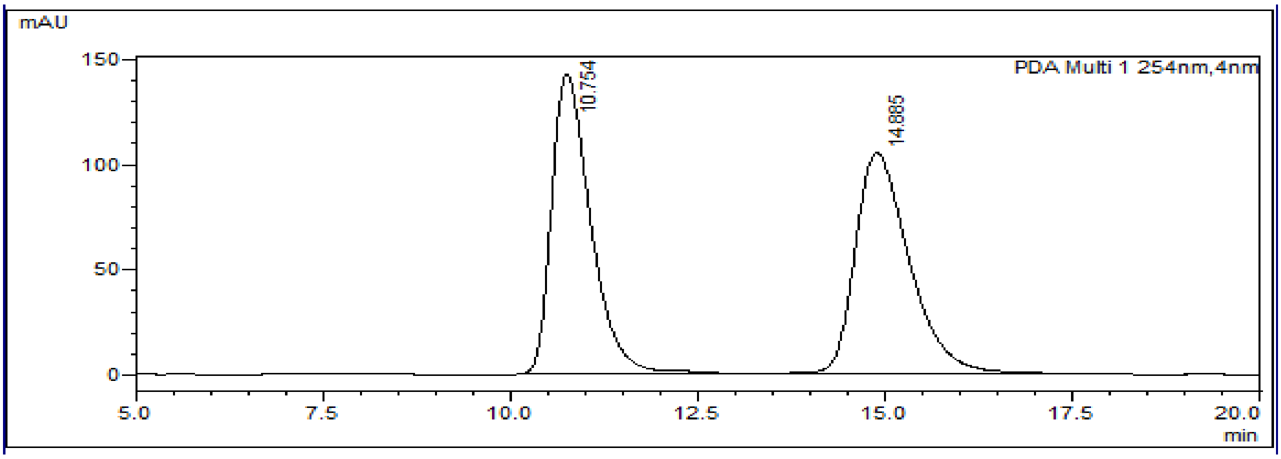

<Peak Table>

PDA Ch1 254nm

Peak#	Ret. Time	Area	Height	Area%	Height%
1	10.754	5177078	142507	49.437	57.596
2	14.885	5295009	104918	50.563	42.404
Total		10472087	247425	100.000	100.000

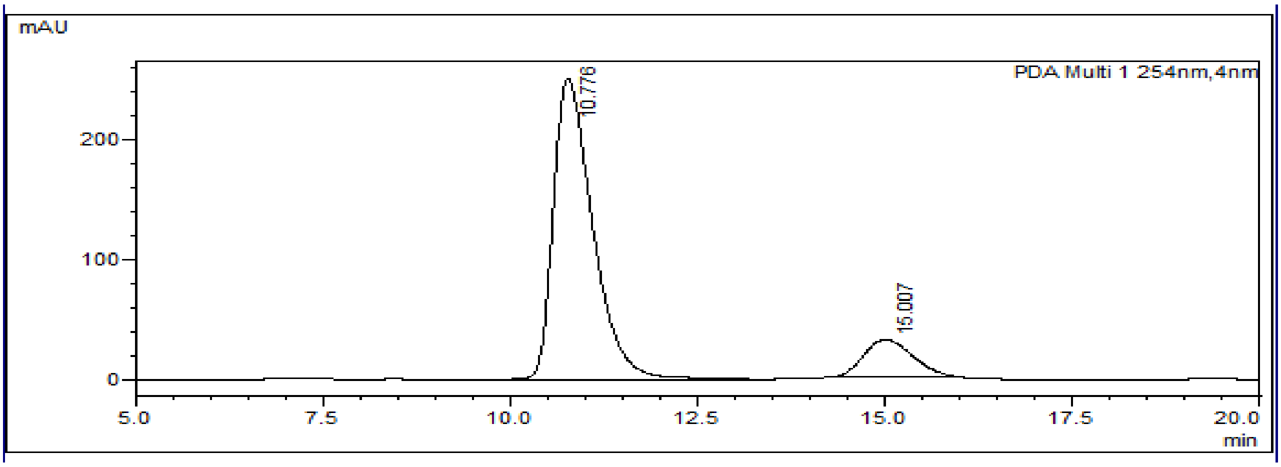

<Peak Table>

PDA Ch1 254nm

Peak#	Ret. Time	Area	Height	Area%	Height%
1	10.776	9103060	249248	86.843	88.991
2	15.007	1379194	30833	13.157	11.009
Total		10482254	280081	100.000	100.000

(3S,4R,4aS,8aR)-8a-Methyl-4-(4-methylbenzoyl)-3-(4,4,5,5-tetramethyl-1,3,2-dioxaborolan-2-yl)-3,4,4a,8a-tetrahydro-2*H*-chromen-6(5*H*)-one (2r):

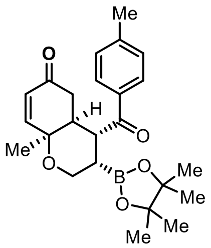

Prepared according to the general procedure as described above in 89% yield (109 mg) with 19:1*dr*. It was purified by flash chromatography (20% EtOAc/hexanes; R_f = 0.3) to afford a brown semi solid; ^{1}H NMR (500 MHz, $CDCl_3$) δ 7.67 (d, *J* = 8.2 Hz, 2H), 7.22 (d, *J* = 8.1 Hz, 2H), 6.68 (dd, *J* = 10.3, 1.4 Hz, 1H), 6.05 (d, *J* = 10.3 Hz, 1H), 4.01 (dd, *J* = 11.6, 4.3 Hz, 1H), 3.86 (dd, *J* = 11.6, 3.3 Hz, 1H), 3.53 (dd, *J* = 9.1, 4.8 Hz, 1H), 2.95 – 2.86 (m, 1H), 2.63 (t, *J* = 4.9 Hz, 2H), 2.39 (s, 3H), 1.53 (dd, *J* = 8.1, 4.1 Hz, 1H), 1.44 (s, 3H), 1.22 (s, 6H), 1.20 (s, 6H); ^{13}C NMR (101 MHz, $CDCl_3$) δ 201.3, 198.7, 155.4, 143.9, 134.3, 129.9, 129.5, 128.5, 83.5, 73.2, 64.4, 45.9, 40.3, 39.6, 25.7, 24.9, 24.8, 21.8; HRMS (ESI) calcd for $C_{24}H_{32}O_5B$ $[M+H]^+$: 411.2337; found: 411.2325; $[\alpha]^{20}_D$ = -17.70° (*c* 1.44, $CHCl_3$); 92:08 *er*; Chiral HPLC analysis of the product: Daicel Chiralpak IA 250X4.6 mm 5μ column; hexane/2-propanol = 97/03, detected at 254 nm, Flow rate = 1 mL/min, Retention times: 20.612 min (major), 11.025 min (minor).

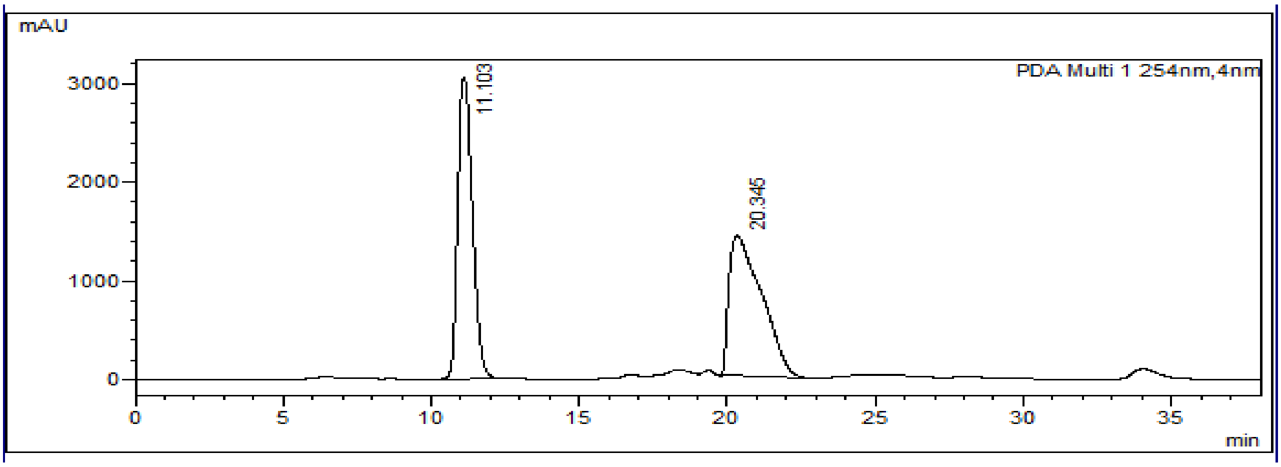

<Peak Table>

PDA Ch1 254nm

Peak#	Ret. Time	Area	Height	Area%	Height%
1	11.103	106146830	3051453	49.369	68.199
2	20.345	108860665	1422910	50.631	31.801
Total		215007495	4474363	100.000	100.000

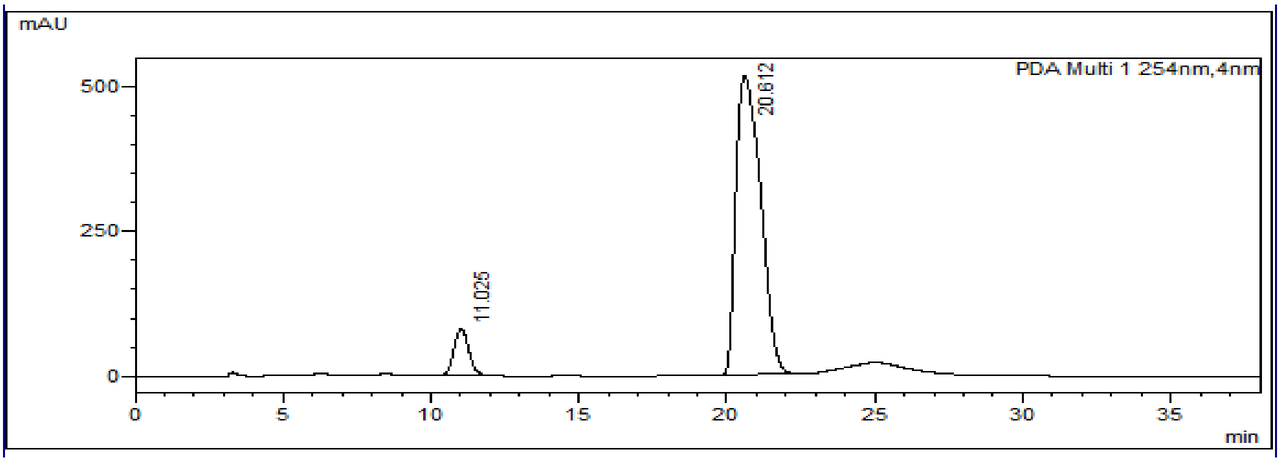

<Peak Table>

PDA Ch1 254nm

Peak#	Ret. Time	Area	Height	Area%	Height%
1	11.025	2735124	79486	8.360	13.357
2	20.612	29981709	515588	91.640	86.643
Total		32716833	595075	100.000	100.000

(3S,4R,4aS,8aR)-4-(4-(*tert*-Butyl)benzoyl)-8a-methyl-3-(4,4,5,5-tetramethyl-1,3,2-dioxaborolan-2-yl)-3,4,4a,8a-tetrahydro-2*H*-chromen-6(5*H*)-one (2s):

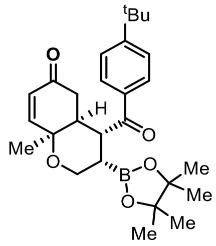

Prepared according to the general procedure as described above in 85% yield (115 mg) with 19:1*dr*. It was purified by flash chromatography (20% EtOAc/hexanes; R_f = 0.4) to afford a brown semi solid; ^{1}H NMR (400 MHz, $CDCl_3$) δ 7.71 (d, *J* = 8.6 Hz, 2H), 7.42 (d, *J* = 8.5 Hz, 2H), 6.67 (dd, *J* = 10.3, 1.4 Hz, 1H), 6.04 (d, *J* = 10.3 Hz, 1H), 4.01 (dd, *J* = 11.7, 4.6 Hz, 1H), 3.86 (dd, *J* = 11.7, 3.4 Hz, 1H), 3.54 (dd, *J* = 8.9, 4.8 Hz, 1H), 3.04 – 2.84 (m, 1H), 2.63 (d, *J* = 4.8 Hz, 2H), 1.54 (dd, *J* = 8.1, 4.5 Hz, 1H), 1.44 (s, 3H), 1.32 (s, 9H), 1.23 (s, 6H), 1.20 (s, 6H); ^{13}C NMR (126 MHz, $CDCl_3$) δ 201.3, 198.7, 156.8, 155.2, 134.1, 129.9, 128.3, 125.8, 83.5, 73.0, 64.3, 45.9, 40.3, 39.5, 35.2, 31.2, 25.6, 24.9, 24.8; HRMS (ESI) calcd for $C_{27}H_{38}BO_5$ $[M+H]^+$: 453.2812; found: 453.2827; $[\alpha]^{20}_D$ = -112.43° (*c* 2.33, $CHCl_3$); 98:02 *er*; Chiral HPLC analysis of the product: Daicel Chiralpak IA 250X4.6 mm 5μ column; hexane/2-propanol = 85/15, detected at 254 nm, Flow rate = 1 mL/min, Retention times: 6.363 min (major), 4.095 min (minor).

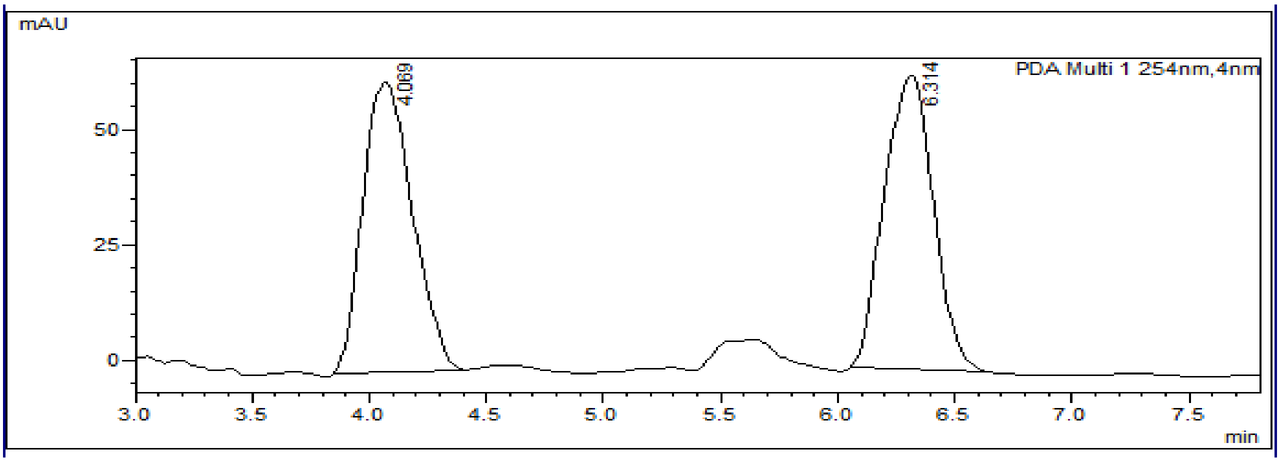

<Peak Table>

PDA Ch1 254nm

Peak#	Ret. Time	Area	Height	Area%	Height%
1	4.069	927905	62684	49.919	49.644
2	6.314	930905	63583	50.081	50.356
Total		1858810	126267	100.000	100.000

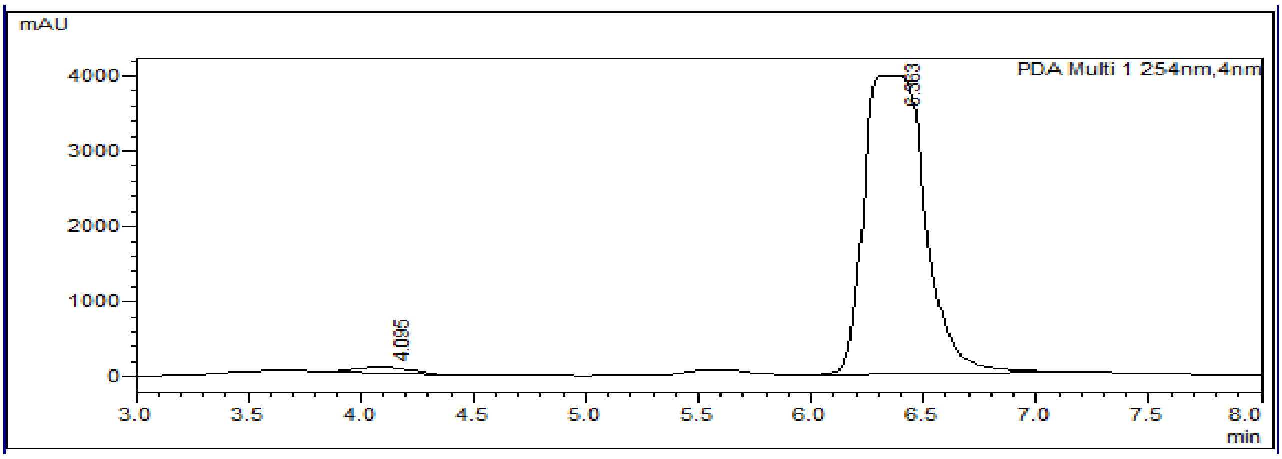

<Peak Table>

PDA Ch1 254nm

Peak#	Ret. Time	Area	Height	Area%	Height%
1	4.095	1253673	75042	1.655	1.861
2	6.363	74488869	3957521	98.345	98.139
Total		75742542	4032563	100.000	100.000

(3S,4R,4aS,8aR)-4-(4-Methoxybenzoyl)-8a-methyl-3-(4,4,5,5-tetramethyl-1,3,2-dioxaborolan-2-yl)-3,4,4a,8a-tetrahydro-2*H*-chromen-6(5*H*)-one (2t):

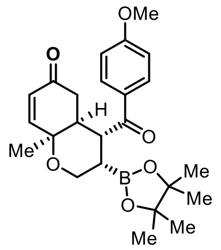

Prepared according to the general procedure as described above in 89% yield (113 mg) with >20:1*dr*. It was purified by flash chromatography (30% EtOAc/hexanes; R_f = 0.3) to afford a white solid; mp = 132–134°C; ^{1}H NMR (500 MHz, $CDCl_3$) δ 7.76 (d, *J* = 8.8 Hz, 2H), 6.89 (d, *J* = 8.8 Hz, 2H), 6.68 (dd, *J* = 10.3, 1.1 Hz, 1H), 6.03 (d, *J* = 10.3 Hz, 1H), 3.99 (dd, *J* = 11.7, 4.1 Hz, 1H), 3.87 – 3.82 (m, 1H), 3.84 (s, 3H), 3.51 (dd, *J* = 9.3, 4.8 Hz, 1H), 2.91 (dt, *J* = 8.8, 4.5 Hz, 1H), 2.63 (dd, *J* = 17.4, 5.0 Hz, 1H), 2.56 (dd, *J* = 17.4, 4.7 Hz, 1H), 1.50 (dd, *J* = 8.0, 3.9 Hz, 1H), 1.44 (s, 3H), 1.22 (s, 6H), 1.19 (s, 6H); ^{13}C NMR (101 MHz, $CDCl_3$) δ 200.0, 198.6, 163.6, 155.5, 130.6, 129.9, 129.6, 114.0, 83.4, 73.2, 64.4, 55.6, 45.6, 40.2, 39.7, 25.7, 24.9, 24.8; HRMS (ESI) calcd for $C_{24}H_{32}BO_6$ $[M+H]^+$: 427.2287; found: 427.2270; $[\alpha]^{20}_D$ = -19.32° (*c* 1.28, $CHCl_3$); 86:14 *er*; Chiral HPLC analysis of the product: Daicel Chiralpak OD-H 250X4.6 mm 5µ column; hexane/2-propanol = 93/07, detected at 244 nm, Flow rate = 1 mL/min, Retention times: 19.373 min (major), 12.823 min (minor).

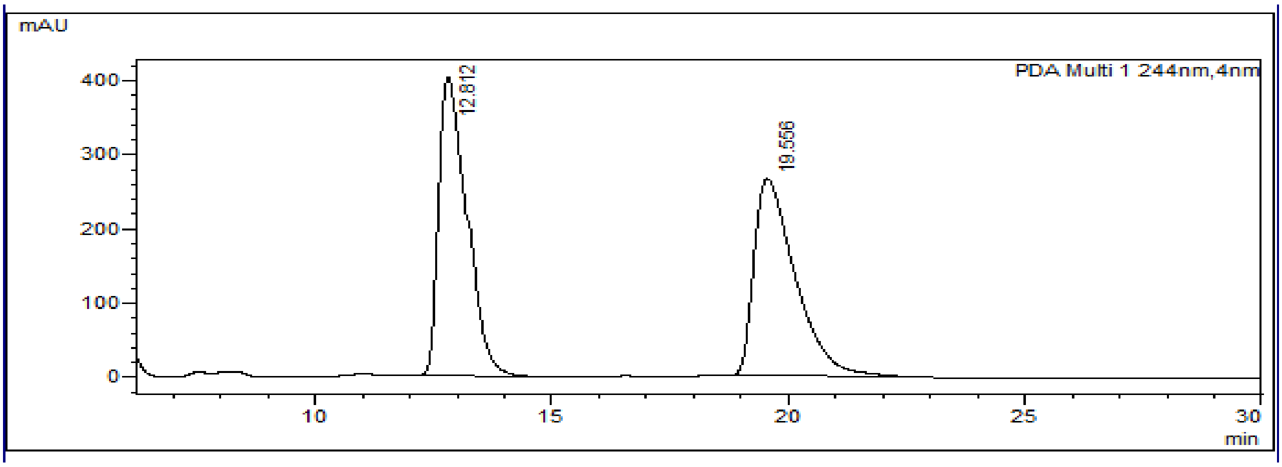

<Peak Table>

PDA Ch1 244nm

Peak#	Ret. Time	Area	Height	Area%	Height%
1	12.812	16655088	401484	50.584	60.188
2	19.556	16270689	265561	49.416	39.812
Total		32925777	667045	100.000	100.000

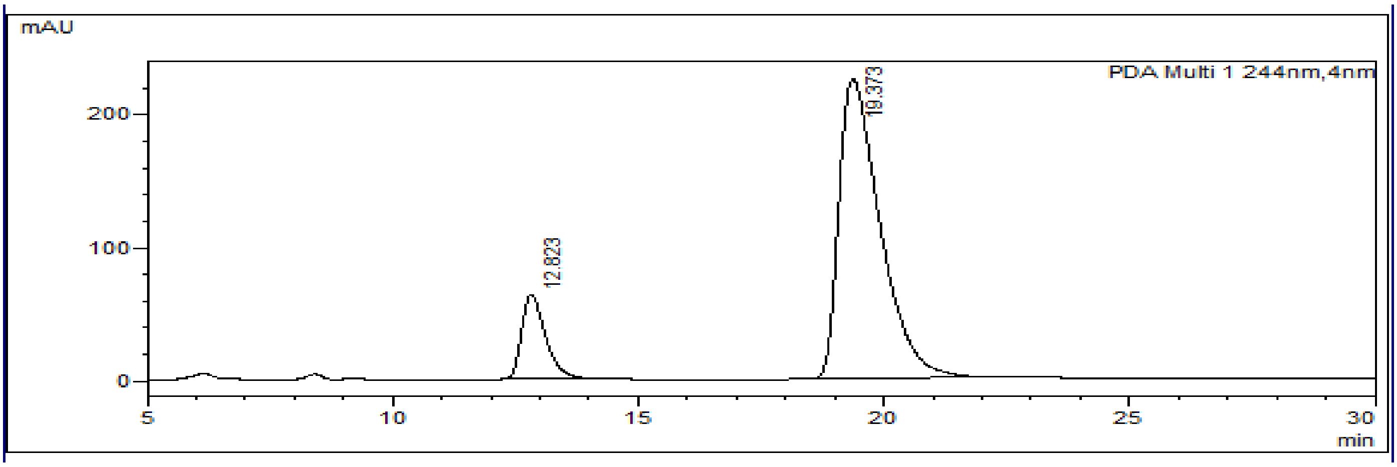

<Peak Table>

PDA Ch1 244nm

Peak#	Ret. Time	Area	Height	Area%	Height%
1	12.823	2105485	63229	13.691	21.912
2	19.373	13272915	225322	86.309	78.088
Total		15378399	288551	100.000	100.000

(3S,4R,4aS,8aR)-4-(4-Chlorobenzoyl)-8a-methyl-3-(4,4,5,5-tetramethyl-1,3,2-dioxaborolan-2-yl)-3,4,4a,8a-tetrahydro-2*H*-chromen-6(5*H*)-one (2u):

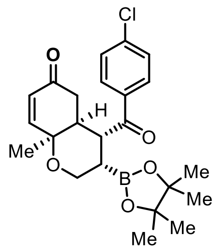

Prepared according to the general procedure as described above in 72% yield (93 mg) with 18:1*dr*. It was purified by flash chromatography (20% EtOAc/hexanes; R_f = 0.3) to afford a brown liquid; ^{1}H NMR (500 MHz, $CDCl_3$) δ 7.70 (d, *J* = 8.6 Hz, 2H), 7.39 (d, *J* = 8.5 Hz, 2H), 6.69 (dd, *J* = 10.3, 1.5 Hz, 1H), 6.05 (d, *J* = 10.3 Hz, 1H), 4.00 (dd, *J* = 11.6, 3.6 Hz, 1H), 3.83 (dd, *J* = 11.6, 3.1 Hz, 1H), 3.47 (dd, *J* = 9.7, 4.9 Hz, 1H), 2.90 (dt, *J* = 13.0, 3.9 Hz, 1H), 2.66 (dd, *J* = 17.4, 5.0 Hz, 1H), 2.56 (dd, *J* = 17.4, 4.0 Hz, 1H), 1.49 (dd, *J* = 7.9, 3.5 Hz, 1H), 1.46 (s, 3H), 1.20 (s, 6H), 1.18 (s, 6H); ^{13}C NMR (101 MHz, $CDCl_3$) δ 200.1, 198.5, 155.5, 139.5, 135.1, 130.1, 129.7, 129.1, 83.7, 73.4, 64.5, 45.8, 40.2, 39.6, 25.9, 24.9, 24.7; HRMS (ESI) calcd for $C_{23}H_{29}O_5ClB$ $[M+H]^+$: 431.1791; found: 431.1776; $[\alpha]^{20}_D$ = -78.28° (*c* 1.28, $CHCl_3$); 88:12 *er*; Chiral HPLC analysis of the product: Daicel Chiralpak IA 250X4.6 mm 5μ column; hexane/2-propanol = 97/03, detected at 254 nm, Flow rate = 1 mL/min, Retention times: 16.123 min (major), 10.129 min (minor).

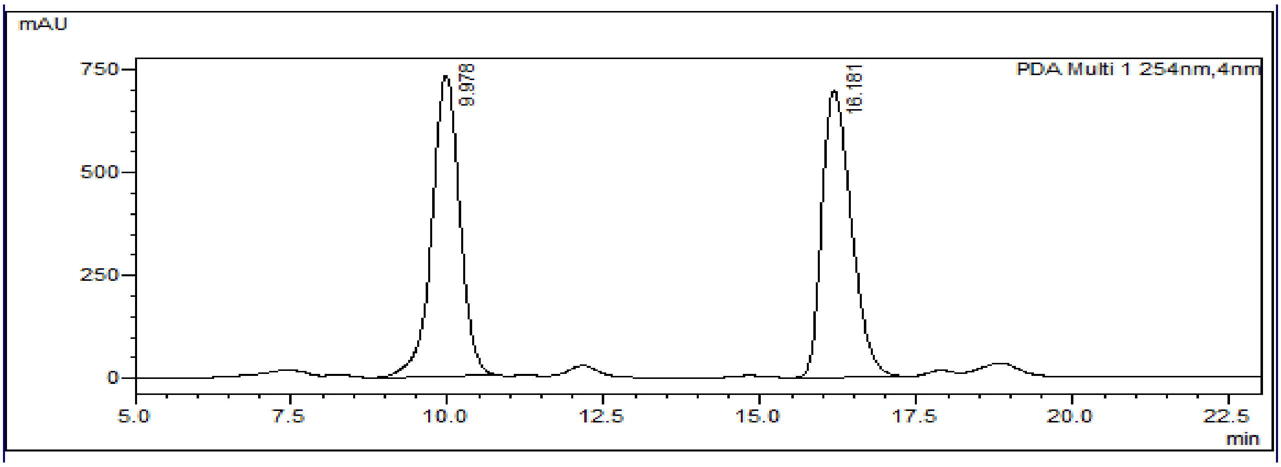

<Peak Table>

PDA Ch1 254nm

Peak#	Ret. Time	Area	Height	Area%	Height%
1	9.978	22609481	728951	49.809	51.222
2	16.181	22783068	694157	50.191	48.778
Total		45392548	1423108	100.000	100.000

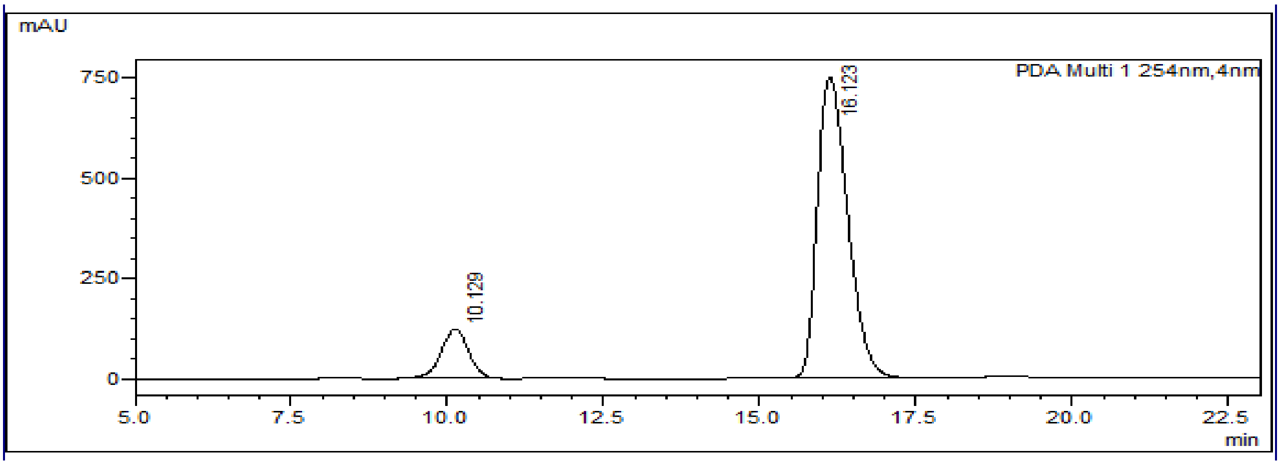

<Peak Table>

PDA Ch1 254nm

Peak#	Ret. Time	Area	Height	Area%	Height%
1	10.129	3507542	119859	12.175	13.818
2	16.123	25300671	747543	87.825	86.182
Total		28808214	867402	100.000	100.000

(3S,4R,4aS,8aR)-4-(4-Bromobenzoyl)-8a-methyl-3-(4,4,5,5-tetramethyl-1,3,2-dioxaborolan-2-yl)-3,4,4a,8a-tetrahydro-2*H*-chromen-6(5*H*)-one (2v):

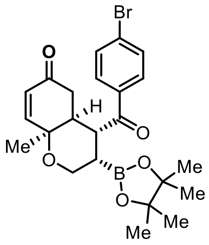

Prepared according to the general procedure as described above in 73% yield (104 mg) with 7:1*dr*. It was purified by flash chromatography (20% EtOAc/hexanes; R_f = 0.3) to afford a brown semi solid; ^{1}H NMR (500 MHz, $CDCl_3$) δ 7.62 (d, *J* = 8.6 Hz, 2H), 7.56 (d, *J* = 8.6 Hz, 2H), 6.69 (dd, *J* = 10.3, 1.5 Hz, 1H), 6.05 (d, *J* = 10.3 Hz, 1H), 4.00 (dd, *J* = 11.6, 3.6 Hz, 1H), 3.83 (dd, *J* = 11.6, 3.1 Hz, 1H), 3.46 (dd, *J* = 9.6, 4.9 Hz, 1H), 2.89 (dt, *J* = 9.0, 4.3 Hz, 1H), 2.66 (dd, *J* = 17.4, 5.0 Hz, 1H), 2.57 (dd, *J* = 16.9, 3.3 Hz, 1H), 1.49 (dd, *J* = 7.7, 3.5 Hz, 1H), 1.46 (s, 3H), 1.20 (s, 6H), 1.19 (s, 6H); ^{13}C NMR (101 MHz, $CDCl_3$) δ 200.3, 198.4, 155.4, 135.6, 132.2, 130.1, 129.9, 128.2, 83.7, 73.4, 64.5, 45.8, 40.3, 39.6, 25.9, 24.9, 24.8; HRMS (ESI) calcd for $C_{23}H_{29}O_5BBr$ $[M+H]^+$: 475.1286; found: 475.1275; $[\alpha]^{20}_D$ = -73.50° (*c* 1.56, $CHCl_3$); 95:05 *er*; Chiral HPLC analysis of the product: Daicel Chiralpak IA 250X4.6 mm 5µ column; hexane/2-propanol = 97/03, detected at 254 nm, Flow rate = 1 mL/min, Retention times: 19.333 min (major), 11.509 min (minor).

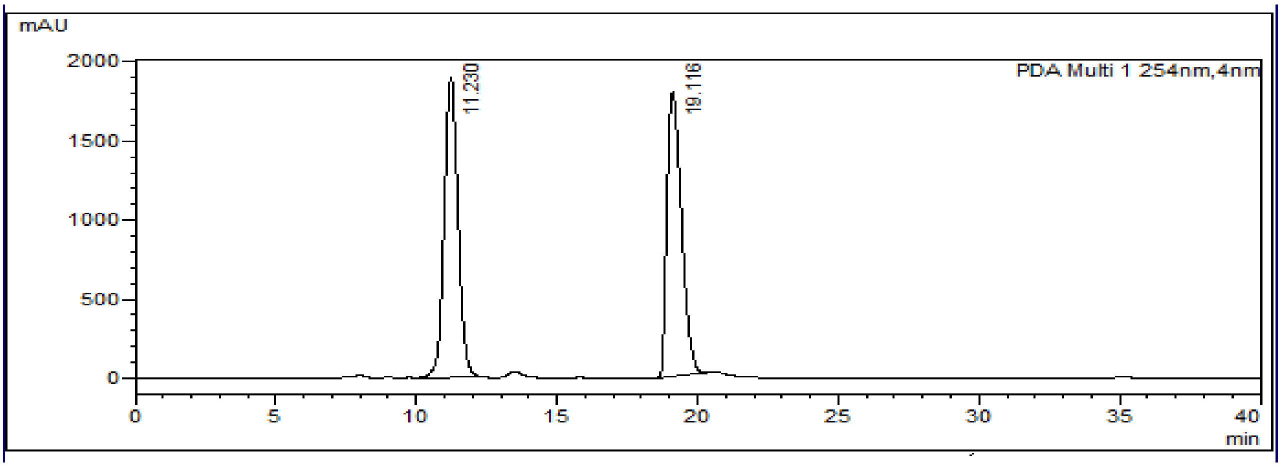

<Peak Table>

PDA Ch1 254nm

Peak#	Ret. Time	Area	Height	Area%	Height%
1	11.230	64704029	1897554	50.115	51.404
2	19.116	64406989	1793869	49.885	48.596
Total		129111017	3691423	100.000	100.000

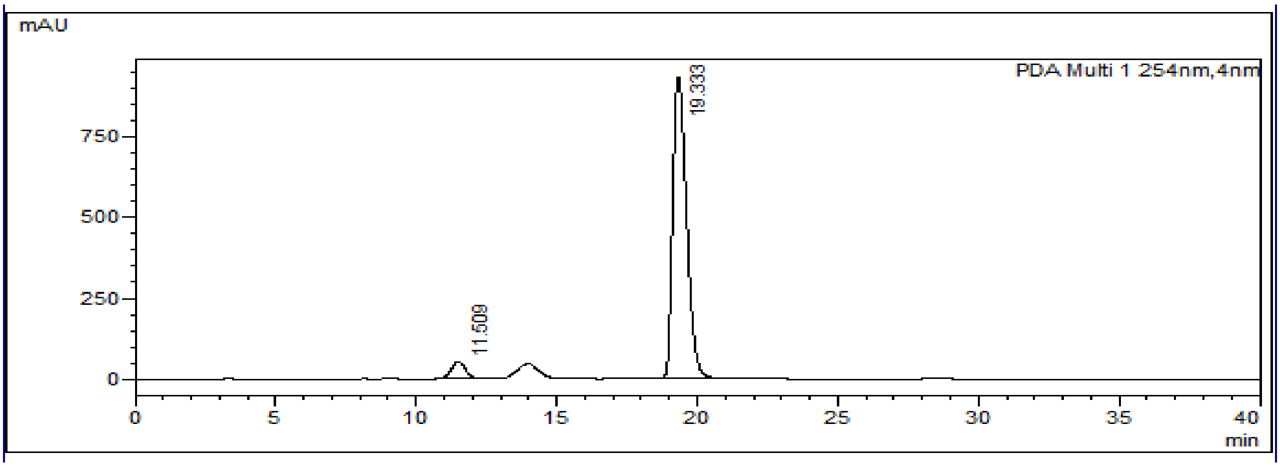

<Peak Table>

PDA Ch1 254nm

Peak#	Ret. Time	Area	Height	Area%	Height%
1	11.509	1815080	53048	5.414	5.392
2	19.333	31709972	930849	94.586	94.608
Total		33525053	983897	100.000	100.000

(3S,4R,4aS,8aR)-4-([1,1'-Biphenyl]-4-carbonyl)-8a-methyl-3-(4,4,5,5-tetramethyl-1,3,2-dioxaborolan-2-yl)-3,4,4a,8a-tetrahydro-2*H*-chromen-6(5*H*)-one (2w):

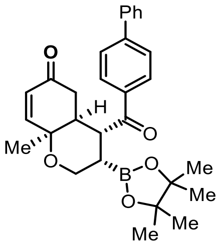

Prepared according to the general procedure as described above in 93% yield (131 mg) with >20:1*dr*. It was purified by flash chromatography (20% EtOAc/hexanes; $R_f = 0.3$) to afford a brown semi solid; ^{1}H NMR (400 MHz, $CDCl_3$) 7.84 (d, *J* = 8.5 Hz, 2H), 7.64 (d, *J* = 8.5 Hz, 2H), 7.62 – 7.56 (m, 2H), 7.50 – 7.44 (m, 2H), 7.43 – 7.36 (m, 1H), 6.70 (dd, *J* = 10.3, 1.5 Hz, 1H), 6.07 (d, *J* = 10.3 Hz, 1H), 4.04 (dd, *J* = 11.6, 4.2 Hz, 1H), 3.88 (dd, *J* = 11.6, 3.3 Hz, 1H), 3.58 (dd, *J* = 9.2, 4.8 Hz, 1H), 2.95 (dt, *J* = 8.8, 4.4 Hz, 1H), 2.66 (t, *J* = 4.4 Hz, 2H), 1.58 (dd, *J* = 8.1, 4.1 Hz, 1H), 1.47 (s, 3H), 1.24 (s, 6H), 1.21 (s, 6H); ^{13}C NMR (126 MHz, $CDCl_3$) δ 201.1, 198.6, 155.4, 145.8, 140.0, 135.4, 130.0, 129.1, 128.9, 128.3, 127.5, 127.4, 83.6, 73.2, 64.4, 46.0, 40.3, 39.6, 25.8, 24.9, 24.8; HRMS (ESI) calcd for $C_{29}H_{34}BO_5$ $[M+H]^+$: 473.2499; found: 473.2516; $[\alpha]^{20}_D$ = -104.02° (*c* 2.33, $CHCl_3$); 94:06 *er*; Chiral HPLC analysis of the product: Daicel Chiralpak IA 250X4.6 mm 5µ column; hexane/2-propanol = 85/15, detected at 254 nm, Flow rate = 1 mL/min, Retention times: 9.667 min (major), 7.002 min (minor).

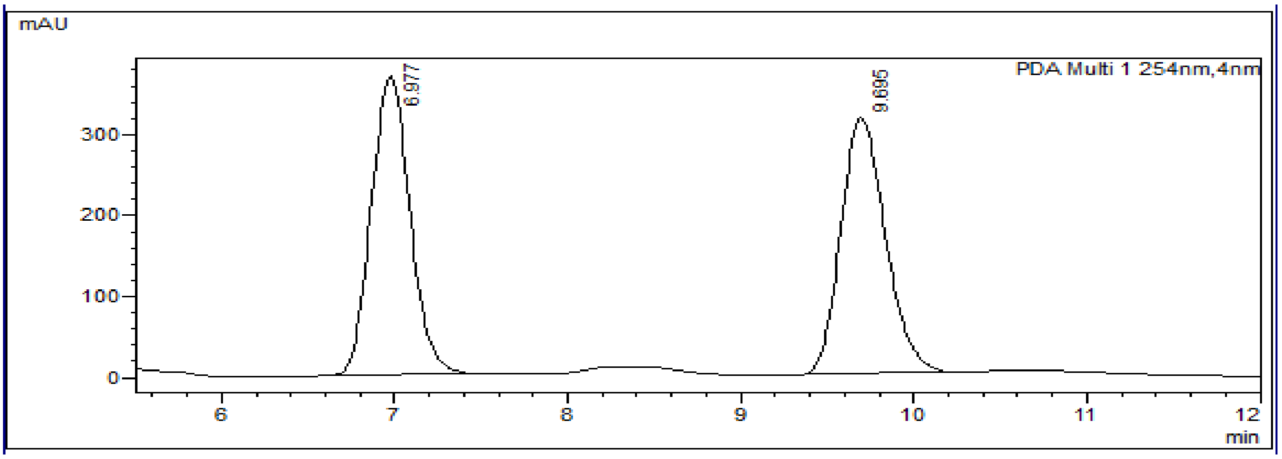

<Peak Table>

PDA Ch1 254nm

Peak#	Ret. Time	Area	Height	Area%	Height%
1	6.977	5701925	368395	50.256	53.841
2	9.695	5643758	315828	49.744	46.159
Total		11345683	684223	100.000	100.000

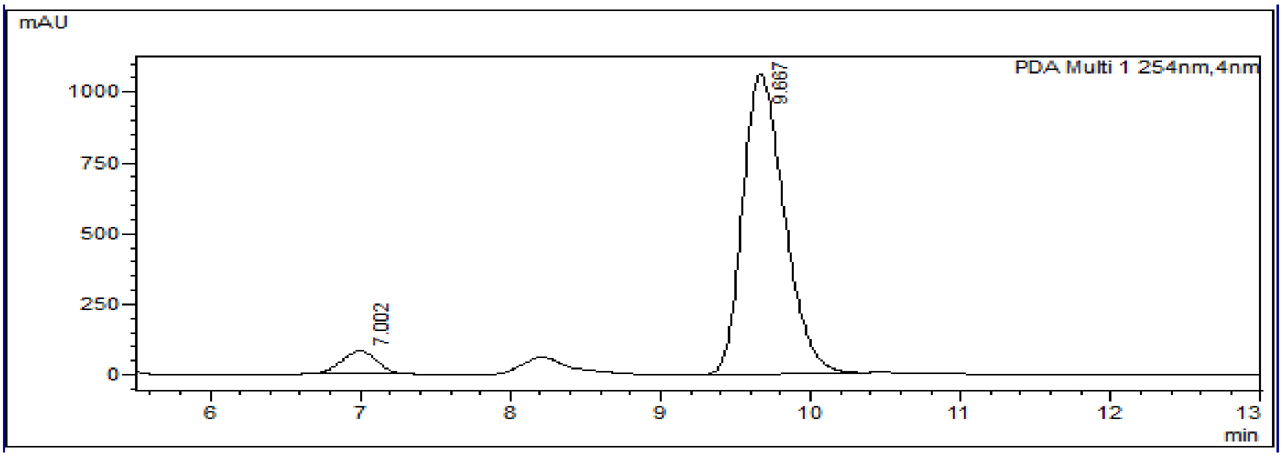

<Peak Table>

PDA Ch1 254nm

Peak#	Ret. Time	Area	Height	Area%	Height%
1	7.002	1261350	79220	5.831	6.955
2	9.667	20369291	1059788	94.169	93.045
Total		21630642	1139008	100.000	100.000

4-((3S,4R,4aS,8aR)-8a-Methyl-6-oxo-3-(4,4,5,5-tetramethyl-1,3,2-dioxaborolan-2-yl)-3,4,4a,5,6,8a-hexahydro-2*H*-chromene-4-carbonyl)benzonitrile (2x):

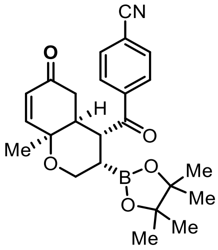

Prepared according to the general procedure as described above in 83% yield (105 mg) with >20:1*dr*. It was purified by flash chromatography (30% EtOAc/hexanes; $R_f = 0.3$) to afford a brown semi solid; ^{1}H NMR (500 MHz, $CDCl_3$) δ 7.83 (d, *J* = 8.6 Hz, 2H), 7.73 (d, *J* = 8.5 Hz, 2H), 6.71 (dd, *J* = 10.3, 1.8 Hz, 1H), 6.07 (dd, *J* = 10.3, 0.8 Hz, 1H), 4.00 (dd, *J* = 11.5, 3.1 Hz, 1H), 3.83 (dd, *J* = 11.6, 3.0 Hz, 1H), 3.48 (dd, *J* = 10.1, 4.9 Hz, 1H), 2.90 (dt, *J* = 9.1, 4.1 Hz, 1H), 2.71 (dd, *J* = 17.4, 5.1 Hz, 1H), 2.56 (dd, *J* = 17.5, 3.2 Hz, 1H), 1.46 (dd, *J* = 5.1, 2.6 Hz, 1H), 1.23 (s, 3H), 1.20 (s, 6H), 1.19 (s, 6H); ^{13}C NMR (101 MHz, $CDCl_3$) δ 199.9, 198.3, 155.5, 140.2, 132.7, 130.3, 128.7, 118.0, 116.3, 83.9, 73.6, 64.6, 46.1, 40.2, 39.6, 26.1, 24.9, 24.7; HRMS (ESI) calcd for $C_{24}H_{29}O_5NB$ $[M+H]^+$: 422.2133;

found: 422.2125; $[\alpha]^{20}_{D}$ =-46.00° (*c* 2.16, $CHCl_3$); 82:18 *er*; Chiral HPLC analysis of the product: Daicel Chiralpak IA 250X4.6 mm 5µ column; hexane/2-propanol = 93/07, detected at 254 nm, Flow rate = 1 mL/min, Retention times: 21.940 min (major), 12.718 min (minor).

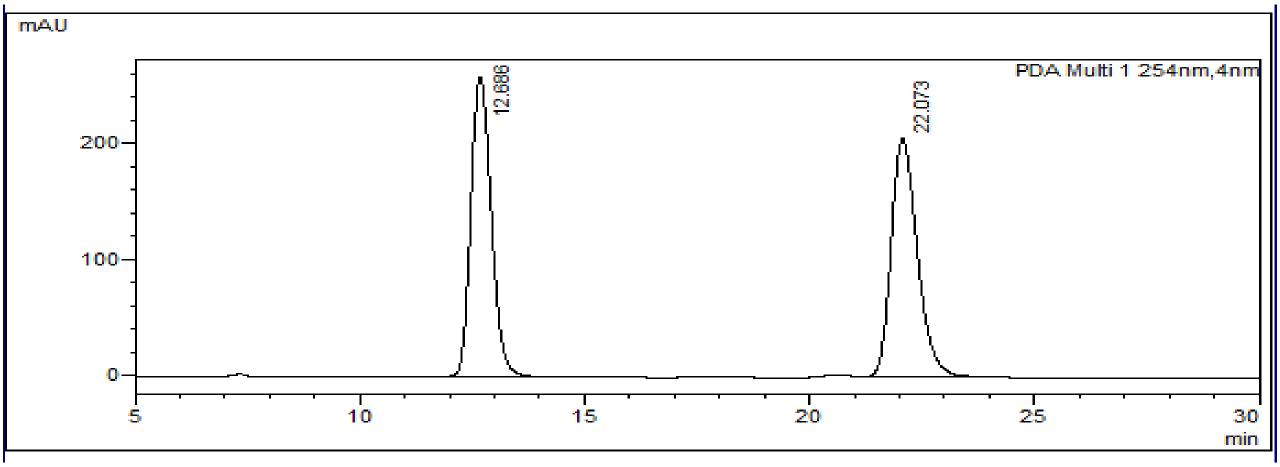

<Peak Table>

PDA Ch1 254nm

Peak#	Ret. Time	Area	Height	Area%	Height%
1	12.686	8247015	258433	49.786	55.761
2	22.073	8317880	205029	50.214	44.239
Total		16564895	463462	100.000	100.000

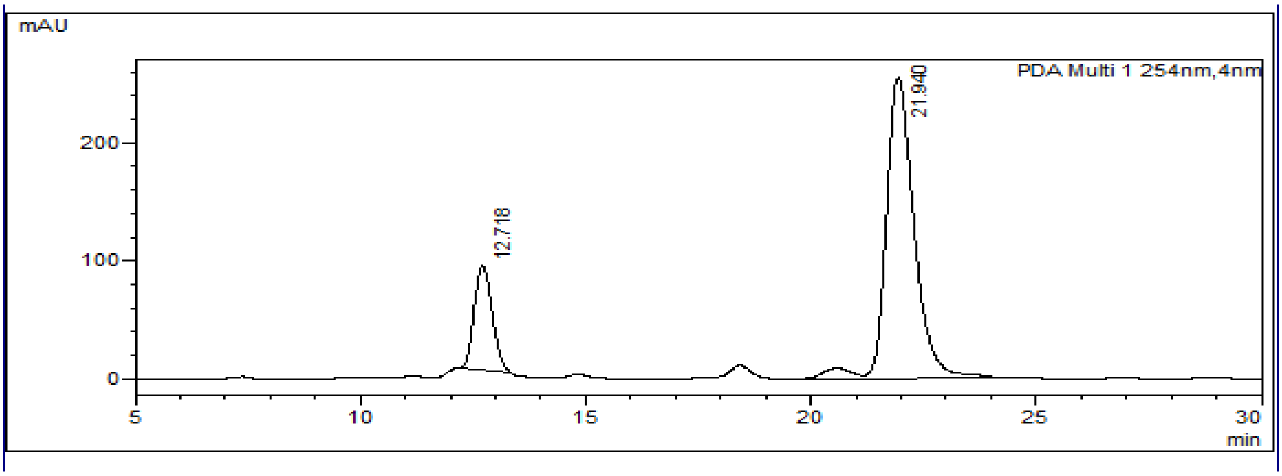

<Peak Table>

PDA Ch1 254nm

Peak#	Ret. Time	Area	Height	Area%	Height%
1	12.718	2494295	88658	18.159	25.792
2	21.940	11241636	255082	81.841	74.208
Total		13735931	343740	100.000	100.000

4-Benzoyl-5,7,8a-trimethyl-3-(4,4,5,5-tetramethyl-1,3,2-dioxaborolan-2-yl)-3,4,4a,8a-tetrahydro-2*H*-chromen-6(5*H*)-one (2zb):

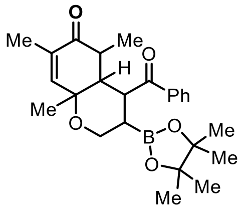

Prepared according to the general procedure as described above in 24% yield (31mg) with 1.5:1.0:1.4 *dr*. It was purified by flash chromatography (20% EtOAc/hexanes; $R_f = 0.3$) to afford a colourless semi solid; HRMS (ESI) calcd for $C_{25}H_{34}BO_5$ $[M+H]^+$: 425.2499; found: 425.2504; $[\alpha]^{20}_D$ = -132.11° (*c* 2.23, $CHCl_3$).

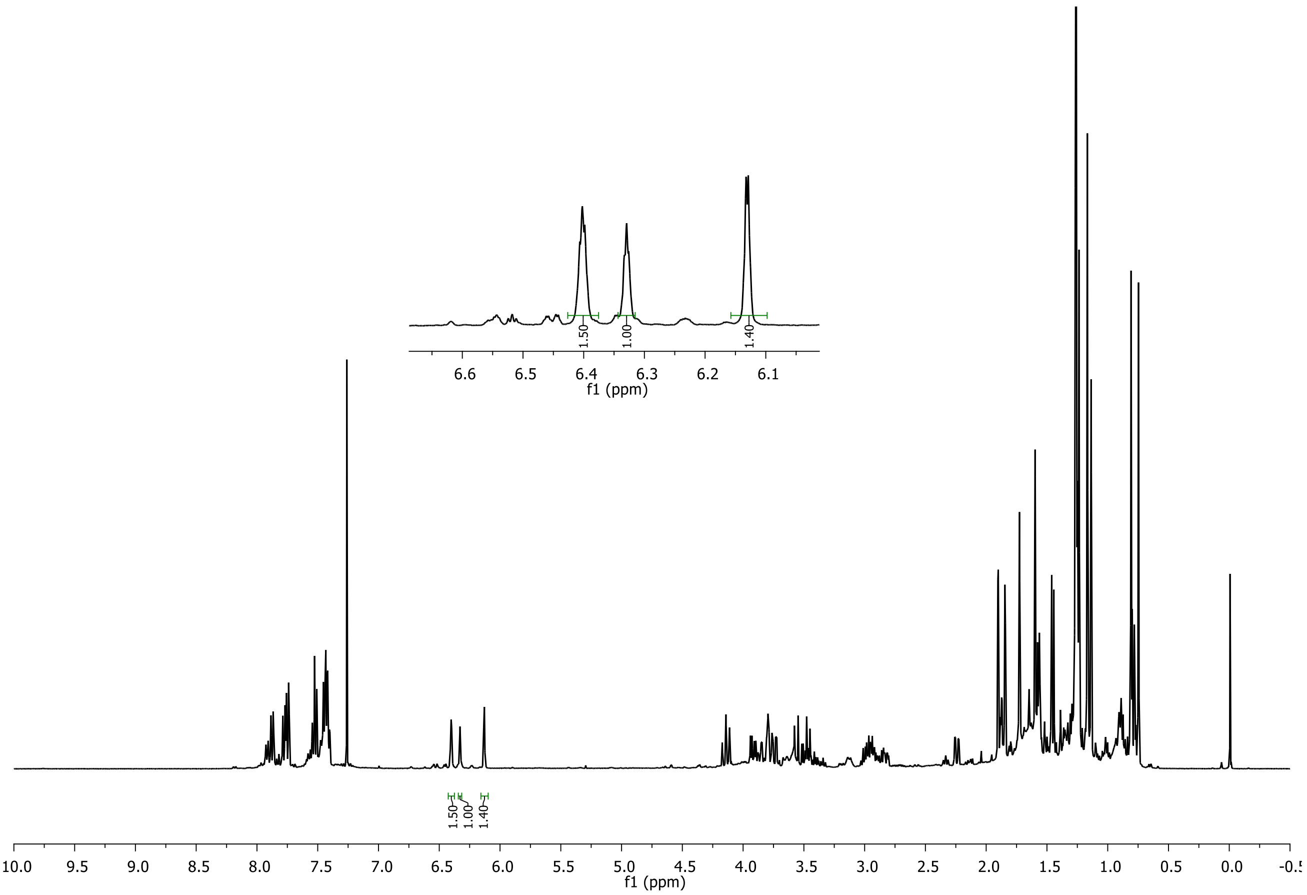

(*S*)-2,4,6-Trimethyl-4-(4-oxo-4-phenyl-2-(4,4,5,5-tetramethyl-1,3,2-dioxaborolan-2-yl)butoxy)cyclohexa-2,5-dien-1-one (2zb′):

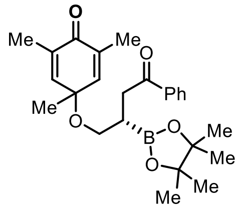

Prepared according to the general procedure as described above in 45% yield (57 mg). It was purified by flash chromatography (20% EtOAc/hexanes; R_f = 0.5) to afford an orange liquid; ^{1}H NMR (400 MHz, $CDCl_3$) δ 7.98 (dd, *J* = 8.3, 1.3 Hz, 2H), 7.59 – 7.53 (m, 1H), 7.50 – 7.43 (m, 2H), 6.50 (dq, *J* = 2.9, 1.4 Hz, 1H), 6.39 (dq, *J* = 2.8, 1.3 Hz, 1H), 3.45 (dd, *J* = 8.9, 4.9 Hz, 1H), 3.38 – 3.31 (m, 1H), 3.30 – 3.14 (m, 2H), 1.93 – 1.88 (m, 1H), 1.86 (d, *J* = 1.4 Hz, 3H), 1.80 (d, *J* = 1.4 Hz, 3H), 1.30 (s, 3H), 1.25 (s, 6H), 1.25 (s, 6H); ^{13}C NMR (101 MHz, $CDCl_3$) δ 200.1, 186.9, 147.7, 147.6, 137.2, 136.1, 136.0, 133.0, 128.6, 128.2, 83.4, 72.1, 65.5, 37.4, 26.9, 24.9, 24.8, 16.1, 16.0; HRMS (ESI) calcd for $C_{25}H_{33}BO_5Na$ $[M+Na]^+$: 447.2319; found: 447.2324; $[\alpha]^{20}_D$ = -110.12° (*c* 1.33, $CHCl_3$); 95:05 *er*; Chiral HPLC analysis of the product: Daicel Chiralpak IA 250X4.6 mm 5μ column; hexane/2-propanol = 97.5/1.5, detected at 240 nm, Flow rate = 0.5 mL/min, Retention times: 13.663 min (major), 16.569 min (minor).

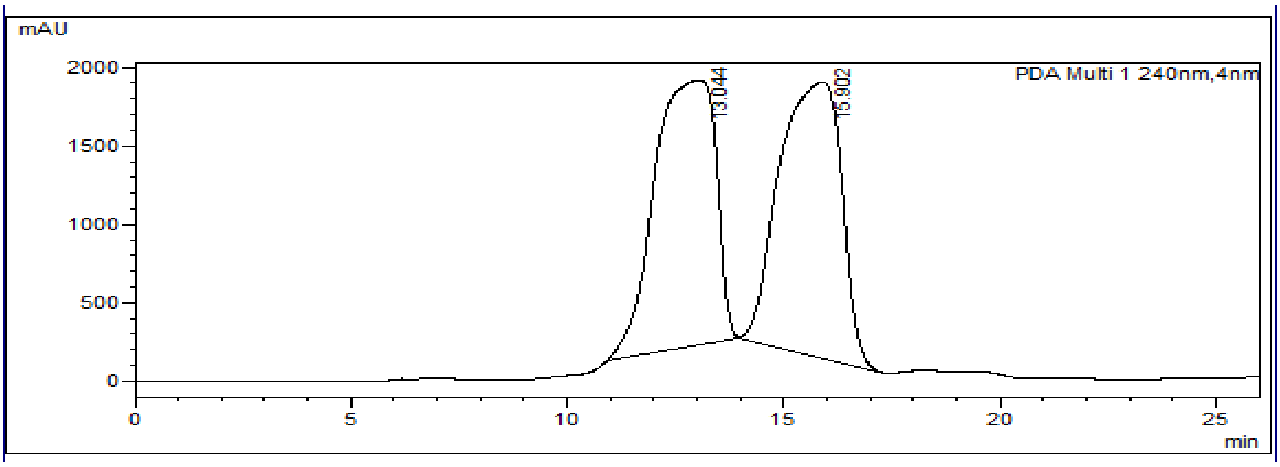

<Peak Table>

PDA Ch1 240nm

Peak#	Ret. Time	Area	Height	Area%	Height%
1	13.044	167893591	1683874	49.089	48.924
2	15.902	174127310	1757923	50.911	51.076
Total		342020901	3441797	100.000	100.000

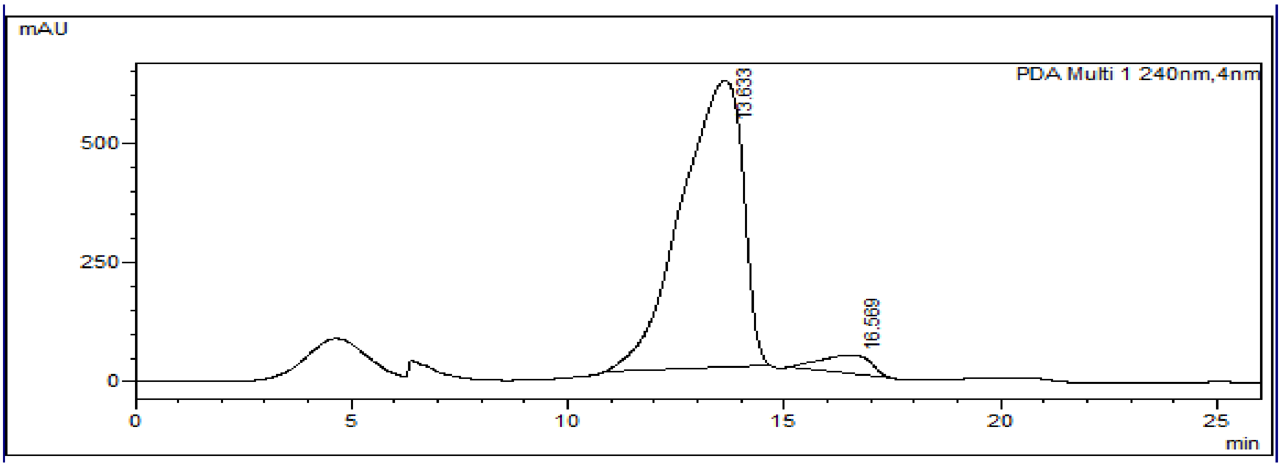

<Peak Table>

PDA Ch1 240nm

Peak#	Ret. Time	Area	Height	Area%	Height%
1	13.633	59811505	600260	94.892	93.763
2	16.569	3219767	39929	5.108	6.237
Total		63031271	640189	100.000	100.000

(3S,4R,4aS,8aR)-4-Benzoyl-3-(5,5-dimethyl-1,3,2-dioxaborinan-2-yl)-8a-methyl-3,4,4a,8a-tetrahydro-2*H*-chromen-6(5H)-one (2zc):

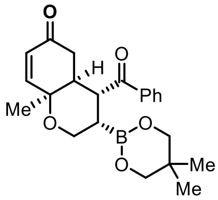

Prepared according to the general procedure as described above in 77% yield (88 mg) with 20:1*dr*. It was purified by flash chromatography (20% EtOAc/hexanes; R_f = 0.3) to afford a brown semi solid; ^{1}H NMR (500 MHz, $CDCl_3$) δ 7.74 (dd, *J* = 5.1, 3.4 Hz, 2H), 7.57 – 7.47 (m, 1H), 7.41 (t, *J* = 7.7 Hz, 2H), 6.66 (dd, *J* = 10.3, 1.3 Hz, 1H), 6.02 (d, *J* = 10.3 Hz, 1H), 4.03 (dd, *J* = 11.6, 4.5 Hz, 1H), 3.82 (dd, *J* = 11.6, 3.5 Hz, 1H), 3.56 – 3.49 (m, 4H), 3.48 (dd, *J* = 8.8, 5.1 Hz, 1H), 2.90 – 2.79 (m, 1H), 2.70 (dd, *J* = 17.3, 5.0 Hz, 1H), 2.63 (dd, *J* = 17.3, 5.1 Hz, 1H), 1.49 (dd, *J* = 8.1, 4.1 Hz, 1H), 1.42 (s, 3H), 0.89 (s, 6H); ^{13}C NMR (101 MHz, $CDCl_3$) δ 202.3, 198.8, 155.3, 137.0, 132.8, 129.8, 128.7, 128.2, 73.0, 72.0, 64.5, 46.0, 40.3, 39.5, 31.7, 25.7, 21.9; HRMS (ESI) calcd for $C_{22}H_{28}BO_5$ $[M+H]^+$: 383.2030; found: 383.2030; $[\alpha]^{20}_D$ = -57.33° (*c*1.22, $CHCl_3$); 86:14 *er*; Chiral HPLC analysis of the product: Daicel Chiralpak IC 250X4.6 mm 5µ column; hexane/2-propanol = 93/07, detected at 254 nm, Flow rate = 1 mL/min, Retention times: 22.603 min (major), 17.413 min (minor).

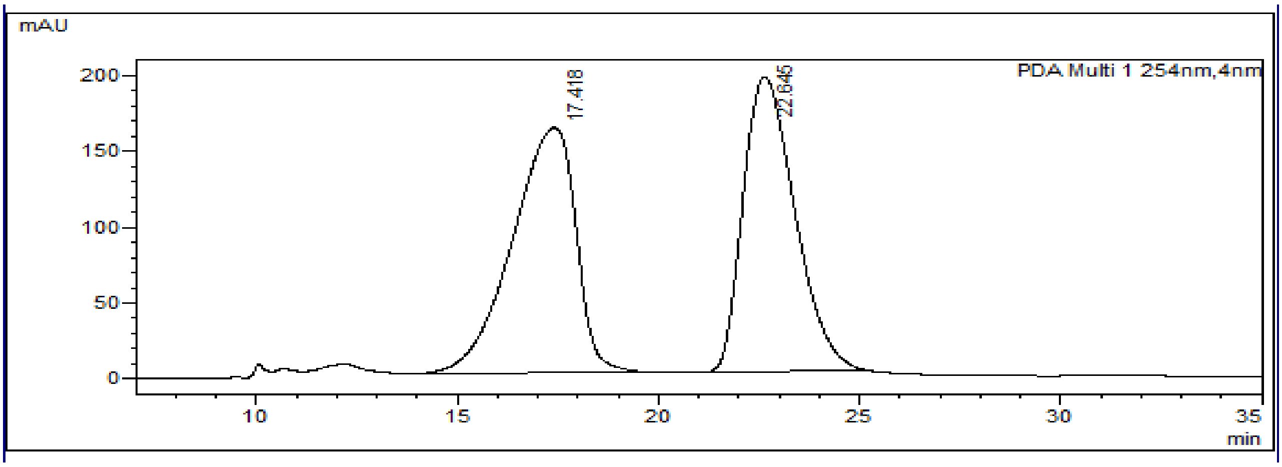

<Peak Table>

PDA Ch1 254nm

Peak#	Ret. Time	Area	Height	Area%	Height%
1	17.418	17700567	161141	50.328	45.358
2	22.645	17469677	194120	49.672	54.642
Total		35170244	355261	100.000	100.000

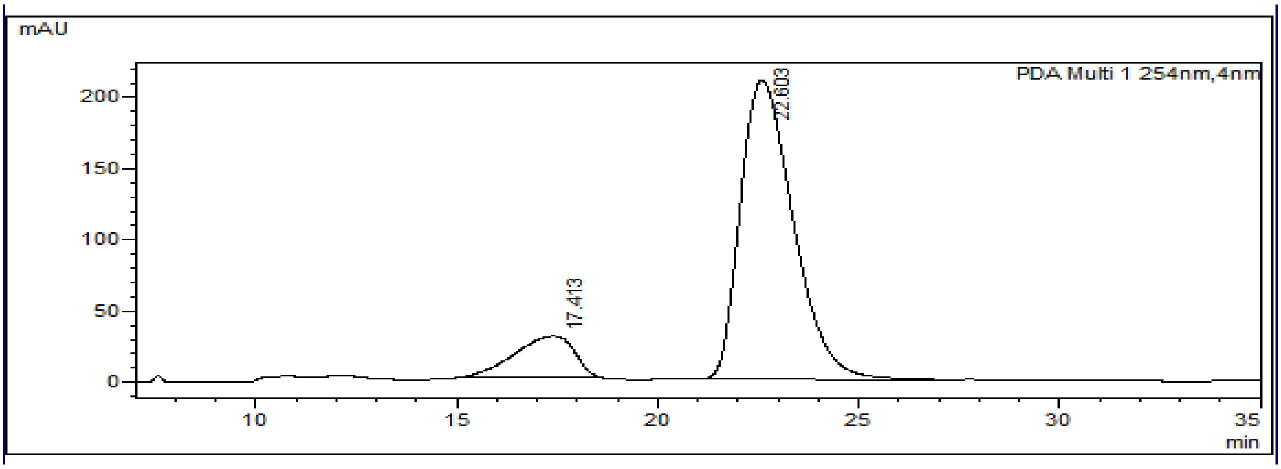

<Peak Table>

PDA Ch1 254nm

Peak#	Ret. Time	Area	Height	Area%	Height%
1	17.413	3018549	29157	13.507	12.196
2	22.603	19329882	209905	86.493	87.804
Total		22348432	239062	100.000	100.000

(3*S*,4*R*,4a*S*,8a*R*)-4-Benzoyl-8a-methyl-3-(4,4,5,5-tetramethyl-1,3,2-dioxaborolan-2-yl)-1-tosyl-1,3,4,4a,5,8a-hexahydroquinolin-6(2*H*)-one (2zd):

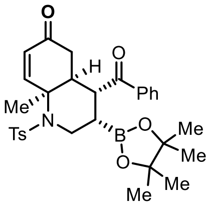

Prepared according to the general procedure as described above in 32% yield (26 mg) with 10:1 *dr*; It was purified by flash chromatography (30% EtOAc/hexanes; R_f = 0.4) to afford a colourless semi solid; ^{1}H NMR (400 MHz, CDCl3) δ 7.72 – 7.63 (m, 4H), 7.56 – 7.51 (m, 1H), 7.46 – 7.41 (m, 2H), 7.27 – 7.22 (m, 2H), 6.84 (d, J = 10.1 Hz, 1H), 5.78 (dd, J = 10.1, 0.7 Hz, 1H), 4.23 (dd, J = 14.0, 4.7 Hz, 1H), 3.57 (dd, J = 16.6, 10.2 Hz, 1H), 3.41 (dd, J = 5.0, 1.2 Hz, 1H), 2.93 (dd, J = 16.5, 13.3 Hz, 1H), 2.49 (ddd, J = 13.3, 3.0, 1.6 Hz, 1H), 2.40 (s, 3H), 2.36 (dd, J = 9.8, 2.8 Hz, 1H), 1.71 – 1.58 (m, 1H), 1.27 (s, 3H), 1.24 (s, 12H); ^{13}C NMR (101 MHz, $CDCl_3$) δ 204.07, 198.76, 153.53, 143.57, 139.11, 135.77, 133.35, 129.83, 129.11, 128.42, 127.44, 127.15, 83.79, 57.66, 48.55, 44.62, 42.21, 40.89, 25.00, 24.69, 22.83, 21.67; HRMS (ESI) calcd for $C_{30}H_{37}BNO_6S$ $[M+H]^+$: 550.2435; found: 550.2432; $[\alpha]^{20}_D$ = -186.18° (*c* 0.95, $CHCl_3$); 99:01 *er*; Chiral HPLC analysis of the product: Daicel Chiralpak Eurocel-01 250X4.6 mm 5µ column; hexane/2-propanol = 93/07, detected at 230 nm, Flow rate = 1 mL/min, Retention times: 40.920 min (major), 29.151 min (minor).

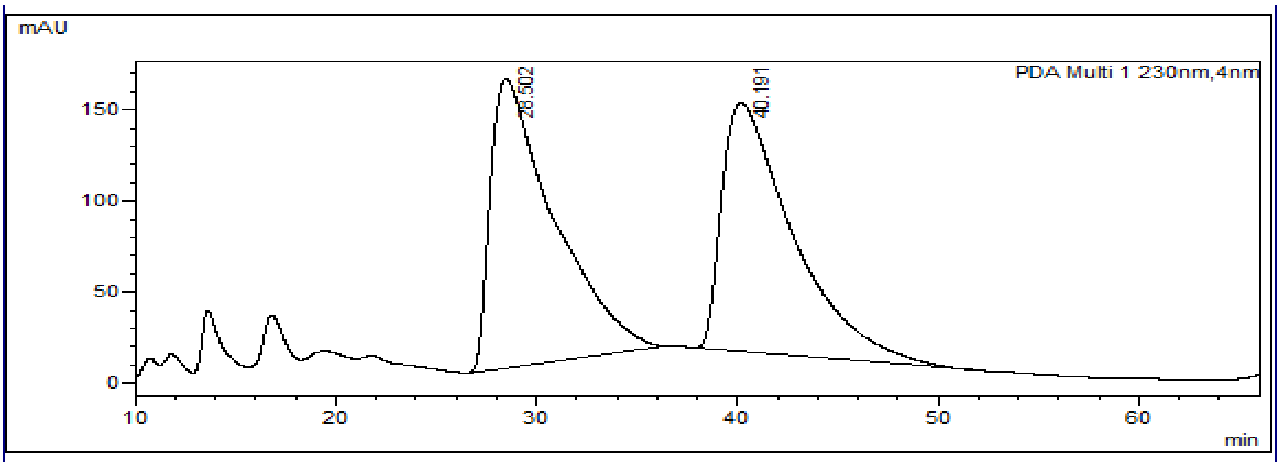

<Peak Table>

PDA Ch1 230nm

Peak#	Ret. Time	Area	Height	Area%	Height%
1	28.502	34801359	158201	50.680	53.665
2	40.191	33866902	136595	49.320	46.335
Total		68668261	294796	100.000	100.000

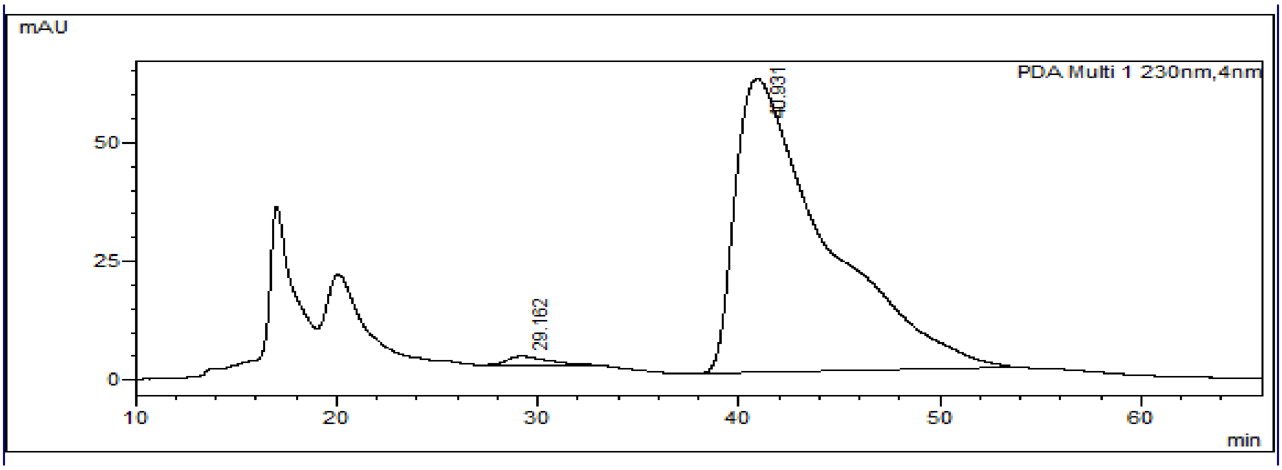

<Peak Table>

PDA Ch1 220nm

Peak#	Ret. Time	Area	Height	Area%	Height%
1	29.151	246803	1693	1.421	2.977
2	40.920	17124730	55168	98.579	97.023
Total		17371533	56861	100.000	100.000

(4aR,7R,8S,8aS)-8-benzoyl-4a-methoxy-7-(4,4,5,5-tetramethyl-1,3,2-dioxaborolan-2-yl)-4a,5,6,7,8,8a-hexahydronaphthalen-2(1*H*)-one (5):

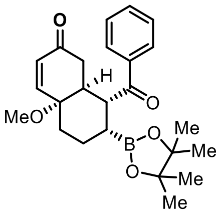

Prepared according to the general procedure as described above in 47% yield (58 mg) with ~3:2 *dr*. It was purified by flash chromatography (30% EtOAc/hexanes; R_f = 0.3) to afford an orange semi solid; ^{1}H NMR (500 MHz, $CDCl_3$) δ 7.88 (dt, *J* = 8.4, 1.6 Hz, 0.8H), 7.71 (dt, *J* = 8.4, 1.6 Hz, 1.2H), 7.57 – 7.53 (m, 0.4H), 7.52 – 7.48 (m, 0.6H), 7.48 – 7.40 (m, 2H), 6.86 (d, *J* = 10.3 Hz, 0.4H), 6.78 (d, *J* = 10.3 Hz, 0.6H), 5.94 (dd, *J* = 10.3, 1.0 Hz, 0.4H), 5.91 (d, *J* = 10.3 Hz, 0.6H), 3.96 (dd, *J* = 11.9, 4.2 Hz, 0.4H), 3.62 (d, *J* = 26.8 Hz, 0.4H), 3.38 (s, 1.2H), 3.32 (d, *J* = 3.3 Hz, 0.6H), 2.98 (dd, *J* = 13.5, 5.0 Hz, 0.6H), 2.88 (s, 1.8H), 2.78 (dt, *J* = 17.5, 8.4 Hz, 1H), 2.69 (dd, *J* = 16.9, 5.0 Hz, 0.6H), 2.42 (dd, *J* = 17.3, 14.2 Hz, 0.4H), 2.06 – 1.88 (m, 2H), 1.80 – 1.70 (m, 1H), 1.70 – 1.63 (m, 1H), 1.54 – 1.45 (m, 1H), 1.27 (s, 3.6H), 1.21 (s, 2.4H), 1.17 (s, 3.6H), 1.11 (s, 2.4H); ^{13}C NMR (101 MHz, $CDCl_3$) δ 203.9, 202.3, 199.1, 198.5, 154.8, 153.7, 137.4, 136.7, 133.0, 132.1, 130.1, 128.9, 128.7, 128.1, 128.0, 83.4, 83.3, 76.0, 75.3, 50.7, 49.7, 48.5, 46.1, 41.6, 40.1, 37.4, 36.3, 31.6, 29.8, 25.1, 24.8, 24.7, 24.4, 21.3, 18.8; HRMS (ESI) calcd for $C_{24}H_{32}BO_5$ $[M+H]^+$: 411.2337; found: 411.2327; $[\alpha]^{20}_{D}$ = -13.23° (*c* 2.13, $CHCl_3$); Chiral HPLC analysis of the product: Daicel Chiralpak IA 250X4.6 mm 5μ column; hexane/2-propanol = 95/05, detected at 254 nm, Flow rate = 1 mL/min. For major isomer:

91:9 *er*, retention times : 6.549 (minor), 7.245 (major); For minor isomer: 85:15 *er*, Retention times : 17.441 (major), 38.420 (minor).

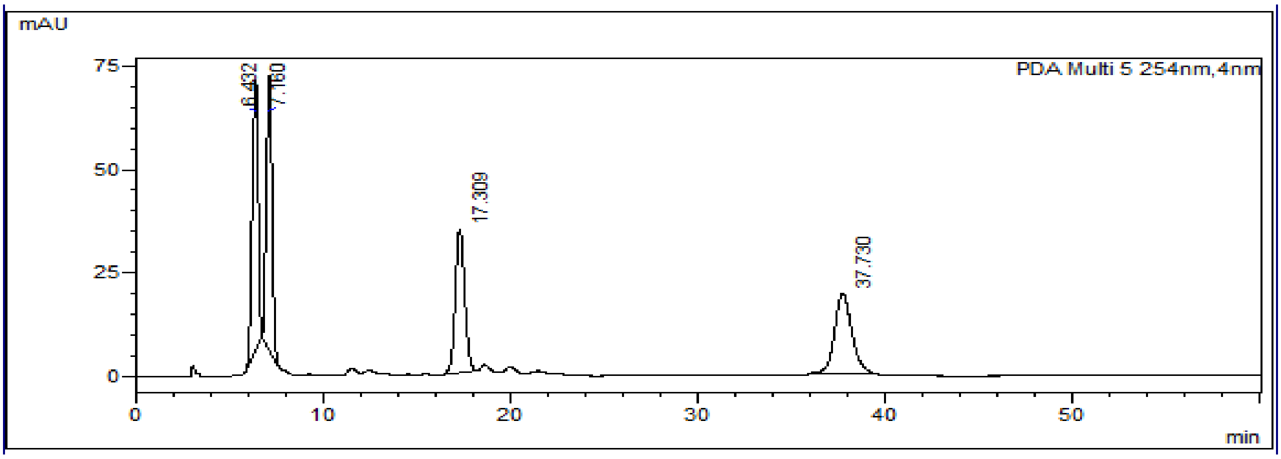

<Peak Table>

PDA Ch5 254nm

Peak#	Ret. Time	Area	Height	Area%	Height%
1	6.432	1424528	65119	27.029	35.146
2	7.160	1426685	66182	27.069	35.720
3	17.309	1204065	34728	22.846	18.743
4	37.730	1215179	19253	23.056	10.391
Total		5270456	185281	100.000	100.000

For major isomer:

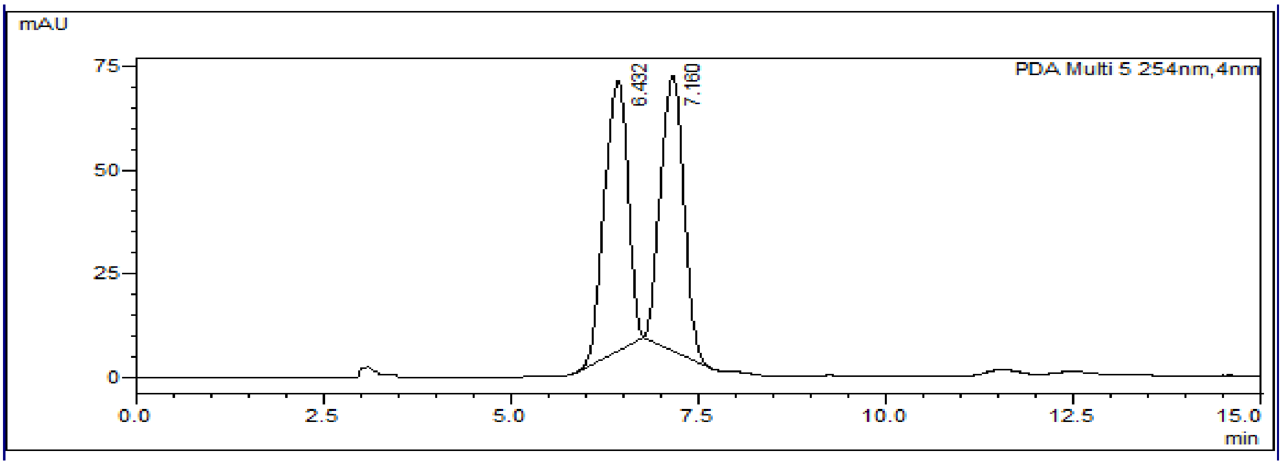

<Peak Table>

PDA Ch5 254nm

Peak#	Ret. Time	Area	Height	Area%	Height%
1	6.432	1424528	65119	49.962	49.595
2	7.160	1426685	66182	50.038	50.405
Total		2851213	131300	100.000	100.000

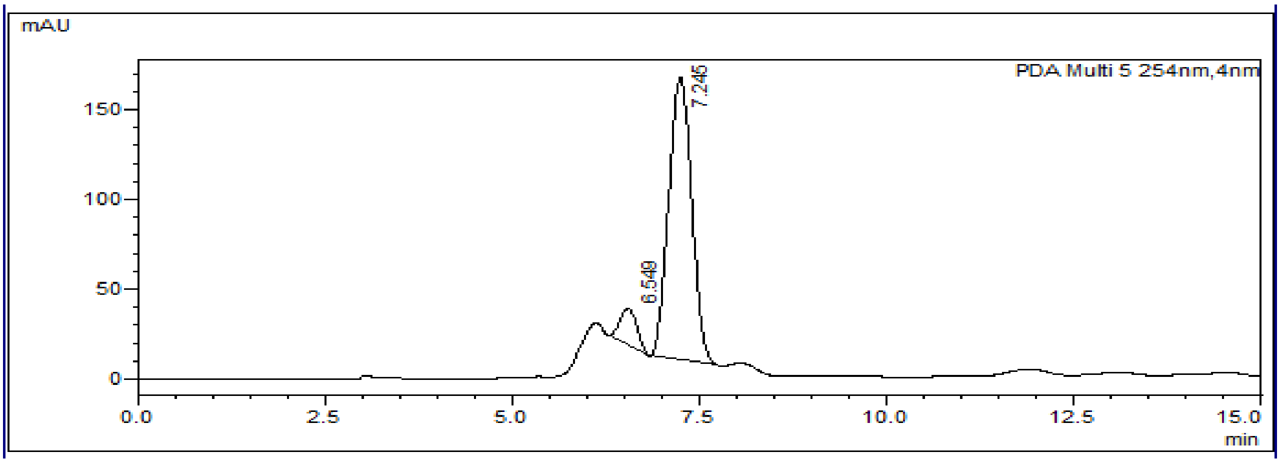

<Peak Table>

PDA Ch5 254nm

Peak#	Ret. Time	Area	Height	Area%	Height%
1	6.549	317294	20428	8.627	11.506
2	7.245	3360732	157108	91.373	88.494
Total		3678026	177536	100.000	100.000

For minor isomer:

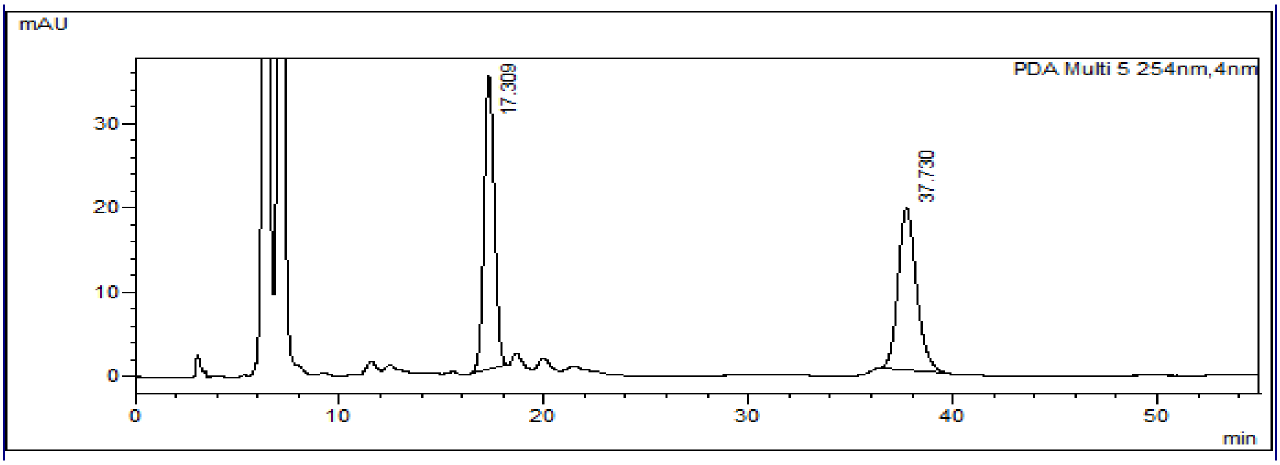

<Peak Table>

PDA Ch5 254nm

Peak#	Ret. Time	Area	Height	Area%	Height%
1	17.309	1205091	34723	50.047	64.442
2	37.730	1202833	19160	49.953	35.558
Total		2407924	53883	100.000	100.000

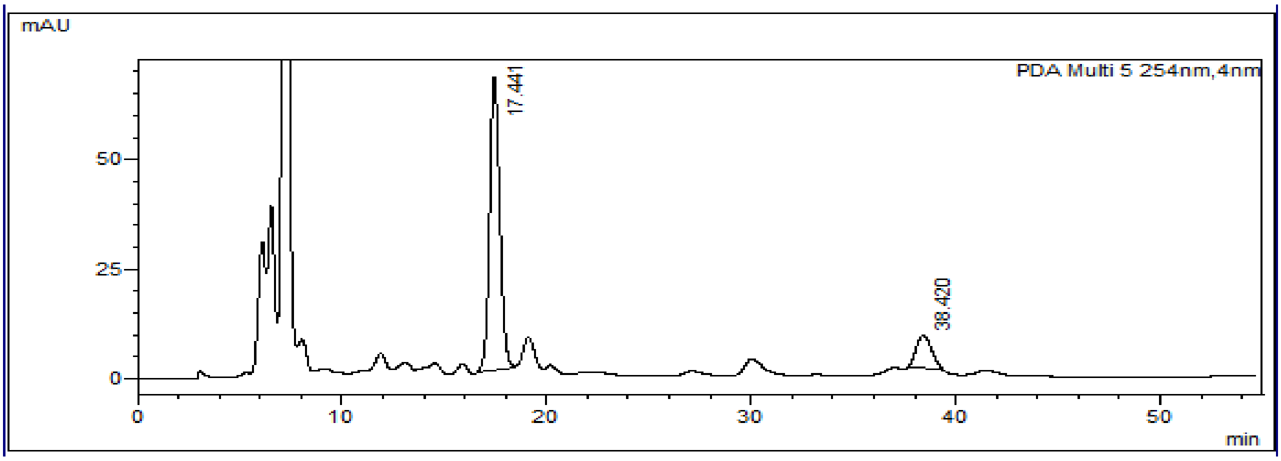

<Peak Table>

PDA Ch5 254nm

Peak#	Ret. Time	Area	Height	Area%	Height%
1	17.441	2293621	66660	85.287	89.783
2	38.420	395688	7585	14.713	10.217
Total		2689310	74245	100.000	100.000

(*R*)-5-(4-Hydroxyphenyl)-1-phenyl-3-(4,4,5,5-tetramethyl-1,3,2-dioxaborolan-2-yl)pentan-1-one (6):

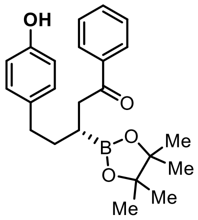

Prepared according to the general procedure as described above in 35% yield (40 mg). It was purified by flash chromatography (10% EtOAc/hexanes; R_f = 0.4) to afford a orange semi solid; ^{1}H NMR (500 MHz, $CDCl_3$) δ 7.94 (dd, *J* = 8.3, 1.2 Hz, 2H), 7.58 – 7.49 (m, 1H), 7.48 – 7.39 (m, 2H), 7.03 (d, *J* = 8.5 Hz, 2H), 6.74 (d, *J* = 8.5 Hz, 2H), 5.44 (br.s, 1H), 3.16 (dd, *J* = 7.2, 2.8 Hz, 2H), 2.61 (dd, *J* = 9.1, 7.1 Hz, 2H), 1.90 – 1.77 (m, 1H), 1.71 – 1.58 (m, 1H), 1.50 – 1.42 (m, 1H), 1.29 (s, 6H), 1.26 (s, 6H); ^{13}C NMR (126 MHz, $CDCl_3$) δ 200.7, 153.9, 137.1, 134.7, 133.0, 129.5, 128.6, 128.2, 115.3, 83.3, 41.2, 34.6, 33.0, 25.0, 24.9; HRMS (ESI) calcd for $C_{23}H_{30}BO_4$ $[M+H]^+$: 381.2232; found: 381.2236; $[\alpha]^{20}_D$ = -18.06° (*c* 0.82, $CHCl_3$); 80:20 *er*; Chiral HPLC analysis of the product: Daicel Chiralpak IA 250X4.6 mm 5μ column; hexane/2-propanol = 95/05, detected at 254 nm, Flow rate = 1 mL/min, Retention times: 18.513 min (major), 23.821 min (minor).

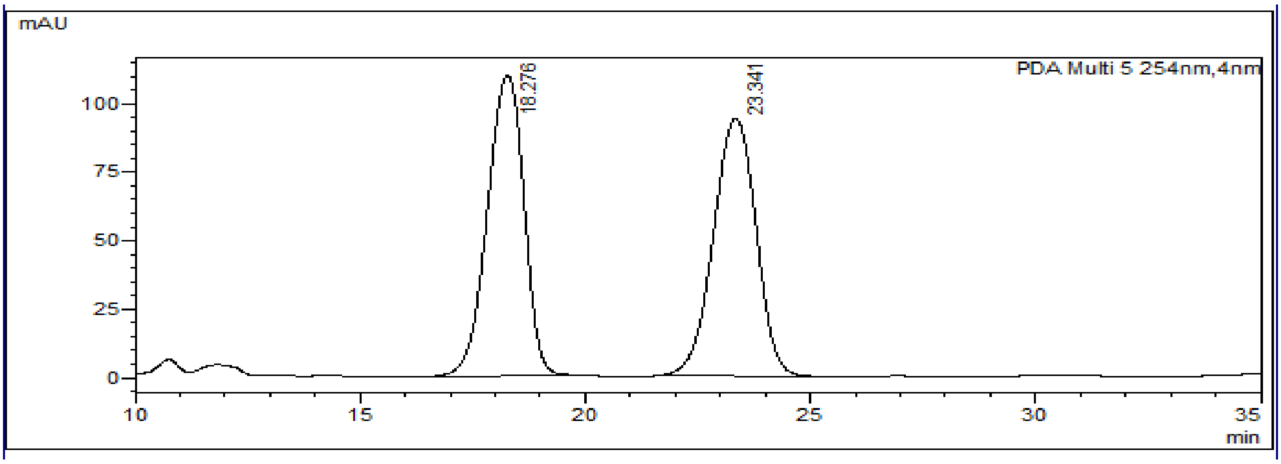

<Peak Table>

PDA Ch5 254nm

Peak#	Ret. Time	Area	Height	Area%	Height%
1	18.276	6227324	109718	50.042	53.847
2	23.341	6216970	94043	49.958	46.153
Total		12444294	203761	100.000	100.000

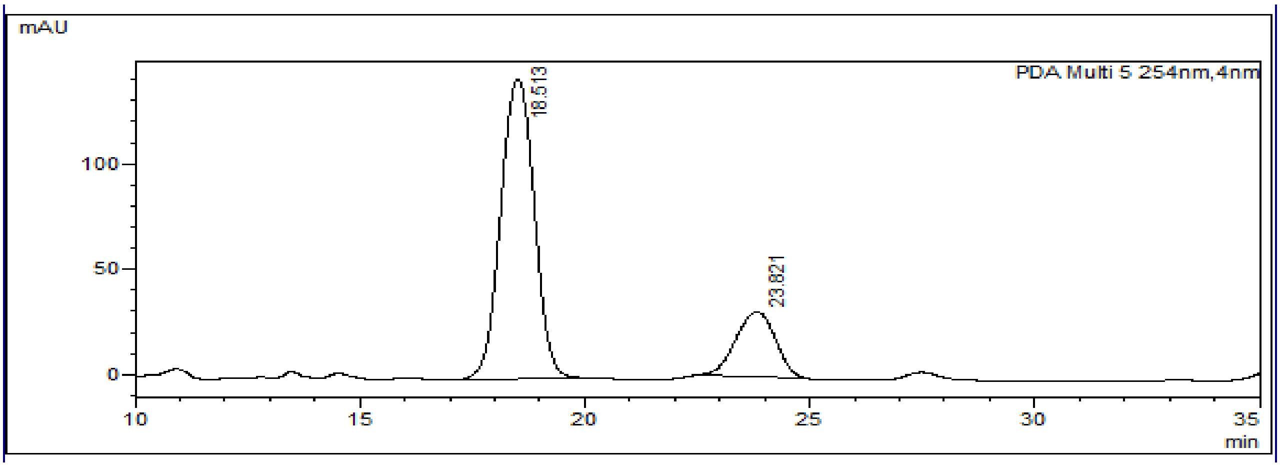

<Peak Table>

PDA Ch5 254nm

Peak#	Ret. Time	Area	Height	Area%	Height%
1	18.513	7395610	142230	79.689	82.271
2	23.821	1885000	30651	20.311	17.729
Total		9280610	172881	100.000	100.000

(3S,4R,4aS,8aR)-4-Benzoyl-8,8a-dimethyl-3-(4,4,5,5-tetramethyl-1,3,2-dioxaborolan-2-yl)-3,4,4a,8a-tetrahydro-2*H*-chromen-6(5*H*)-one (8):

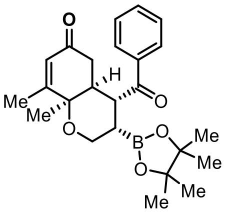

Prepared according to the general procedure as described above in 41% yield (50 mg) with 12:1*dr*. It was purified by flash chromatography (30% EtOAc/hexanes; R_f = 0.5) to afford a colourless solid; ^{1}H NMR (500 MHz, $CDCl_3$) δ 7.77 (dd, *J* = 8.3, 1.2 Hz, 2H), 7.54 – 7.49 (m, 1H), 7.43 – 7.37 (m, 2H), 6.01 (t, 1H), 3.94 (dd, *J* = 11.3, 2.0 Hz, 1H), 3.61 – 3.53 (m, 2H), 2.99 (ddd, *J* = 11.2, 5.3, 2.6 Hz, 1H), 2.68 (dd, *J* = 17.5, 5.3 Hz, 1H), 2.48 (ddd, *J* = 17.6, 2.5, 1.2 Hz, 1H), 1.99 (s, 3H), 1.53 (s, 3H), 1.48 (dd, *J* = 5.0, 2.8 Hz, 1H), 1.23 (s, 6H), 1.22 (s, 6H); ^{13}C NMR (126 MHz, $CDCl_3$) δ 201.1, 197.7, 164.3, 137.1, 133.0, 129.3, 128.8, 128.3, 83.6, 76.1, 65.0, 45.4, 40.4, 40.3, 25.8, 25.0, 24.8, 18.3; HRMS (ESI) calcd for $C_{24}H_{32}BO_5$ $[M+H]^+$:411.2337; found: 411.2328; $[\alpha]^{20}_D$ = -31.24° (*c* 2.11, $CHCl_3$); 96:04 *er*; Chiral HPLC analysis of the product: Daicel Chiralpak IA 250X4.6 mm 5μ column; hexane/2-propanol = 99/01, detected at 244 nm, Flow rate = 1 mL/min, Retention times: 50.016 min (major), 22.494 min (minor).

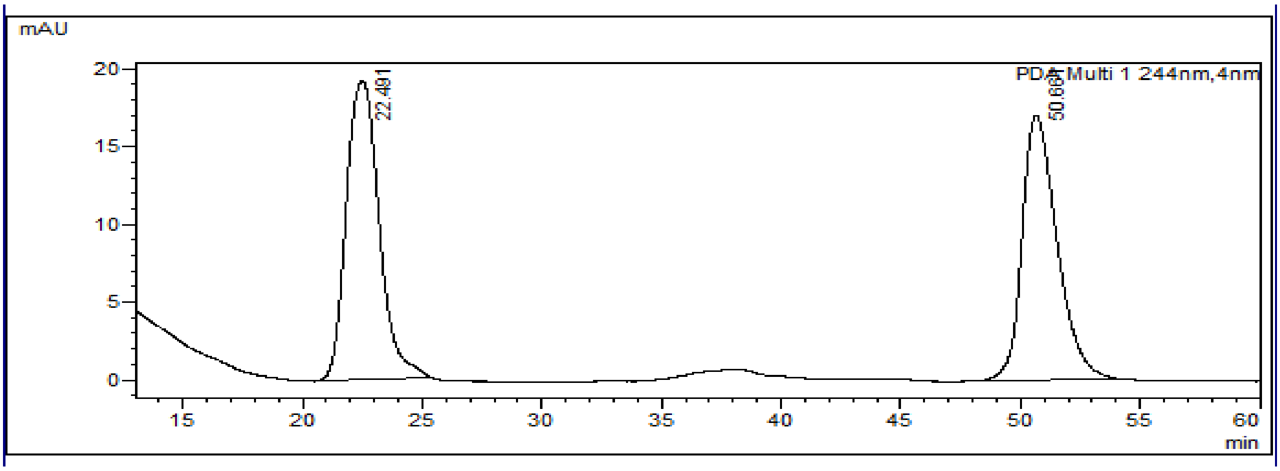

<Peak Table>

PDA Ch1 244nm

Peak#	Ret. Time	Area	Height	Area%	Height%
1	22.491	1841352	19224	51.626	53.088
2	50.661	1725365	16987	48.374	46.912
Total		3566717	36211	100.000	100.000

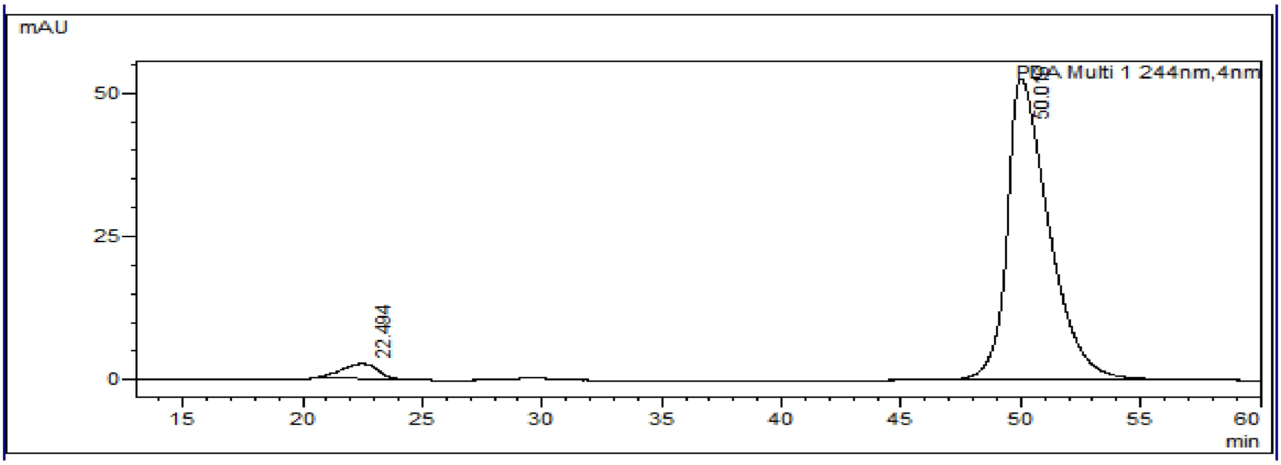

<Peak Table>

PDA Ch1 244nm

Peak#	Ret. Time	Area	Height	Area%	Height%
1	22.494	279416	2606	4.241	4.739
2	50.016	6308472	52373	95.759	95.261
Total		6587887	54979	100.000	100.000

2d. General Procedure for the one-pot sequential borylative cyclization/oxidation reaction

A solution of $Cu(CH_3CN)_4PF_6$ (2.8 mg, 2.5 mol%), (*S*)-SEGPHOS (9 mg, 5 mol%), $B_2(pin)_2$ (84 mg, 0.33 mmol) and ^{t}BuOLi (0.53 µl, 0.6 mmol, 1 M in THF) in dry THF (2.0 mL) was stirred at room temperature for 15 min and then maintained at -78 ^oC. A solution of enone **1** (0.3 mmol) in dry THF (1.0 mL) was added via syringe and the resulting mixture was stirred at -78 ^oC for 3 h then $NaBO_3{\cdot}H_2O$ (150 mg, 1.5 mmol) in H_2O (2 mL) was added in one portion and the resulting was mixture stirred vigorously at room temperature for 3 h under open to air. The reaction mixture was quenched with saturated NH_4Cl solution (10 mL) and extracted with EtOAc (3 × 15 mL) and dried over anhydrous Na_2SO_4, filtered, and concentrated in *vacuo*. The resultant crude product was purified by column chromatography (hexanes/EtOAc).

(3S,4S,4aS,8aR)-4-Benzoyl-3-hydroxy-8a-methyl-3,4,4a,8a-tetrahydro-2*H*-chromen-6(5*H*)-one (10a):

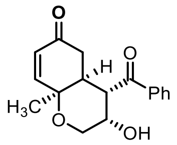

Prepared according to the general procedure as described above in 89% yield (76 mg). It was purified by flash chromatography (50% EtOAc/hexanes; R_f = 0.3) to afford a white solid; *dr* = >30:1; mp = 154–156°C; ^{1}H NMR (400 MHz, $CDCl_3$) δ 7.84 (d, *J* = 7.6 Hz, 2H), 7.67 – 7.56 (m, 1H), 7.54 – 7.40 (m, 2H), 6.84 (dd, *J* = 10.4, 2.3 Hz, 1H), 6.15 (d, *J* = 10.3 Hz, 1H), 3.94 (br.s, 1H), 3.89 (dd, *J* = 12.4, 2.3 Hz, 1H), 3.75 (d, *J* = 12.3 Hz, 1H), 3.66 (dd, *J* = 11.9, 2.7 Hz, 1H), 3.06 (ddt, *J* = 11.9, 4.8, 2.2 Hz, 1H), 2.78 – 2.69 (m, 2H), 2.26 (d, *J* = 17.7 Hz, 1H), 1.61 (s, 3H); ^{13}C NMR (101 MHz, $CDCl_3$) δ 199.9, 197.1, 154.4, 136.3, 134.0, 131.1, 129.1, 128.4, 74.1, 67.5, 65.9, 47.3, 40.0, 36.8, 26.4; HRMS (ESI) calcd for $C_{17}H_{19}O_4$ $[M+H]^+$: 287.1278; found: 287.1270; $[\alpha]^{20}_D$ = -45.43° (*c* 0.96, $CHCl_3$); 95:05 *er*; Chiral HPLC analysis of the product: Daicel Chiralpak IC 250X4.6 mm 5µ column; hexane/2-propanol = 60/40, detected at 254 nm, Flow rate = 1 mL/min, Retention times: 17.676 min (major), 9.671 min (minor).

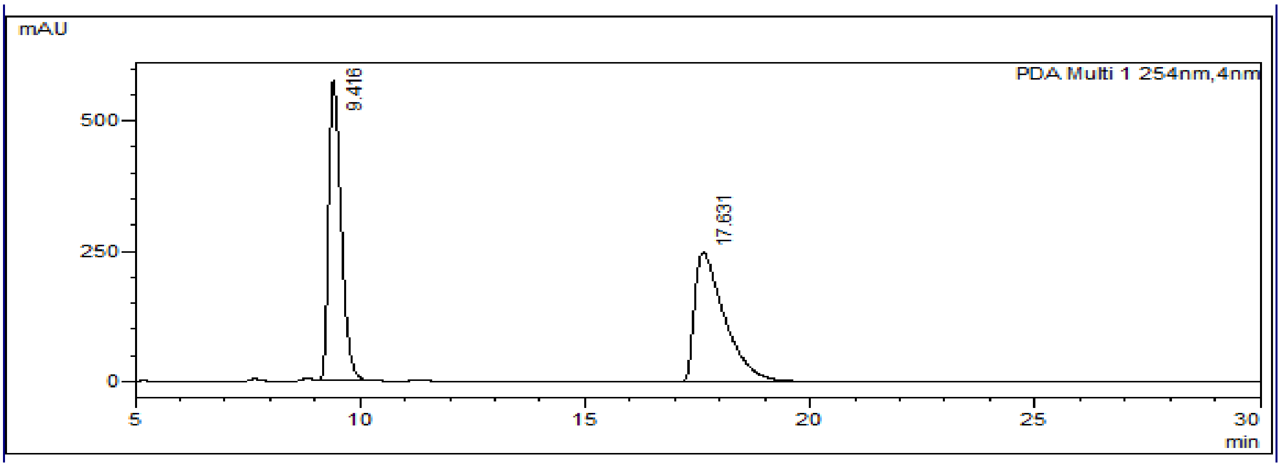

<Peak Table>

PDA Ch1 254nm

Peak#	Ret. Time	Area	Height	Area%	Height%
1	9.416	11237125	575675	49.514	69.966
2	17.631	11457882	247118	50.486	30.034
Total		22695006	822793	100.000	100.000

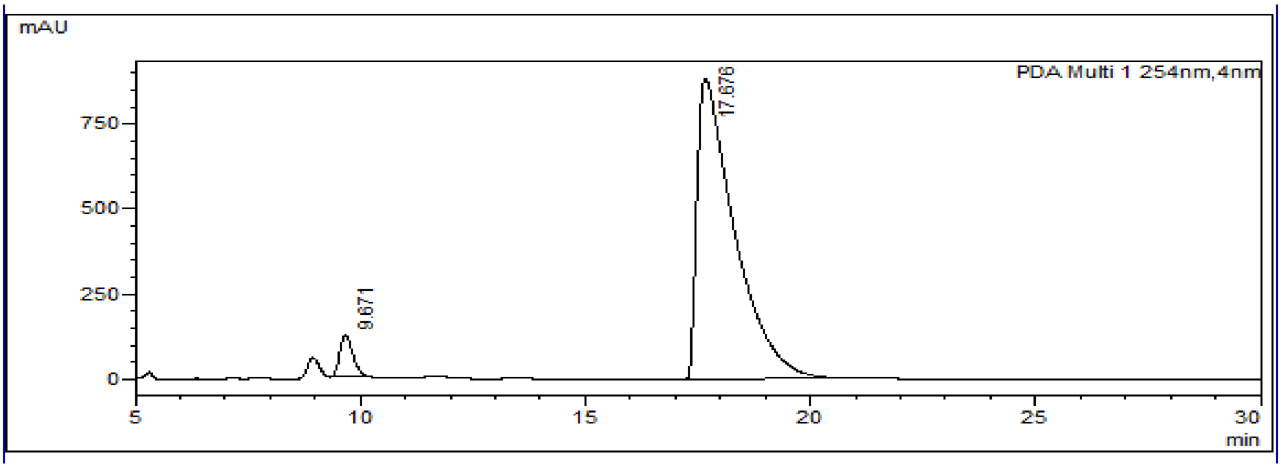

<Peak Table>

PDA Ch1 254nm

Peak#	Ret. Time	Area	Height	Area%	Height%
1	9.671	2465164	122451	4.595	12.191
2	17.676	51182459	882012	95.405	87.809
Total		53647623	1004463	100.000	100.000

(3S,4S,4aS,8aR)-4-Benzoyl-3-hydroxy-8a-propyl-3,4,4a,8a-tetrahydro-2*H*-chromen-6(5*H*)-one (10b):

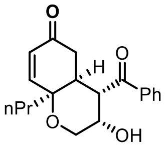

Prepared according to the general procedure as described above in 89% yield (84 mg). It was purified by flash chromatography (50% EtOAc/hexanes; R_f = 0.2) to afford a colourless semi solid; *dr* = >30:1; ^{1}H NMR (400 MHz, $CDCl_3$) δ 7.84 (dd, *J* = 8.3, 1.2 Hz, 2H), 7.64 – 7.54 (m, 1H), 7.52 – 7.42 (m, 2H), 6.89 (dd, *J* = 10.4, 2.2 Hz, 1H), 6.16 (dd, *J* = 10.4, 1.2 Hz, 1H), 3.93 (br.s, 1H), 3.90 (dd, *J* = 15.0, 2.9 Hz, 1H), 3.74 (d, *J* = 11.5 Hz, 1H), 3.68 (dd, *J* = 11.8, 2.7 Hz, 1H), 3.17 – 3.04 (m, 1H), 2.72 (dt, *J* = 10.9, 5.5 Hz, 2H), 2.27 – 2.17 (m, 1H), 1.91 (ddd, *J* = 13.6, 12.0, 4.8 Hz, 1H), 1.86 – 1.66 (m, 2H), 1.57 – 1.43 (m, 1H), 0.97 (t, *J* = 7.3 Hz, 3H); ^{13}C NMR (101 MHz, $CDCl_3$) δ 200.0, 197.2, 154.6, 136.3, 134.0, 131.3, 129.1, 128.4, 75.9, 67.4, 65.9, 47.3, 42.0, 39.7, 34.7, 16.8, 14.6; HRMS (ESI) calcd for $C_{19}H_{23}O_4$ $[M+H]^+$: 315.1591; found: 315.1580; $[\alpha]^{20}_D$ = -51.40° (*c* 0.92, $CHCl_3$); 91:09 *er*; Chiral HPLC analysis of the product: Daicel Chiralpak IA 250X4.6 mm 5μ column; hexane/2-propanol = 80/20, detected at 254 nm, Flow rate = 1 mL/min, Retention times: 24.863 min (major), 8.832 min (minor).

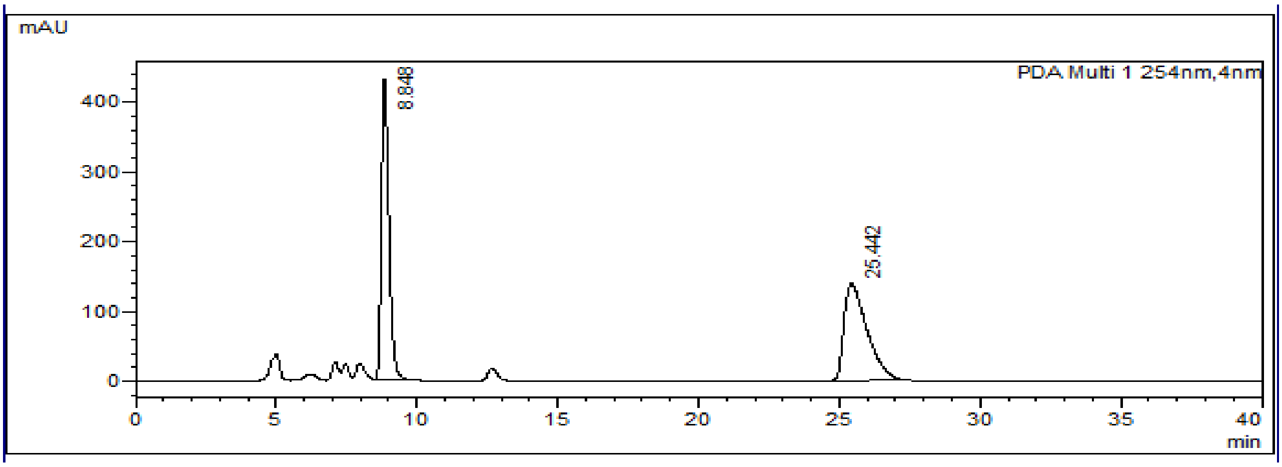

<Peak Table>

PDA Ch1 254nm

Peak#	Ret. Time	Area	Height	Area%	Height%
1	8.848	7900316	429599	50.174	75.635
2	25.442	7845624	138387	49.826	24.365
Total		15745940	567986	100.000	100.000

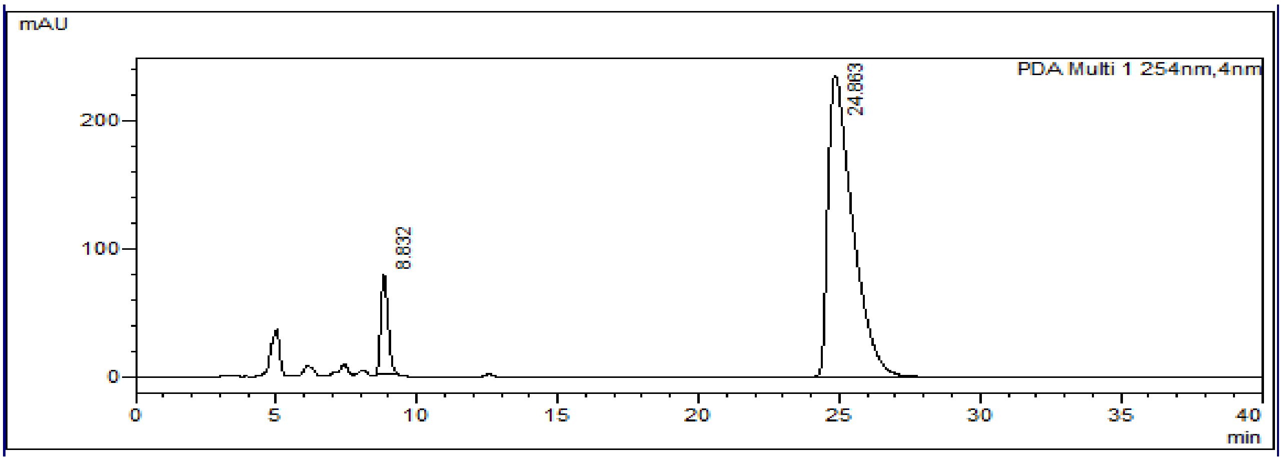

<Peak Table>

PDA Ch1 254nm

Peak#	Ret. Time	Area	Height	Area%	Height%
1	8.832	1388101	78090	8.788	24.967
2	24.863	14407661	234679	91.212	75.033
Total		15795762	312769	100.000	100.000

(3S,4S,4aS,8aR)-4-Benzoyl-3-hydroxy-8a-isopropyl-3,4,4a,8a-tetrahydro-2*H*-chromen-6(5*H*)-one (10c):

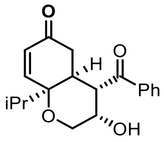

Prepared according to the general procedure as described above in 80% yield (75 mg). It was purified by flash chromatography (50% EtOAc/hexanes; R_f = 0.3) to afford a white semi solid; *dr* = >30:1; ^{1}H

NMR (300 MHz, $CDCl_3$) δ 7.83 (d, *J* = 7.6 Hz, 2H), 7.59 (t, *J* = 7.3 Hz, 1H), 7.46 (t, *J* = 7.6 Hz, 2H), 6.99 (dd, *J* = 10.5, 2.0 Hz, 1H), 6.19 (d, *J* = 10.5 Hz, 1H), 3.90 (br.s, 1H), 3.90 (d, *J* = 10.6 Hz, 1H), 3.75 (d, *J* = 11.9 Hz, 1H), 3.68 (dd, *J* = 11.8, 2.2 Hz, 1H), 3.41 – 3.22 (m, 1H), 2.71 (dd, *J* = 18.2, 5.2 Hz, 1H), 2.60 (d, *J* = 6.0 Hz, 1H), 2.31 – 2.11 (m, 2H), 1.20 (d, *J* = 6.8 Hz, 3H), 1.09 (d, *J* = 6.8 Hz, 3H); ^{13}C NMR (101 MHz, $CDCl_3$) δ 200.0, 197.0, 155.9, 136.4, 133.9, 131.2, 129.1, 128.4, 77.3, 67.2, 66.0, 47.8, 39.0, 36.1, 32.2, 17.4, 17.0; HRMS (ESI) calcd for $C_{19}H_{23}O_4$ $[M+H]^+$: 315.1591; found: 315.1582; $[\alpha]^{20}_D$ = -60.41° (*c* 1.72, $CHCl_3$); 90:10 *er*; Chiral HPLC analysis of the product: Daicel Chiralpak IC 250X4.6 mm 5μ column; hexane/2-propanol = 80/20, detected at 254 nm, Flow rate = 1 mL/min, Retention times: 30.001 min (major), 13.676 min (minor).

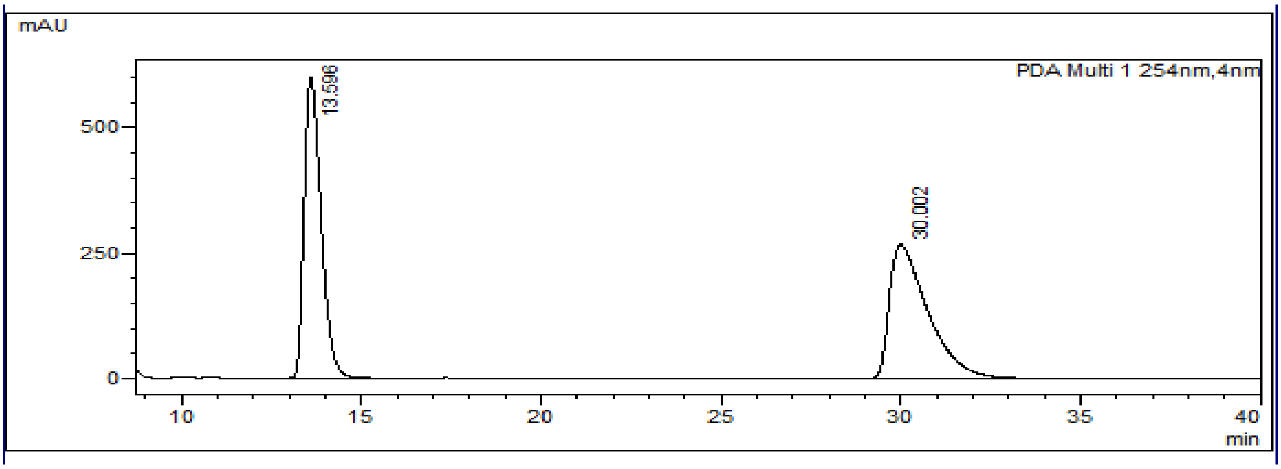

<Peak Table>

PDA Ch1 254nm

Peak#	Ret. Time	Area	Height	Area%	Height%
1	13.596	19686465	598984	49.808	69.209
2	30.002	19837935	266489	50.192	30.791
Total		39524400	865473	100.000	100.000

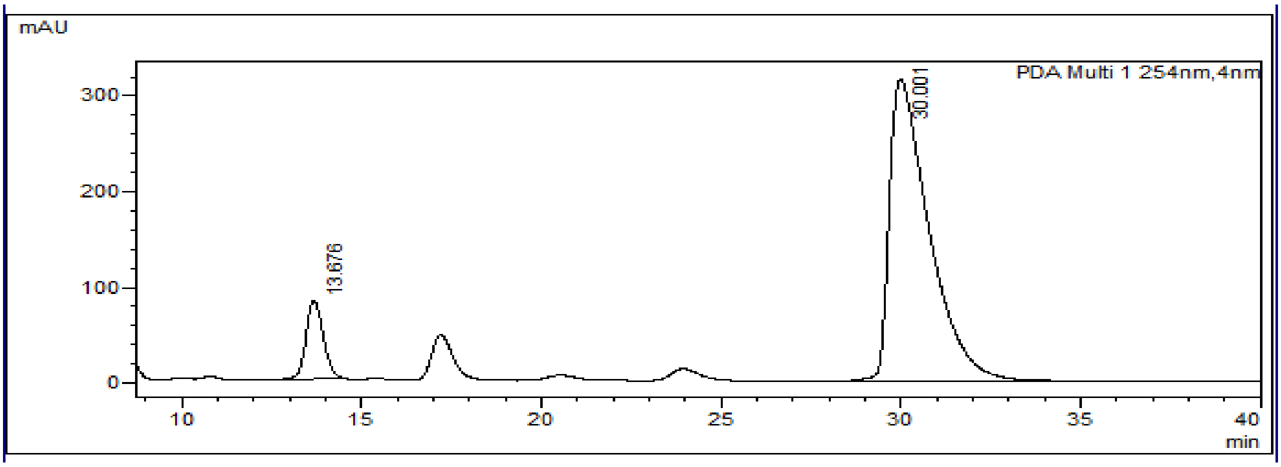

<Peak Table>

PDA Ch1 254nm

Peak#	Ret. Time	Area	Height	Area%	Height%
1	13.676	2744089	82159	10.159	20.669
2	30.001	24268504	315345	89.841	79.331
Total		27012593	397504	100.000	100.000

(3S,4S,4aS,8aR)-4-Benzoyl-3-hydroxy-8a-(4-pentylcyclohexyl)-3,4,4a,8a-tetrahydro-2*H*-chromen-6(5*H*)-one (10d):

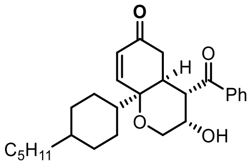

Prepared according to the general procedure as described above in 90% yield (114 mg). It was purified by flash chromatography (50% EtOAc/hexanes; R_f = 0.3) to afford a colourless semi solid; *dr* = >30:1; ^{1}H NMR (500 MHz, $CDCl_3$) δ 7.83 (dd, *J* = 8.4, 1.2 Hz, 2H), 7.58 (ddd, *J* = 8.6, 2.4, 1.2 Hz, 1H), 7.50 – 7.37 (m, 2H), 6.99 (dd, *J* = 10.5, 2.2 Hz, 1H), 6.18 (dd, *J* = 10.5, 1.1 Hz, 1H), 3.90 (br.s, 1H), 3.87 (dd, *J* = 12.4, 1.8 Hz, 1H), 3.73 (d, *J* = 11.5 Hz, 1H), 3.68 (dd, *J* = 11.7, 2.6 Hz, 1H), 3.40 – 3.31 (m, 1H), 2.72 (dd, *J* = 18.2, 5.3 Hz, 1H), 2.57 (d, *J* = 7.0 Hz, 1H), 2.21 – 2.14 (m, 1H), 2.10 – 2.00 (m, 1H), 1.92 – 1.78 (m, 3H), 1.78 – 1.69 (m, 1H), 1.69 – 1.44 (m, 3H), 1.35 – 1.12 (m, 9H), 0.88 (t, *J* = 7.1 Hz, 3H), 0.97 – 0.81 (m, 1H); ^{13}C NMR (101 MHz, $CDCl_3$) δ 200.0, 197.2, 156.1, 136.3, 133.9, 131.0, 129.1, 128.4, 67.2, 65.9, 47.8, 46.1, 39.0, 37.3, 33.4, 33.3, 32.3, 31.5, 26.7, 26.7, 26.4, 22.8, 14.3; HRMS (ESI) calcd for $C_{27}H_{37}O_4$ $[M+H]^+$: 425.2686; found: 425.2668; $[\alpha]^{20}_D$ = -52.12° (*c* 1.32, $CHCl_3$); 91:09 *er*; Chiral HPLC analysis of the product: Daicel Chiralpak IC 250X4.6 mm 5µ column; hexane/2-propanol = 80/20, detected at 254 nm, Flow rate = 1 mL/min, Retention times: 19.612 min (major), 11.369 min (minor).

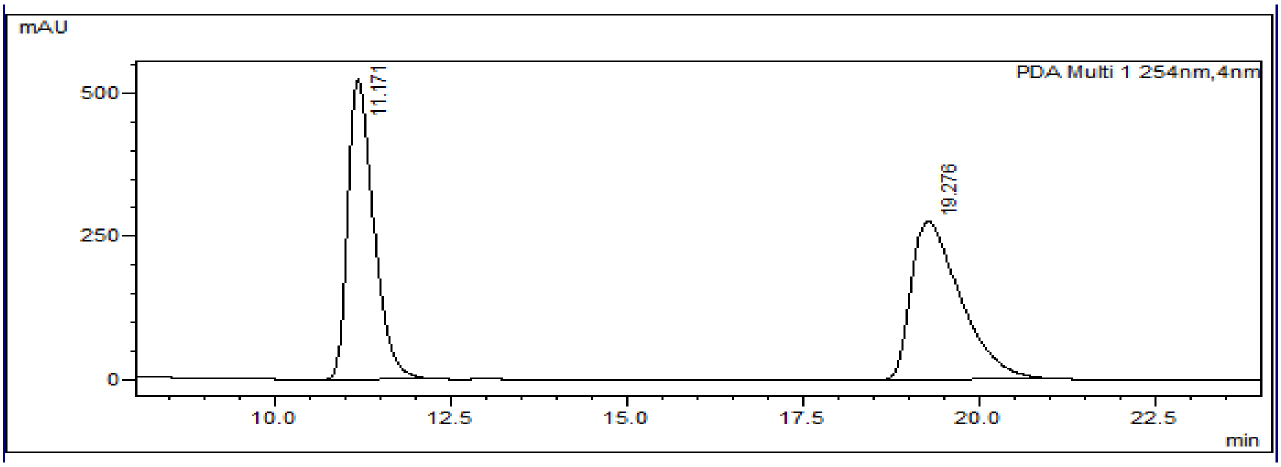

<Peak Table>

PDA Ch1 254nm

Peak#	Ret. Time	Area	Height	Area%	Height%
1	11.171	13508186	522806	49.938	65.472
2	19.276	13541909	275708	50.062	34.528
Total		27050095	798514	100.000	100.000

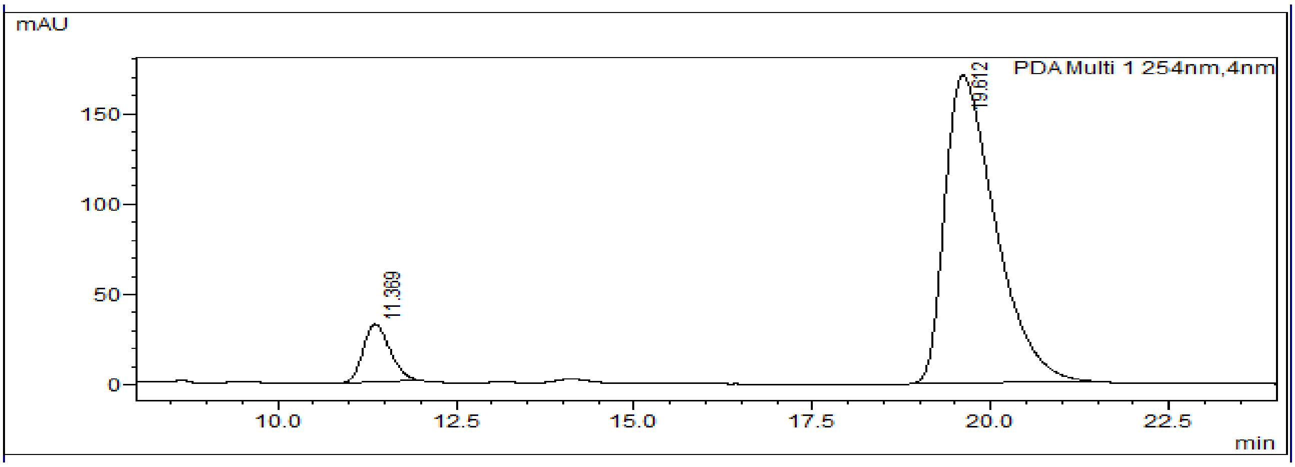

<Peak Table>

PDA Ch1 254nm

Peak#	Ret. Time	Area	Height	Area%	Height%
1	11.369	820298	31739	8.857	15.706
2	19.612	8440830	170345	91.143	84.294
Total		9261128	202084	100.000	100.000

(3S,4S,4aS,8aR)-4-Benzoyl-8a-benzyl-3-hydroxy-3,4,4a,8a-tetrahydro-2*H*-chromen-6(5*H*)-one (10e):

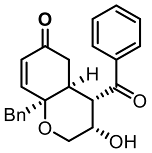

Prepared according to the general procedure as described above in 85% yield (92 mg). It was purified by flash chromatography (50% EtOAc/hexanes; R_f = 0.3) to afford a colourless semi solid; *dr* = 23:1; ^{1}H NMR (500 MHz, $CDCl_3$) δ 7.81 (dd, *J* = 8.4, 1.2 Hz, 2H), 7.61 – 7.55 (m, 1H), 7.48 – 7.42 (m, 2H), 7.35 – 7.28 (m, 5H), 6.64 (dd, *J* = 10.4, 2.2 Hz, 1H), 6.21 (dd, *J* = 10.4, 1.2 Hz, 1H), 3.93 – 3.86 (m, 2H), 3.70 (dd, *J* = 9.2, 3.7 Hz, 1H), 3.64 (dd, *J* = 11.8, 2.7 Hz, 1H), 3.25 (d, *J* = 13.8 Hz, 1H), 3.12 (d, *J* = 13.8 Hz, 1H), 3.10 – 3.04 (m, 1H), 2.81 (dd, *J* = 17.9, 5.3 Hz, 1H), 2.43 (d, *J* = 7.5 Hz, 1H), 2.32 – 2.23 (m, 1H); ^{13}C NMR (101 MHz, $CDCl_3$) δ 199.2, 197.1, 154.0, 136.2, 135.4, 133.9, 131.7, 130.8, 129.1, 128.4, 128.3, 127.3, 76.0, 67.4, 65.9, 47.7, 45.4, 39.8, 34.2; HRMS (ESI) calcd for $C_{23}H_{23}O_4$ $[M+H]^+$: 363.1591; found: 363.1583; $[\alpha]^{20}_D$ = -45.33° (*c* 2.11, $CHCl_3$); 90:10 *er*; Chiral HPLC analysis of the product: Daicel Chiralpak OD-H 250X4.6 mm 5µ column; hexane/2-propanol

= 80/20, detected at 254 nm, Flow rate = 1 mL/min, Retention times: 34.662 min (major), 14.695 min (minor).

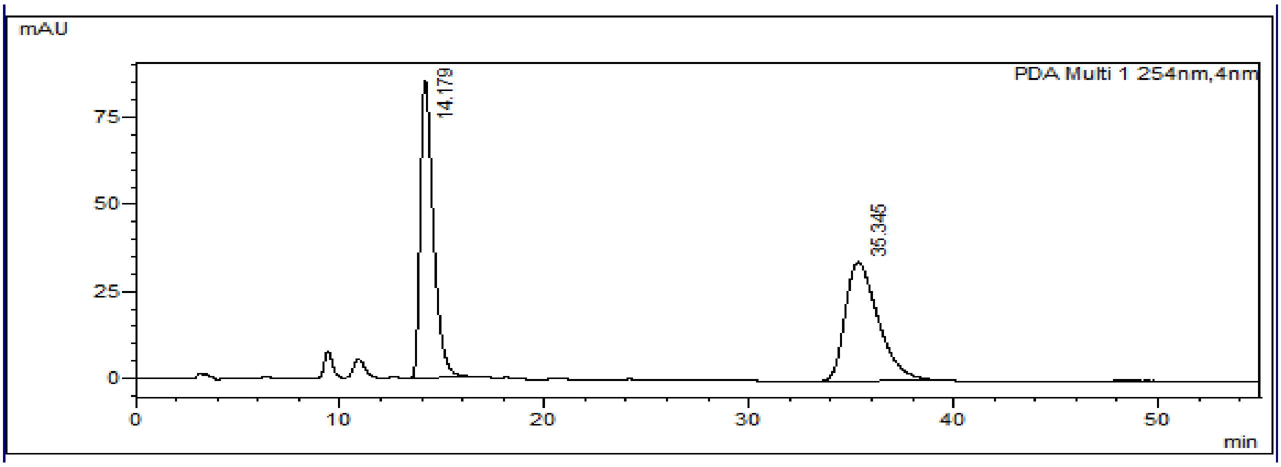

<Peak Table>

PDA Ch1 254nm

Peak#	Ret. Time	Area	Height	Area%	Height%
1	14.179	3829767	85506	50.031	71.497
2	35.345	3825055	34088	49.969	28.503
Total		7654822	119594	100.000	100.000

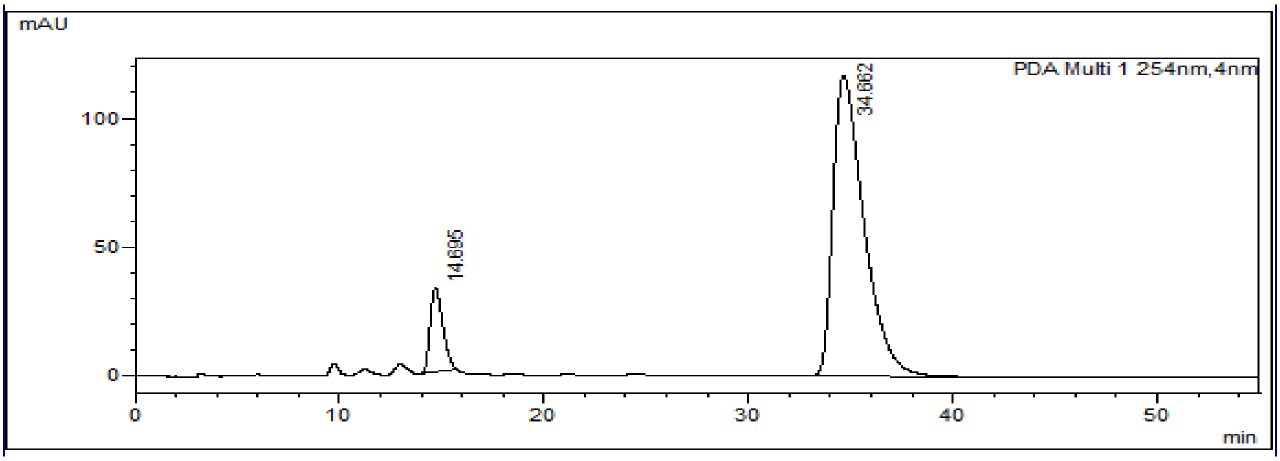

<Peak Table>

PDA Ch1 254nm

Peak#	Ret. Time	Area	Height	Area%	Height%
1	14.695	1417653	32771	10.328	21.938
2	34.662	12308359	116606	89.672	78.062
Total		13726012	149376	100.000	100.000

(3S,4S,4aS,8aR)-4-Benzoyl-3-hydroxy-8a-phenyl-3,4,4a,8a-tetrahydro-2*H*-chromen-6(5*H*)-one (10f):

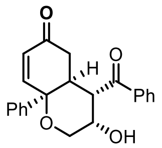

Prepared according to the general procedure as described above in 96% yield (100 mg). It was purified by flash chromatography (50% EtOAc/hexanes; R_f = 0.3) to afford a colourless semi solid; *dr* = >30:1; ^{1}H NMR (500 MHz, $CDCl_3$) δ 7.86 (dd, *J* = 8.4, 1.2 Hz, 2H), 7.65 – 7.56 (m, 3H), 7.53 – 7.45 (m, 2H), 7.44 – 7.33 (m, 3H), 6.98 (dd, *J* = 10.4, 2.3 Hz, 1H), 6.54 (dd, *J* = 10.4, 1.3 Hz, 1H), 4.12 (dd, *J* = 12.5, 2.3 Hz, 1H), 4.05 (br.s, 1H), 3.95 (dd, *J* = 12.4, 0.8 Hz, 1H), 3.84 (dd, *J* = 11.9, 2.6 Hz, 1H), 3.28 (ddt, *J* = 11.9, 4.7, 2.3 Hz, 1H), 2.97 (br.s, 1H), 2.43 (dd, *J* = 17.4, 4.9 Hz, 1H), 2.09 (ddd, *J* = 17.4, 2.2, 1.4 Hz, 1H); ^{13}C NMR (101 MHz, $CDCl_3$) δ 200.3, 197.8, 151.2, 140.3, 136.2, 134.2, 133.4, 129.2, 128.9, 128.8, 128.5, 126.6, 78.6, 67.8, 65.6, 47.52, 39.1, 38.6; HRMS (ESI) calcd for $C_{22}H_{21}O_4$ $[M+H]^+$:349.1434; found: 349.1428; $[\alpha]^{20}_D$ = -28.37° (*c* 1.20, $CHCl_3$); 93:07 *er*; Chiral HPLC analysis of the product: Daicel Chiralpak IA 250X4.6 mm 5µ column; hexane/2-propanol = 80/20, detected at 244 nm, Flow rate = 1 mL/min, Retention times: 13.755 min (major), 11.548 min (minor).

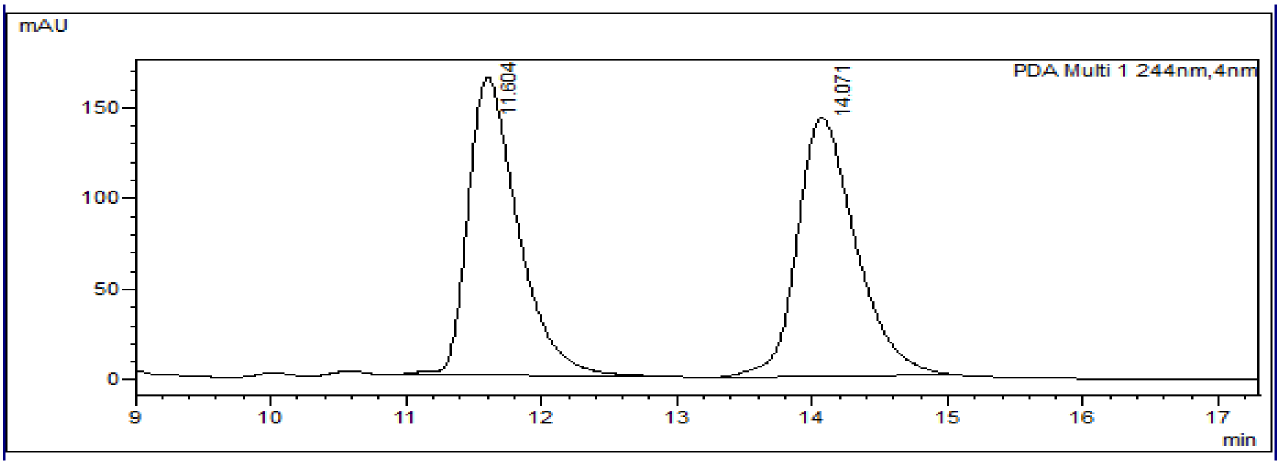

<Peak Table>

PDA Ch1 244nm

Peak#	Ret. Time	Area	Height	Area%	Height%
1	11.604	4192132	163869	49.491	53.502
2	14.071	4278350	142415	50.509	46.498
Total		8470482	306284	100.000	100.000

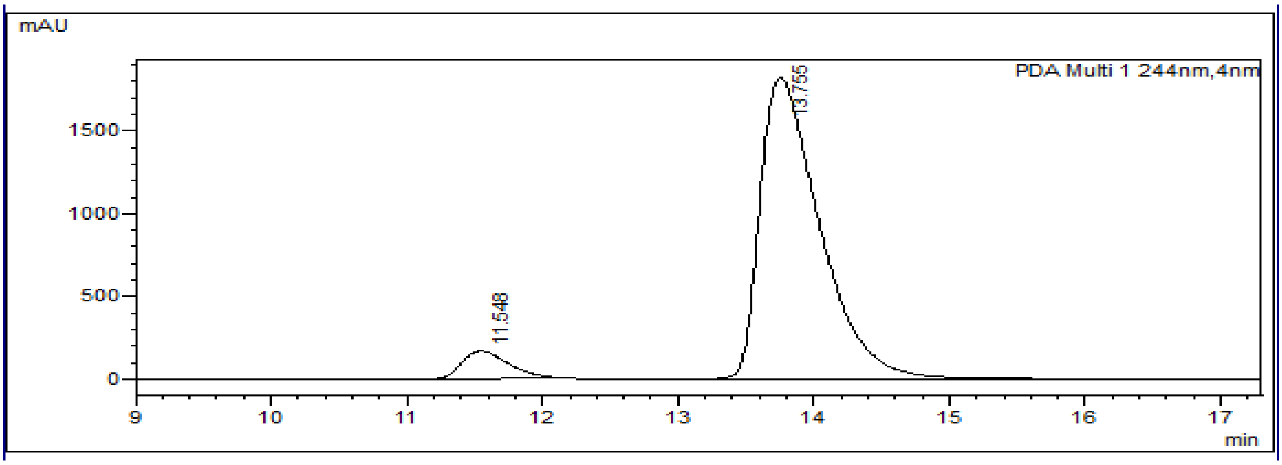

<Peak Table>

PDA Ch1 244nm

Peak#	Ret. Time	Area	Height	Area%	Height%
1	11.548	3996958	167310	6.649	8.421
2	13.755	56112227	1819452	93.351	91.579
Total		60109185	1986762	100.000	100.000

(3S,4S,4aS,8aR)-3-Hydroxy-8a-methyl-4-(4-methylbenzoyl)-3,4,4a,8a-tetrahydro-2*H*-chromen-6(5*H*)-one (10g):

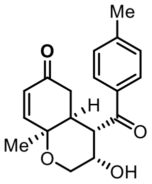

Prepared according to the general procedure as described above in 82% yield (74 mg). It was purified by flash chromatography (50% EtOAc/hexanes; $R_f = 0.3$) to afford a white solid; *dr* = >30:1; mp = 158–160°C; ^{1}H NMR (400 MHz, $CDCl_3$) δ 7.73 (d, *J* = 8.3 Hz, 2H), 7.25 (d, *J* = 8.0 Hz, 2H), 6.83 (dd, *J* = 10.4, 2.2 Hz, 1H), 6.12 (dd, *J* = 10.3, 1.2 Hz, 1H), 3.91 (br.s, 1H), 3.88 (dd, *J* = 15.3, 3.1 Hz, 1H), 3.72 (d, *J* = 11.5 Hz, 1H), 3.64 (dd, *J* = 11.9, 2.7 Hz, 1H), 3.12 – 2.99 (m, 1H), 2.93 (d, *J* = 5.6 Hz, 1H), 2.70 (dd, *J* = 17.7, 5.2 Hz, 1H), 2.40 (s, 3H), 2.28 – 2.13 (m, 1H), 1.59 (s, 3H); ^{13}C NMR (101 MHz, $CDCl_3$) δ 199.7, 197.1, 154.6, 145.1, 133.7, 131.0, 129.8, 128.5, 74.0, 67.5, 65.9, 46.8, 39.9, 36.9, 26.4, 21.8; HRMS (ESI) calcd for $C_{18}H_{21}O_4$ $[M+H]^+$: 301.1434; found: 301.1418; $[\alpha]^{20}_D$ = -10.23° (*c* 0.82, $CHCl_3$); 90:10 *er*; Chiral HPLC analysis of the product: Daicel Chiralpak OD-H 250X4.6 mm 5μ column; hexane/2-propanol = 80/20, detected at 254 nm, Flow rate = 1 mL/min, Retention times: 30.559 min (major), 15.395 min (minor).

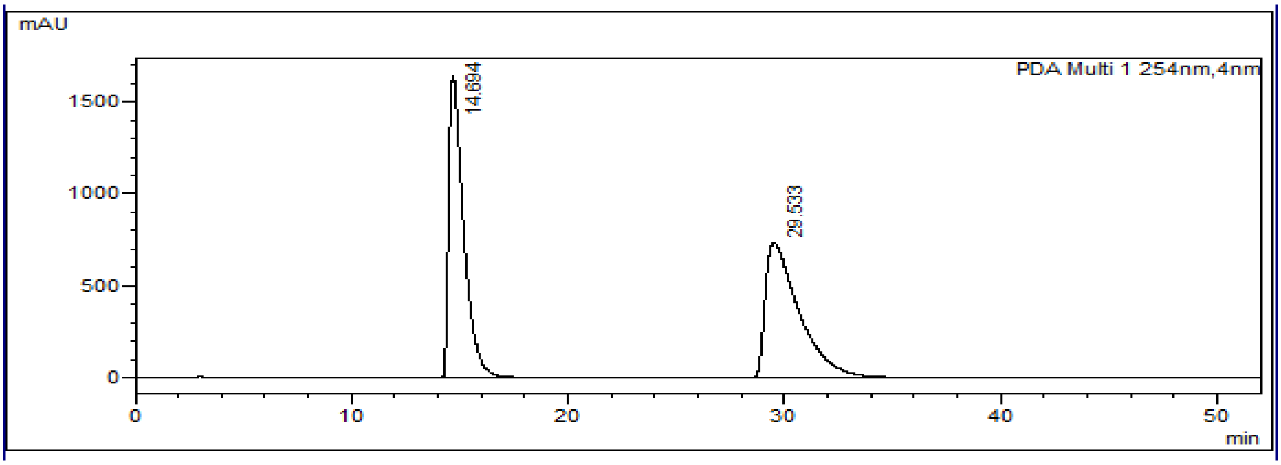

<Peak Table>

PDA Ch1 254nm

Peak#	Ret. Time	Area	Height	Area%	Height%
1	14.694	78997003	1640955	49.964	69.096
2	29.533	79111382	733939	50.036	30.904
Total		158108385	2374894	100.000	100.000

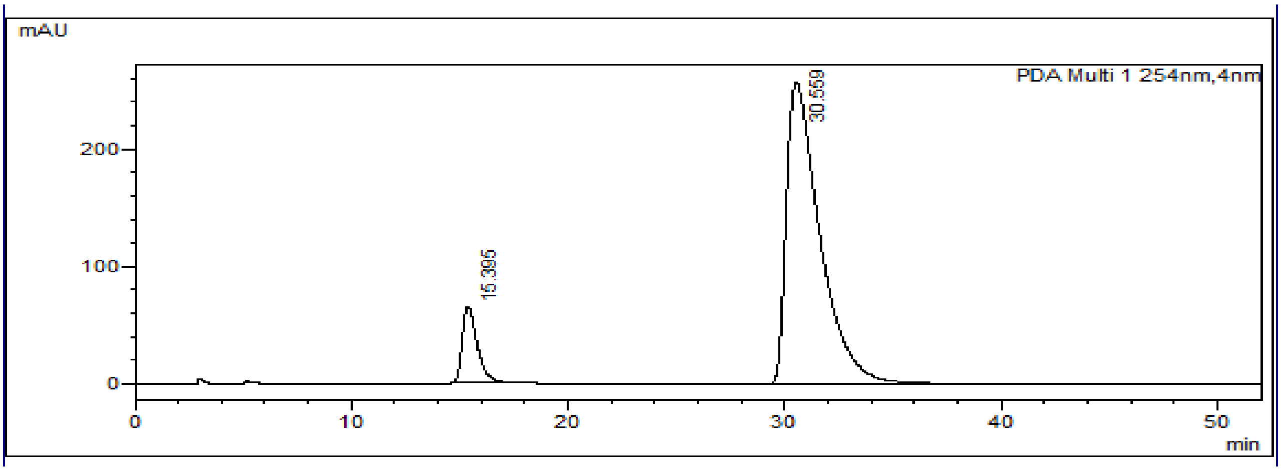

<Peak Table>

PDA Ch1 254nm

Peak#	Ret. Time	Area	Height	Area%	Height%
1	15.395	3042264	64546	9.872	20.080
2	30.559	27773537	256903	90.128	79.920
Total		30815801	321449	100.000	100.000

(3S,4S,4aS,8aR)-3-Hydroxy-4-(4-methoxybenzoyl)-8a-methyl-3,4,4a,8a-tetrahydro-2*H*-chromen-6(5*H*)-one (10h):

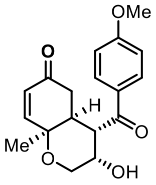

Prepared according to the general procedure as described above in 88% yield (84 mg). It was purified by flash chromatography (50% EtOAc/hexanes; R_f = 0.2) to afford a white semi solid; *dr* = >30:1; ^{1}H NMR (500 MHz, $CDCl_3$) δ 7.84 (d, *J* = 9.0 Hz, 2H), 6.94 (d, *J* = 9.0 Hz, 2H), 6.85 (dd, *J* = 10.3, 2.2 Hz, 1H), 6.14 (dd, *J* = 10.3, 1.2 Hz, 1H), 3.94 – 3.89 (m, 2H), 3.87 (s, 3H), 3.71 (dd, *J* = 12.8, 1.5 Hz, 1H), 3.65 (dd, *J* = 11.9, 2.6 Hz, 1H), 3.18 – 2.96 (m, 2H), 2.70 (dd, *J* = 17.7, 5.2 Hz, 1H), 2.23 – 2.17 (m, 1H), 1.60 (s, 3H); ^{13}C NMR (101 MHz, $CDCl_3$) δ 199.0, 197.2, 164.5, 154.8, 131.0, 130.9, 129.2, 114.3, 74.1, 67.5, 65.9, 55.7, 46.2, 40.0, 37.3, 26.4; HRMS (ESI) calcd for $C_{18}H_{21}O_5$ $[M+H]^+$: 317.1389; found: 317.1386; $[\alpha]^{20}_D$ = -9.87° (*c* 0.98, $CHCl_3$); 89:11 *er*; Chiral HPLC analysis of the product: Daicel Chiralpak OD-H 250X4.6 mm 5µ column; hexane/2-propanol = 75/25, detected at 254 nm, Flow rate = 1 mL/min, Retention times: 38.753 min (major), 21.820 min (minor).

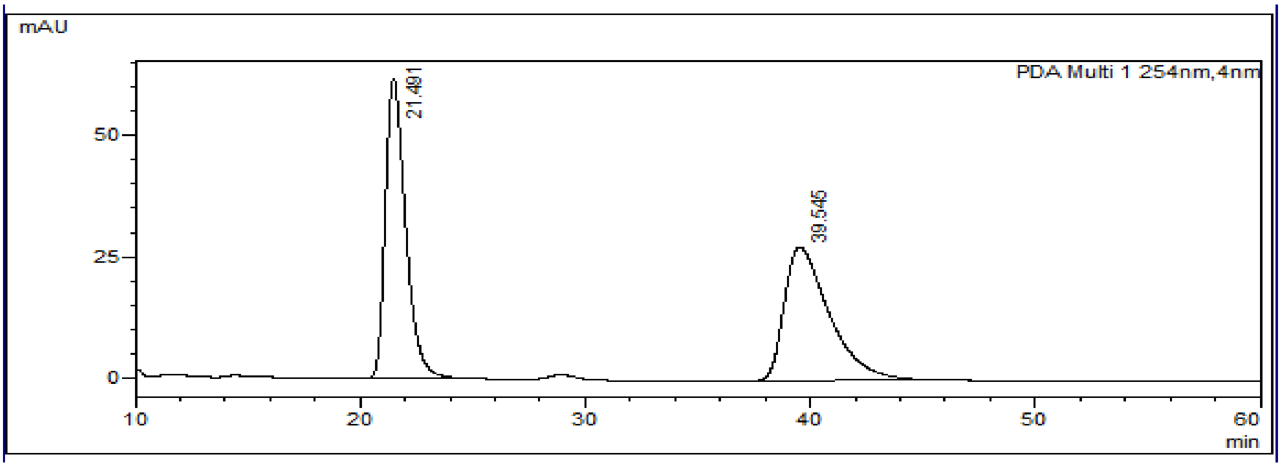

<Peak Table>

PDA Ch1 254nm

Peak#	Ret. Time	Area	Height	Area%	Height%
1	21.491	3838039	61560	50.559	69.255
2	39.545	3753182	27329	49.441	30.745
Total		7591221	88889	100.000	100.000

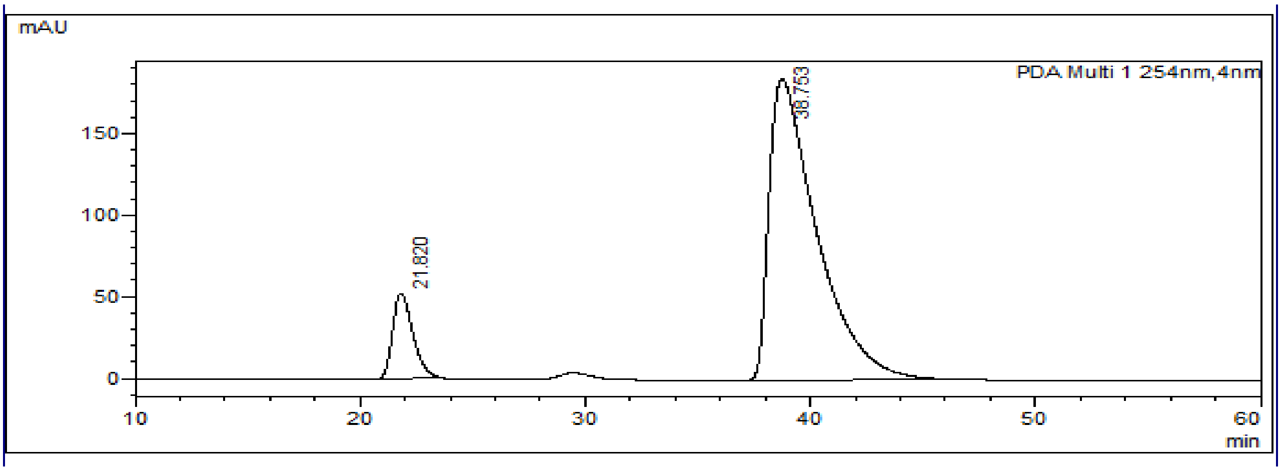

<Peak Table>

PDA Ch1 254nm

Peak#	Ret. Time	Area	Height	Area%	Height%
1	21.820	3255378	52061	10.674	22.020
2	38.753	27241738	184367	89.326	77.980
Total		30497116	236428	100.000	100.000

(3S,4S,4aS,8aR)-4-(4-Chlorobenzoyl)-3-hydroxy-8a-methyl-3,4,4a,8a-tetrahydro-2*H*-chromen-6(5*H*)-one (10i):

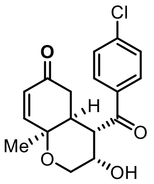

Prepared according to the general procedure as described above in 68% yield (65 mg). It was purified by flash chromatography (50% EtOAc/hexanes; R_f = 0.3) to afford a white semi solid; *dr* = >30:1; ^{1}H NMR (400 MHz, $CDCl_3$) δ 7.78 (d, *J* = 8.7 Hz, 2H), 7.44 (d, *J* = 8.7 Hz, 2H), 6.83 (dd, *J* = 10.4, 2.2 Hz, 1H), 6.14 (dd, *J* = 10.4, 1.3 Hz, 1H), 3.92 (br.s, 1H), 3.89 (dd, *J* = 15.9, 4.1 Hz, 1H), 3.75 (d, *J* = 11.8 Hz, 1H), 3.57 (dd, *J* = 11.8, 2.8 Hz, 1H), 3.05 (ddt, *J* = 7.3, 4.6, 2.1 Hz, 1H), 2.74 (dd, *J* = 17.7, 5.2 Hz, 1H), 2.68 (d, *J* = 6.8 Hz, 1H), 2.30 – 2.19 (m, 1H), 1.61 (s, 3H); ^{13}C NMR (101 MHz, $CDCl_3$) δ 198.4, 197.1, 154.3, 140.6, 134.5, 131.2, 129.8, 129.5, 74.1, 67.5, 65.9, 47.5, 40.1, 36.7, 26.4; HRMS (ESI) calcd for $C_{17}H_{18}ClO_4$ $[M+H]^+$: 321.0894; found: 321.0885; $[\alpha]^{20}_D$ = -41.22° (*c* 1.19, $CHCl_3$); 87:13 *er*; Chiral HPLC analysis of the product: Daicel Chiralpak OD-H 250X4.6 mm 5µ column; hexane/2-propanol = 80/20, detected at 254 nm, Flow rate = 1 mL/min, Retention times: 24.962 min (major), 15.637 min (minor).

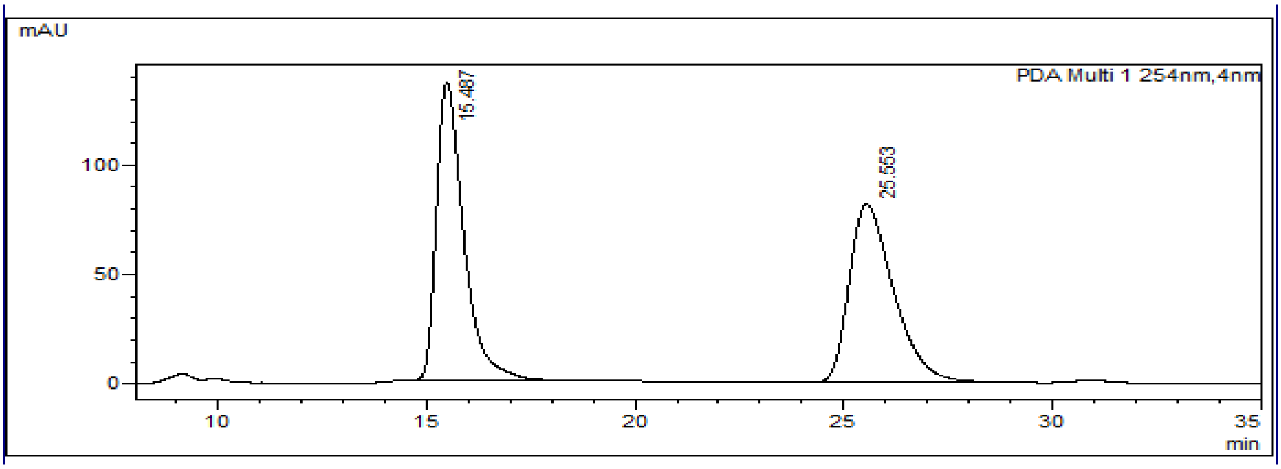

<Peak Table>

PDA Ch1 254nm

Peak#	Ret. Time	Area	Height	Area%	Height%
1	15.487	6244433	136508	50.745	62.591
2	25.553	6061186	81586	49.255	37.409
Total		12305619	218095	100.000	100.000

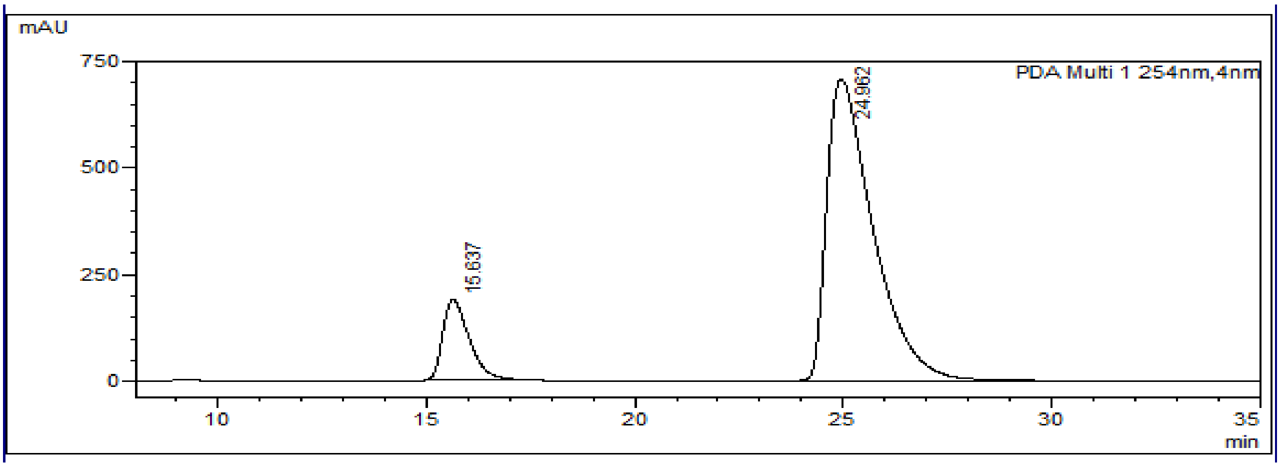

<Peak Table>

PDA Ch1 254nm

Peak#	Ret. Time	Area	Height	Area%	Height%
1	15.637	8173580	188096	12.933	21.012
2	24.962	55024570	707076	87.067	78.988
Total		63198150	895172	100.000	100.000

(3S,4S,4aS,8aR)-4-(4-Bromobenzoyl)-3-hydroxy-8a-methyl-3,4,4a,8a-tetrahydro-2*H*-chromen-6(5*H*)-one (10j):

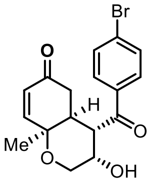

Prepared according to the general procedure as described above in 73% yield (80 mg). It was purified by flash chromatography (10% EtOAc/hexanes; R_f = 0.4) to afford a colourless semi solid; *dr* = >30:1; ^{1}H NMR (400 MHz, $CDCl_3$) δ 7.78 – 7.66 (m, 2H), 7.65 – 7.49 (m, 2H), 6.83 (dd, *J* = 10.4, 2.2 Hz, 1H), 6.14 (dd, *J* = 10.4, 1.2 Hz, 1H), 3.91 (br.s, 1H), 3.89 (dd, *J* = 15.8, 3.6 Hz, 1H), 3.75 (d, *J* = 11.5 Hz, 1H), 3.56 (dd, *J* = 11.8, 2.8 Hz, 1H), 3.04 (ddt, *J* = 7.3, 4.6, 2.1 Hz, 1H), 2.74 (dd, *J* = 17.7, 5.2 Hz, 1H), 2.68 (d, *J* = 6.6 Hz, 1H), 2.28 – 2.18 (m, 1H), 1.61 (s, 3H); ^{13}C NMR (101 MHz, $CDCl_3$) δ 198.6, 197.1, 154.3, 134.9, 132.4, 131.2, 129.8, 129.3, 74.1, 67.5, 65.9, 47.5, 40.1, 36.7, 26.4; HRMS (ESI) calcd for $C_{17}H_{18}BrO_4$ [M+H]$^+$: 365.0383; found: 365.0391; $[\alpha]^{20}_{D}$ = -40.92° (*c* 0.77, $CHCl_3$); Chiral HPLC analysis of the product: Daicel Chiralpak OD-H 250X4.6 mm 5µ column; hexane/2-

propanol = 80/20, detected at 254 nm, Flow rate = 1 mL/min, Retention times: 25.286 min (major), 16.700 min (minor).

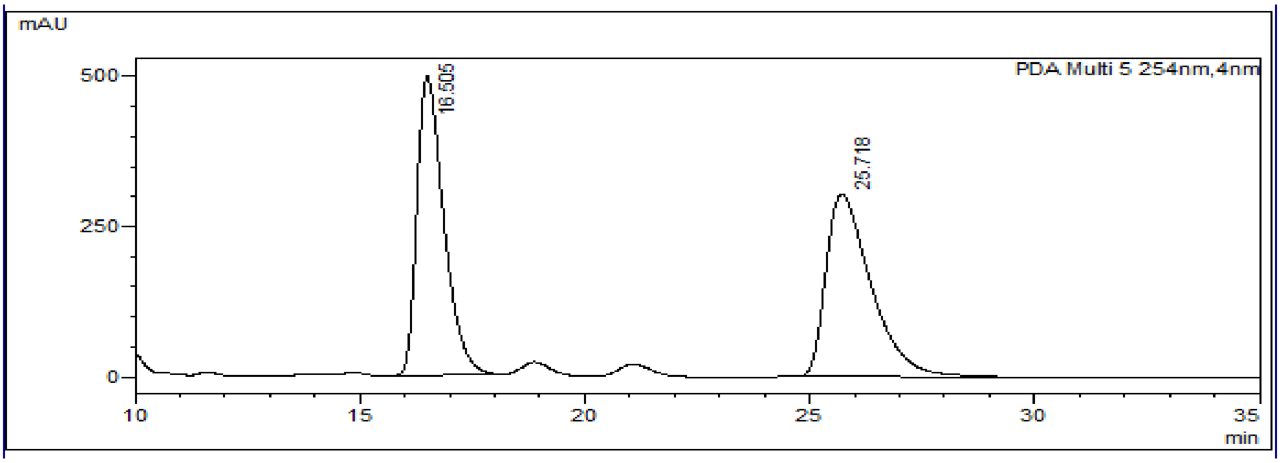

<Peak Table>

PDA Ch5 254nm

Peak#	Ret. Time	Area	Height	Area%	Height%
1	16.505	20730576	498156	49.567	62.152
2	25.718	21093060	303358	50.433	37.848
Total		41823636	801514	100.000	100.000

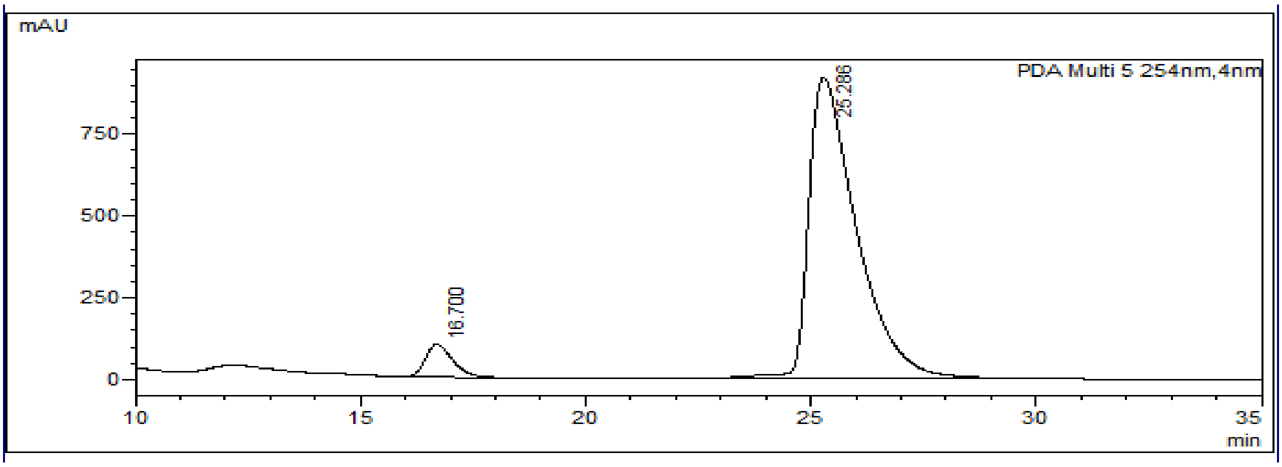

<Peak Table>

PDA Ch5 254nm

Peak#	Ret. Time	Area	Height	Area%	Height%
1	16.700	4106579	98545	5.725	9.686
2	25.286	67618788	918876	94.275	90.314
Total		71725366	1017421	100.000	100.000

(3S,4S,4aS,8aR)-4-([1,1'-Biphenyl]-4-carbonyl)-3-hydroxy-8a-methyl-3,4,4a,8a-tetrahydro-2*H*-chromen-6(5*H*)-one (10k):

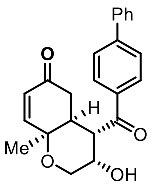

Prepared according to the general procedure as described above in 85% yield (92 mg). It was purified by flash chromatography (50% EtOAc/hexanes; $R_f = 0.3$) to afford a colourless semi solid; *dr* = >30:1; ^{1}H NMR (500 MHz, $CDCl_3$) δ 7.97 – 7.87 (m, 2H), 7.71 – 7.67 (m, 2H), 7.63 – 7.59 (m, 2H), 7.50 – 7.45 (m, 2H), 7.43 – 7.39 (m, 1H), 6.86 (dd, *J* = 10.4, 2.2 Hz, 1H), 6.17 (dd, *J* = 10.4, 1.2 Hz, 1H), 3.98 (br.s, 1H), 3.92 (dd, *J* = 12.4, 2.2 Hz, 1H), 3.77 (dd, *J* = 12.3, 0.8 Hz, 1H), 3.70 (dd, *J* = 11.9, 2.7 Hz, 1H), 3.12 – 3.06 (m, 1H), 2.81 (d, *J* = 6.2 Hz, 1H), 2.75 (dd, *J* = 17.7, 5.3 Hz, 1H), 2.33 – 2.25 (m, 1H), 1.62 (s, 3H); ^{13}C NMR (101 MHz, $CDCl_3$) δ 199.5, 197.1, 154.5, 146.8, 139.7, 134.9, 131.2, 129.1, 129.1, 128.6, 127.8, 127.4, 74.1, 67.6, 66.0, 47.2, 40.1, 36.9, 26.4; HRMS (ESI) calcd for $C_{23}H_{23}O_4$ $[M+H]^+$: 363.1591; found: 363.1586; $[\alpha]^{20}_D$ = -50.6° (*c* 1.00, $CHCl_3$); 97:03 *er*; Chiral HPLC analysis of the product: Daicel Chiralpak OD-H 250X4.6 mm 5μ column; hexane/2-propanol = 75/25, detected at 254 nm, Flow rate = 1 mL/min, Retention times: 32.262 min (major), 26.652 min (minor).

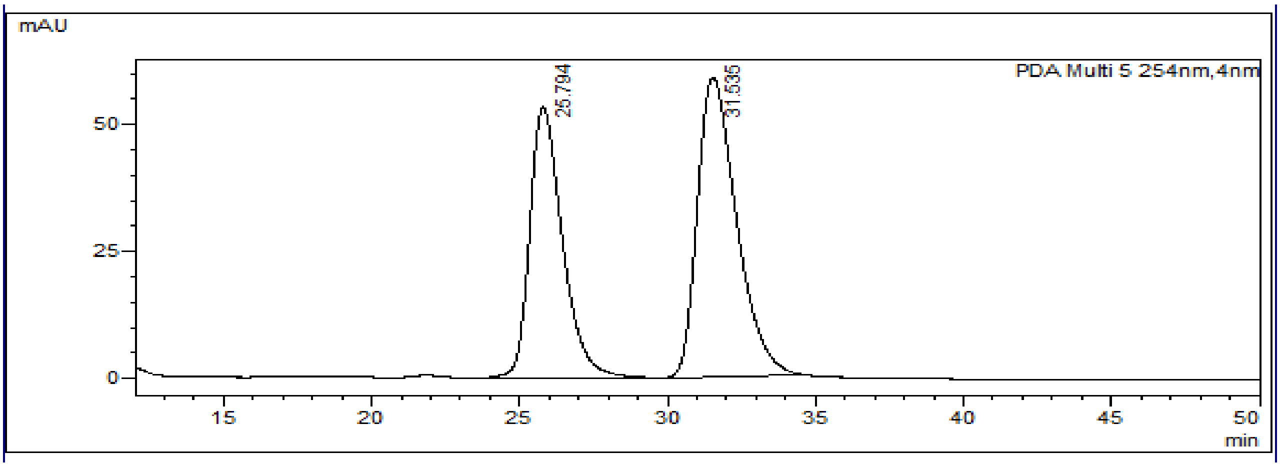

<Peak Table>

PDA Ch5 254nm

Peak#	Ret. Time	Area	Height	Area%	Height%
1	25.794	4120378	53378	43.186	47.575
2	31.535	5420567	58821	56.814	52.425
Total		9540945	112199	100.000	100.000

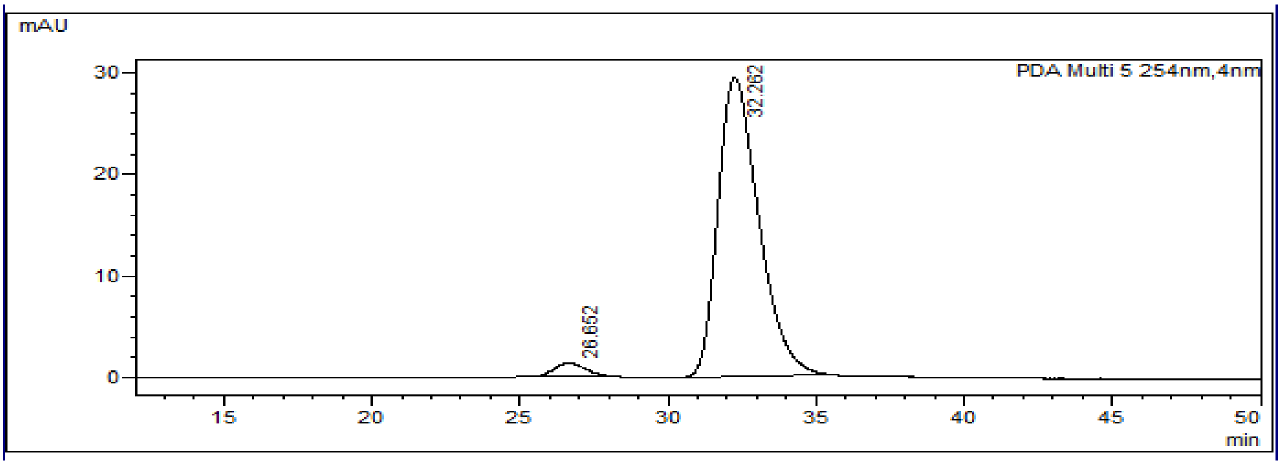

<Peak Table>

PDA Ch5 254nm

Peak#	Ret. Time	Area	Height	Area%	Height%
1	26.652	88050	1241	3.034	4.054
2	32.262	2813929	29375	96.966	95.946
Total		2901980	30616	100.000	100.000

(3S,4S,4aS,8aR)-4-(3-Bromobenzoyl)-3-hydroxy-8a-methyl-3,4,4a,8a-tetrahydro-2*H*-chromen-6(5*H*)-one (10l):

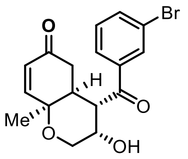

Prepared according to the general procedure as described above in 65% yield (71 mg). It was purified by flash chromatography (50% EtOAc/hexanes; R_f = 0.3) to afford a colourless semi solid; *dr* = >30:1; ^{1}H NMR (400 MHz, $CDCl_3$) δ 7.96 (t, *J* = 1.8 Hz, 1H), 7.72 (tdd, *J* = 2.9, 2.3, 1.0 Hz, 2H), 7.35 (t, *J* = 7.9 Hz, 1H), 6.82 (dd, *J* = 10.4, 2.2 Hz, 1H), 6.15 (dd, *J* = 10.4, 1.3 Hz, 1H), 3.92 (br,s, 1H), 3.88 (dd, *J* = 12.4, 2.2 Hz, 1H), 3.77 (dd, *J* = 12.4, 1.0 Hz, 1H), 3.53 (dd, *J* = 11.8, 2.8 Hz, 1H), 3.10 – 2.98 (m, 1H), 2.75 (dd, *J* = 17.7, 5.2 Hz, 1H), 2.57 (d, *J* = 7.4 Hz, 1H), 2.31 – 2.19 (m, 1H), 1.61 (s, 3H); ^{13}C NMR (101 MHz, $CDCl_3$) δ 198.0, 197.0, 154.2, 138.0, 136.7, 131.4, 131.3, 130.6, 126.8, 123.5, 74.2, 67.5, 65.9, 48.0, 40.1, 36.5, 26.4; HRMS (ESI) calcd for $C_{17}H_{18}BrO_4$ $[M+H]^+$: 365.0383; found: 365.0392; $[\alpha]^{20}_D$ = -35.18° (*c* 0.72, $CHCl_3$); 93:07 *er*; Chiral HPLC analysis of the product: Daicel Chiralpak OD-H 250X4.6 mm 5µ column; hexane/2-propanol = 75/25, detected at 254 nm, Flow rate = 1 mL/min, Retention times: 21.606 min (major), 10.842 min (minor).

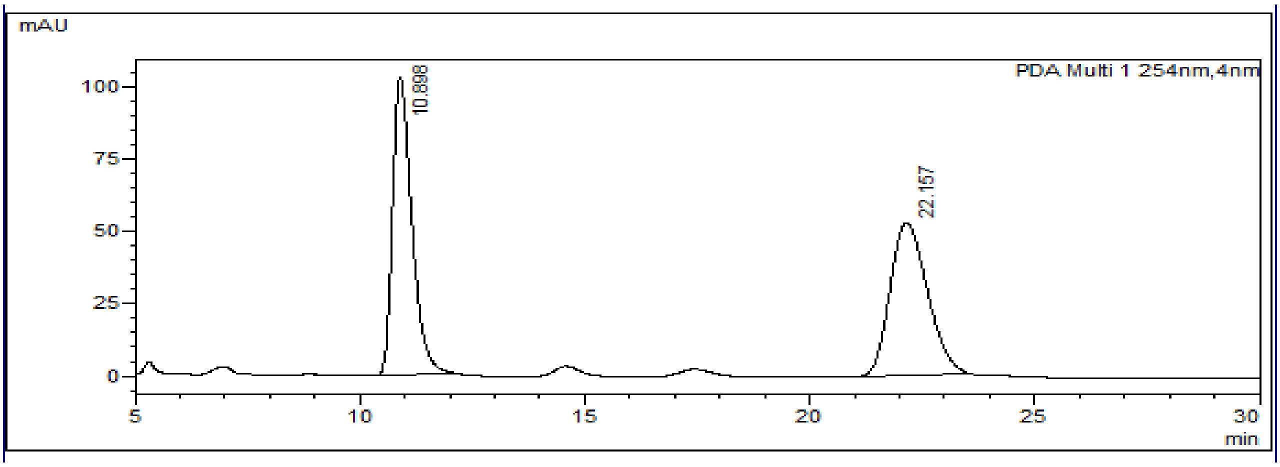

<Peak Table>

PDA Ch1 254nm

Peak#	Ret. Time	Area	Height	Area%	Height%
1	10.898	3082526	102904	50.071	66.003
2	22.157	3073841	53003	49.929	33.997
Total		6156367	155907	100.000	100.000

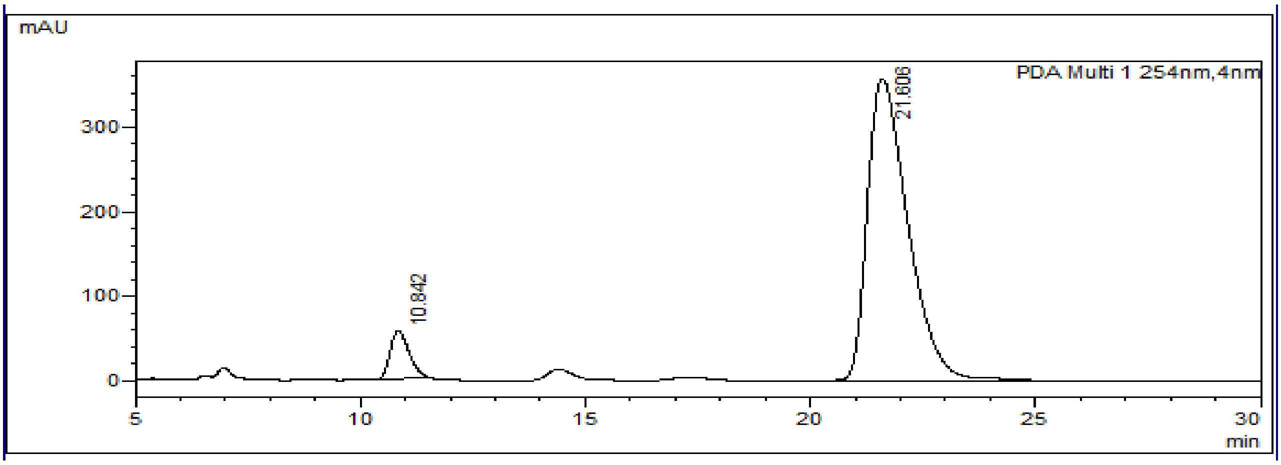

<Peak Table>

PDA Ch1 254nm

Peak#	Ret. Time	Area	Height	Area%	Height%
1	10.842	1610866	56498	6.727	13.681
2	21.606	22335924	356482	93.273	86.319
Total		23946790	412980	100.000	100.000

(4aR,9S,10S,10aS)-10-Benzoyl-9-hydroxy-4a-methoxy-4a,9,10,10a-tetrahydrophenanthren-2(1*H*)-one (10m):

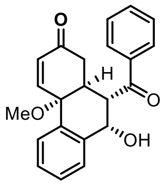

Prepared according to the general procedure as described above in 61% yield (64 mg). It was purified by flash chromatography (40% EtOAc/hexanes; R_f = 0.3) to afford a brown semi solid; *dr* = >30:1; ^{1}H NMR (400 MHz, $CDCl_3$) δ 7.89 (dd, *J* = 8.3, 1.1 Hz, 2H), 7.67 – 7.59 (m, 1H), 7.55 (d, *J* = 7.6 Hz, 1H), 7.52 – 7.41 (m, 5H), 6.95 (dd, *J* = 10.2, 1.0 Hz, 1H), 6.13 (d, *J* = 10.2 Hz, 1H), 4.93 (dd, *J* = 5.2, 3.5 Hz, 1H), 3.92 (dd, *J* = 9.0, 3.4 Hz, 1H), 3.89 – 3.81 (m, 1H), 3.75 (d, *J* = 5.7 Hz, 1H), 3.20 (s, 3H), 2.85 (dd, *J* = 16.6, 4.7 Hz, 1H), 2.26 (dd, *J* = 16.6, 6.5 Hz, 1H); ^{13}C NMR (101 MHz, $CDCl_3$) δ 201.2, 197.5, 150.0, 138.6, 136.7, 136.0, 134.1, 129.6, 129.5, 129.1, 128.5, 127.8, 76.6, 68.7, 51.3, 49.3, 38.9, 33.4; HRMS (ESI) calcd for $C_{22}H_{24}NO_4$ $[M+NH_4^+]^+$: 366.1699; found: 366.1694; $[\alpha]^{20}_D$ = -108.26° (*c* 0.82, $CHCl_3$); 79:21 *er*; Chiral HPLC analysis of the product: Daicel Chiralpak IA 250X4.6 mm 5μ column; hexane/2-propanol = 90/10, detected at 254 nm, Flow rate = 1 mL/min, Retention times: 24.325 min (major), 13.827 min (minor).

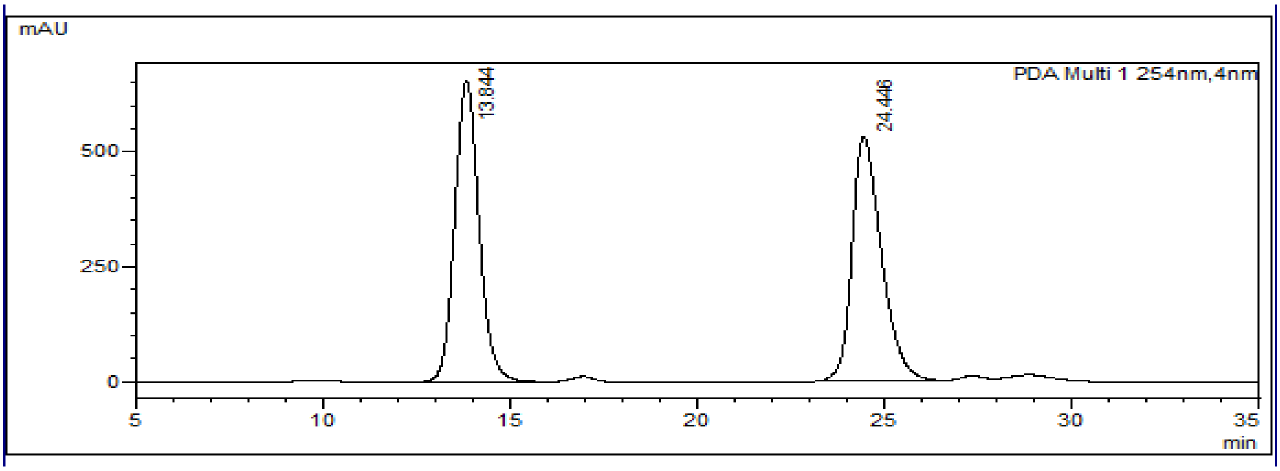

<Peak Table>

PDA Ch1 254nm

Peak#	Ret. Time	Area	Height	Area%	Height%
1	13.844	29490269	654840	50.536	55.291
2	24.446	28864630	529504	49.464	44.709
Total		58354900	1184343	100.000	100.000

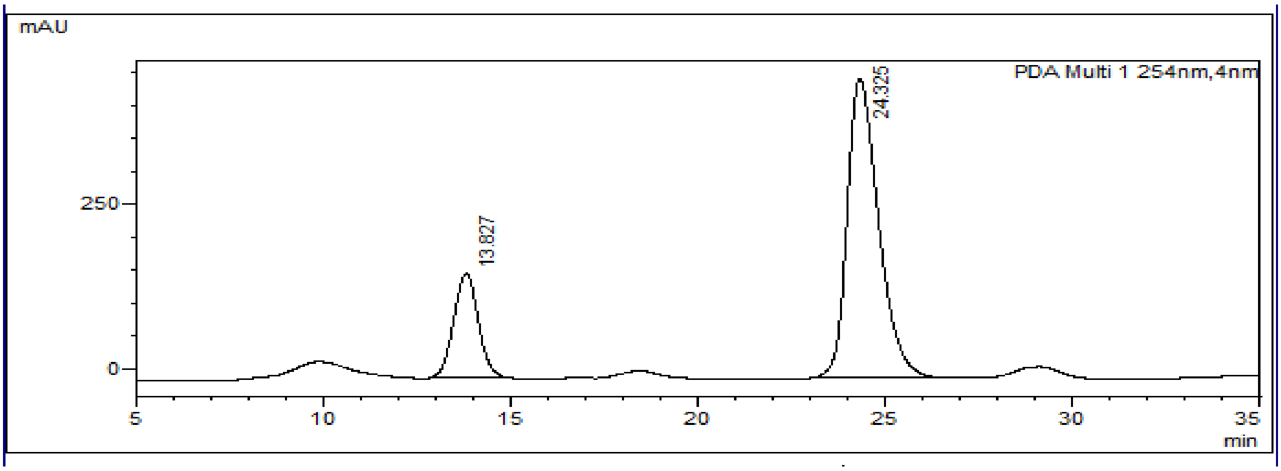

<Peak Table>

PDA Ch1 254nm

Peak#	Ret. Time	Area	Height	Area%	Height%
1	13.827	7253436	158913	21.488	25.889
2	24.325	26501534	454918	78.512	74.111
Total		33754971	613831	100.000	100.000

4-Benzoyl-3-hydroxy-5,7,8a-trimethyl-3,4,4a,8a-tetrahydro-2*H*-chromen-6(5*H*)-one (10n):

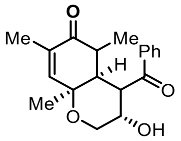

Prepared according to the general procedure as described above in 44 % yield (42 mg). It was purified by flash chromatography (10% EtOAc/hexanes; R_f = 0.3) to afford a light yellow semi solid; HRMS (ESI) calcd for $C_{19}H_{23}O_4$ $[M+H]^+$: 315.1591; found: 315.1584.

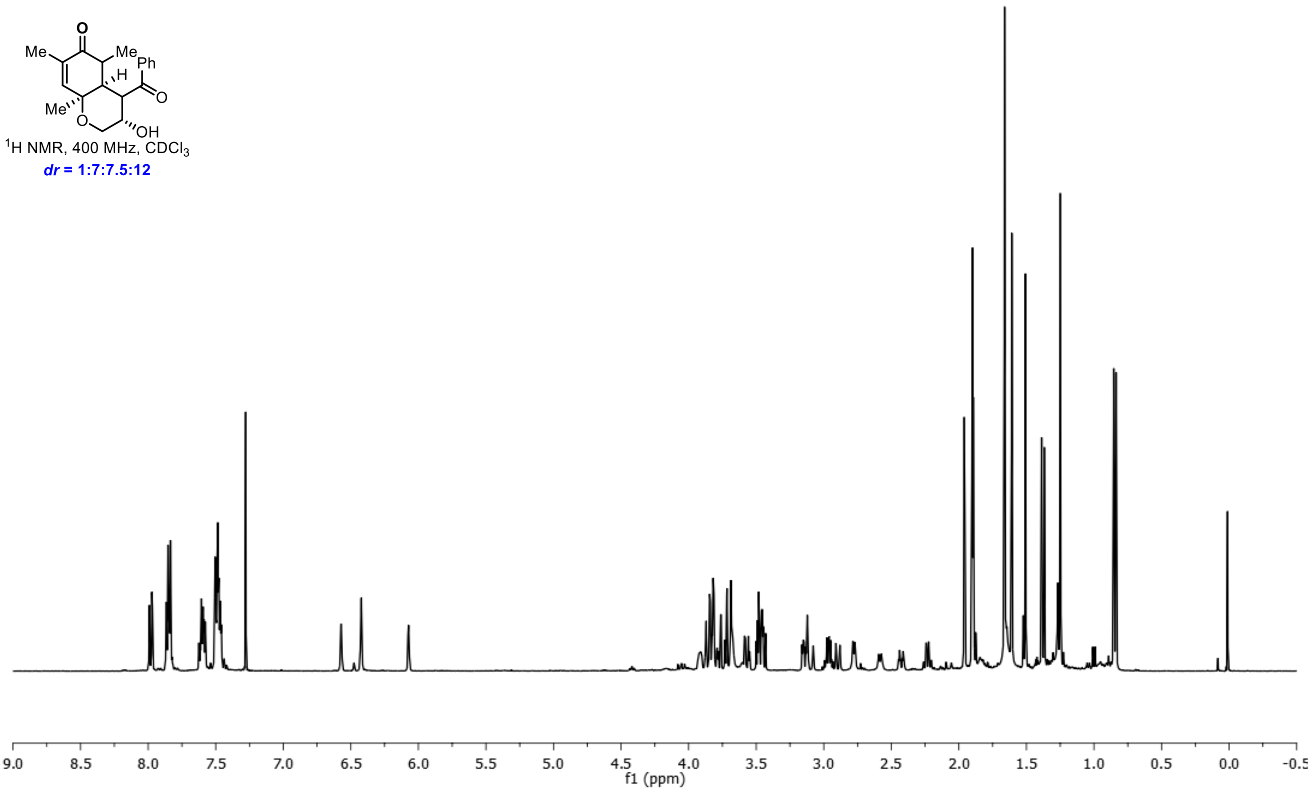

(3*S*,4*S*,4a*S*,8a*R*)-4-Benzoyl-3-hydroxy-8a-methyl-1-tosyl-1,3,4,4a,5,8a-hexahydroquinolin-6(2*H*)-one (10o):

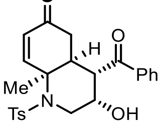

Prepared according to the general procedure as described above in 29% yield (19 mg) with 12:1 *dr*; It was purified by flash chromatography (30% EtOAc/hexanes; R_f = 0.2) to afford a colourless semi solid; ^1H NMR (400 MHz, $CDCl_3$) δ 7.86 – 7.80 (m, 2H), 7.68 – 7.55 (m, 3H), 7.53 – 7.43 (m, 2H), 7.26 – 7.22 (m, 2H), 7.13 (d, J = 10.2 Hz, 1H), 5.77 (dd, J = 10.2, 0.5 Hz, 1H), 4.41 (td, J = 10.8, 5.9

Hz, 1H), 4.26 (dd, J = 13.2, 5.8 Hz, 1H), 3.79 (dd, J = 10.1, 4.3 Hz, 1H), 3.59 (s, 1H), 3.18 (dd, J = 13.1, 11.3 Hz, 1H), 2.67 – 2.57 (m, 1H), 2.40 (s, 3H), 2.26 (dd, J = 16.7, 13.8 Hz, 1H), 1.82 (d, J = 2.3 Hz, 1H), 1.78 (s, 3H); ^{13}C NMR (101 MHz, $CDCl_3$) δ 200.0, 197.0, 151.4, 144.2, 138.3, 135.3, 134.3, 129.9, 129.3, 128.3, 128.2, 127.2, 62.9, 57.6, 51.0, 46.5, 45.0, 35.3, 22.6, 21.7; HRMS (ESI) calcd for $C_{24}H_{26}NO_5S$ $[M+H]^+$: 440.1532; found: 440.1537; $[\alpha]^{20}_D$ = -98.56° (*c* 0.82, $CHCl_3$); 97:03 *er*; Chiral HPLC analysis of the product: Daicel Chiralpak IC 250X4.6 mm 5μ column; hexane/2-propanol = 35/65, detected at 220 nm, Flow rate = 1 mL/min, Retention times: 16.040 min (major), 18.203 min (minor).

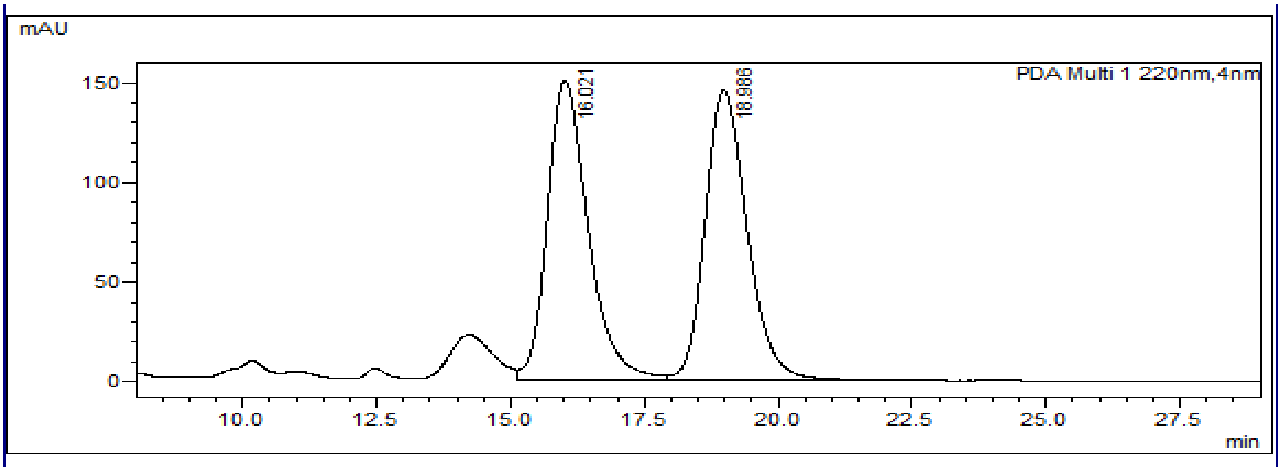

<Peak Table>

PDA Ch1 220nm

Peak#	Ret. Time	Area	Height	Area%	Height%
1	16.021	7981192	150355	50.245	50.851
2	18.986	7903488	145320	49.755	49.149
Total		15884680	295675	100.000	100.000

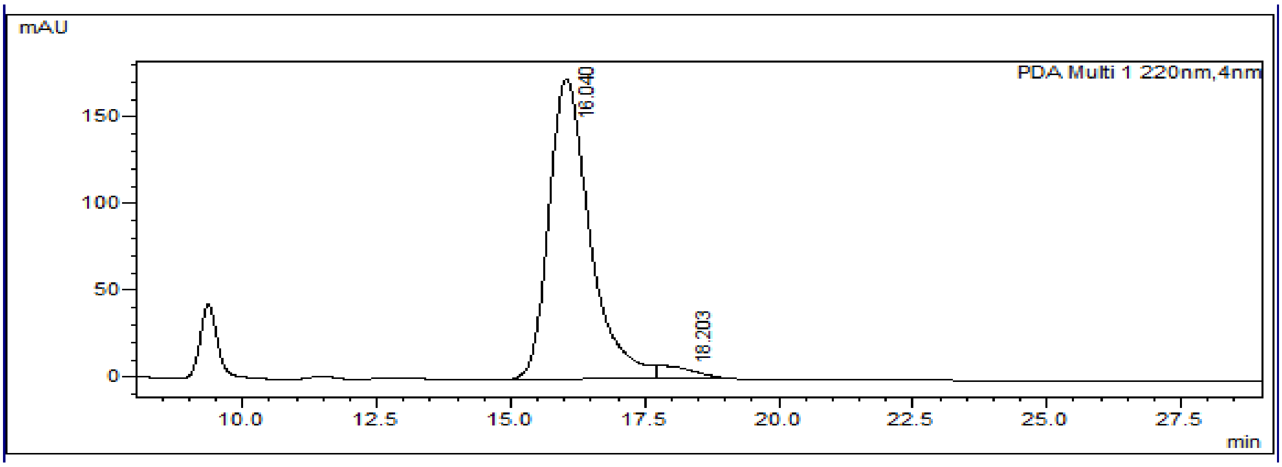

<Peak Table>

PDA Ch1 220nm

Peak#	Ret. Time	Area	Height	Area%	Height%
1	16.040	9204495	172801	96.540	96.665
2	18.203	329895	5962	3.460	3.335
Total		9534391	178764	100.000	100.000

(3*S*,4*S*,4a*S*,8a*R*)-3-Hydroxy-8a-methyl-4-(4-methylbenzoyl)-1-tosyl-1,3,4,4a,5,8a-hexahydroquinolin-6(2*H*)-one (10p):

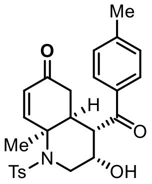

Prepared according to the general procedure as described above in 27% yield (18 mg) with 13:1*dr*. It was purified by flash chromatography (30% EtOAc/hexanes; R_f = 0.3) to afford a colourless semi solid; ^{1}H NMR (400 MHz, $CDCl_3$) δ 7.72 (d, *J* = 8.3 Hz, 2H), 7.61 (d, *J* = 8.3 Hz, 2H), 7.30 – 7.23 (m, 4H), 7.13 (d, *J* = 10.2 Hz, 1H), 5.76 (dd, *J* = 10.2, 0.7 Hz, 1H), 4.40 (td, *J* = 10.7, 5.7 Hz, 1H), 4.25 (dd, *J* = 13.1, 5.8 Hz, 1H), 3.75 (dd, *J* = 10.1, 4.3 Hz, 1H), 3.59 (s, 1H), 3.17 (dd, *J* = 13.1, 11.3 Hz, 1H), 2.47 – 2.41 (m, 1H), 2.40 (s, 6H), 2.28 – 2.22 (m, 1H), 1.81 (dd, *J* = 3.1, 0.8 Hz, 1H), 1.77 (s, 3H);^{13}C NMR (101 MHz, $CDCl_3$) δ 199.6, 197.1, 151.5, 145.4, 144.2, 138.3, 132.8, 130.0, 129.9, 128.5, 128.2, 127.2, 62.9, 57.6, 50.8, 46.5, 45.2, 35.3, 22.6, 21.9, 21.7; HRMS (ESI) calcd for $C_{25}H_{28}NO_5S$ $[M+H]^+$: 454.1688; found: 454.1685; $[\alpha]^{20}_D$ = -122.66° (*c* 1.26, $CHCl_3$); 98:02 *er*; Chiral HPLC analysis of the product: Daicel Chiralpak AD-H 250X4.6 mm 5µ column; hexane/2-propanol = 80/20, detected at 220 nm, Flow rate = 1 mL/min, Retention times: 38.125 min (major), 47.298 min (minor).

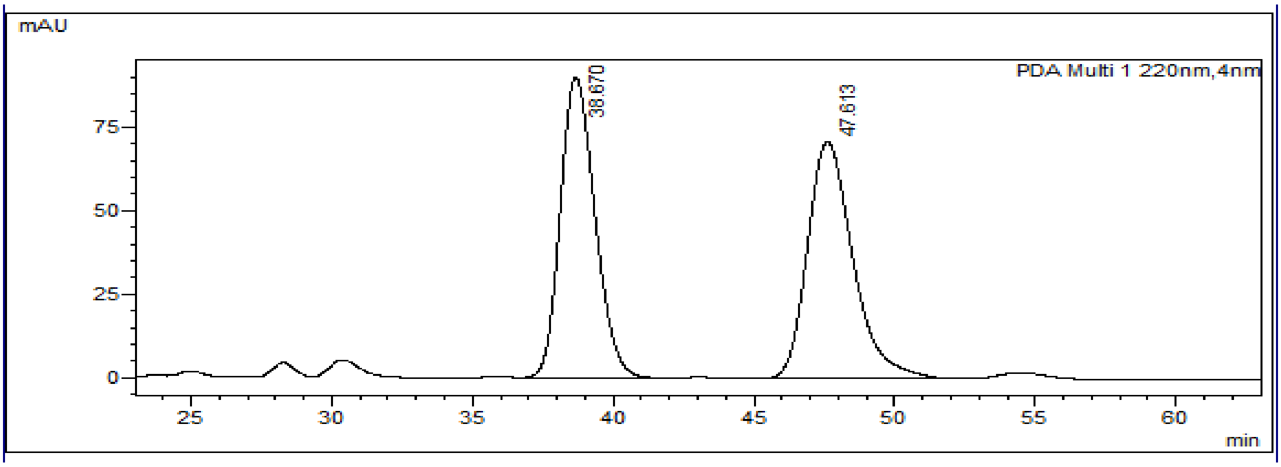

<Peak Table>

PDA Ch1 220nm

Peak#	Ret. Time	Area	Height	Area%	Height%
1	38.670	7701105	89811	49.235	55.900
2	47.613	7940474	70851	50.765	44.100
Total		15641578	160663	100.000	100.000

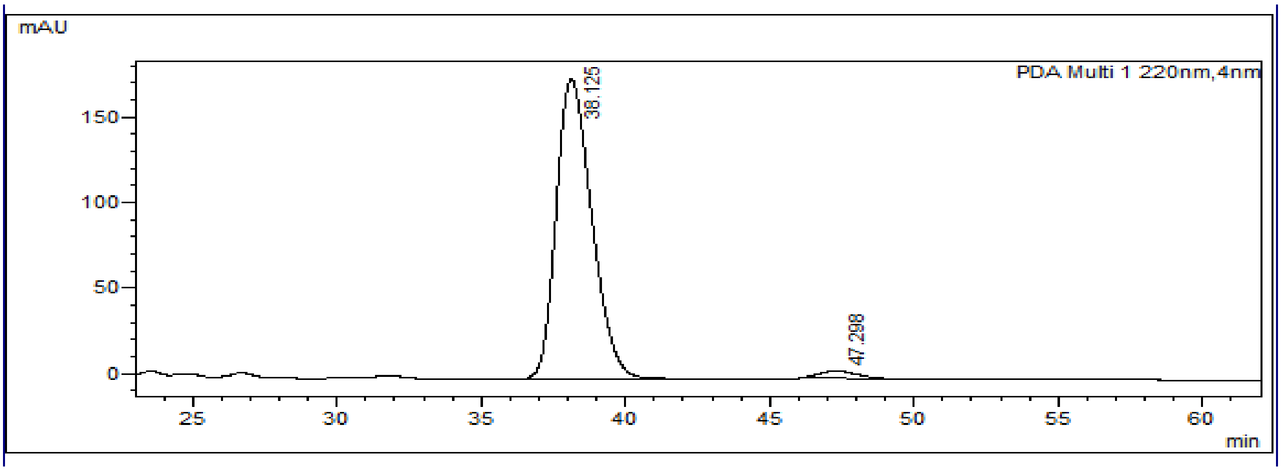

<Peak Table>

PDA Ch1 220nm

Peak#	Ret. Time	Area	Height	Area%	Height%
1	38.125	15043683	175235	97.573	97.683
2	47.298	374202	4157	2.427	2.317
Total		15417884	179393	100.000	100.000

(3*S*,4*S*,4a*S*,8a*R*)-4-(4-Fluorobenzoyl)-3-hydroxy-8a-methyl-1-tosyl-1,3,4,4a,5,8a-hexahydroquinolin-6(2*H*)-one (10q):

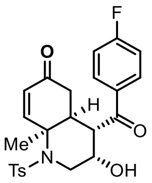

Prepared according to the general procedure as described above in 38% yield (26 mg) with 06:1*dr*; It was purified by flash chromatography (30% EtOAc/hexanes; R_f = 0.2) to afford a colourless semi solid; ^{1}H NMR (400 MHz, $CDCl_3$) δ 7.89 – 7.83 (m, 2H), 7.61 (d, *J* = 8.3 Hz, 2H), 7.29 – 7.22 (m, 2H), 7.21 – 7.08 (m, 3H), 5.78 (d, *J* = 10.1 Hz, 1H), 4.41 (td, *J* = 10.7, 5.7 Hz, 1H), 4.26 (dd, *J* = 13.2, 5.8 Hz, 1H), 3.75 (dd, *J* = 10.1, 4.2 Hz, 1H), 3.17 (dd, *J* = 13.1, 11.3 Hz, 1H), 2.64 (s, 1H), 2.40 (s, 3H), 2.37 (dd, *J* = 9.0, 4.9 Hz, 1H), 2.29 – 2.19 (m, 1H), 1.81 (dd, *J* = 2.7, 0.9 Hz, 1H), 1.77 (s, 3H); ^{13}C NMR (101 MHz, $CDCl_3$) δ 198.2, 196.9, 166.4 (d, J_{CF} = 257.3 Hz), 151.4, 144.2, 138.3, 131.7 (d, J_{CF} = 2.0 Hz), 131.1 (d, J_{CF} = 9.5 Hz), 129.9, 128.3, 127.2, 116.6 (d, J_{CF} = 22.1 Hz), 62.9, 57.5, 50.9, 46.5, 45.1, 35.3, 22.6, 21.7; ^{19}F NMR (376 MHz, $CDCl_3$) δ -102.70 (s, 1F); HRMS (ESI) calcd for $C_{24}H_{25}FNO_5S$ $[M+H]^+$: 458.1437; found: 458.1435; $[\alpha]^{20}_D$ = -89.67° (*c* 0.72, $CHCl_3$); 96:04 *er*; Chiral HPLC analysis of the product: Daicel Chiralpak Eurocel-01 250X4.6 mm 5µ column; hexane/2-propanol = 80/20, detected at 240 nm, Flow rate = 1 mL/min, Retention times: 20.251 min (major), 16.193 min (minor).

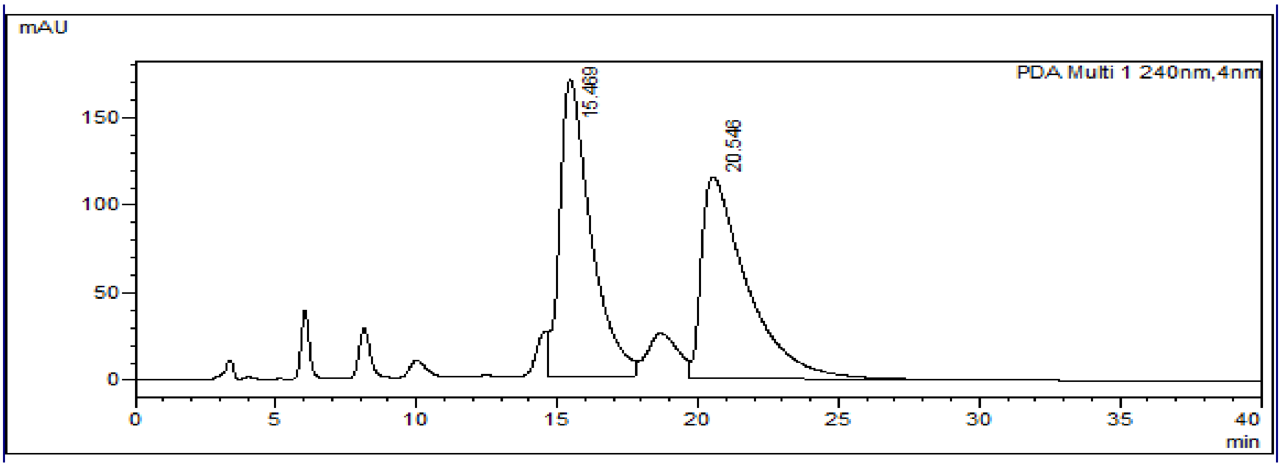

<Peak Table>

PDA Ch1 240nm

Peak#	Ret. Time	Area	Height	Area%	Height%
1	15.469	12988453	169761	50.334	59.662
2	20.546	12816029	114777	49.666	40.338
Total		25804482	284538	100.000	100.000

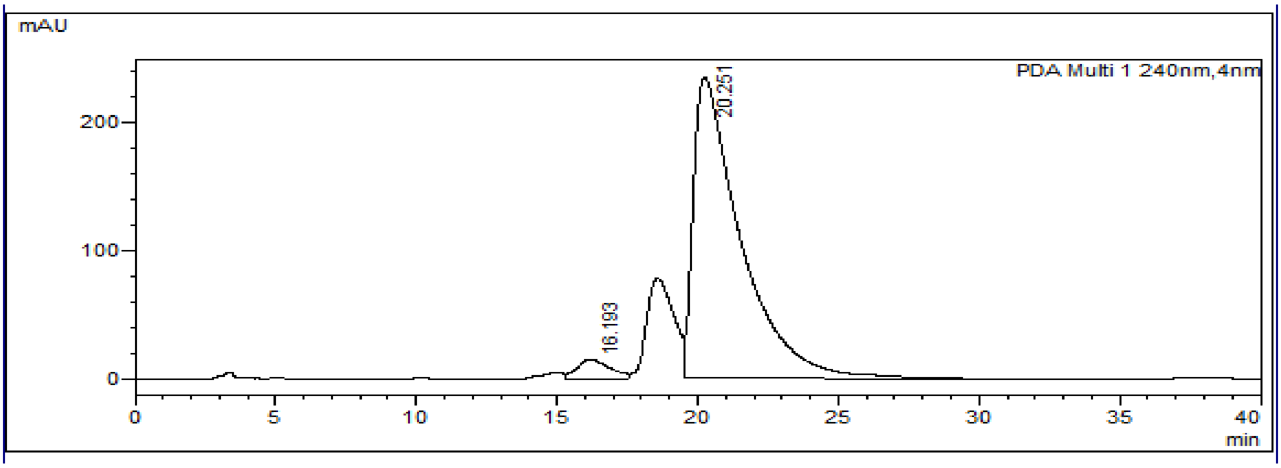

<Peak Table>

PDA Ch1 240nm

Peak#	Ret. Time	Area	Height	Area%	Height%
1	16.193	1185529	14628	4.151	5.886
2	20.251	27375362	233882	95.849	94.114
Total		28560892	248510	100.000	100.000

(3*S*,4*S*,4a*S*,8a*R*)-3-Hydroxy-4-(2-methoxybenzoyl)-8a-methyl-1-tosyl-1,3,4,4a,5,8a-hexahydroquinolin-6(2*H*)-one (10r):

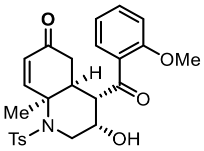

Prepared according to the general procedure as described above in 34% yield (24 mg) with 11:1*dr*; It was purified by flash chromatography (40% EtOAc/hexanes; R_f = 0.3) to afford a colourless semi solid; ^{1}H NMR (400 MHz, $CDCl_3$) δ 7.70 (dd, *J* = 7.8, 1.8 Hz, 1H), 7.61 (d, *J* = 8.3 Hz, 2H), 7.55 – 7.45 (m, 1H), 7.24 (d, *J* = 8.1 Hz, 2H), 7.13 (d, *J* = 10.2 Hz, 1H), 7.06 – 6.99 (m, 1H), 6.94 (d, *J* = 8.3 Hz, 1H), 5.76 (d, *J* = 10.2 Hz, 1H), 4.32 (td, *J* = 10.8, 6.2 Hz, 1H), 4.20 (dd, *J* = 13.0, 5.7 Hz, 1H), 3.94 (dd, *J* = 10.0, 3.9 Hz, 1H), 3.89 (s, 3H), 3.12 (dd, *J* = 13.0, 11.2 Hz, 1H), 2.92 (s, 1H), 2.43 – 2.36 (m, 1H), 2.39 (s, 3H), 2.25 (dd, *J* = 16.5, 13.9 Hz, 1H), 1.77 (dd, *J* = 16.9, 2.7 Hz, 1H), 1.69 (s, 3H); ^{13}C NMR (101 MHz, $CDCl_3$) δ 202.2, 197.5, 158.1, 151.8, 144.0, 138.4, 135.1, 131.5, 129.9, 128.0, 127.2, 125.7, 121.8, 111.7, 63.5, 57.8, 55.7, 55.4, 46.6, 43.7, 35.9, 22.5, 21.7; HRMS (ESI) calcd for $C_{25}H_{28}NO_6S$ $[M+H]^+$: 470.1637; found: 470.1637; $[\alpha]^{20}_D$ = -92.73° (*c* 0.83, $CHCl_3$); 98:02 *er*; Chiral HPLC analysis of the product: Daicel Chiralpak IC 250X4.6 mm 5µ column; hexane/2-propanol = 35/65, detected at 220 nm, Flow rate = 1 mL/min, Retention times: 38.831 min (major), 46.813 min (minor).

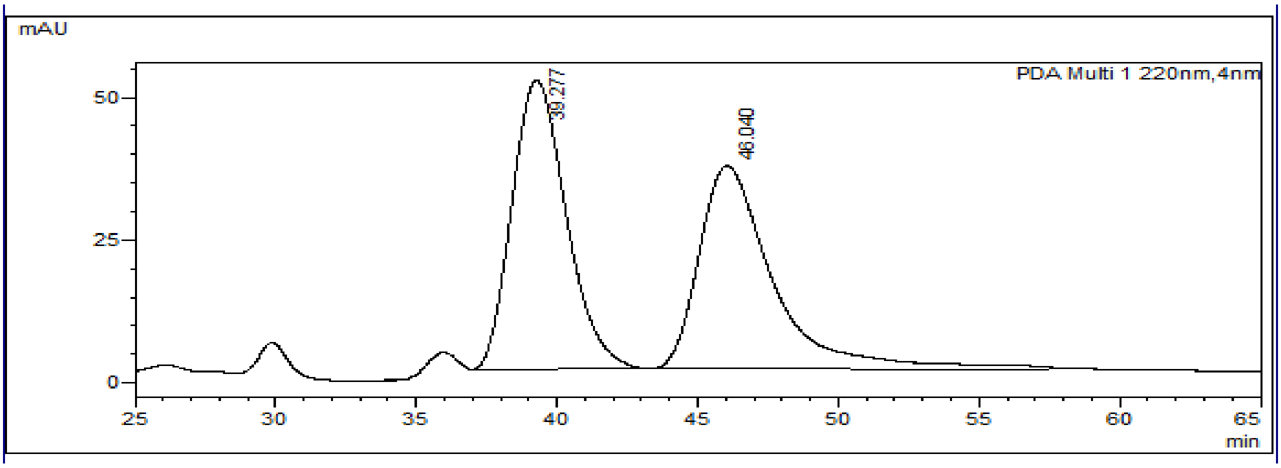

<Peak Table>

PDA Ch1 220nm

Peak#	Ret. Time	Area	Height	Area%	Height%
1	39.277	6802450	50582	50.905	58.709
2	46.040	6560664	35575	49.095	41.291
Total		13363114	86157	100.000	100.000

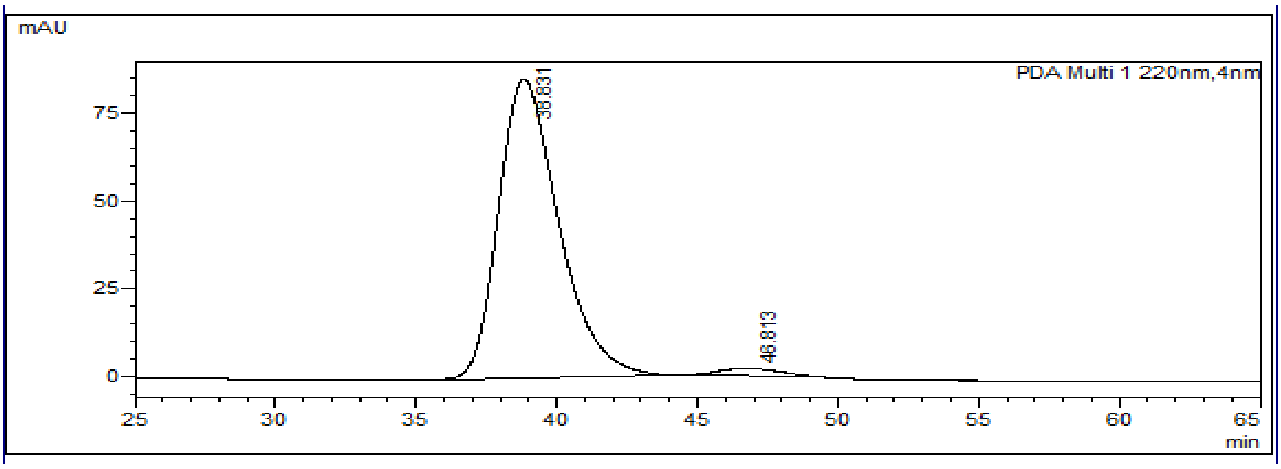

<Peak Table>

PDA Ch1 220nm

Peak#	Ret. Time	Area	Height	Area%	Height%
1	38.831	12667673	85020	97.509	97.513
2	46.813	323570	2168	2.491	2.487
Total		12991244	87188	100.000	100.000

2e. General Procedure for the One-pot borylative cyclization/oxidation without base reaction:

A solution of $Cu(CH_3CN)_4PF_6$ (2.8 mg, 2.5 mol%), (*S*)-SEGPHOS (9 mg, 5 mol%) and $B_2(pin)_2$ (84 mg, 0.33 mmol) in dry THF (2.0 mL) was stirred at room temperature for 15 min and then maintained at -78 °C. A solution of enone **1** (0.3 mmol) in dry THF (1.0 ml) was added via syringe and the resulting mixture was stirred at -78 °C for 3 h then $NaBO_3 \cdot H_2O$ (150 mg, 1.5 mmol) in H_2O (2 mL) was added in one portion and the resulting was mixture stirred vigorously at room temperature for 3 h under open to air. The reaction mixture was quenched with saturated NH_4Cl (10 mL) solution and extracted with EtOAc (3 × 15 mL) and dried over anhydrous Na_2SO_4, filtered, and concentrated in vacuo. The resultant crude product was purified by column chromatography (hexanes/EtOAc).

(3S,4aR,8aS)-8a-Methyl-3-(2-oxo-2-phenylethyl)-2,3,4a,8a-tetrahydrobenzo[*b*][1,4]dioxin-6(5*H*)-one (11a):

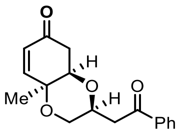

Prepared according to the general procedure as described above in 73% yield (63 mg). It was purified by flash chromatography (30% EtOAc/hexanes; R_f = 0.4) to afford a white solid; mp = 168–170°C; ^1H NMR (400 MHz, $CDCl_3$) δ 7.90 (dd, *J* = 8.3, 1.2 Hz, 2H), 7.65 – 7.51 (m, 1H), 7.49 – 7.37 (m, 2H), 6.65 (dd, *J* = 10.4, 2.8 Hz, 1H), 6.08 (dd, *J* = 10.4, 1.0 Hz, 1H), 4.26 (dtd, *J* = 10.3, 6.2, 2.5 Hz, 1H), 4.00 (q, *J* = 3.0 Hz, 1H), 3.85 (dd, *J* = 11.5, 2.5 Hz, 1H), 3.50 (dd, *J* = 11.5, 10.4 Hz, 1H), 3.23 (dd, *J* = 16.9, 6.0 Hz, 1H), 2.79 (dd, *J* = 16.9, 6.4 Hz, 1H), 2.65 (dd, *J* = 17.3, 3.1 Hz, 1H), 2.57 (ddd, *J* = 17.3, 3.0, 1.1 Hz, 1H), 1.39 (s, 3H); ^{13}C NMR (101 MHz, $CDCl_3$) δ 197.0, 196.0, 152.3, 136.8, 133.5, 130.8, 128.7, 128.3, 78.4, 77.4, 71.5, 67.1, 42.1, 40.6, 24.4; HRMS (ESI) calcd for $C_{17}H_{19}O_4$ $[M+H]^+$: 287.1283; found: 287.1277; $[\alpha]^{20}_D$ = -61.93° (*c* 1.26, $CHCl_3$); 71:29 *er*; Chiral HPLC analysis of the product: Daicel Chiralpak IA 250X4.6 mm 5μ column; hexane/2-propanol = 90/10, detected at 254 nm, Flow rate = 1 mL/min, Retention times: 16.736 min (major), 19.665 min (minor).

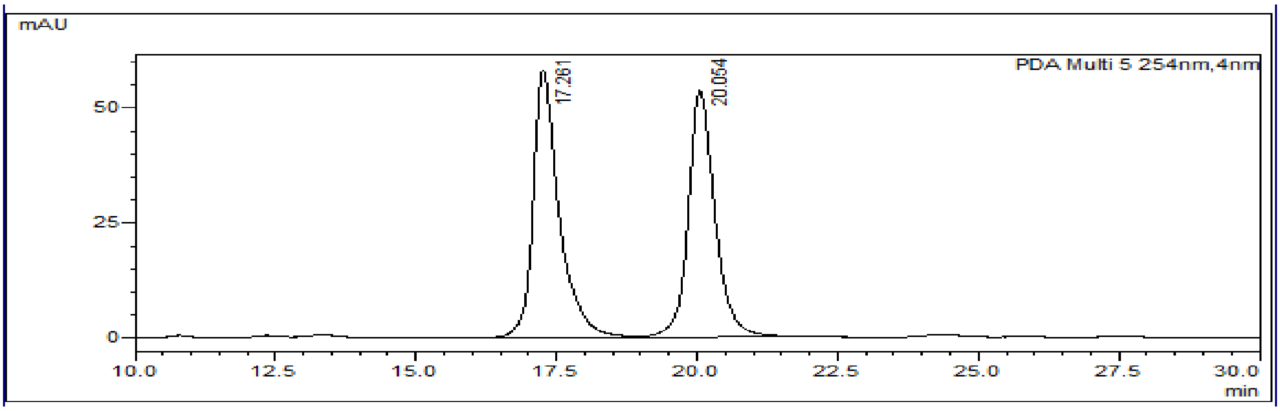

<Peak Table>

PDA Ch5 254nm

Peak#	Ret. Time	Area	Height	Area%	Height%
1	17.261	1869124	57930	51.497	52.047
2	20.054	1760467	53374	48.503	47.953
Total		3629590	111303	100.000	100.000

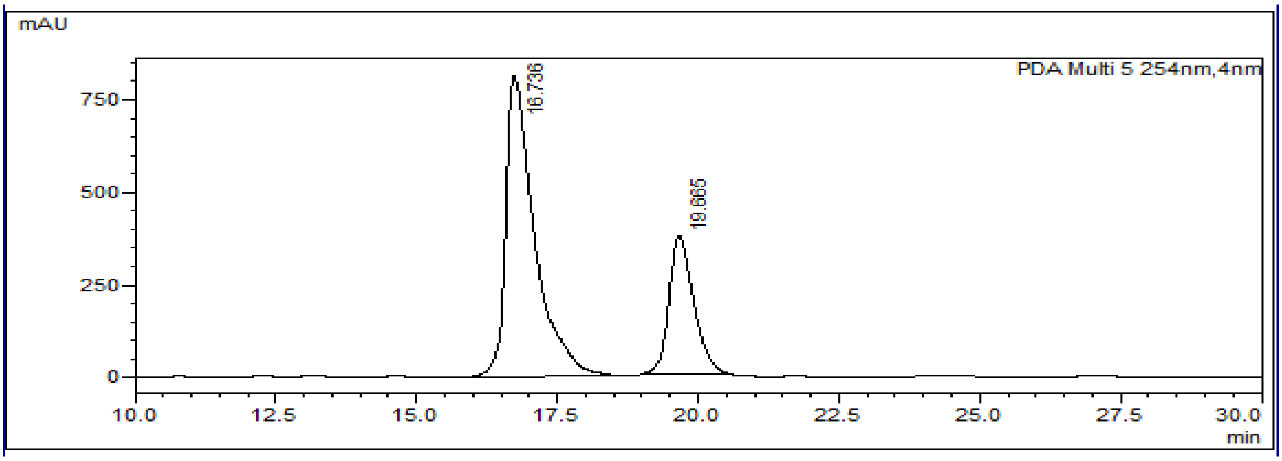

<Peak Table>

PDA Ch5 254nm

Peak#	Ret. Time	Area	Height	Area%	Height%
1	16.736	28952368	812567	71.442	68.538
2	19.665	11573604	373013	28.558	31.462
Total		40525971	1185579	100.000	100.000

(3S,4aR,8aS)-8a-Ethyl-3-(2-oxo-2-phenylethyl)-2,3,4a,8a-tetrahydrobenzo[*b*][1,4]dioxin-6(5*H*)-one (11b):

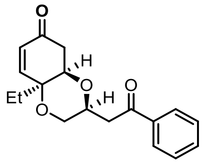

Prepared according to the general procedure as described above in 72% yield (65 mg). It was purified by flash chromatography (30% EtOAc/hexanes; R_f = 0.4) to afford a white semi solid; ^{1}H NMR (400 MHz, $CDCl_3$) δ 7.90 (dd, *J* = 8.3, 1.2 Hz, 2H), 7.69 – 7.51 (m, 1H), 7.49 – 7.40 (m, 2H), 6.70 (dd, *J* = 10.5, 2.8 Hz, 1H), 6.11 (dd, *J* = 10.5, 1.1 Hz, 1H), 4.29 – 4.17 (m, 1H), 4.06 (q, *J* = 3.0 Hz, 1H), 3.86 (dd, *J* = 11.5, 2.5 Hz, 1H), 3.49 (dd, *J* = 11.5, 10.4 Hz, 1H), 3.23 (dd, *J* = 16.9, 6.0 Hz, 1H), 2.79 (dd, *J* = 16.9, 6.4 Hz, 1H), 2.65 (dd, *J* = 17.5, 3.2 Hz, 1H), 2.54 (ddd, *J* = 17.5, 3.0, 1.1 Hz, 1H), 1.80 – 1.66 (m, 2H), 1.04 (t, *J* = 7.5 Hz, 3H); ^{13}C NMR (126 MHz, $CDCl_3$) δ 197.1, 196.1, 152.3, 136.9, 133.5, 131.1, 128.7, 128.3, 77.0, 73.4, 71.5, 67.0, 41.9, 40.6, 31.4, 7.5; HRMS (ESI) calcd for $C_{18}H_{24}NO_4$ $[M+NH_4^+]^+$: 318.1699; found: 318.1692; $[\alpha]^{20}_D$ = -73.12° (*c* 0.68, $CHCl_3$); 72:28 *er*; Chiral HPLC analysis of the product: Daicel Chiralpak Eurocel-01 250X4.6 mm 5µ column; hexane/2-

propanol = 85/15, detected at 254 nm, Flow rate = 1 mL/min, Retention times: 15.989 min (major), 13.288 min (minor).

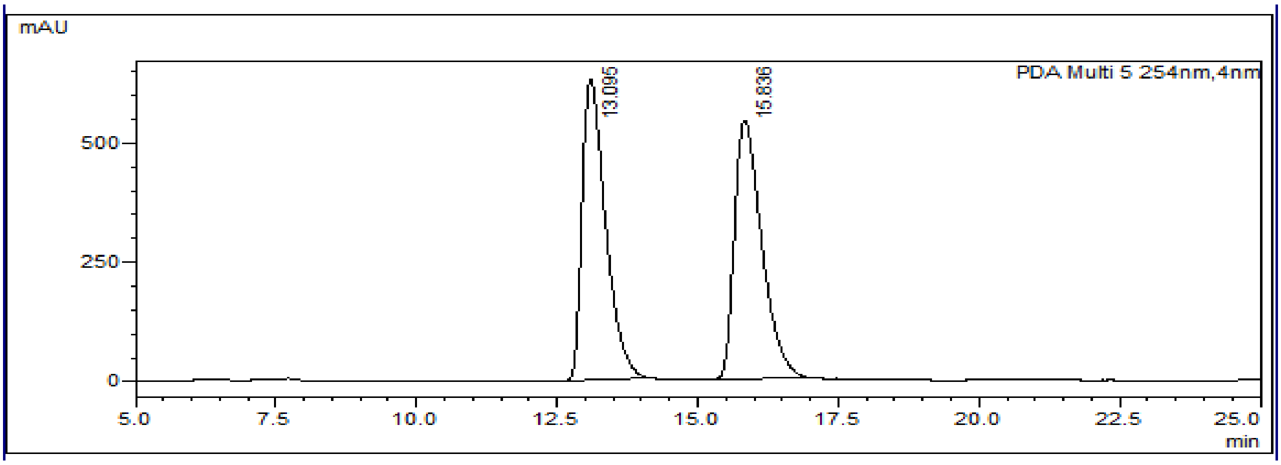

<Peak Table>

PDA Ch5 254nm

Peak#	Ret. Time	Area	Height	Area%	Height%
1	13.095	18432389	631503	49.880	53.799
2	15.836	18520718	542325	50.120	46.201
Total		36953106	1173827	100.000	100.000

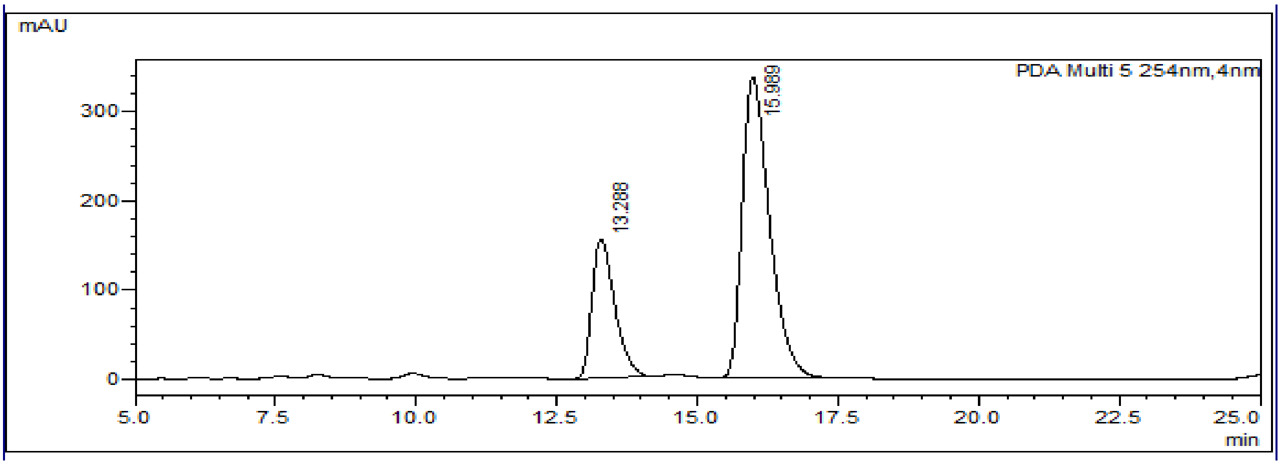

<Peak Table>

PDA Ch5 254nm

Peak#	Ret. Time	Area	Height	Area%	Height%
1	13.288	4363263	155496	27.689	31.633
2	15.989	11395014	336067	72.311	68.367
Total		15758277	491563	100.000	100.000

(3S,4aR,8aS)-3-(2-(4-Bromophenyl)-2-oxoethyl)-8a-methyl-2,3,4a,8a-tetrahydrobenzo[*b*][1,4]dioxin-6(5*H*)-one (11c):

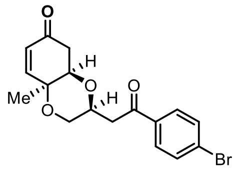

Prepared according to the general procedure as described above in 51% yield (56 mg). It was purified by flash chromatography (30% EtOAc/hexanes; R_f = 0.4) to afford a white semi solid; ^{1}H NMR (500 MHz, $CDCl_3$) δ 7.80 – 7.72 (m, 2H), 7.62 – 7.54 (m, 2H), 6.65 (dd, *J* = 10.4, 2.8 Hz, 1H), 6.08 (dd, *J* = 10.4, 1.2 Hz, 1H), 4.31 – 4.19 (m, 1H), 4.00 (q, *J* = 3.0 Hz, 1H), 3.83 (dd, *J* = 11.6, 2.6 Hz, 1H), 3.50 (dd, *J* = 11.5, 10.4 Hz, 1H), 3.18 (dd, *J* = 16.8, 6.2 Hz, 1H), 2.74 (dd, *J* = 16.8, 6.2 Hz, 1H), 2.64 (dd, *J* = 17.3, 3.1 Hz, 1H), 2.56 (ddd, *J* = 17.3, 3.0, 1.2 Hz, 1H), 1.39 (s, 3H); ^{13}C NMR (101 MHz, $CDCl_3$) δ 196.1, 195.9, 152.2, 135.6, 132.1, 130.8, 129.8, 128.8, 78.5, 71.6, 71.5, 67.1, 42.1, 40.6, 24.4; HRMS (ESI) calcd for $C_{17}H_{21}NO_4Br$ $[M+NH_4^+]^+$: 382.0649; found: 382.0647; $[\alpha]^{20}_D$ = 102.13° (*c* 0.25, $CHCl_3$); 81:19 *er*; Chiral HPLC analysis of the product: Daicel Chiralpak IC 250X4.6 mm 5µ column; hexane/2-propanol = 75/25, detected at 254 nm, Flow rate = 1 mL/min, Retention times: 18.878 min (major), 20.536 min (minor).

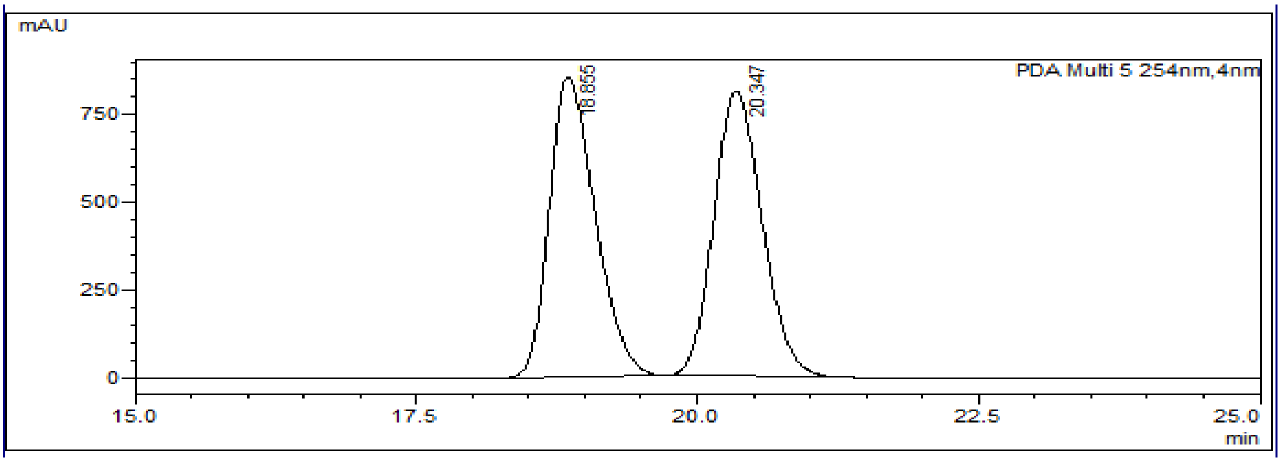

<Peak Table>

PDA Ch5 254nm

Peak#	Ret. Time	Area	Height	Area%	Height%
1	18.855	24324856	854127	50.005	51.264
2	20.347	24319576	812016	49.995	48.736
Total		48644432	1666144	100.000	100.000

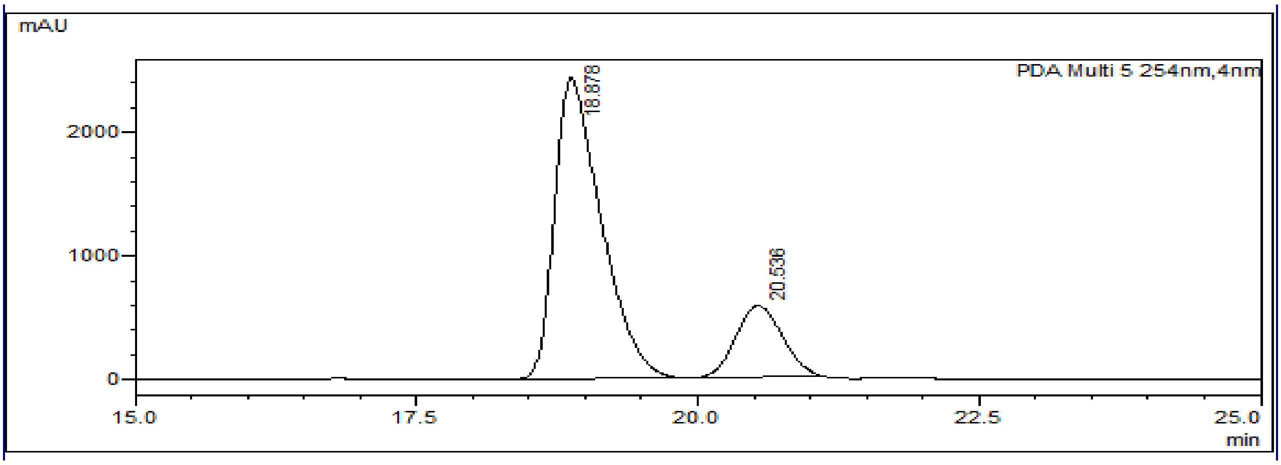

<Peak Table>

PDA Ch5 254nm

Peak#	Ret. Time	Area	Height	Area%	Height%
1	18.878	70364545	2436351	80.881	80.831
2	20.536	16632586	577779	19.119	19.169
Total		86997132	3014130	100.000	100.000

(4aS,6R,10bR)-10b-Methoxy-6-(2-oxo-2-phenylethyl)-4,4a,6,10b-tetrahydro-3*H*-benzo[*c*]chromen-3-one (11e):

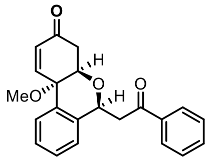

Prepared according to the general procedure as described above in 60% yield (63 mg). It was purified by flash chromatography (30% EtOAc/hexanes; R_f = 0.3) to afford a brown semi solid; ^{1}H NMR (400 MHz, $CDCl_3$) δ 7.88 (dd, *J* = 8.4, 1.2 Hz, 2H), 7.62 – 7.56 (m, 1H), 7.52 (dd, *J* = 7.7, 1.4 Hz, 1H), 7.50 – 7.44 (m, 2H), 7.34 (dd, *J* = 11.3, 4.0 Hz, 1H), 7.29 (td, *J* = 7.4, 1.6 Hz, 1H), 7.14 (d, *J* = 7.4 Hz, 1H), 6.89 (dd, *J* = 10.1, 1.6 Hz, 1H), 6.03 (dd, *J* = 10.1, 0.7 Hz, 1H), 3.96 – 3.78 (m, 1H), 3.35 (dtd, *J* = 6.4, 5.0, 1.5 Hz, 1H), 3.15 (dd, *J* = 16.4, 10.8 Hz, 1H), 3.10 (s, 3H), 2.93 (ddd, *J* = 21.5, 16.6, 4.9 Hz, 2H), 2.35 (ddd, *J* = 16.7, 5.2, 0.6 Hz, 1H); ^{13}C NMR (101 MHz, $CDCl_3$) δ 201.4, 198.2, 151.1, 137.,0 136.5, 135.7, 133.8, 129.0, 128.8, 128.8, 128.5, 128.3, 127.7, 127.5, 76.4, 50.9, 43.8, 38.2, 37.7, 33.1; HRMS (ESI) calcd for $C_{22}H_{20}O_4Na$ $[M+Na]^+$: 371.1254; found: 371.1262; $[\alpha]^{20}_D$ = -98.44° (*c* 1.78, $CHCl_3$); 70:30 *er*; Chiral HPLC analysis of the product: Daicel Chiralpak IA 250X4.6 mm 5µ column; hexane/2-propanol = 95/05, detected at 254 nm, Flow rate = 1 mL/min, Retention times: 19.619 min (major), 13.950 min (minor).

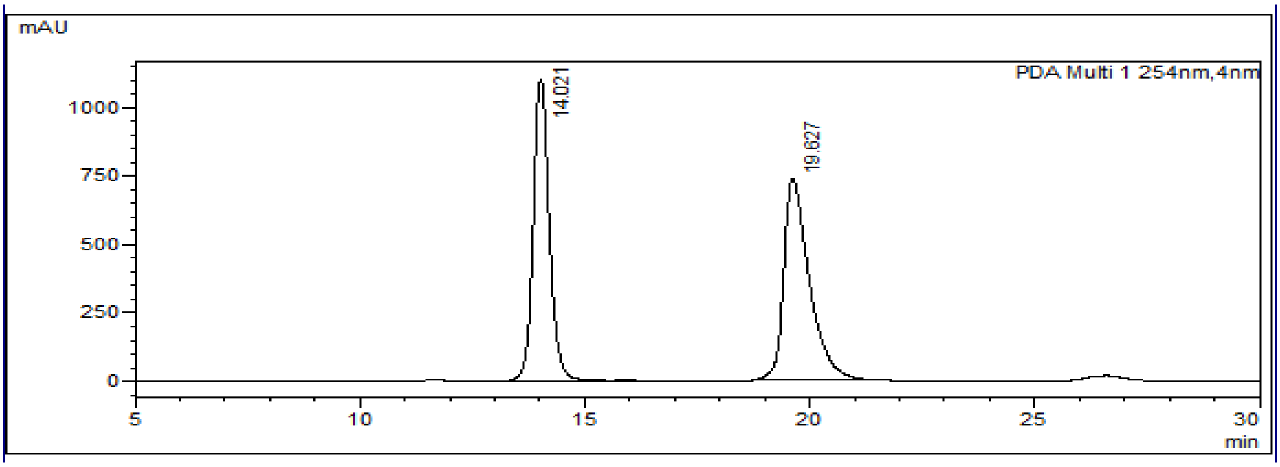

<Peak Table>

PDA Ch1 254nm

Peak#	Ret. Time	Area	Height	Area%	Height%
1	14.021	27694491	1102160	48.573	59.908
2	19.627	29322210	737600	51.427	40.092
Total		57016701	1839761	100.000	100.000

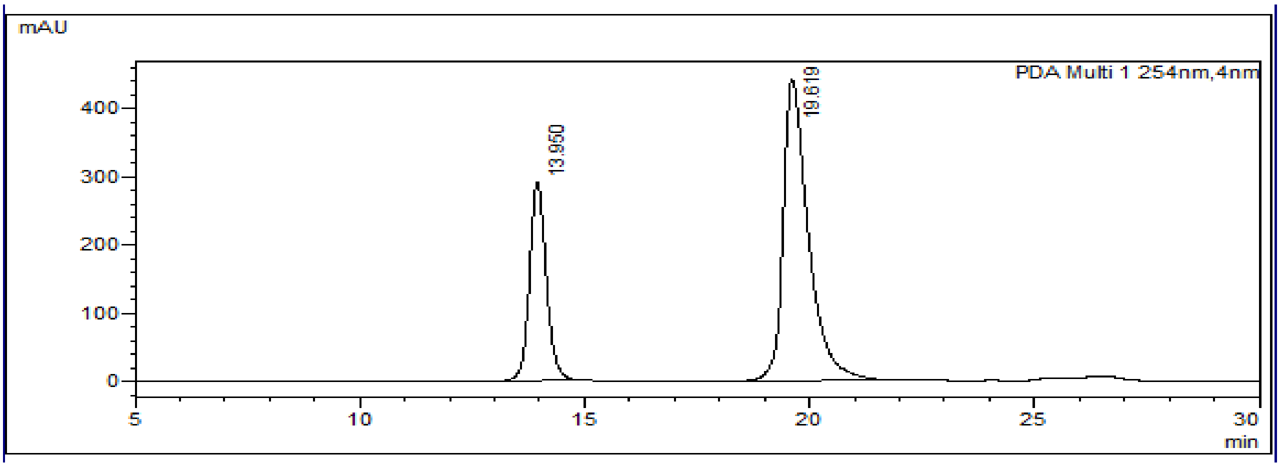

<Peak Table>

PDA Ch1 254nm

Peak#	Ret. Time	Area	Height	Area%	Height%
1	13.950	7521911	291332	30.230	39.729
2	19.619	17360120	441967	69.770	60.271
Total		24882031	733300	100.000	100.000

HPLC reports of compounds 10f, 10g, 10h and 10K in Table 5:

Compound 10f :

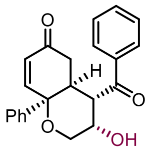

Chiral HPLC analysis of the product **10f**: 76:24 *er*; Daicel Chiralpak IA 250X4.6 mm 5µ column; hexane/2-propanol = 80/20, detected at 254 nm, Flow rate = 1 mL/min, Retention times: 14.981 min (major), 12.335 min (minor).

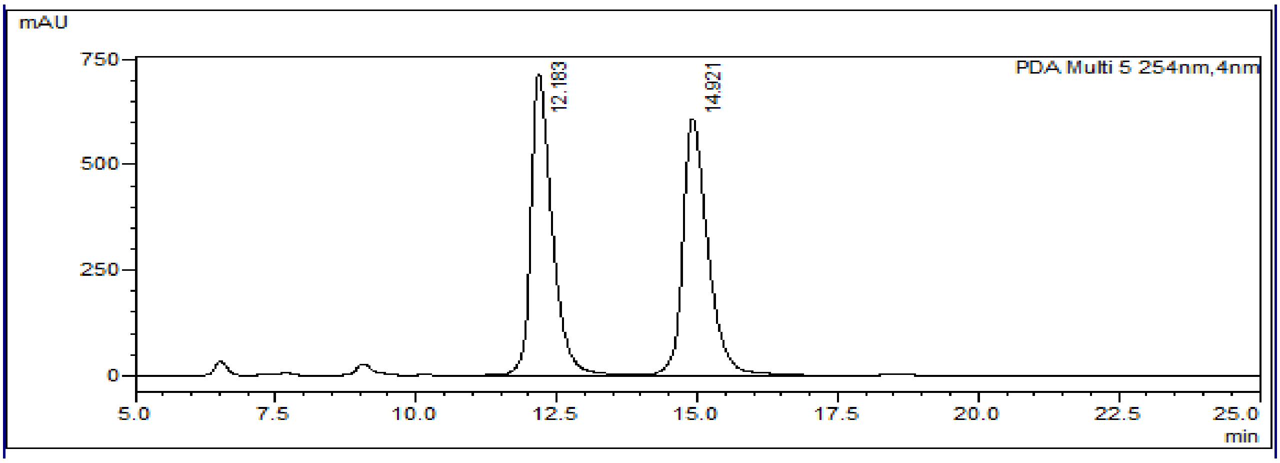

<Peak Table>

PDA Ch5 254nm

Peak#	Ret. Time	Area	Height	Area%	Height%
1	12.183	18939316	715431	49.897	53.969
2	14.921	19017703	610191	50.103	46.031
Total		37957018	1325622	100.000	100.000

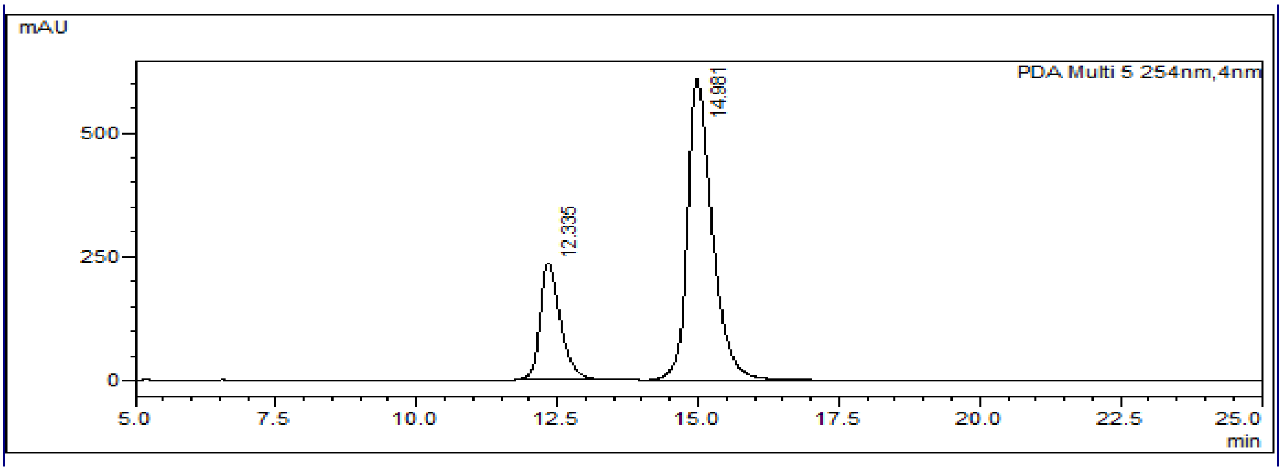

<Peak Table>

PDA Ch5 254nm

Peak#	Ret. Time	Area	Height	Area%	Height%
1	12.335	5820070	234665	23.774	27.867
2	14.981	18660726	607434	76.226	72.133
Total		24480796	842098	100.000	100.000

Compound 10g :

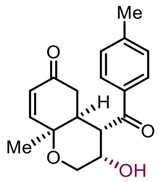

Chiral HPLC analysis of the product **10g**: 72:28 *er*; Daicel Chiralpak OD-H 250X4.6 mm 5μ column; hexane/2-propanol = 80/20, detected at 254 nm, Flow rate = 1 mL/min, Retention times: 29.891 min (major), 15.320 min (minor).

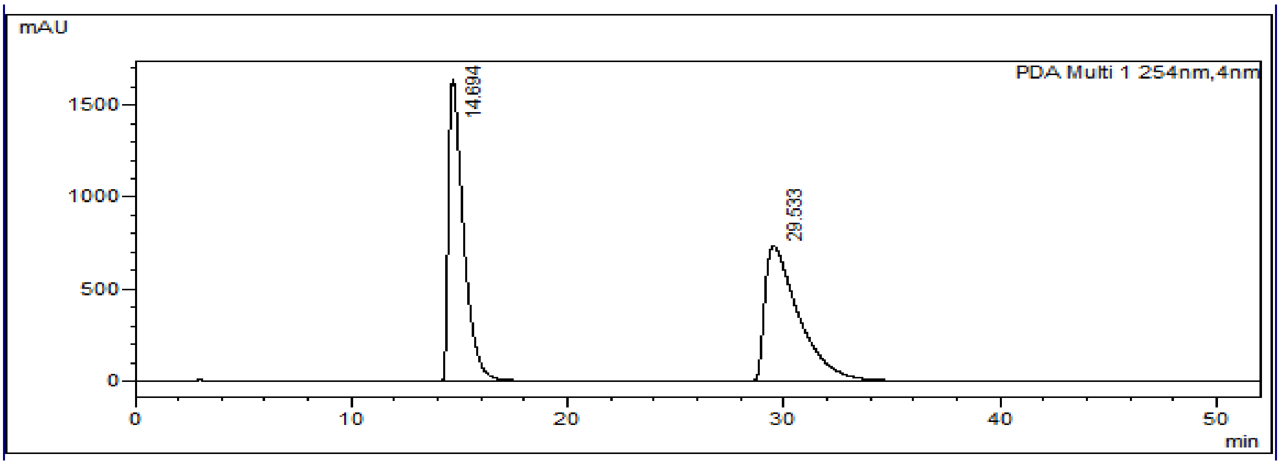

<Peak Table>

PDA Ch1 254nm

Peak#	Ret. Time	Area	Height	Area%	Height%
1	14.694	78997003	1640955	49.964	69.096
2	29.533	79111382	733939	50.036	30.904
Total		158108385	2374894	100.000	100.000

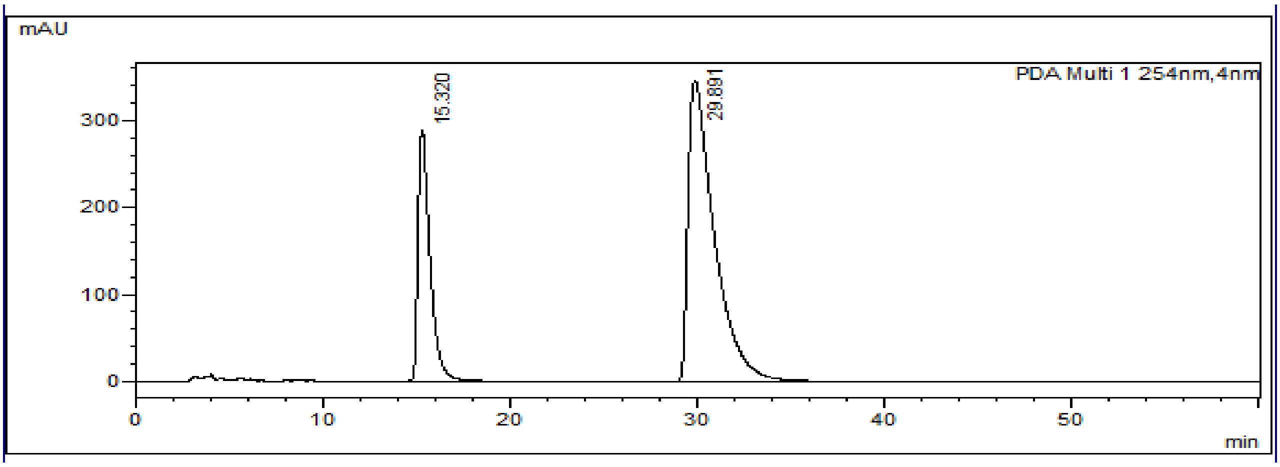

<Peak Table>

PDA Ch1 254nm

Peak#	Ret. Time	Area	Height	Area%	Height%
1	15.320	12914642	288566	27.651	45.453
2	29.891	33790811	346307	72.349	54.547
Total		46705454	634873	100.000	100.000

Compound 10h :

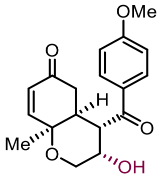

Chiral HPLC analysis of the product **10h**: 77:23 *er*; Daicel Chiralpak Eurocel-01 250X4.6 mm 5µ column; hexane/2-propanol = 75/25, detected at 254 nm, Flow rate = 1 mL/min, Retention times: 30.553 min (major), 17.822 min (minor).

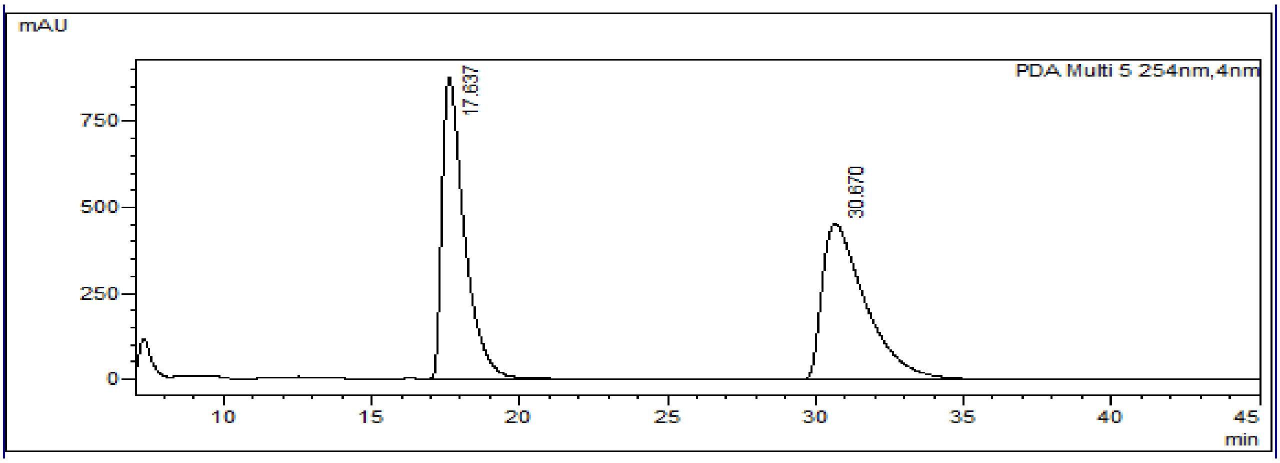

<Peak Table>

PDA Ch5 254nm

Peak#	Ret. Time	Area	Height	Area%	Height%
1	17.637	46140435	876712	50.224	66.018
2	30.670	45729568	451269	49.776	33.982
Total		91870002	1327981	100.000	100.000

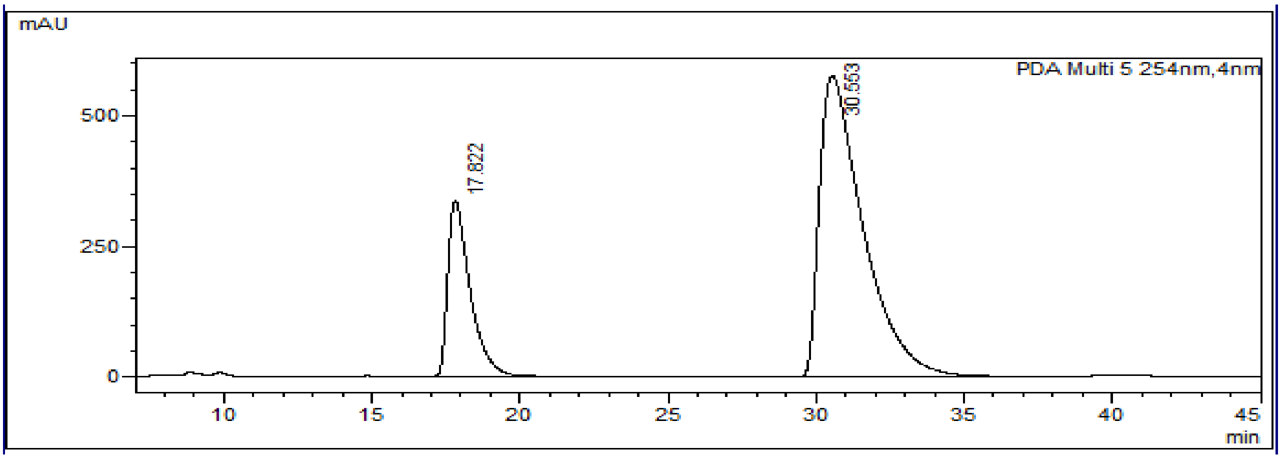

<Peak Table>

PDA Ch5 254nm

Peak#	Ret. Time	Area	Height	Area%	Height%
1	17.822	17561239	335072	22.901	36.796
2	30.553	59120515	575550	77.099	63.204
Total		76681754	910622	100.000	100.000

Compound 10k:

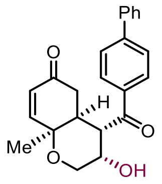

71:29 *er*; Chiral HPLC analysis of the product: Daicel Chiralpak OD-H 250X4.6 mm 5μ column; hexane/2-propanol = 75/25, detected at 254 nm, Flow rate = 1 mL/min, Retention times: 32.399 min (major), 26.811 min (minor).

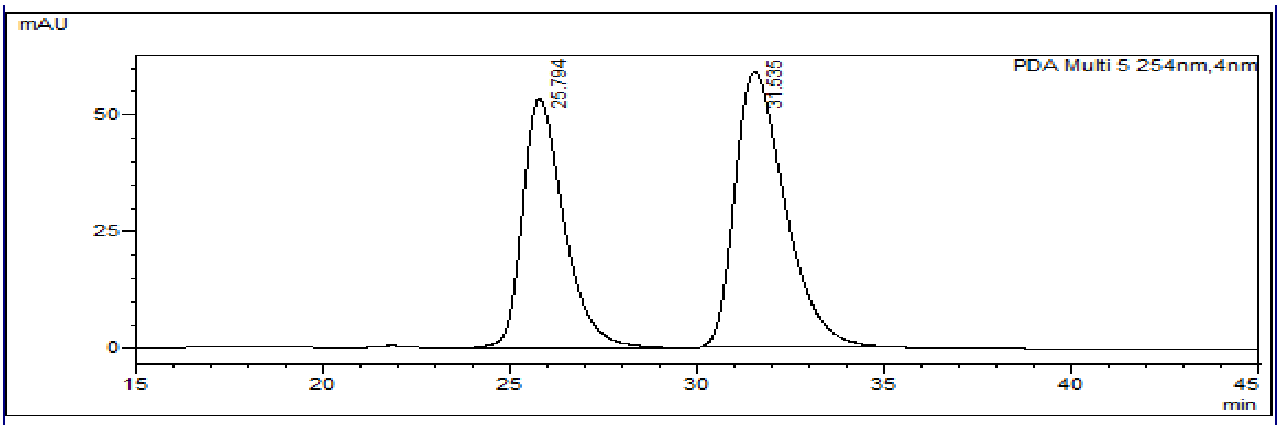

<Peak Table>

PDA Ch5 254nm

Peak#	Ret. Time	Area	Height	Area%	Height%
1	25.794	4110576	53352	43.056	47.565
2	31.535	5436473	58814	56.944	52.435
Total		9547049	112166	100.000	100.000

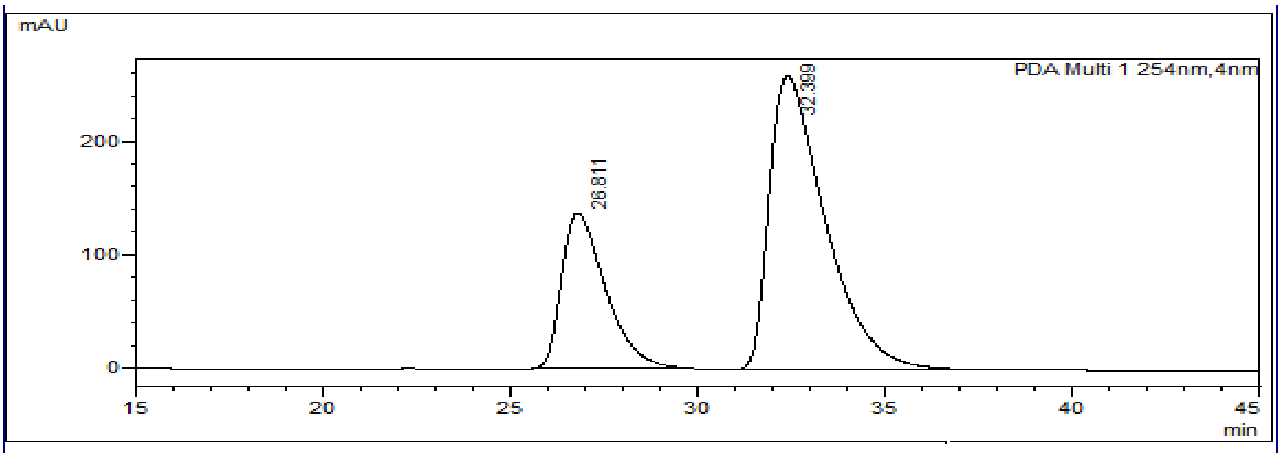

<Peak Table>

PDA Ch1 254nm

Peak#	Ret. Time	Area	Height	Area%	Height%
1	26.811	11134120	136893	29.202	34.578
2	32.399	26994400	259003	70.798	65.422
Total		38128519	395896	100.000	100.000

2f. Gram-scale synthesis of 2a:

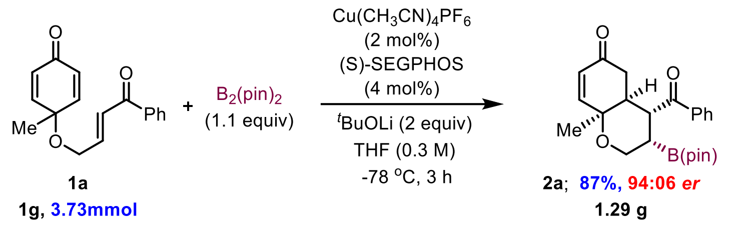

A solution of $Cu(CH_3CN)_4PF_6$ (28 mg, 2.0 mol%), (*S*)-SEGPHOS (91 mg, 5 mol%), $B_2(pin)_2$ (1.042 gm, 4.10 mmol) and ^{t}BuOLi (6.72 mL, 2 equiv, 1.0 M in THF) in dry THF (10 mL) was stirred at room temperature for 30 min and then maintained at -78 °C. A solution of enone **1a** (1 gm, 3.73 mmol) in dry THF (3.0 mL) was added via syringe and the resulting mixture was stirred at -78 °C for 3 h. The reaction mixture was quenched with saturated NH_4Cl (20 mL) solution and extracted with EtOAc (3 × 30 mL) and dried over anhydrous Na_2SO_4, filtered, and concentrated in *vacuo*. The resultant crude product was purified by column chromatography over silica gel (hexanes/EtOAc:70/30) to afford the desired product **2a** in 87% yield (1.29 g) as a brown semi solid with 94 :06 *er*. Chiral HPLC analysis of the product: Daicel Chiralpak IA 250x4.6 mm 5µ column; hexane/2-propanol = 80/20, detected at 240 nm, Flow rate = 1 mL/min, Retention times: 5.291 min (major), 4.451 min (minor).

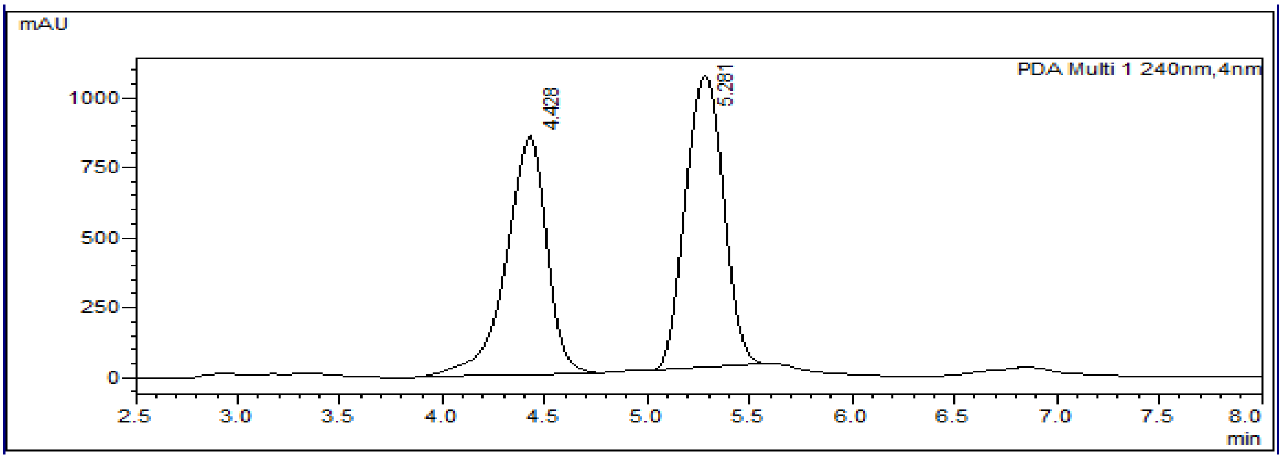

<Peak Table>

PDA Ch1 240nm

Peak#	Ret. Time	Area	Height	Area%	Height%
1	4.428	12192756	855463	47.746	45.130
2	5.281	13343731	1040081	52.254	54.870
Total		25536488	1895545	100.000	100.000

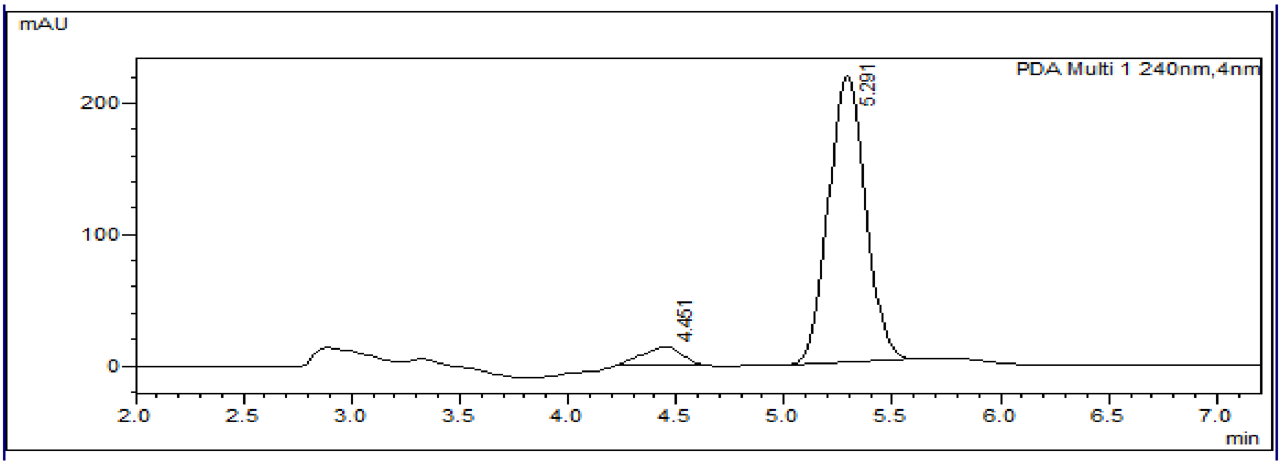

<Peak Table>

PDA Ch1 240nm

Peak#	Ret. Time	Area	Height	Area%	Height%
1	4.451	168900	13829	6.060	5.970
2	5.291	2618434	217811	93.940	94.030
Total		2787334	231640	100.000	100.000

2g. Synthetic utility:

(4aS,8aS)-4-Benzoyl-8a-methyl-4a,8a-dihydro-2*H*-chromen-6(5*H*)-one (12):

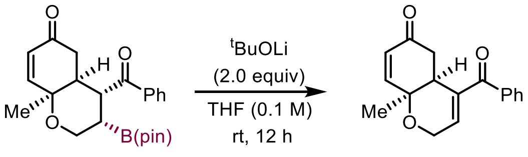

To a stirred solution of bicyclic product **2a** (80 mg, 0.2 mmol) dissolved in dry THF (0.1 M, 2 mL) under argon atmosphere was added ^{t}BuOLi (36 µL, 2.0 equiv, 1 M in THF). The reaction mixture was stirred at room temperature for 12 h. The reaction mixture was quenched with saturated NH_4Cl solution (10 mL) and extracted with EtOAc (3 × 10 mL), dried over anhydrous Na_2SO_4 and filtered through pad of celite. The organic layer was evaporated under reduced pressure and purified by column chromatography (hexanes/EtOAc:70/30 solvent system) to afford the eliminated **12** (38 mg, 70 %) as a colorless semi solid; ^{1}H NMR (400 MHz, $CDCl_3$) δ 7.70 – 7.59 (m, 2H), 7.58 – 7.51 (m, 1H), 7.48 – 7.39 (m, 2H), 6.73 (d, *J* = 10.2 Hz, 1H),6.53 (td, J = 2.5, 1.0 Hz, 1H), 6.08 (d, *J* = 10.2 Hz, 1H), 4.50 (ddd, *J* = 19.4, 2.7, 1.7 Hz, 1H), 4.34 (dt, *J* = 14.3, 2.6 Hz, 1H), 3.32 (ddd, *J* = 6.3, 4.9, 2.4 Hz, 1H), 2.67 (dd, *J* = 16.3, 4.9 Hz, 1H), 2.58 (dd, *J* = 16.3, 9.1 Hz, 1H), 1.47 (s, 3H); ^{13}C NMR (126 MHz, $CDCl_3$) δ 198.1, 195.5, 150.6, 139.2, 137.9, 137.6, 132.5, 130.2, 129.4, 128.5, 70.5, 61.7, 39.4, 37.5, 23.4; HRMS (ESI) calcd for $C_{17}H_{17}O_3$ $[M+H]^+$: 269.1178; found: 269.1170; $[\alpha]^{20}_D$ = -198.80° (*c* 0.8, $CHCl_3$).

(3R,4aS,8aS)-4-Benzoyl-8a-methyl-3-phenyl-3,4,4a,8a-tetrahydro-2*H*-chromen-6(5*H*)-one (13):

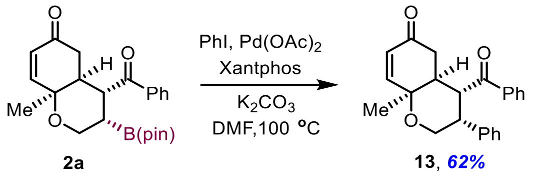

A mixture of potassium carbonate (56.0 mg, 0.40 mmol, 2.0 equiv), palladium acetate, (1.0 mg, 0.004 mmol, 0.02 equiv), Xantphos (3.5 mg, 0.006 mmol, 0.03 equiv), phenyl iodide (27 µL, 0.24 mmol, 1.2 equiv) and the boron compound **2a** (80.0 mg, 0.2 mmol, 1.0 equiv) in DMF (1.5 mL) and water (0.2 ml) were stirred at 100 °C for 15 h. The mixture was poured into cold water (10 mL), extracted with EtOAc (3 x 15 mL), dried over Na_2SO_4 and filtered. The filtrate was evaporated under reduced pressure and residue was purified by column chromatography (hexanes/EtOAc:70/30) to afford the cross coupling product **13** (43 mg, 62 %) as a brown semi solid; *dr* = >30:1; ^{1}H NMR (500 MHz, $CDCl_3$) δ 7.91 – 7.81 (m, 2H), 7.60 – 7.53 (m, 1H), 7.50 – 7.40 (m, 2H), 7.35 – 7.23 (m, 4H), 7.23 – 7.15 (m, 1H), 6.66 (d, *J* = 10.0 Hz, 1H), 6.00 (dd, *J* = 10.0, 0.9 Hz, 1H), 4.54 (dd, *J* = 11.8, 4.4 Hz, 1H), 3.95 (dd, *J* = 12.2, 5.6 Hz, 1H), 3.83 (t, *J* = 12.1 Hz, 1H), 3.66 (td, *J* = 11.9, 5.6 Hz, 1H), 2.89 (dd, *J* = 16.4, 13.7 Hz, 1H), 2.48 (dt, *J* = 13.7, 4.2 Hz, 1H), 1.98 (dd, *J* = 16.4, 4.0 Hz, 1H), 1.81 (s, 3H); ^{13}C NMR (101 MHz, $CDCl_3$) δ 199.0, 198.1, 150.5, 139.5, 135.8, 133.7, 129.2, 128.9, 128.2, 128.0, 127.3, 69.9, 66.5, 46.3, 38.7, 37.6, 35.3, 22.4; HRMS (ESI) calcd for $C_{23}H_{23}O_3$ $[M+H]^+$: 347.1642; found: 347.1637; $[\alpha]^{20}_D$ = +29.3° (*c* 0.25, $CHCl_3$).

4-Benzoyl-8a-methyl-3-(4-phenyl-1*H*-1,2,3-triazol-1-yl)-3,4,4a,8a-tetrahydro-2*H*-chromen-6(5*H*)-one (14):

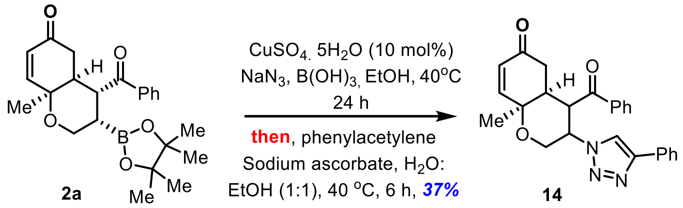

A pressure tube with magnetic stir bar was charged with substrate **2a** (80 mg, 0.2 mmol, 1.0 equiv.), $CuSO_4.5H_2O$ (5 mg, 0.02 mmol, 10 mol %), $B(OH)_3$ (14 mg, 0.22 mmol, 1.1 equiv.), NaN_3 (20 mg, 0.3 mmol, 1.5 equiv.) and ethanol (2 mL). The resulting mixture was vigorously stirred at 40 °C for 24 h. After this period, sodium ascorbate (2 mg, 3 mol %), phenylacetylene (26.4 µL, 0.24 mmol, 1.2 equiv.) and water (2 mL) were added at room temperature. Again, the resulting mixture was vigorously stirred at 40 °C for 24 h and washed with saturated NaCl solution (5 mL) and then extracted with ethyl acetate (3 x 5 mL). The organic layer was washed with a saturated aqueous solution of NaCl, dried under $MgSO_4$, filtered and concentrated. The crude product was purified by flash chromatography

using hexane:EtOAc (70:30) as an eluent, providing **14** as a white semi solid with 37% yield (23 mg); dr = >20:1; ^{1}H NMR (500 MHz, $CDCl_3$) δ 7.94 (s, 1H), 7.91 – 7.84 (m, 2H), 7.77 (dd, *J* = 8.2, 1.1 Hz, 2H), 7.60 – 7.51 (m, 1H), 7.47 – 7.40 (m, 2H), 7.40 – 7.34 (m, 2H), 7.32 – 7.26 (m, 1H), 6.67 (d, *J* = 10.0 Hz, 1H), 6.01 (d, *J* = 10.1 Hz, 1H), 5.29 (td, *J* = 11.4, 5.7 Hz, 1H), 5.11 (dd, *J* = 11.3, 4.6 Hz, 1H), 4.38 (t, *J* = 11.7 Hz, 1H), 4.14 (dd, *J* = 11.9, 5.8 Hz, 1H), 2.75 (dd, *J* = 15.9, 13.8 Hz, 1H), 2.63 (dt, *J* = 13.7, 4.1 Hz, 1H), 2.00 (dd, *J* = 15.8, 3.5 Hz, 1H), 1.86 (s, 3H); ^{13}C NMR (101 MHz, $CDCl_3$) δ 197.5, 197.2, 149.8, 147.0, 134.8, 134.3, 130.3, 129.3, 129.2, 128.9, 128.4, 128.3, 125.8, 122.0, 70.3, 63.4, 52.5, 46.5, 39.3, 35.0, 22.2; HRMS (ESI) calcd for $C_{25}H_{24}N_3O_3$ $[M+H]^+$: 414.1818; found: 414.1822; $[\alpha]^{20}_D$ = -95.37° (*c* 1.26, $CHCl_3$).

(4R,4aS,8aS)-4-benzoyl-8a-methyl-3,4,4a,8a-tetrahydro-2H-chromen-6(5H)-one (15):

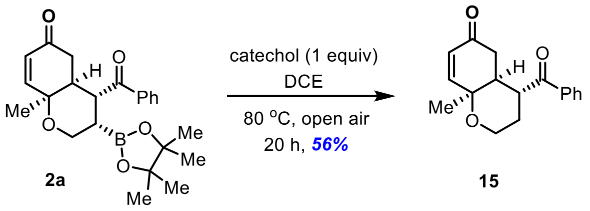

A 10 mL RB flask with magnetic stir bar was charged with substrate **2a** (80 mg, 0.2 mmol, 1.0 equiv.) and was dissolved in DCE solvent followed by addition of catechol (22 mg, 0.2 mmol, 1.0 equiv.). The resulting clear solution was stirred for 20 h at 80 °C open to air and then allowed to cool to room temperature. The reaction mixture was diluted with 3 mL water and the aqueous layer was extracted with CH_2Cl_2 (3 mL X 2). The combined organic layer was evaporated *in vacuo*, dried over anhyd. Na_2SO_4 and purified by silica-gel column chromatography (20% EtOAc in Hexanes) to obtain **15** in 56% yield (23 mg) as a colourless semi solid; ^{1}H NMR (400 MHz, $CDCl_3$) δ 7.91 (d, *J* = 7.3 Hz, 2H), 7.65 – 7.52 (m, 1H), 7.50 – 7.41 (m, 2H), 6.66 (dd, *J* = 10.4, 2.7 Hz, 1H), 6.09 (d, *J* = 10.4 Hz, 1H), 4.32 – 4.21 (m, 1H), 4.01 (q, *J* = 2.9 Hz, 1H), 3.85 (dd, *J* = 11.5, 2.4 Hz, 1H), 3.60 – 3.43 (m, 1H), 3.24 (dd, *J* = 16.9, 6.0 Hz, 1H), 2.80 (dd, *J* = 16.9, 6.4 Hz, 1H), 2.61 (qd, *J* = 17.3, 3.0 Hz, 2H), 1.40 (s, 3H); ^{13}C NMR (101 MHz, $CDCl_3$) δ 197.1, 196.0, 152.4, 136.9, 133.6, 130.8, 128.8, 128.3, 78.5, 71.6, 67.2, 42.2, 40.6, 24.5; HRMS (ESI) calcd for $C_{17}H_{17}O_3$ $[M-H]^-$: 269.1172; found: 269.1175; $[\alpha]^{20}_D$ = -69.86° (*c* 0.78, $CHCl_3$).

(3S,4R,4aS,10aR)-4-Benzoyl-3-(4,4,5,5-tetramethyl-1,3,2-dioxaborolan-2-yl)octahydrofuro[2,3-i]chromen-6(2*H*)-one (16):

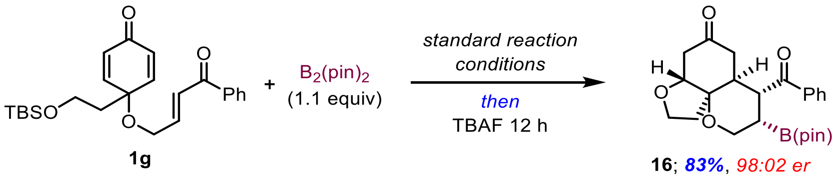

A solution of $Cu(CH_3CN)_4PF_6$ (2 mg, 0.005 mmol, 2.5 mol%), (*S*)-SEGPHOS (6 mg, 0.001 mmol, 5 mol%), $B_2(pin)_2$ (54 mg, 0.21 mmol, 1.1 equiv) and ^tBuOLi (35 µL, 2 equiv, 1 M in THF) in dry THF (2.0 ml) was stirred at room temperature for 15 min and then maintained at -78 °C. A solution of enone **1g** (80 mg, 0.20 mmol 1.0 equiv) in dry THF (1.0 mL) was added via syringe and the resulting mixture was stirred at -78 °C for 3 h then TBAF (112 µL, 2 equiv, 1.0 M in THF) added to the reaction mixture via syringe and continuously stirred for 12 h. The reaction mixture was quenched with saturated NH_4Cl solution (10 mL) and extracted with EtOAc (3 × 10 mL) and dried over anhydrous Na_2SO_4, filtered, and concentrated *in vacuo.* The resultant crude product was purified by column chromatography (n-hexane/EtOAc: 50/50 solvent systems) afforded the compound **16** (69 mg, 83 %) as a colourless semi solid; *dr* = 10:1; ^{1}H NMR (500 MHz, $CDCl_3$) δ 7.84 – 7.72 (m, 2H), 7.60 – 7.55 (m, 1H), 7.52 – 7.45 (m, 2H), 4.11 (t, *J* = 12.4 Hz, 1H), 4.01 (dd, *J* = 12.3, 5.2 Hz, 1H), 3.82 (dd, *J* = 7.2, 5.3 Hz, 1H), 3.71 (dt, *J* = 8.7, 7.6 Hz, 1H), 3.53 (d, *J* = 4.9 Hz, 1H); 3.52– 3.47 (m, 1H), 2.97 (dd, *J* = 15.2, 13.1 Hz, 1H), 2.67 (dd, *J* = 15.8, 5.3 Hz, 1H), 2.48 – 2.30 (m, 3H), 2.15 (ddd, *J* = 12.6, 7.5, 4.8 Hz, 1H), 1.70 (dt, *J* = 12.4, 5.1 Hz, 1H), 1.44 (dt, *J* = 12.9, 7.7 Hz, 1H), 1.25 (s, 6H), 1.15 (s, 6H); ^{13}C NMR (101 MHz, $CDCl_3$) δ 209.3, 203.6, 135.8, 133.4, 129.2, 129.1, 128.3, 128.2, 83.6, 83.5, 80.4, 66.3, 63.2, 46.9, 42.5, 42.4, 37.7, 34.3, 25.0, 24.6; HRMS (ESI) calcd for $C_{24}H_{32}BO_6$ $[M+H]^+$: 427.2292; found: 427.2293; $[\alpha]^{20}_D$ = -119.1° (*c* 0.7, $CHCl_3$); 98:02 *er*; Chiral HPLC analysis of the product: Daicel Chiralpak IC 250X4.6 mm 5µ column; hexane/2-propanol = 80/20, detected at 254 nm, Flow rate = 1 mL/min, Retention times: 12.035 min (major), 10.814 min (minor).

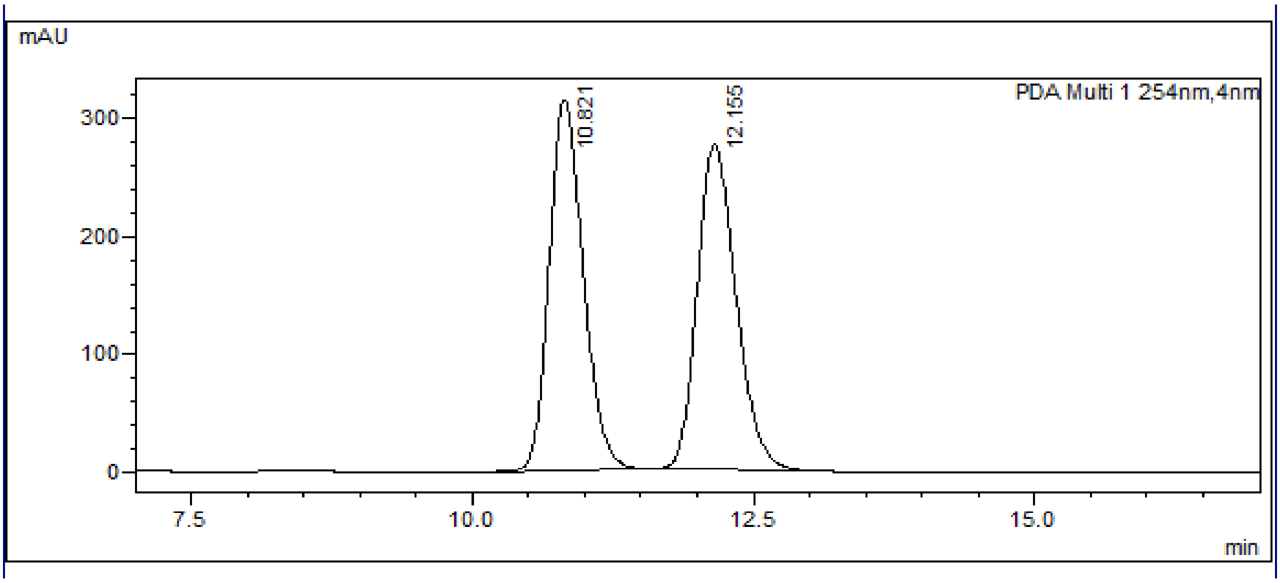

<Peak Table>

PDA Ch1 254nm

Peak#	Ret. Time	Area	Height	Area%	Height%
1	10.821	6606021	312793	50.019	53.197
2	12.155	6600876	275198	49.981	46.803
Total		13206898	587991	100.000	100.000

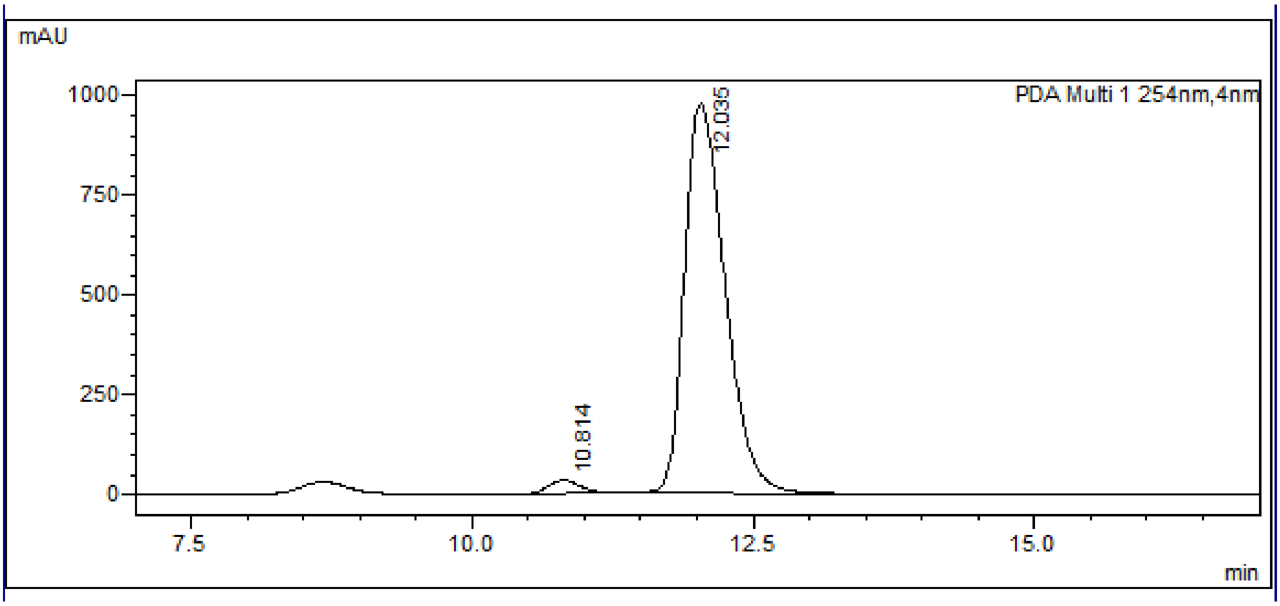

<Peak Table>

PDA Ch1 254nm

Peak#	Ret. Time	Area	Height	Area%	Height%
1	10.814	588398	31426	2.340	3.113
2	12.035	24559378	977937	97.660	96.887
Total		25147775	1009363	100.000	100.000

2h. X-Ray crystallographic data

2ha. X-ray crystallographic data for compound 2n:

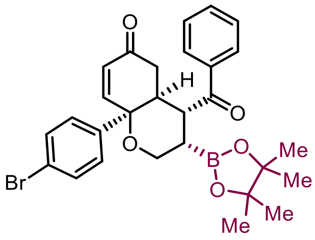

2n, CCDC-2112453

The purified compound **2n** was dissolved in a mixed solvent of acetonitrile/*n*-hexane (1:1), and placed in a dark cabinet for slowly evaporation. Orange crystals were collected after few days for X-ray analysis.

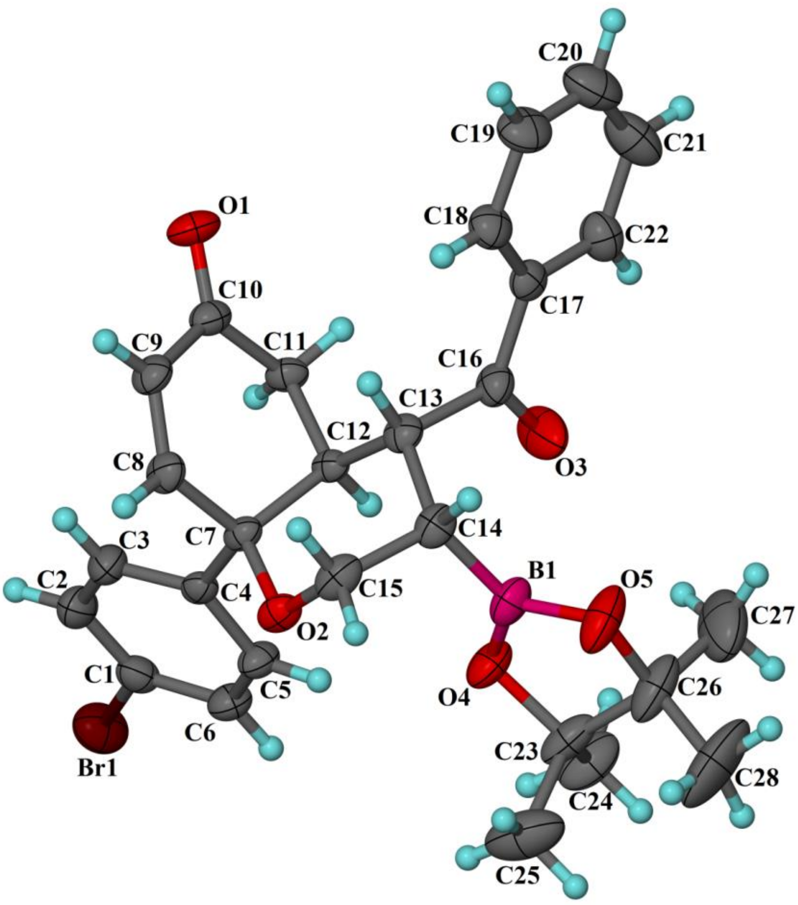

Supplementary Figure 1: ORTEP diagram of compound **2n** (KB151) compound with the atom-numbering. Displacement ellipsoids are drawn at the 35% probability level and H atoms are shown as small spheres of arbitrary radius. The absolute configuration of the compound was assigned based on the anomalous dispersion method.

Crystal data for Compound 2n (KB151): $C_{28}H_{30}O_5B_1Br_1$, *M* = 537.24, Orthorhombic, space group $P2_12_12_1$ (No.19), *a* = 6.1474(4)Å, *b* = 14.3847(8)Å, *c* = 29.8976(17)Å, *α* = 90°, *β* = 90°, *γ* = 90°, *V* = 2643.8(3)Å^3, *Z* = 4, D_c = 1.350 g/cm^3, F_{000} = 1112, Bruker D8 QUEST PHOTON-100, Mo-Kα radiation, λ = 0.71073 Å, *T* = 293(2)K, $2\theta_{max}$ = 55º, μ= 1.590 mm^{-1}, 27844 reflections collected, 6051 unique (R_{int} = 0.0990), 320 parameters, *R1* = 0.0534, *wR2* = 0.1231, *R* indices based on 3795 reflections with I > 2σ(I) (refinement on F^2), Final *GooF* = 1.028, largest difference hole and peak = -0.338 and 0.335 e.Å^{-3}.

Data collection and Structure solution details: Single crystal X-ray data were collected at room temperature on a Bruker D8 QUEST equipped with a four-circle kappa diffractometer and Photon 100 detector. An Iμs microfocus Mo source (λ=0.71073Å) supplied the multi-mirror monochromated incident beam. A combination of Phi and Omega scans were used to collect the necessary data.

Integration and scaling of intensity data were accomplished using SAINT program.[7] The structures were solved by Direct Methods using SHELXS97[8] and refinement was carried out by full-matrix least-squares technique using SHELXL-2014/7.[8-9] Anisotropic displacement parameters were included for all non-hydrogen atoms. All H atoms were positioned geometrically and treated as riding on their parent C atoms, with C-H distances of 0.93--0.97 Å, and with U_{iso}(H) = 1.2U_{eq} (C) or 1.5U_{eq} for methyl atoms. The absolute configuration of the compound KB151 (C7, R; C12, S; C13, R; C14, S) was assigned based on the refinement of Flack parameter (0.079(10)).[10,11] The **CCDC** deposition number **2112453** contains the supplementary crystallographic data for this paper which can be obtained free of charge at https://www.ccdc.cam.ac.uk/structures/

2hb. X-ray crystallographic data for compound 10a:

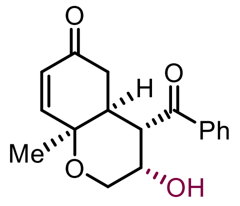

10a, CCDC-2071966

The purified compound **10a** was dissolved in a mixed solvent of dichloromethane/*n*-hexane (1:3), and placed in a dark cabinet for slowly evaporation. Colourless crystals were collected after few days for X-ray analysis.

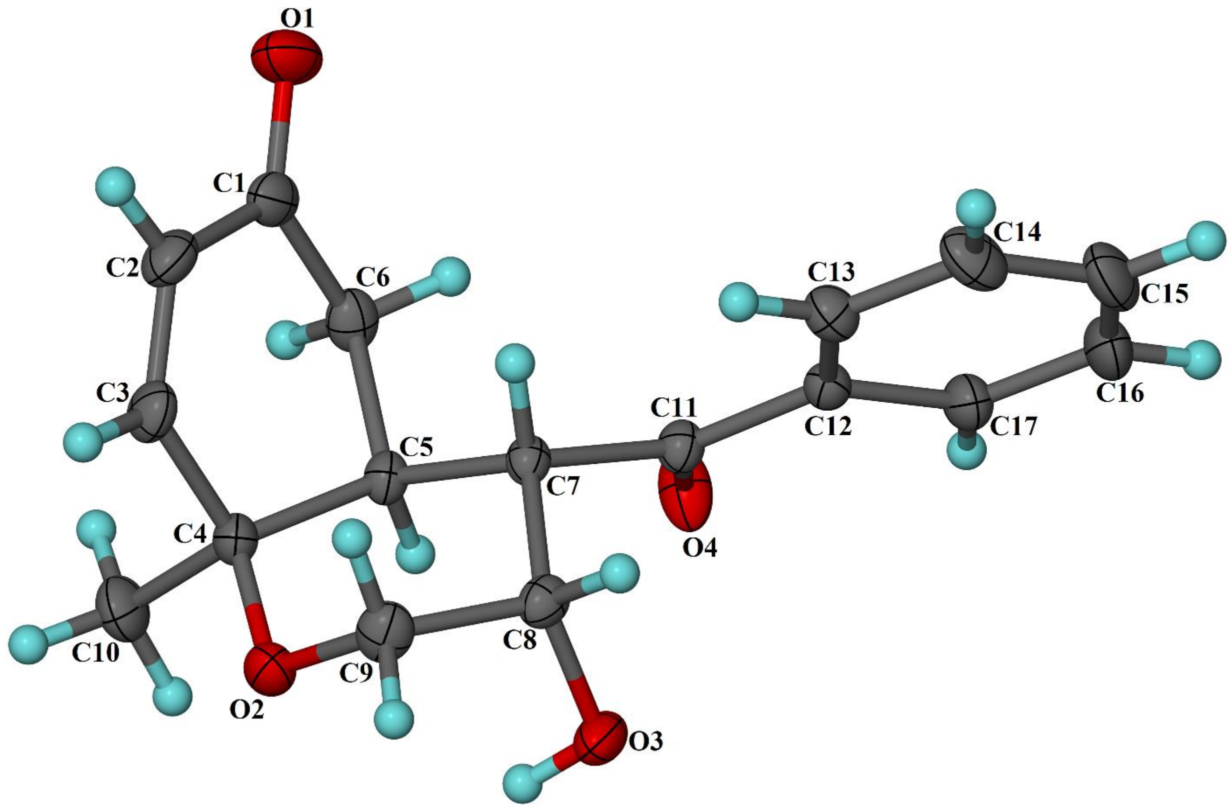

Supplementary Figure 2: ORTEP diagram of compound **10a** (KA942) with the atom-numbering. Displacement ellipsoids are drawn at the 35% probability level and H atoms are shown as small spheres of arbitrary radius.

2hc. X-ray crystallographic data for compound 11a:

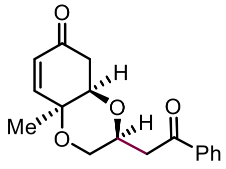

11a, CCDC-2071967

The purified compound **11a** was dissolved in a mixed solvent of dichloromethane/*n*-hexane (1:3), and placed in a dark cabinet for slowly evaporation. Colourless crystals were collected after few days for X-ray analysis.

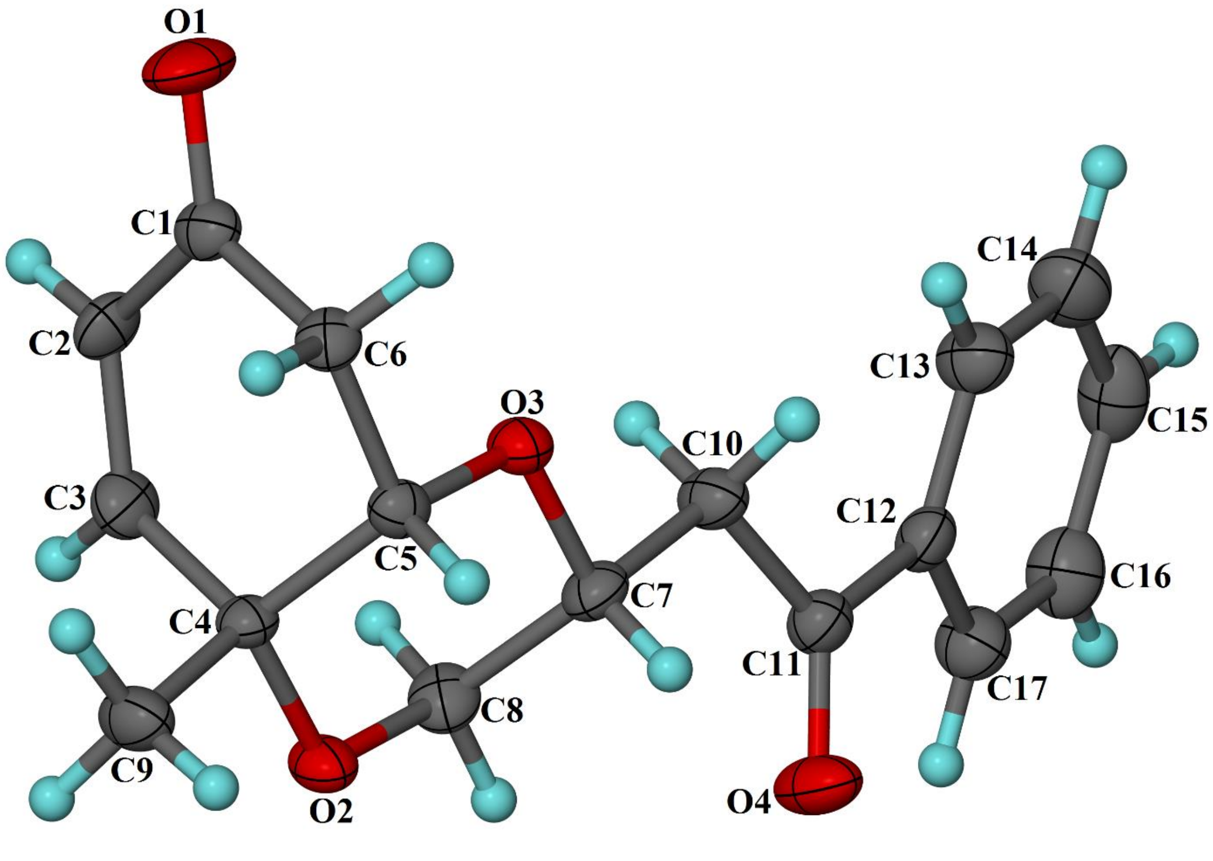

Supplementary Figure 3: ORTEP diagram of compound **11a** (KA950) with the atom-numbering. Displacement ellipsoids are drawn at the 35% probability level and H atoms are shown as small spheres of arbitrary radius.

Crystal data for compound 10a (KA942): $C_{17}H_{18}O_4$, *M* = 286.31, Monoclinic, space group $P2_1/c$ (No.14), *a* = 9.9807(11)Å, *b* = 19.629(2)Å, *c* = 7.3867(7)Å, *α* = 90°, *β* = 95.570(3)°, *γ* = 90°, *V* = 1440.3(3)Å^3, *Z* = 4, D_c = 1.320 g/cm^3, F_{000} = 608, Bruker D8 QUEST PHOTON-100, Mo-Kα radiation, λ = 0.71073 Å, *T* = 293(2)K, $2\theta_{max}$ = 55º, μ= 0.094 mm^{-1}, 22095 reflections collected, 3287 unique (R_{int} = 0.0604), 250 parameters, *R1* = 0.0499, *wR2* =0.1203, *R* indices based on 2381 reflections with I > 2σ(I) (refinement on F^2), Final *GooF* = 1.082, largest difference hole and peak = -0.205 and 0.292 e.Å^{-3}.

Crystal data for compound 11a (KA950): $C_{17}H_{18}O_4$, *M* = 286.31, Orthorhombic, space group *Pbca* (No.61), *a* = 8.3541(9)Å, *b* = 10.7508(9)Å, *c* = 32.832(3)Å, *α* = 90°, *β* = 90°, *γ* = 90°, *V* = 1843.2(7)Å^3, *Z* = 8, D_c = 1.290 g/cm^3, F_{000} = 1216, Bruker D8 QUEST PHOTON-100, Mo-Kα radiation, λ = 0.71073 Å, *T* = 293(2)K, $2\theta_{max}$ = 50º, μ= 0.091 mm^{-1}, 9012 reflections collected, 2594 unique (R_{int} = 0.0773), 191 parameters, *R1* = 0.0661, *wR2* =0.1353, *R* indices based on 1457 reflections with I > 2σ(I) (refinement on F^2), Final *GooF* = 1.078, largest difference hole and peak = -0.273 and 0.304 e.Å^{-3}.

Data collection and Structure solution details: Single crystal X-ray data were collected at room temperature on a Bruker D8 QUEST equipped with a four-circle kappa diffractometer and Photon 100 detector. An Iμs microfocus Mo source (λ=0.71073Å) supplied the multi-mirror monochromated incident beam. A combination of Phi and Omega scans were used to collect the necessary data. Integration and scaling of intensity data were accomplished using SAINT program.[7] The structures were solved by Direct Methods using SHELXS97[8] and refinement was carried out by full-matrix least-squares technique using SHELXL-2014/7.[8-9] Anisotropic displacement parameters were included for all non-hydrogen atoms. All H atoms were positioned geometrically and treated as riding on their parent C atoms, with C-H distances of 0.93--0.97 Å, and with U_{iso}(H) = 1.2U_{eq} (C) or 1.5U_{eq} for methyl atoms. The O bound H atoms were located in the difference Fourier map and the positional parameters of H atoms were refined. The absolute configuration of the compound KA1125 (C7, R; C12, S; C13, R; C14, S) was assigned based on the refinement of Flack parameter (0.09(2))[10,11]. **CCDC 2071966** and **2071967** deposition numbers contain the supplementary crystallographic data for this paper which can be obtained free of charge at https://www.ccdc.cam.ac.uk/structures/

2i. Computational Studies:

Density functional theory (DFT) calculations were carried out by the Gaussian 09[12] program package. All the models were optimized in the gas phase by employing the B3LYP[13] functional, along with a mixed basis set approach, where SDD was used for Cu and 6-31G(d) for other atoms. Harmonic frequency calculations were performed for all stationary points to confirm them as minima and transition states. For better accuracy, single-point energy calculations were carried out by employing the M06[14]/SDD-6-311+G(d,p) level of theory. The solvent effect of THF was also taken into consideration in the mentioned single point energy calculations by using the SMD[15] solvation model. Zero-point energies and thermal corrections estimated by the B3LYP/SDD-6-31G(d) level of theory was incorporated into the final ΔG value. In order to reduce the computational expense, the ligand model was designed by replacing the two phenyl groups on each phosphorus atom with two methyl groups. The catalyst activation step with the original ligand was found to be similar vs. the modified ligand (ΔΔG = +0.4 kcal/mol). All the free energy values shown in the energy profile (Fig-B) are with respect to the separately taken active catalyst (the boryl copper complex) and the substrate (1a). This kind of approach (the above mentioned DFT method) for studying the transition metal catalysed reactions has been recommended by several benchmark studies on organometallic compounds.[16,17]

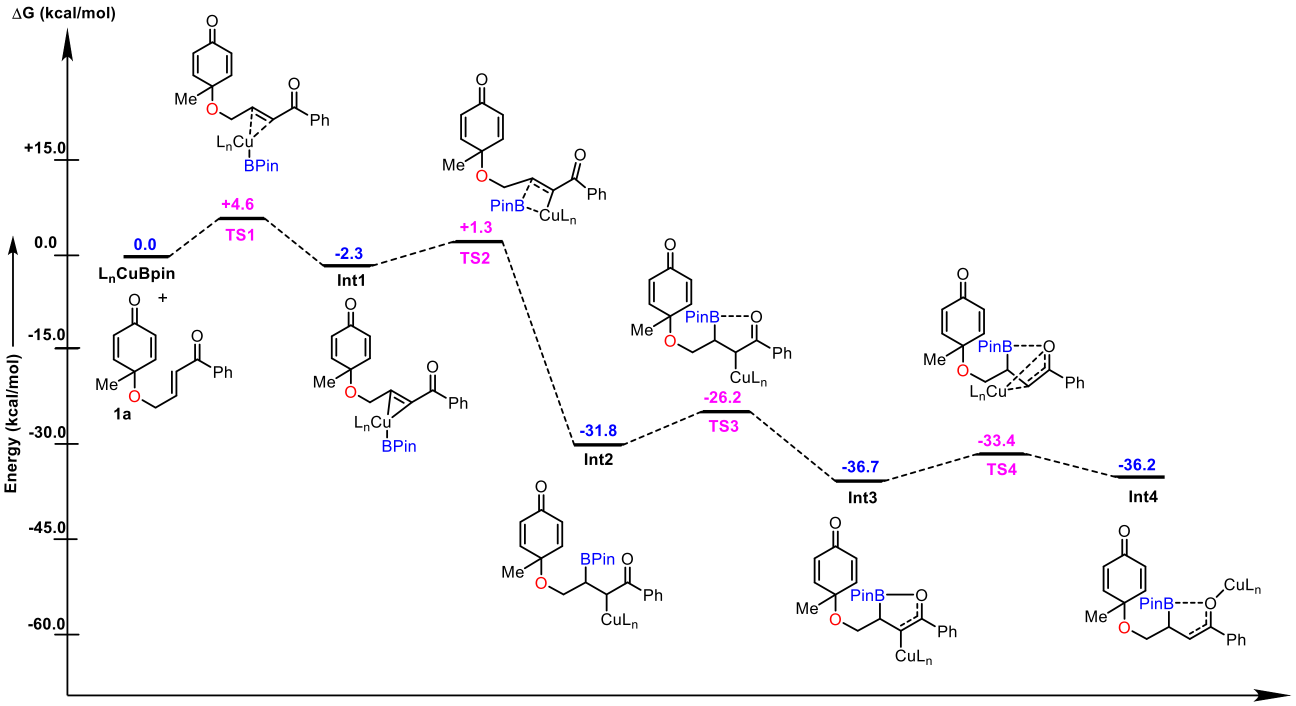

Supplementary Figure 4: Computed free energy pathway with relevant transition states. All the values are in kcal/mol with respect to the separate reactants L_nCu-Bpin and substrate **1a**.

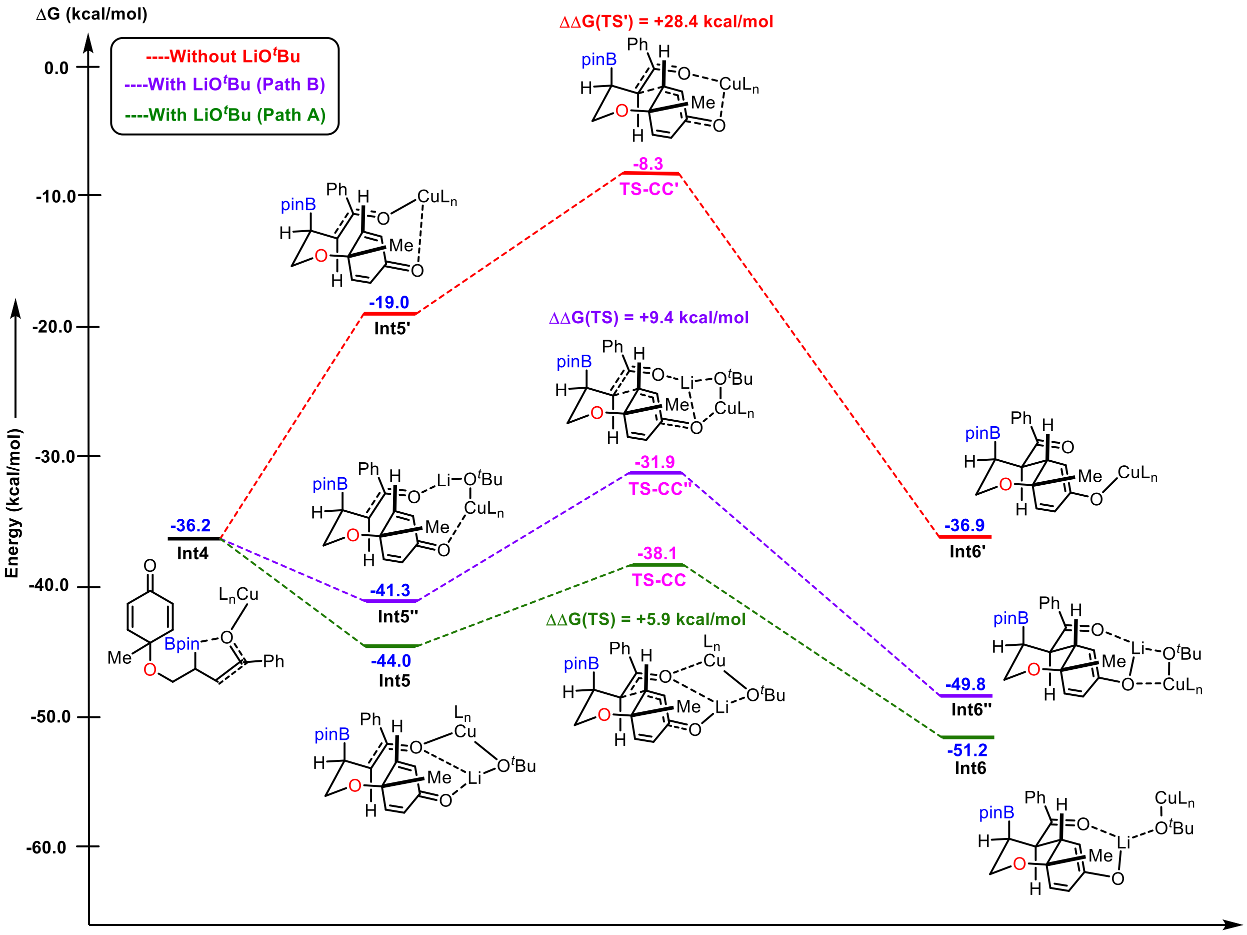

Supplementary Figure 5: Computed free energy pathway with relevant transition states (Continuation of Supplementary Figure 4)**.** All the values are in kcal/mol with respect to the separate reactants L_nCu-Bpin and substrate **1a**.

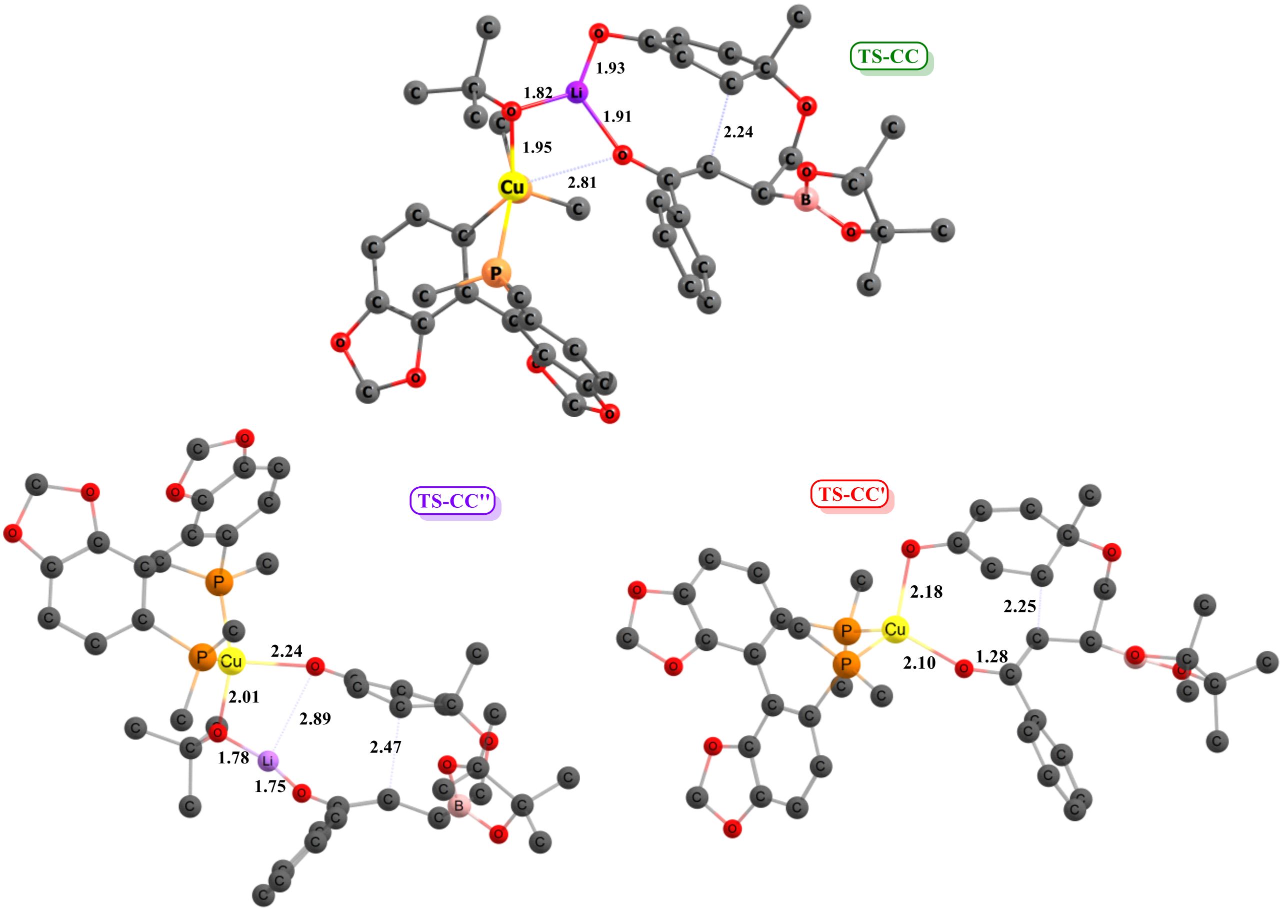

Supplementary Figure 6: The optimized figures of TS-CC and TS-CC’ and TS-CC’’. Hydrogen atoms have been removed for clarity. Bond lengths are shown in Å.

2j. NMR Spectra:

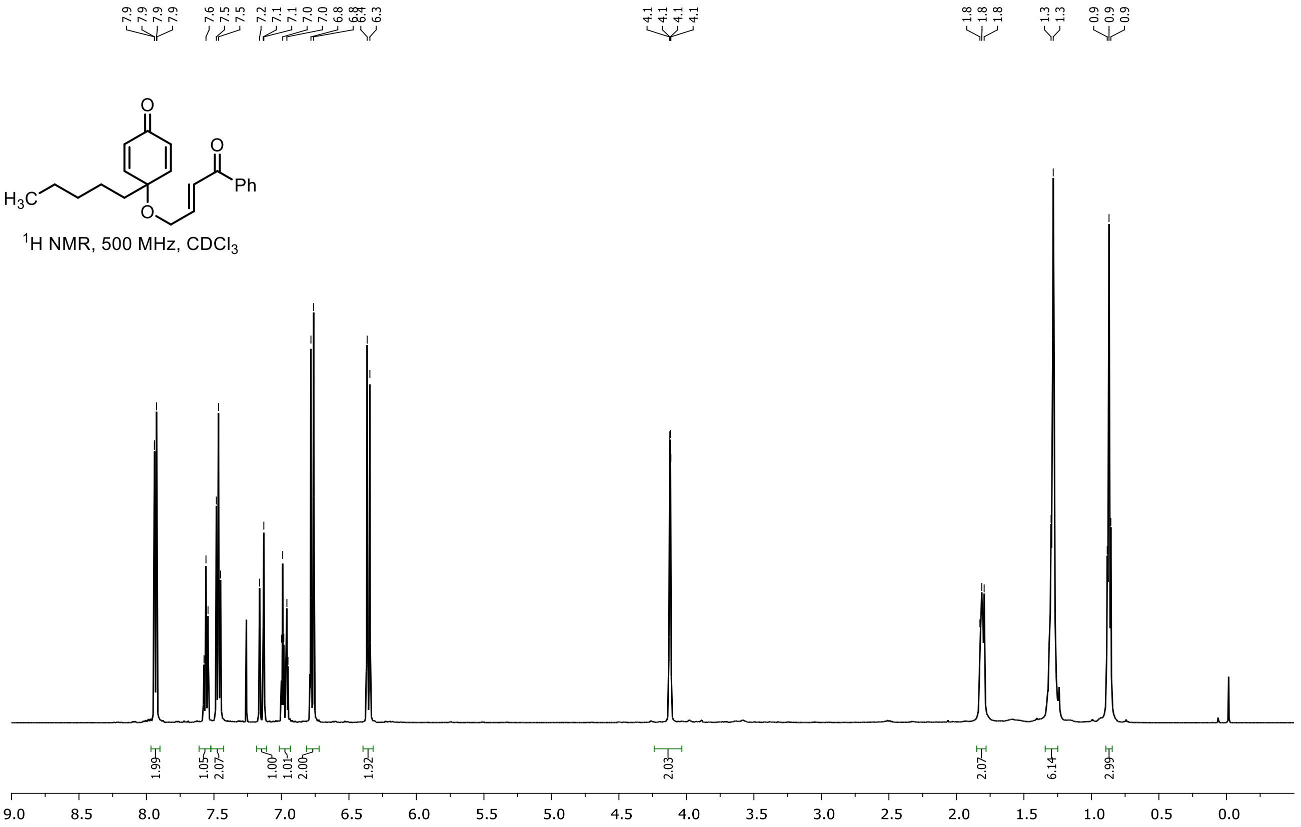

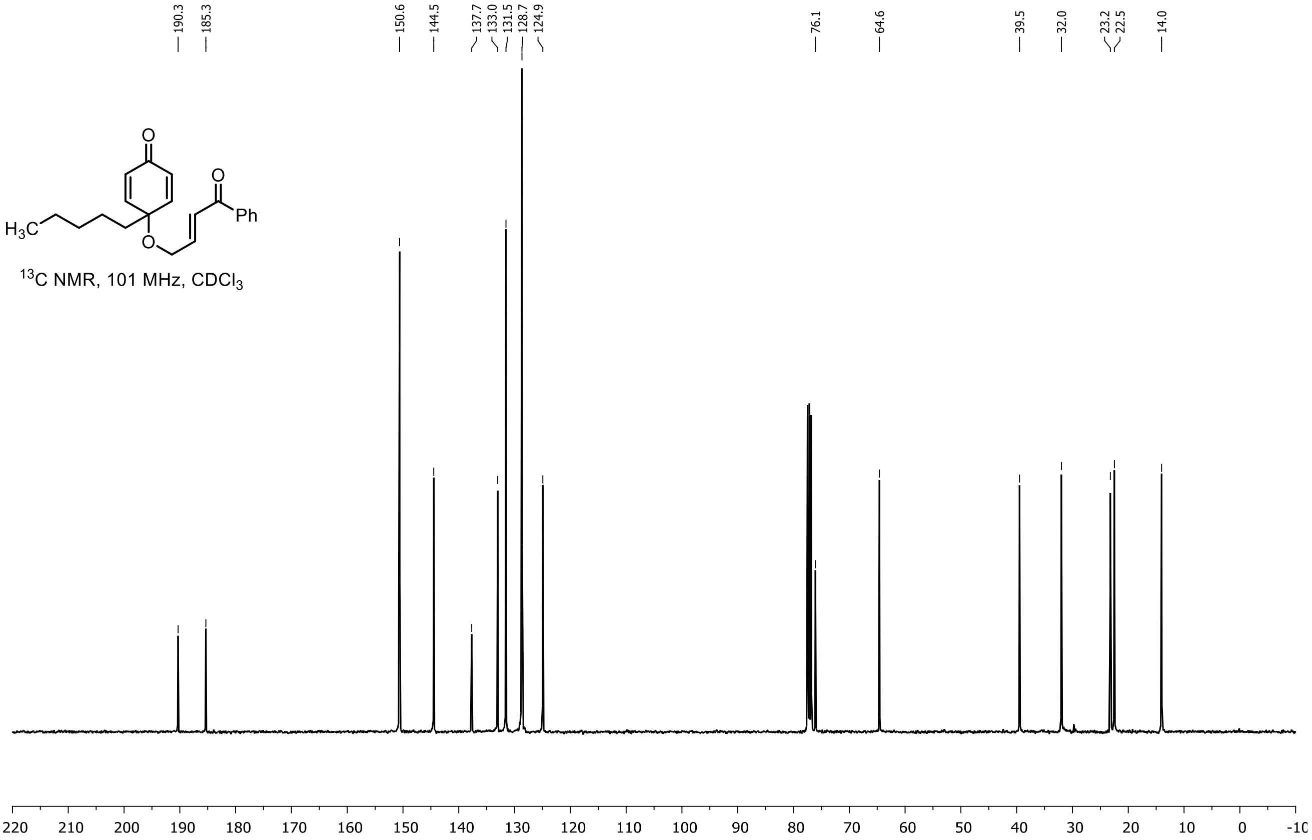

Supplementary Figure 7. **^{1}H NMR and ^{13}C NMR spectra of compound 1e.**

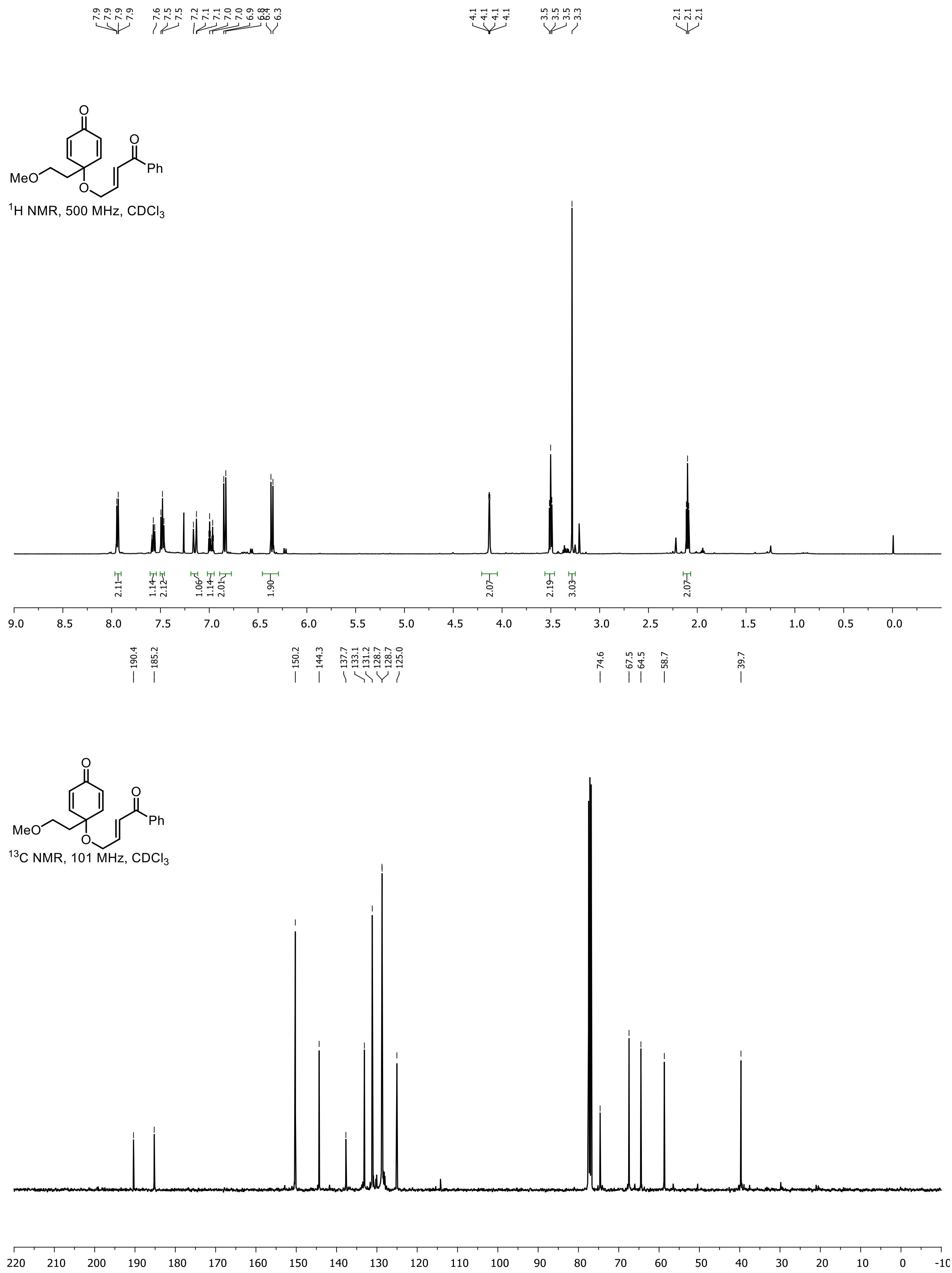

Supplementary Figure 8. ^{1}**H NMR and** ^{13}**C NMR spectra of compound 1f.**

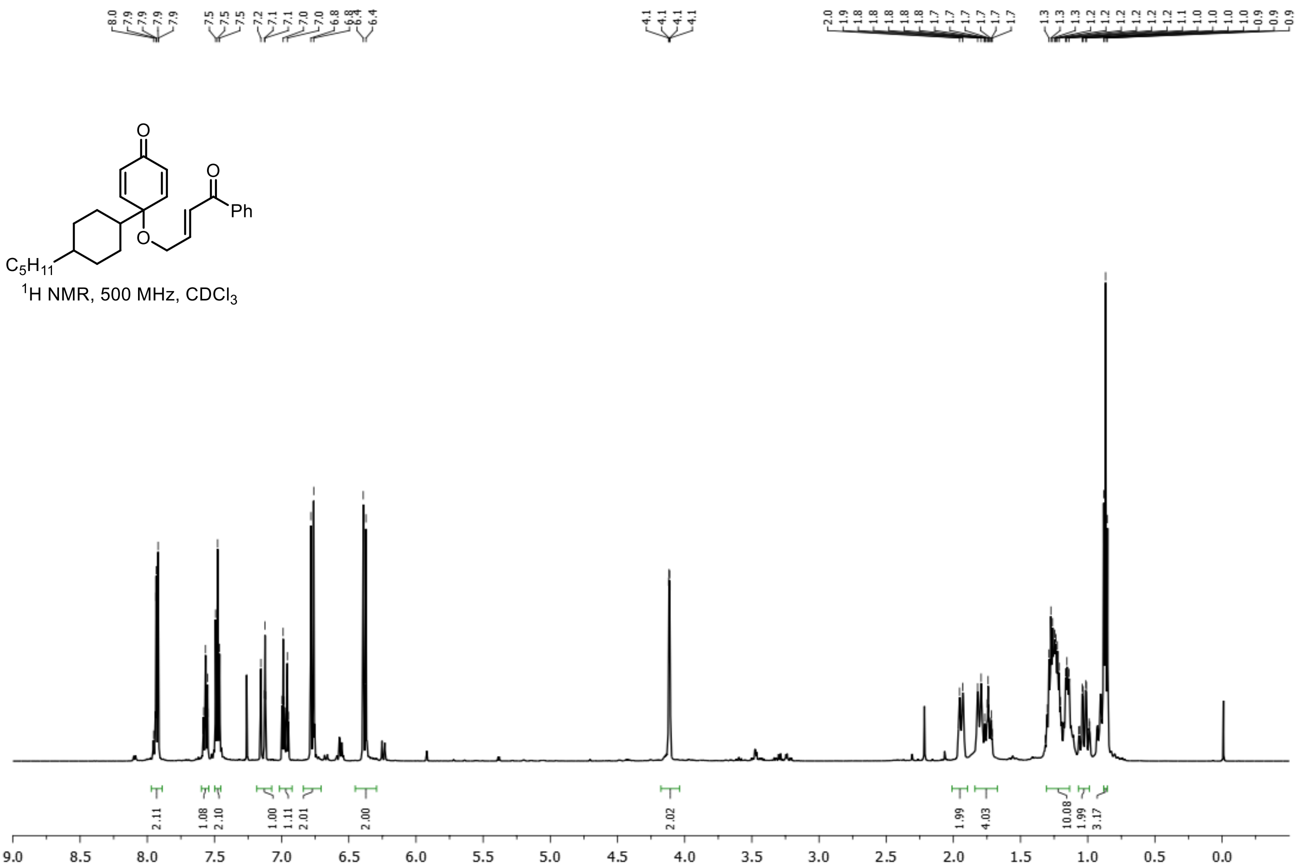

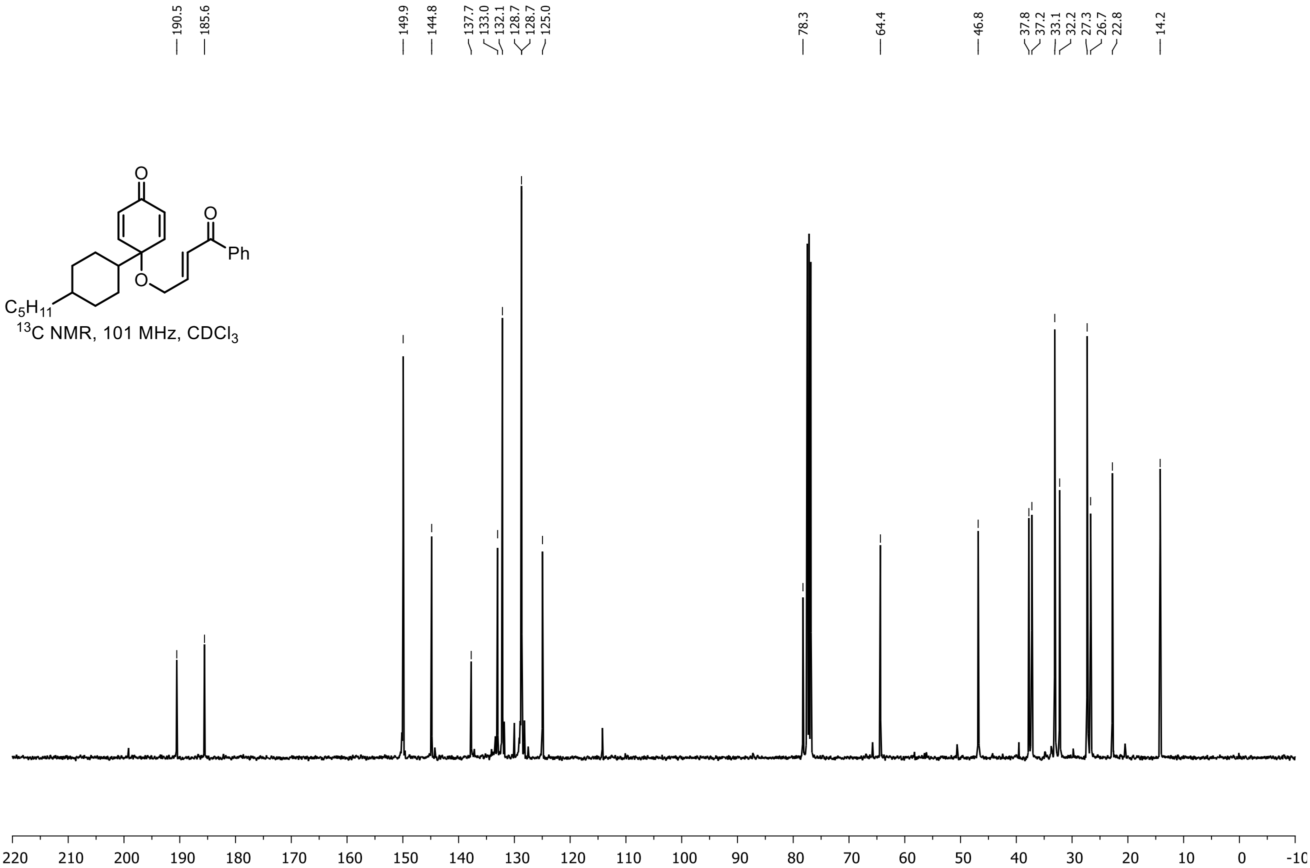

Supplementary Figure 9. **^{1}H NMR and ^{13}C NMR spectra of compound 1j.**

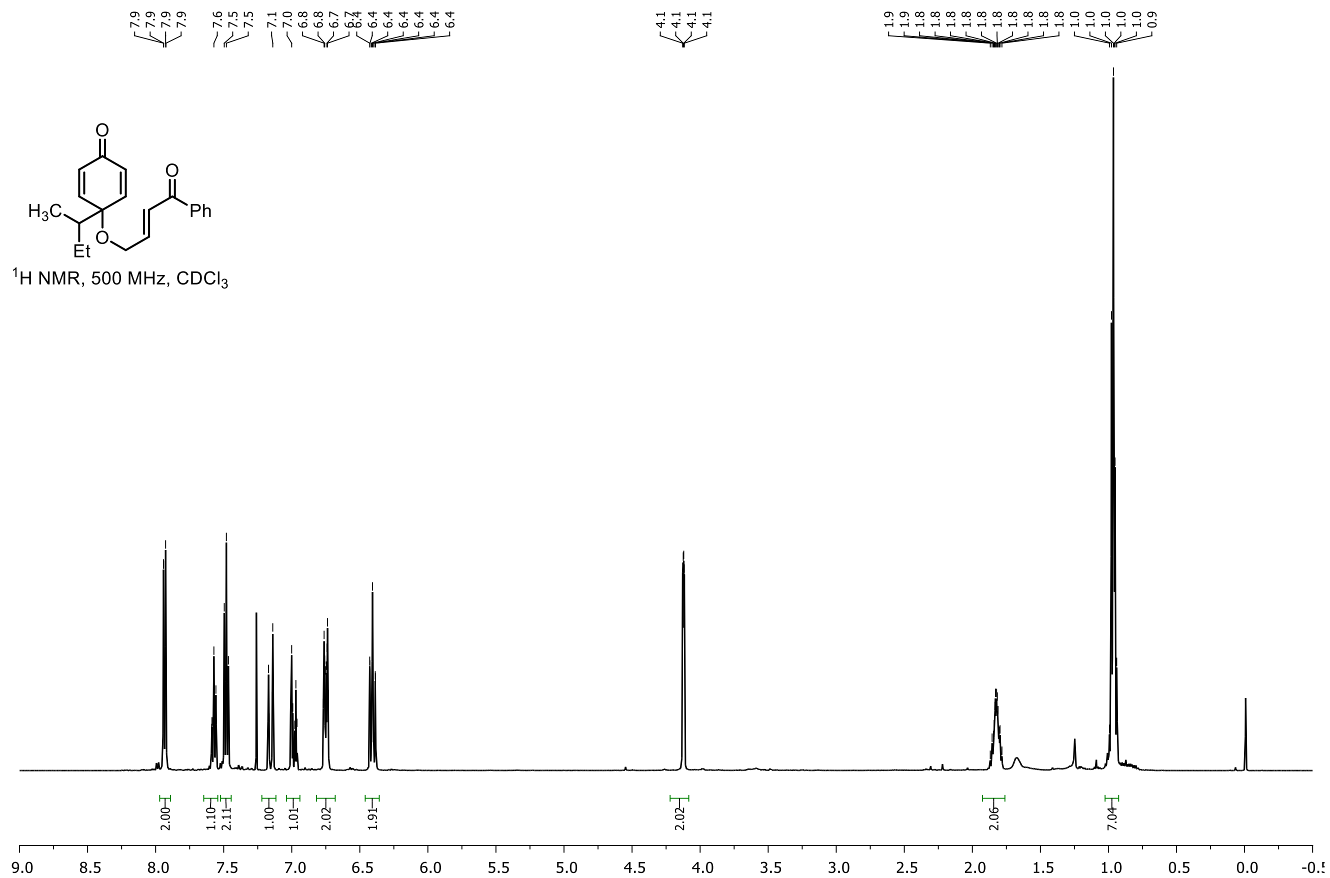

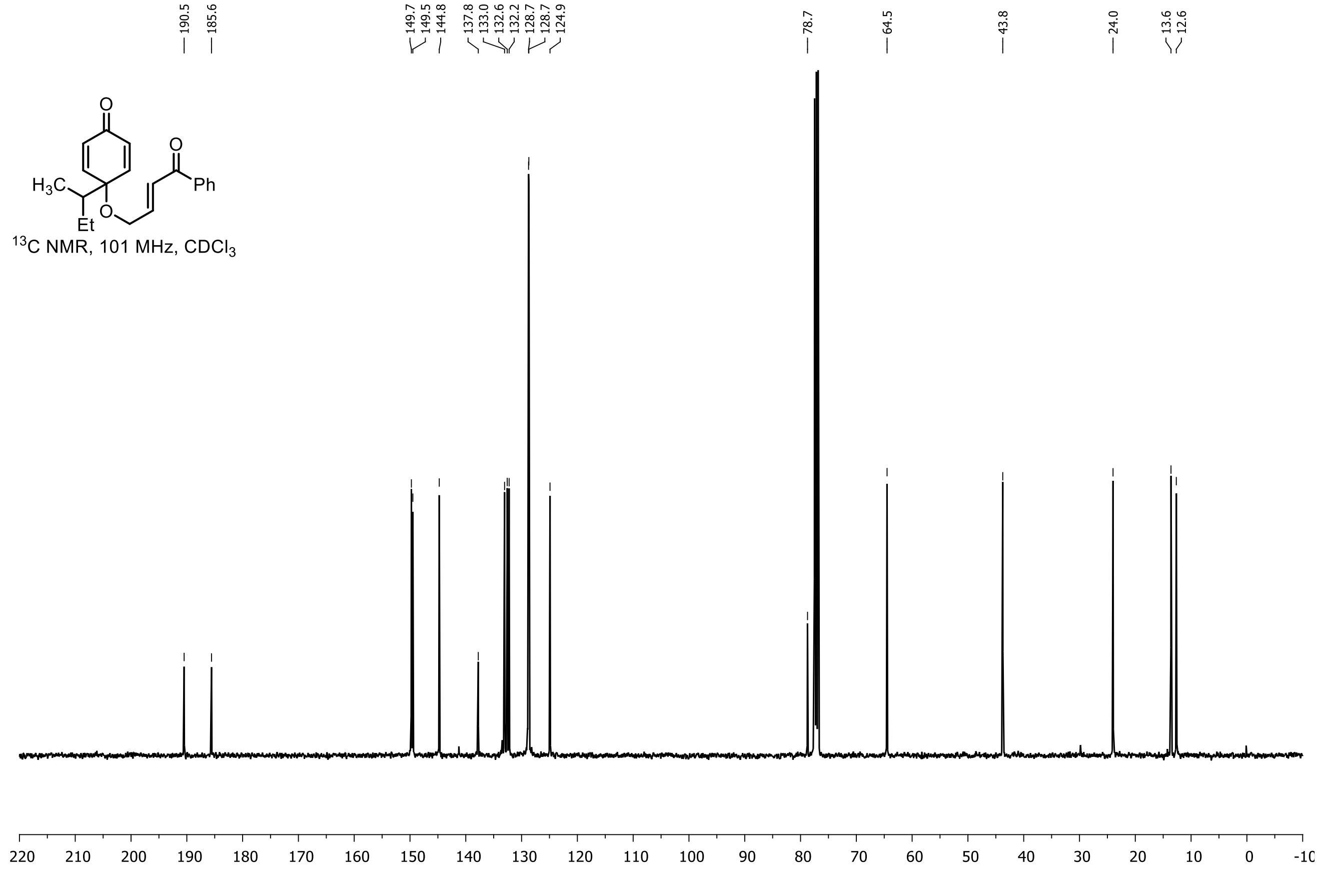

Supplementary Figure 10. **^{1}H NMR and ^{13}C NMR spectra of compound 1k.**

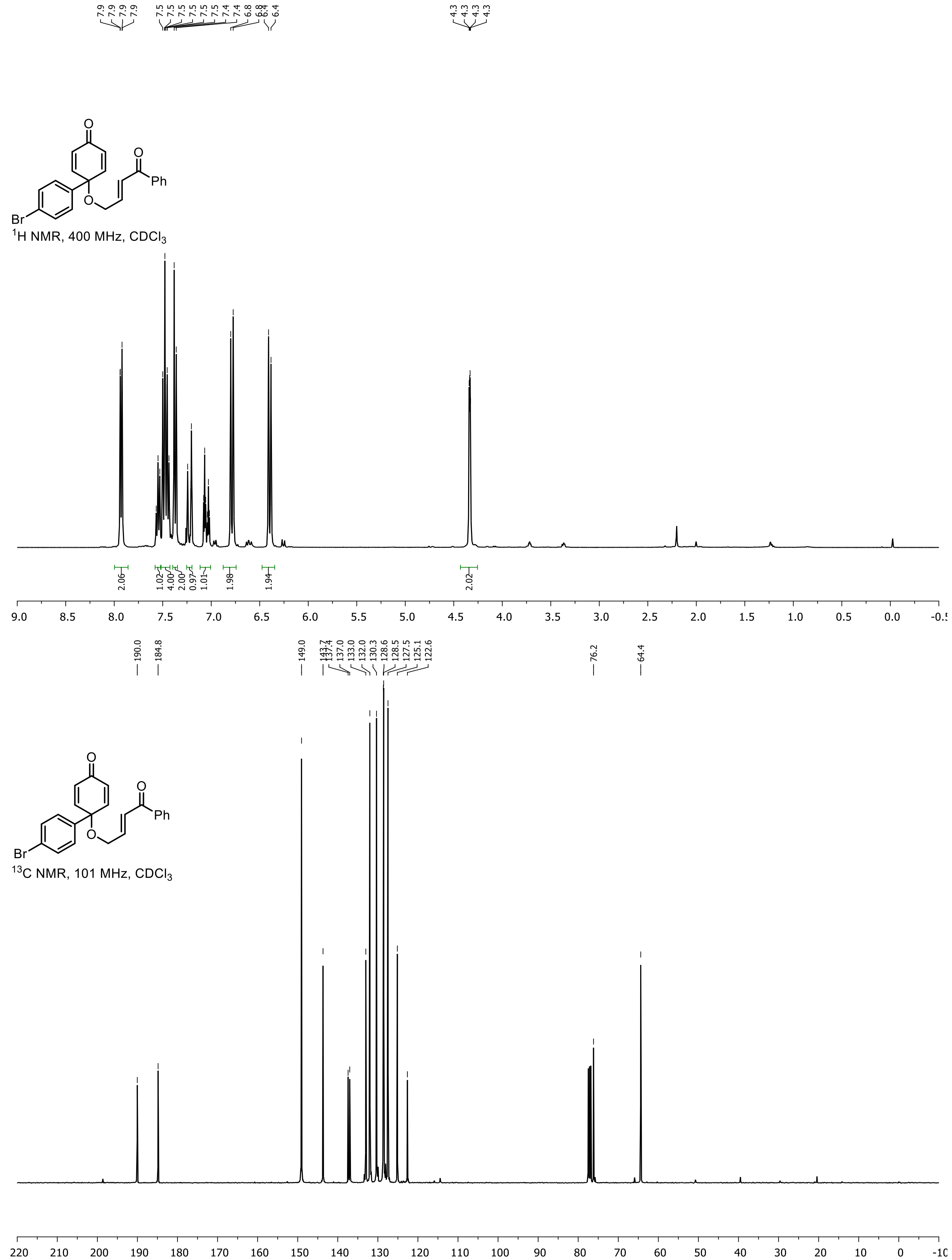

Supplementary Figure 11. ^{1}H NMR and ^{13}C NMR spectra of compound 1n.

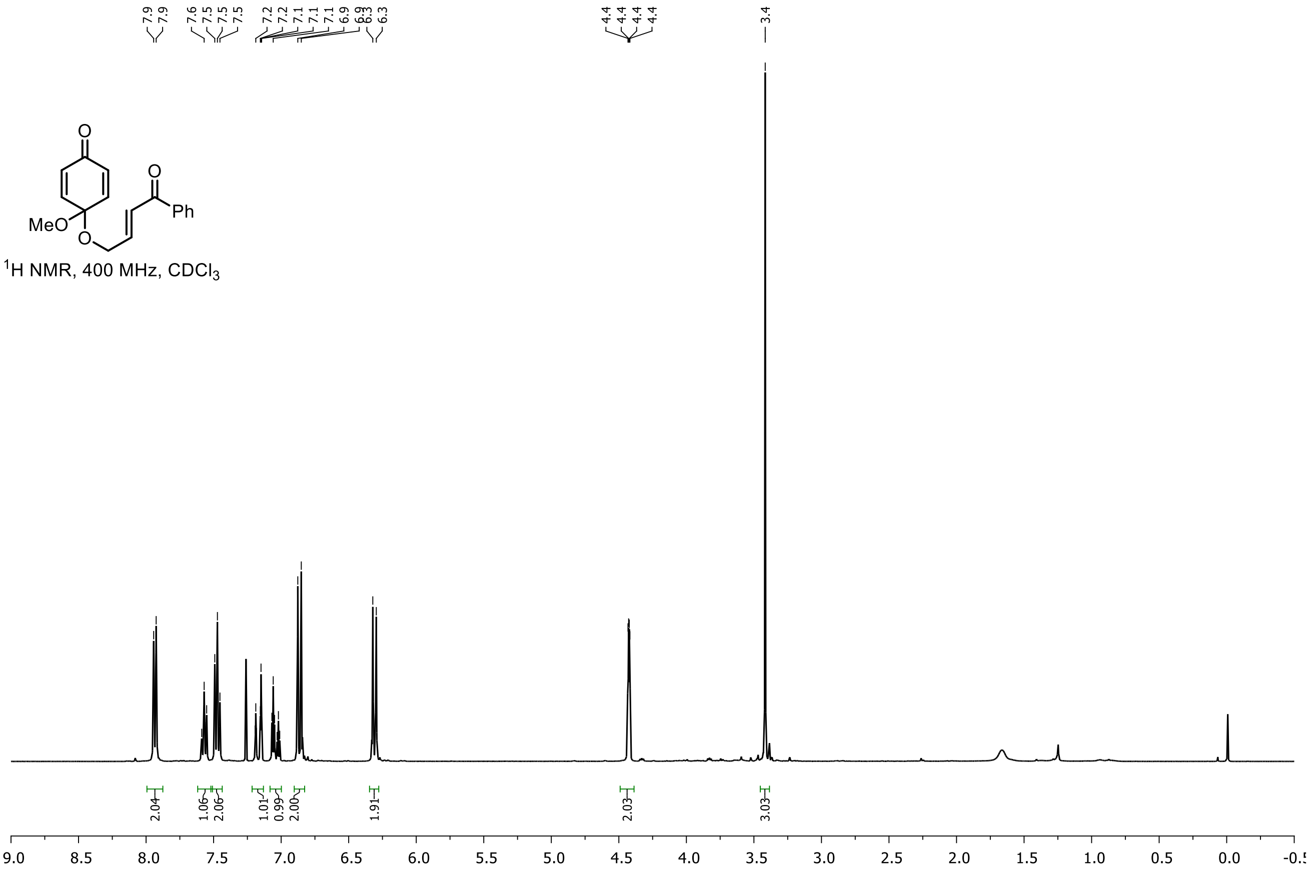

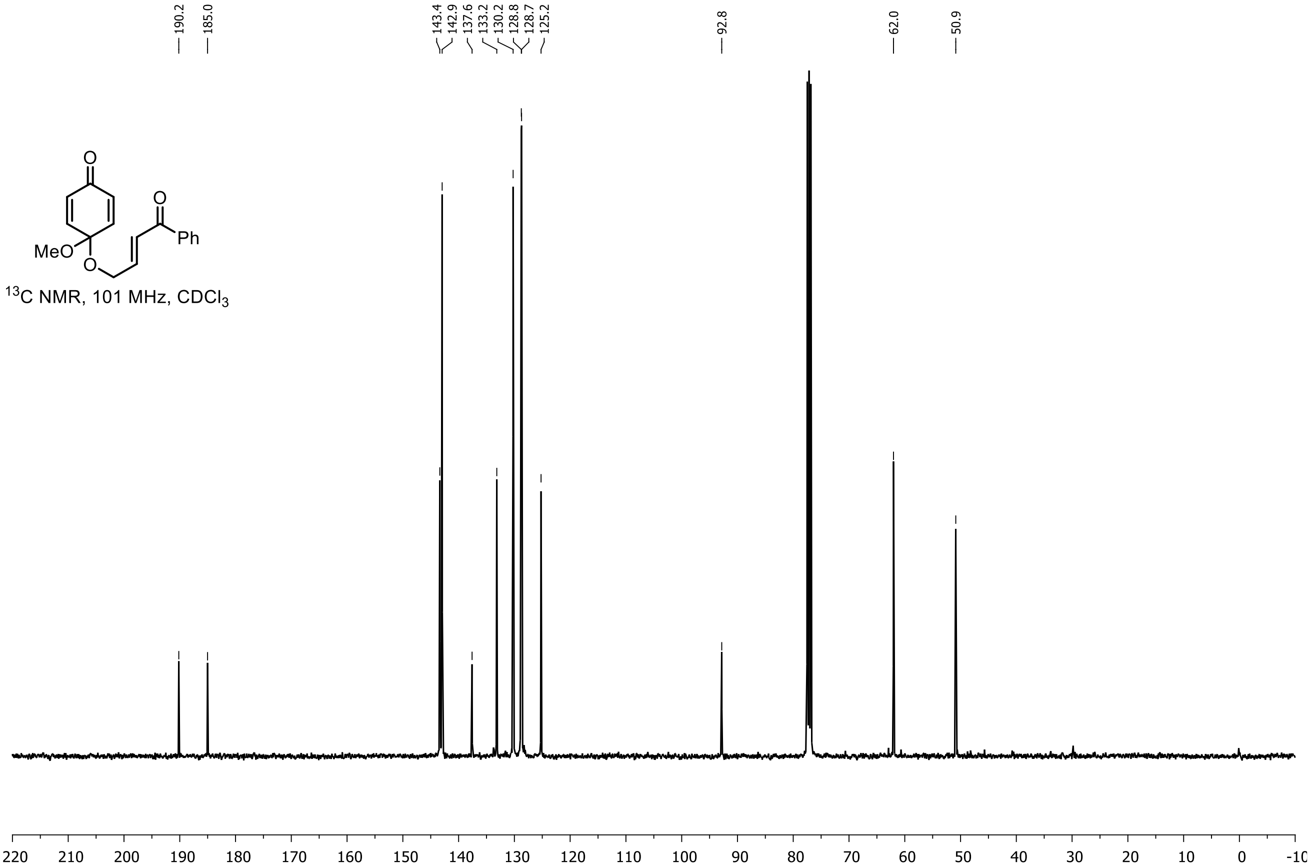

Supplementary Figure 12. ^{1}H NMR and ^{13}C NMR spectra of compound 1o.

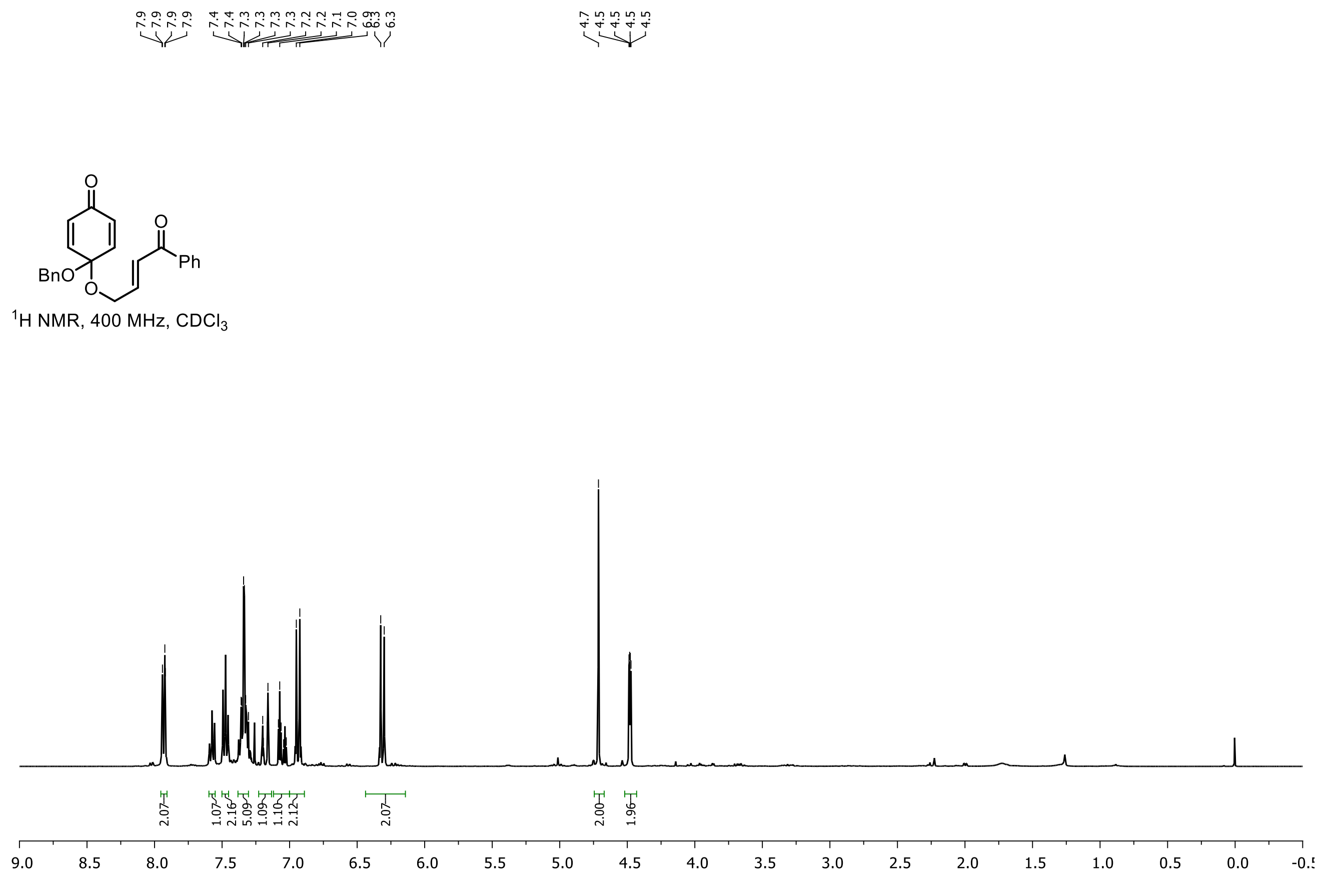

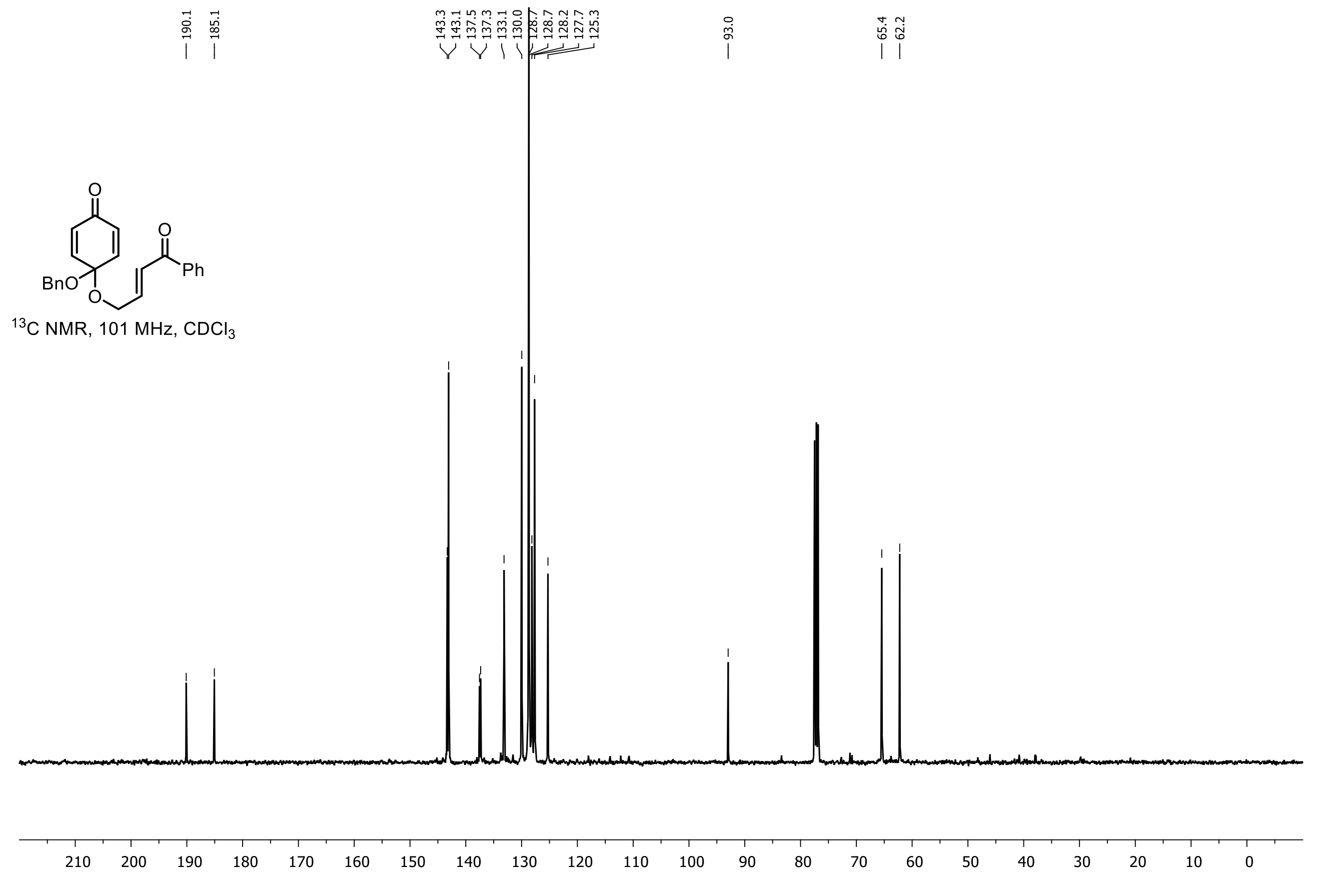

Supplementary Figure 13. ^{1}H NMR and ^{13}C NMR spectra of compound 1q.

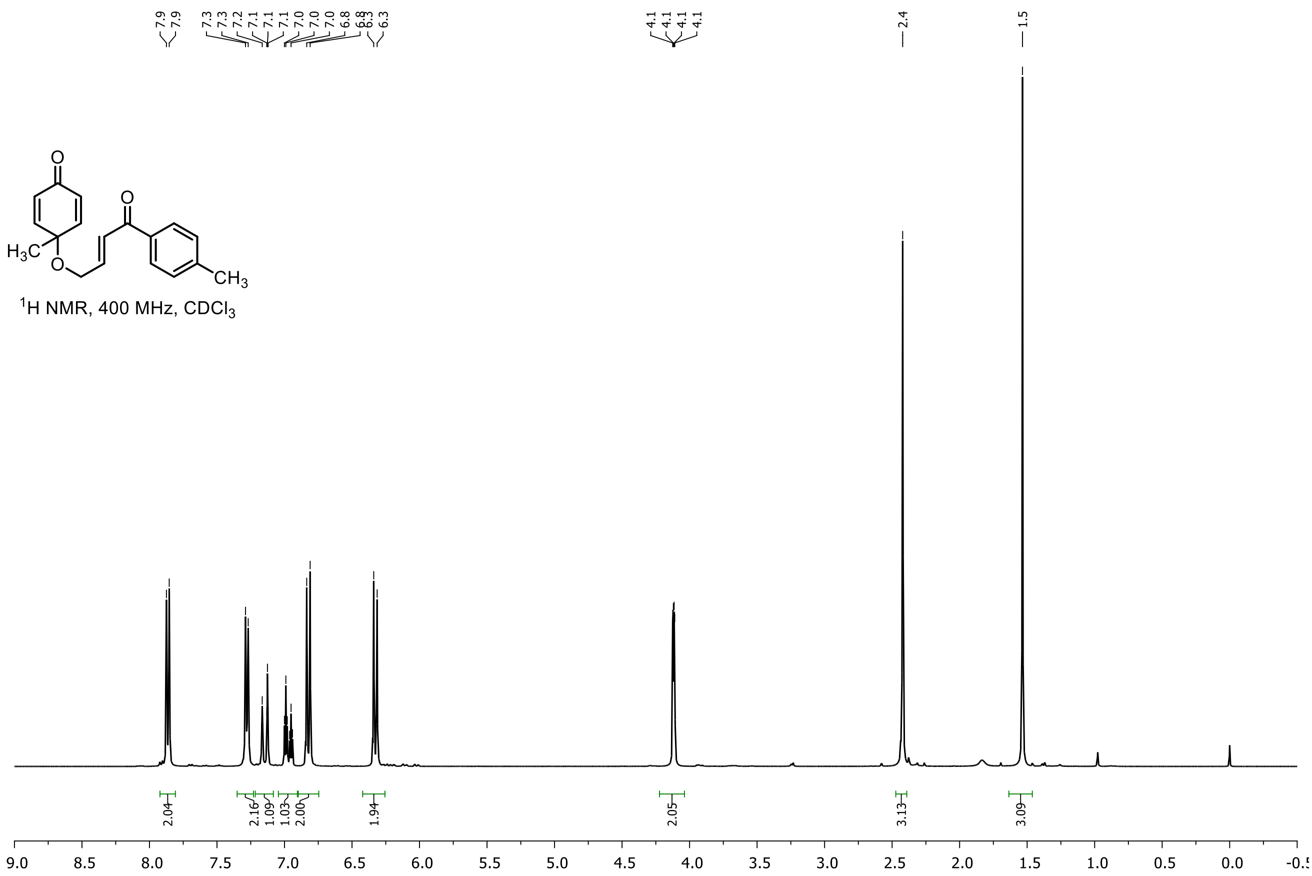

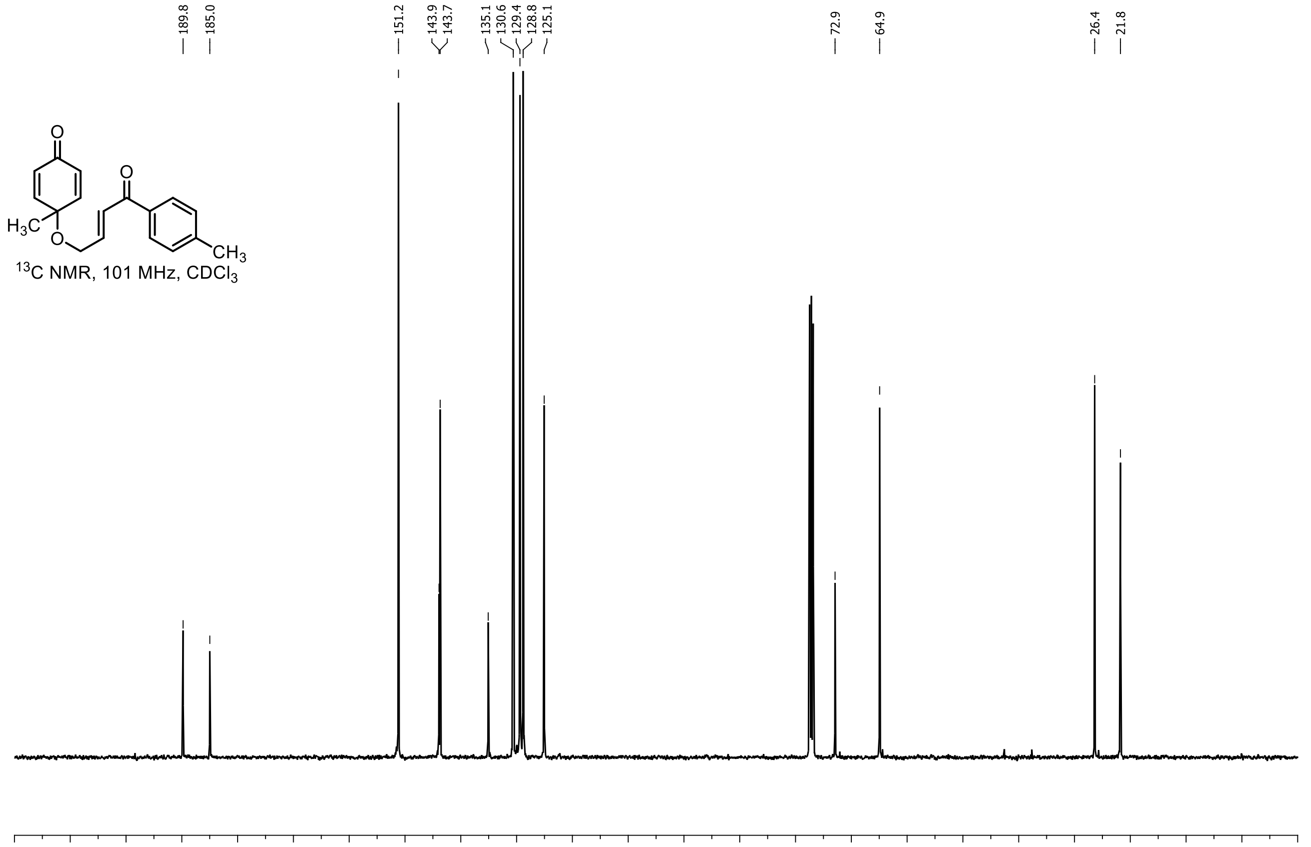

Supplementary Figure 14. ^{1}H NMR and ^{13}C NMR spectra of compound 1r.

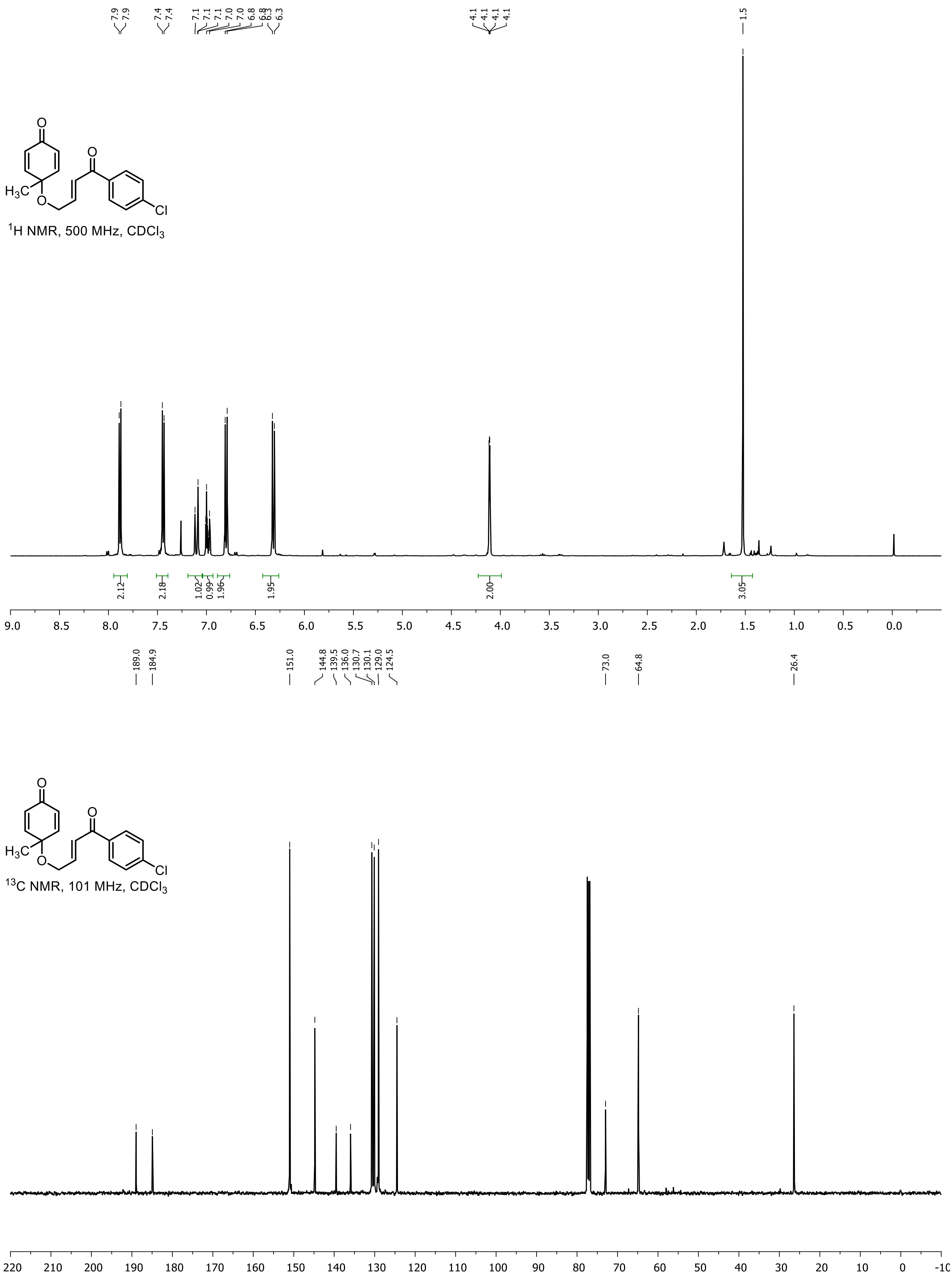

Supplementary Figure 15. ^{1}H NMR and ^{13}C NMR spectra of compound 1u.

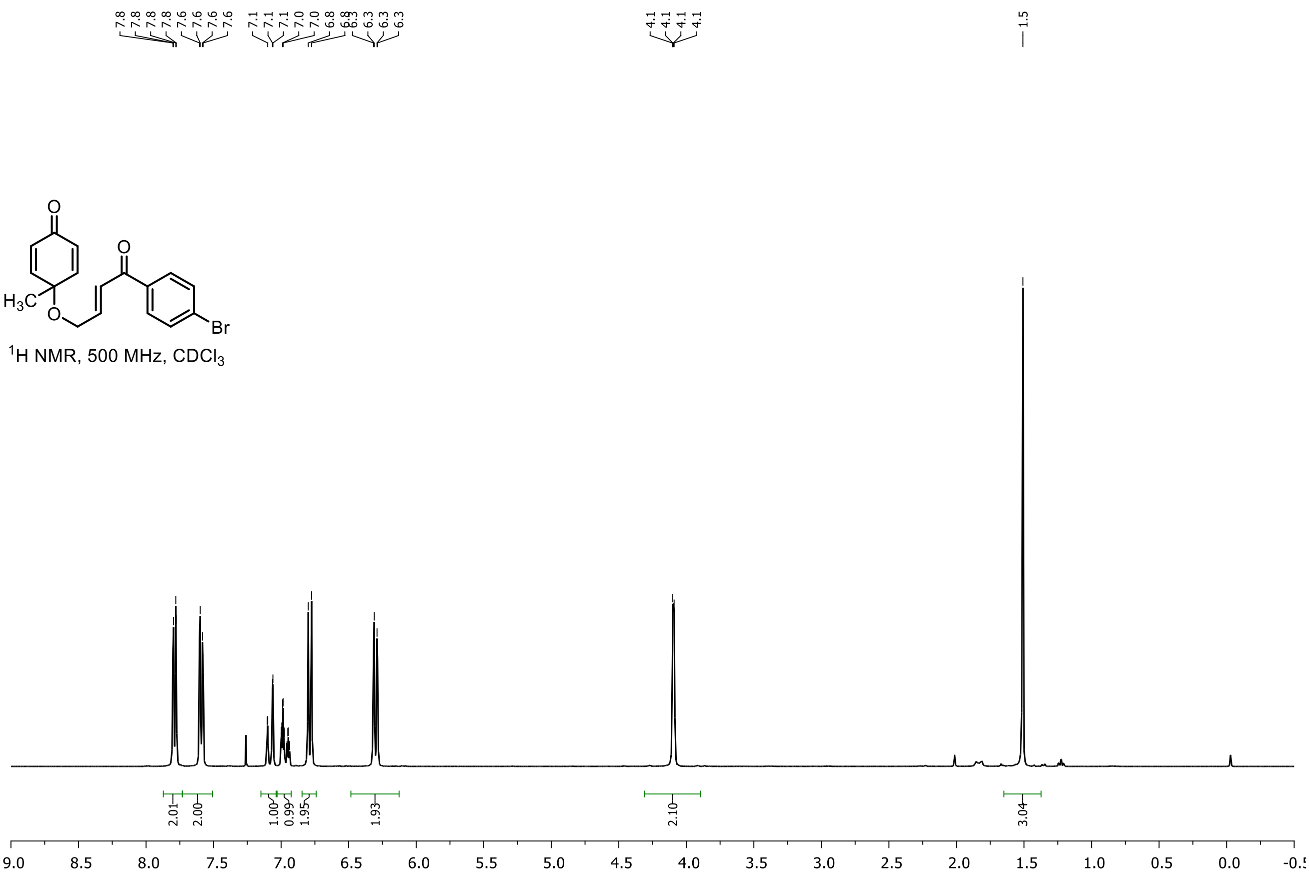

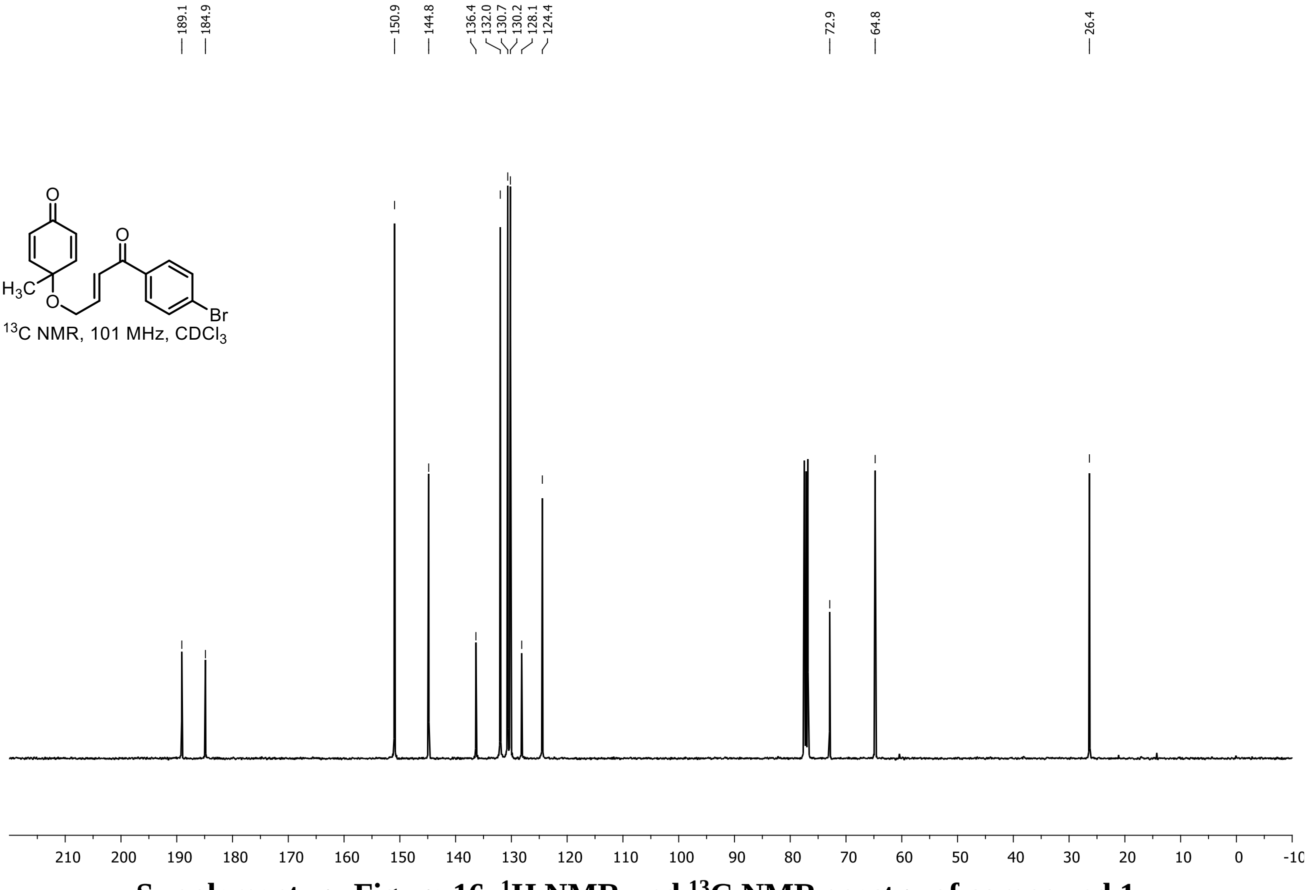

Supplementary Figure 16. ^{1}H NMR and ^{13}C NMR spectra of compound 1v.

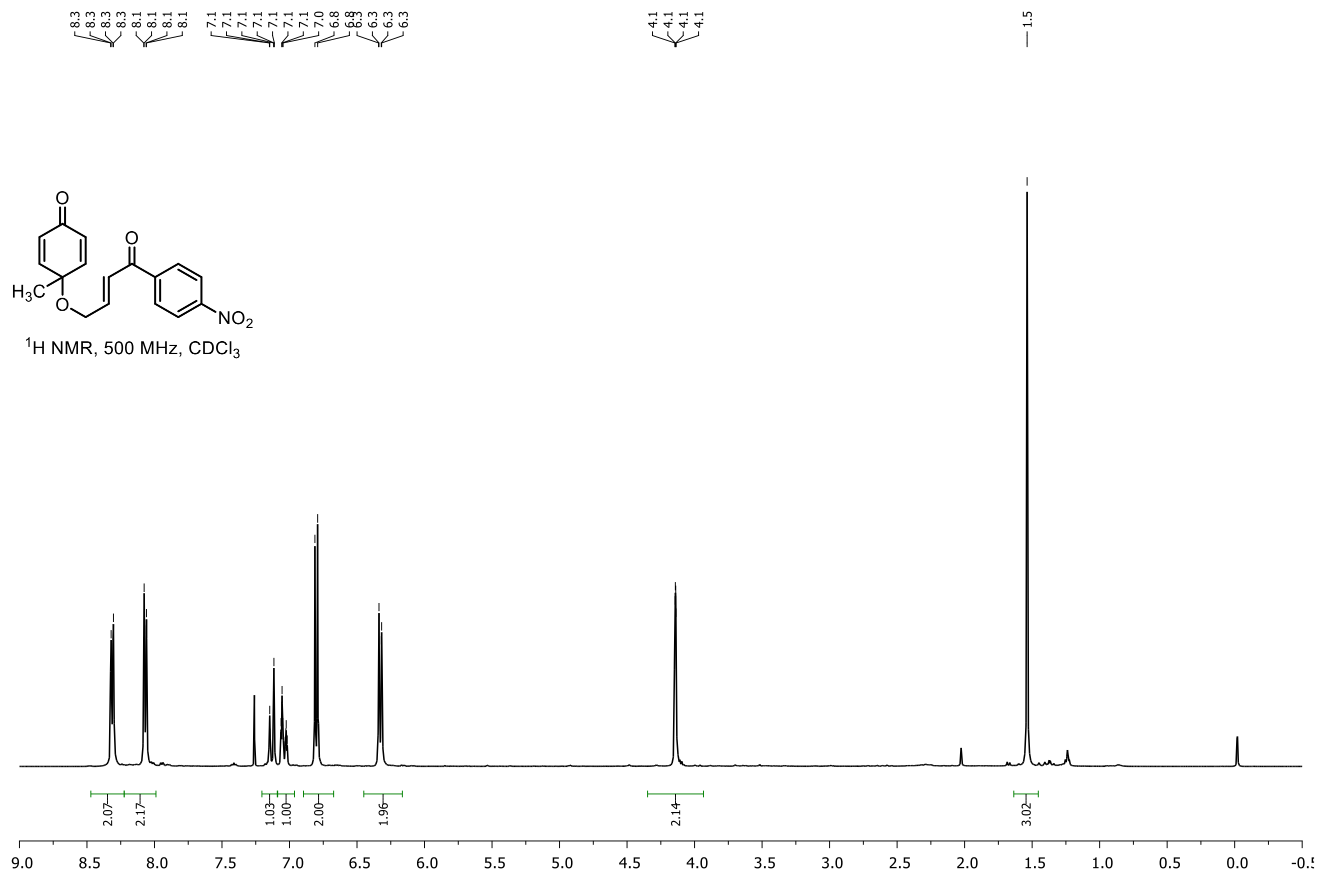

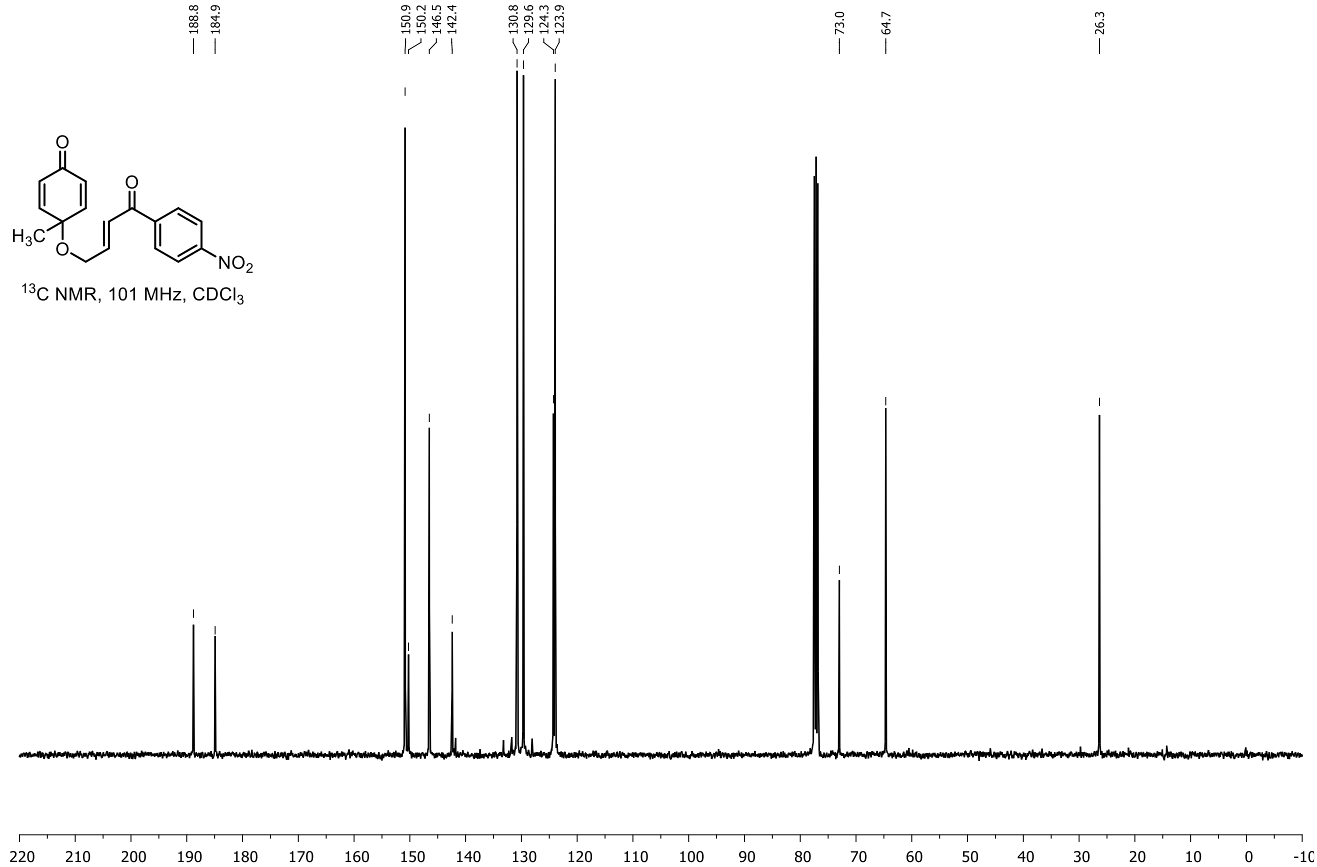

Supplementary Figure 17. **^{1}H NMR and ^{13}C NMR spectra of compound SM-2.**

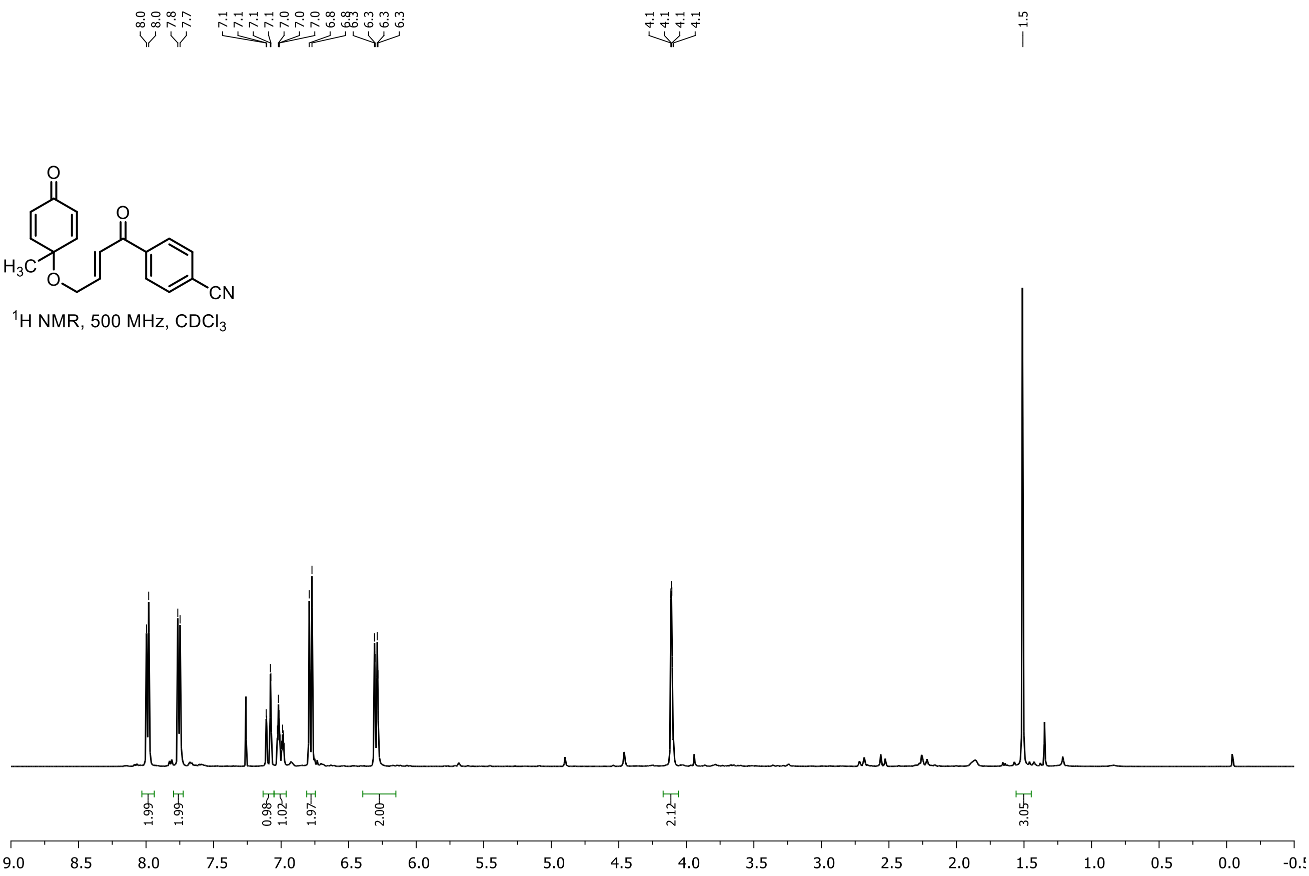

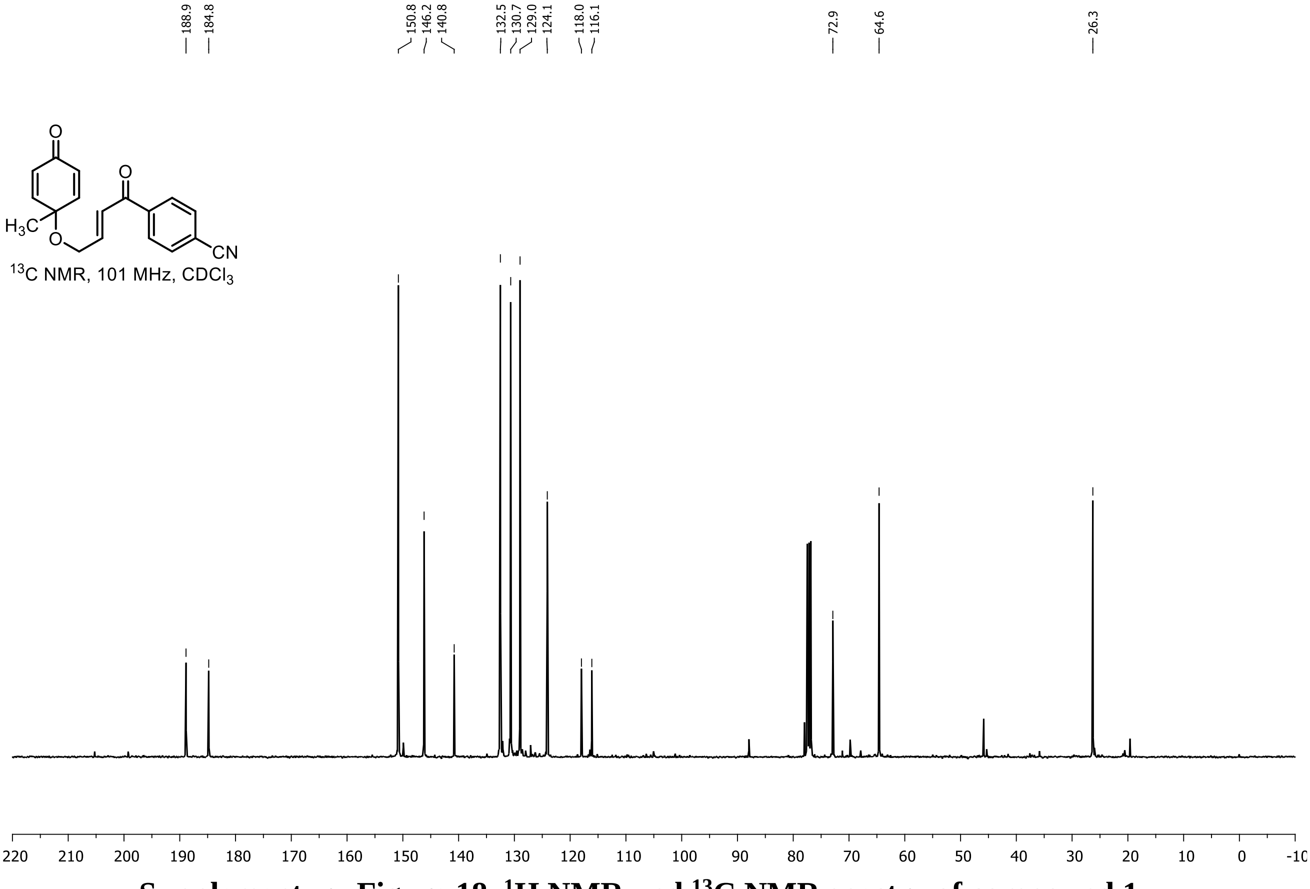

Supplementary Figure 18. ^{1}**H NMR and** ^{13}**C NMR spectra of compound 1x.**

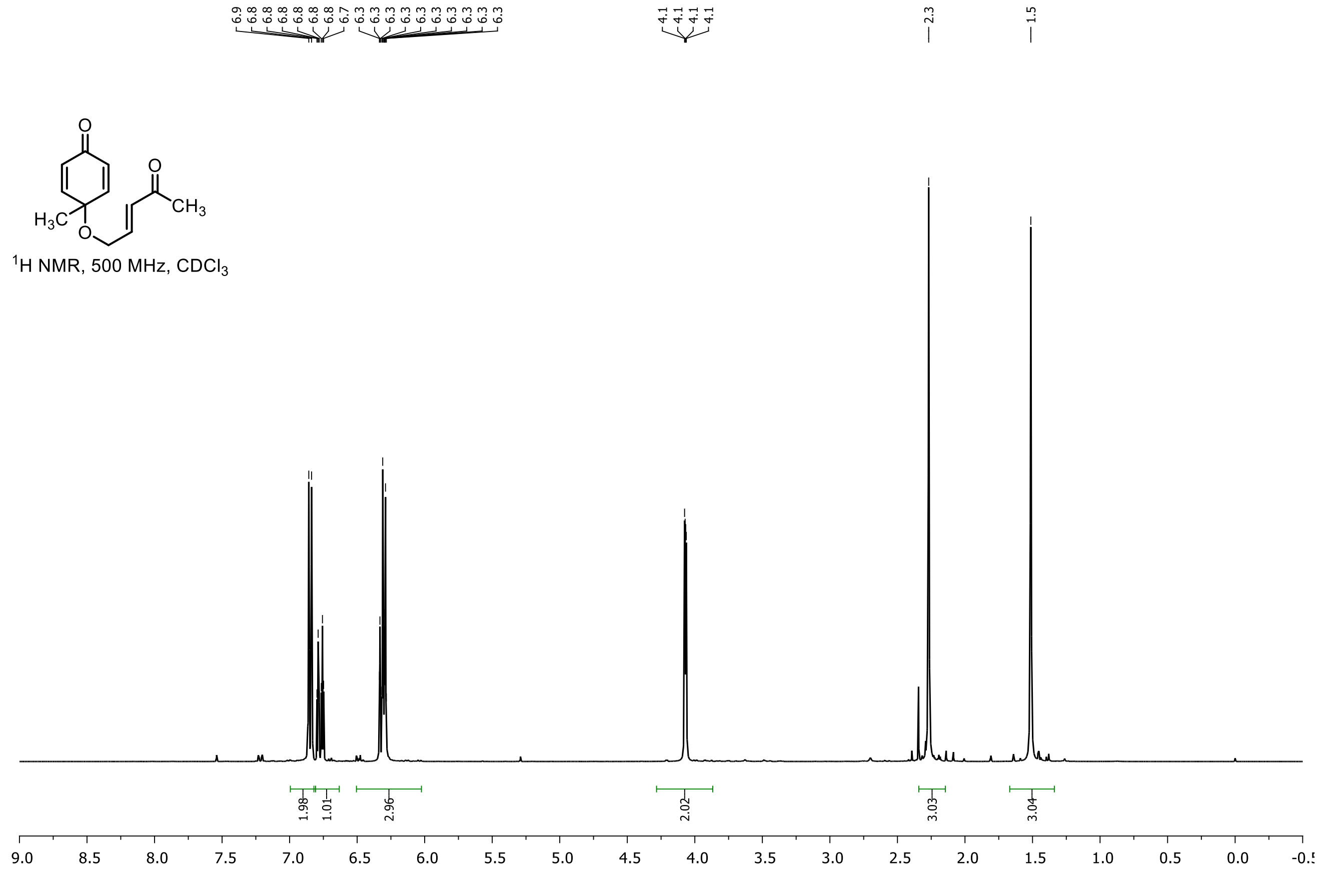

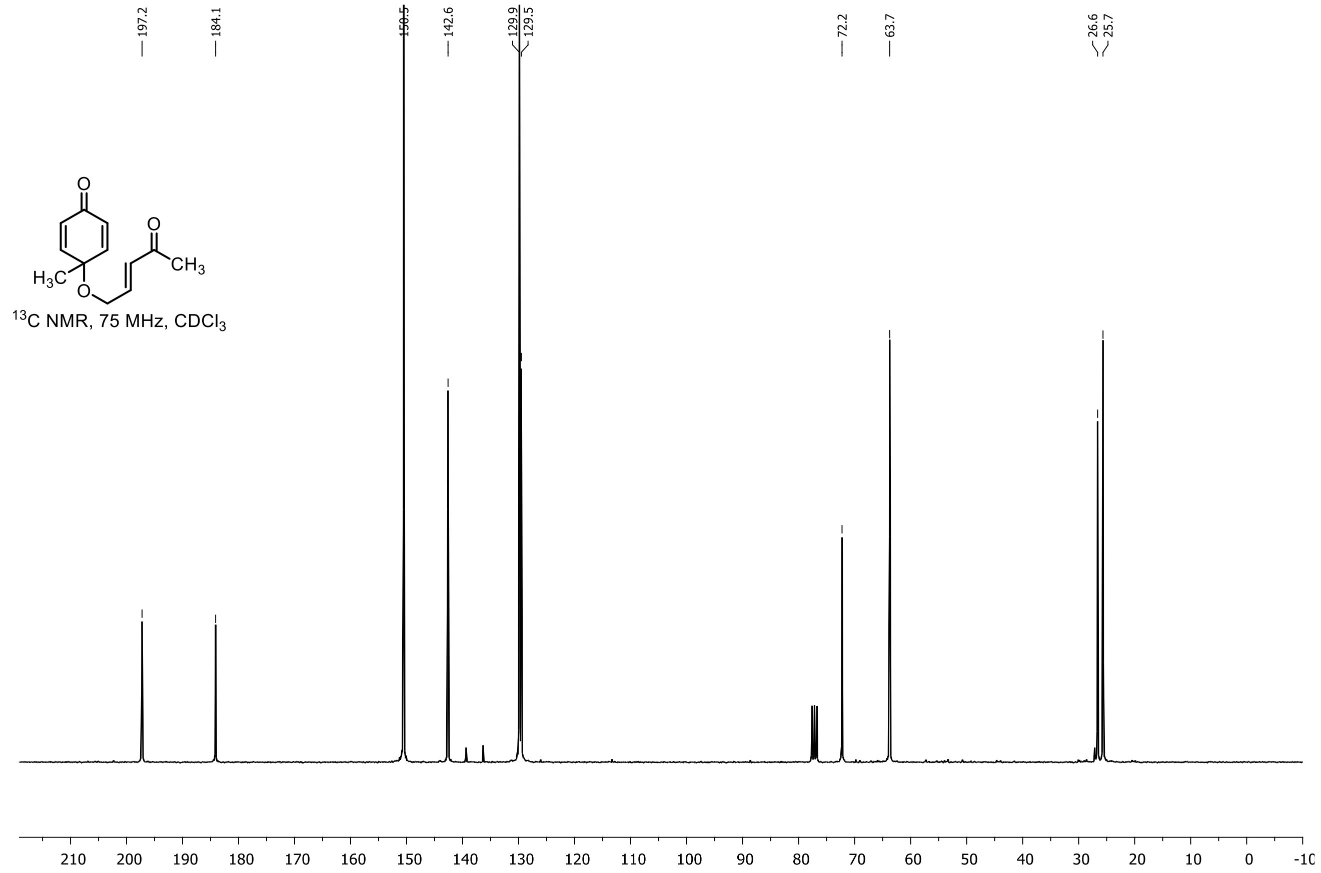

Supplementary Figure 19. **^{1}H NMR and ^{13}C NMR spectra of compound 1za.**

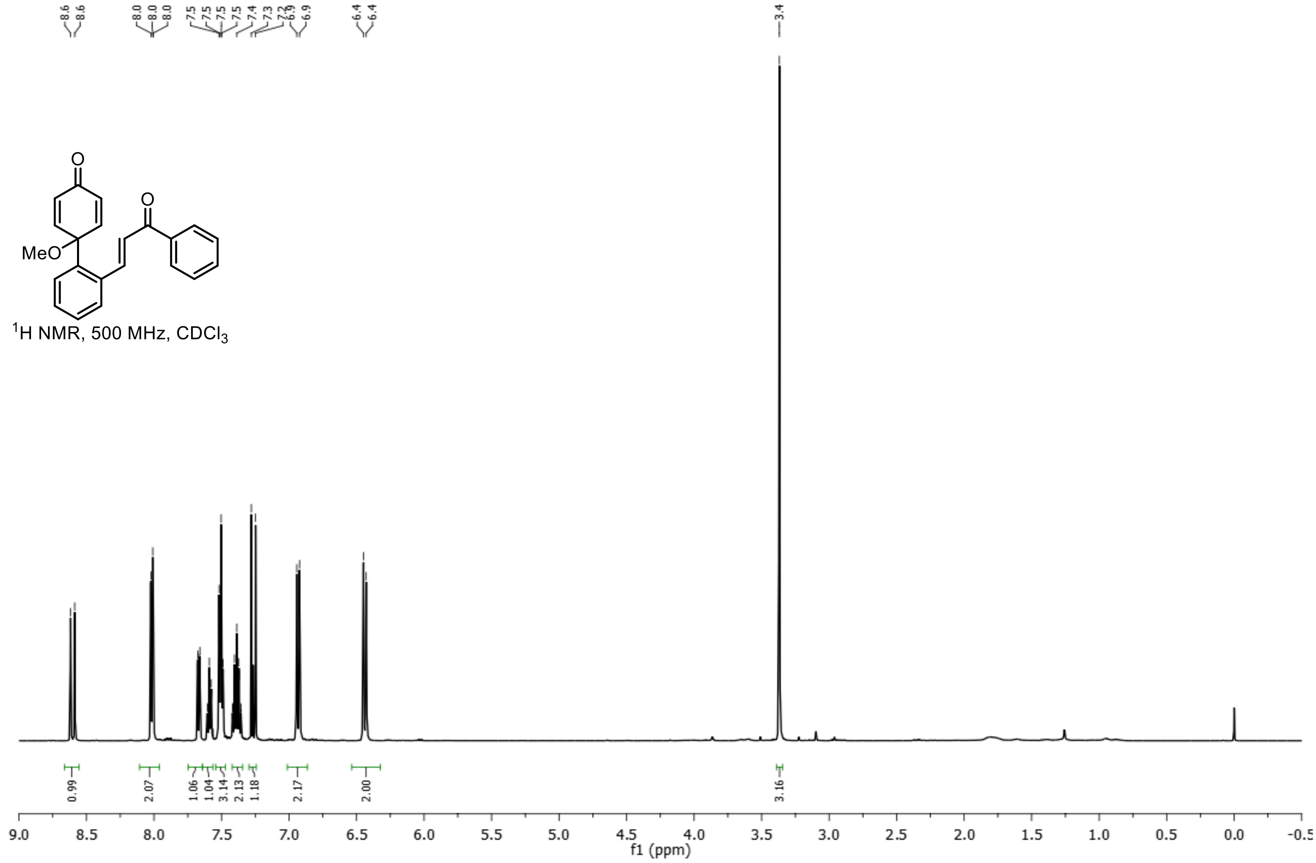

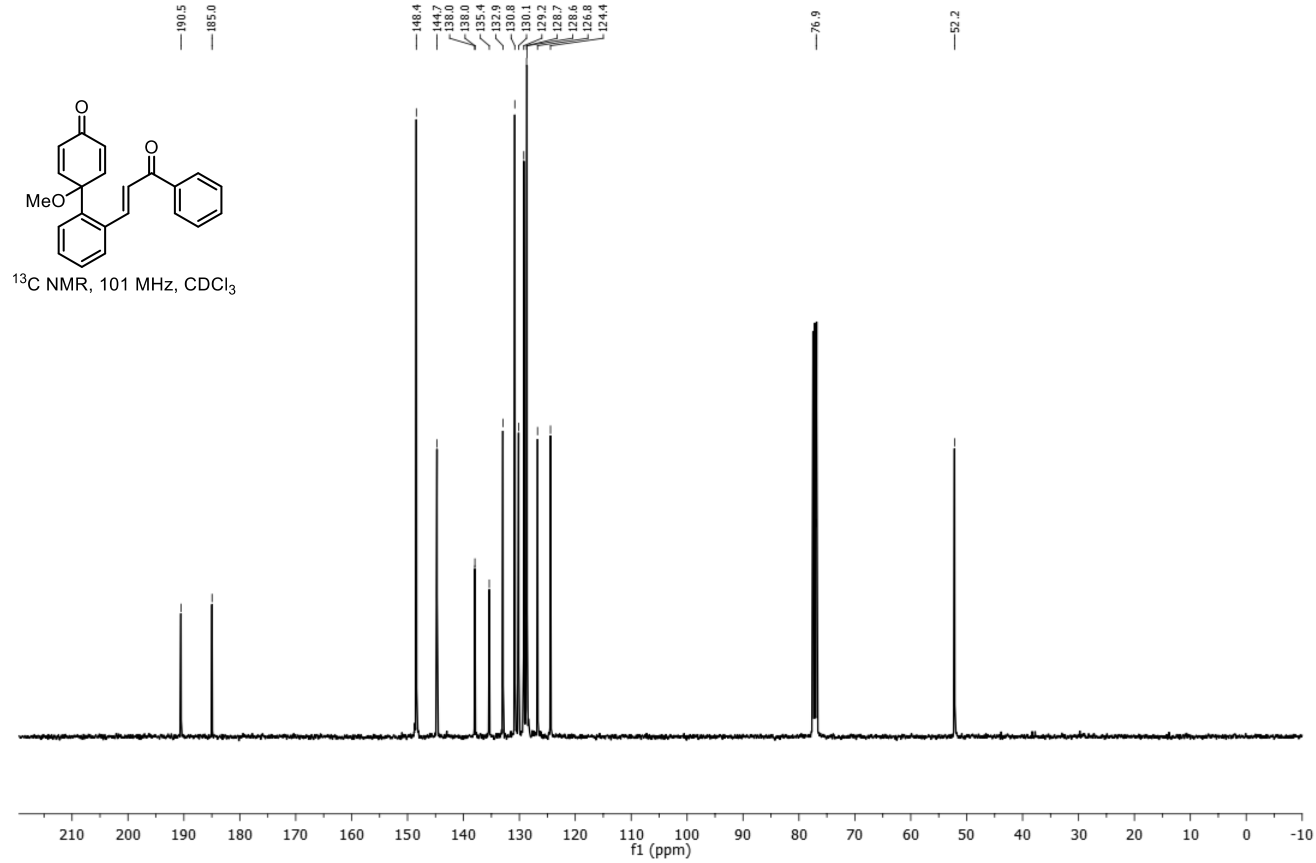

Supplementary Figure 20. **^{1}H NMR and ^{13}C NMR spectra of compound SM-3.**

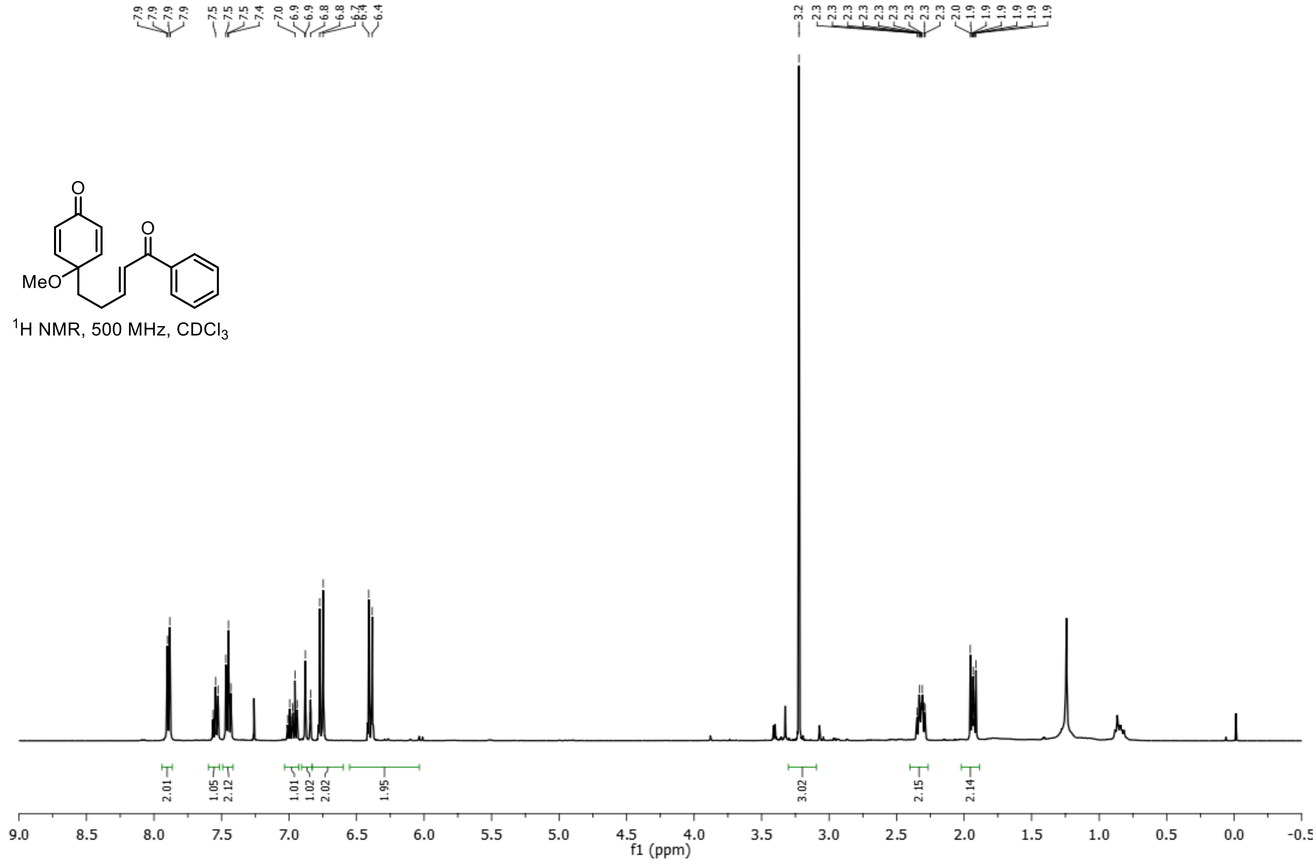

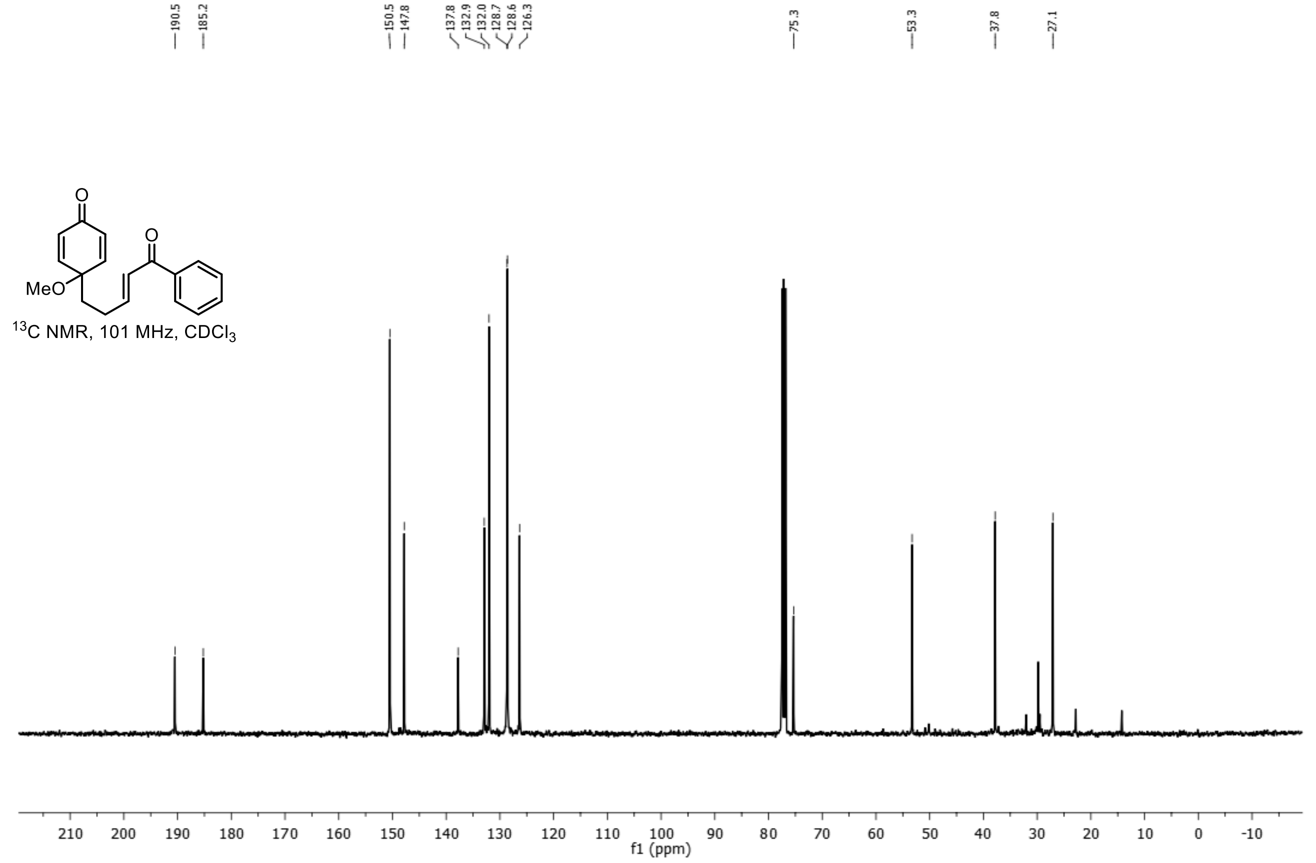

Supplementary Figure 21. **^{1}H NMR and ^{13}C NMR spectra of compound 4.**

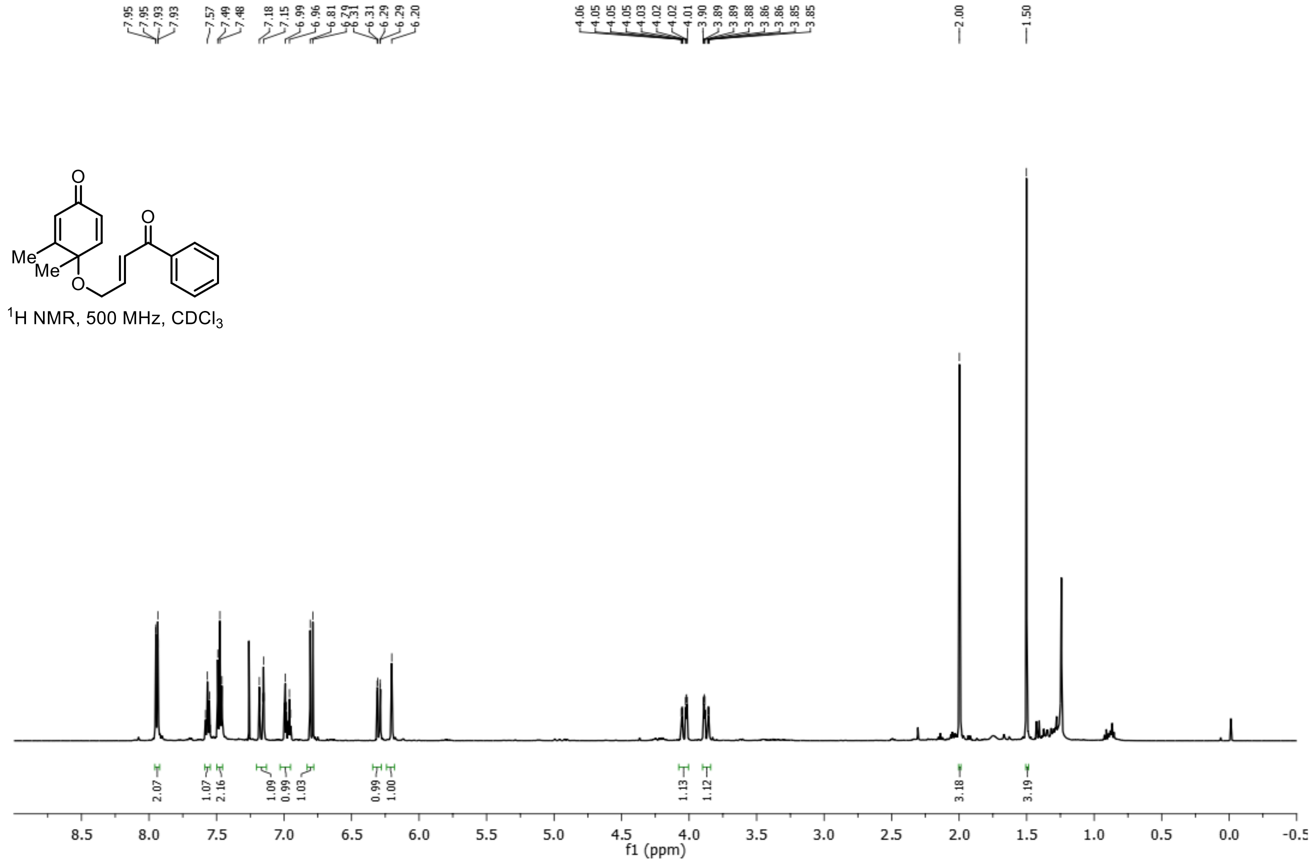

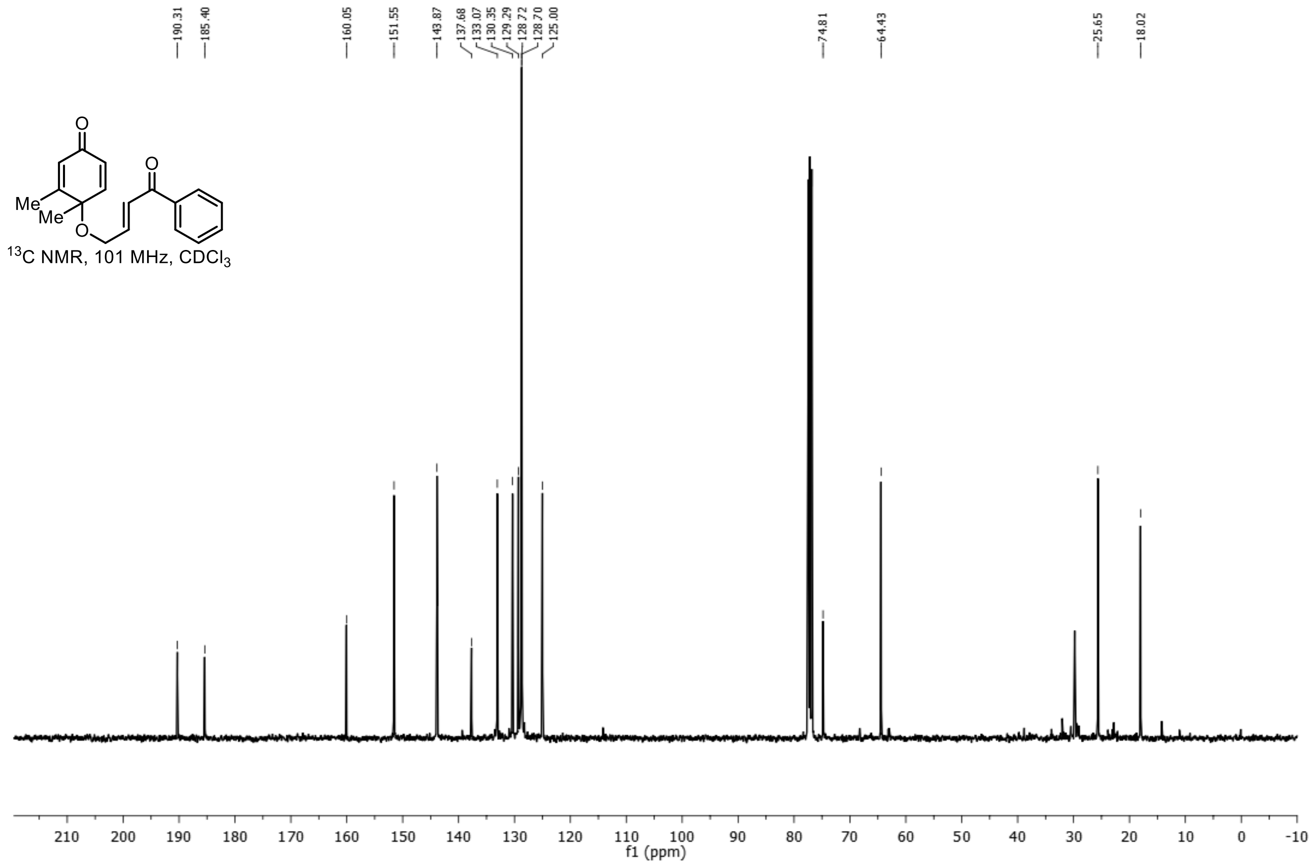

Supplementary Figure 22. ^{1}H NMR and ^{13}C NMR spectra of compound 7.

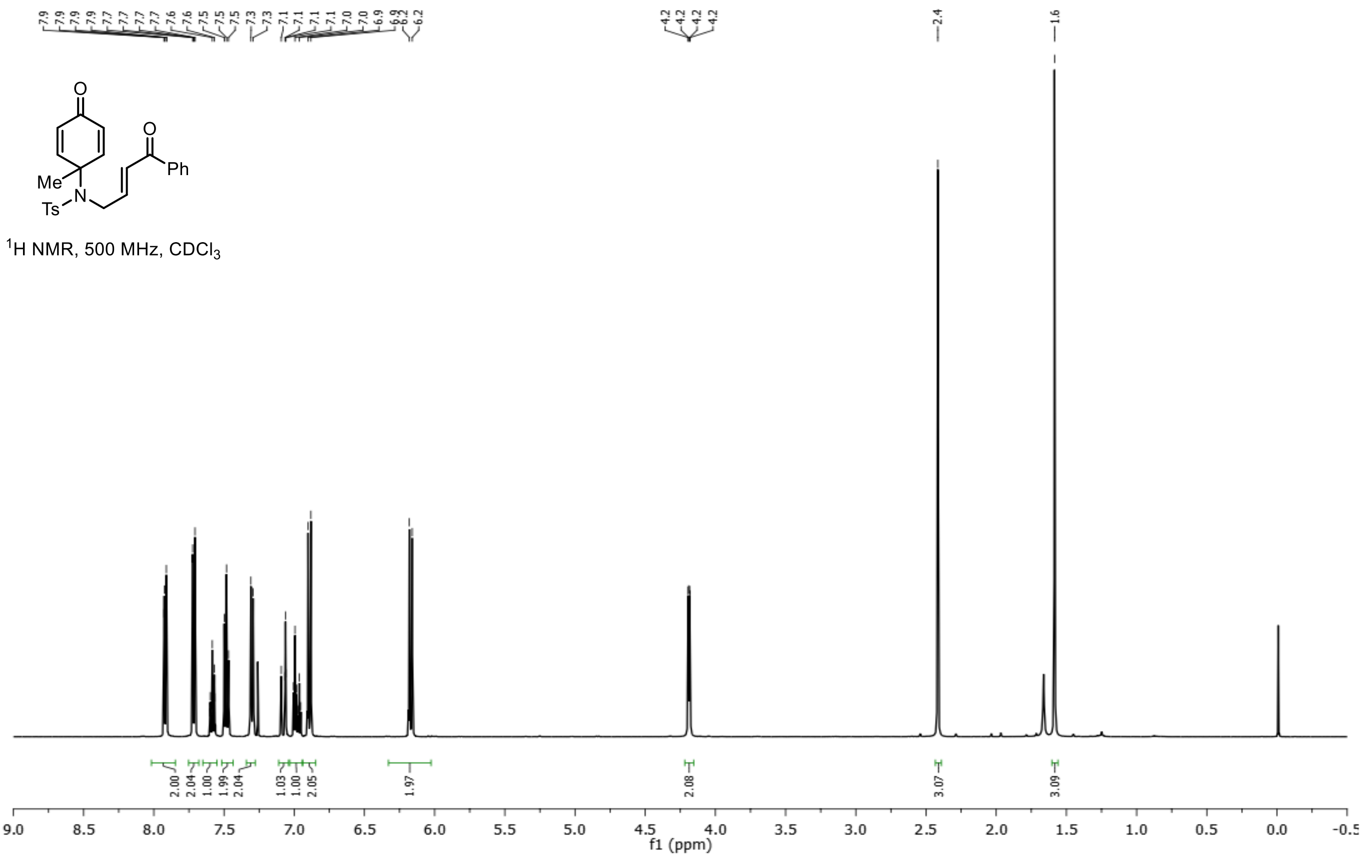

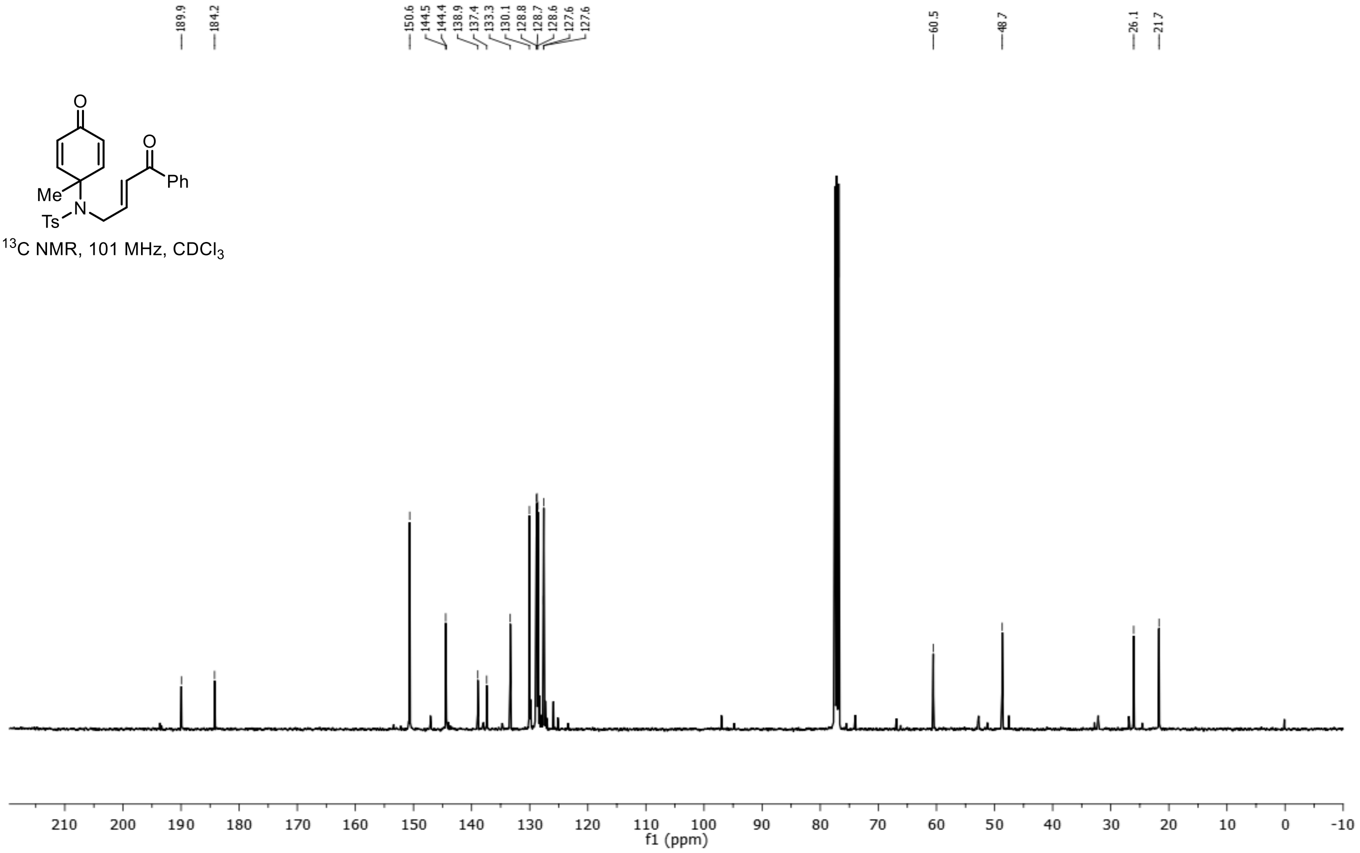

Supplementary Figure 23. ^{1}H NMR and ^{13}C NMR spectra of compound 1o′.

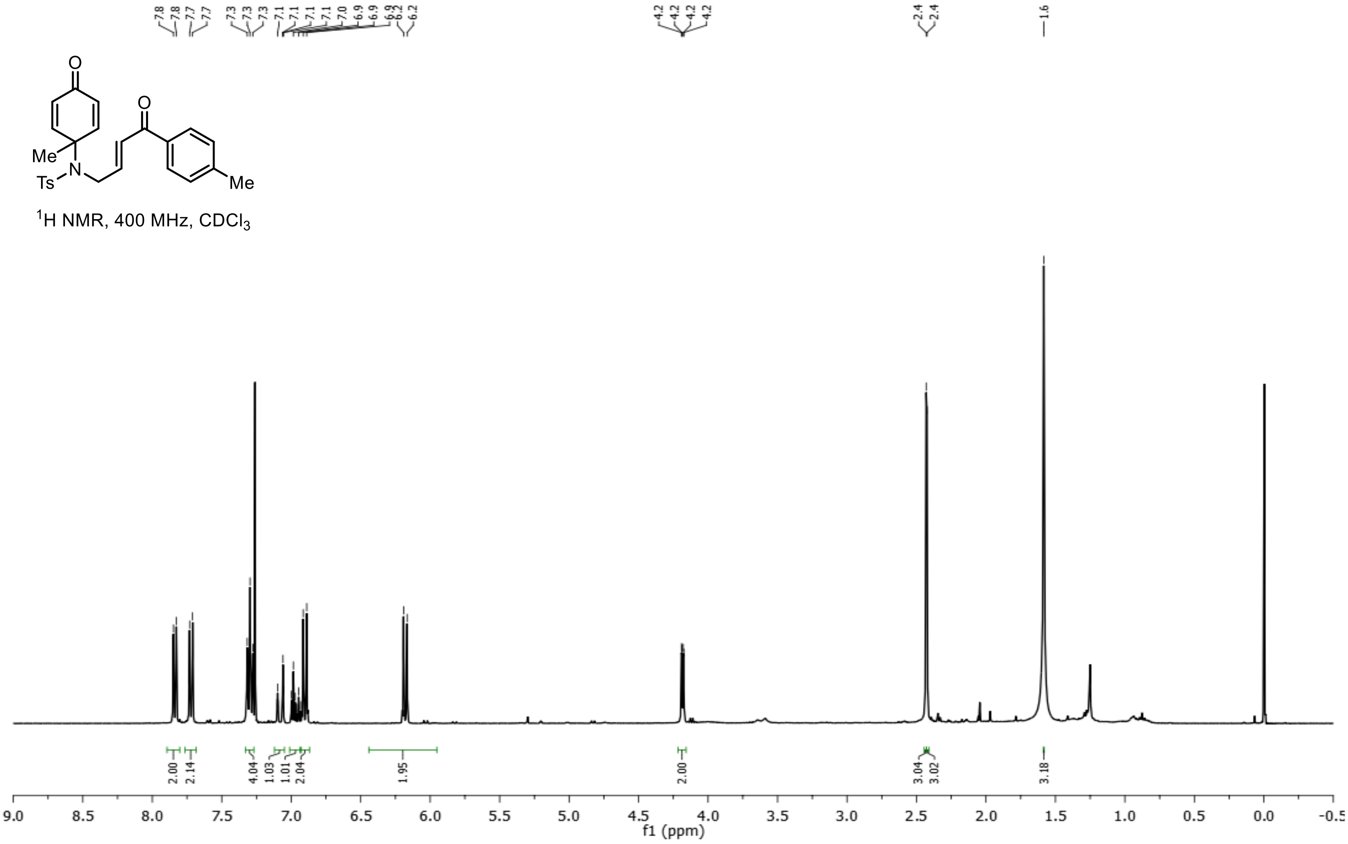

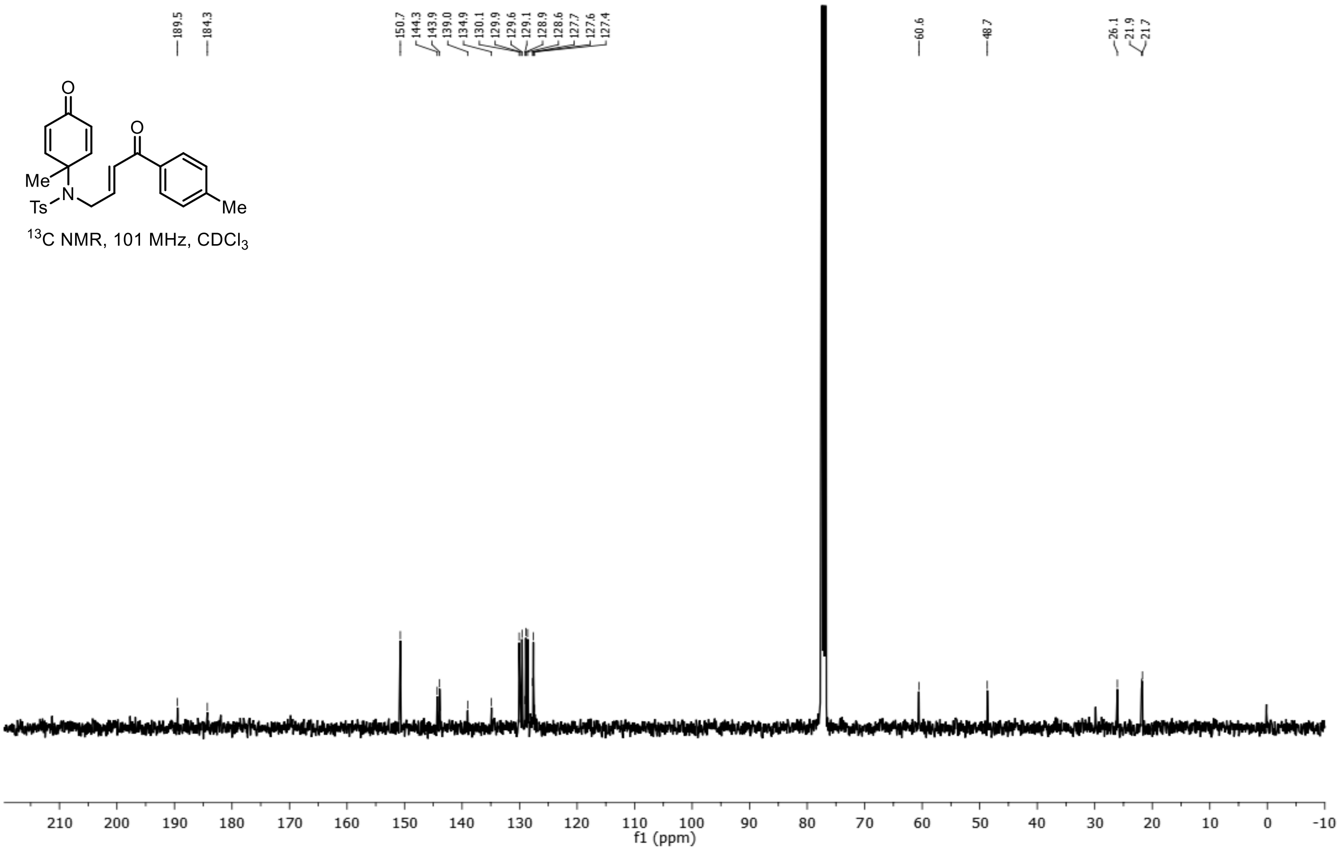

Supplementary Figure 24. **^{1}H NMR and ^{13}C NMR spectra of compound 1p′.**

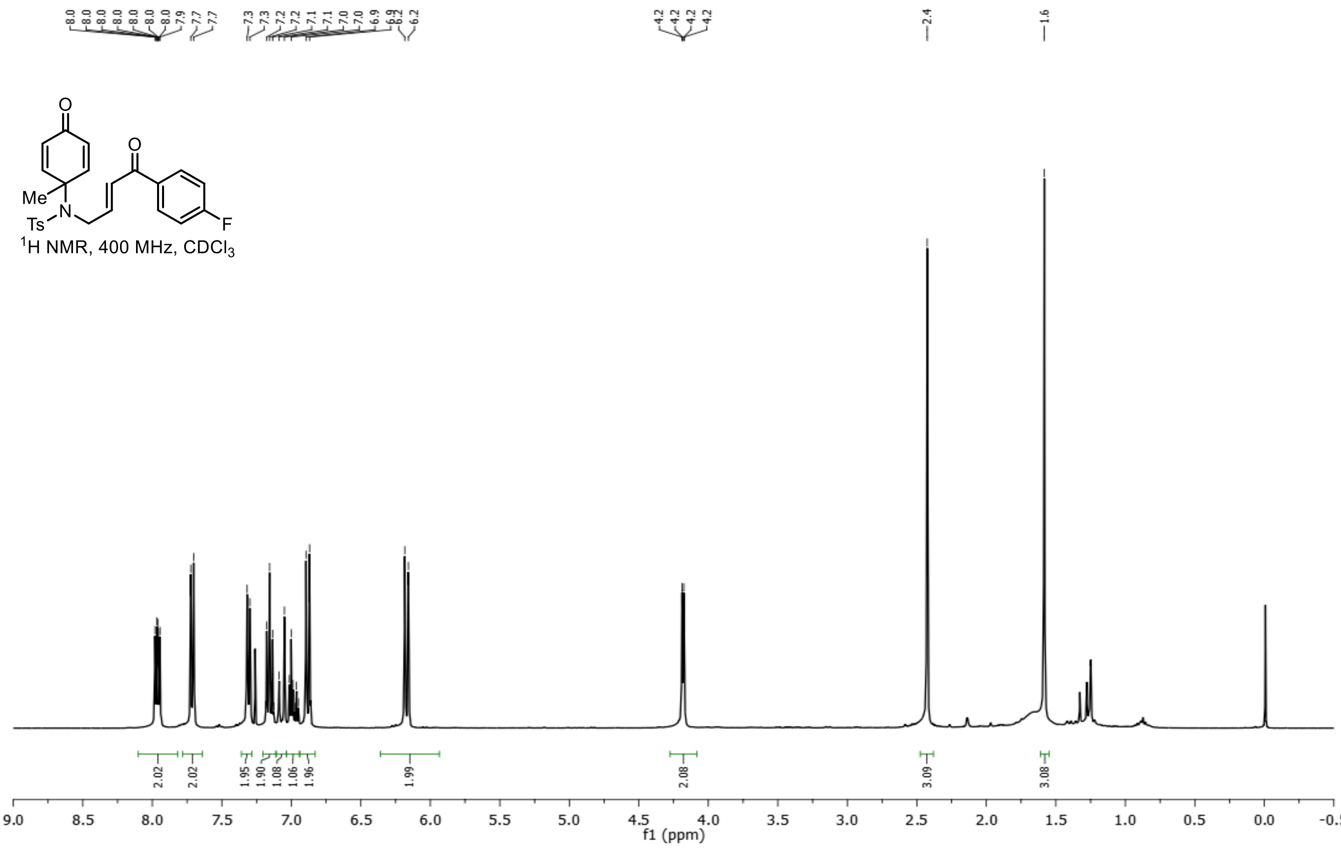

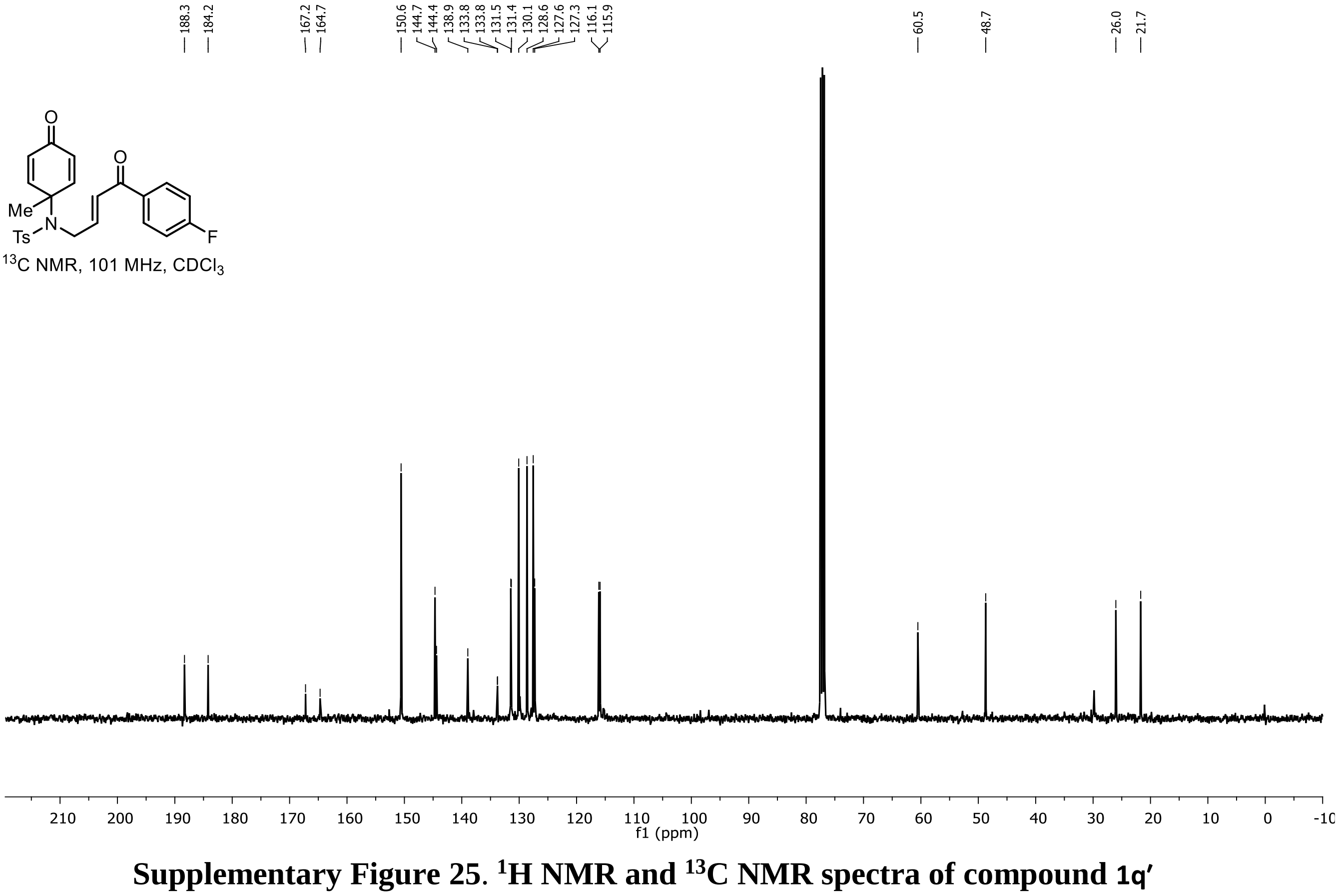

Supplementary Figure 25. ^{1}H NMR and ^{13}C NMR spectra of compound 1q′

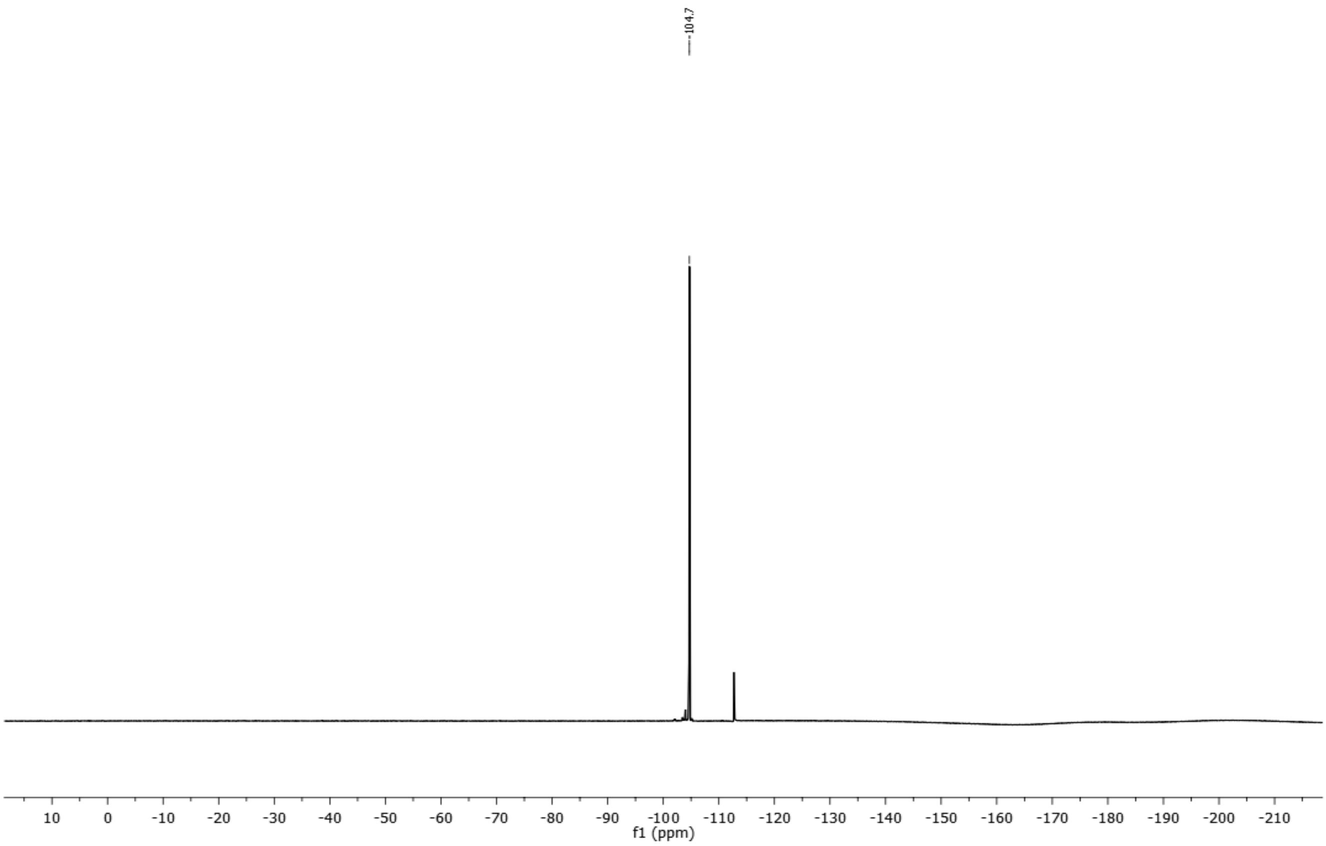

Supplementary Figure 26. **^{19}F NMR spectra of compound 1q′.**

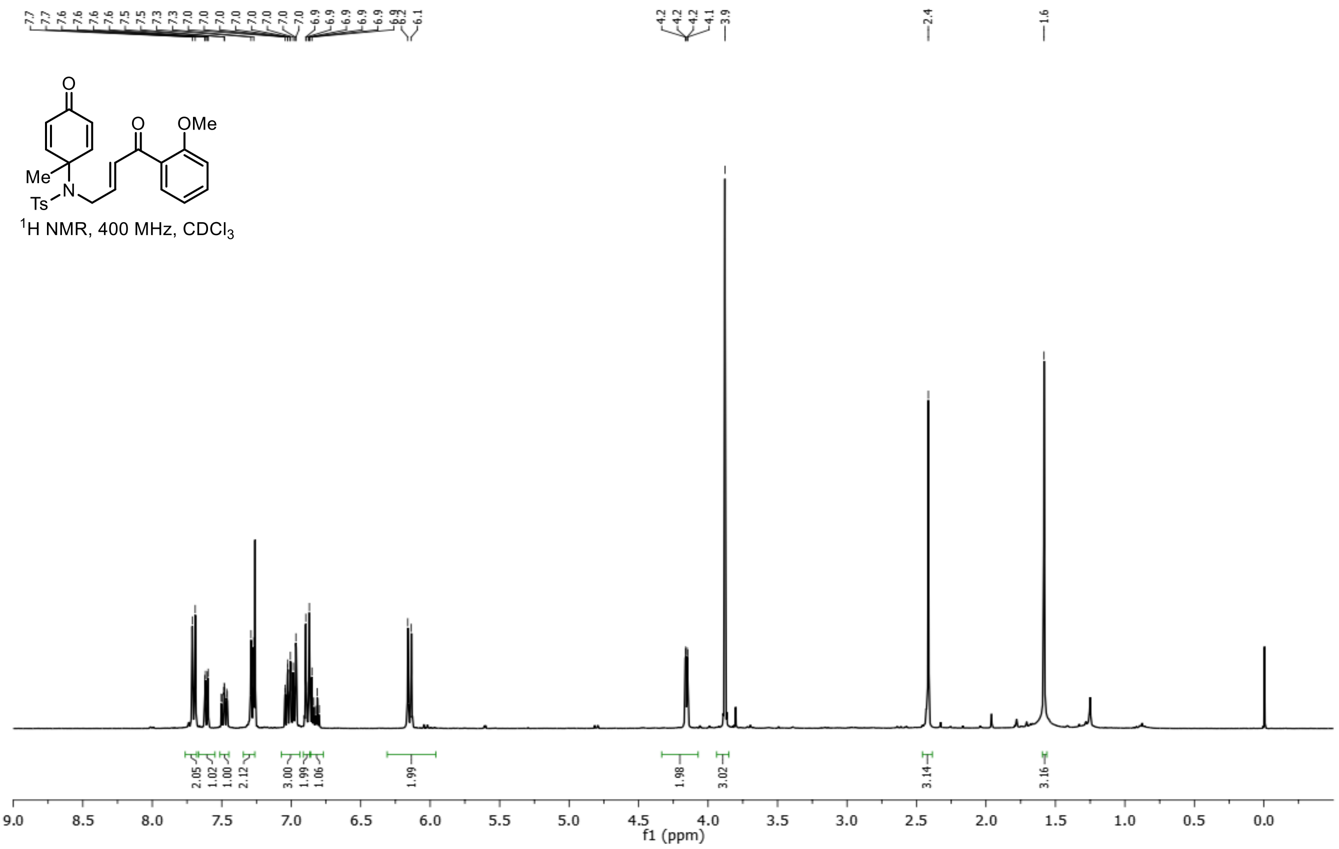

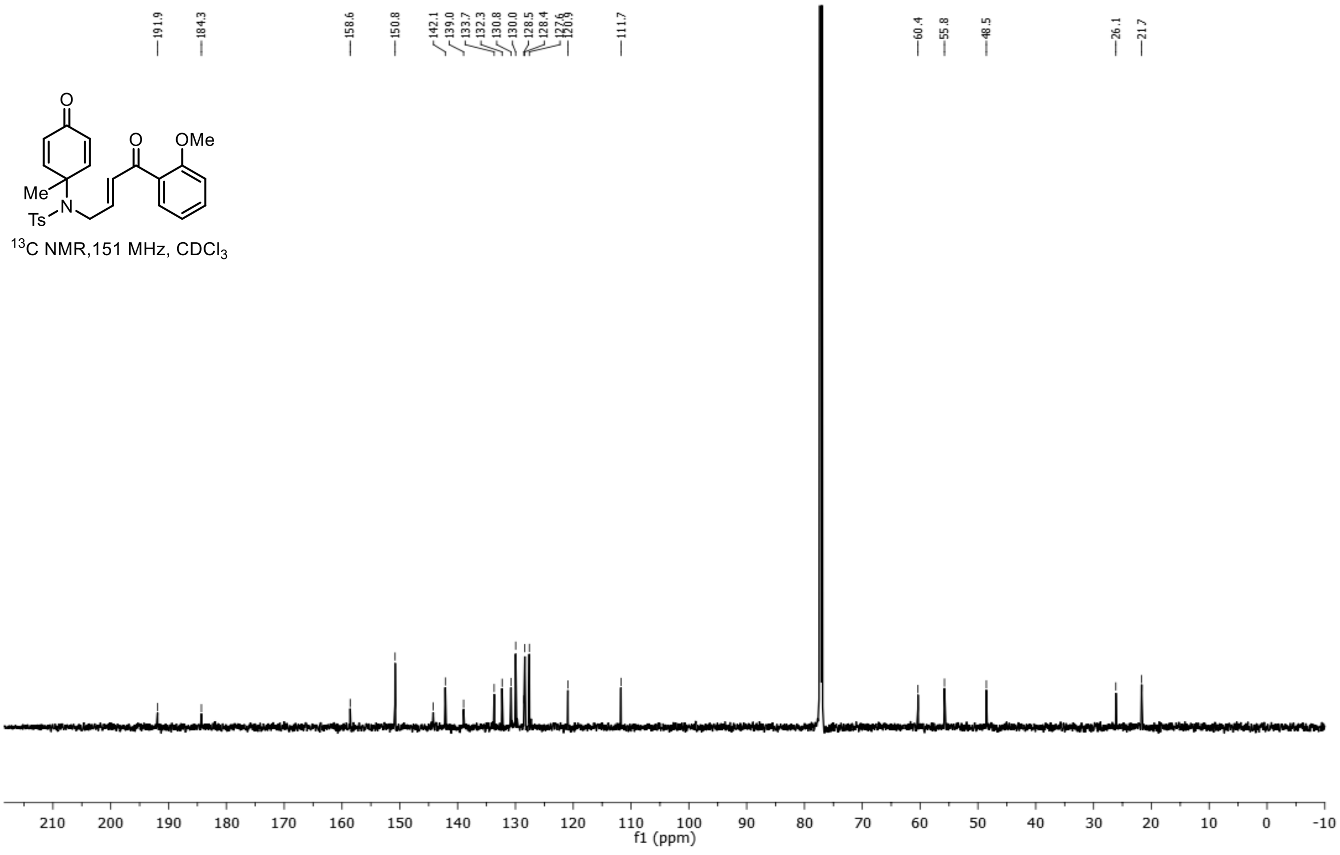

Supplementary Figure 27. ^{1}H NMR and ^{13}C NMR spectra of compound 1r′

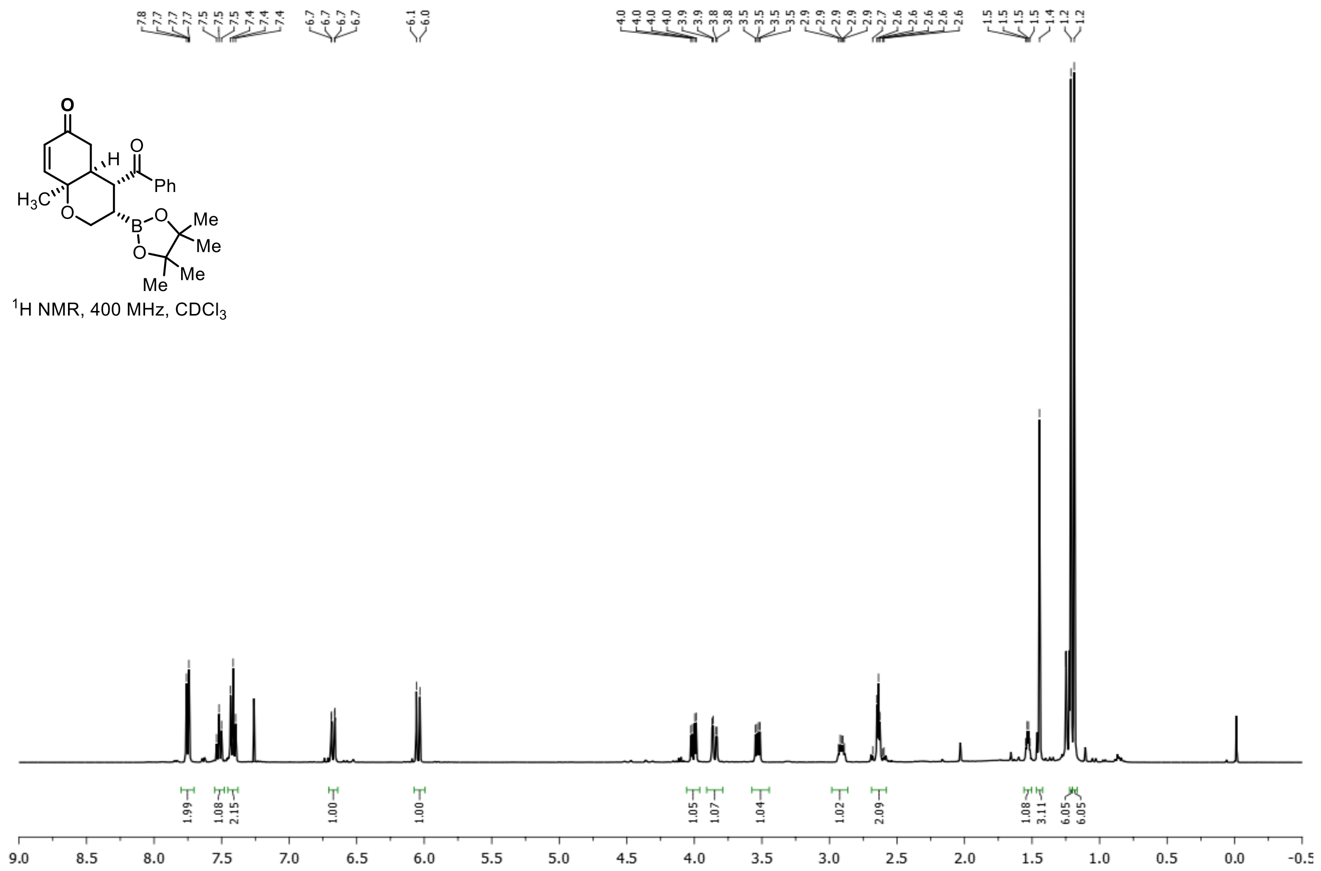

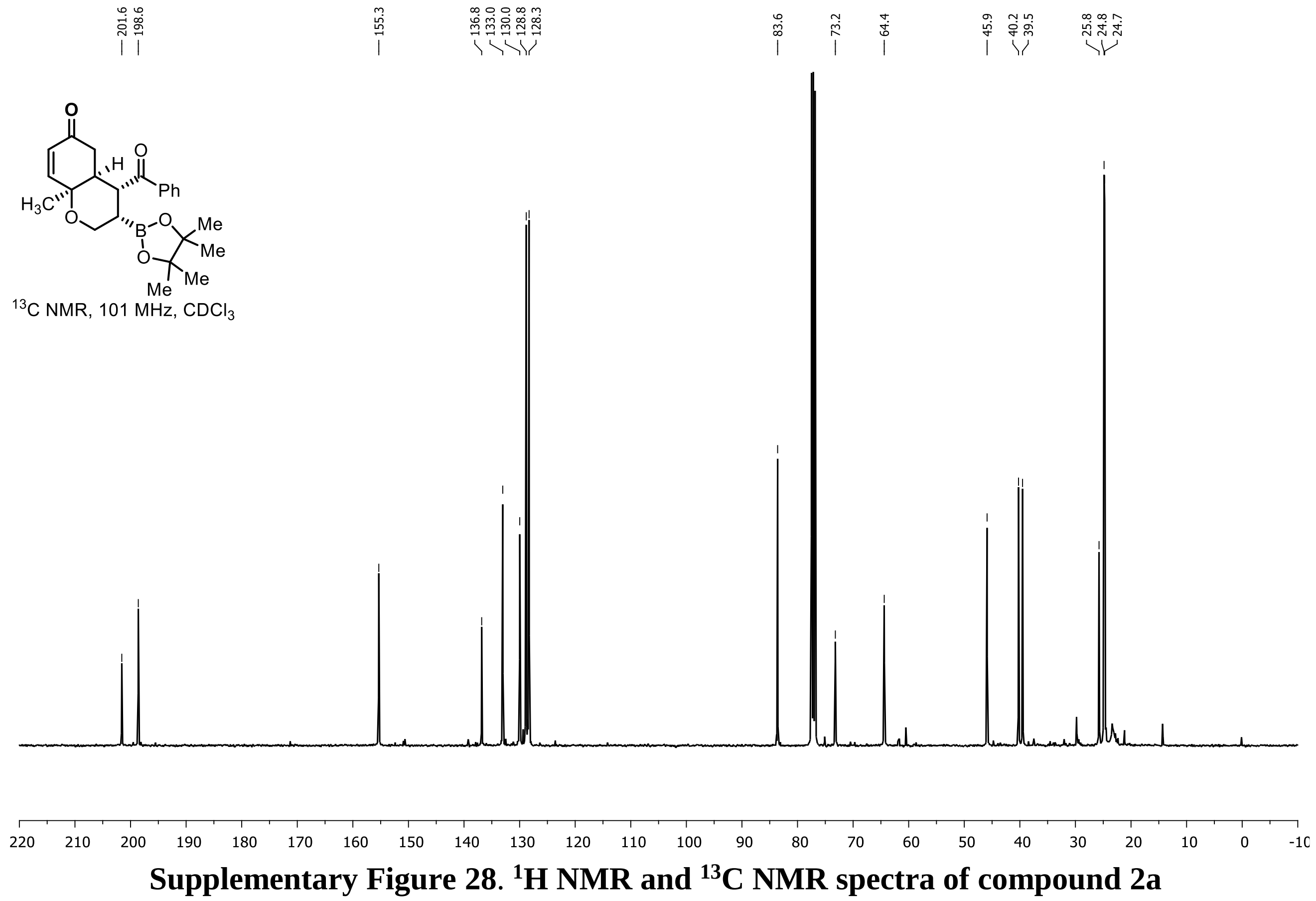

Supplementary Figure 28. **^{1}H NMR and ^{13}C NMR spectra of compound 2a**

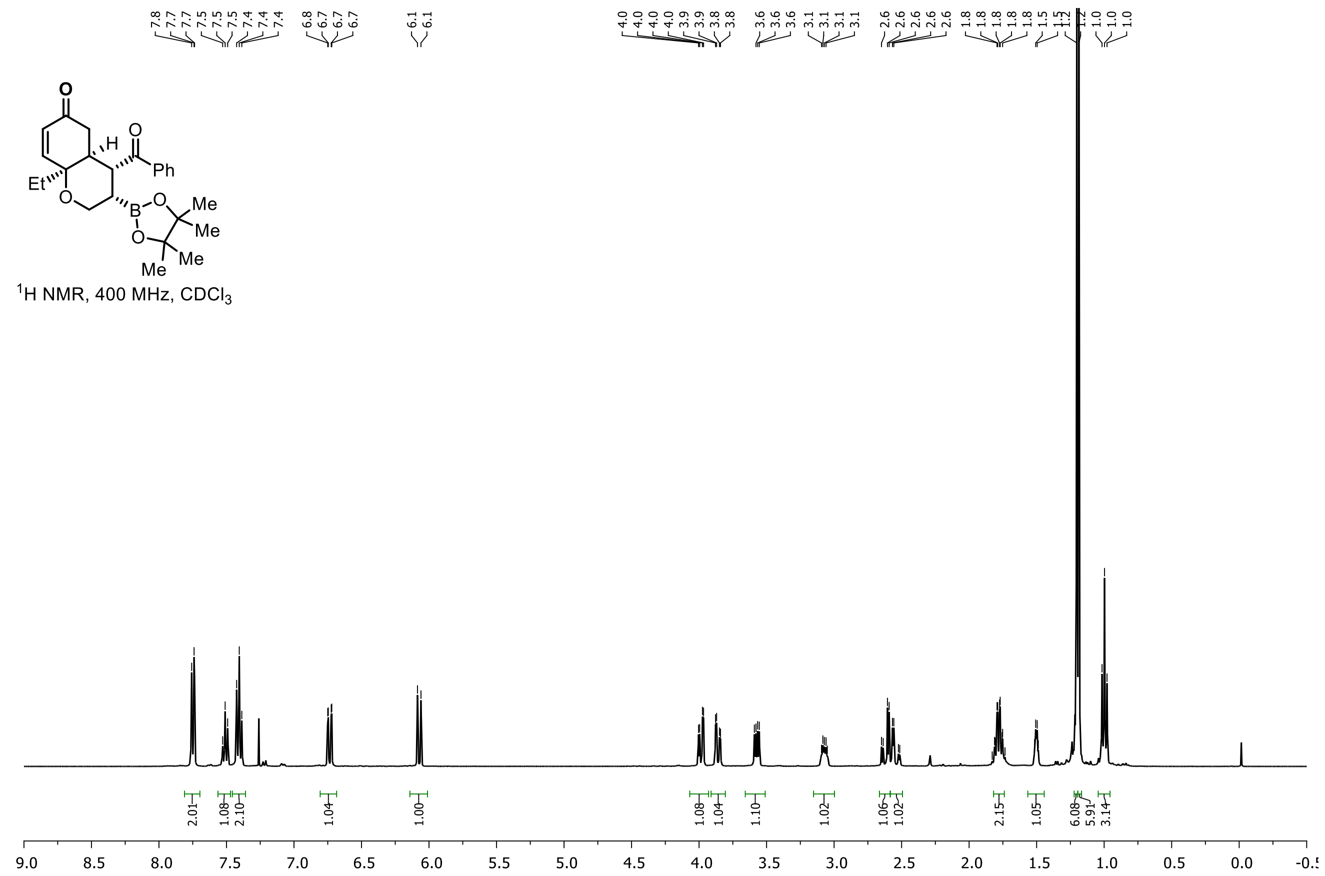

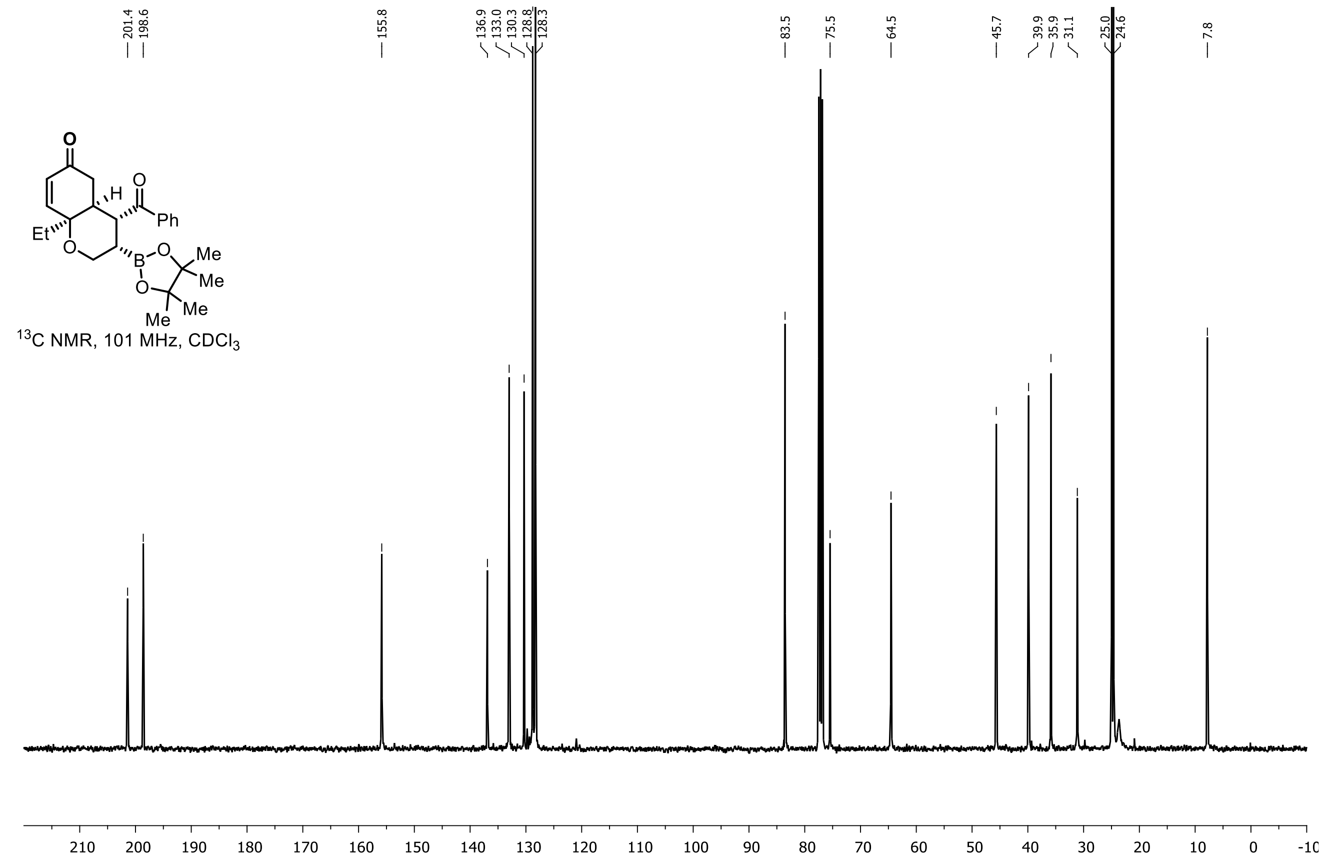

Supplementary Figure 29. **^{1}H NMR and ^{13}C NMR spectra of compound 2b**

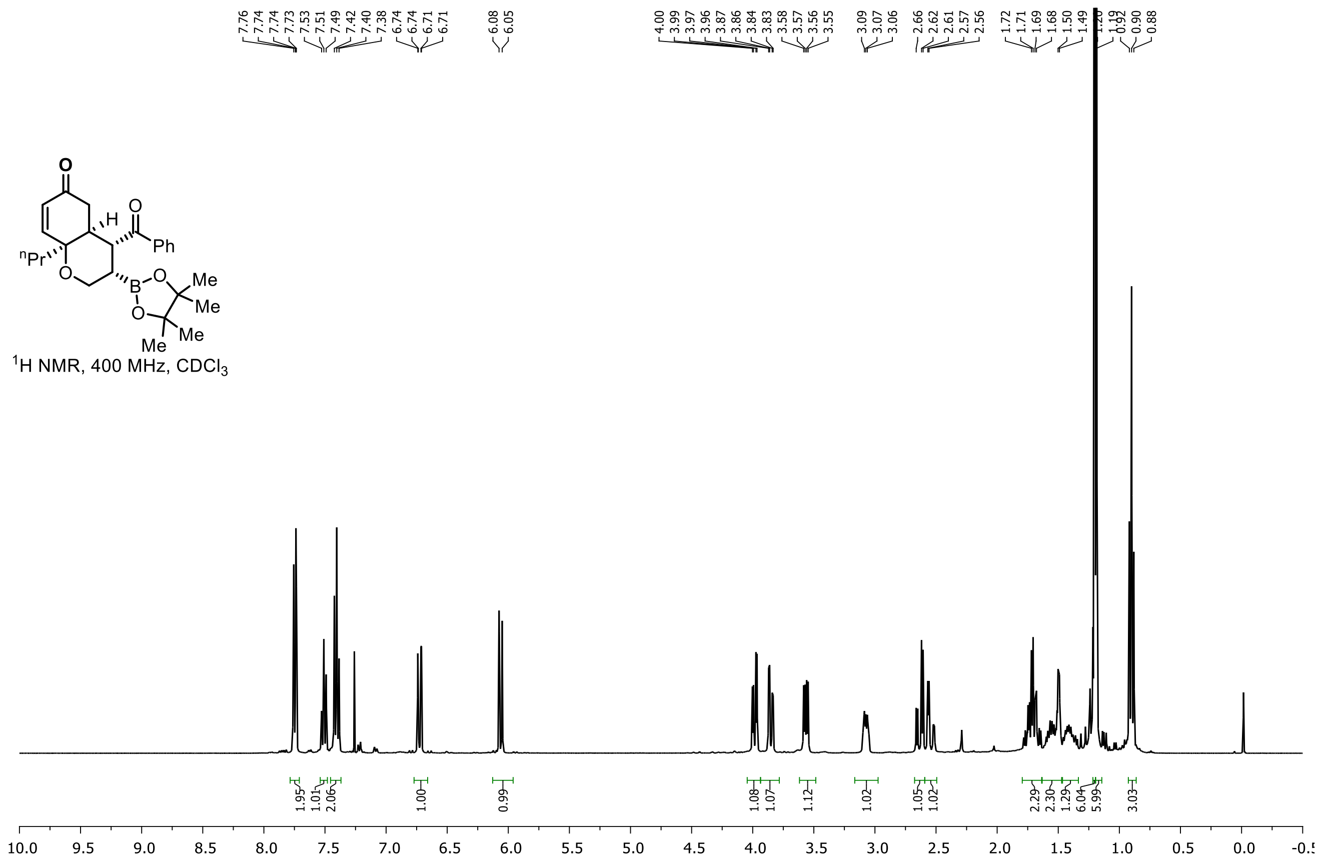

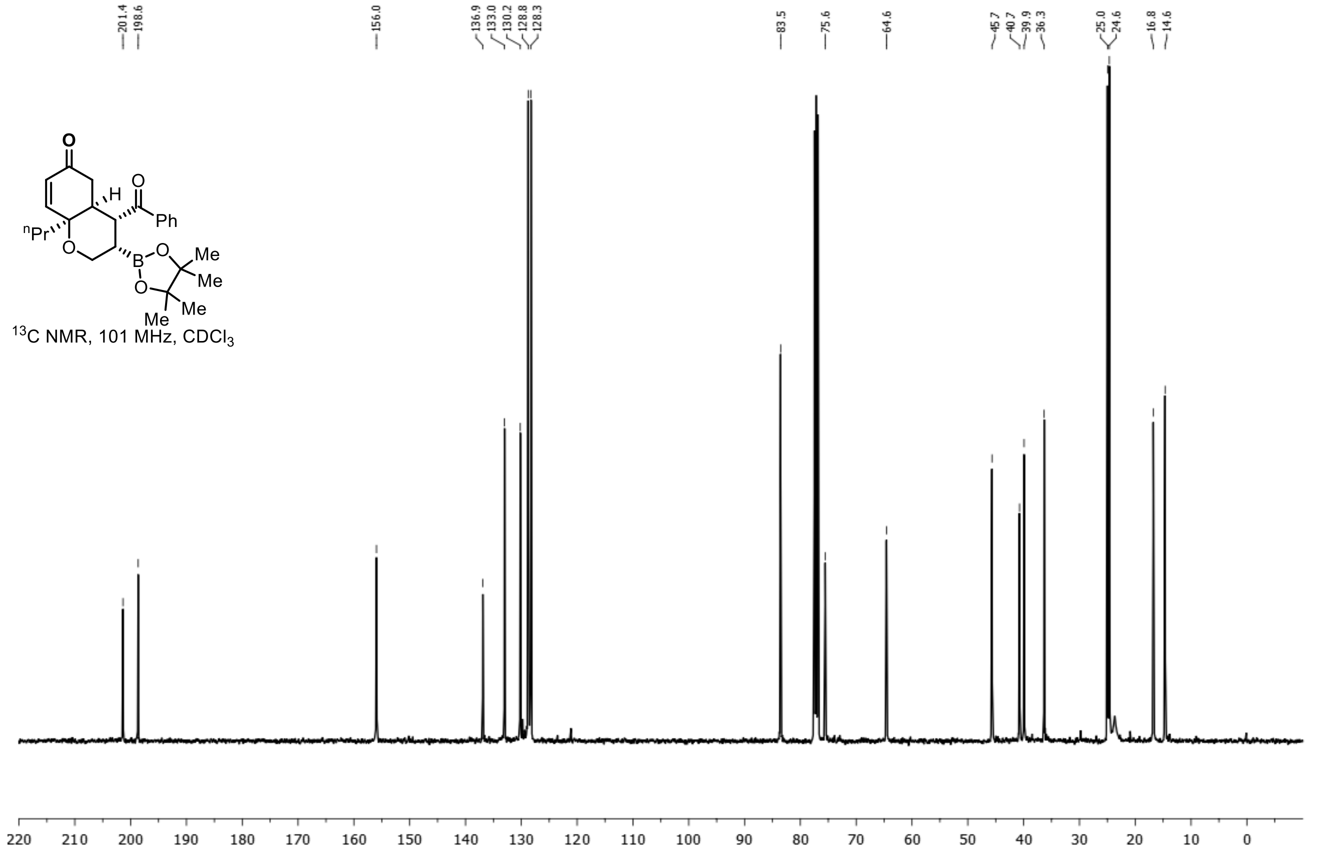

Supplementary Figure 30. ^{1}H NMR and ^{13}C NMR spectra of compound 2c

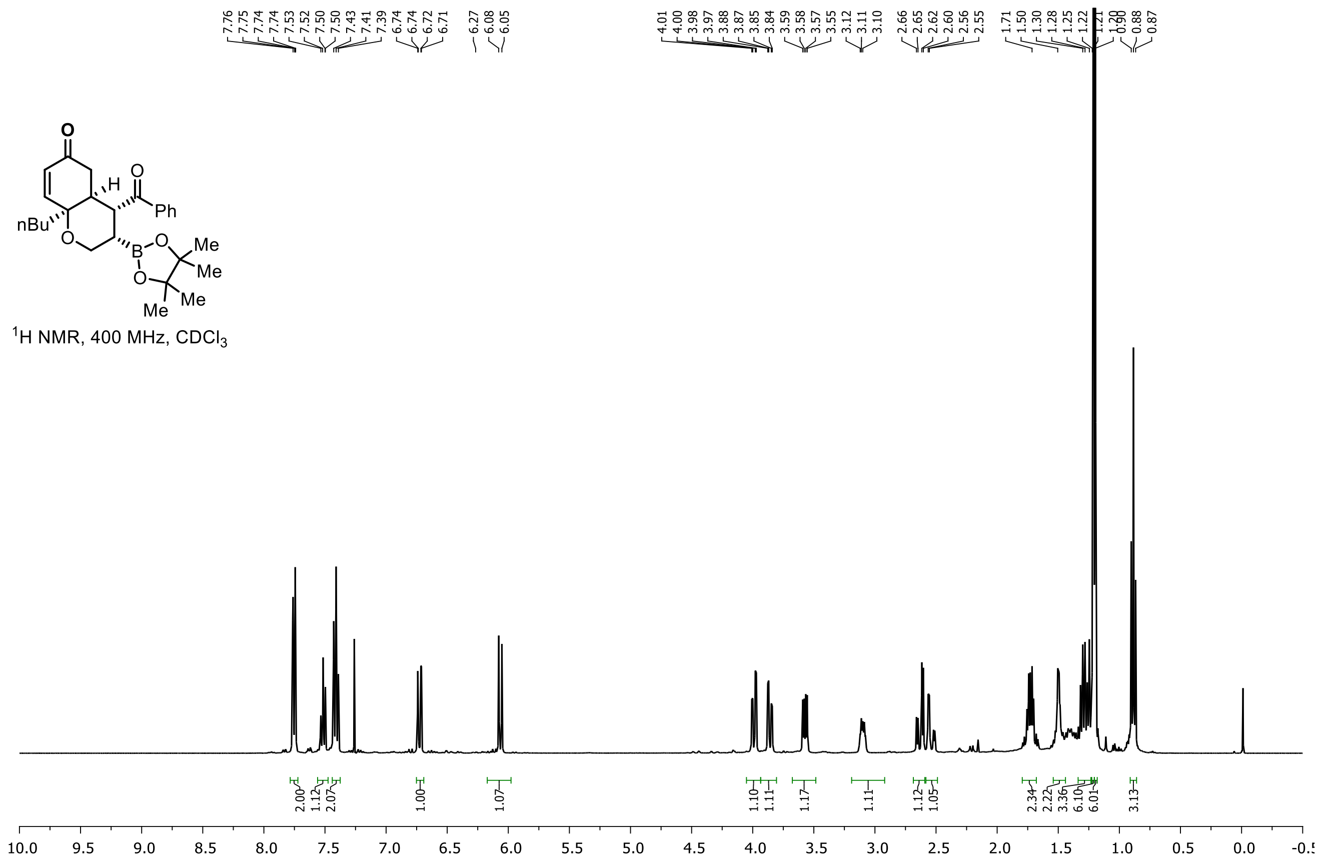

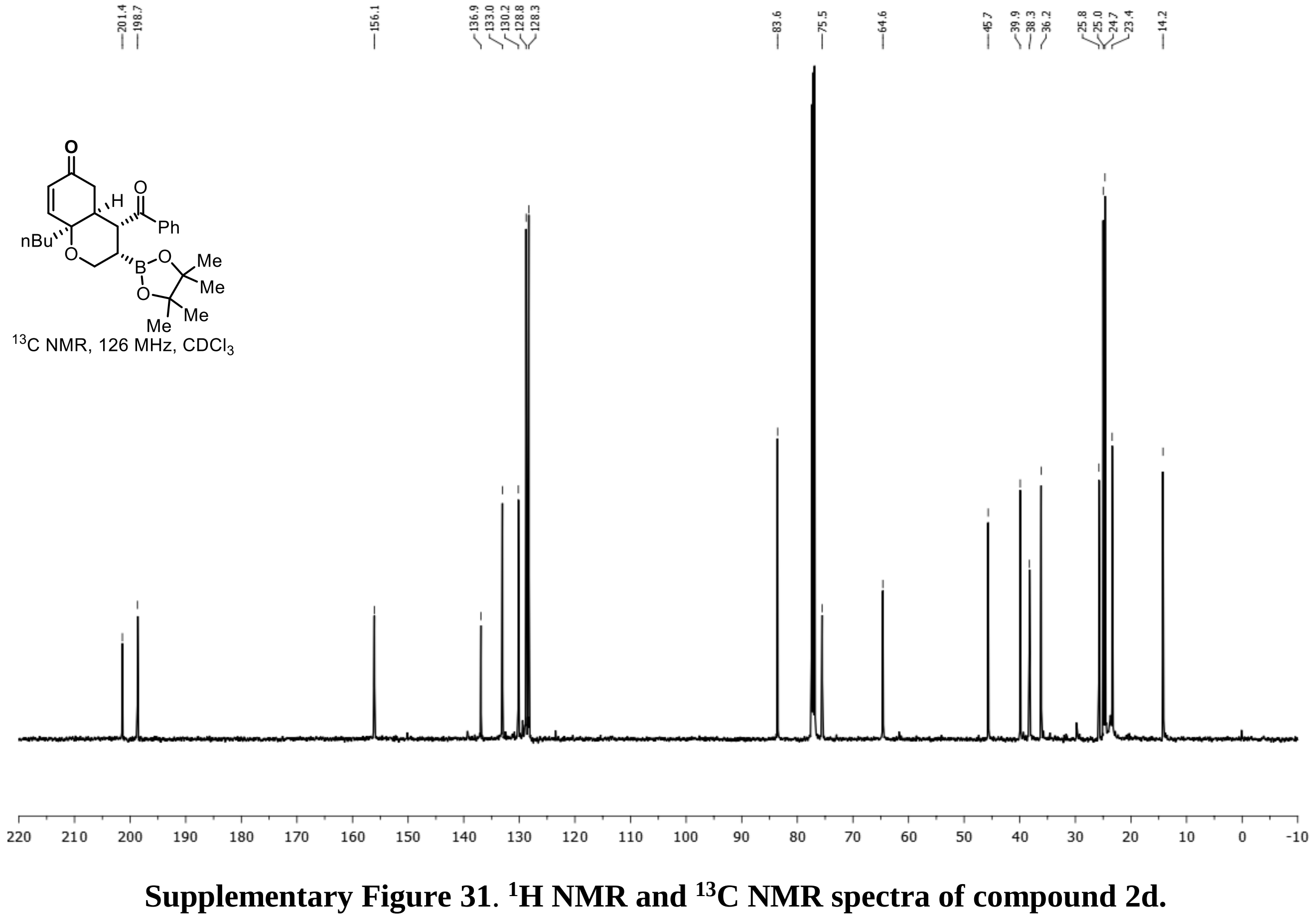

Supplementary Figure 31. **^{1}H NMR and ^{13}C NMR spectra of compound 2d.**

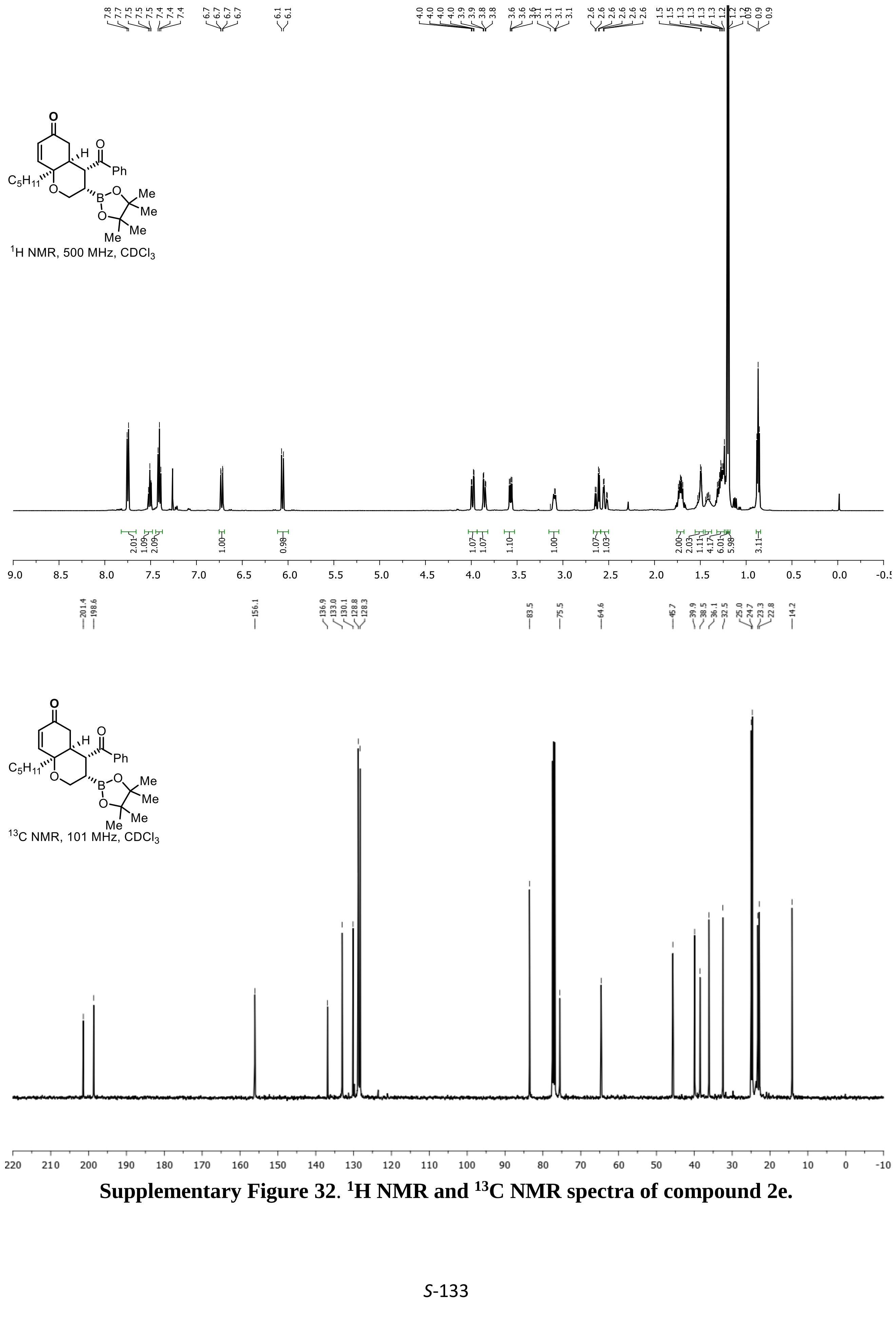

Supplementary Figure 32. **^{1}H NMR and ^{13}C NMR spectra of compound 2e.**

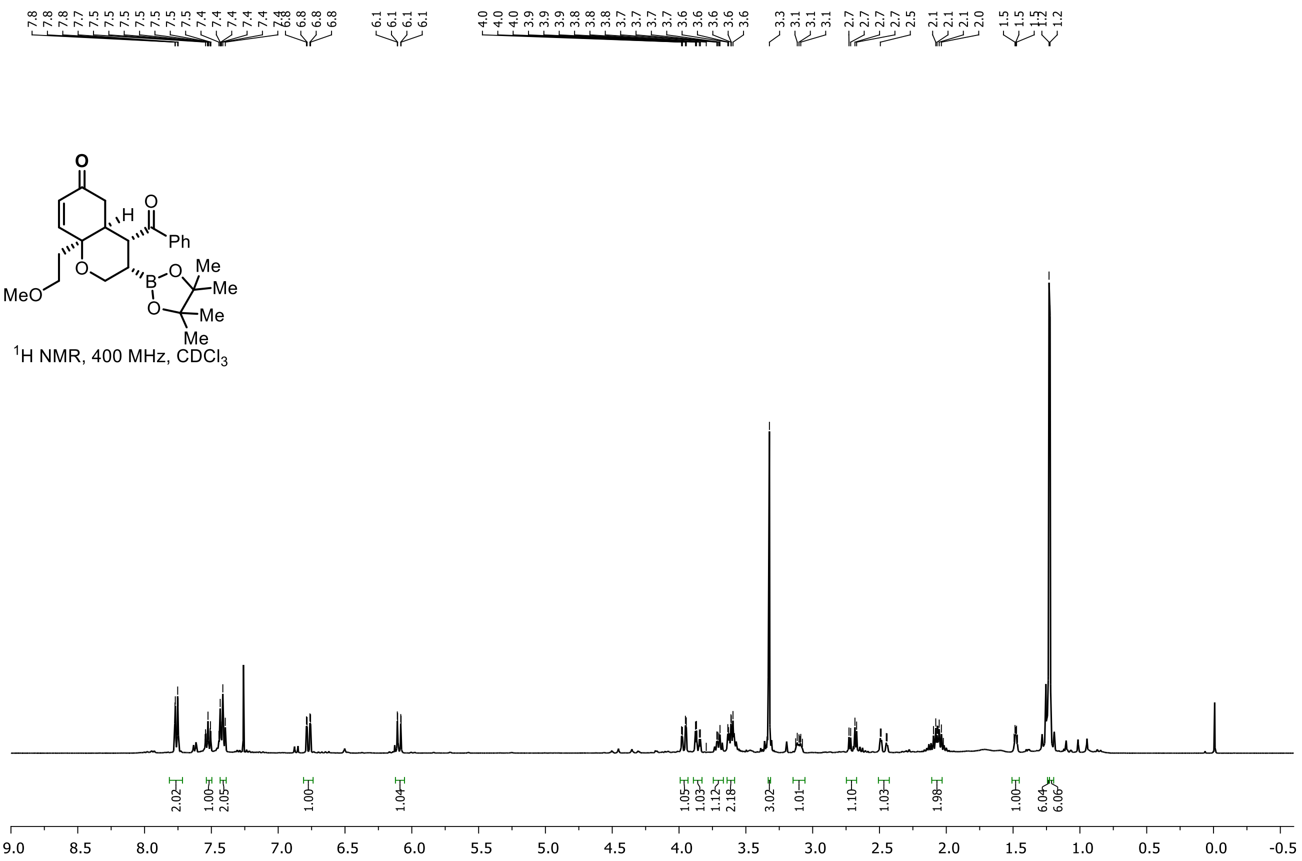

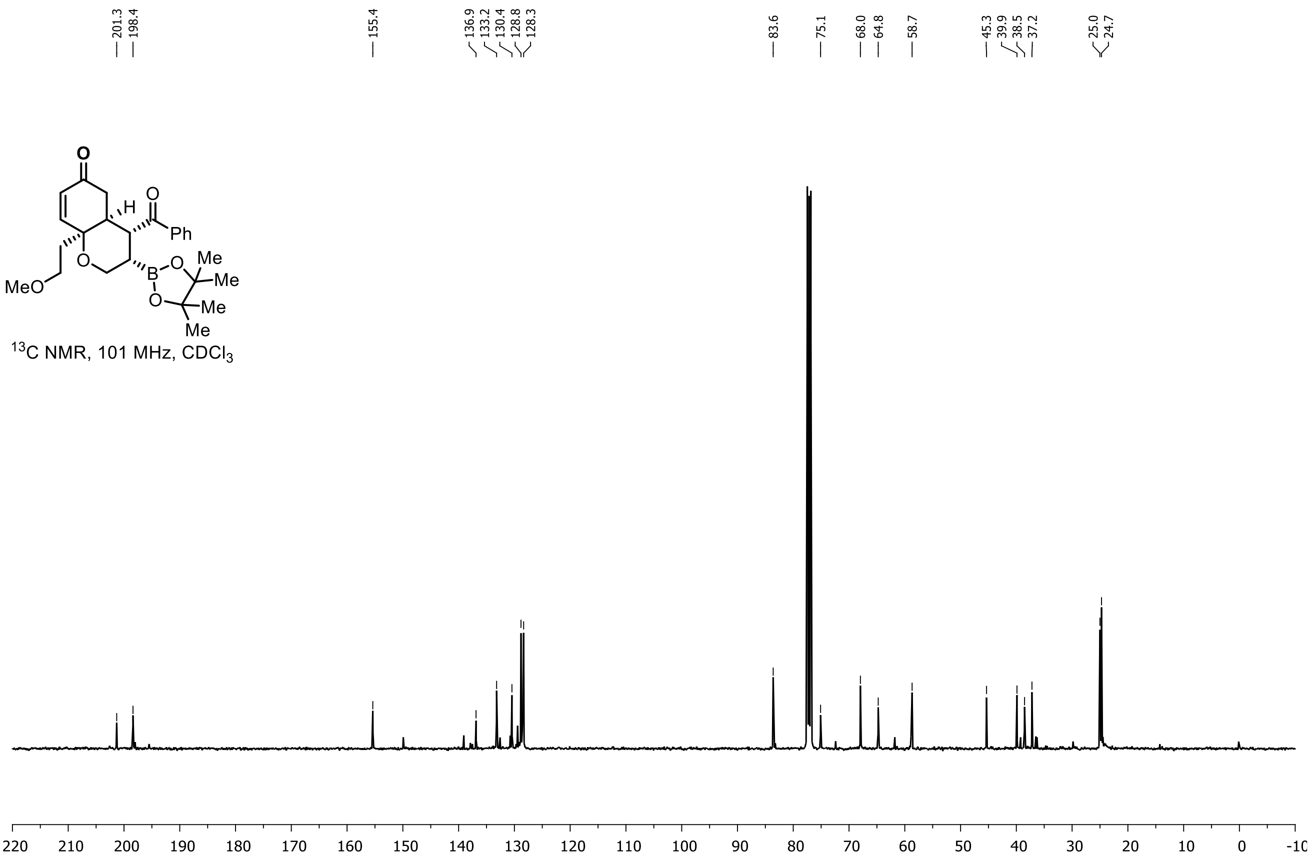

Supplementary Figure 33. ^{1}H NMR and ^{13}C NMR spectra of compound 2f.

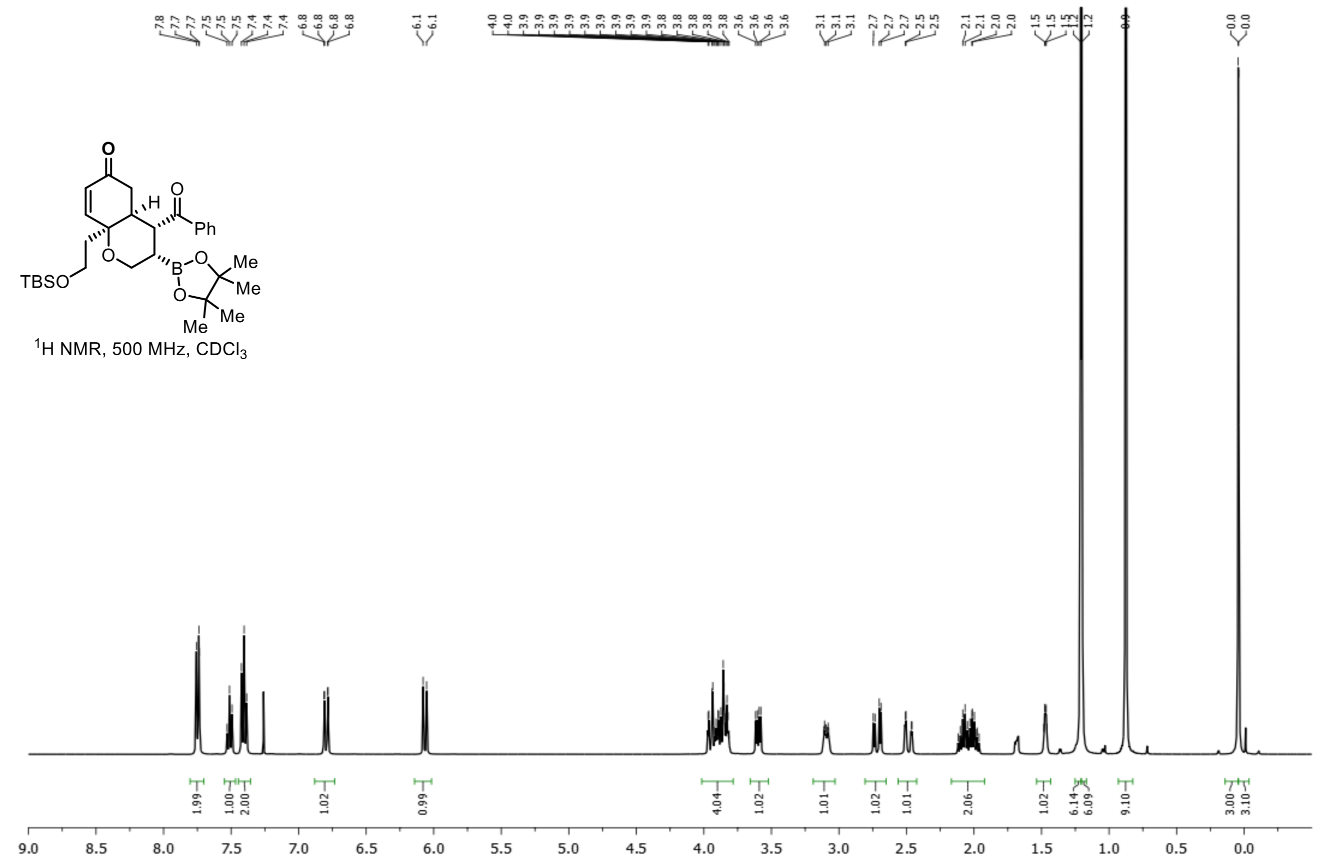

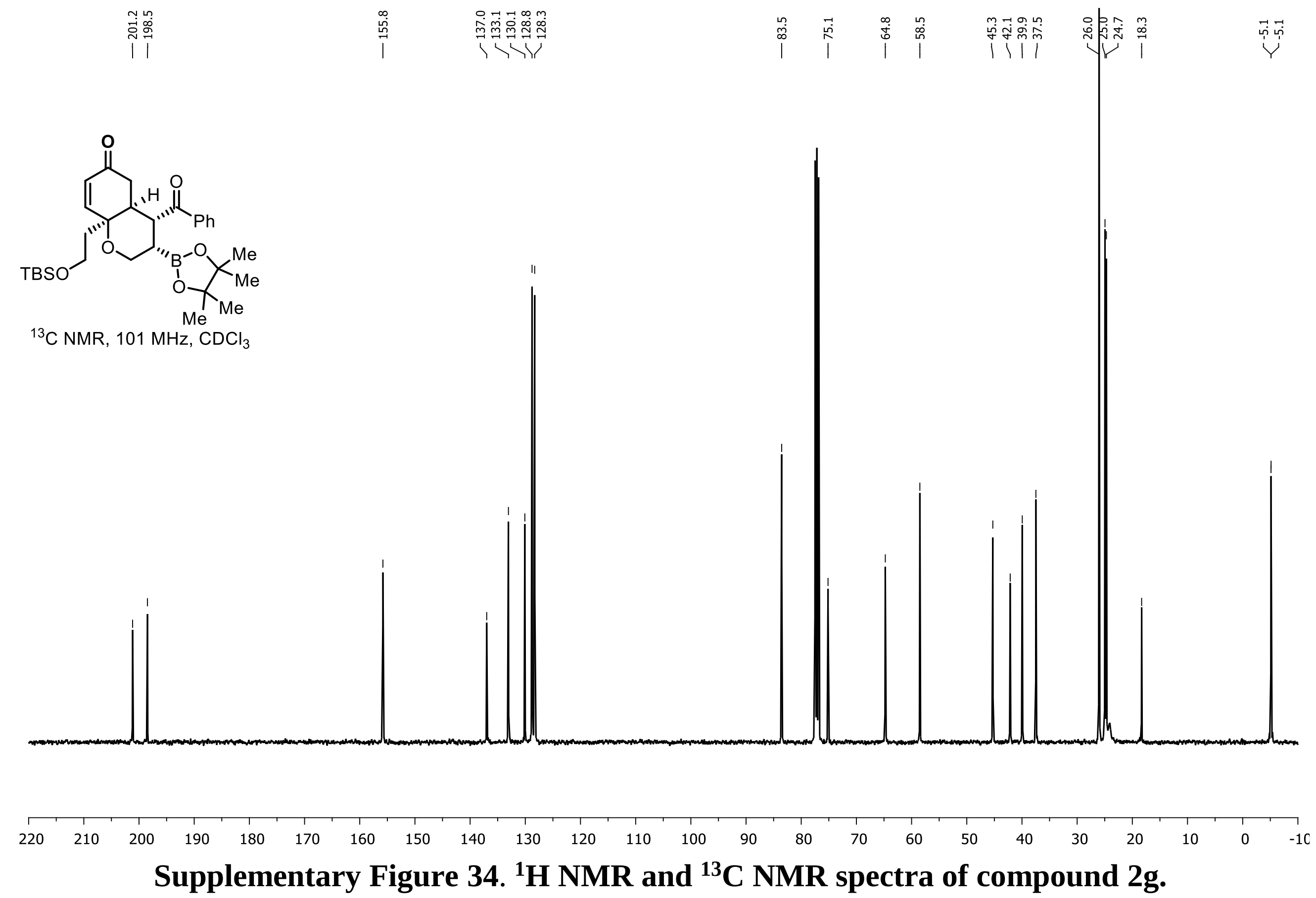

Supplementary Figure 34. ^{1}H NMR and ^{13}C NMR spectra of compound 2g.

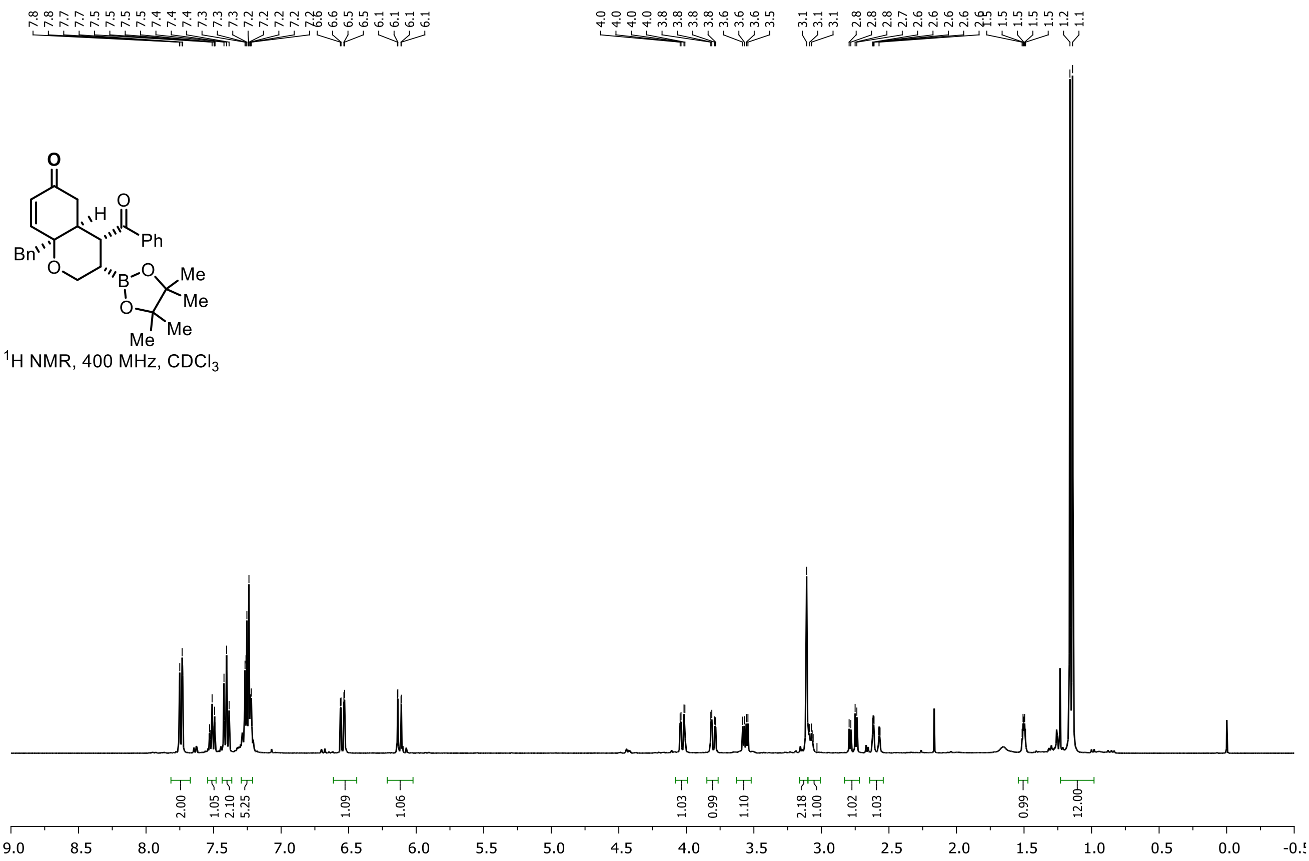

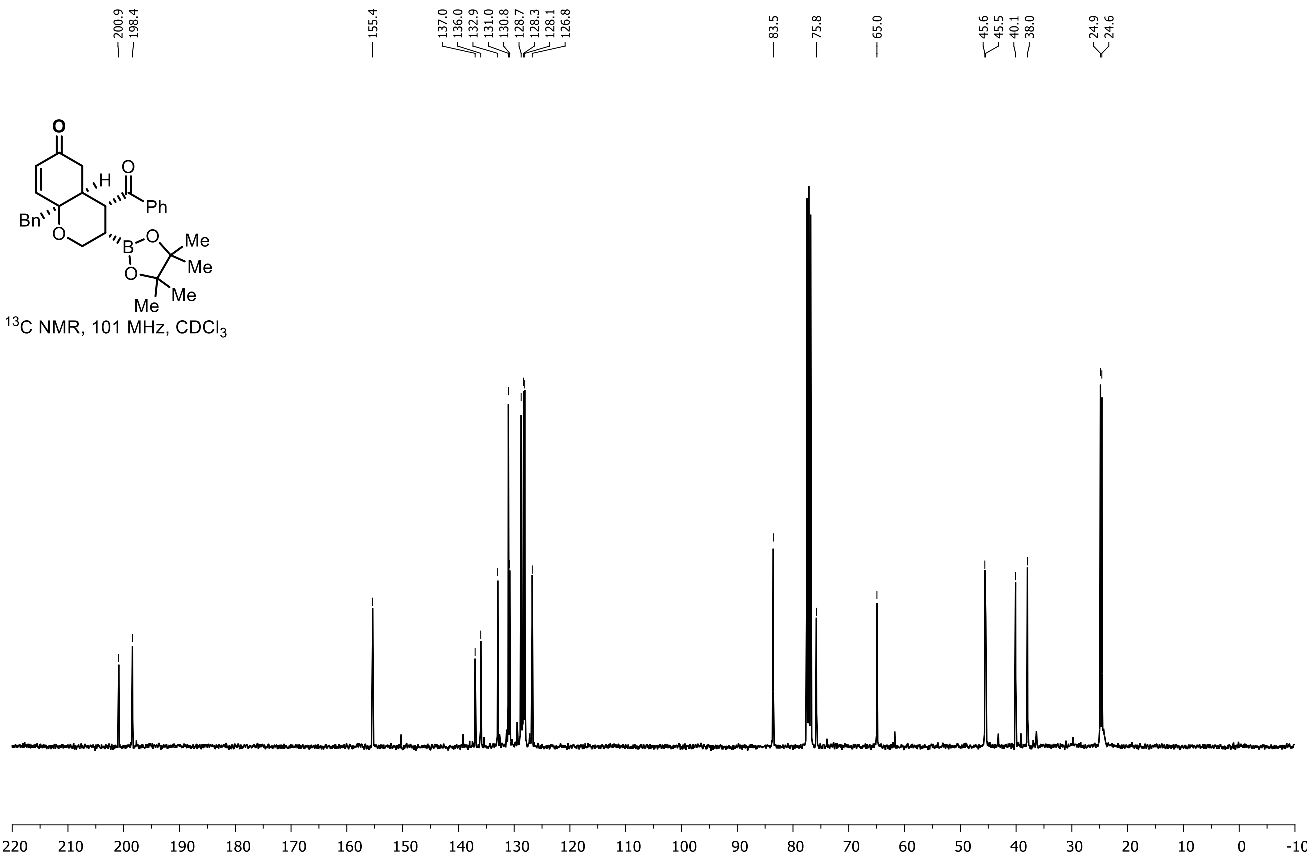

Supplementary Figure 35. ^{1}H NMR and ^{13}C NMR spectra of compound 2h.

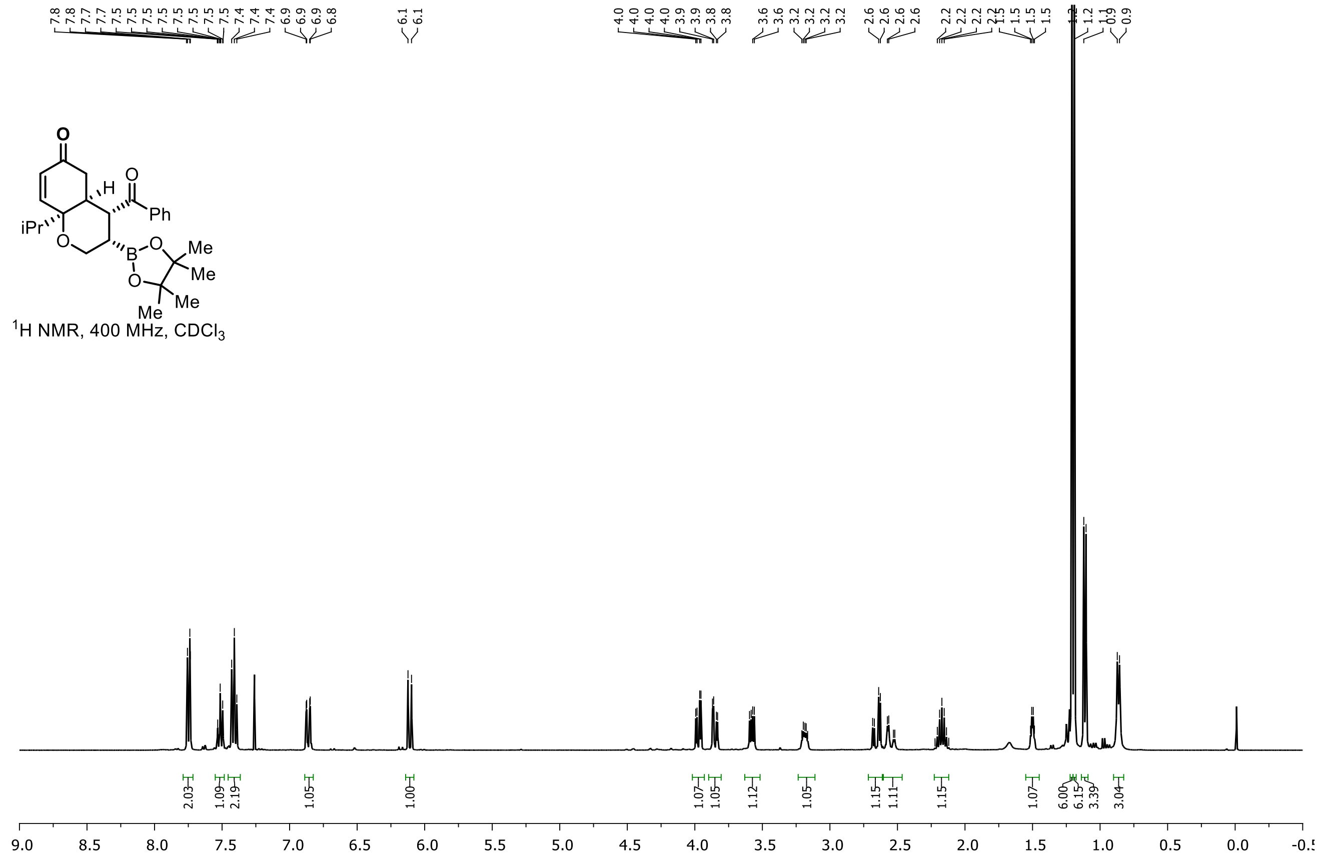

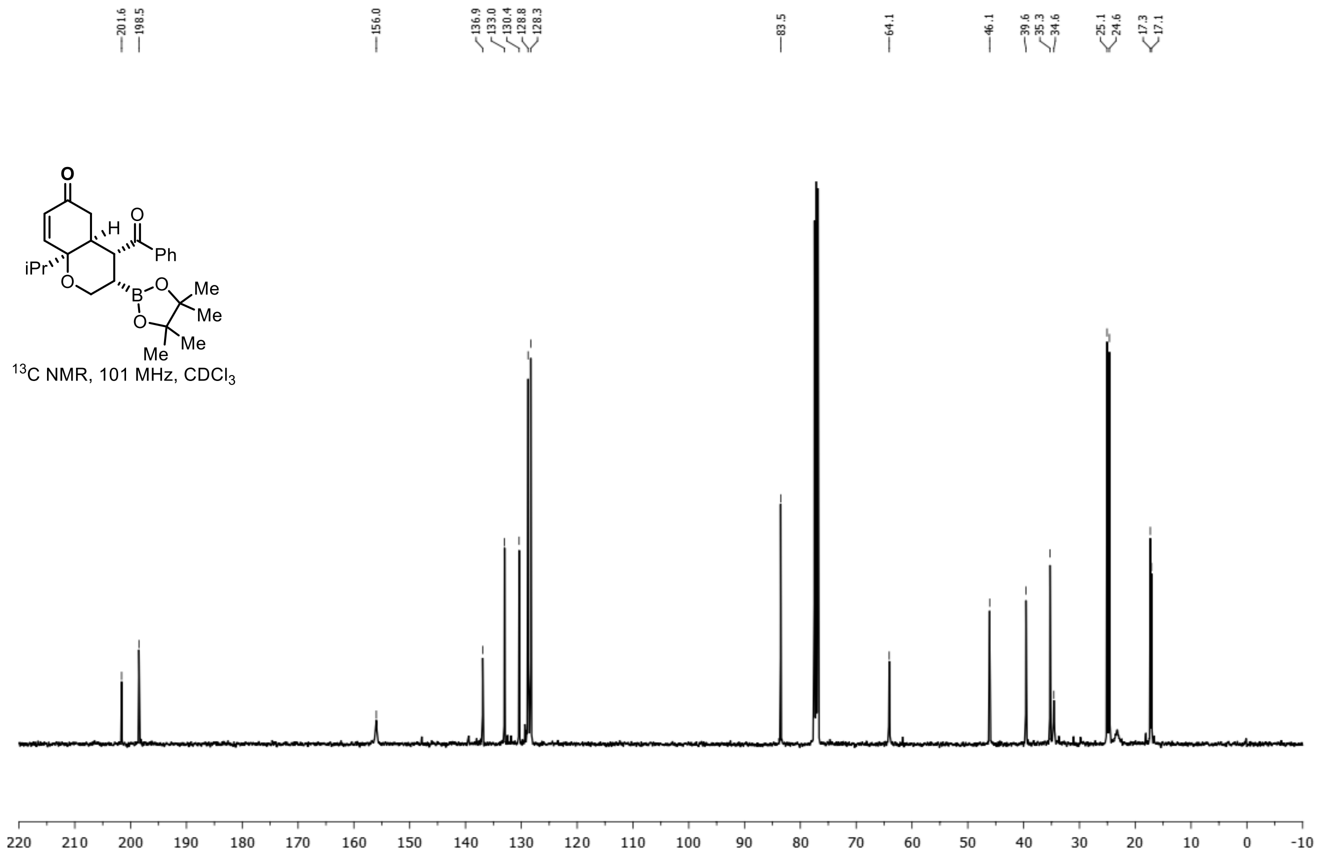

Supplementary Figure 36. **^{1}H NMR and ^{13}C NMR spectra of compound 2i.**

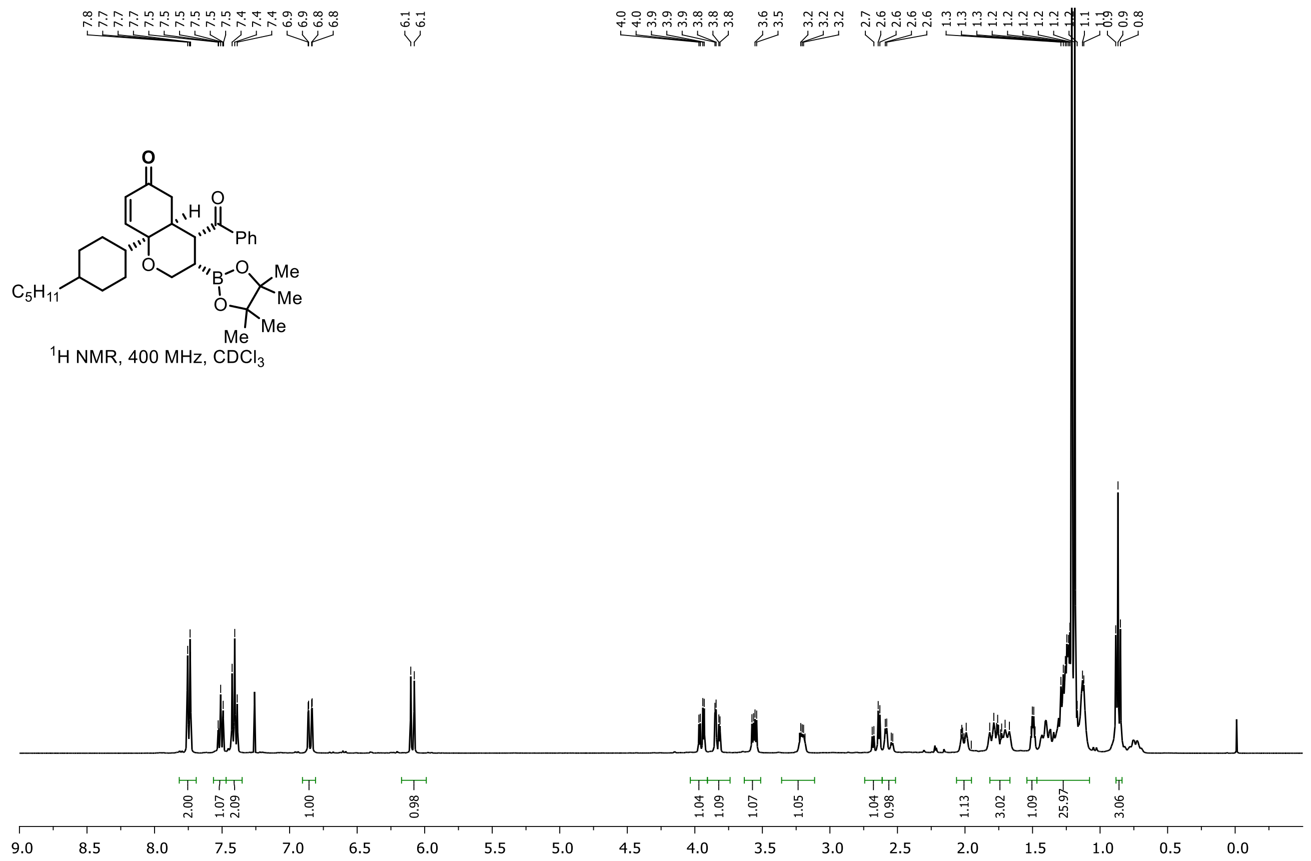

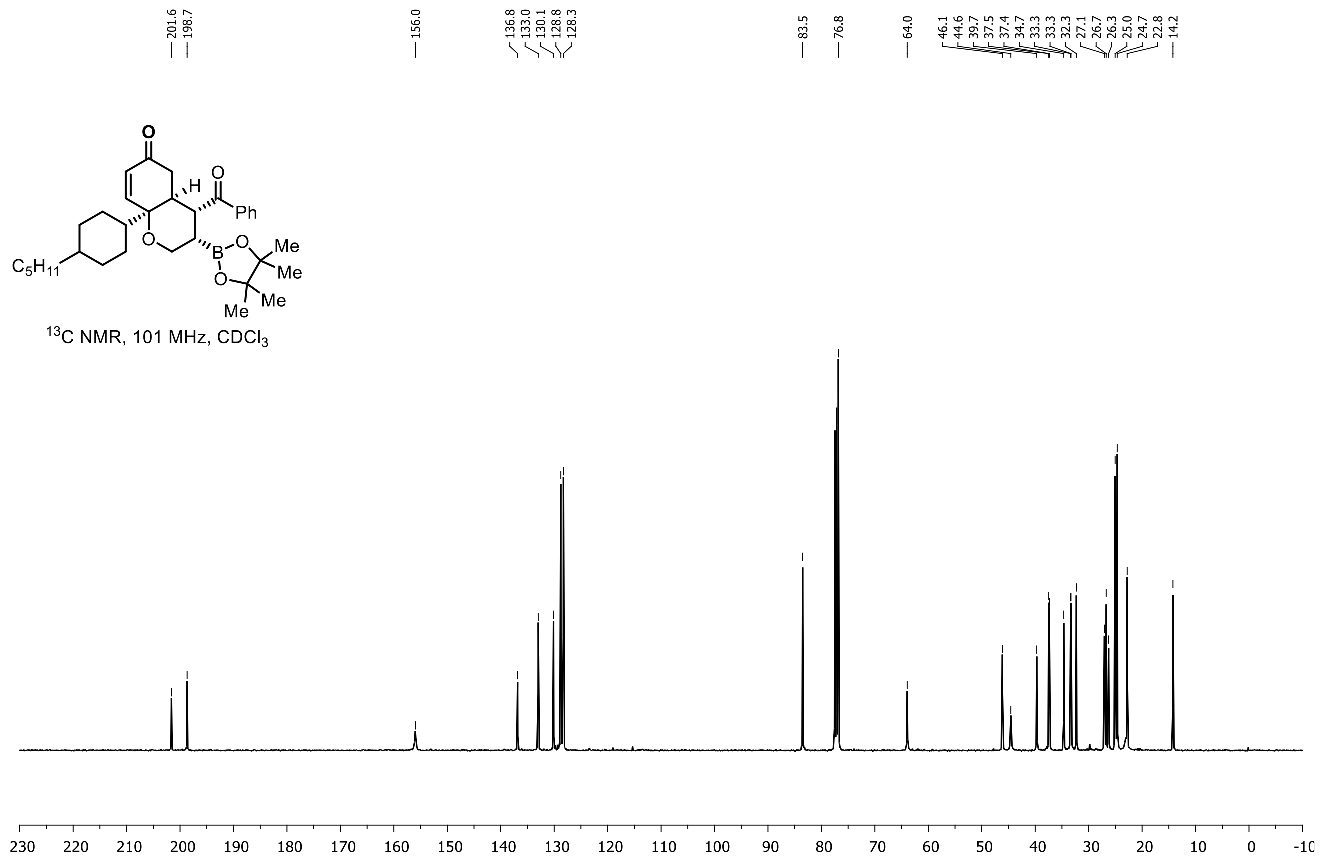

Supplementary Figure 37. ^{1}H NMR and ^{13}C NMR spectra of compound 2j.

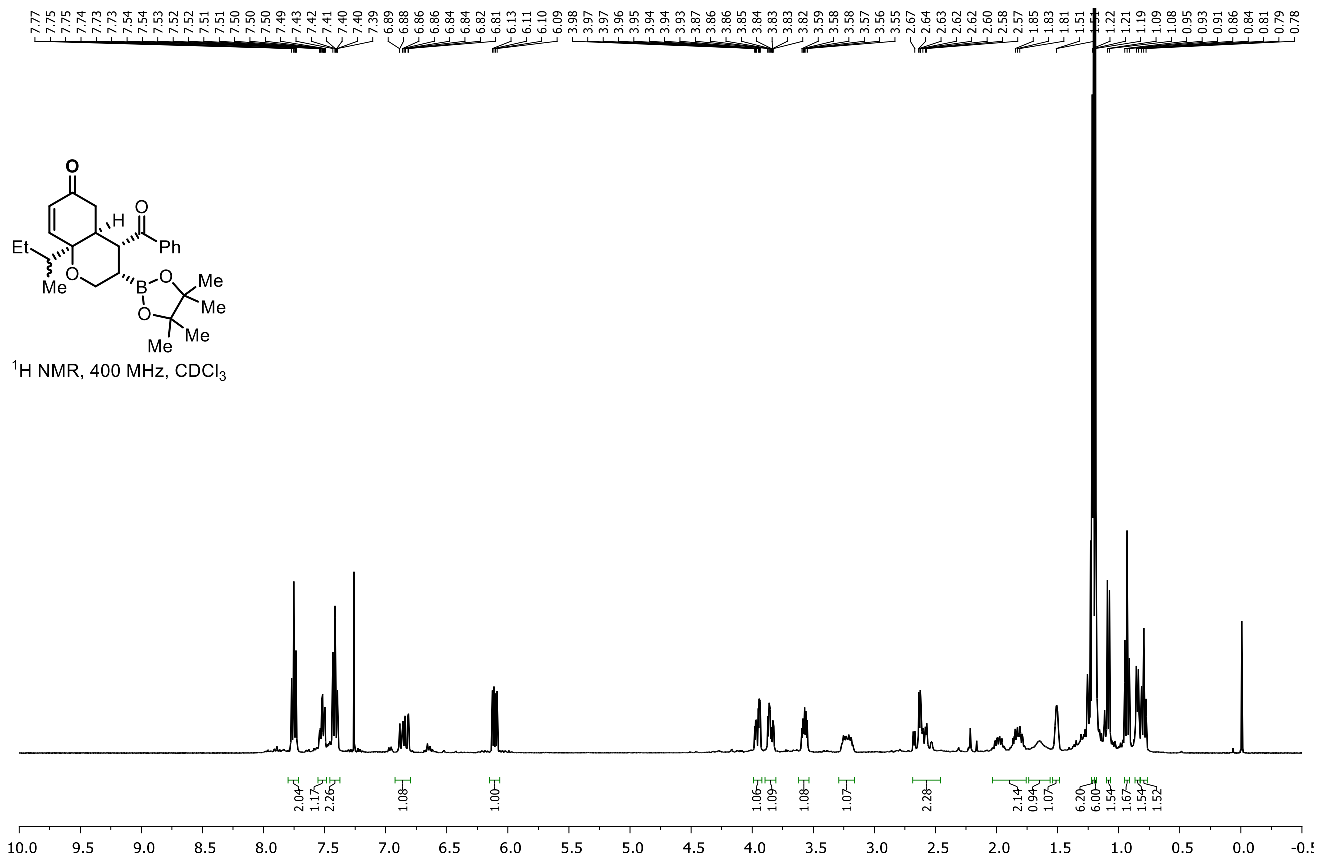

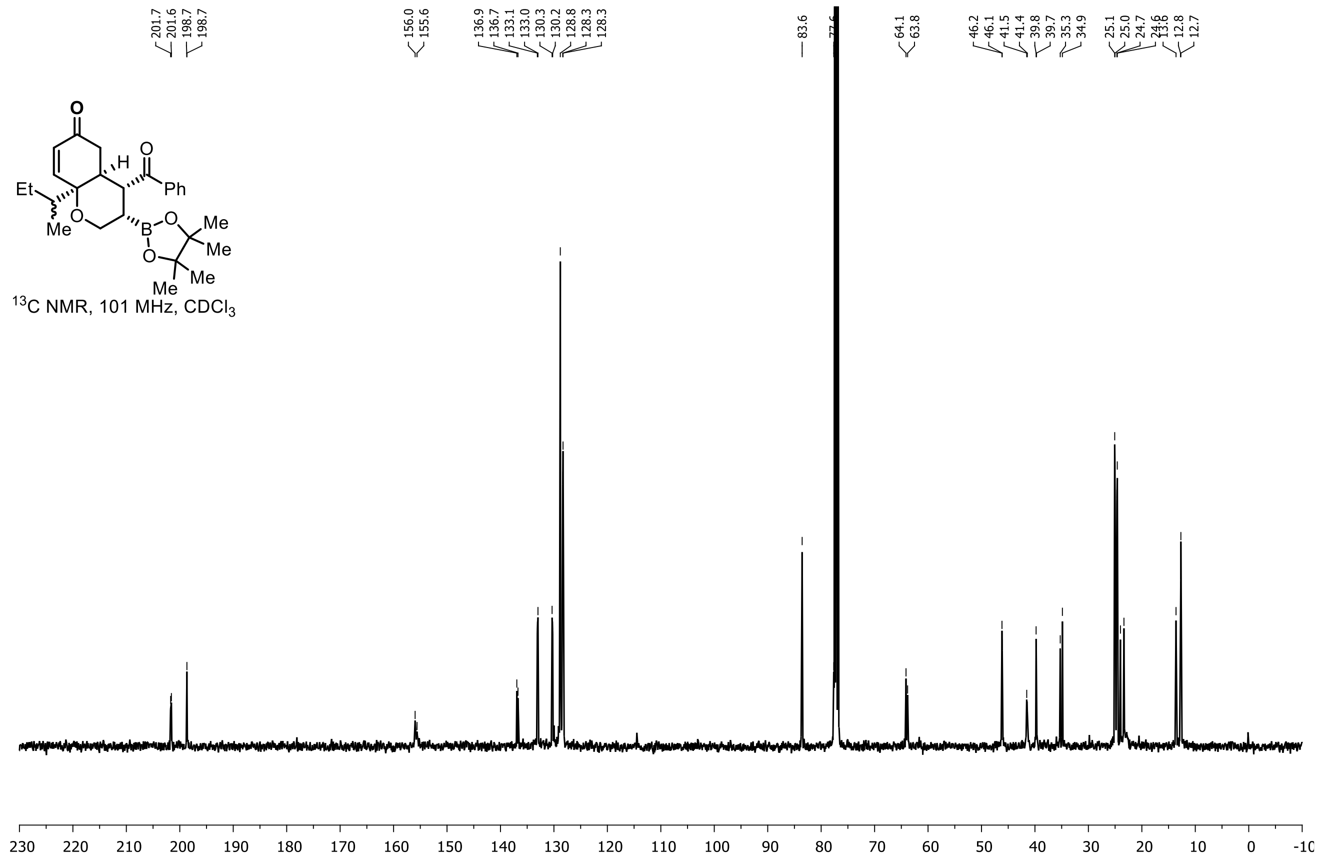

Supplementary Figure 38. **^{1}H NMR and ^{13}C NMR spectra of compound 2k.**

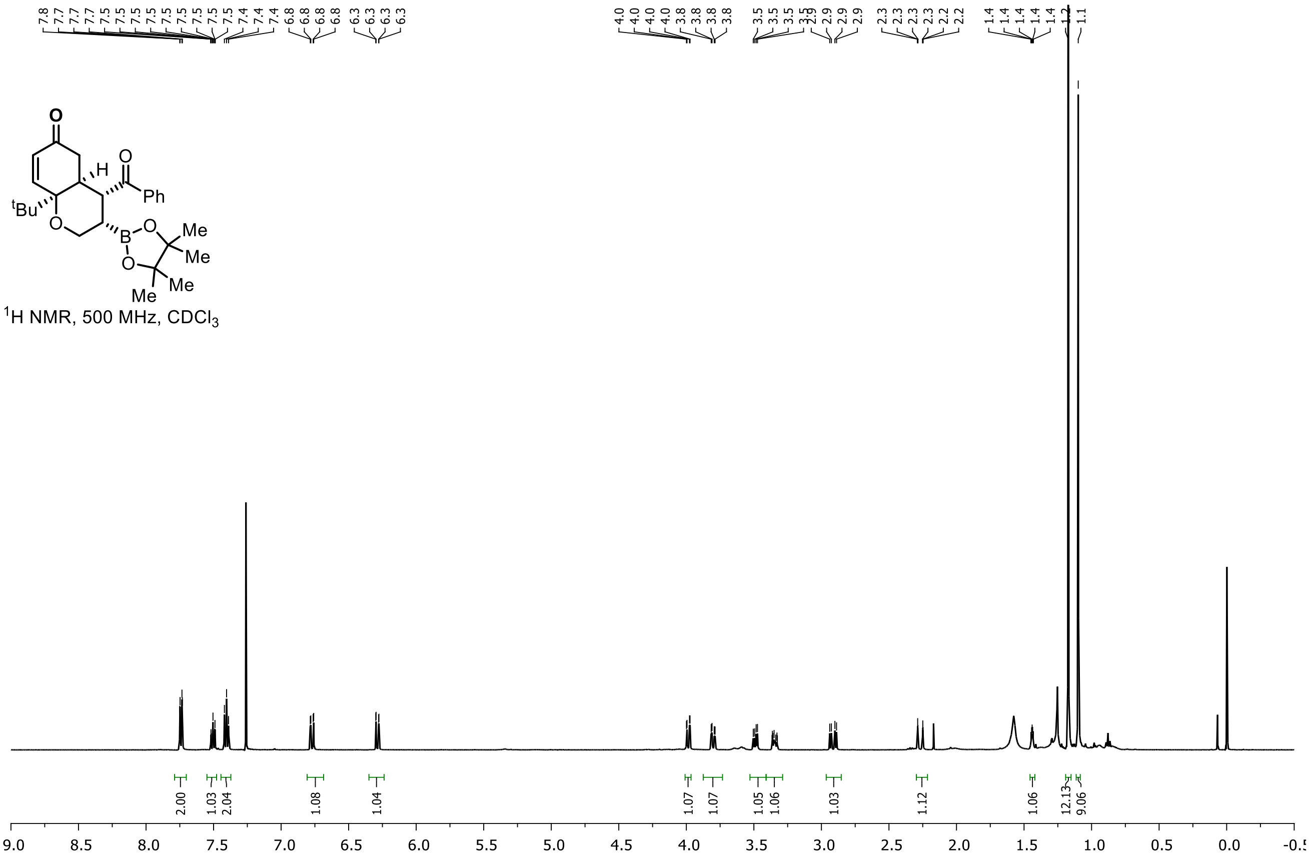

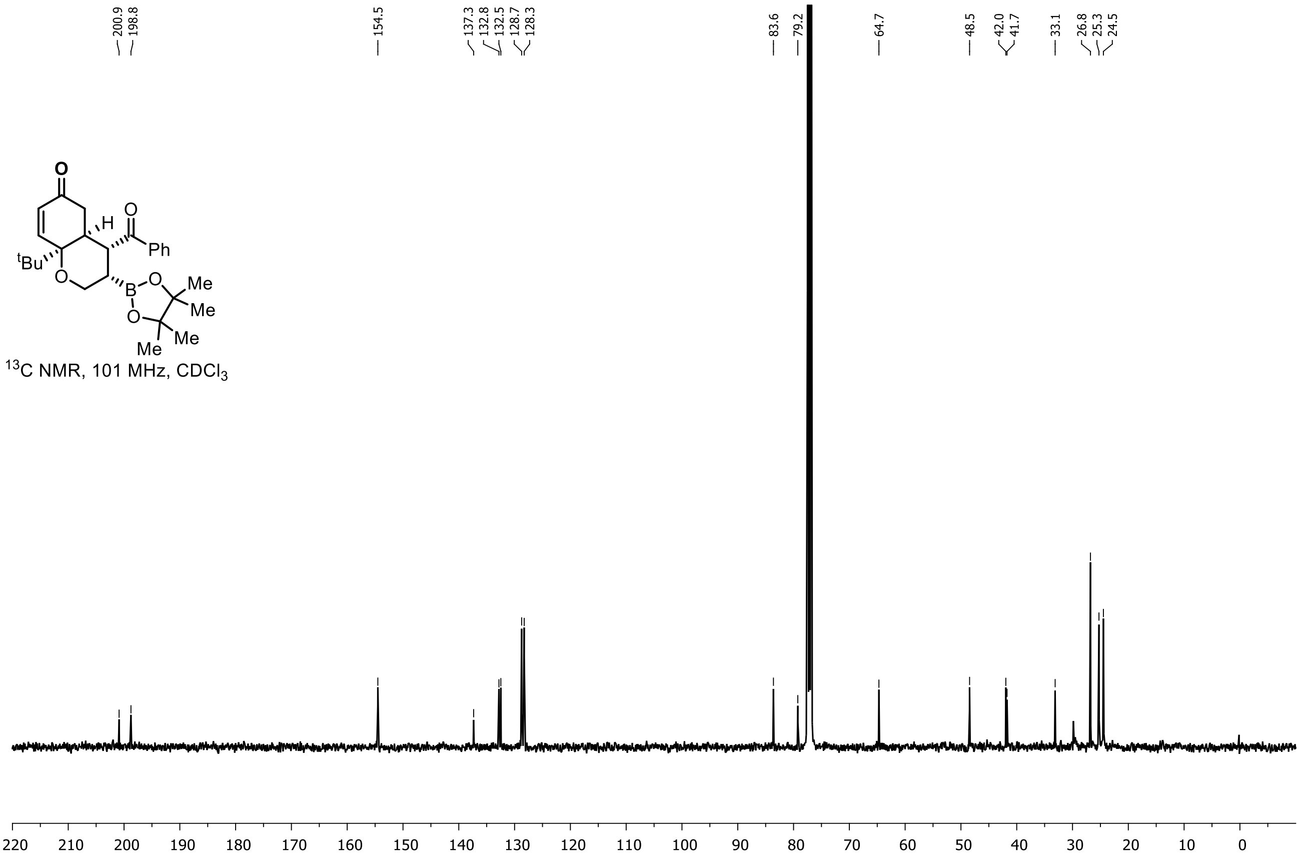

Supplementary Figure 39. **^{1}H NMR and ^{13}C NMR spectra of compound 2l.**

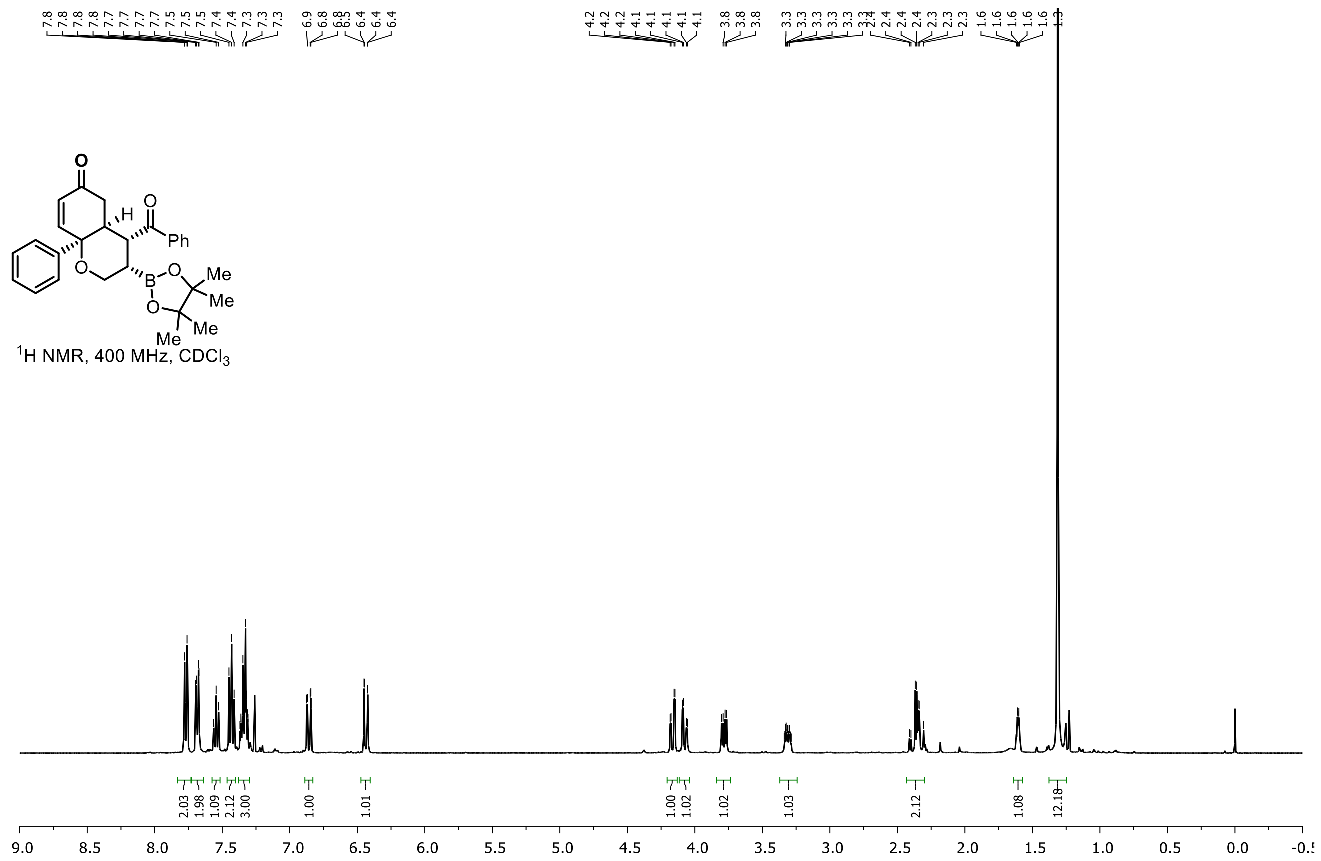

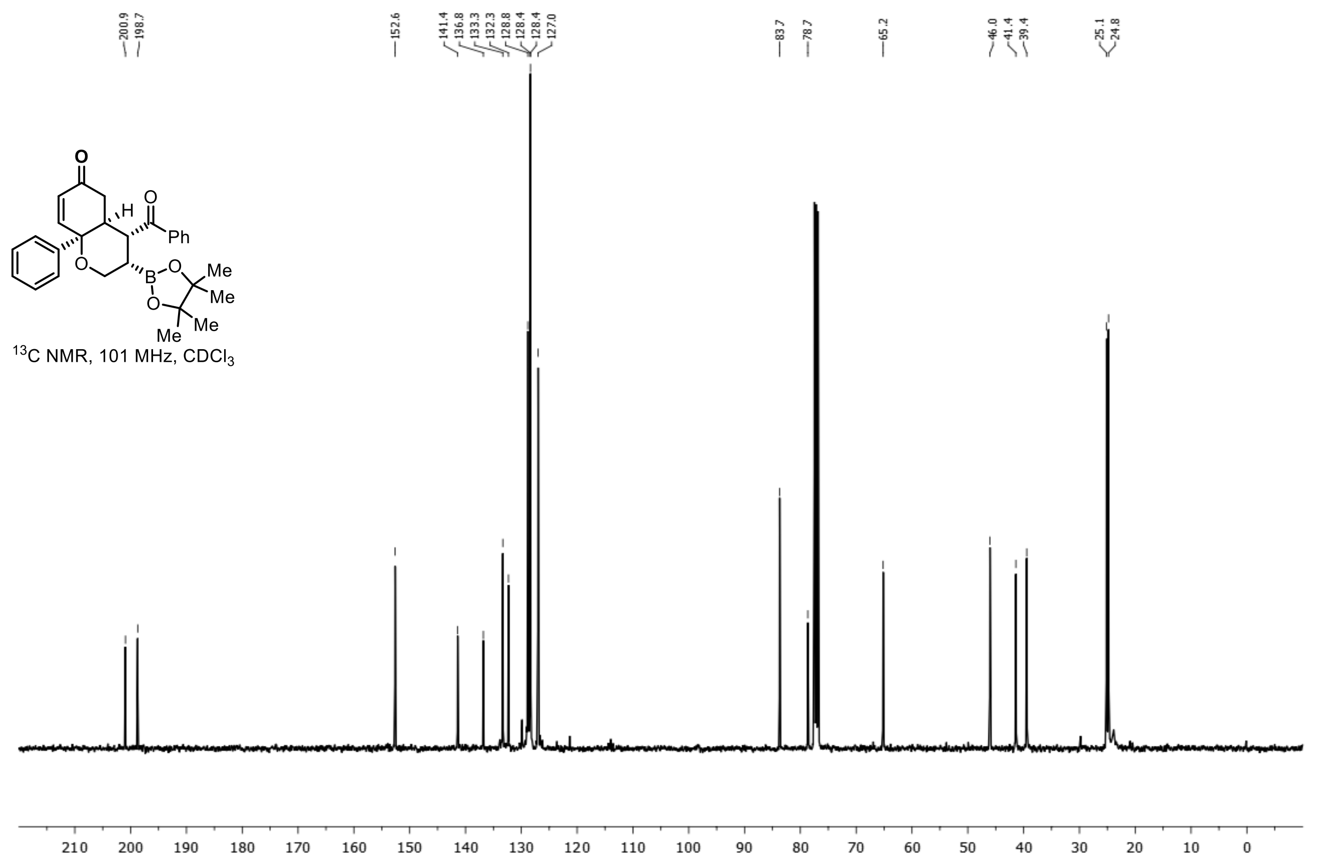

Supplementary Figure 40. ^{1}H NMR and ^{13}C NMR spectra of compound 2m.

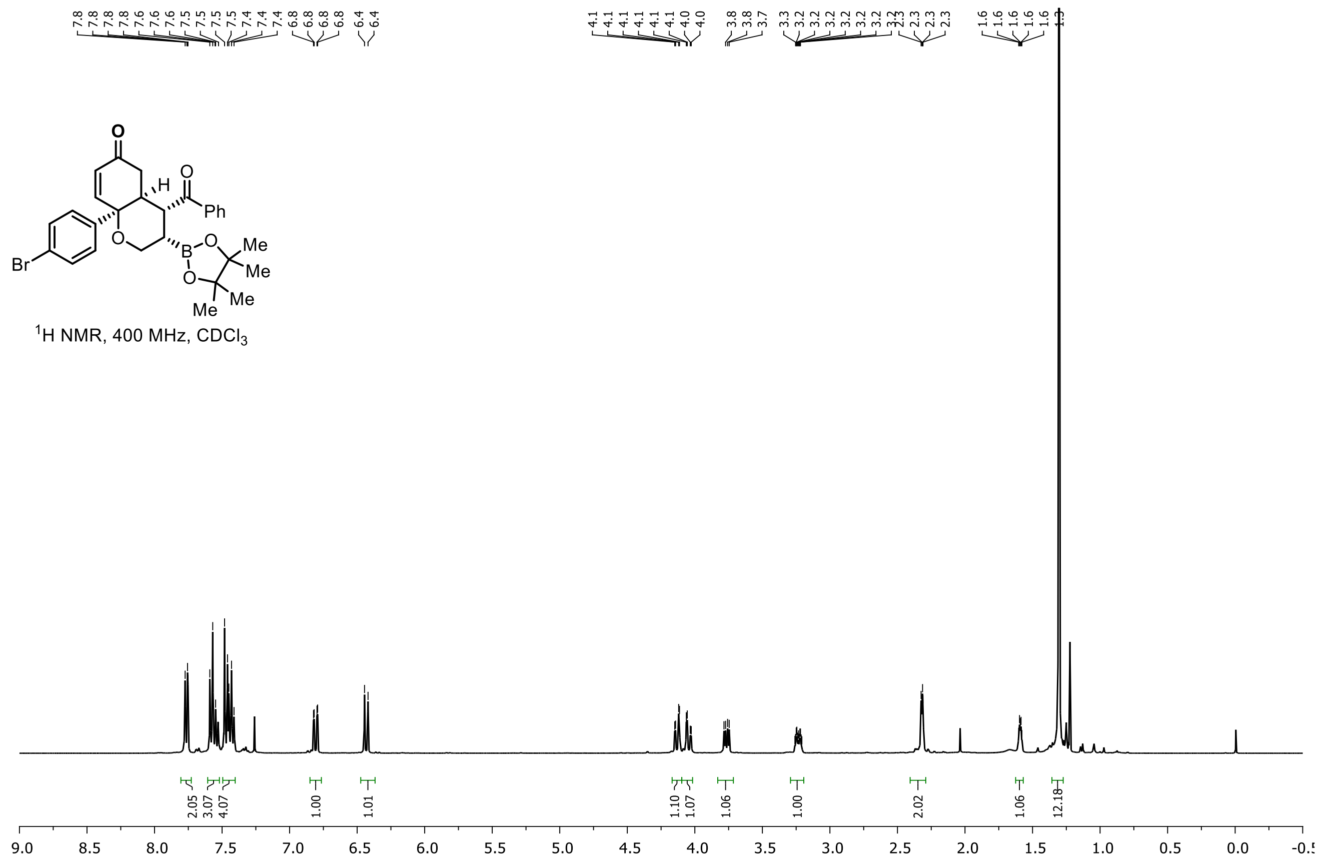

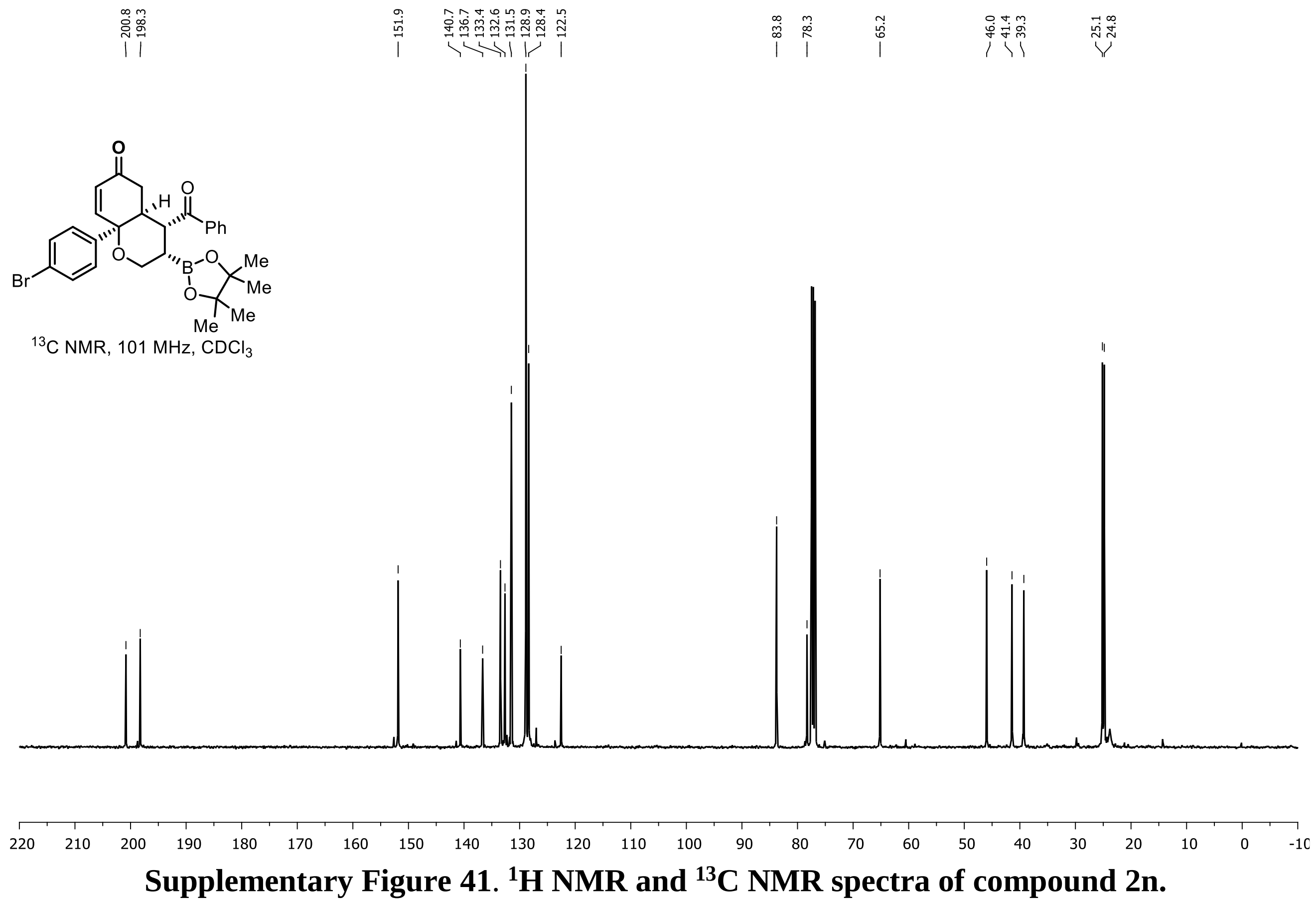

Supplementary Figure 41. ^{1}H NMR and ^{13}C NMR spectra of compound 2n.

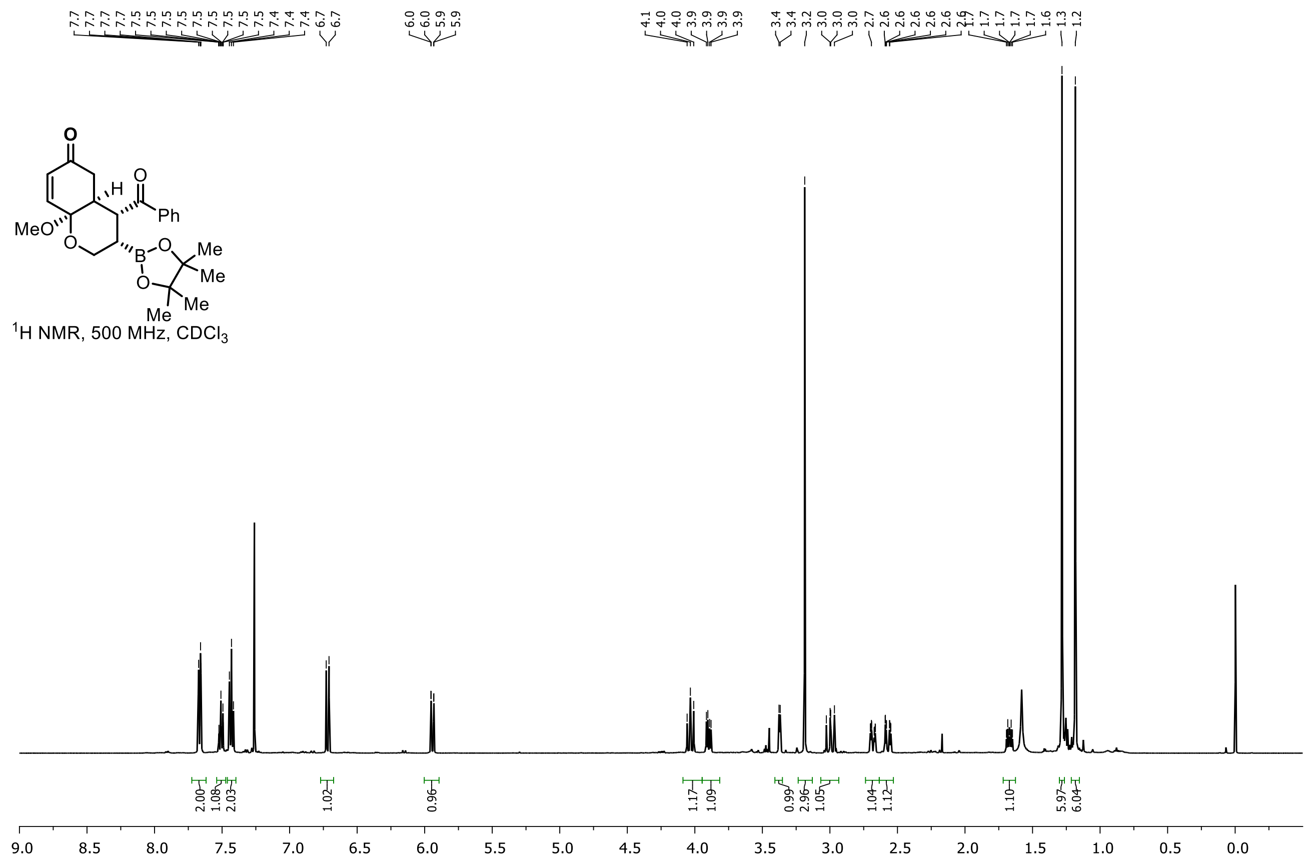

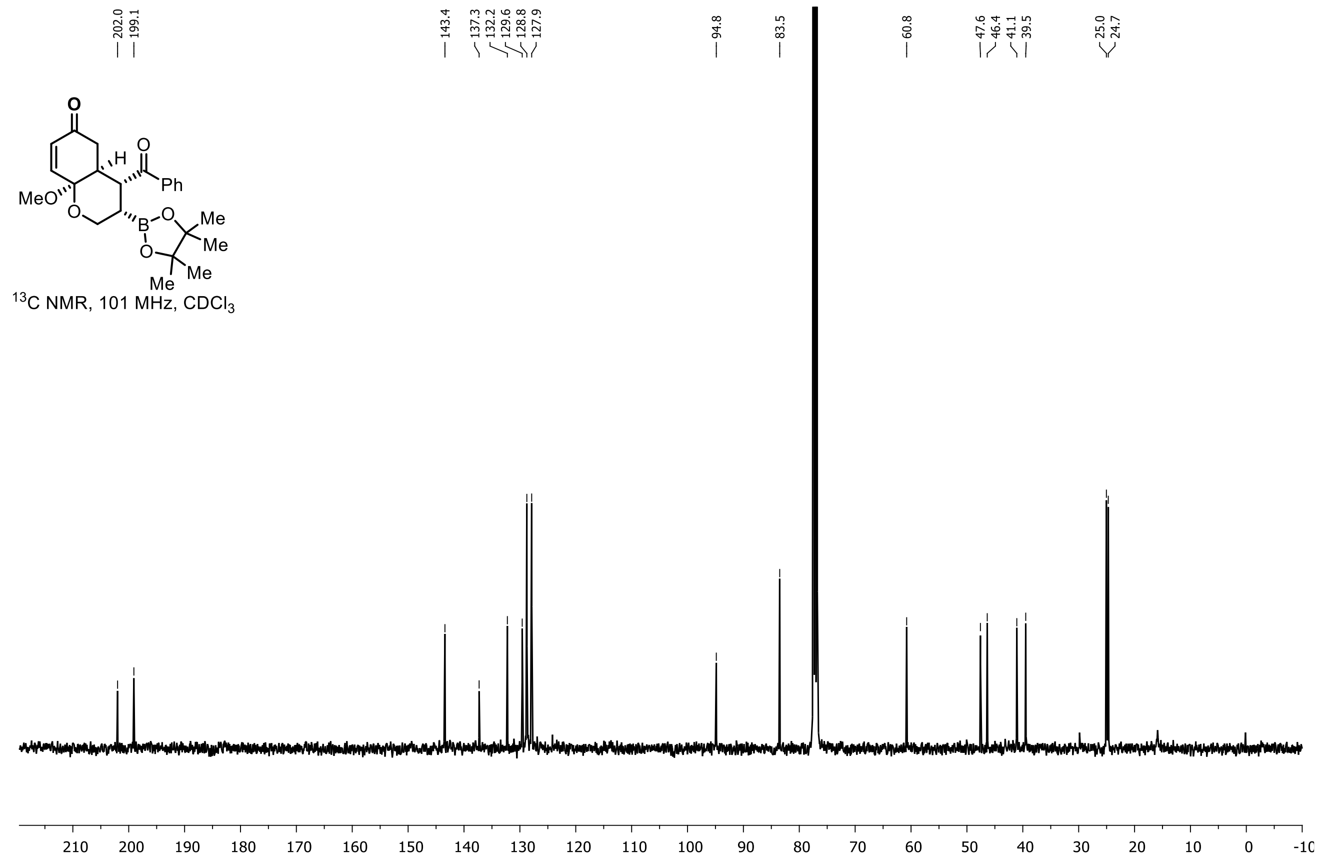

Supplementary Figure 42. **^{1}H NMR and ^{13}C NMR spectra of compound 2o.**

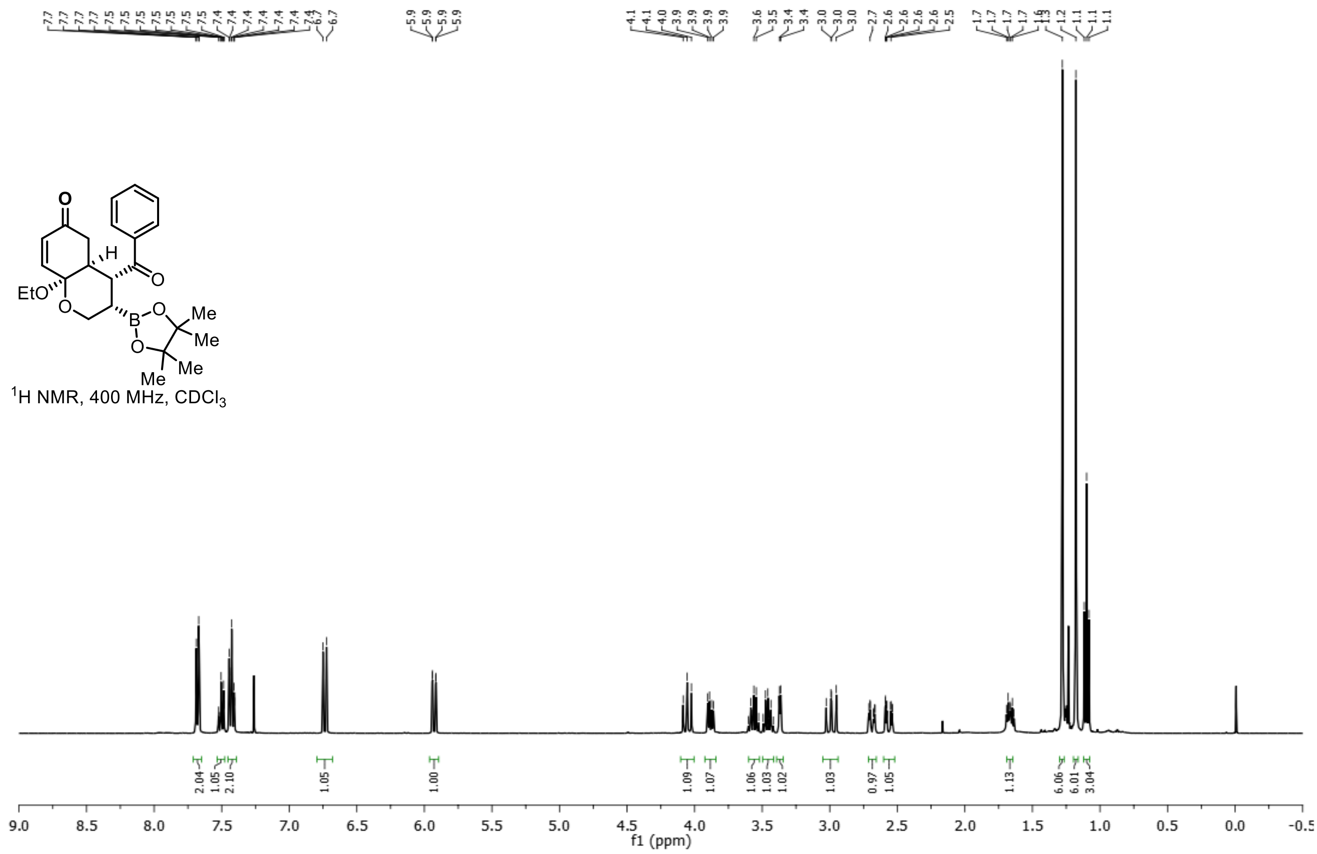

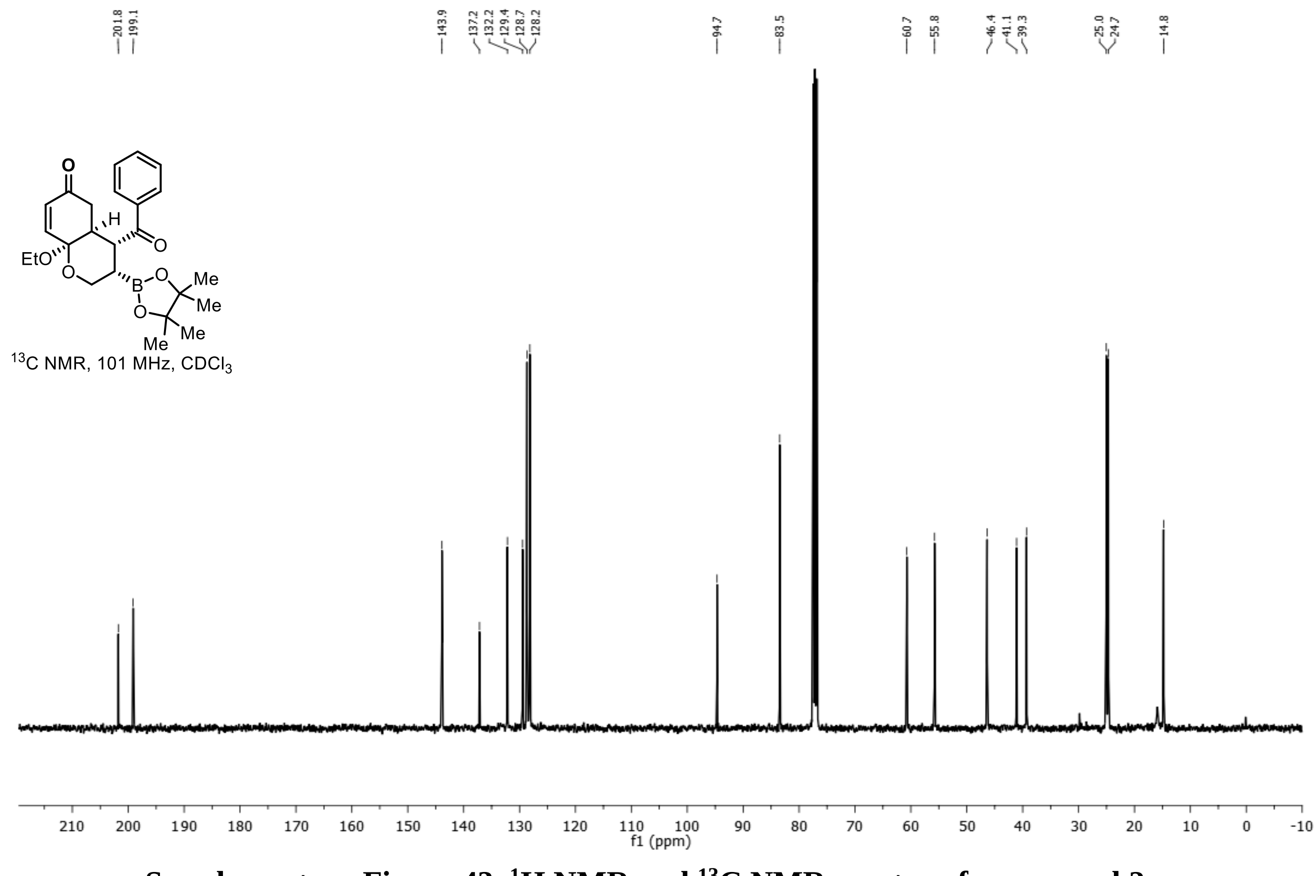

Supplementary Figure 43. ^{1}H NMR and ^{13}C NMR spectra of compound 2p.

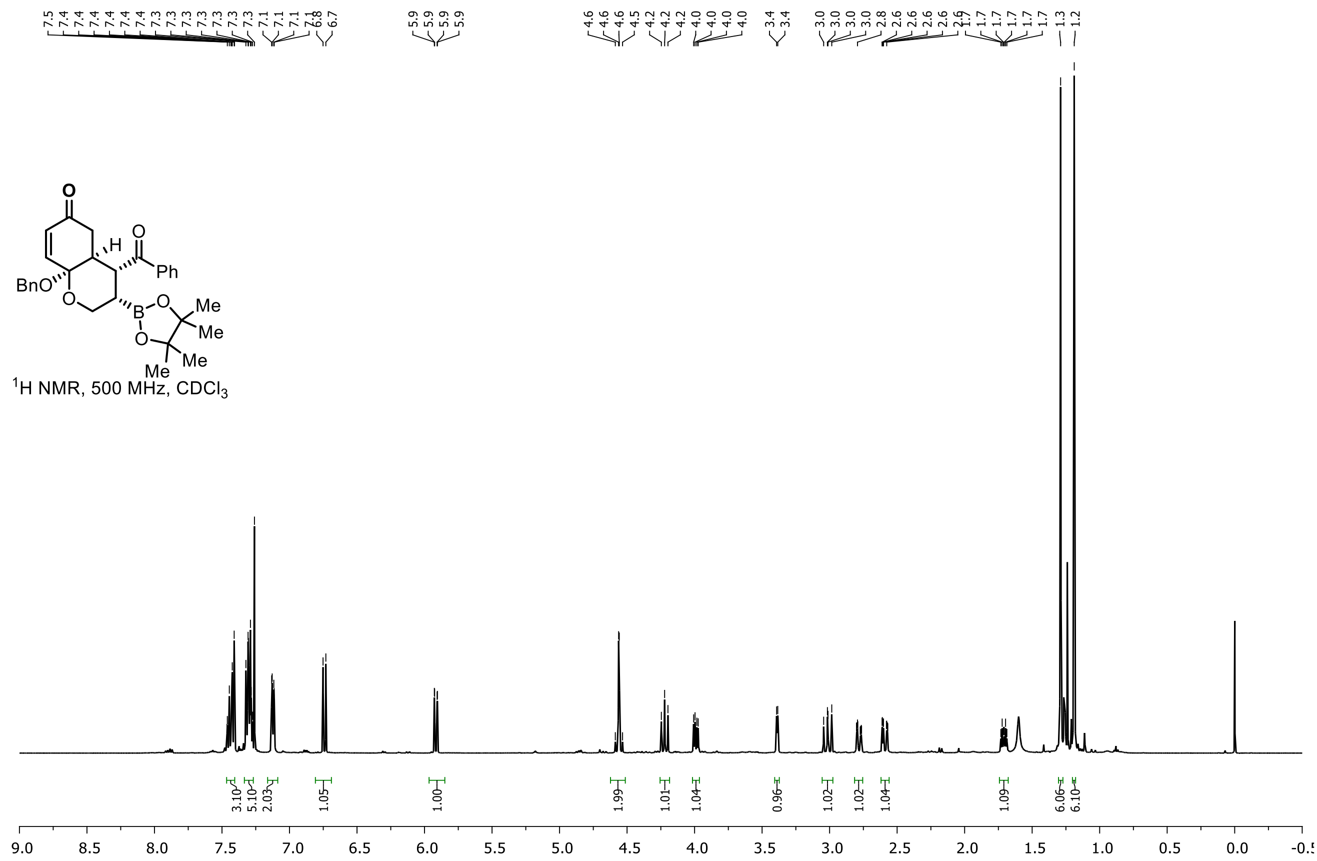

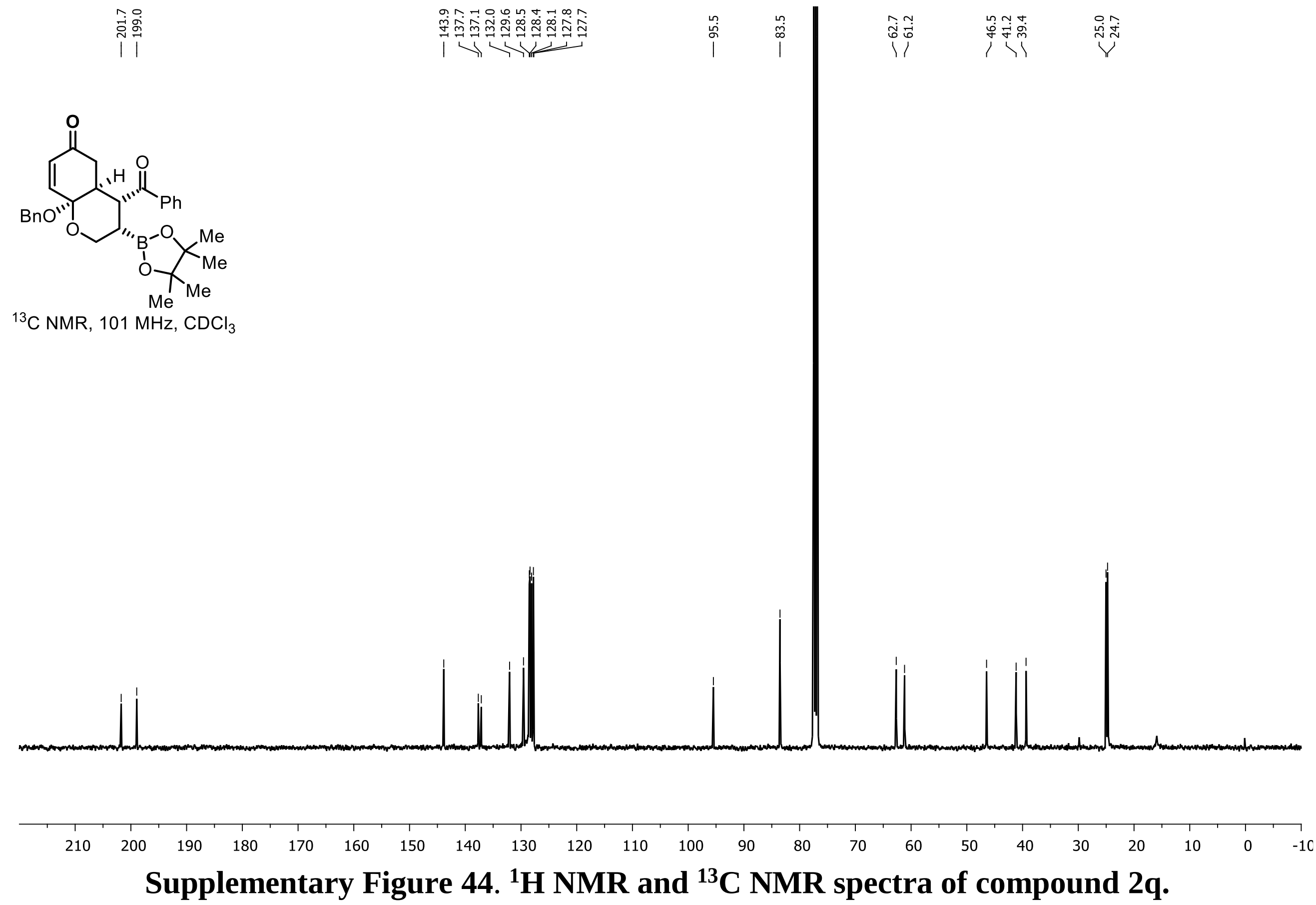

Supplementary Figure 44. **^{1}H NMR and ^{13}C NMR spectra of compound 2q.**

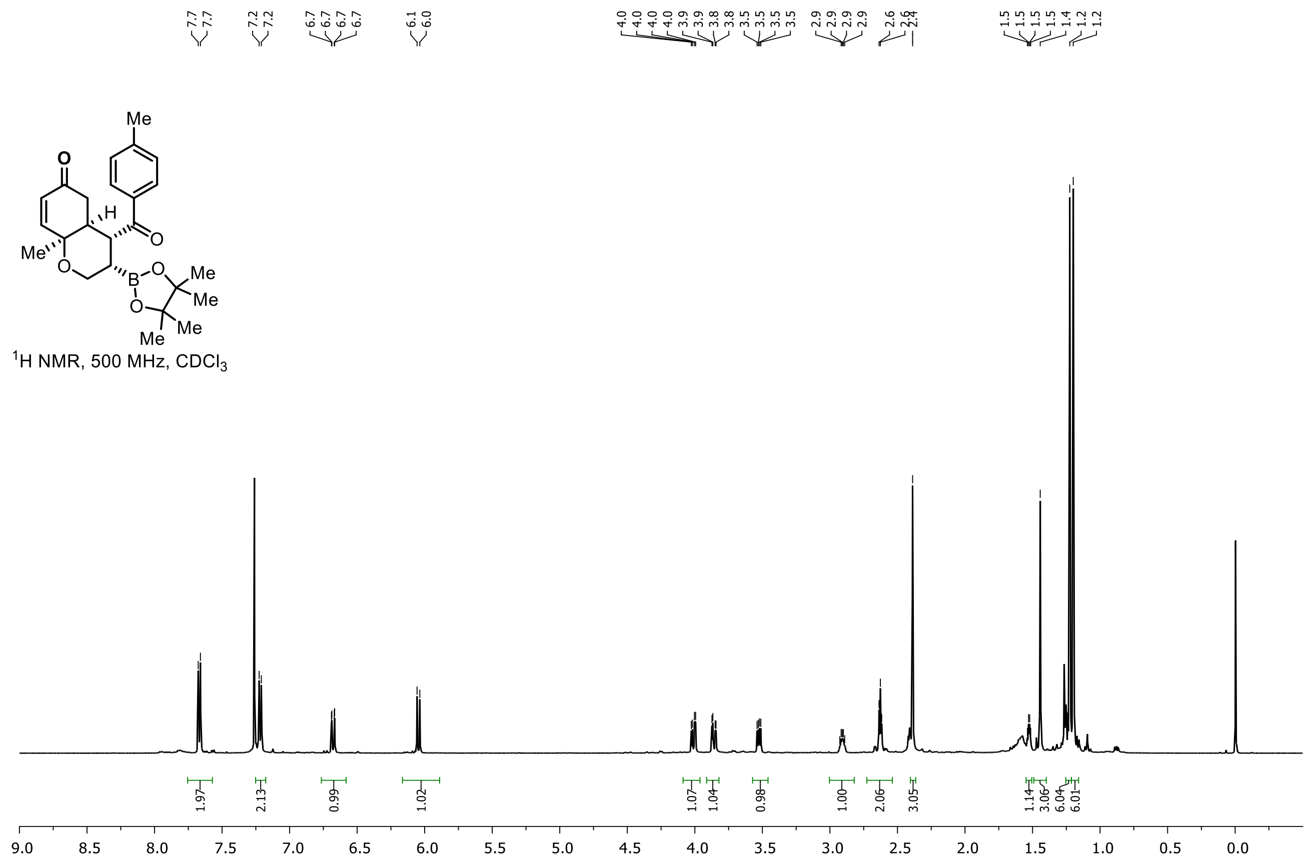

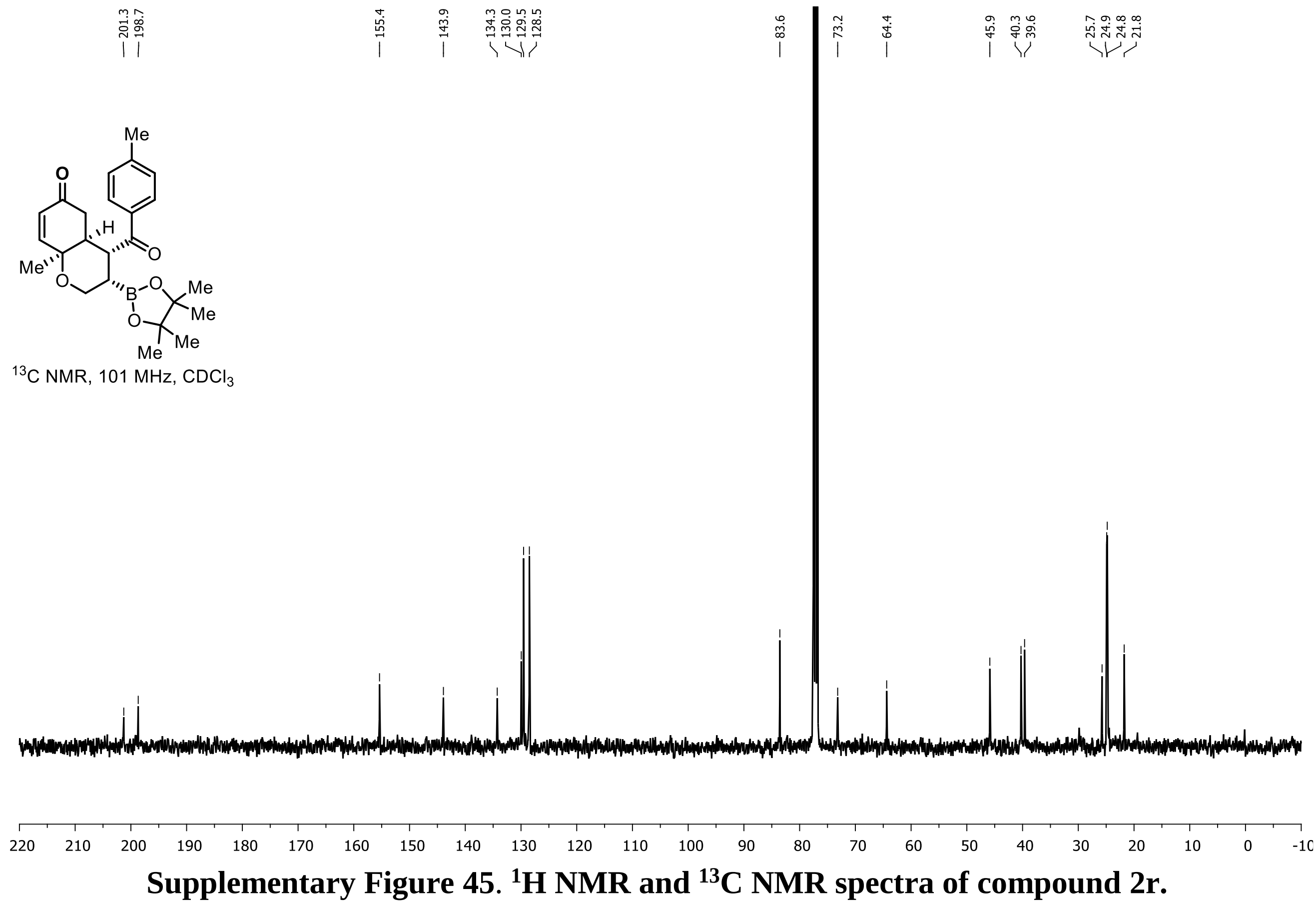

Supplementary Figure 45. ^{1}H NMR and ^{13}C NMR spectra of compound 2r.

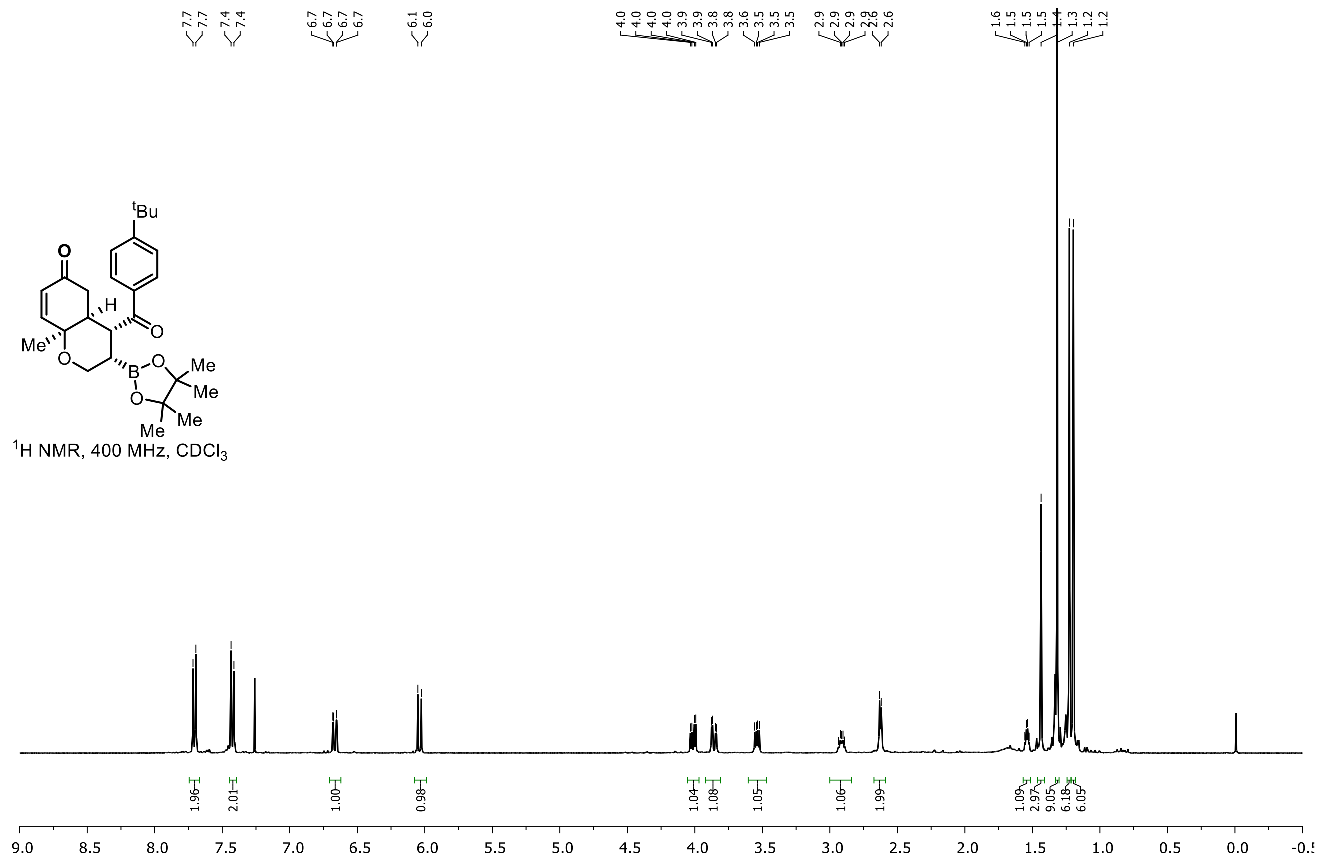

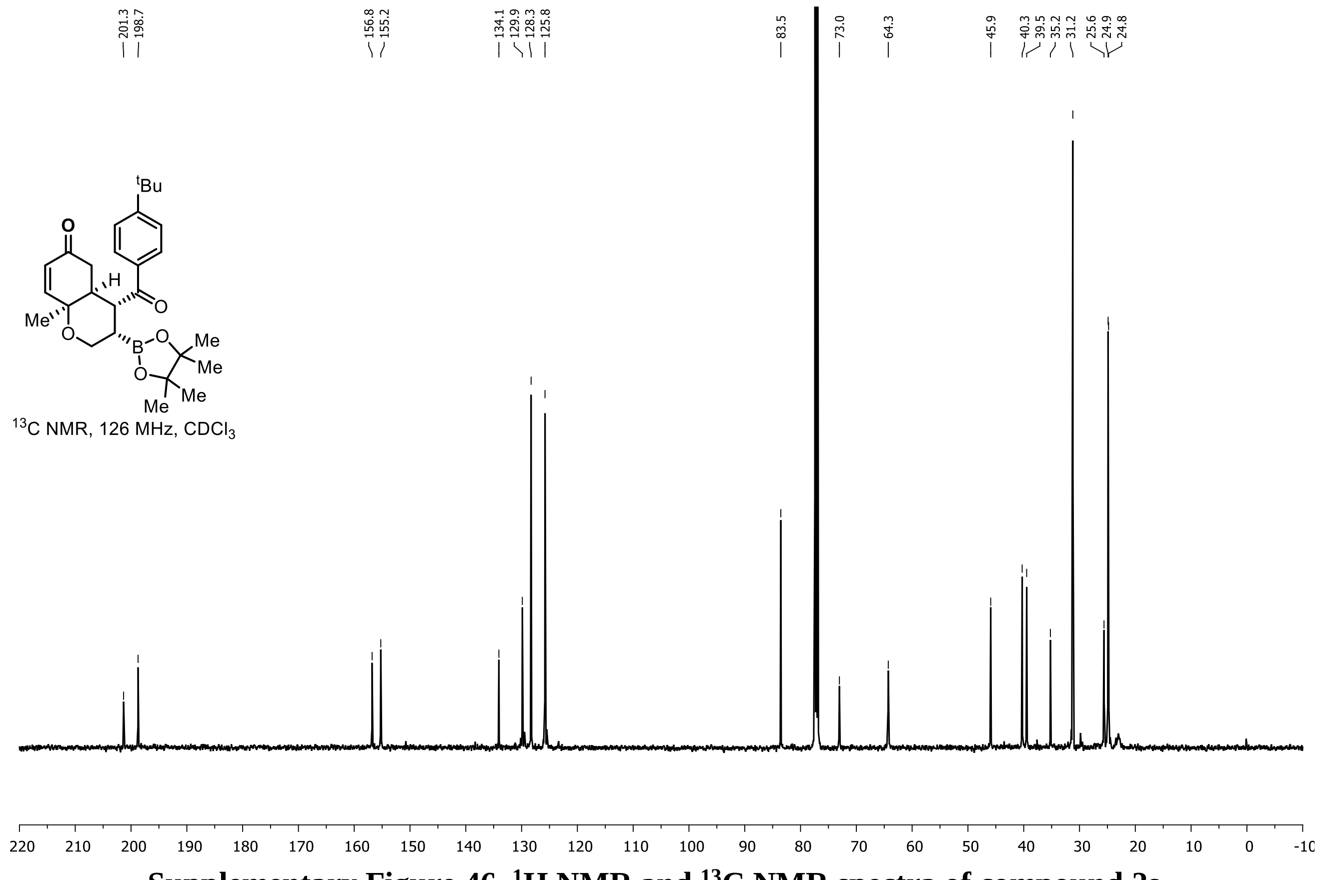

Supplementary Figure 46. **^{1}H NMR and ^{13}C NMR spectra of compound 2s.**

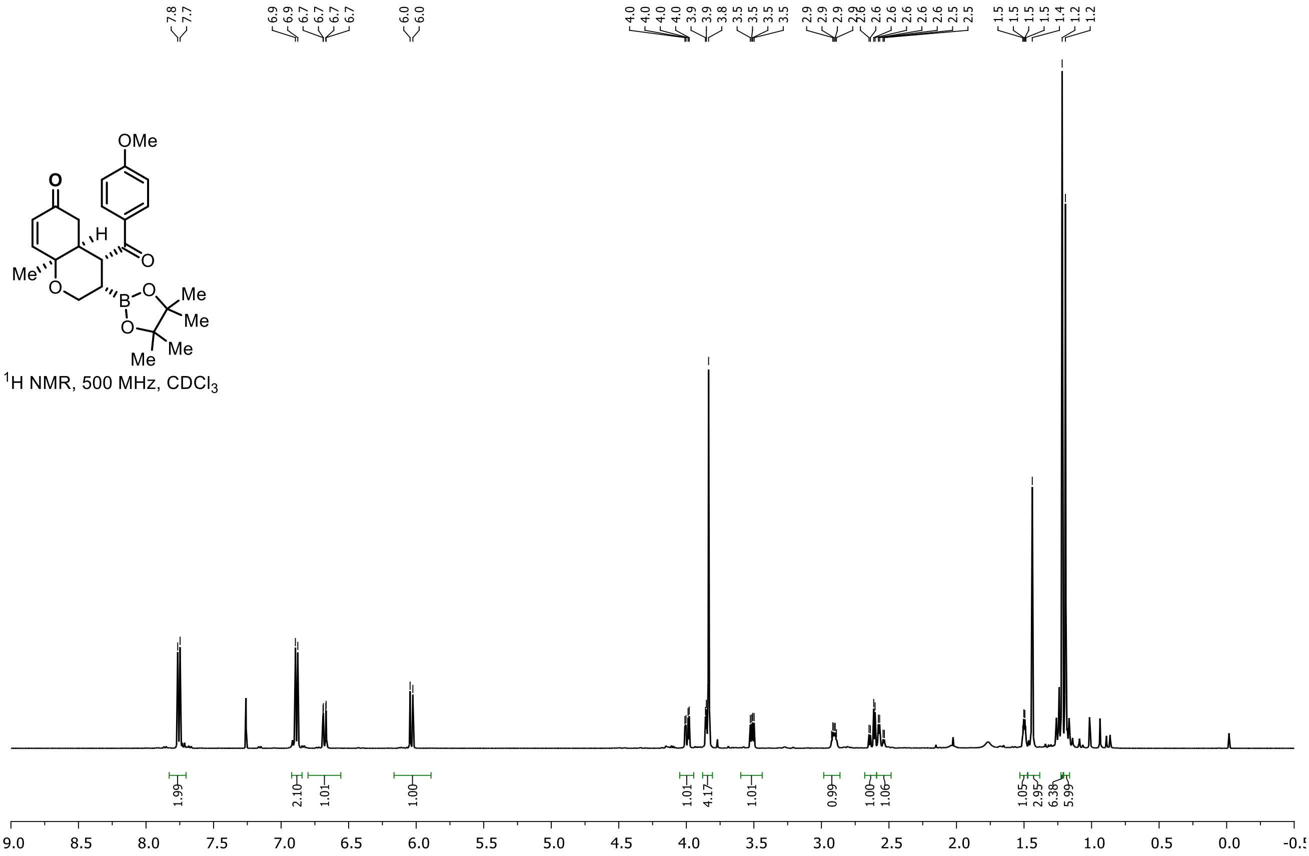

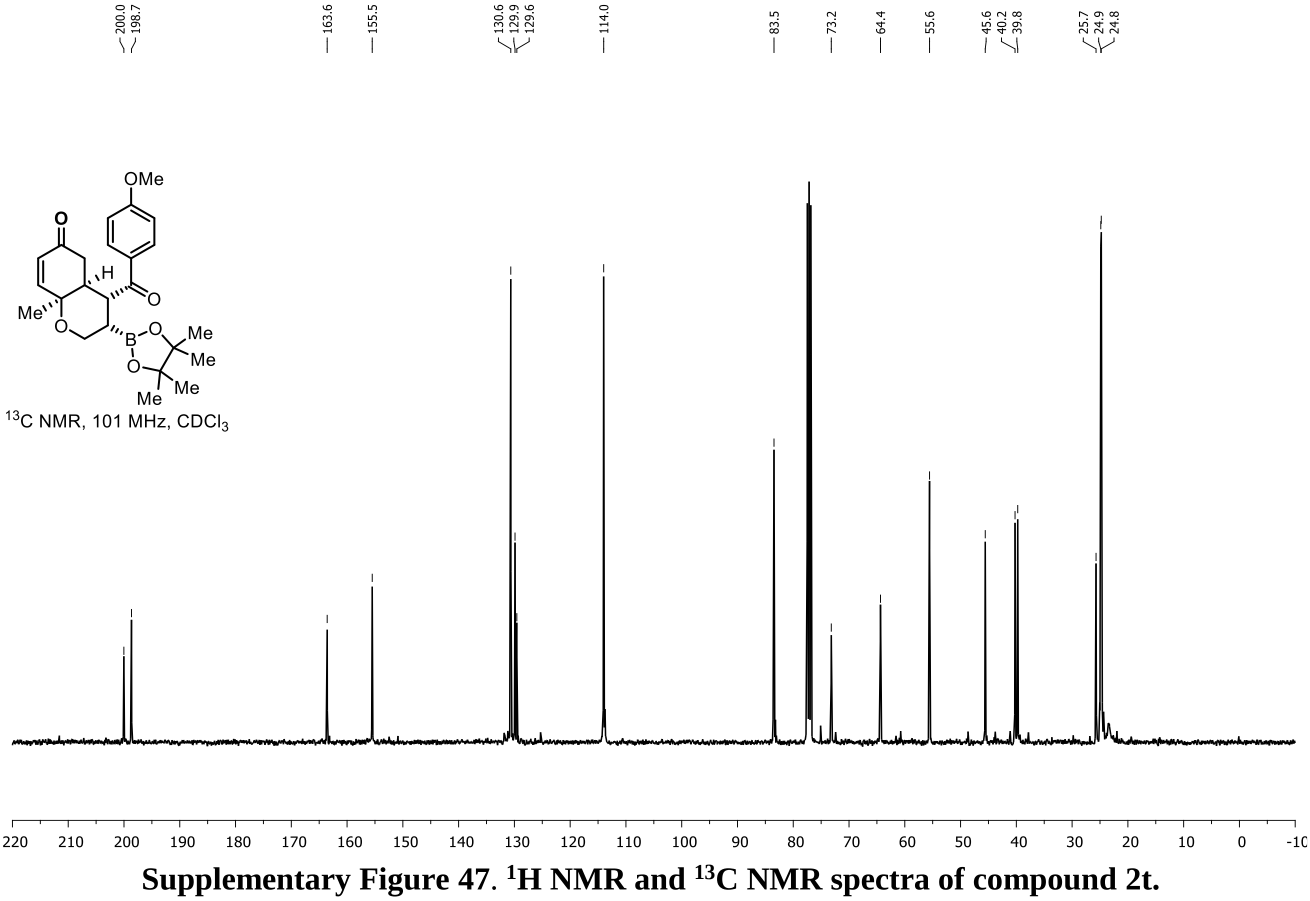

Supplementary Figure 47. **^{1}H NMR and ^{13}C NMR spectra of compound 2t.**

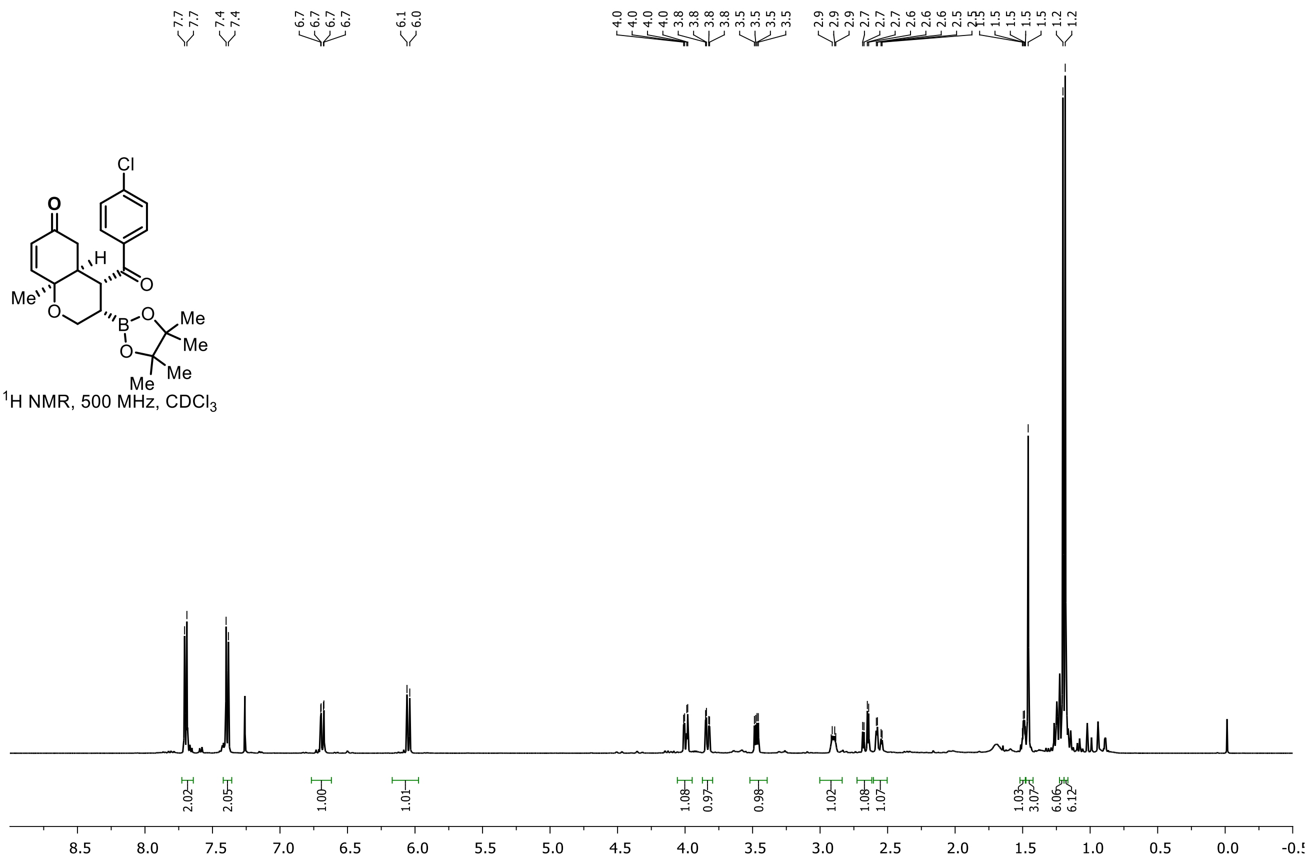

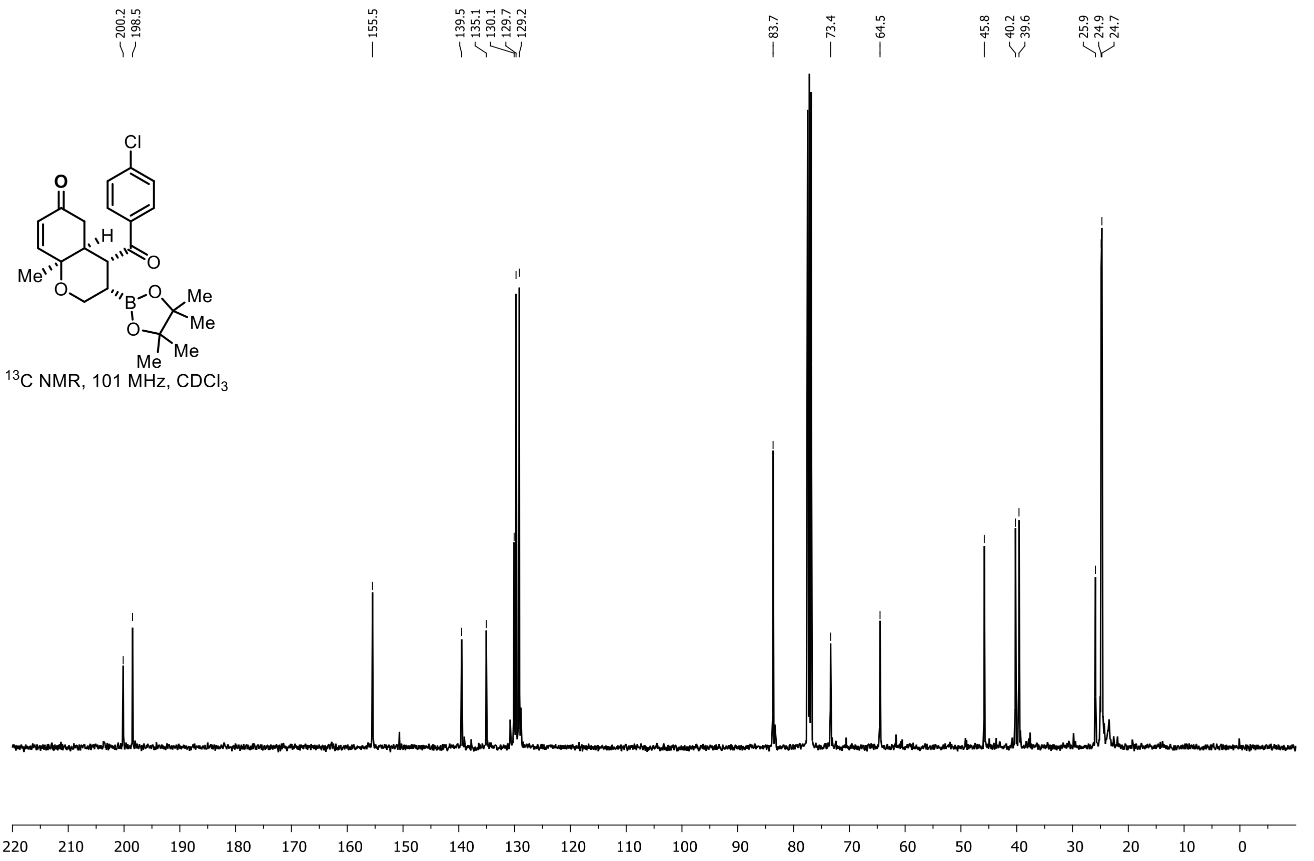

Supplementary Figure 48. ^{1}H NMR and ^{13}C NMR spectra of compound 2u.

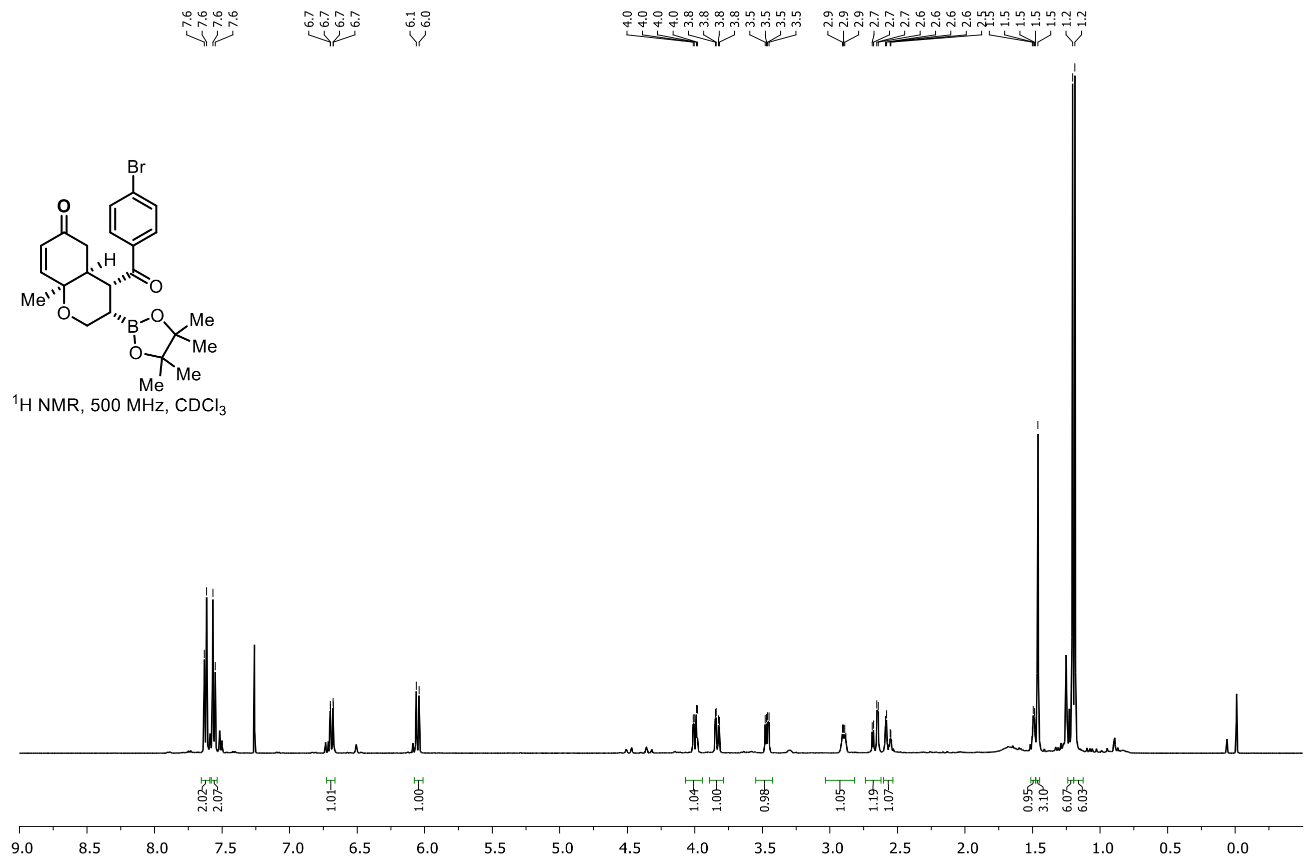

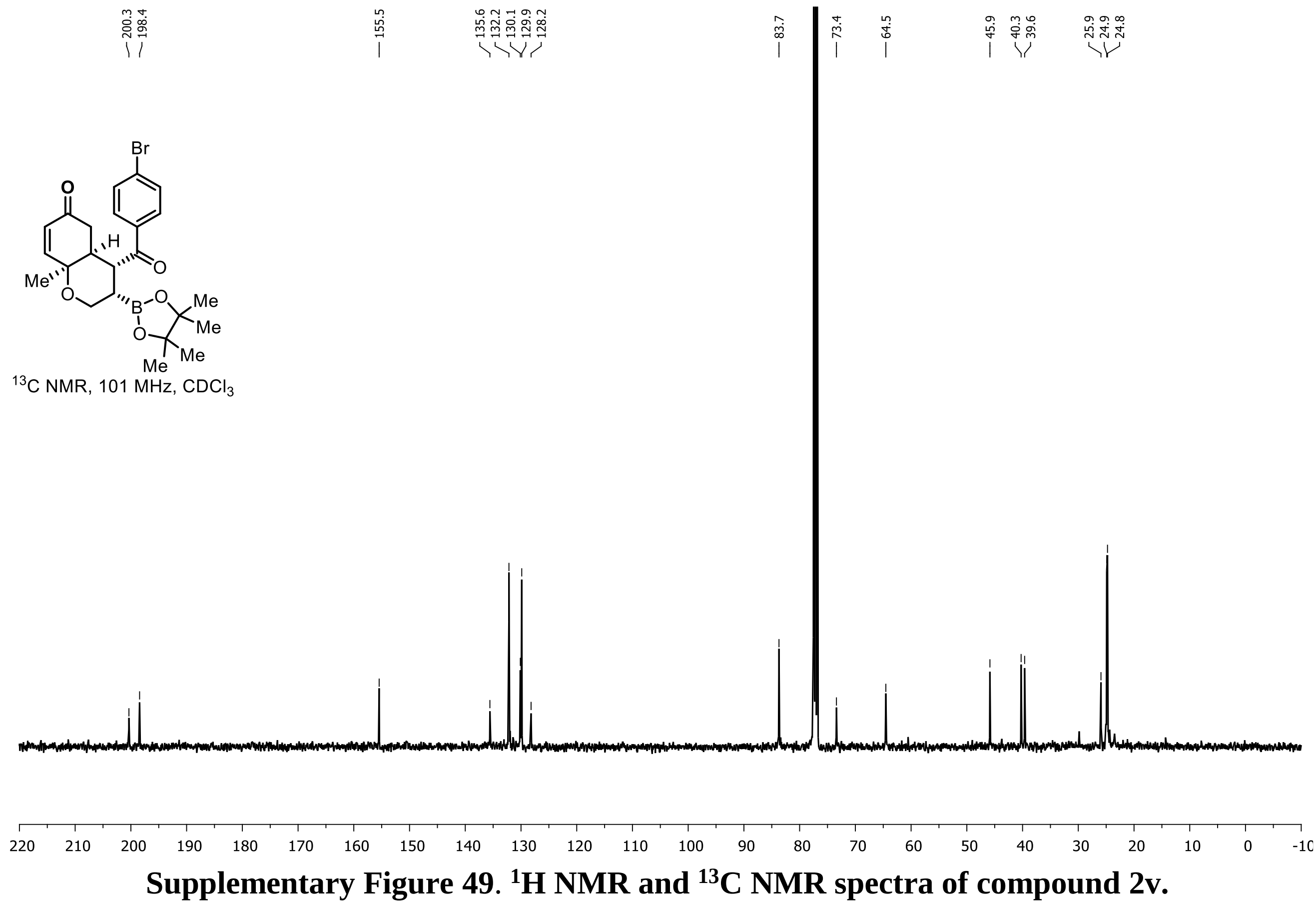

Supplementary Figure 49. **^{1}H NMR and ^{13}C NMR spectra of compound 2v.**

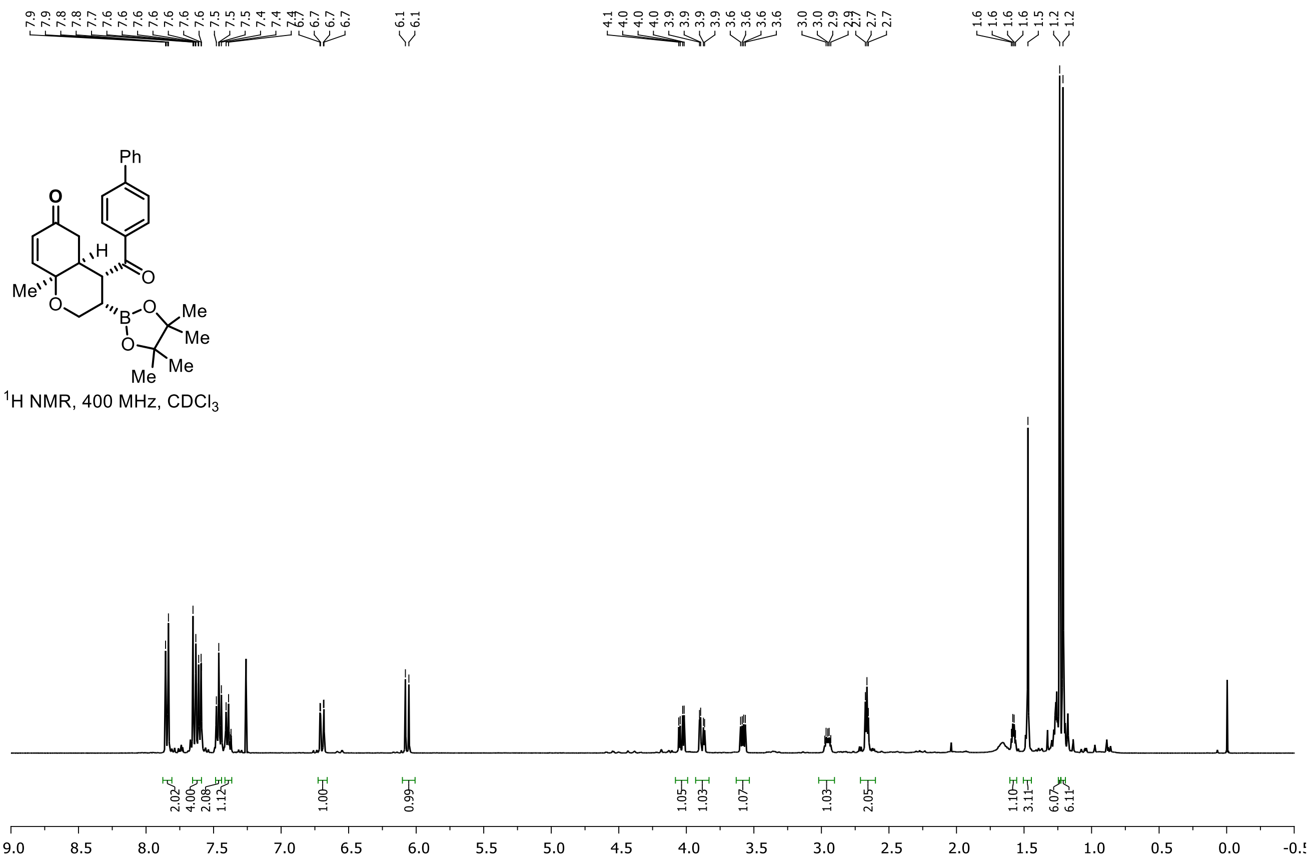

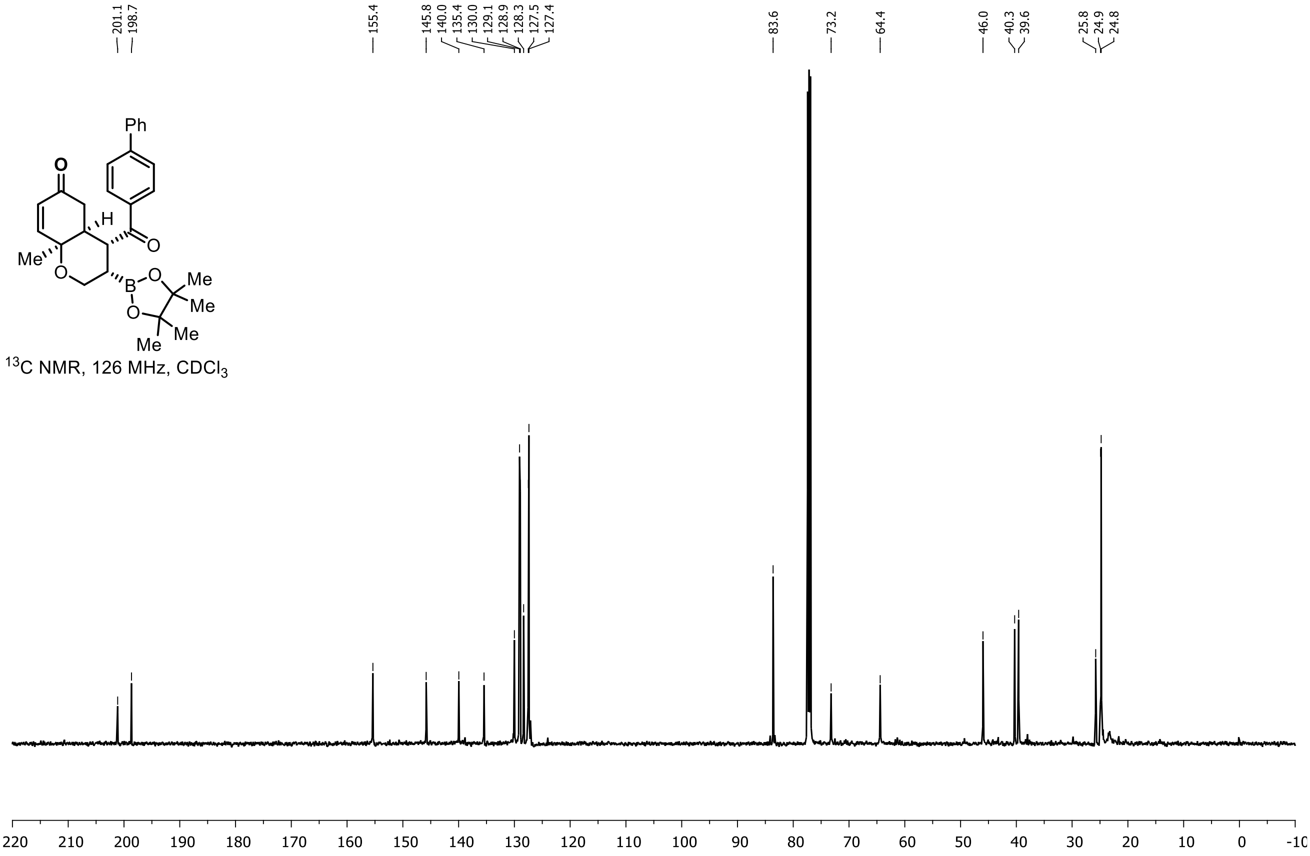

Supplementary Figure 50. ^{1}H NMR and ^{13}C NMR spectra of compound 2w.

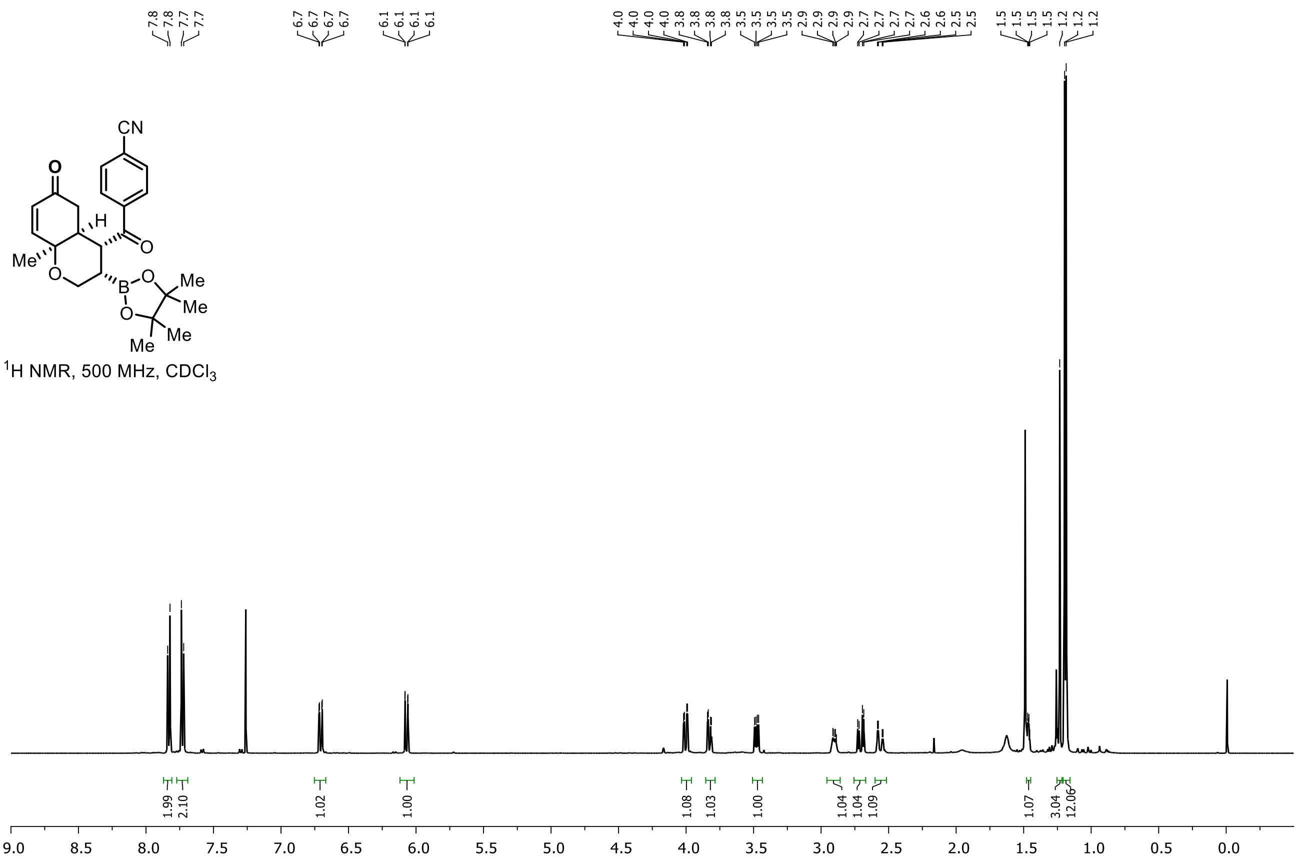

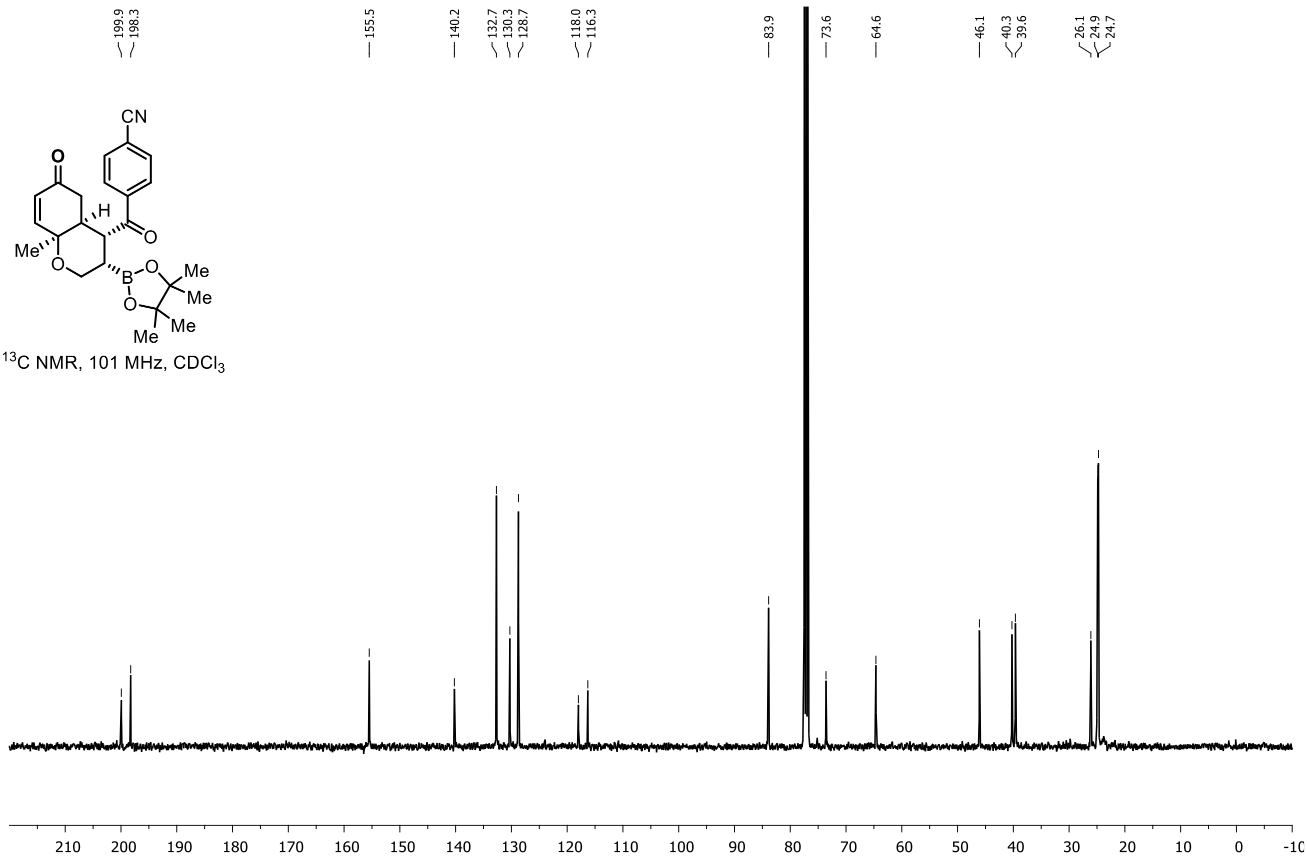

Supplementary Figure 51. **^{1}H NMR and ^{13}C NMR spectra of compound 2x.**

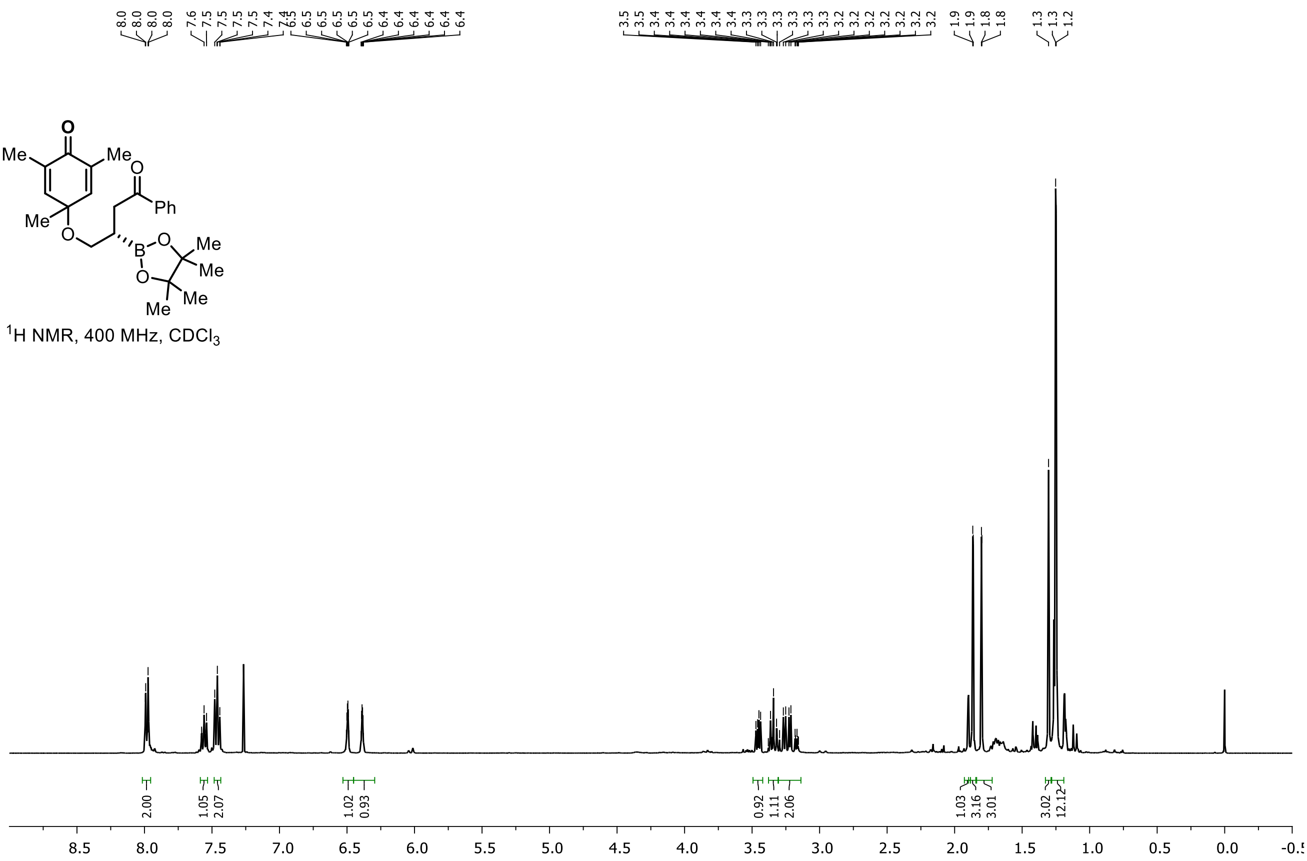

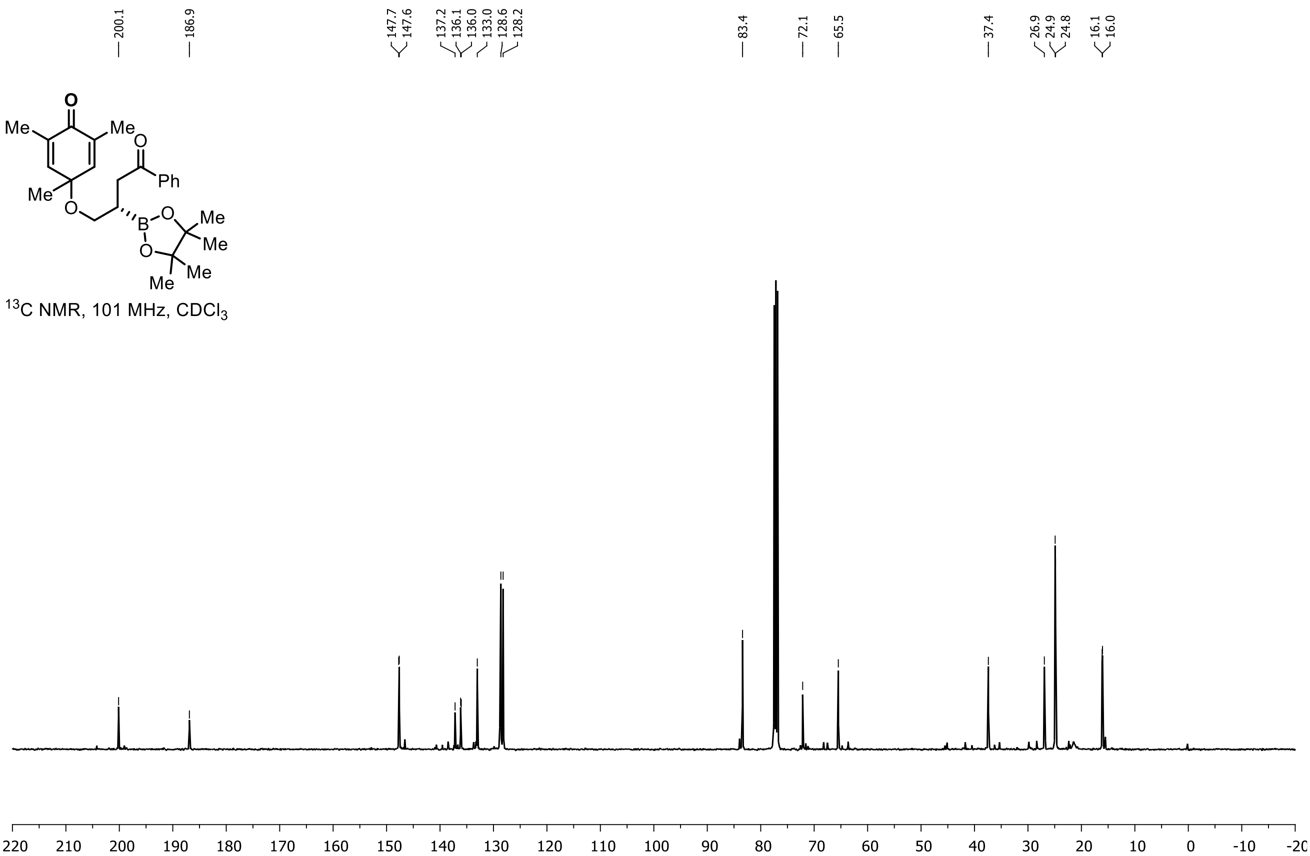

Supplementary Figure 52. **^{1}H NMR and ^{13}C NMR spectra of compound 2zb.**

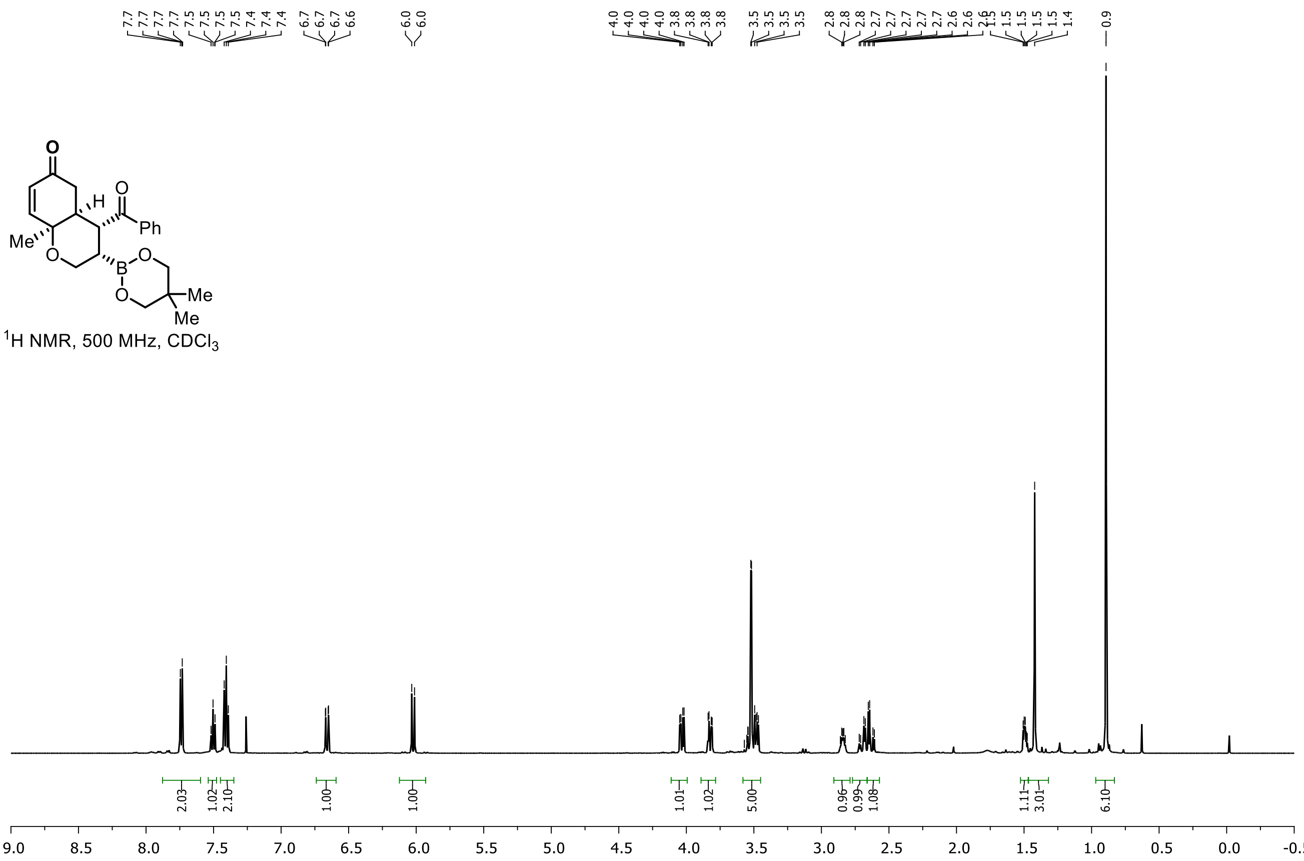

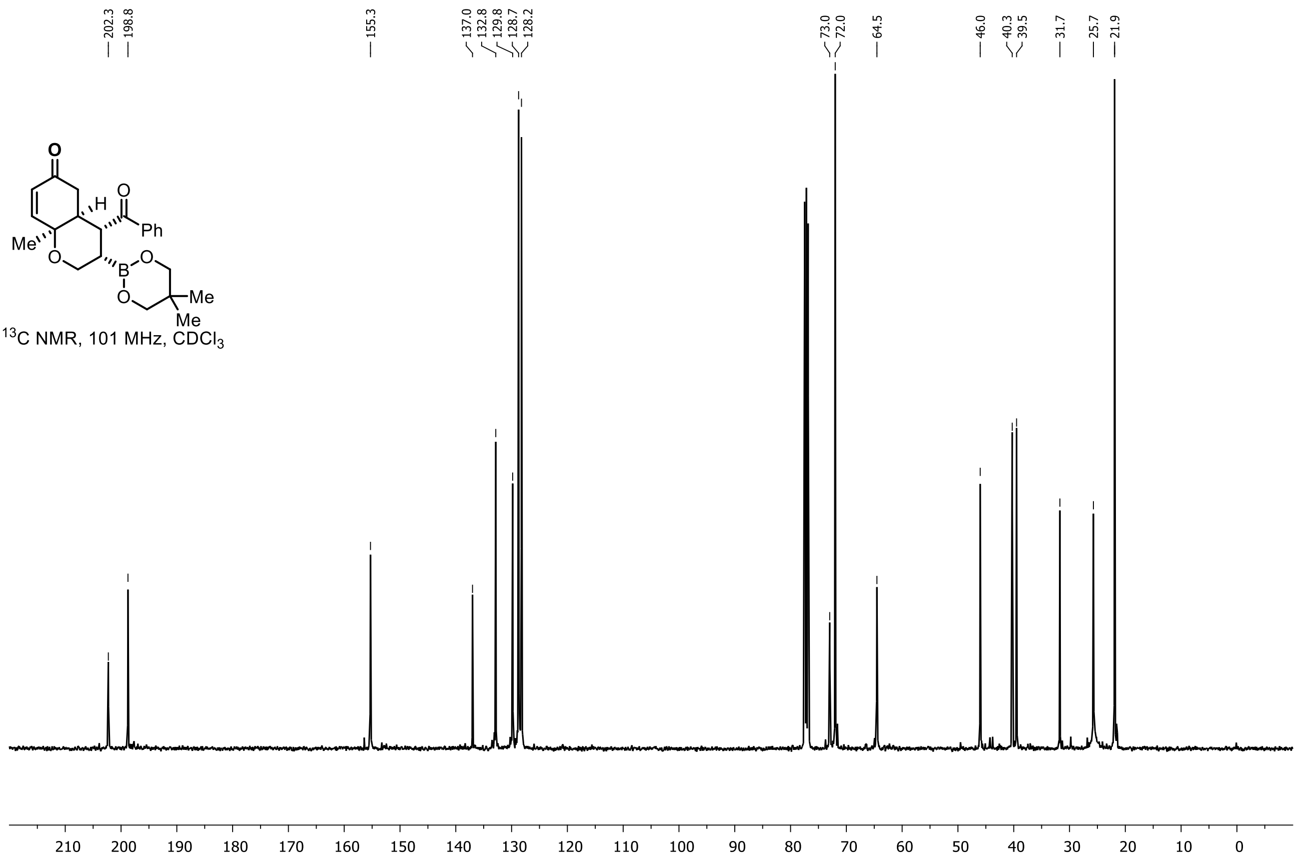

Supplementary Figure 53. **^{1}H NMR and ^{13}C NMR spectra of compound 2zc.**

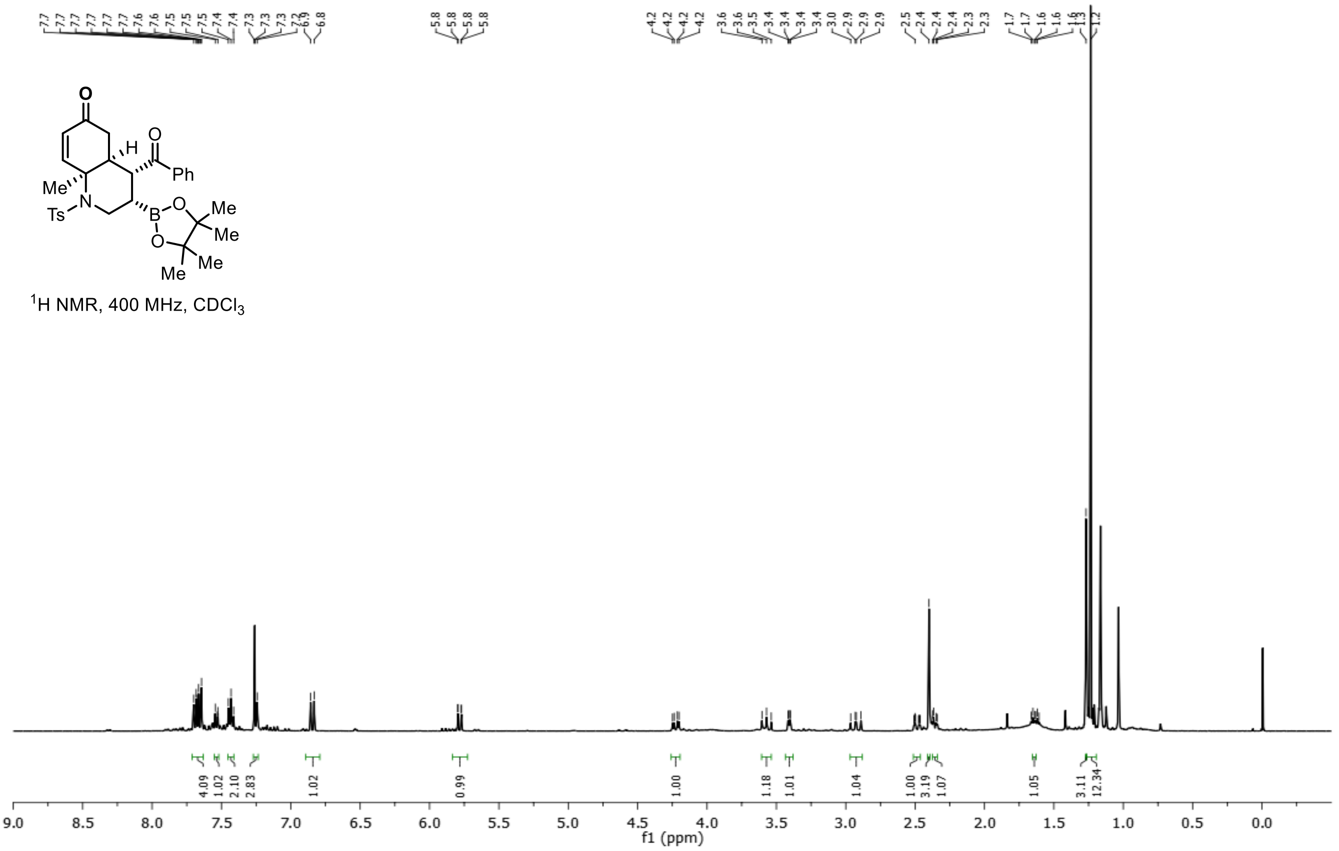

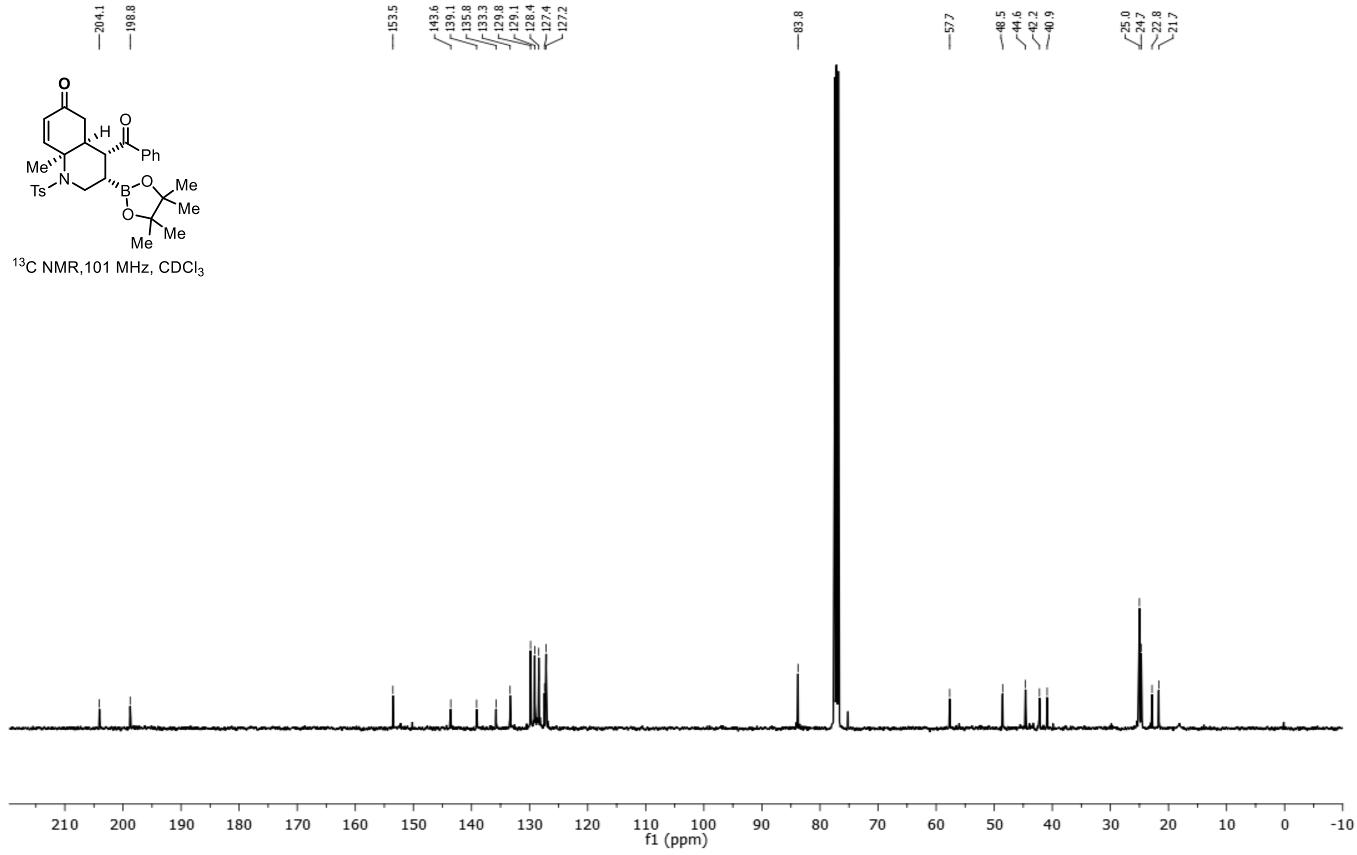

Supplementary Figure 54. **^{1}H NMR and ^{13}C NMR spectra of compound 2zd.**

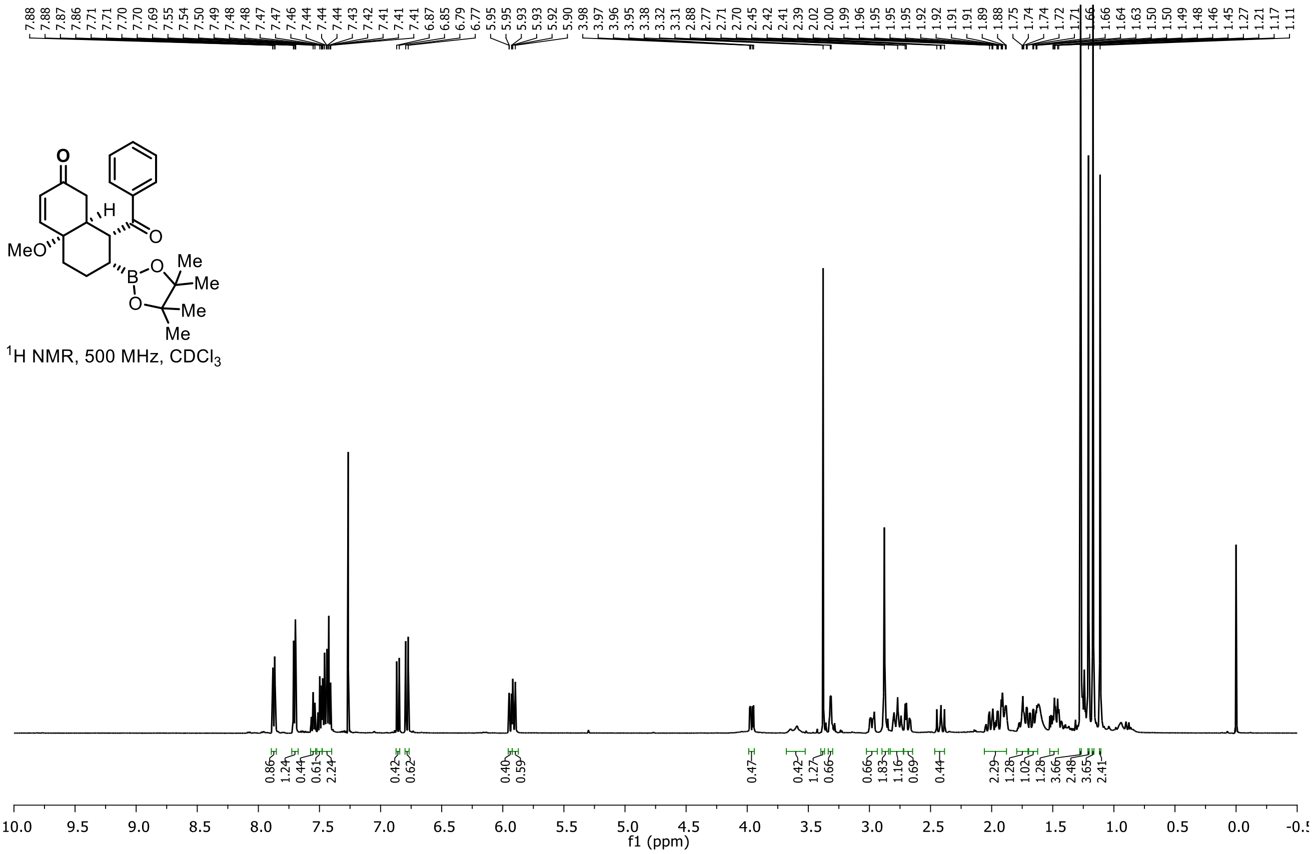

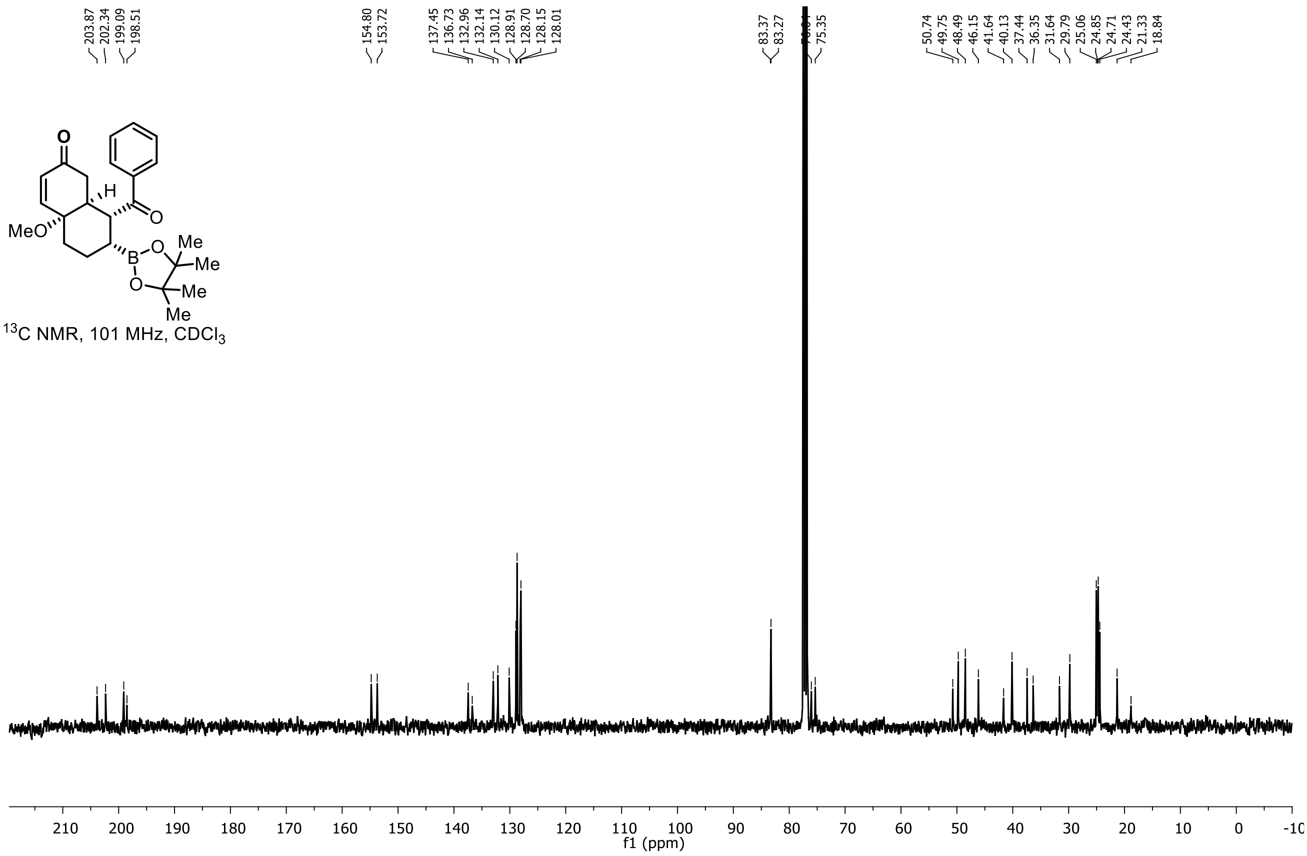

Supplementary Figure 55. ^{1}H NMR and ^{13}C NMR spectra of compound 5.

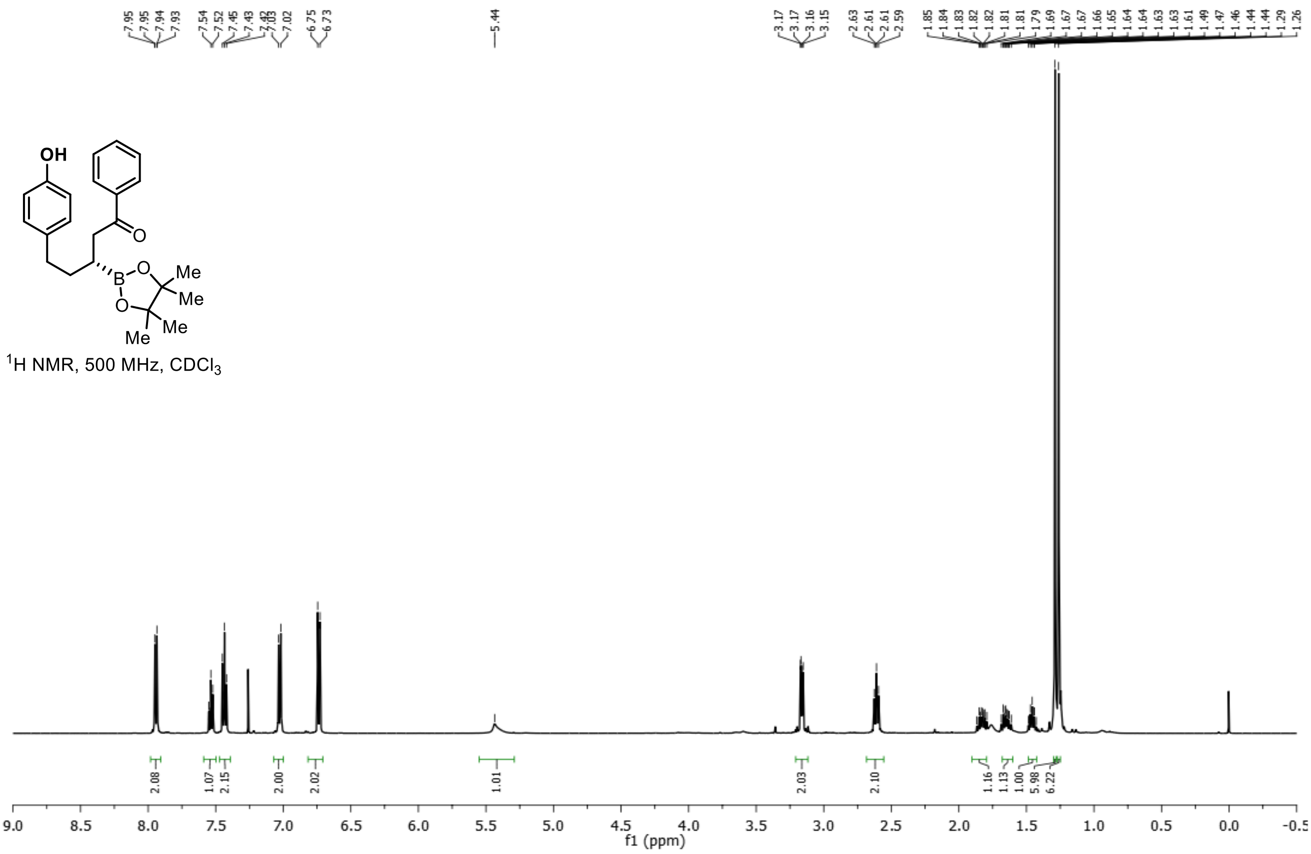

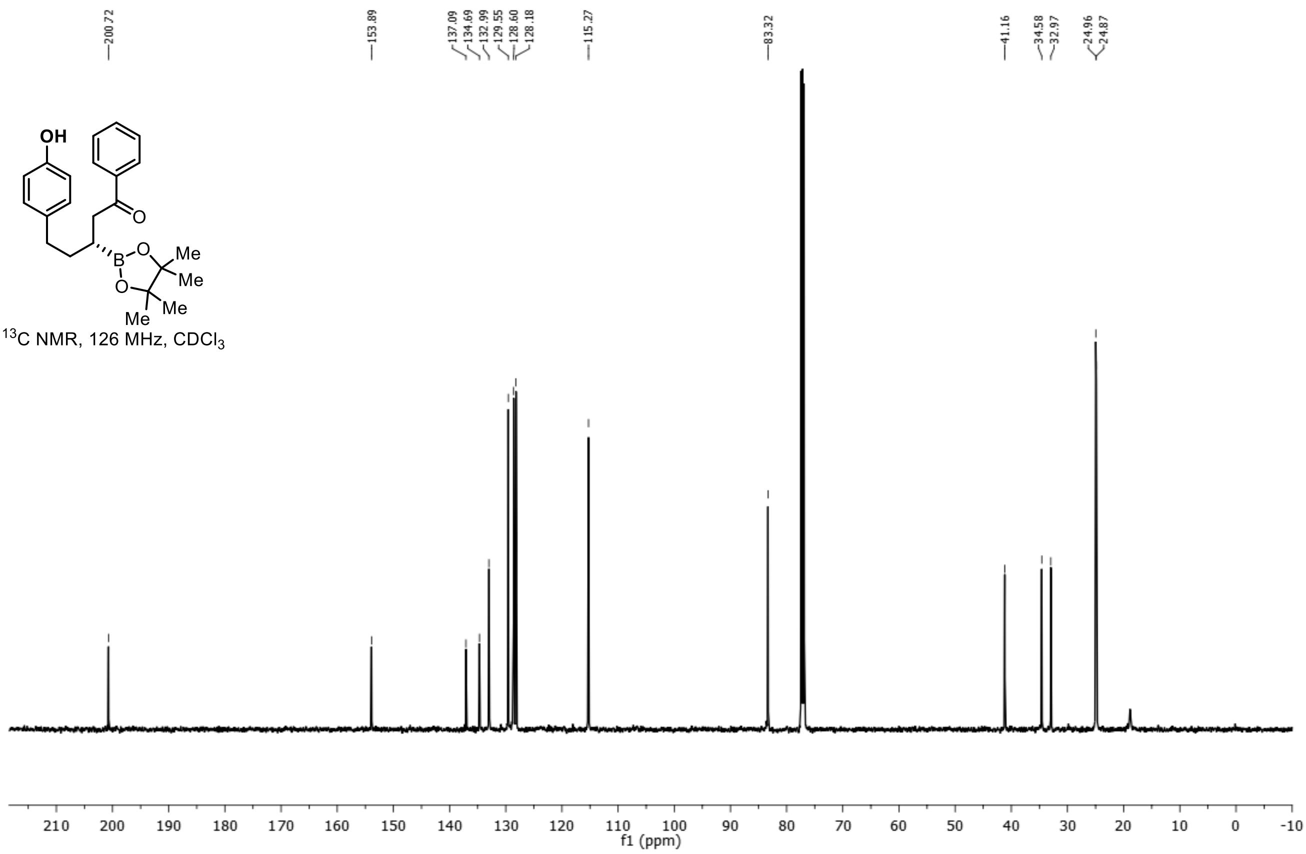

Supplementary Figure 56. ^{1}H NMR and ^{13}C NMR spectra of compound 6.

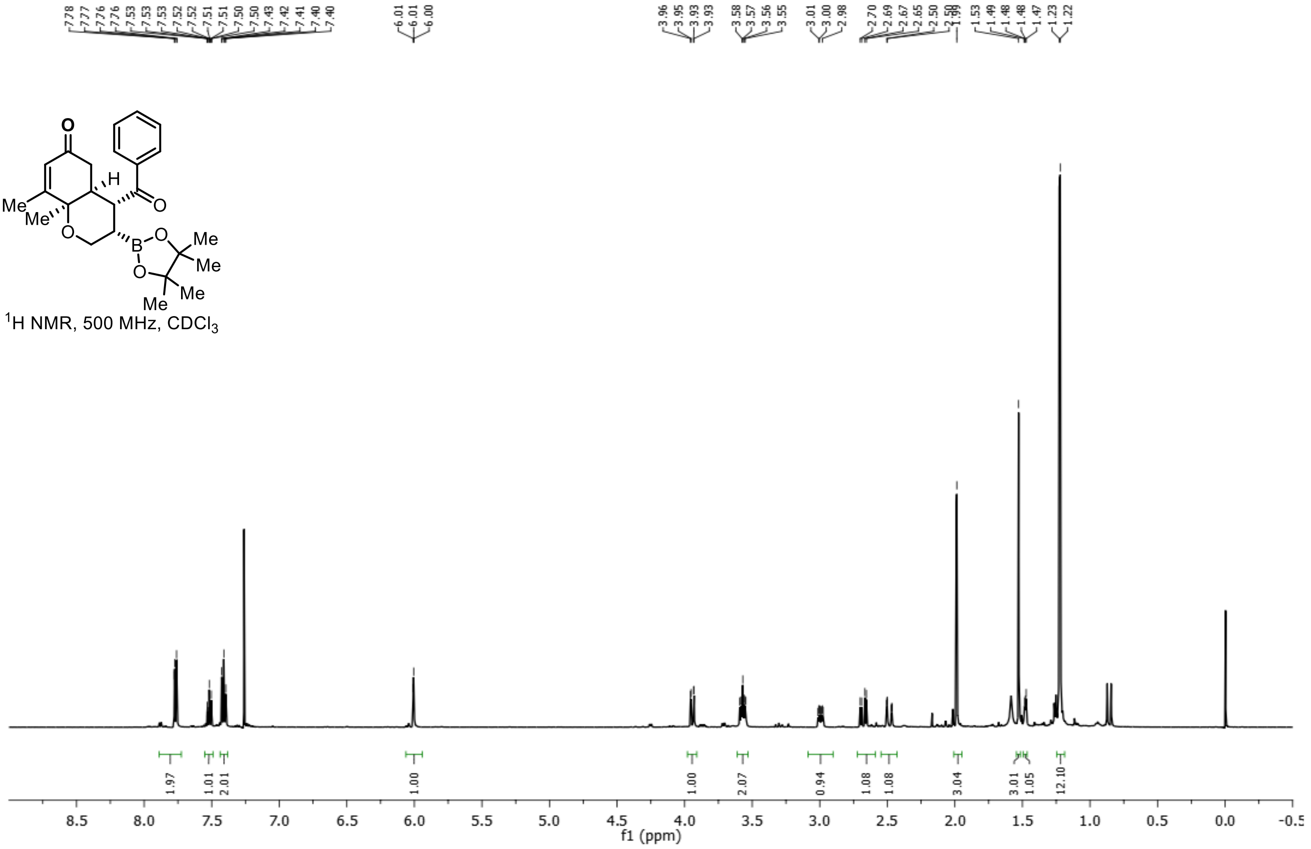

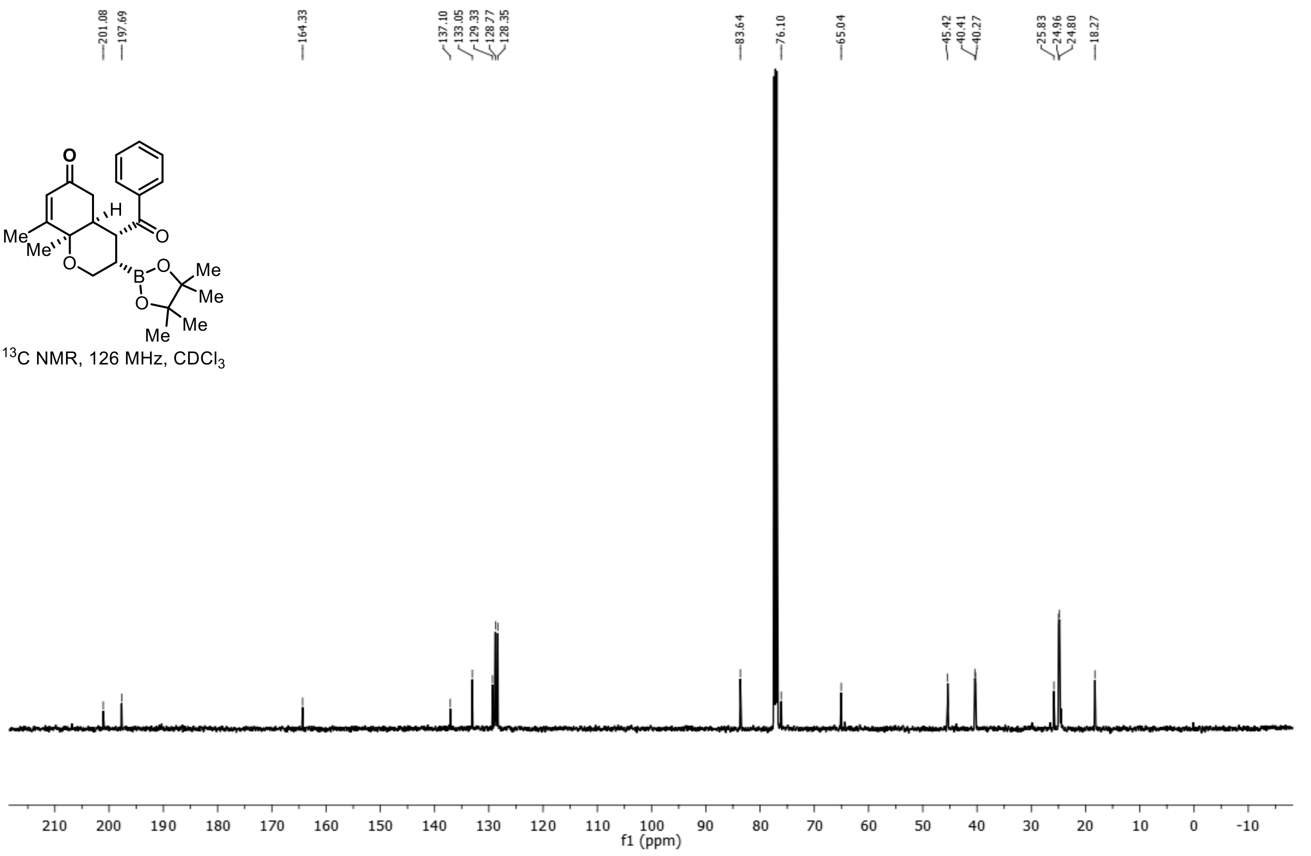

Supplementary Figure 57. ^{1}H NMR and ^{13}C NMR spectra of compound 8.

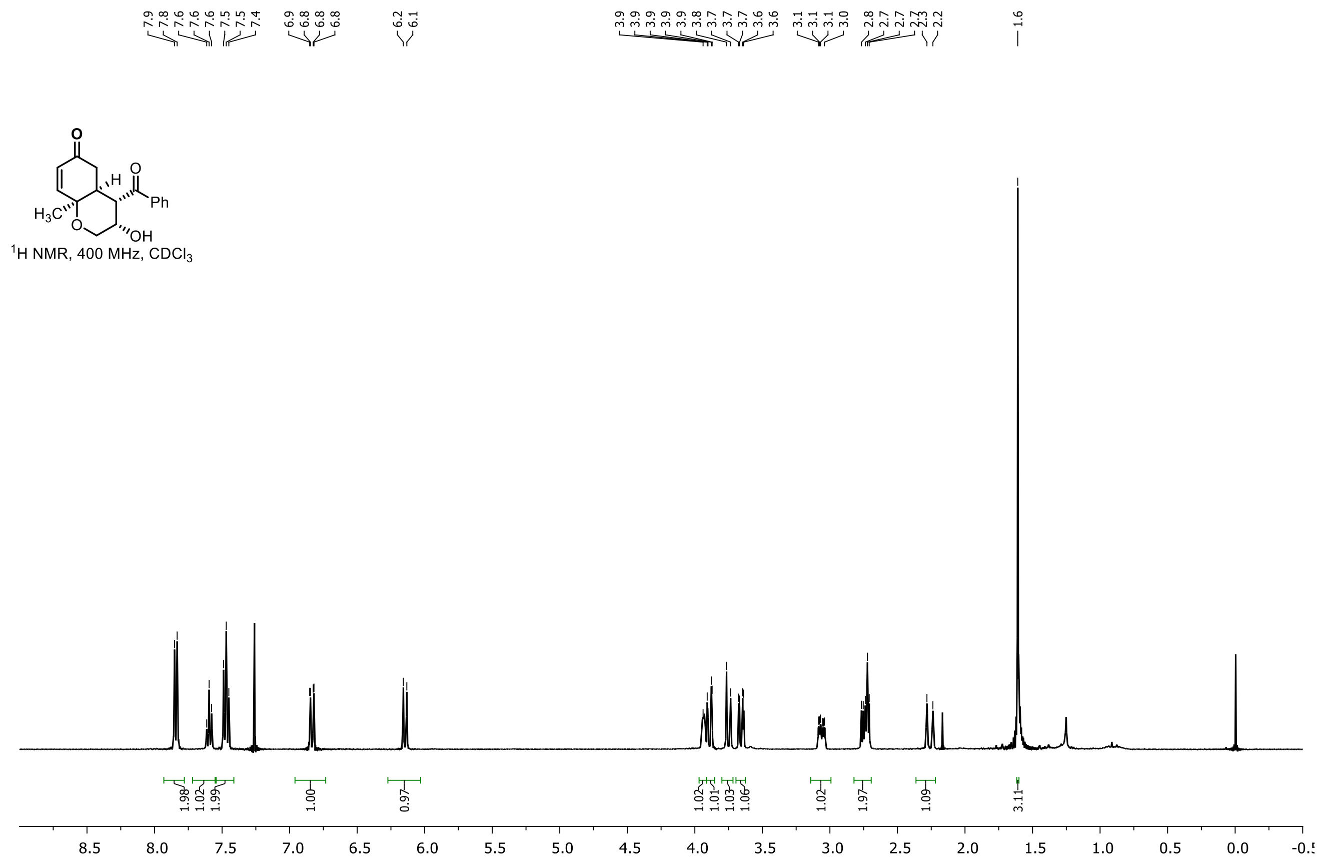

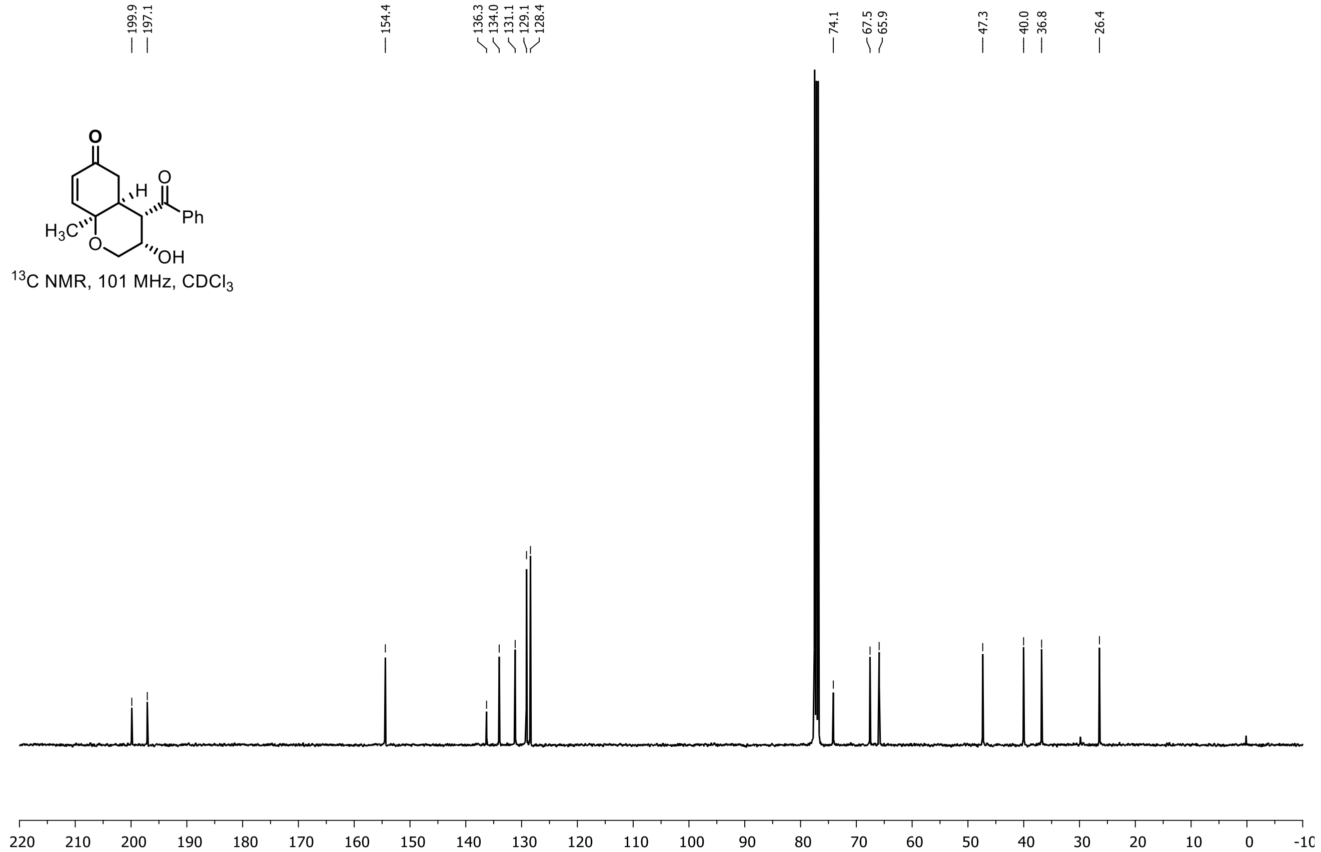

Supplementary Figure 58. **^{1}H NMR and ^{13}C NMR spectra of compound 10a.**

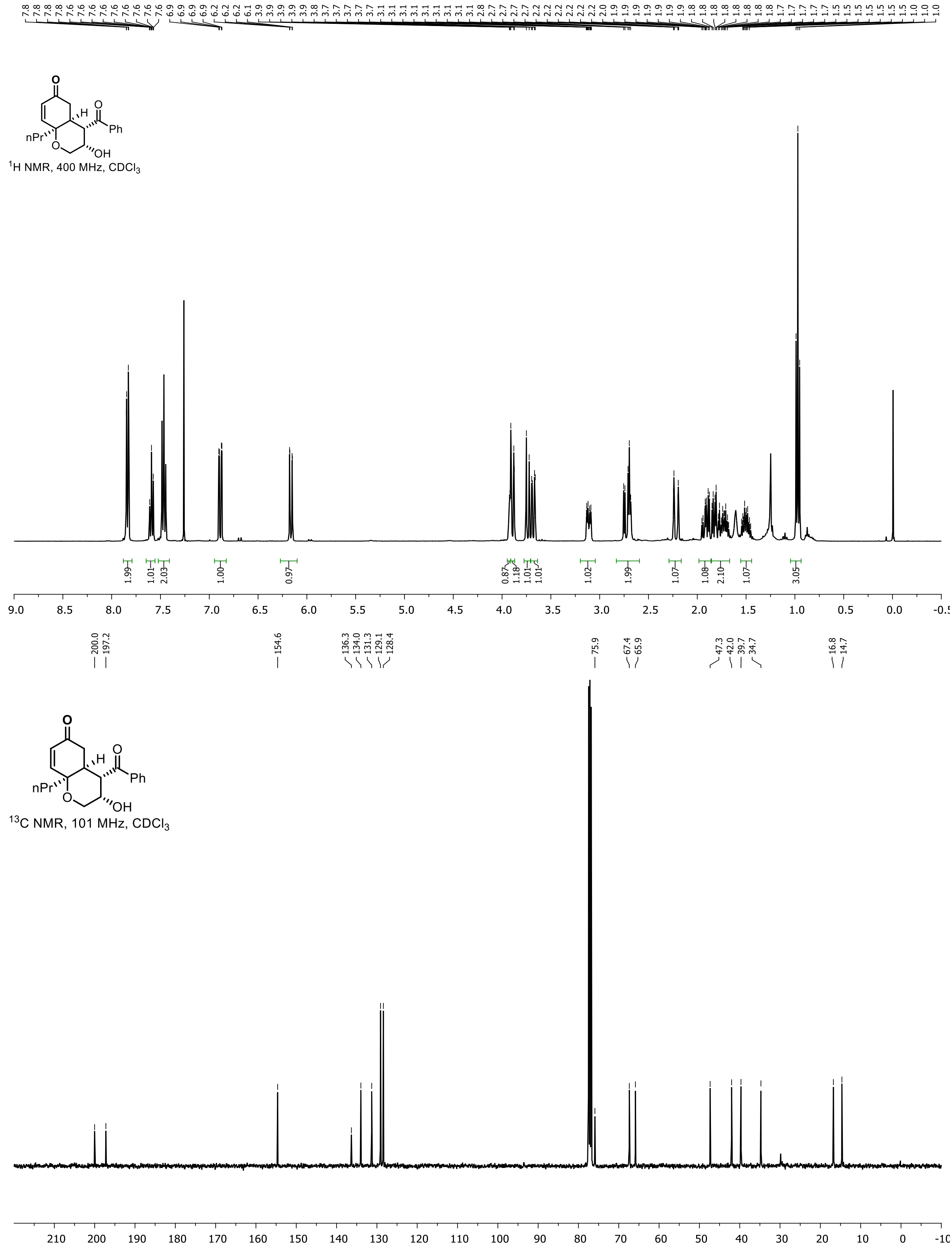

Supplementary Figure 59. **^{1}H NMR and ^{13}C NMR spectra of compound 10b.**

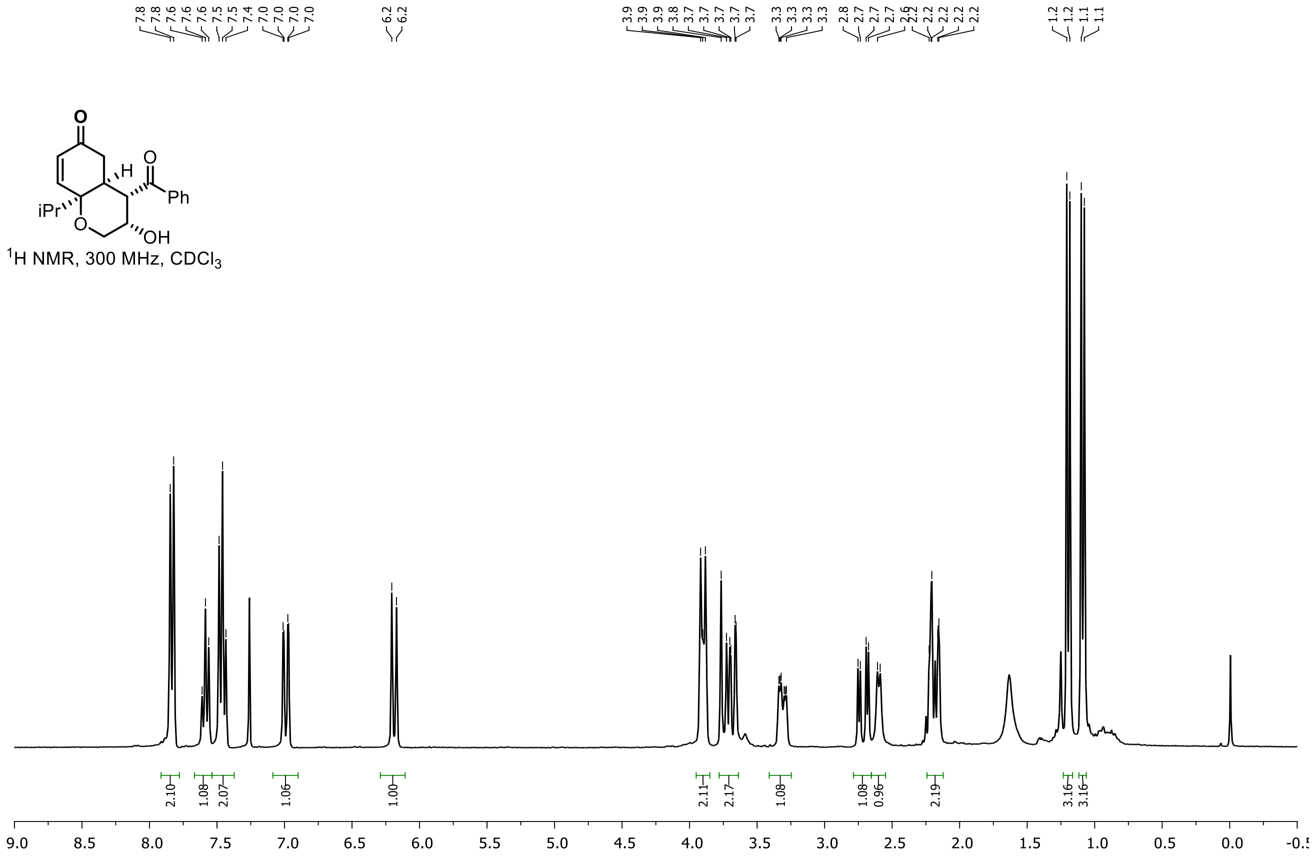

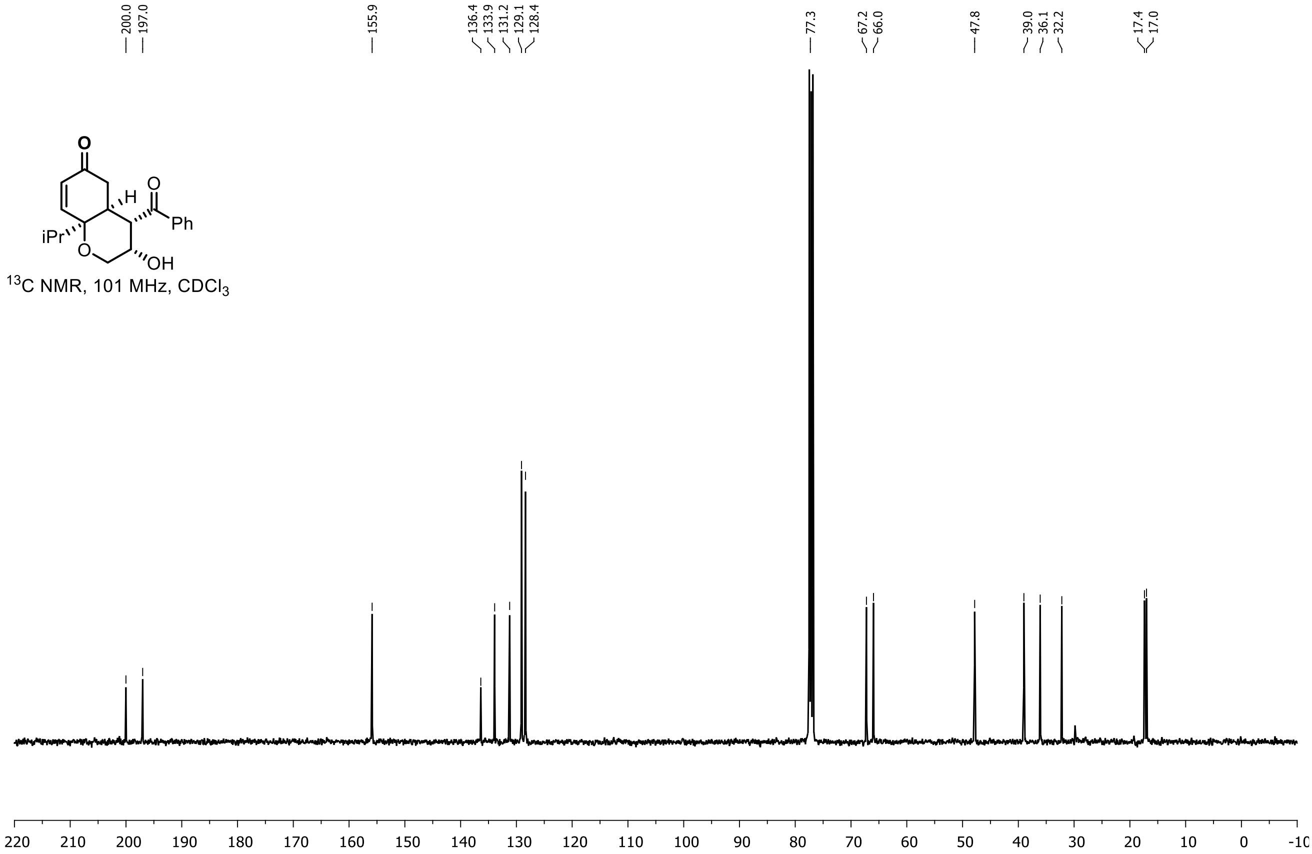

Supplementary Figure 60. **^{1}H NMR and ^{13}C NMR spectra of compound 10c.**

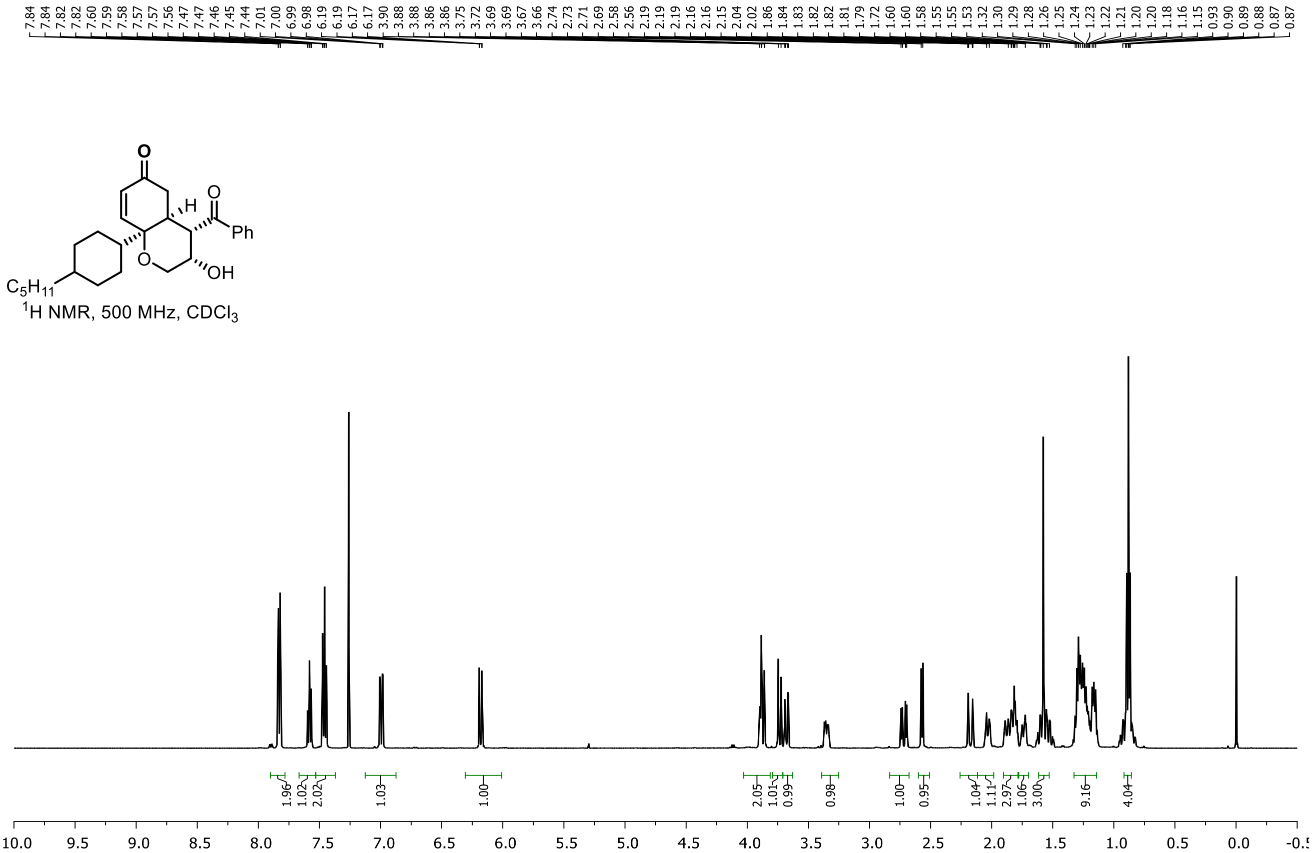

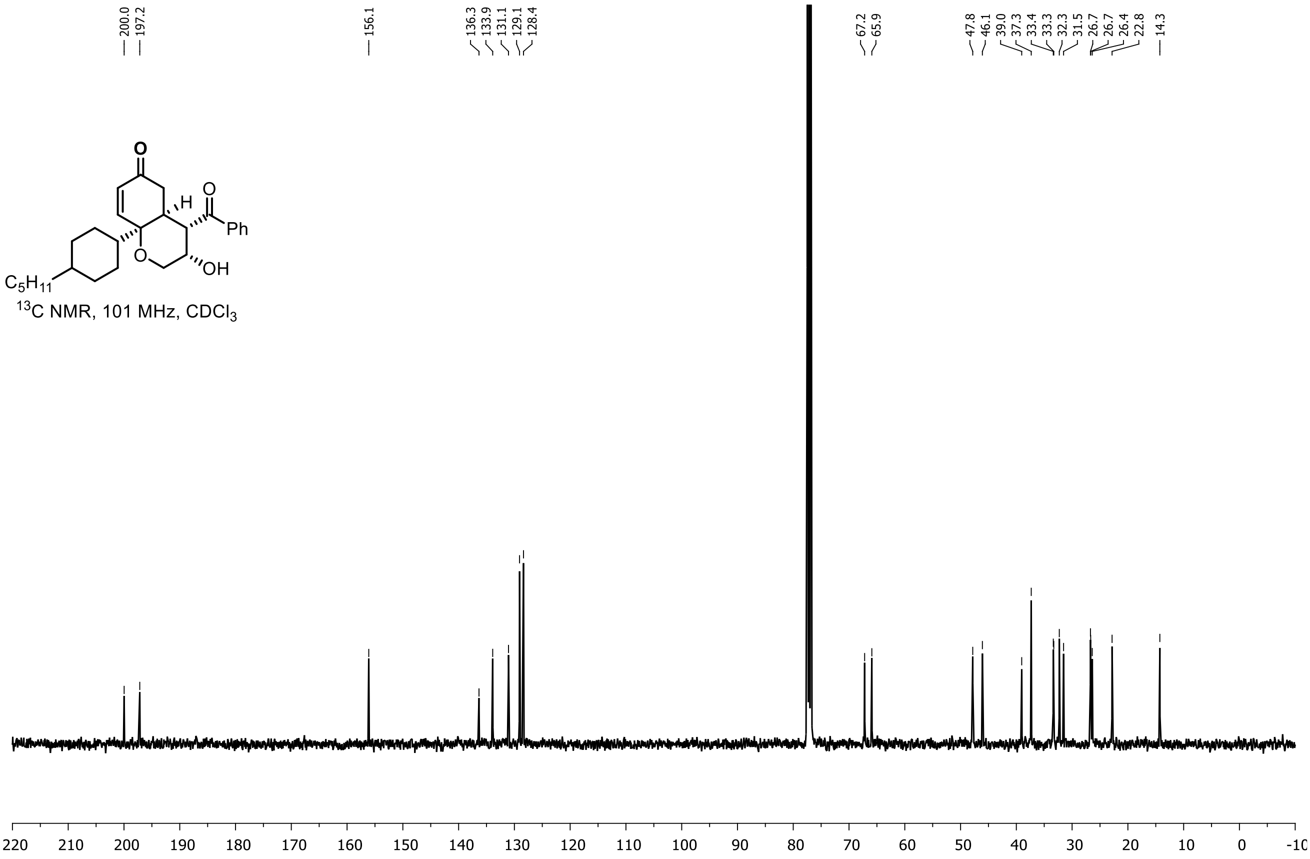

Supplementary Figure 61. ^1H NMR and ^{13}C NMR spectra of compound 10d.

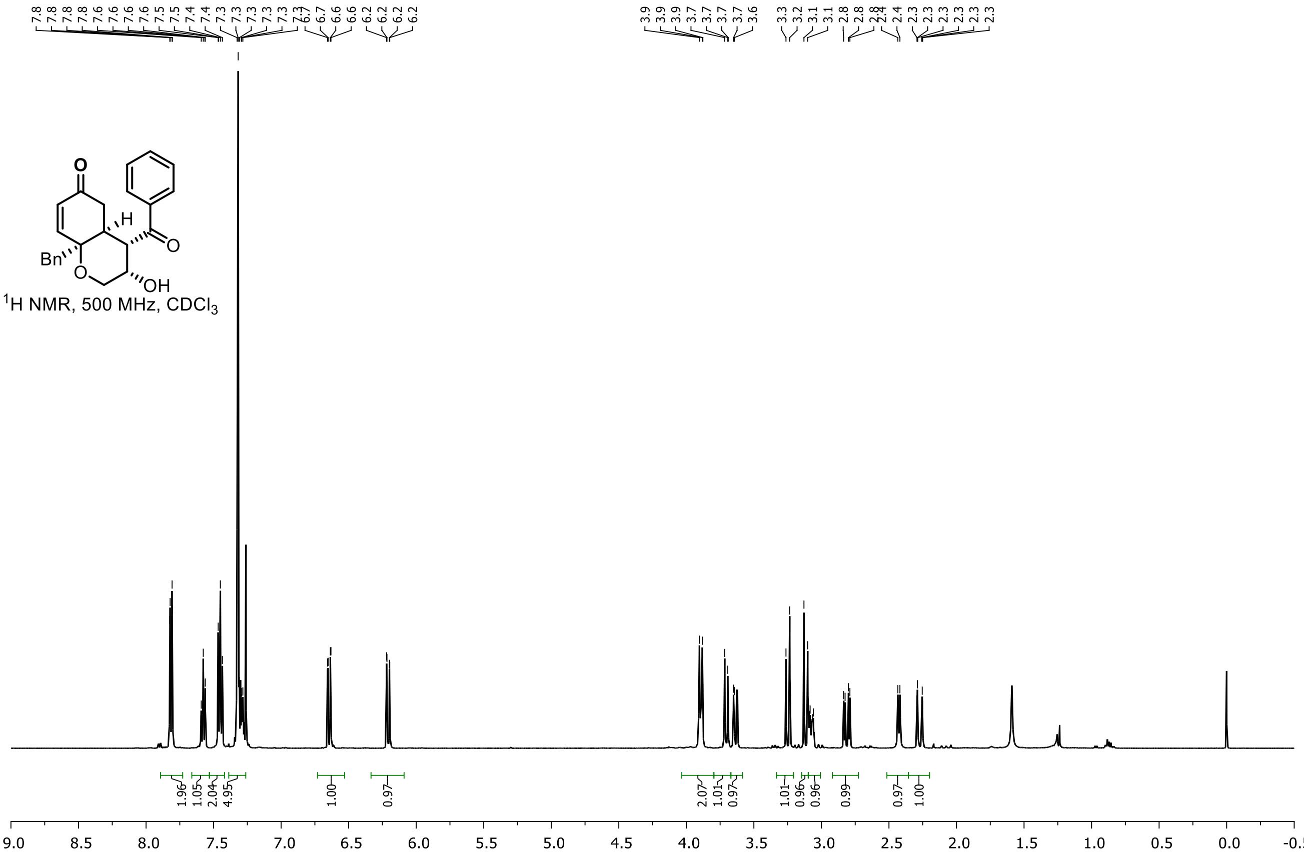

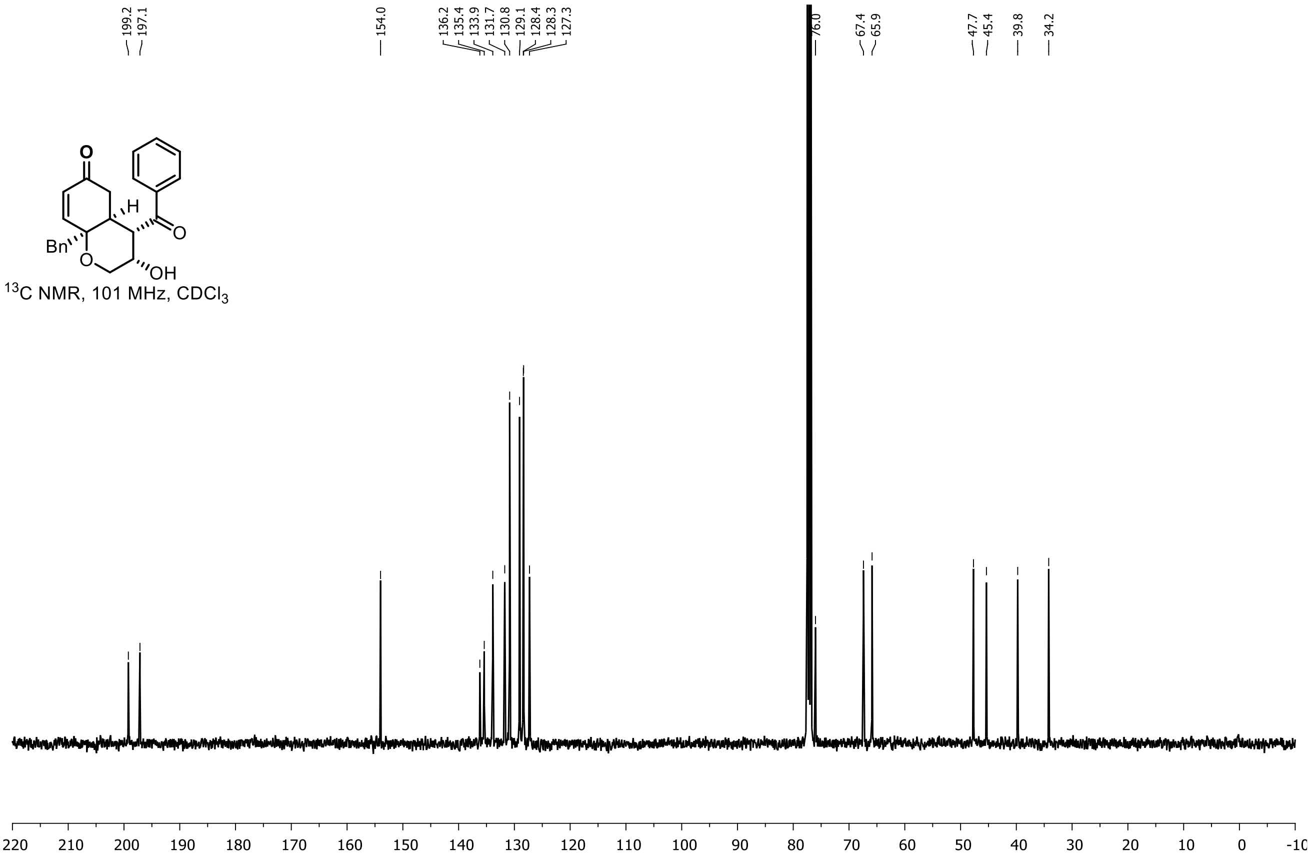

Supplementary Figure 62. **^{1}H NMR and ^{13}C NMR spectra of compound 10e.**

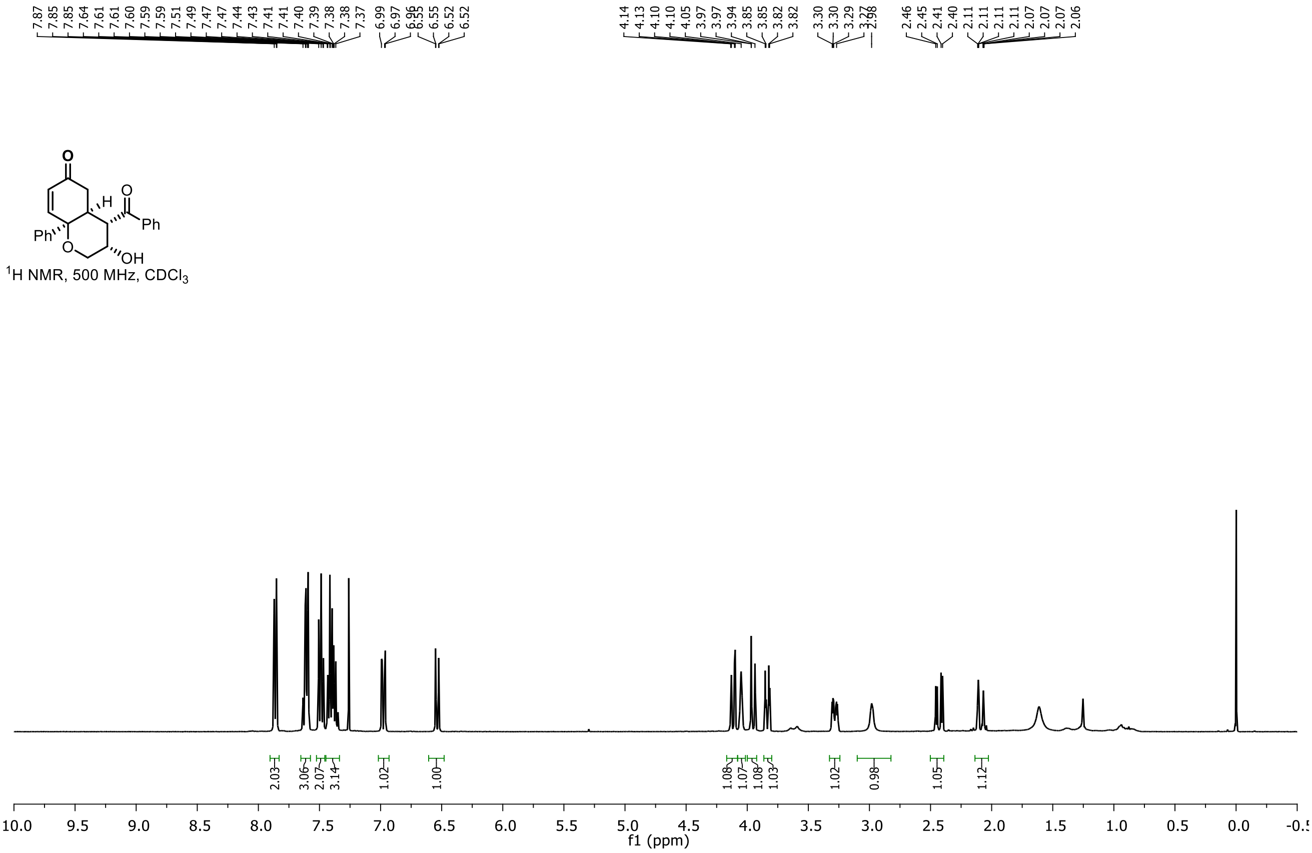

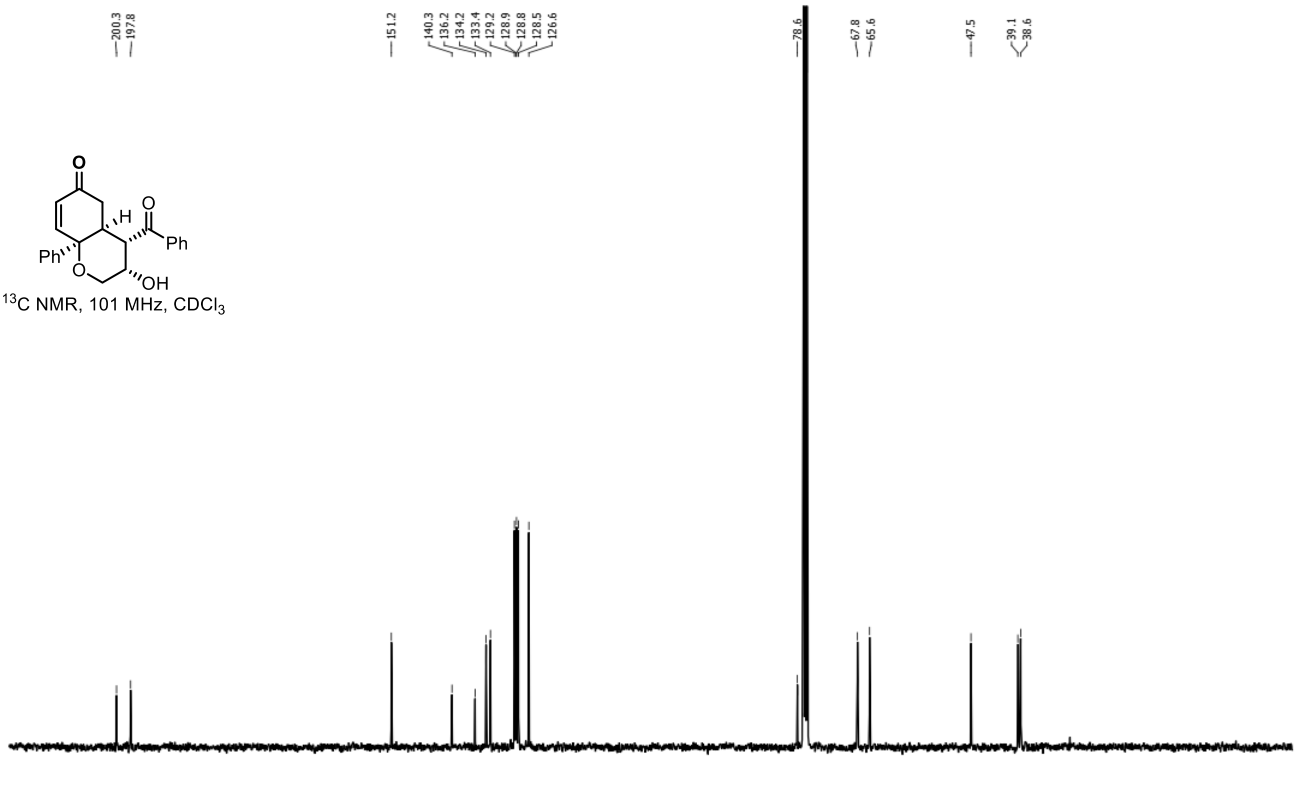

Supplementary Figure 63. ^{1}H NMR and ^{13}C NMR spectra of compound 10f.

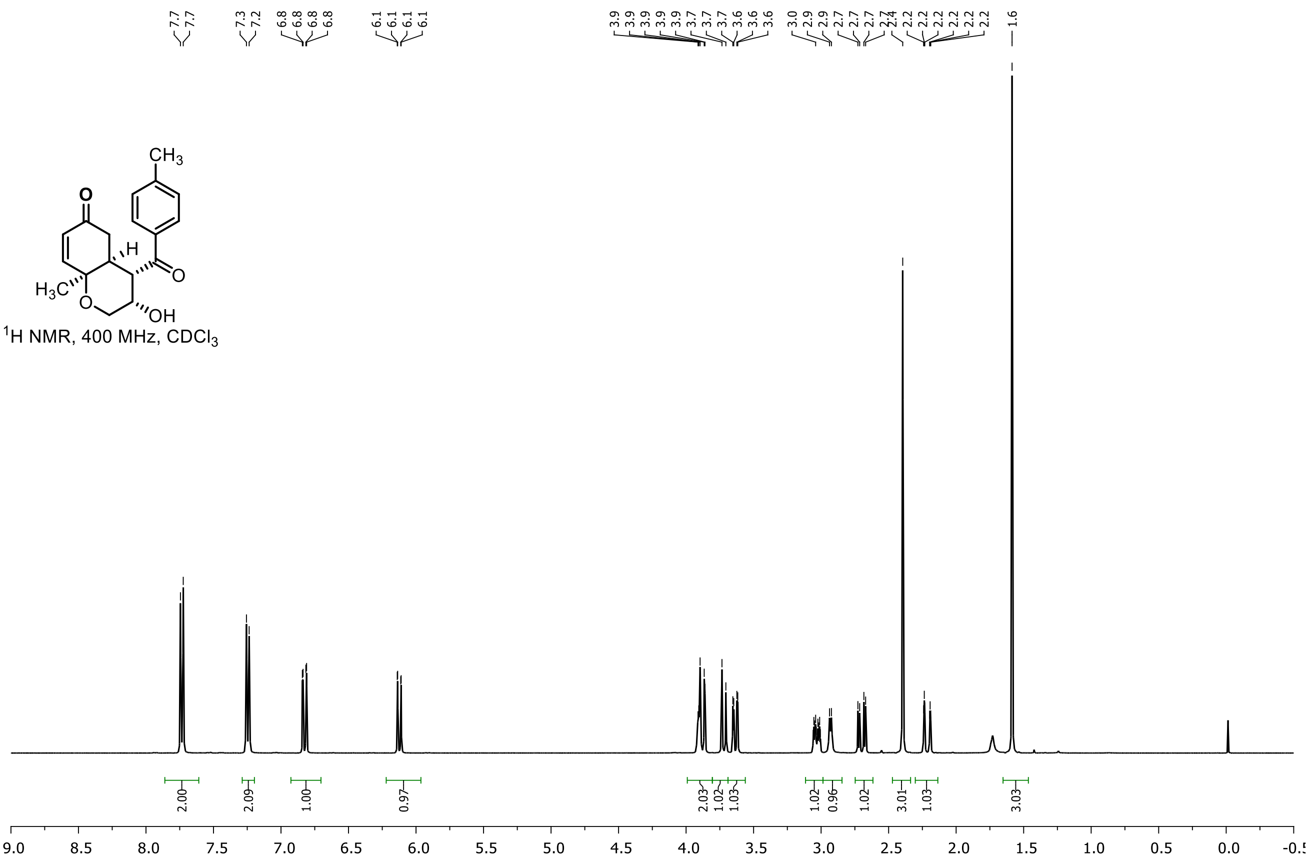

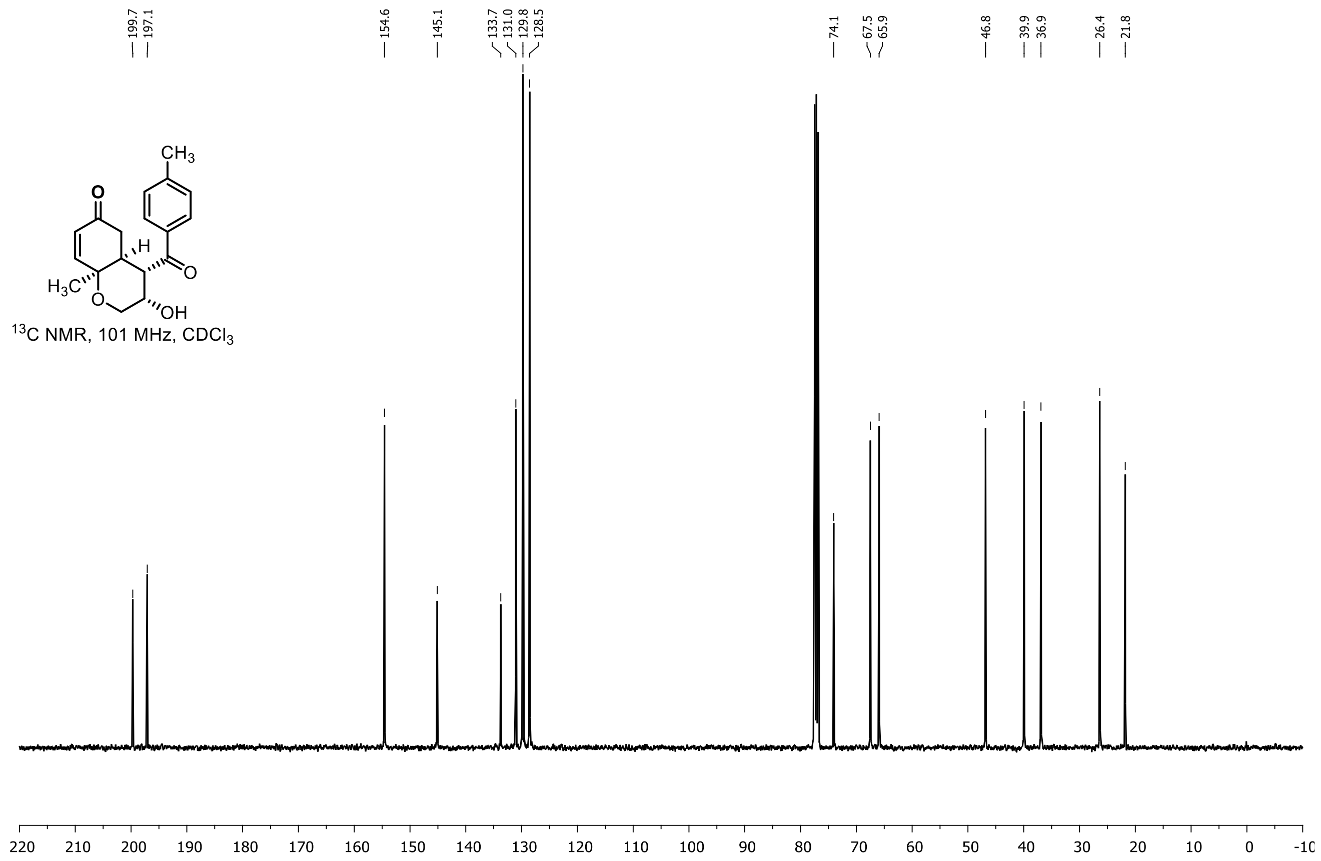

Supplementary Figure 64. **^{1}H NMR and ^{13}C NMR spectra of compound 10g.**

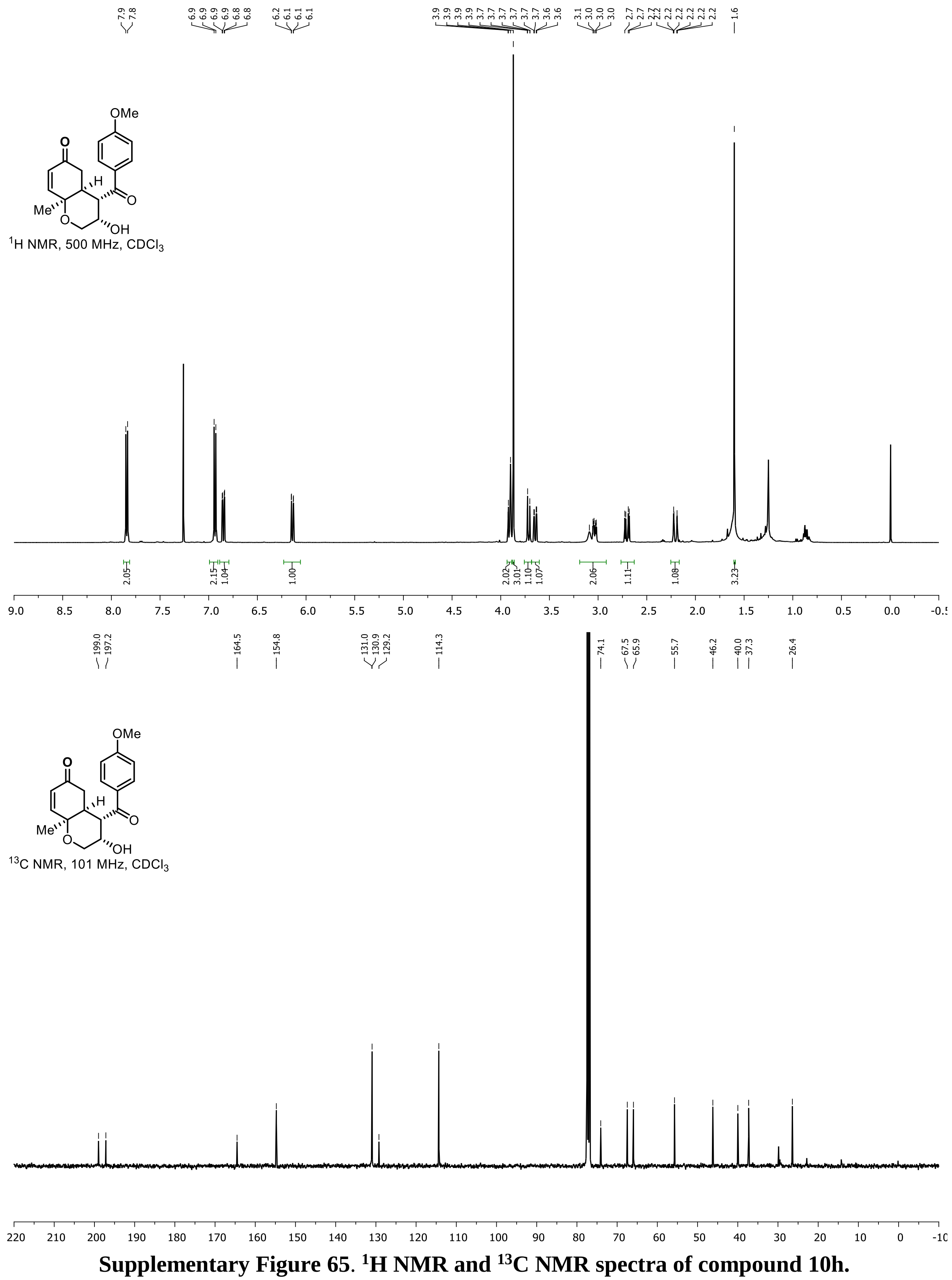

Supplementary Figure 65. **^{1}H NMR and ^{13}C NMR spectra of compound 10h.**

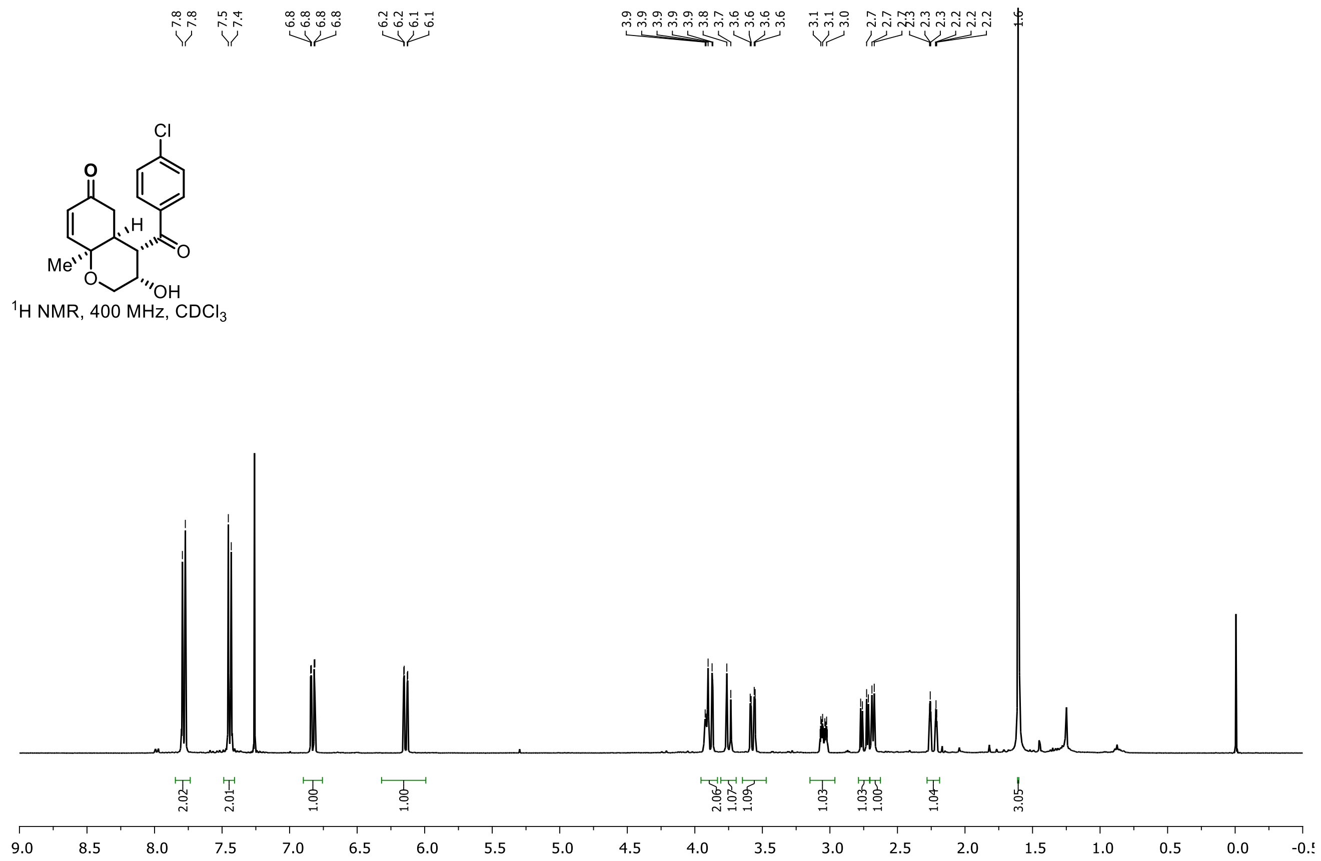

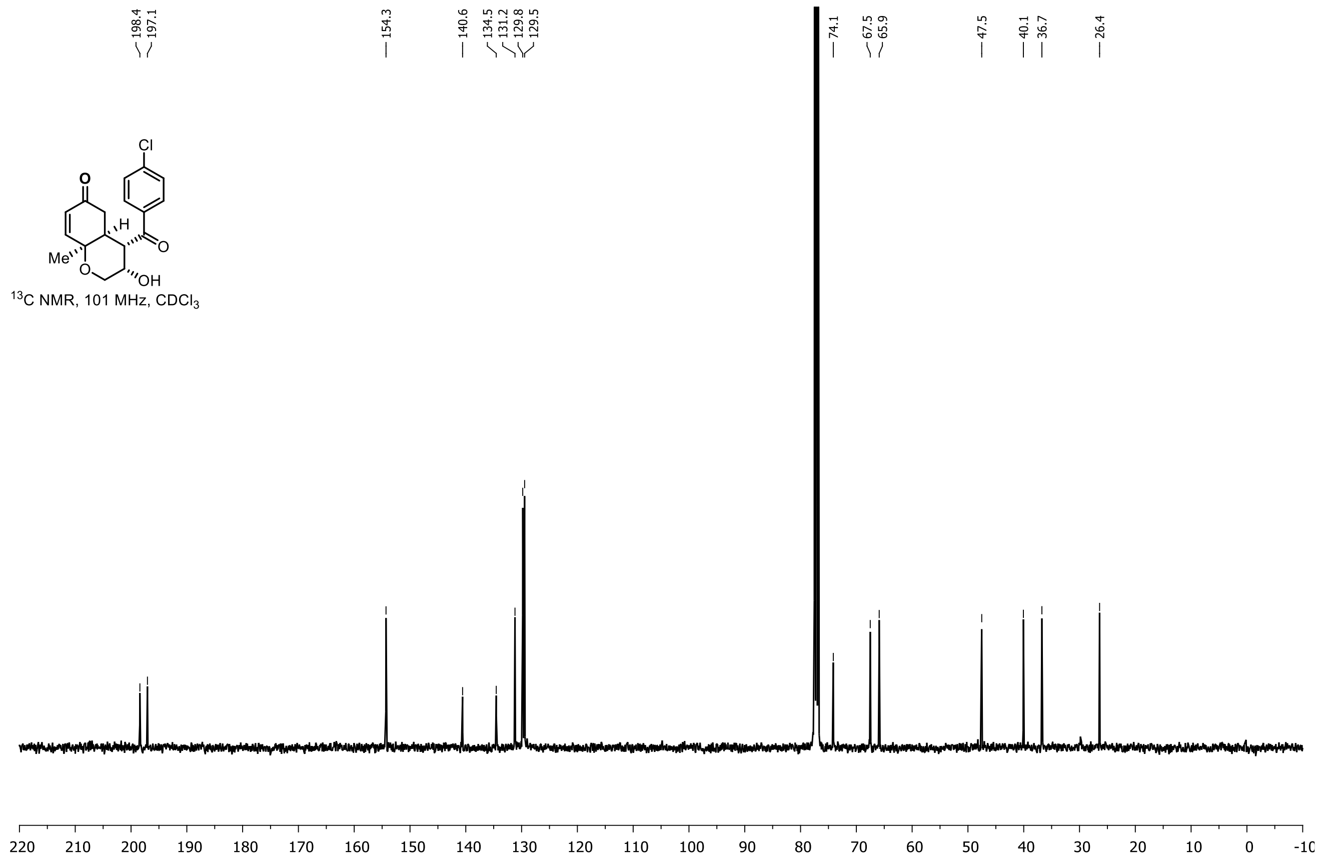

Supplementary Figure 66. ^{1}H NMR and ^{13}C NMR spectra of compound 10i.

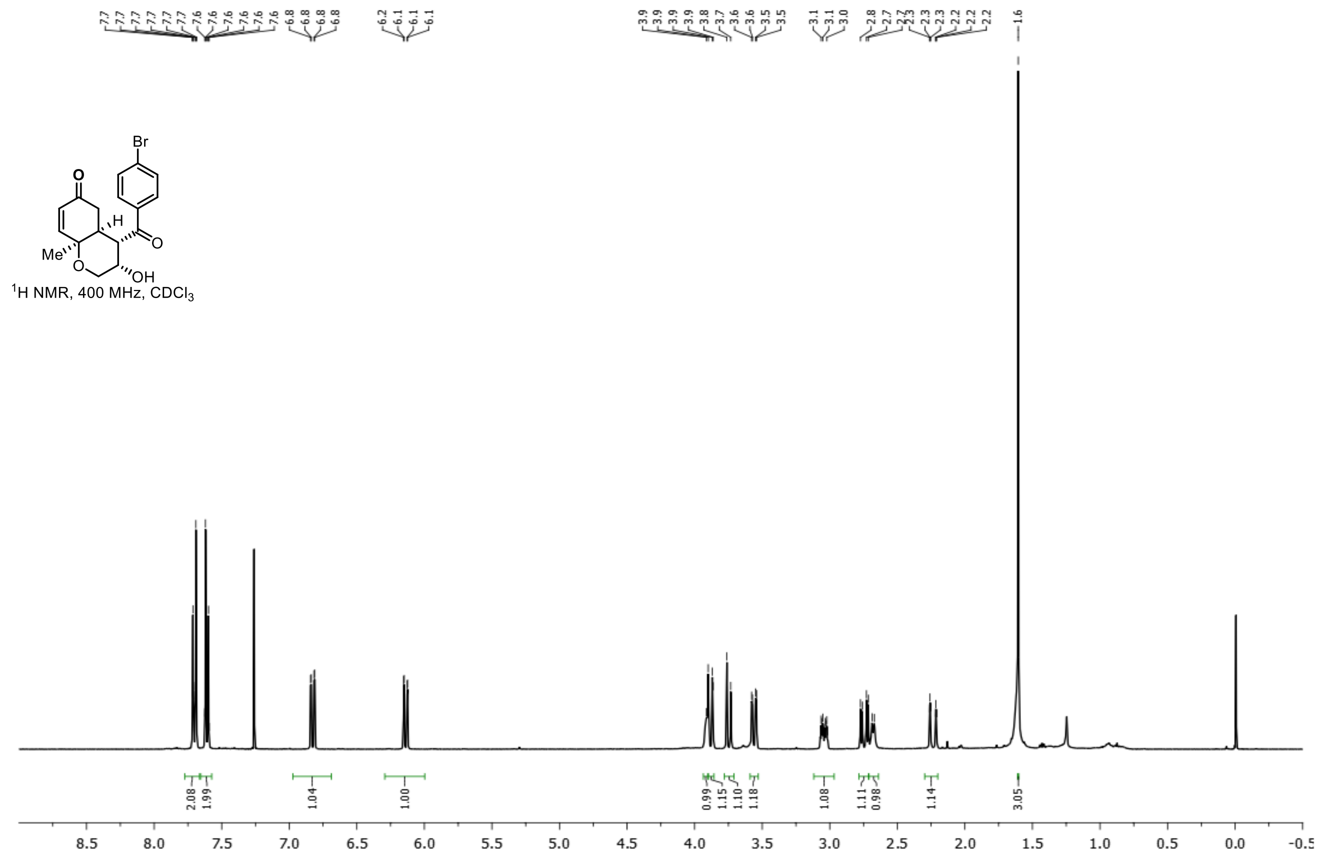

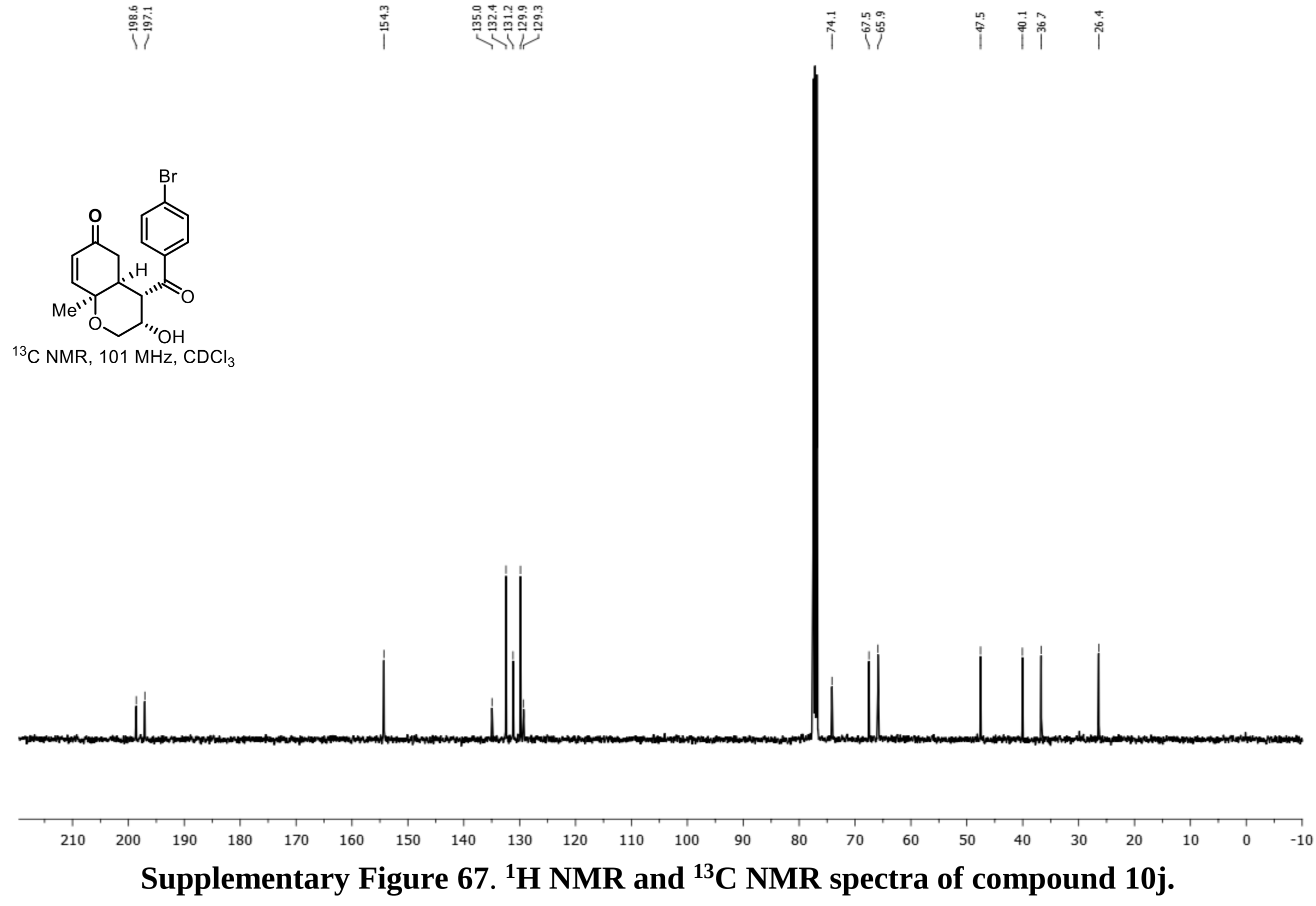

Supplementary Figure 67. **^{1}H NMR and ^{13}C NMR spectra of compound 10j.**

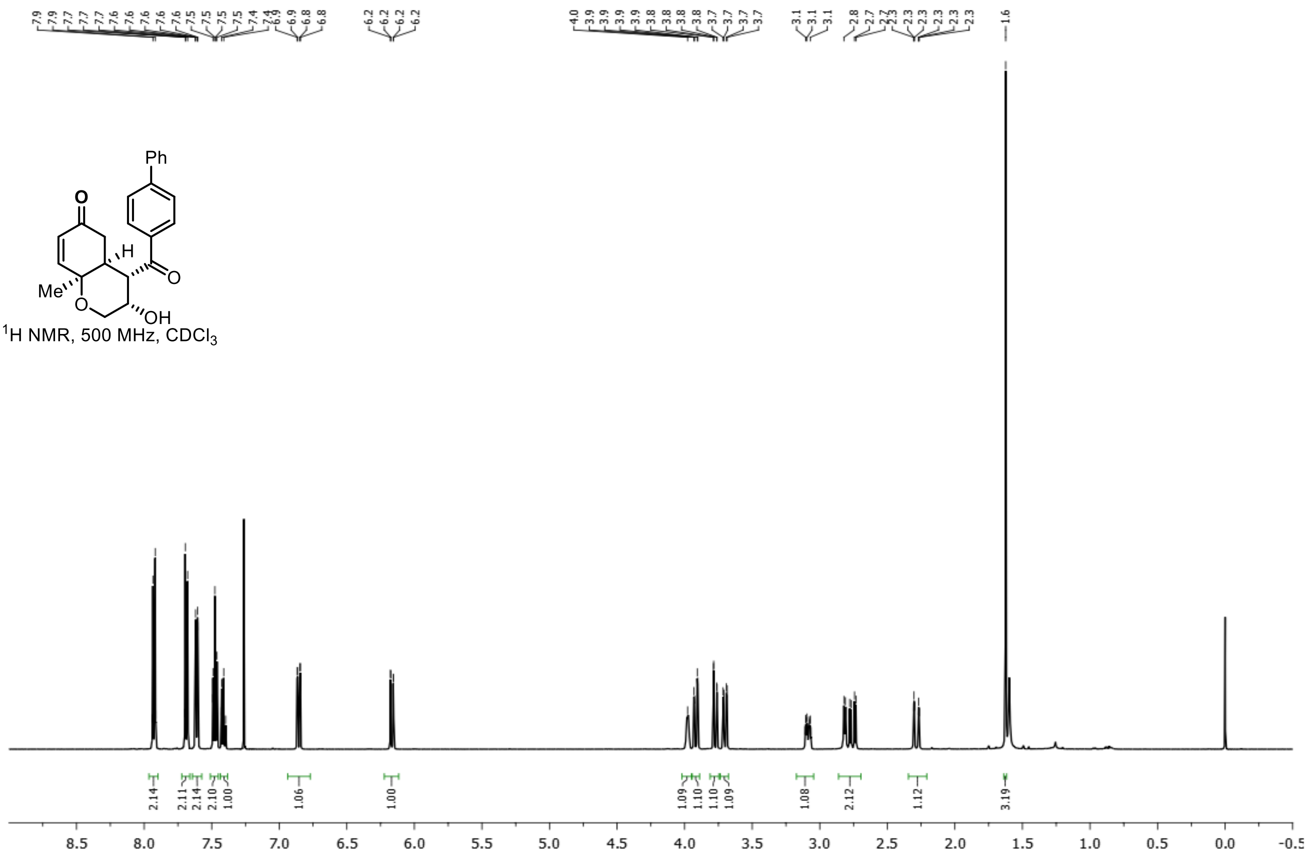

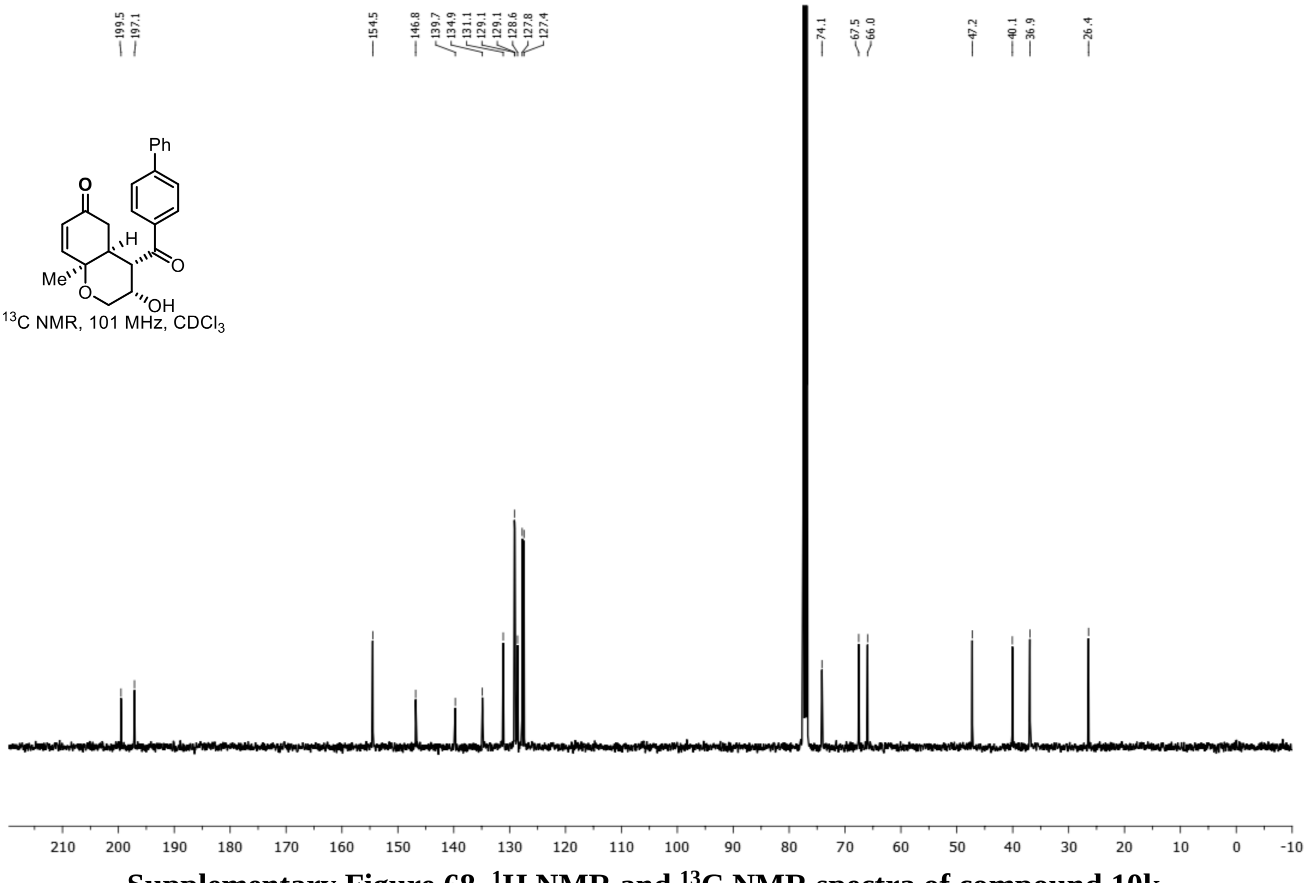

Supplementary Figure 68. **^{1}H NMR and ^{13}C NMR spectra of compound 10k.**

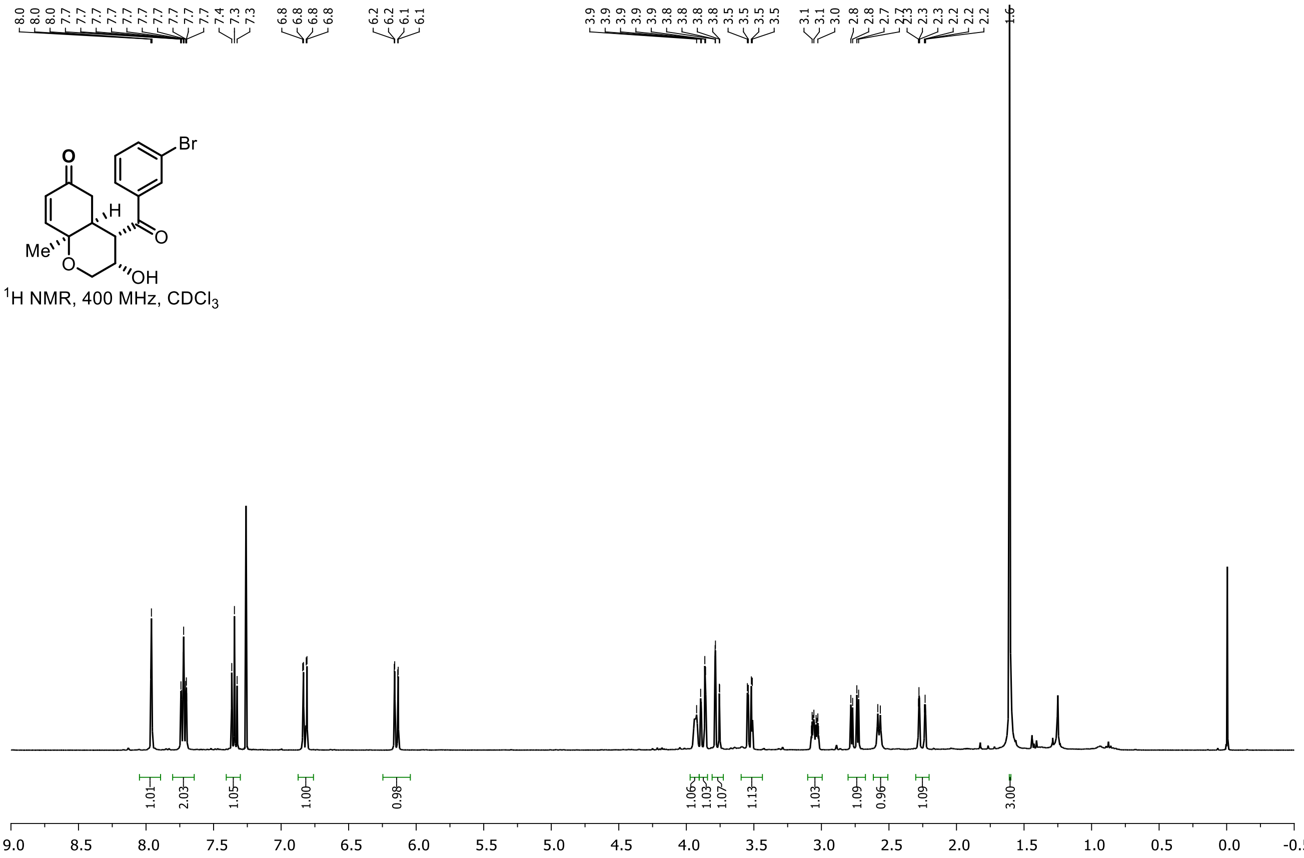

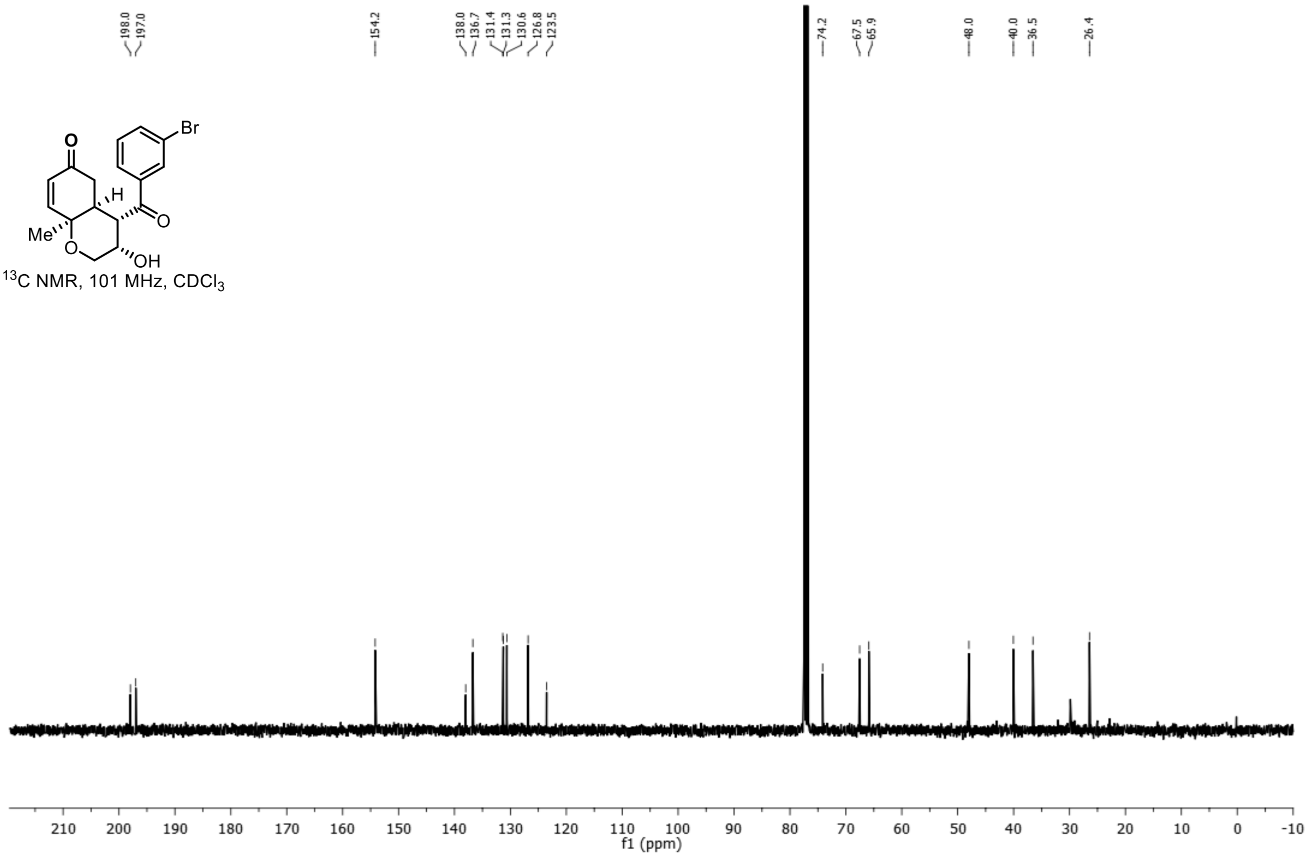

Supplementary Figure 69. **^{1}H NMR and ^{13}C NMR spectra of compound 10l.**

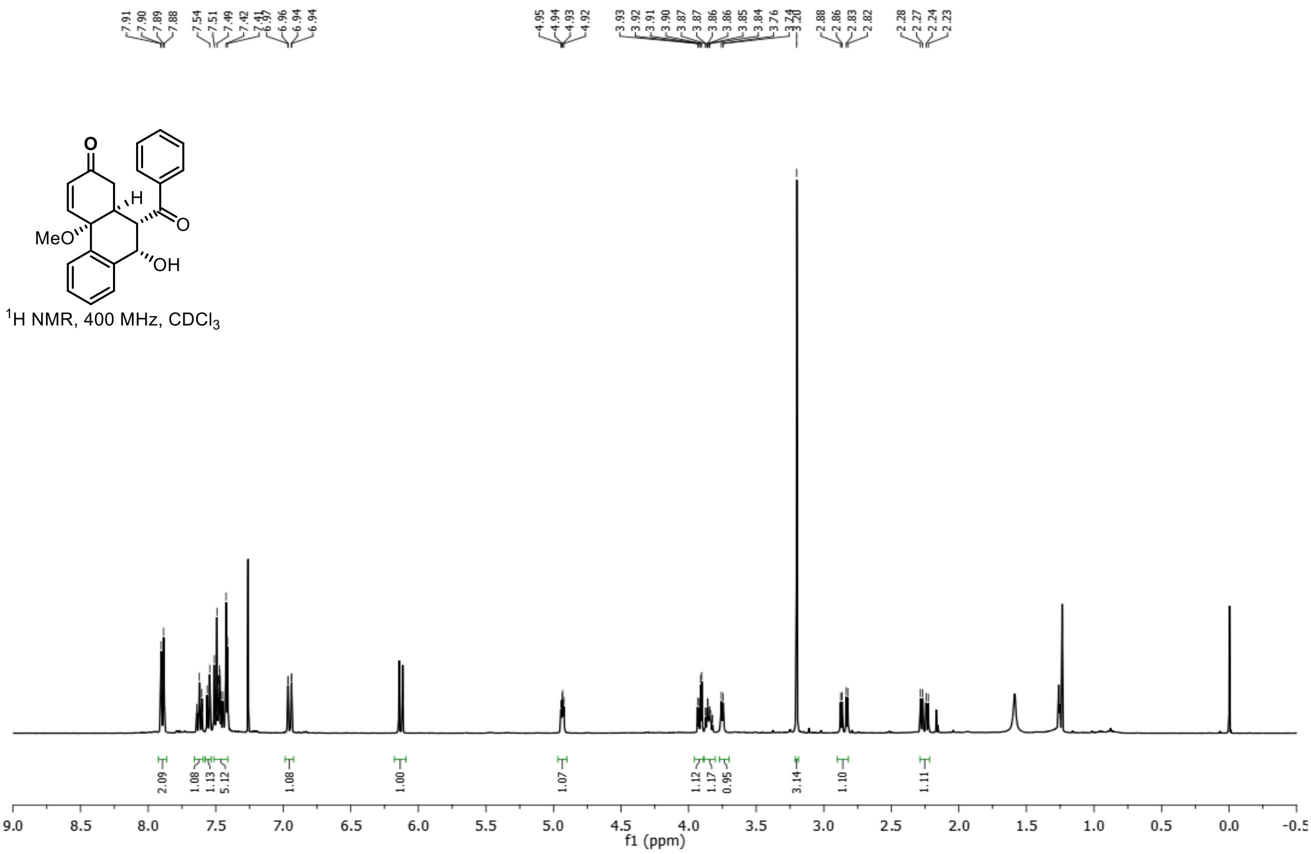

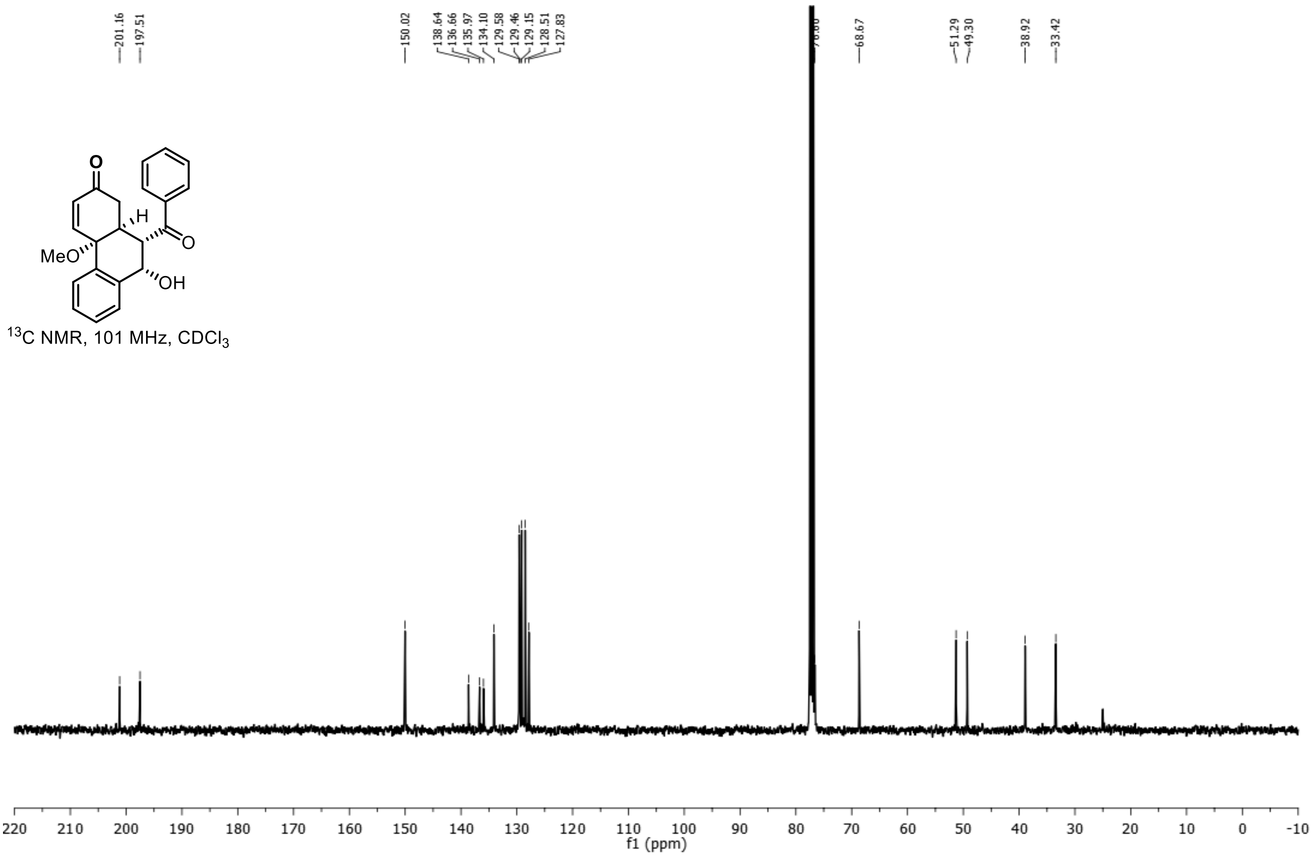

Supplementary Figure 70. ^{1}H NMR and ^{13}C NMR spectra of compound 10m.

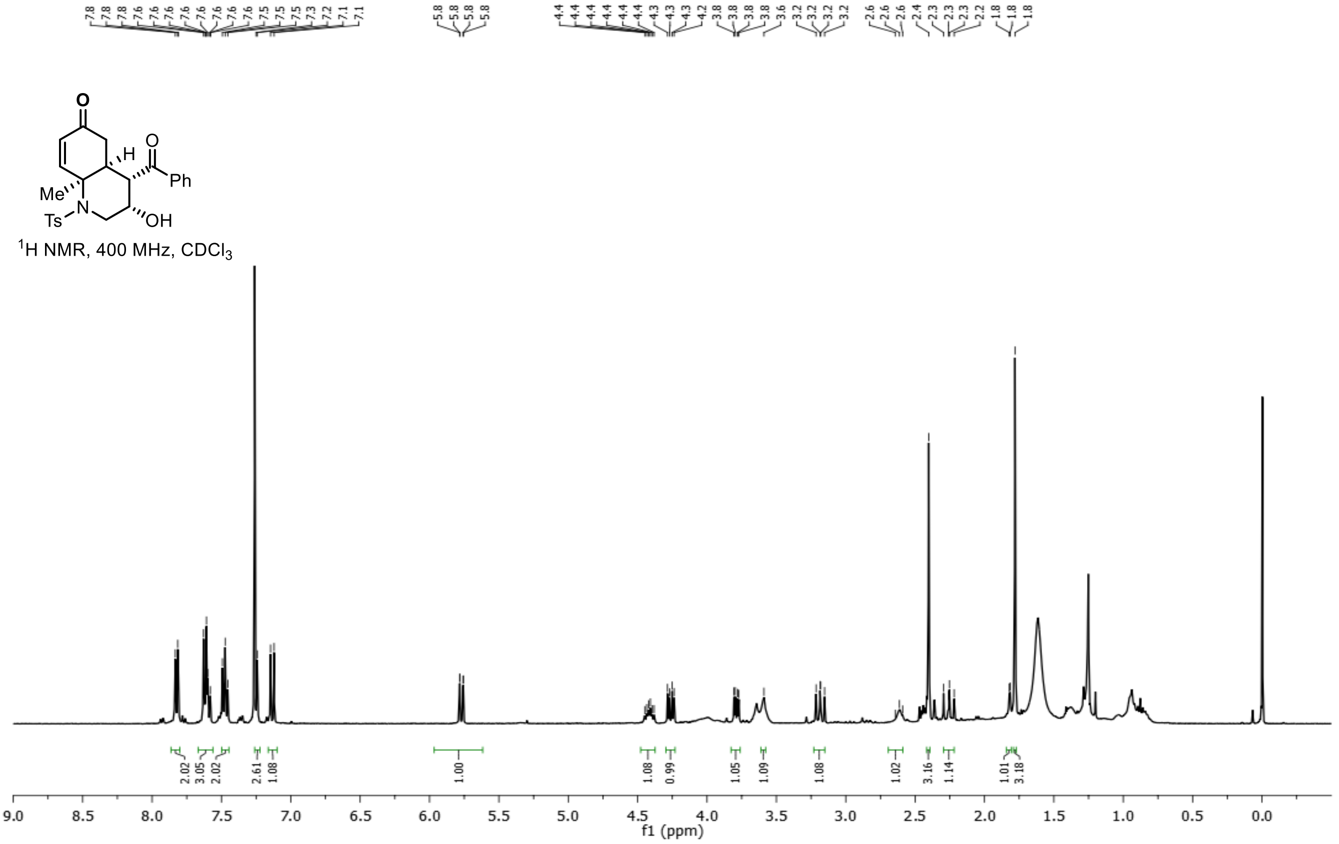

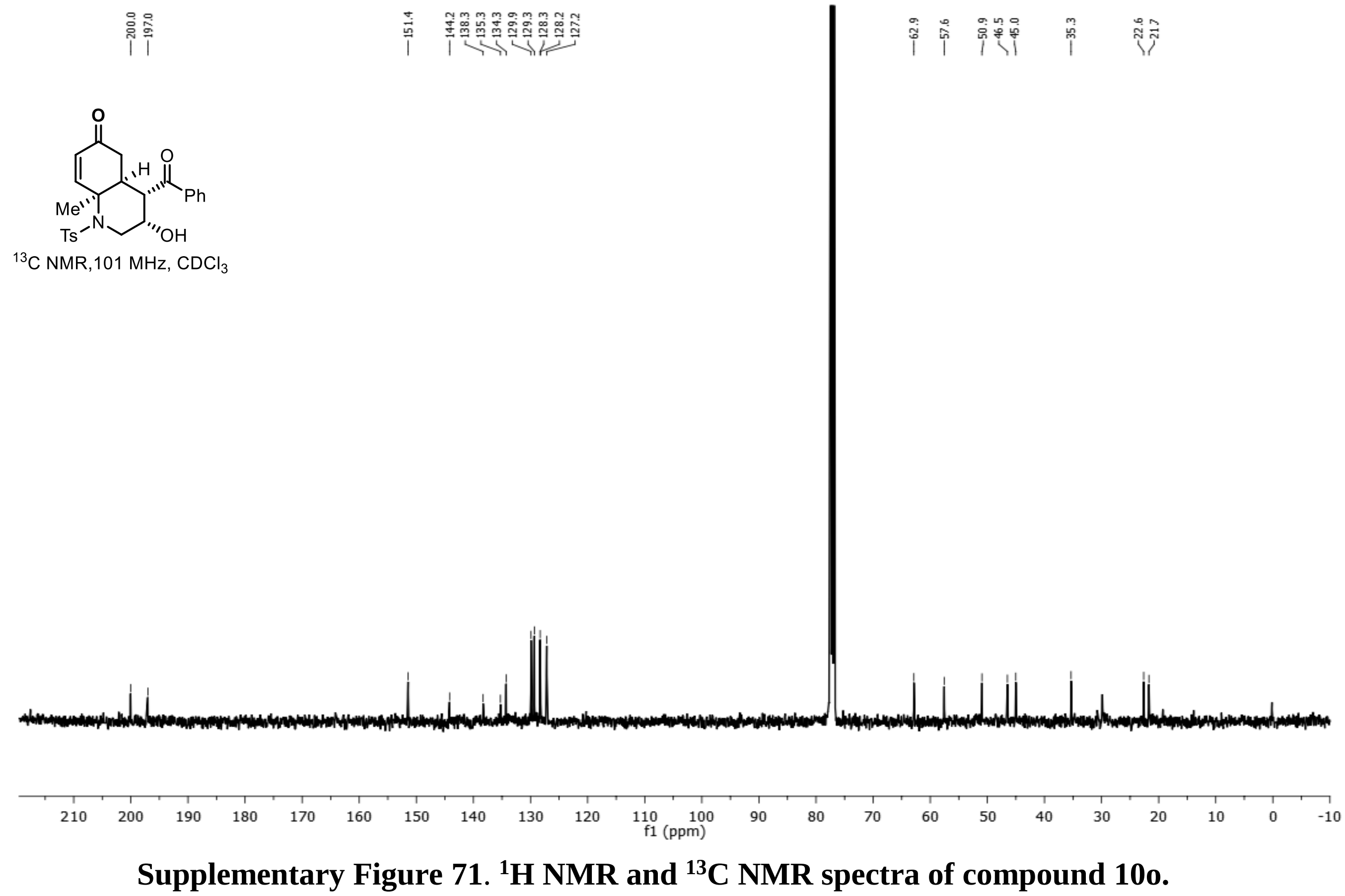

Supplementary Figure 71. **^{1}H NMR and ^{13}C NMR spectra of compound 10o.**

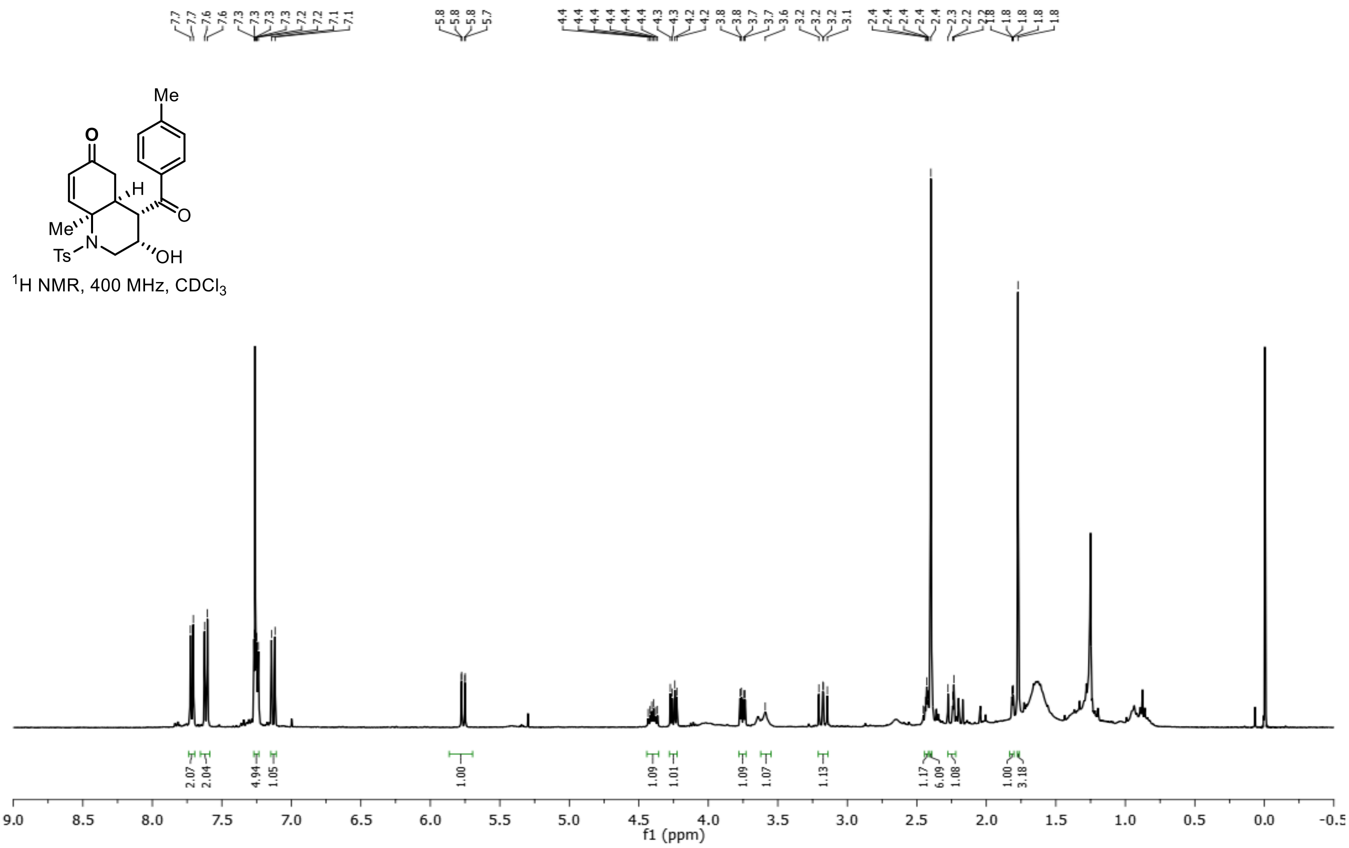

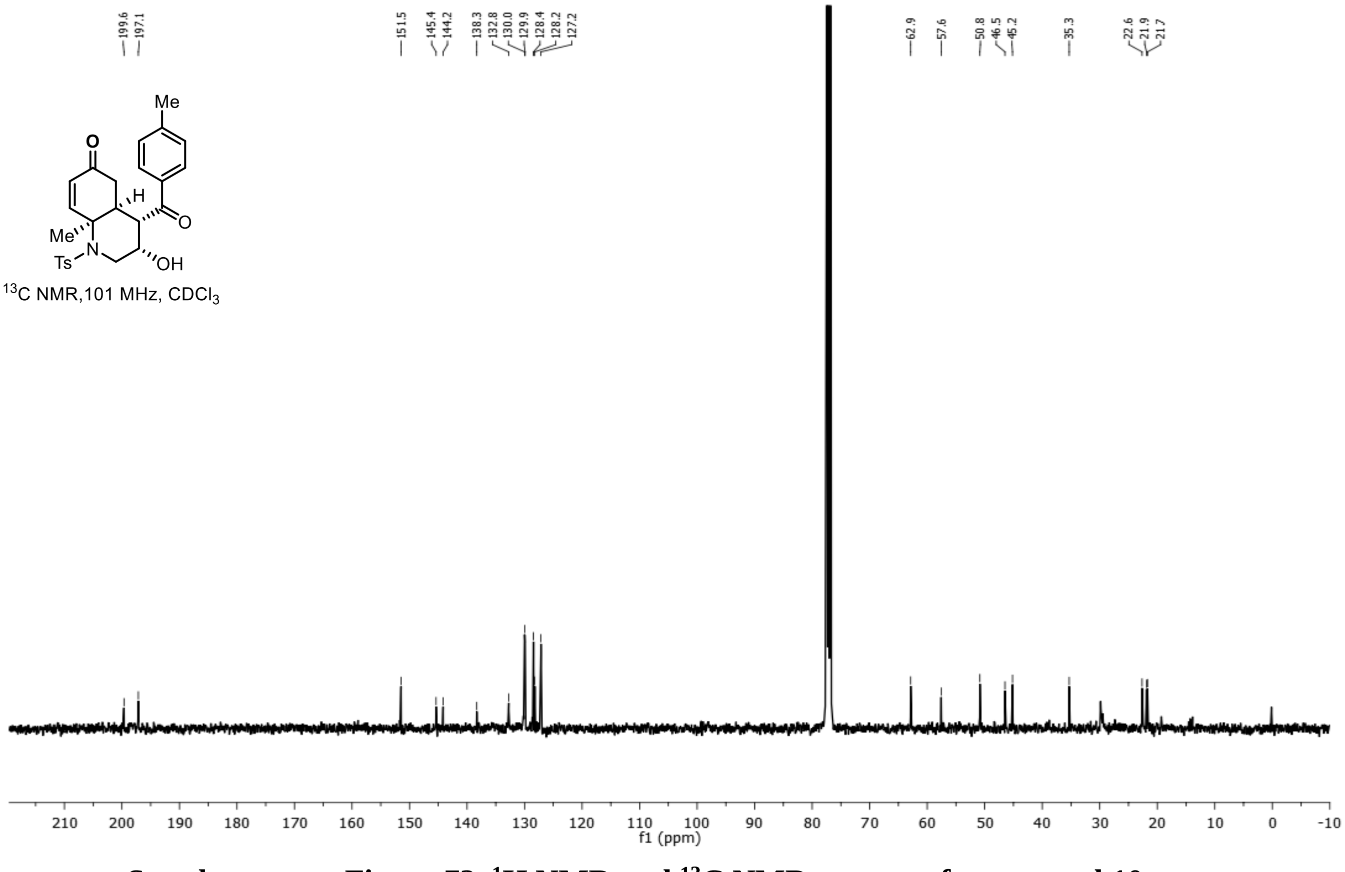

Supplementary Figure 72. **^{1}H NMR and ^{13}C NMR spectra of compound 10p.**

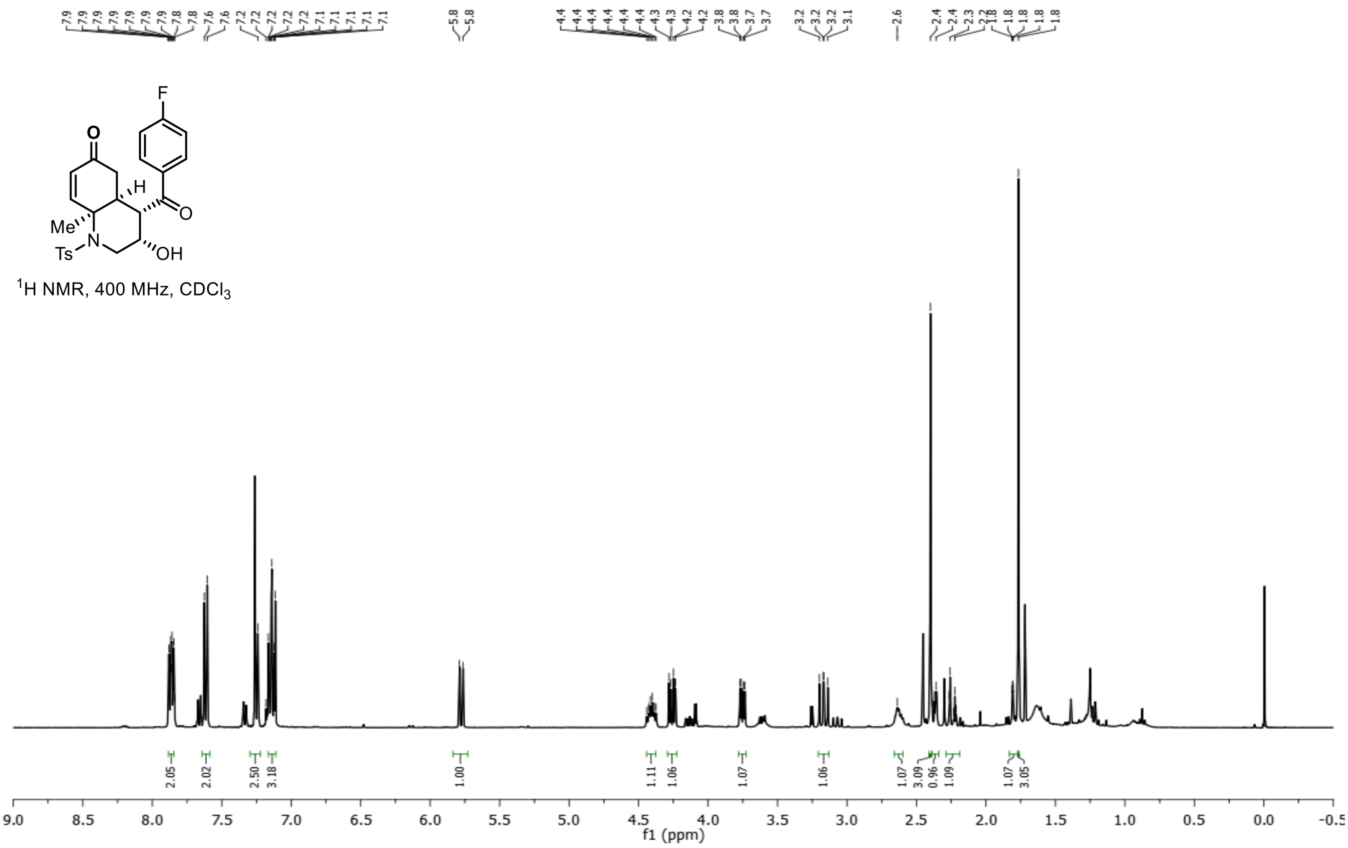

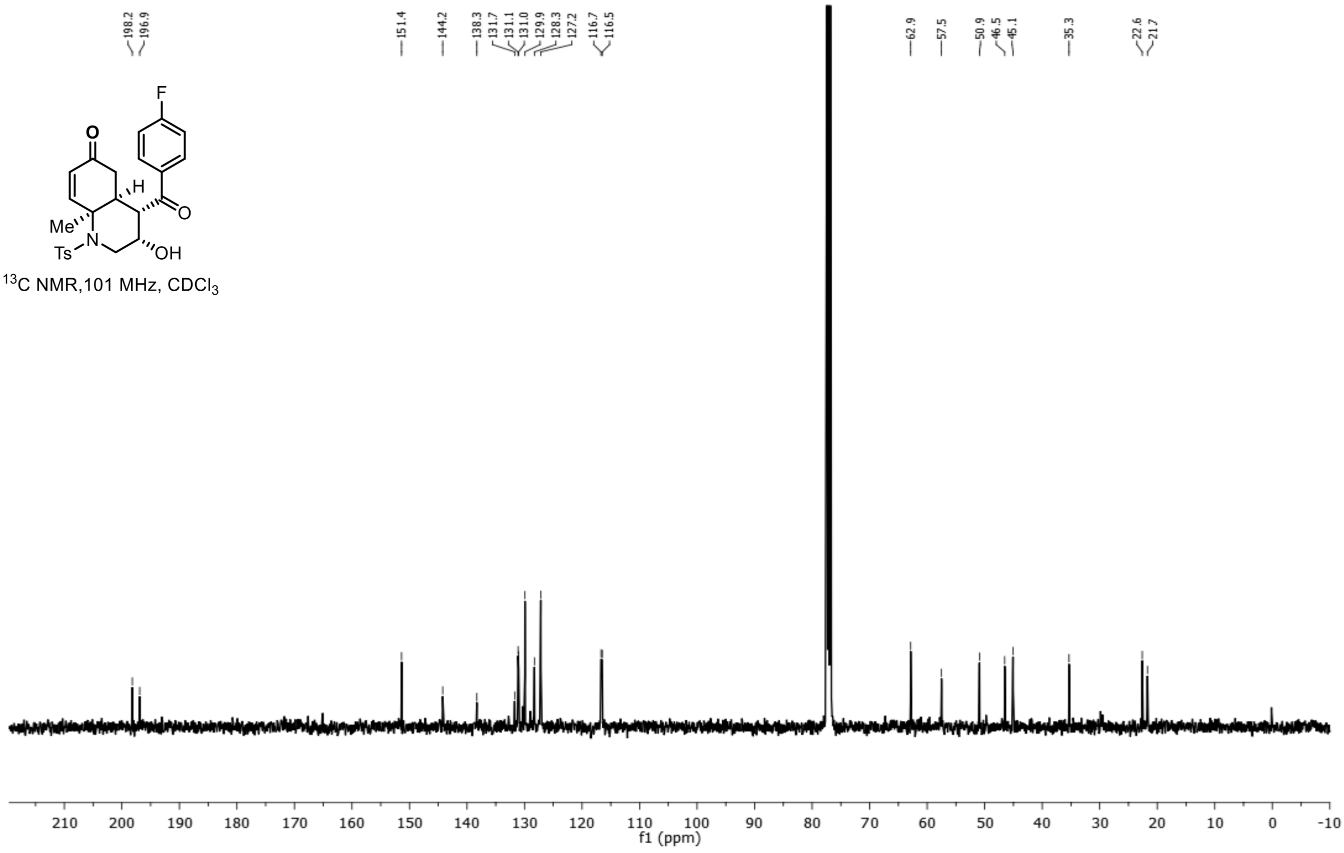

Supplementary Figure 73. **^{1}H NMR and ^{13}C NMR spectra of compound 10q.**

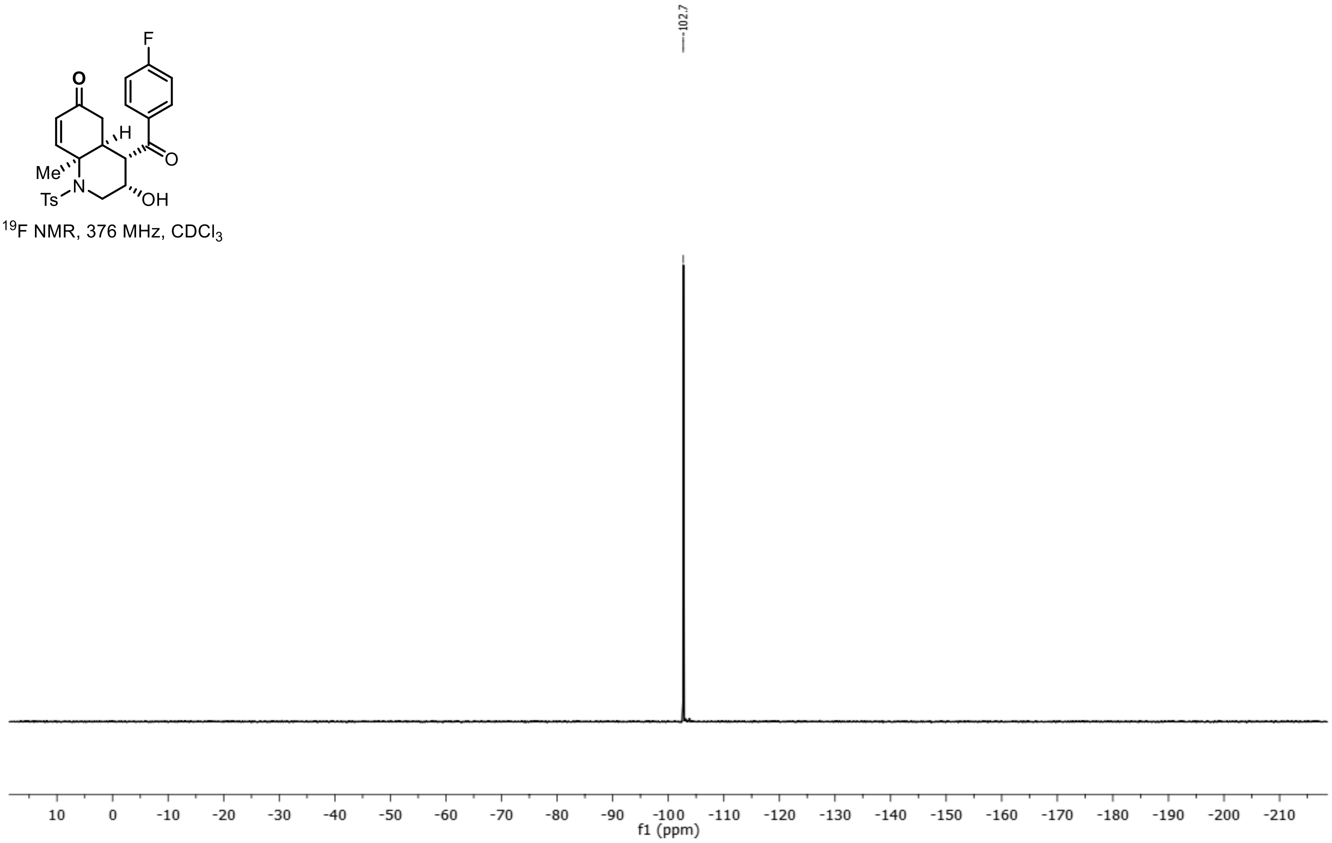

Supplementary Figure 74. **^{19}F NMR spectra of compound 10q.**

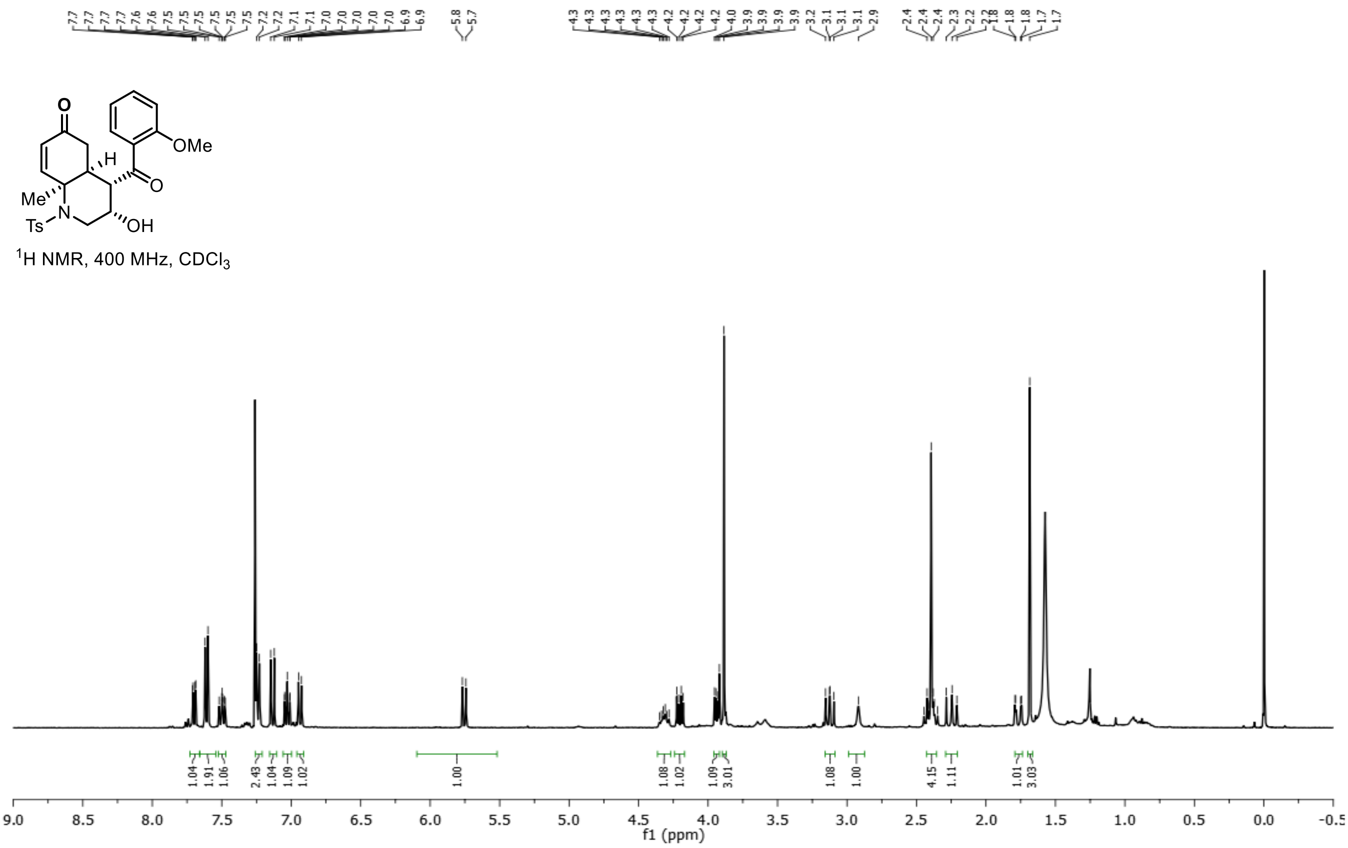

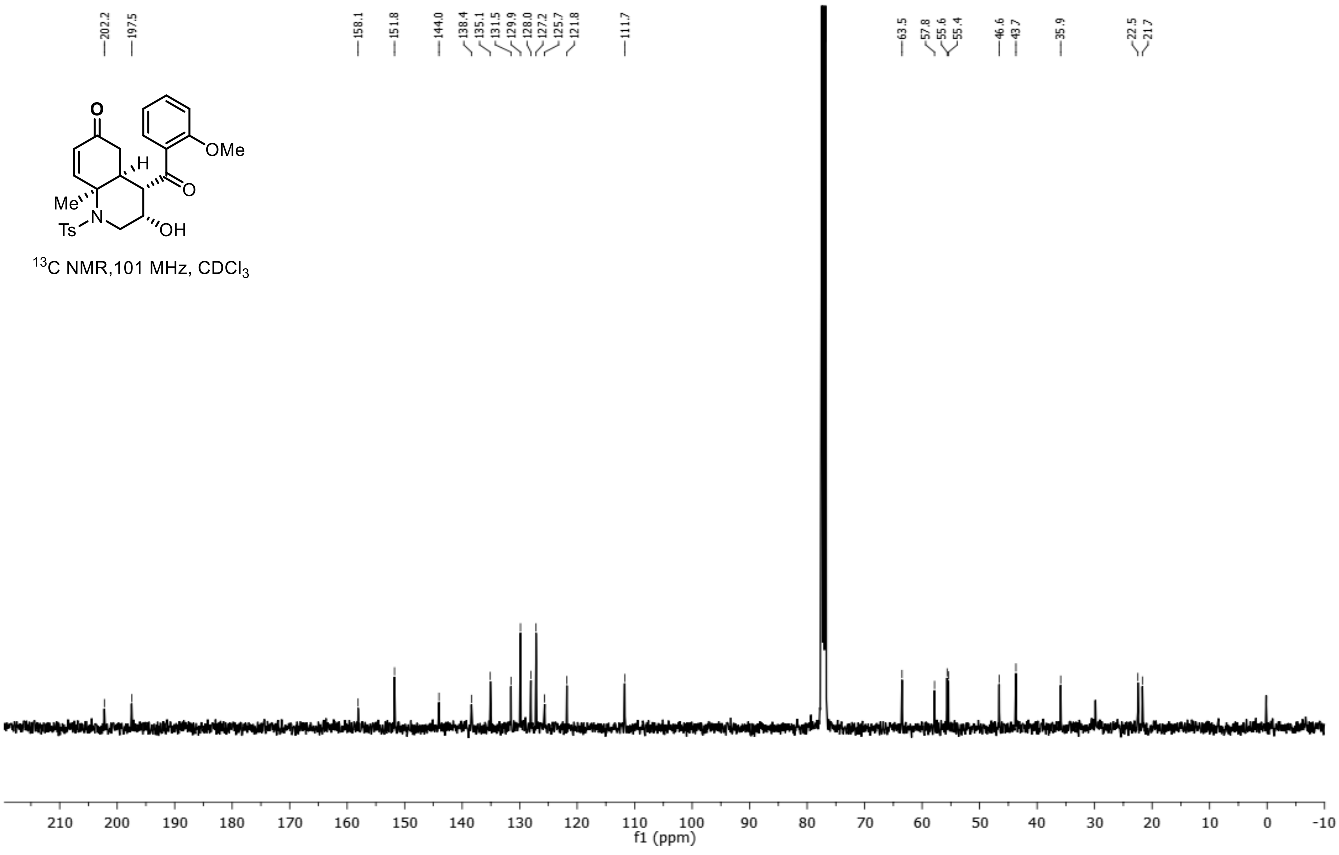

Supplementary Figure 75. ^{1}H NMR and ^{13}C NMR spectra of compound 10r.

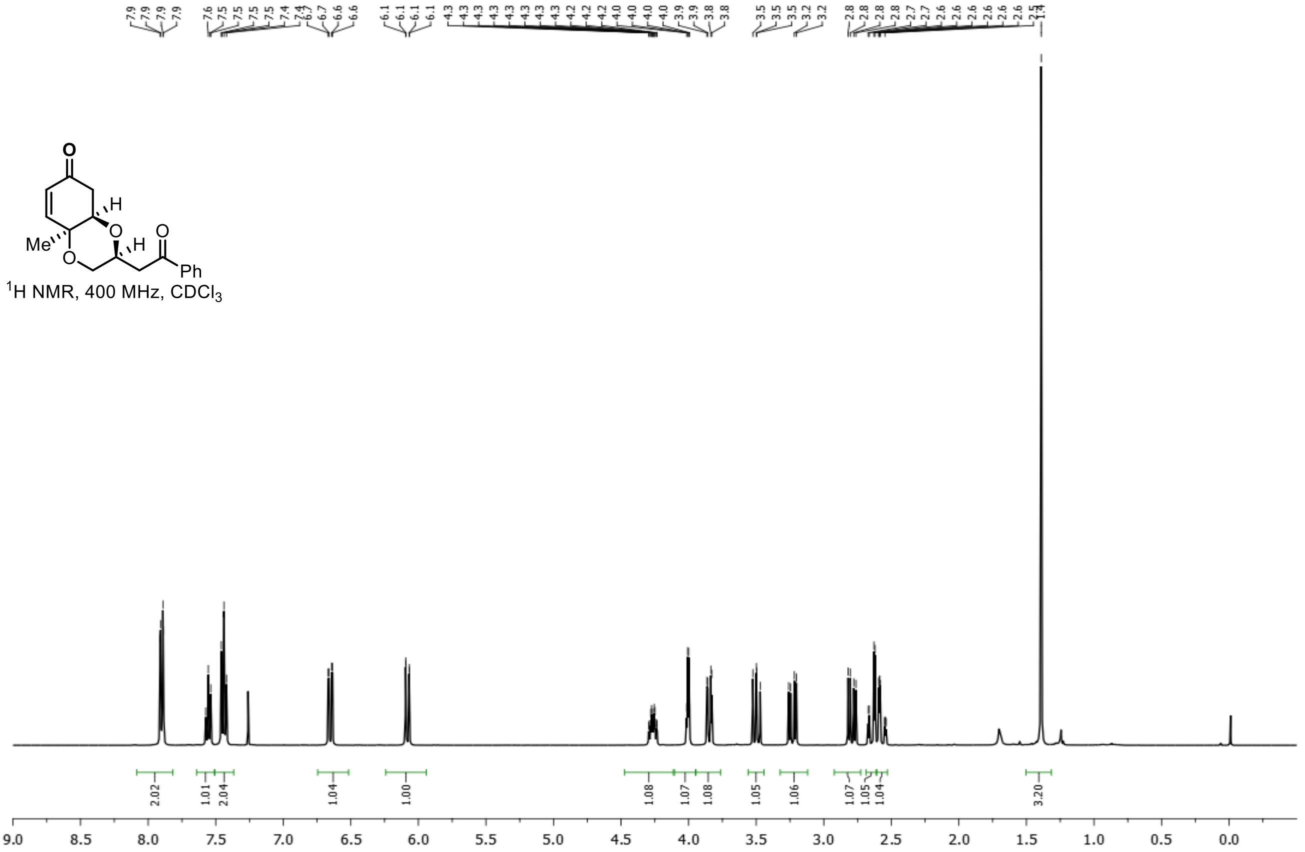

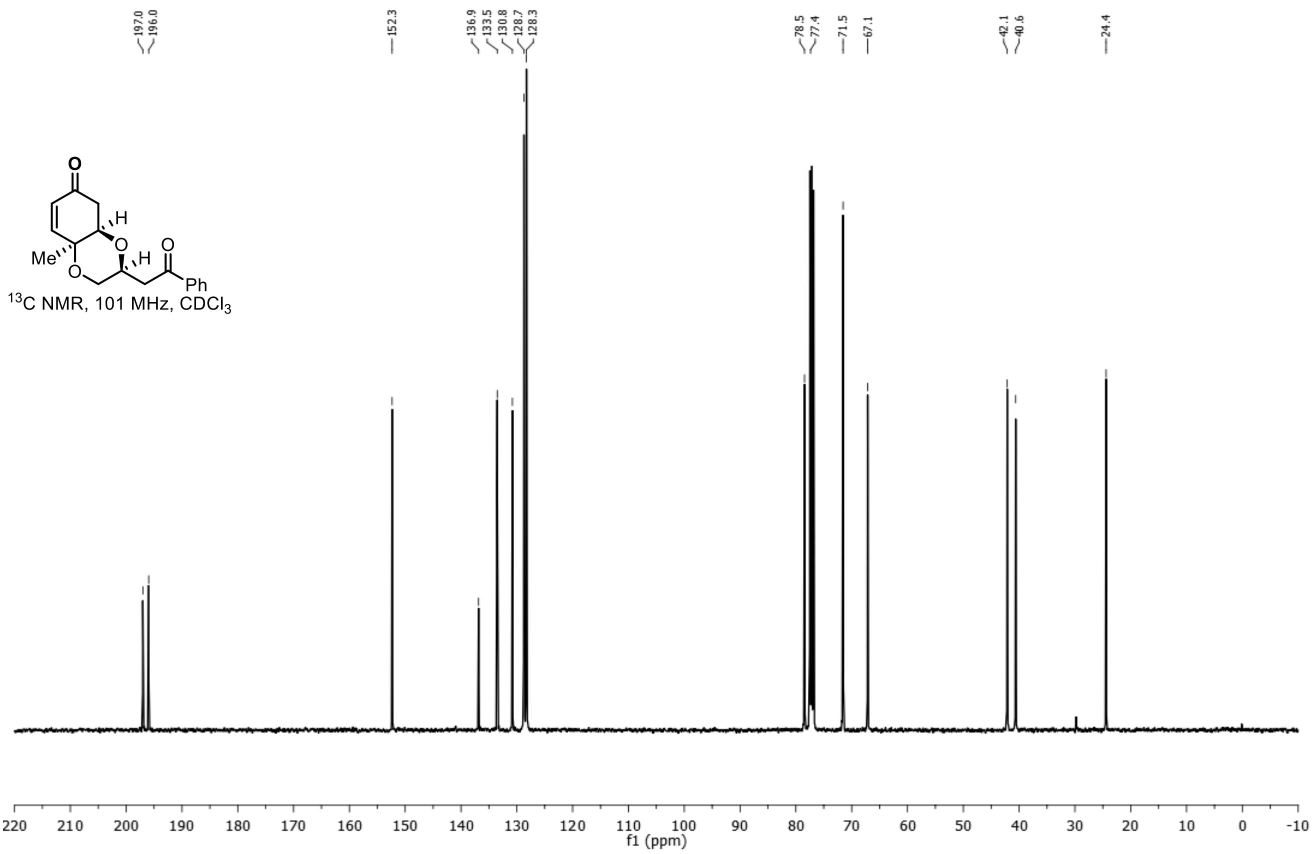

Supplementary Figure 76. **^{1}H NMR and ^{13}C NMR spectra of compound 11a.**

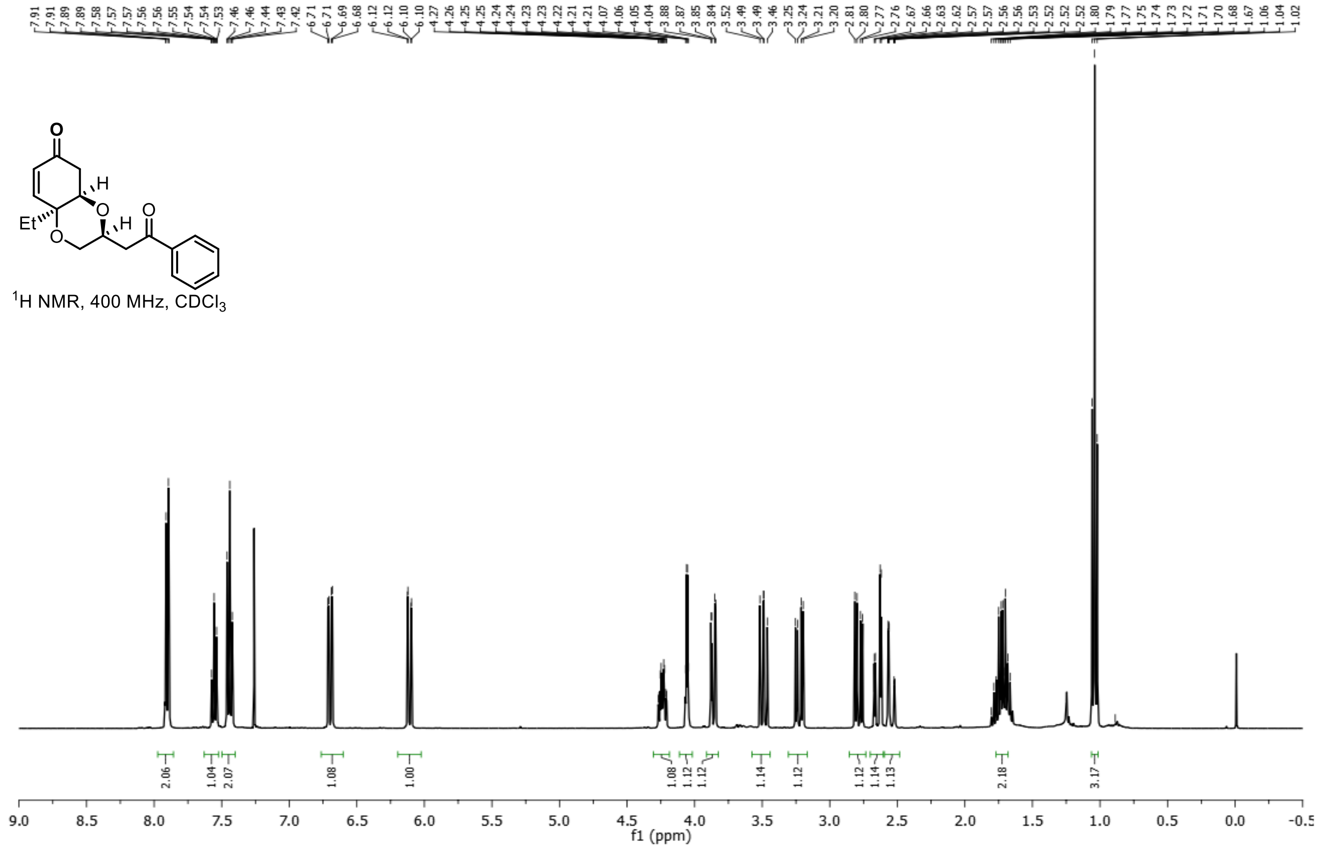

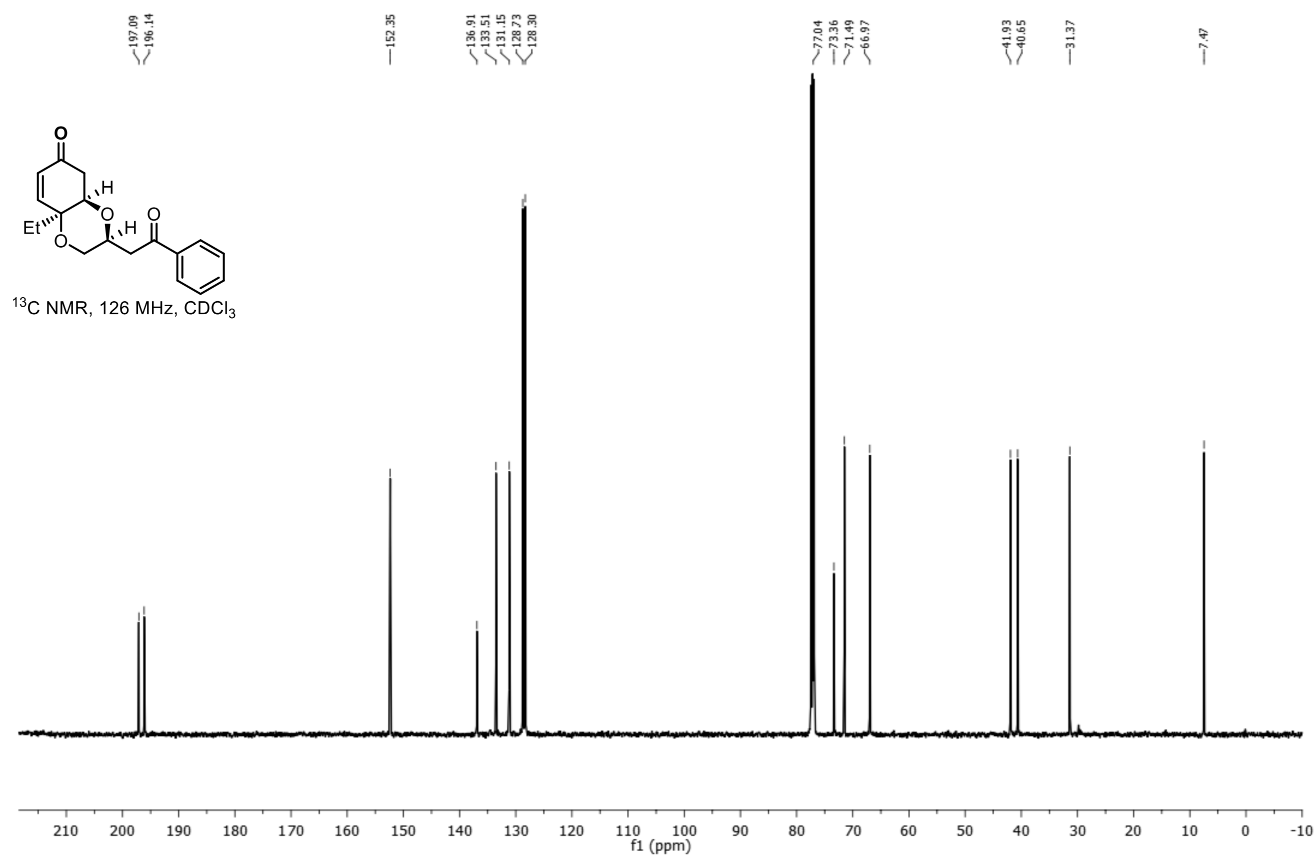

Supplementary Figure 77. **^{1}H NMR and ^{13}C NMR spectra of compound 11b.**

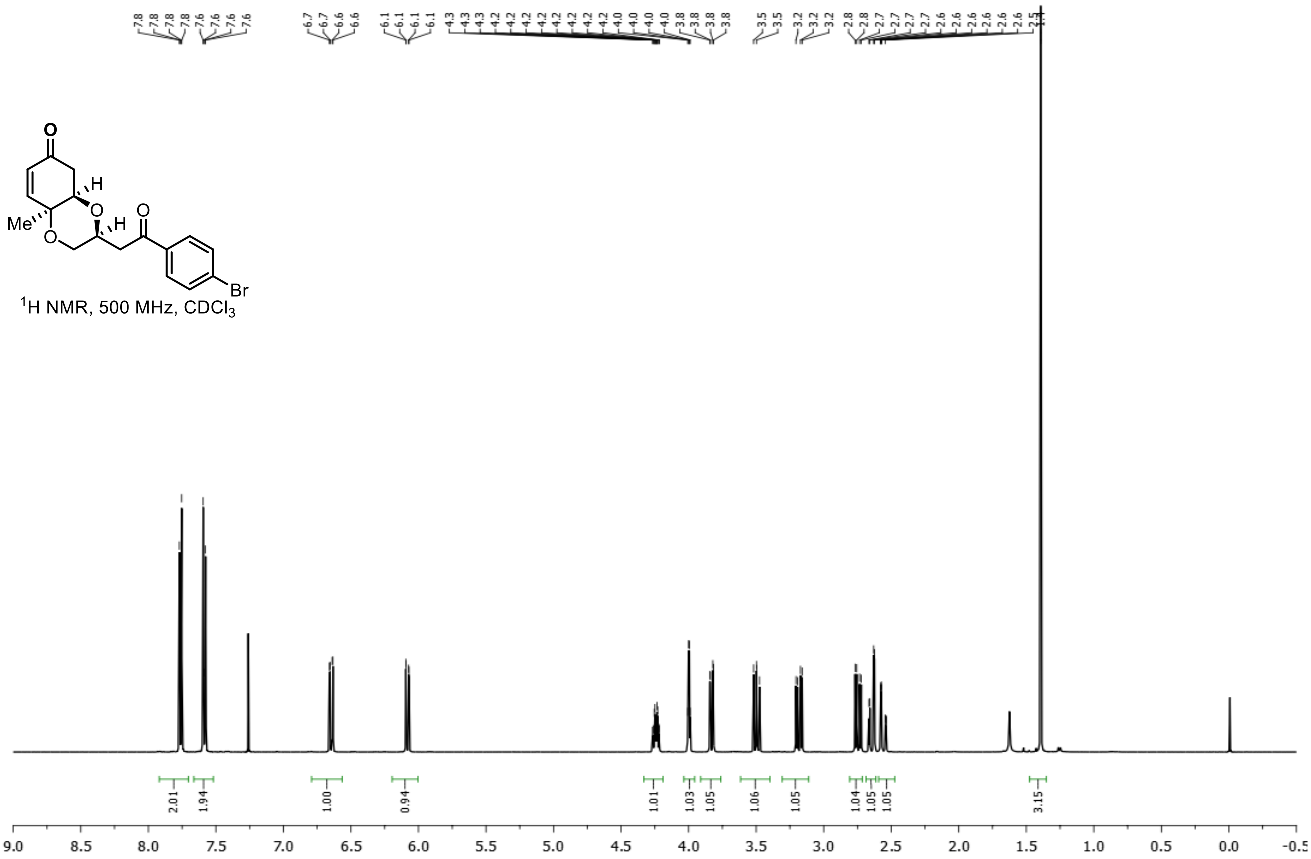

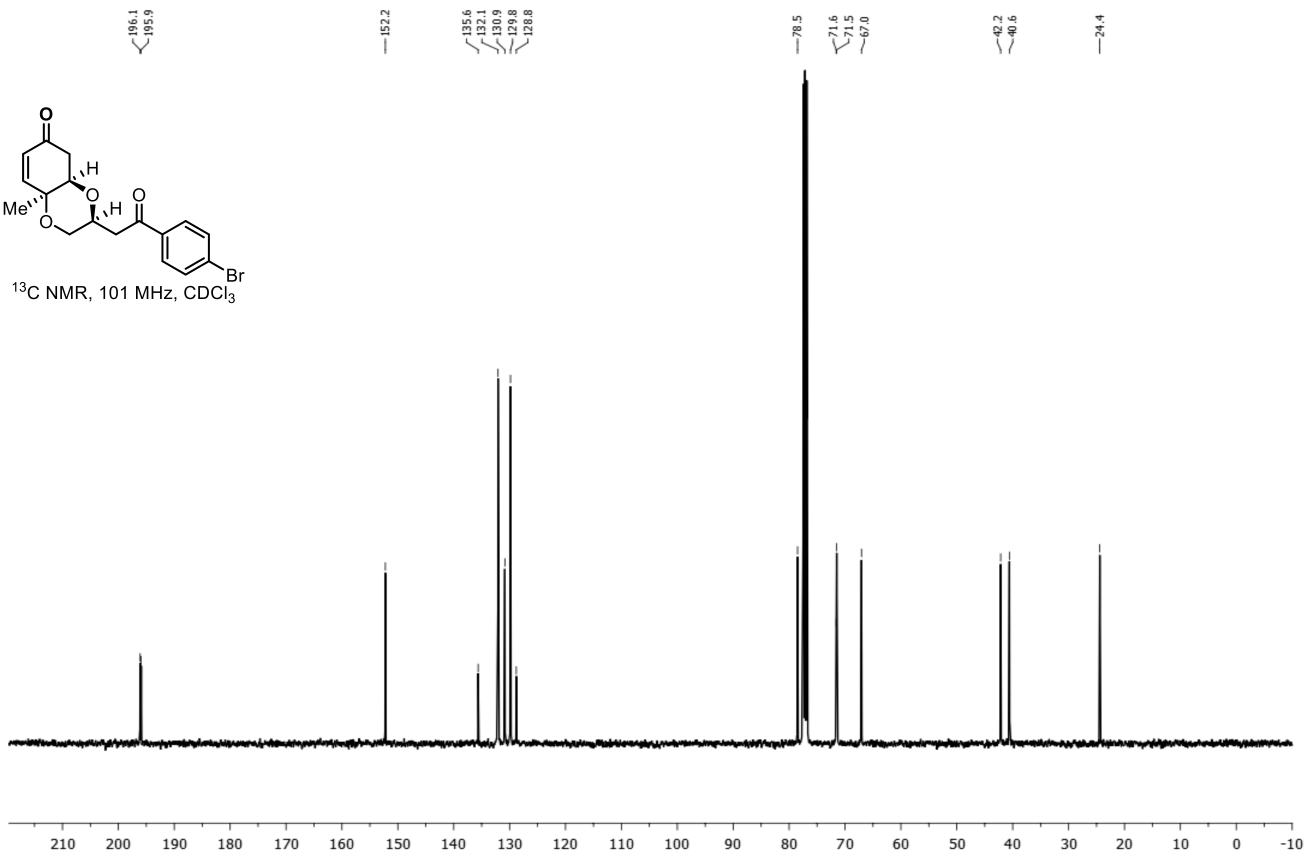

Supplementary Figure 78. ^{1}H NMR and ^{13}C NMR spectra of compound 11c.

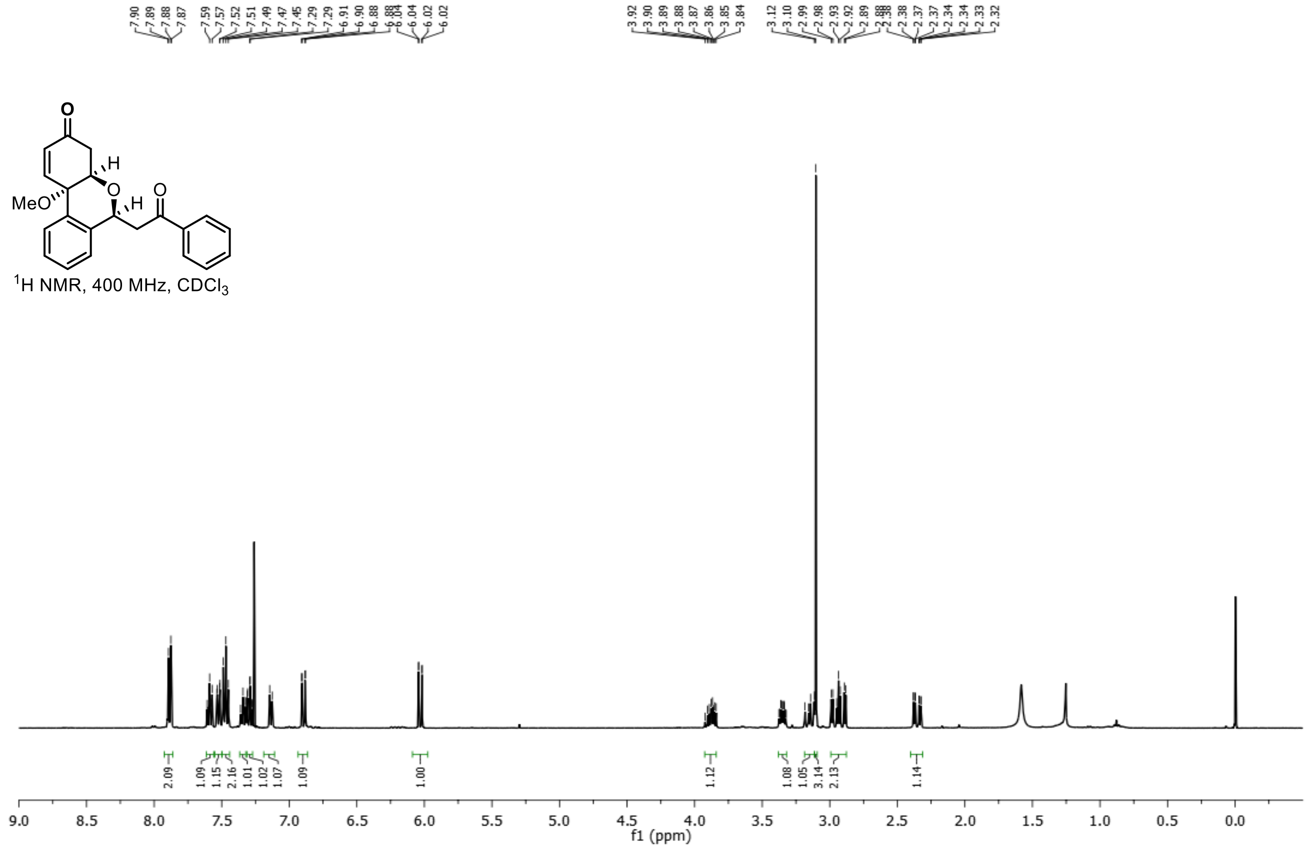

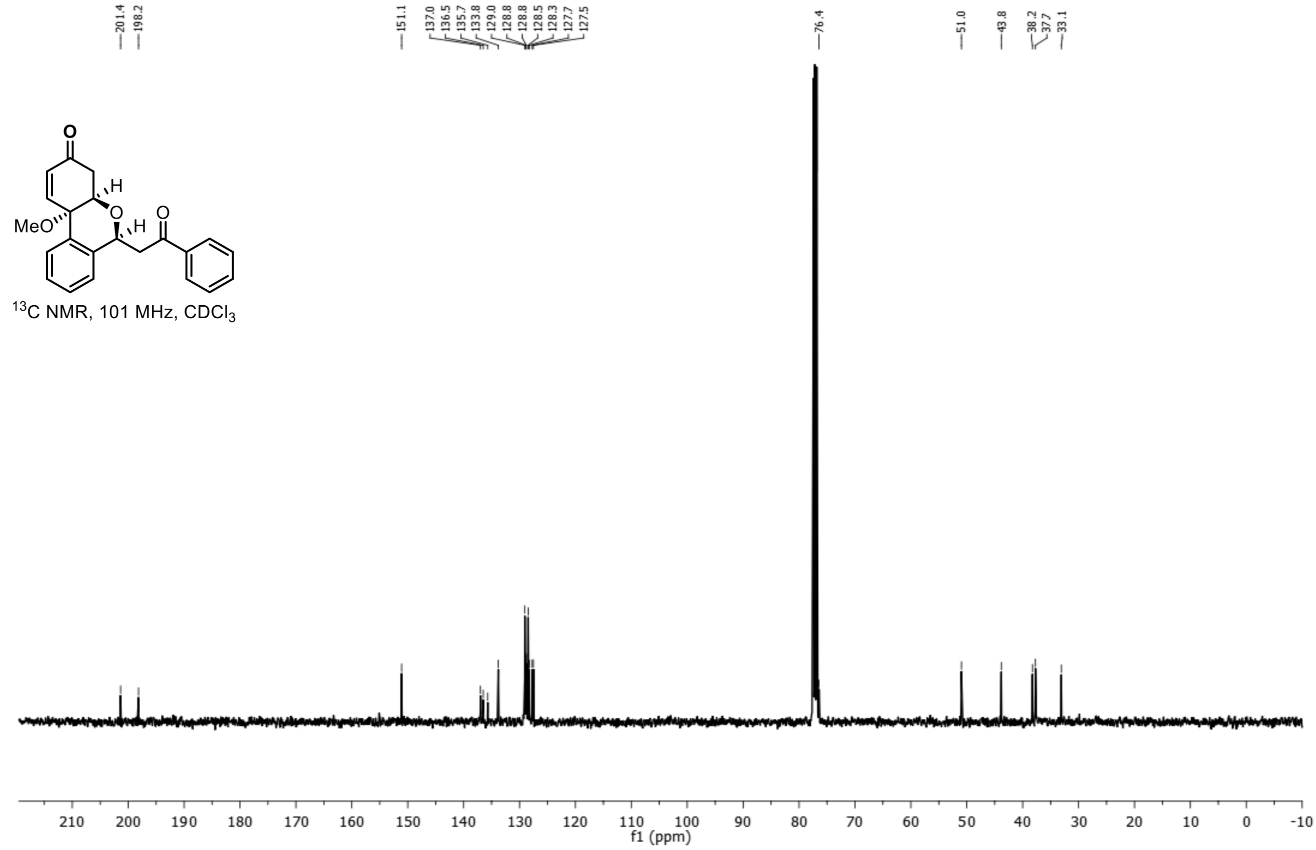

Supplementary Figure 79. **^{1}H NMR and ^{13}C NMR spectra of compound 11e.**

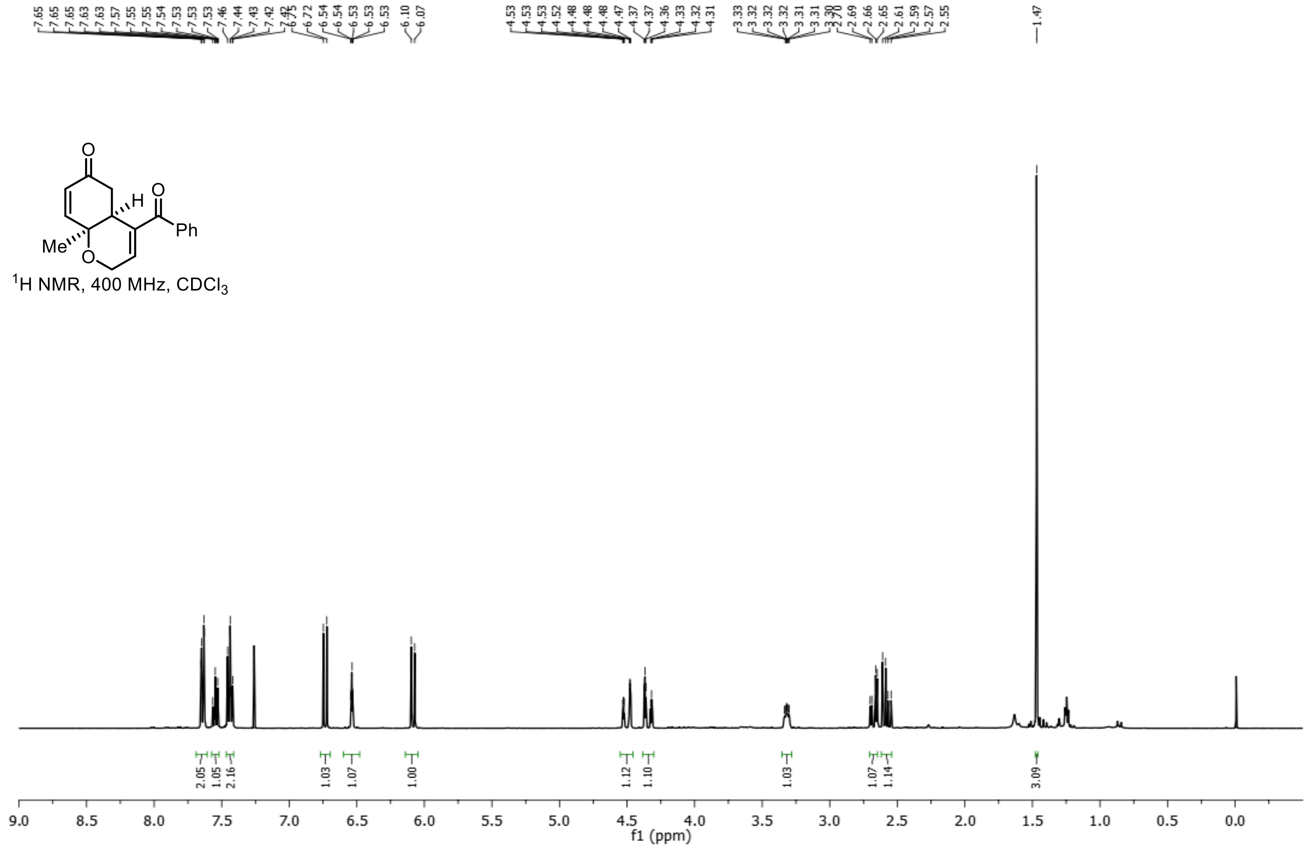

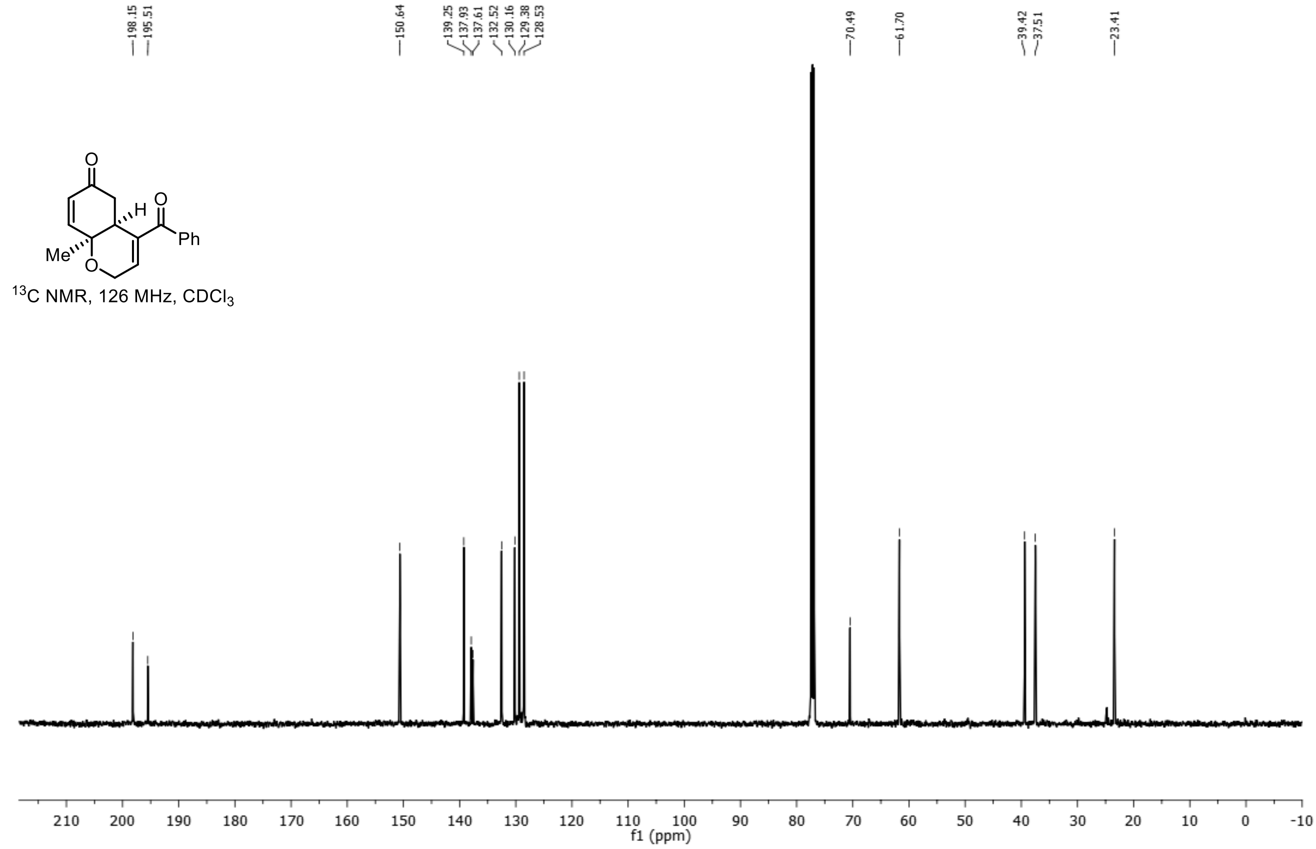

Supplementary Figure 80. **^{1}H NMR and ^{13}C NMR spectra of compound 12.**

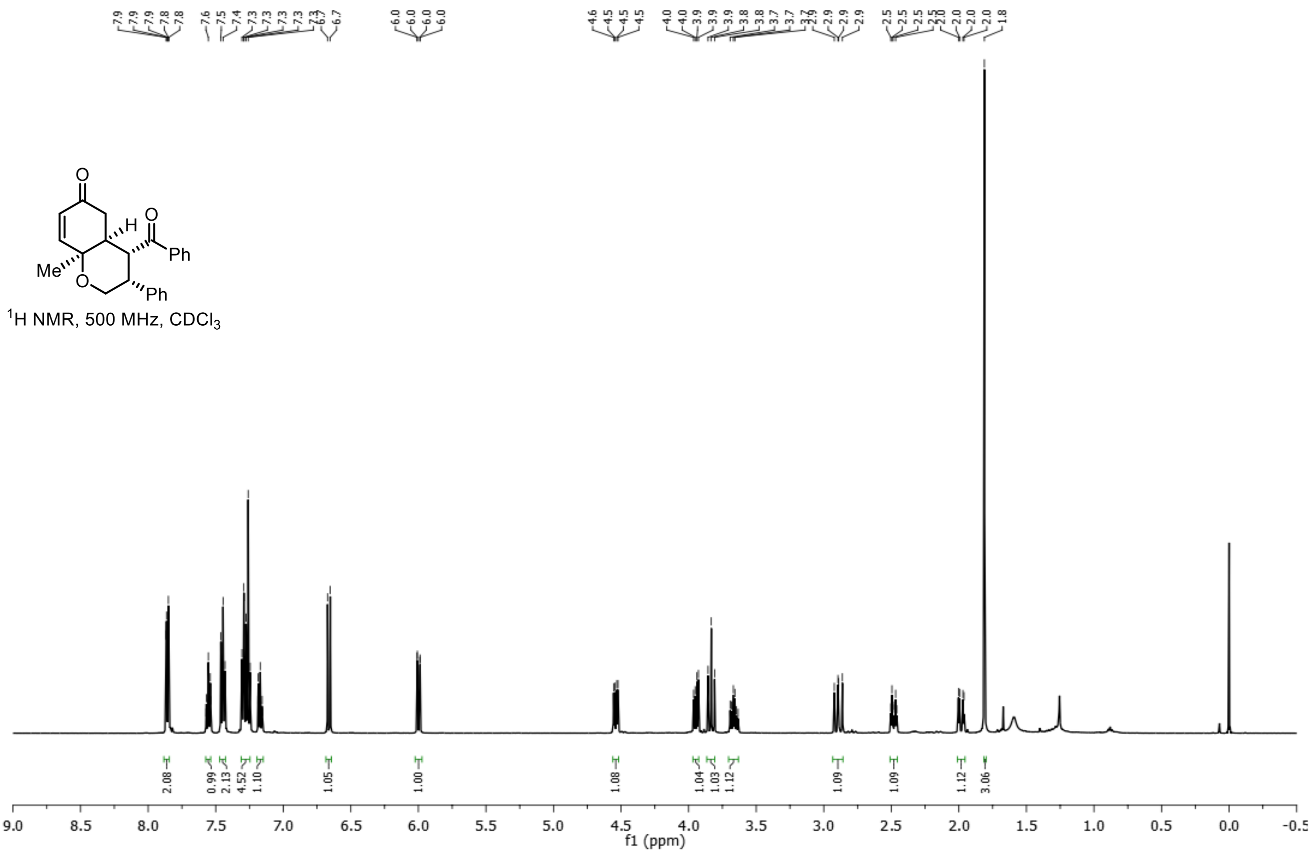

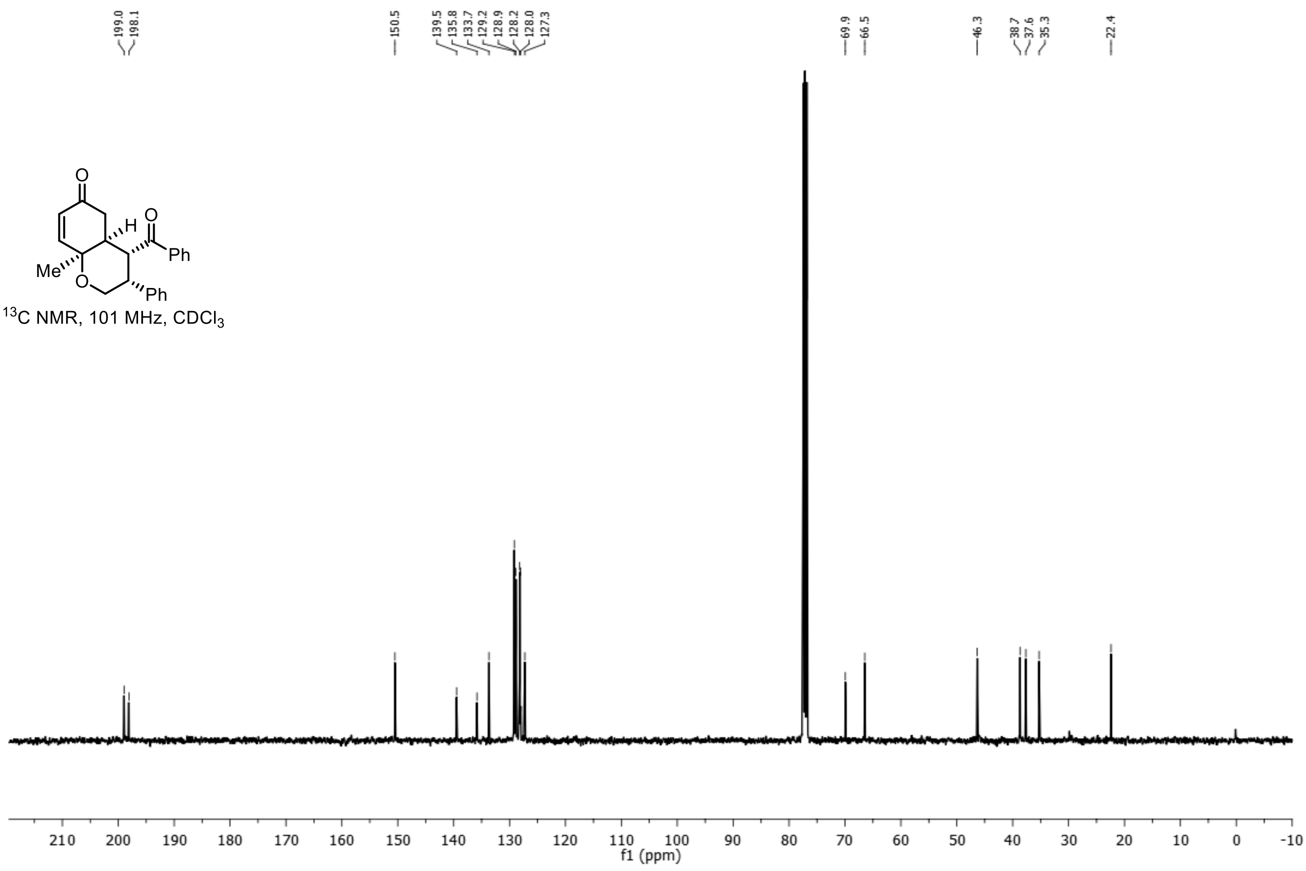

Supplementary Figure 81. **^{1}H NMR and ^{13}C NMR spectra of compound 13.**

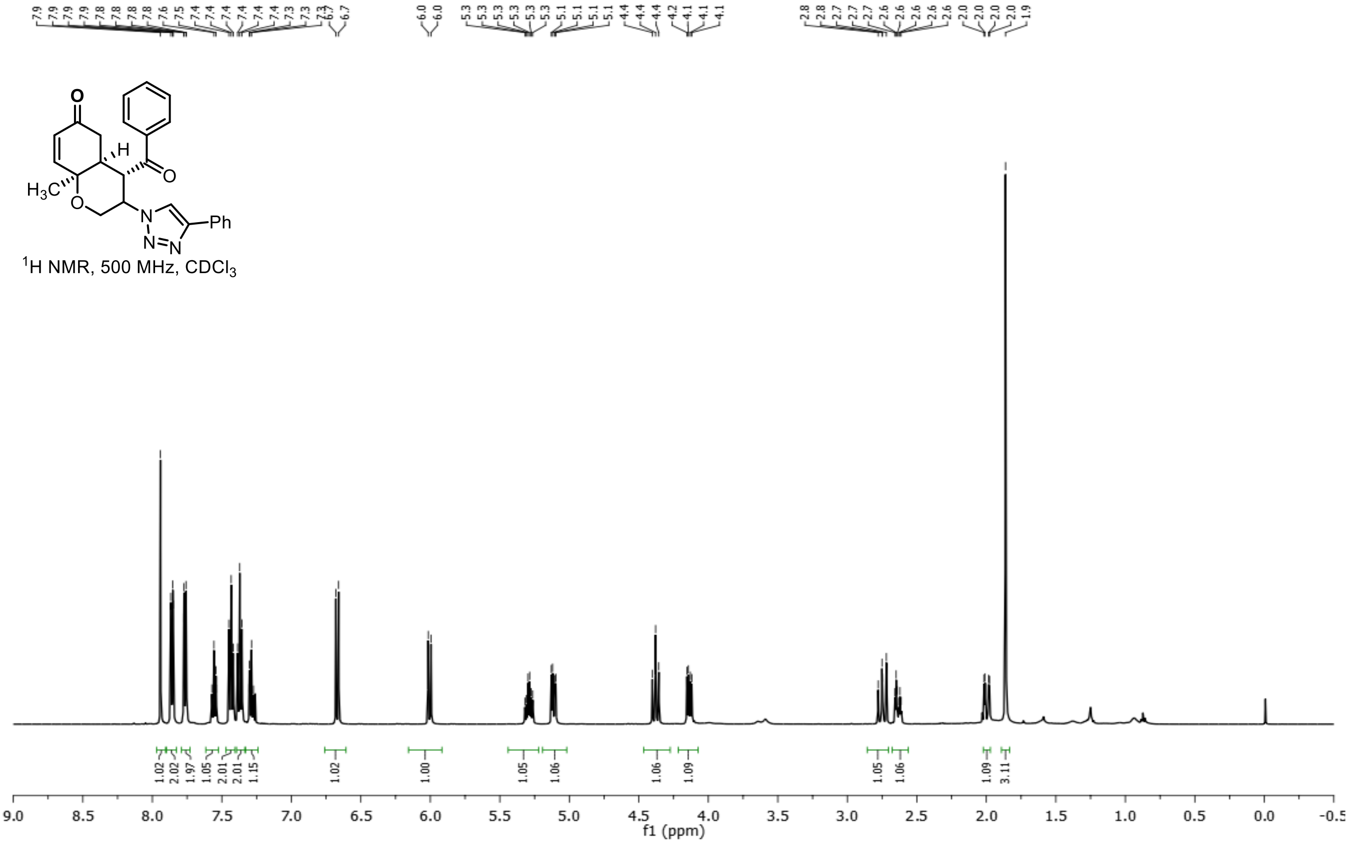

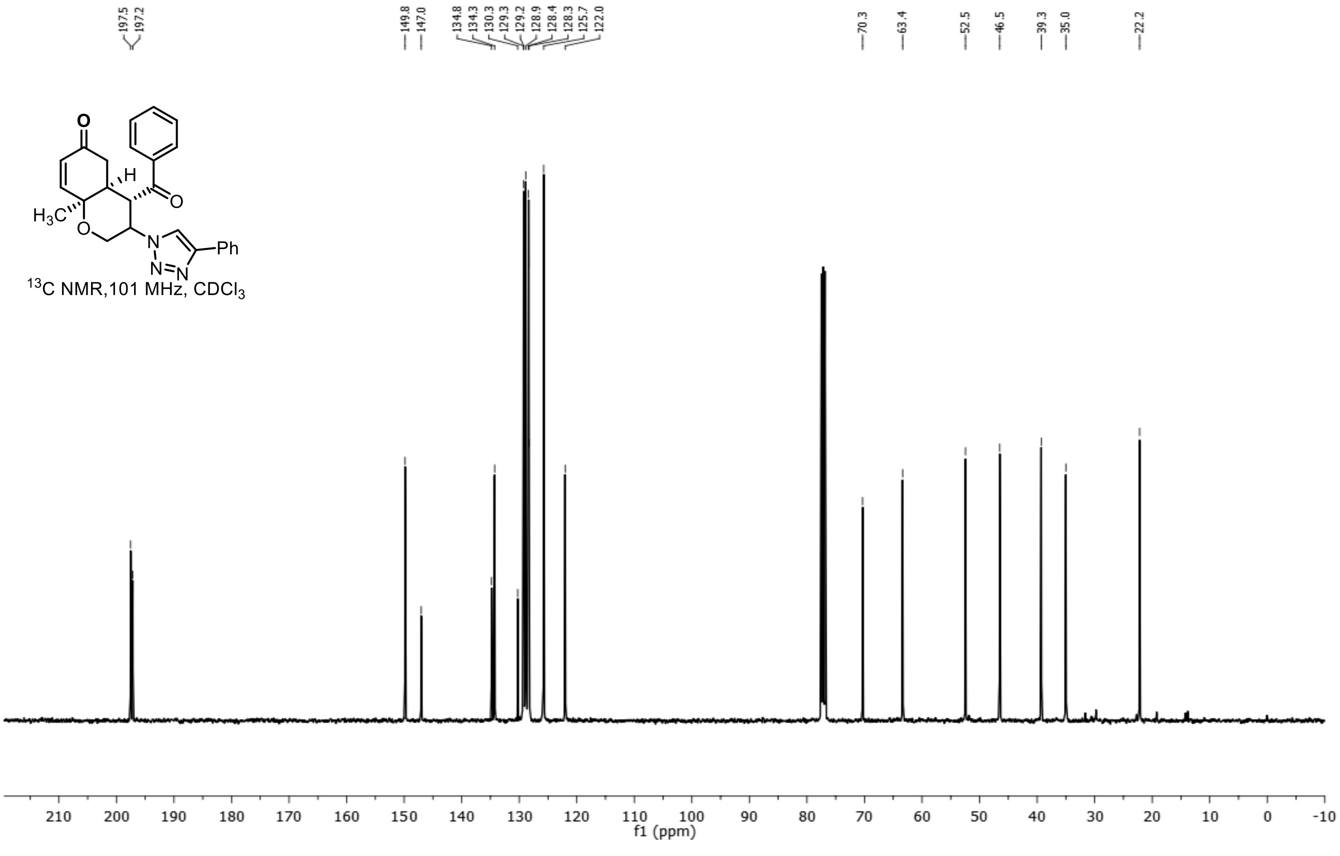

Supplementary Figure 82. **^{1}H NMR and ^{13}C NMR spectra of compound 14.**

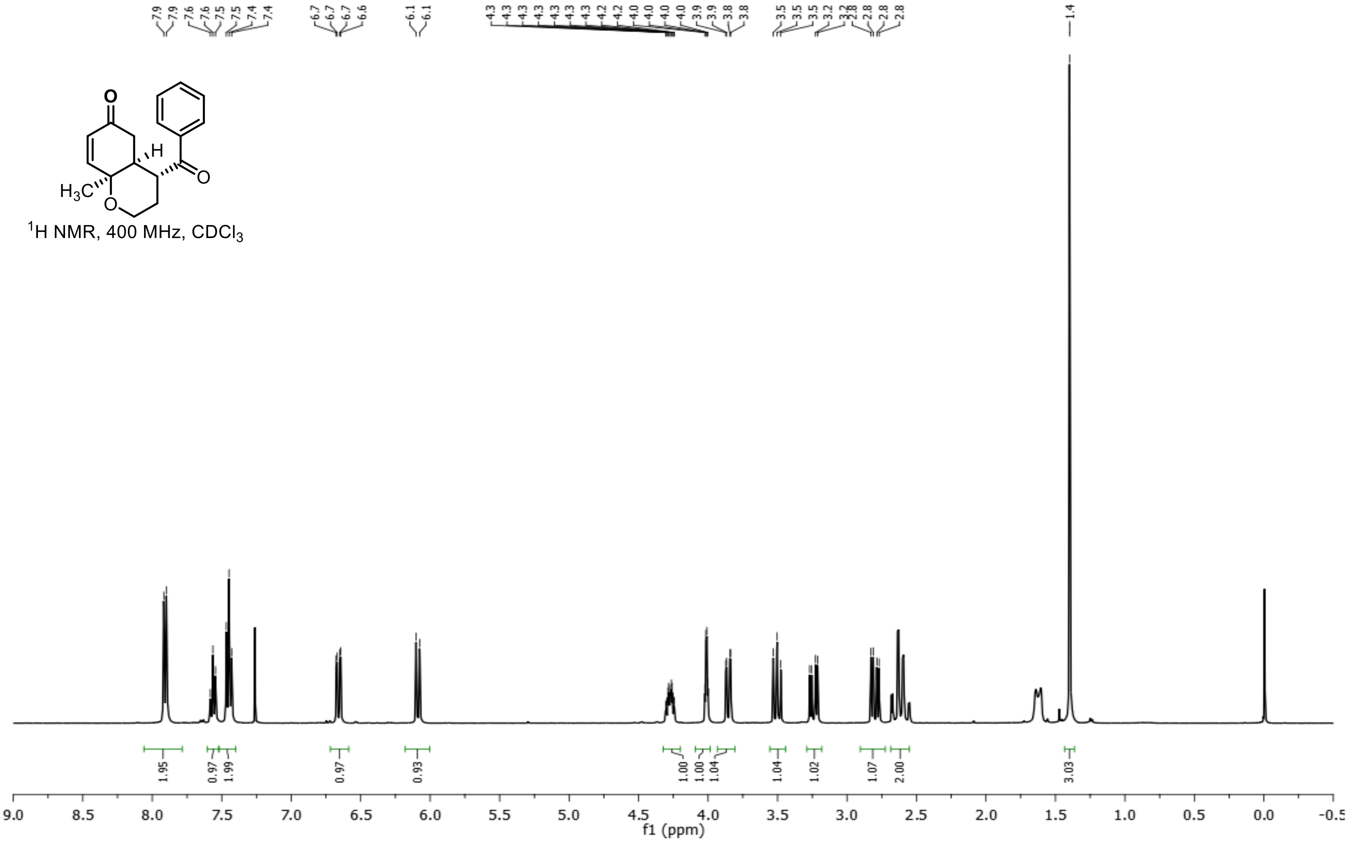

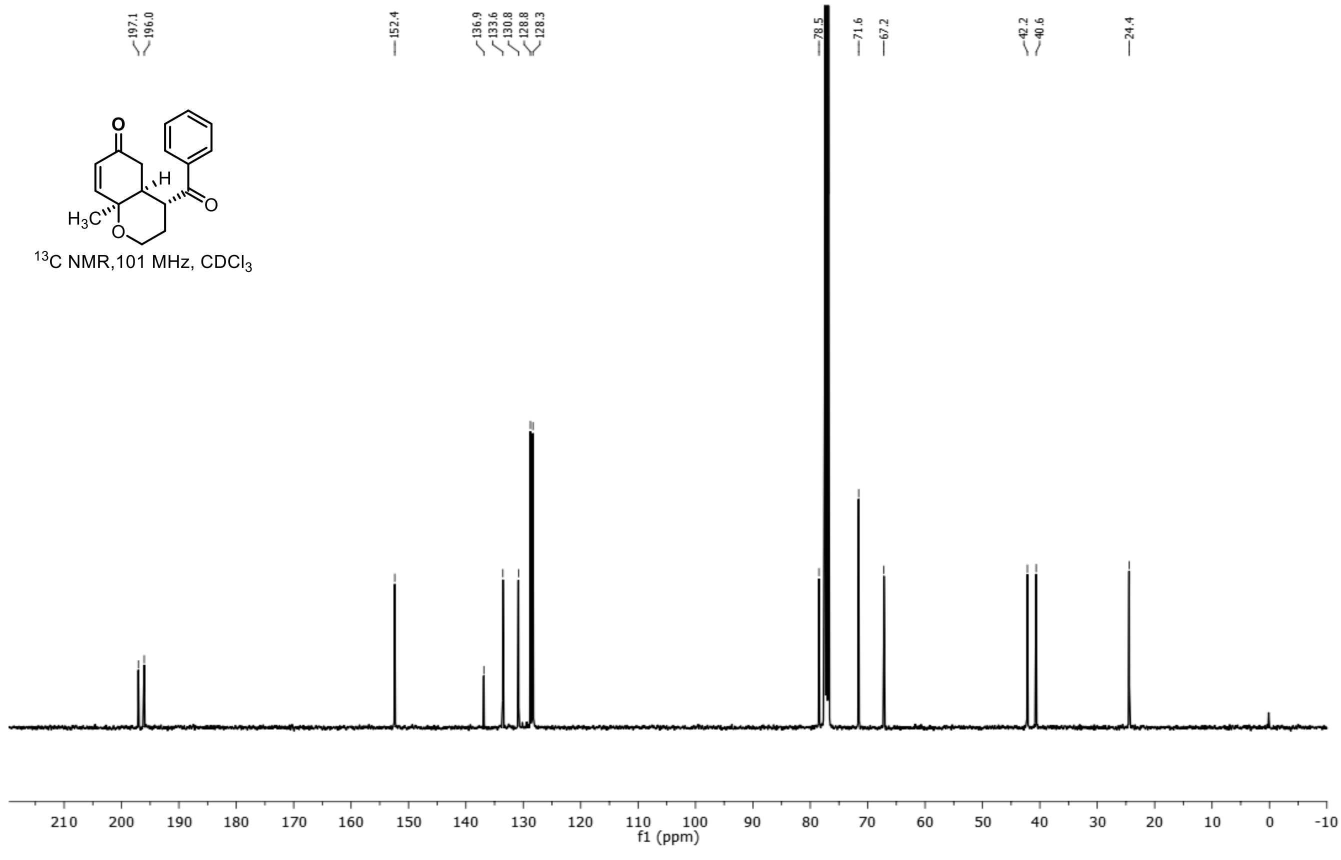

Supplementary Figure 83. **^{1}H NMR and ^{13}C NMR spectra of compound 15.**

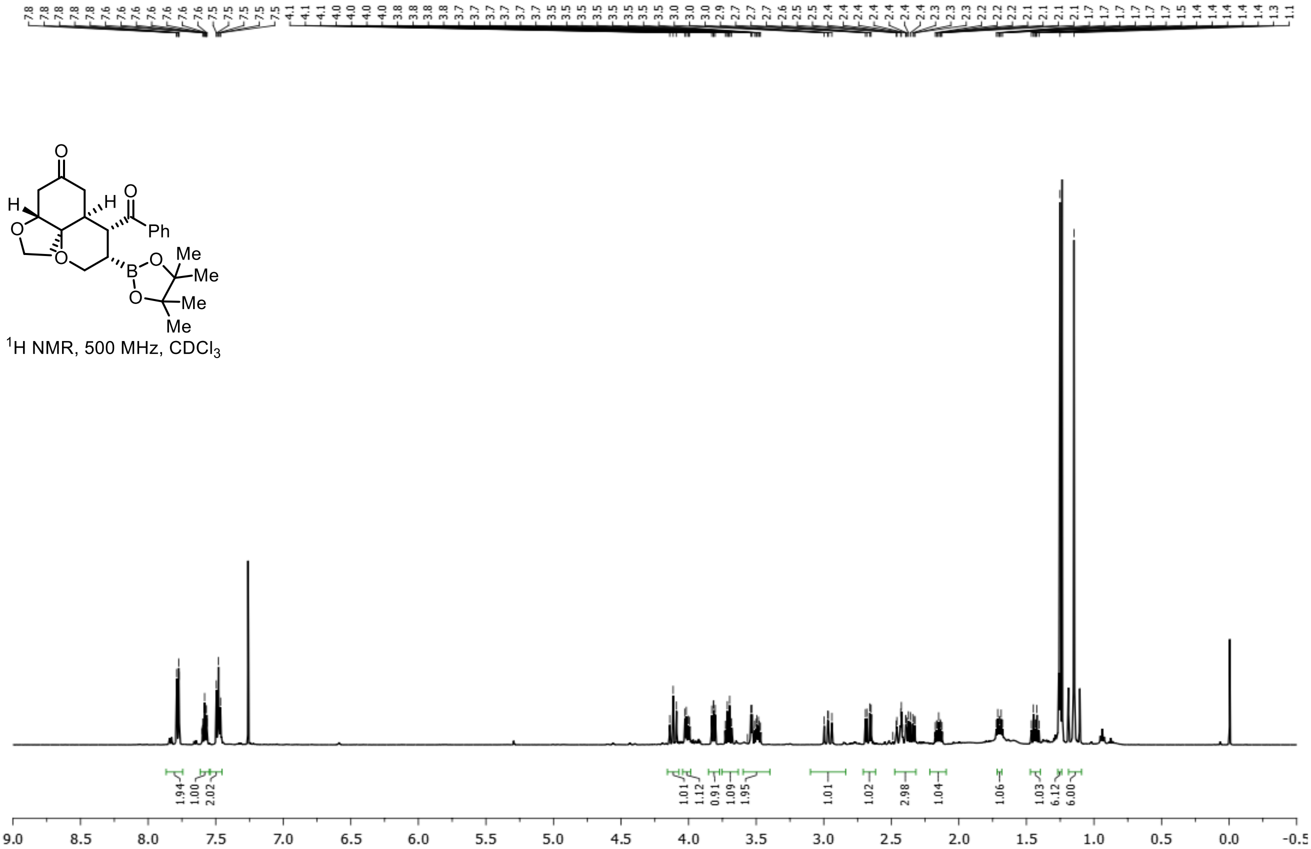

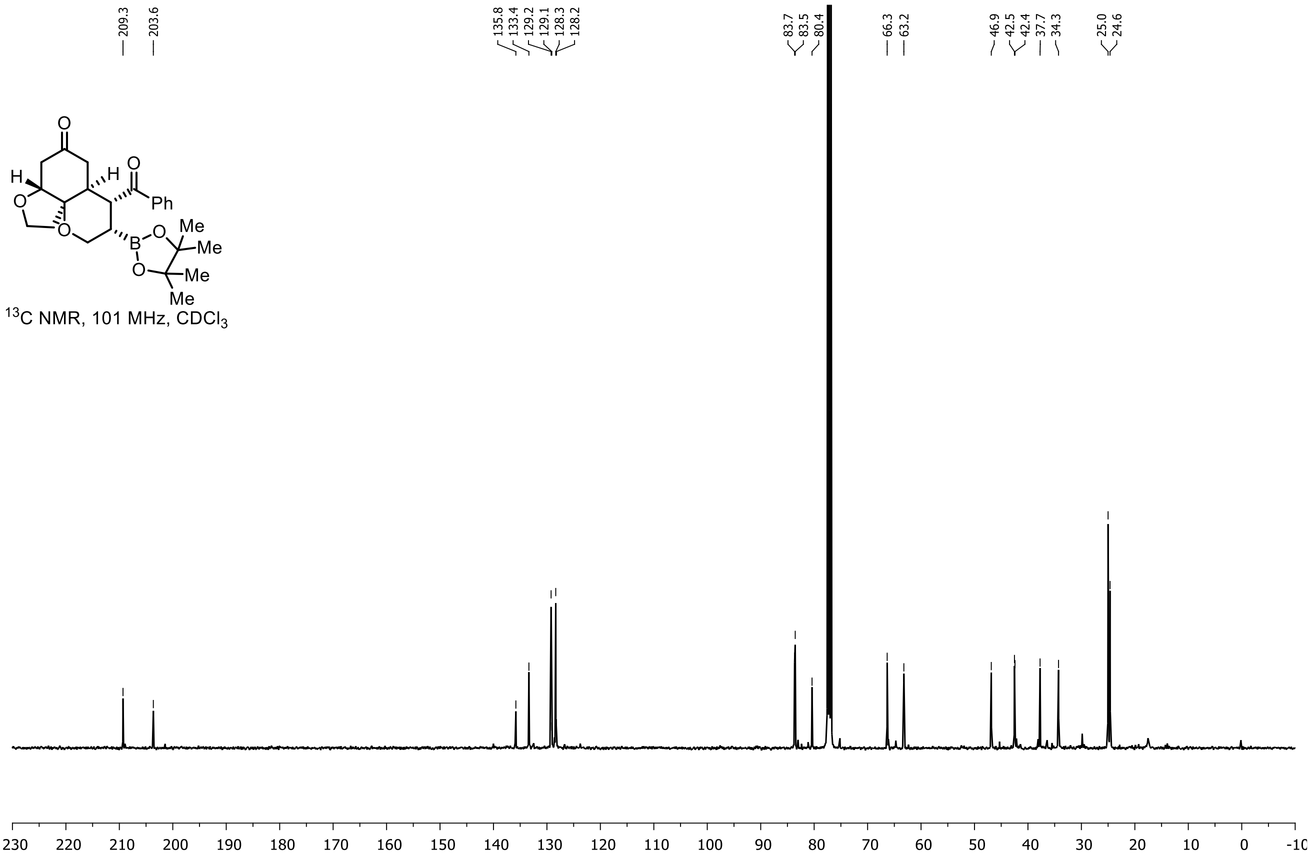

Supplementary Figure 84. ^{1}H NMR and ^{13}C NMR spectra of compound 16.

3. Supplementry References:

1. Burns A. R., González S. & Lam H. W. Enantioselective copper(I)-catalyzed borylative aldol cyclizations of enone diones. *Angew. Chem. Int. Ed.* **51**, 10827−10831 (2012).
2. Liu Q. & Rovis T. Asymmetric synthesis of hydrobenzofuranones via desymmetrization of cyclohexadienones using the intramolecular Stetter reaction. *J. Am. Chem. Soc.* **128**, 2552–2553 (2006).
3. Jadhav S. B., Thopate S. B., Nanubolu J. B. & Chegondi R. Rh-Catalyzed diastereoselective desymmetrization of enone tethered-cyclohexadienones via tandem arylative cyclization. *Org. Biomol. Chem.* **17**, 1937−1946 (2019).
4. Thopate S.B., Jadhav S.B., Nanubolu J. B. & Chegondi R. Stereoselective desymmetrization of cyclohexadienone-tethered enones: efficient access to highly strained polycyclic indoles. *ACS Catal.* **9**, 10012–10019 (2019).
5. Li K., Jin Z., Chan W.-L. & Lu Y. Enantioselective construction of bicyclic pyran and hydrindane scaffolds via intramolecular Rauhut–Currier reactions catalyzed by thiourea-phosphines. *ACS Catal.* **8**, 8810−8815 (2018).
6. Liu J., Gao Y., Wang L. & Du Y. Chiron approach for the total synthesis of (+)-synargentolide B. *Tetrahedron* **73**, 6443−6447 (2017).
7. SMART & SAINT. Software Reference manuals. Versions 6.28a & 5.625, Bruker Analytical X-ray Systems Inc., Madison, Wisconsin, U.S.A., 2001.
8. Sheldrick, G. M. SHELXS97 and SHELXL Version 2014/7, http://shelx.uni-ac.gwdg.de/SHELX/index.php
9. Muller P., Herbst-Imer R., Spek A. L., Schneider T. R., & Sawaya M. R. Crystal Structure Refinement: A Crystallographer's Guide to SHELXL. Muller, P. Ed. Oxford University Press: Oxford, New York, 57–91 (2006).
10. Spek A. L. Structure validation in chemical crystallography. *Acta Cryst.* **D65**, 148-155 (2009).
11. Flack H. D. On enantiomorph-polarity estimation. *Acta Cryst.* **A39**, 876-881 (1983).
12. Frisch M. J. et al., Gaussian 09, Revision A.01, Gaussian, Inc., Wallingford CT, 2009.
13. Becke A. D. Density-functional thermochemistry. III. The role of exact exchange. *J. Chem. Phys.* **98**, 5648−5652 (1993).
14. Zhao Y. & Truhlar D. G. The M06 suite of density functionals for main group thermochemistry, thermochemical kinetics, noncovalent interactions, excited states, and transition elements: two new functionals and systematic testing of four M06-class functionals and 12 other functionals. *Theoretical chemistry accounts* **120**, 215−241 (2008).

15. Marenich A. V., Cramer C. J. & Truhlar D. G. Universal solvation model based on solute electron density and on a continuum model of the solvent defined by the bulk dielectric constant and atomic surface tensions. *J. Phys. Chem. B.* **113**, 6378–6396 (2009).
16. Simón L. & Goodman J. M. How reliable are DFT transition structures? comparison of GGA, hybrid-meta-GGA and meta-GGA functionals. *Organic & biomolecular chemistry* **9**, 689−700 (2011).
17. Poater A., Pump E., Vummaleti S. V. C. & Cavallo L. The right computational recipe for olefin metathesis with Ru-based catalysts: the whole mechanism of ring-closing olefin metathesis. *Journal of chemical theory and computation*, **10**, 4442−4448 (2014).
