# Supplementary material for: Enantioselective Cu(I)-catalyzed borylative cyclization of enone-tethered cyclohexadienones and mechanistic insights
Source: Nat Commun. 2022 Feb 14;13:854. doi: 10.1038/s41467-022-28288-7 (PMC8844005; doi:10.1038/s41467-022-28288-7)
Supplement: Supplementary file 3 — Source Data [file 41467_2022_28288_MOESM3_ESM.pdf]

Supplementary Information

Enantioselective Cu(I)-Catalyzed Borylative Cyclization of Enone-Tethered Cyclohexadienones and Mechanistic Insights

Sandip B. Jadhav, Soumya Ranjan Dash, Sundaram Maurya, Jagadeesh Babu Nanubolu, Kumar Vanka and Rambabu Chegondi*

E-mail: rchegondi@iict.res.in

Optimized Cartesian Coordinates:

B_2Pin_2				Bpin-O^{t}Bu			
C	-0.858094	-0.605627	0.231969	C	-1.751352	0.398081	-0.610790
C	-1.727637	0.395303	-0.620451	C	-1.027485	-0.674384	0.295378
C	3.756752	2.610567	-2.157531	B	0.483468	0.825371	-0.530614
C	3.529774	3.500973	-0.876812	O	-0.739567	1.427609	-0.741113
B	0.474851	1.026880	-0.668196	O	0.371189	-0.467992	-0.046572
B	1.875396	1.923826	-1.043525	C	-3.003943	1.024538	-0.000410
O	0.500570	-0.251018	-0.161671	H	-3.411930	1.771288	-0.689219
O	-0.803841	1.502381	-0.838756	H	-3.776580	0.266941	0.174641
O	2.517610	2.745595	-0.147701	H	-2.783905	1.525377	0.945000
O	2.486225	1.904444	-2.275093	C	-2.055057	-0.114772	-2.025691
C	-1.076509	-2.086006	-0.075471	H	-2.865574	-0.851390	-2.026744
H	-0.401216	-2.691041	0.538077	H	-2.356277	0.731516	-2.650641
H	-2.105037	-2.386204	0.156458	H	-1.170276	-0.571915	-2.480335
H	-0.873986	-2.315953	-1.123894	C	-1.167612	-0.389317	1.796874
C	-0.950153	-0.363413	1.744730	H	-2.184986	-0.583352	2.152681
H	-1.924139	-0.664986	2.144869	H	-0.481097	-1.039078	2.348913
H	-0.174760	-0.951388	2.245452	H	-0.912369	0.649498	2.029385
H	-0.783916	0.690201	1.989728	C	-1.387061	-2.129181	0.000358
C	-2.097866	-0.142929	-2.009273	H	-0.808127	-2.790796	0.653125
H	-2.843751	-0.942708	-1.948286	H	-2.450730	-2.314624	0.188995
H	-2.516149	0.673742	-2.605728	H	-1.164243	-2.398165	-1.034679
H	-1.217869	-0.526557	-2.534815	O	1.632059	1.488981	-0.797158
C	-2.972129	0.938810	0.079252	C	2.977895	0.992244	-0.587464
H	-3.480972	1.650508	-0.578845	C	3.207347	-0.250912	-1.457681
H	-3.675013	0.130226	0.311464	C	3.191765	0.684434	0.900253
H	-2.720685	1.459350	1.006019	C	3.885481	2.141458	-1.038048
C	2.912161	4.871950	-1.184345	H	3.014695	-0.015983	-2.510374
H	2.600325	5.338769	-0.244924	H	4.244607	-0.593327	-1.368822
H	3.628040	5.537100	-1.679119	H	2.546322	-1.067546	-1.154622
H	2.027769	4.775879	-1.821825	H	2.990084	1.576022	1.504297
C	4.749516	3.667269	0.027797	H	2.528700	-0.119784	1.229901
H	5.559178	4.186159	-0.498492	H	4.227847	0.376502	1.080792
H	4.477400	4.265592	0.903292	H	4.940710	1.869221	-0.926359
H	5.124657	2.704734	0.382589	H	3.697719	2.385514	-2.088773
C	4.000016	3.381005	-3.454060	H	3.691093	3.037691	-0.439860
H	4.921946	3.970853	-3.391612				
H	4.105397	2.675909	-4.284880				
H	3.171072	4.052969	-3.687295				
C	4.836737	1.535622	-1.973885				

H	4.791366	0.840111	-2.817613	
H	5.840905	1.971742	-1.939926	
H	4.674885	0.961822	-1.056105	

LiO^{t}Bu				Substrate (1a)			
C	-0.997952	-5.330525	-2.943440	O	12.258448	8.781504	-0.061057
C	-2.218347	-5.670394	-3.826668	C	11.360507	9.146296	0.689467
H	-3.134375	-5.291929	-3.357213	O	7.975188	10.635099	2.432473
H	-2.115489	-5.187688	-4.805993	C	10.227669	8.256994	1.044045
H	-2.331908	-6.750985	-3.984436	H	10.227956	7.276192	0.576226
C	0.287692	-5.857934	-3.617506	C	9.261255	8.648207	1.885454
H	0.419496	-5.377178	-4.594161	H	8.428194	7.991252	2.131506
H	1.160317	-5.613497	-2.999395	O	3.891940	13.102712	0.918406
H	0.265100	-6.945217	-3.767544	C	9.252992	9.987626	2.586188
C	-1.162757	-5.997656	-1.560098	C	9.376464	9.768187	4.107584
H	-0.305350	-5.749289	-0.922809	H	9.329454	10.732586	4.623179
H	-2.068983	-5.621114	-1.070600	H	10.320939	9.275101	4.353369
H	-1.236390	-7.090735	-1.629762	H	8.545299	9.147996	4.457827
O	-0.908124	-3.955466	-2.785989	C	10.368731	10.894547	2.119126
Li	-0.803847	-2.365848	-2.604954	H	10.353869	11.899826	2.537748
				C	11.337017	10.507582	1.277853
				H	12.146475	11.168935	0.980848
				C	4.275115	12.728236	2.024110
				C	7.593416	10.956439	1.102640
				H	8.356300	11.586663	0.615137
				H	7.513629	10.046022	0.484770
				C	6.285110	11.675448	1.096185
				H	5.905561	11.963182	0.116659
				C	5.559877	11.991244	2.176592
				H	5.922082	11.720759	3.160626
				C	3.721690	12.501004	4.515868
				H	4.563689	11.835267	4.672097
				C	2.901448	12.813532	5.600215
				H	3.120868	12.397246	6.579681
				C	1.803244	13.656939	5.427857
				H	1.167687	13.902475	6.274642
				C	2.335465	13.866427	3.080987
				H	2.132327	14.258313	2.089938
				C	1.521736	14.183408	4.163348
				H	0.666494	14.839388	4.024876
				C	3.449179	13.025280	3.242585

PPh_2CuO^tBu (Original Ligand)							
P	-1.212973	1.519637	-0.094213	C	-1.015025	-1.756559	-0.411003
P	1.649155	-0.761835	-0.255503	C	-1.985705	-2.479562	-1.086163
C	-2.302493	1.179397	-1.546372	C	-1.752571	-3.160776	-2.278952
C	-3.564103	0.571584	-1.450387	C	-1.390577	-1.146801	0.906380
H	-3.920610	0.209564	-0.490678	C	-1.655544	0.237798	1.164171
C	-4.359871	0.412591	-2.586756	C	-2.077635	0.640159	2.438768

H	-5.341581	-0.046774	-2.497090
C	-3.904813	0.851957	-3.832644
H	-4.526400	0.727040	-4.715363
C	-2.647965	1.450287	-3.939130
H	-2.285375	1.791229	-4.905221
C	-1.851725	1.610792	-2.803880
H	-0.873414	2.077806	-2.890476
C	-1.907416	3.099643	0.562475
C	-3.286958	3.337699	0.693316
H	-4.003250	2.572722	0.407928
C	-3.748115	4.558703	1.182117
H	-4.816752	4.733256	1.279102
C	-2.837027	5.557947	1.539609
H	-3.198311	6.511448	1.917043
C	-1.468017	5.331619	1.401394
H	-0.757011	6.109975	1.666297
C	-0.995328	4.108941	0.912642
H	0.075262	3.942157	0.780231
C	3.110864	-1.129038	-1.325360
C	3.337272	-0.282842	-2.424332
H	2.666824	0.553630	-2.604857
C	4.421331	-0.495874	-3.276041
H	4.581284	0.168754	-4.120616
C	5.307013	-1.547633	-3.030601
H	6.159078	-1.707430	-3.685884
C	5.099522	-2.385927	-1.933916
H	5.789292	-3.201413	-1.732636
C	4.007138	-2.181966	-1.087892
H	3.860770	-2.840471	-0.237852
C	2.029850	-1.624760	1.330577
C	2.548789	-0.864351	2.390190
H	2.680336	0.207109	2.267139
C	2.892775	-1.475095	3.597511
H	3.293442	-0.874501	4.409538
C	2.718520	-2.850738	3.761042
H	2.987675	-3.326442	4.700487
C	2.194027	-3.614274	2.714444
H	2.059201	-4.686363	2.835592
C	1.847160	-3.005688	1.506480
H	1.426175	-3.603467	0.703709
Cu	1.172676	1.432865	-0.148381
C	-0.507904	-3.173592	-2.867249
C	0.506168	-2.461581	-2.202825
C	0.287327	-1.766622	-1.006114
H	-2.259605	1.691930	2.624554
C	-2.272993	-0.261748	3.499760
C	-2.023378	-1.589016	3.232082
H	-0.317455	-3.708626	-3.791046
H	1.495723	-2.457227	-2.643577
C	-1.605237	-2.008478	1.970938
C	-1.623526	-3.782234	3.332506
O	-1.468597	-3.377073	1.969491
O	-2.158683	-2.672846	4.062184
H	-2.604721	0.072222	4.476753
C	-3.923342	-3.361432	-1.784602
H	-4.446759	-4.238249	-1.391706
H	-4.615131	-2.687182	-2.305888
O	-3.294923	-2.660326	-0.708107
O	-2.899774	-3.785521	-2.692423
H	-0.642246	-4.052249	3.745856
H	-2.321850	-4.622001	3.384886
O	1.870160	3.159958	0.074695
C	3.188952	3.622179	0.180153
C	3.987463	2.814935	1.226253
C	3.903349	3.534978	-1.186229
C	3.128404	5.100844	0.624030
H	3.473355	2.851687	2.194777
H	4.061892	1.765468	0.915139
H	5.006640	3.201616	1.362198
H	3.332467	4.092348	-1.938157
H	4.922053	3.944788	-1.150694
H	3.968859	2.490019	-1.512272
H	4.125739	5.551955	0.710342
H	2.546950	5.681266	-0.101519
H	2.629752	5.177920	1.597713

$PPh_2CuBPin$ (Original Ligand)			
P	-0.079598	1.797933	0.065819
P	-0.079174	-1.797669	-0.066169
C	-0.733522	2.481160	-1.519141
C	-1.983528	3.107969	-1.640627
H	-2.649508	3.162309	-0.784697
C	-2.386662	3.648239	-2.863614
H	-3.351945	4.143104	-2.941625
C	-1.551359	3.563628	-3.980339
O	4.268138	0.391292	1.071878
C	5.647951	-0.046500	-0.784094
C	5.648120	0.047099	0.784168
C	6.553609	-1.127891	-1.375344
C	5.927353	1.298401	-1.473725
C	5.927478	-1.297820	1.473787
C	6.554037	1.128387	1.375202
H	6.450833	-1.135744	-2.466009

H	-1.867660	3.984573	-4.931126	H	7.607470	-0.936912	-1.137705
C	-0.310657	2.932647	-3.871103	H	6.288774	-2.121718	-1.006151
H	0.341628	2.856620	-4.736957	H	6.973346	1.607469	-1.363665
C	0.095503	2.393275	-2.649378	H	5.708524	1.199840	-2.542099
H	1.060832	1.899160	-2.568788	H	5.284883	2.087133	-1.070230
C	0.641174	3.292788	0.885856	H	6.973398	-1.607055	1.363493
C	0.036085	4.560190	0.839381	H	5.708927	-1.199171	2.542211
H	-0.895011	4.699055	0.298302	H	5.284789	-2.086475	1.070493
C	0.627798	5.651378	1.474831	H	6.451457	1.136314	2.465885
H	0.150644	6.626971	1.427821	H	7.607825	0.937238	1.137380
C	1.832726	5.491674	2.165457	H	6.289281	2.122237	1.006006
H	2.293913	6.343689	2.658505	C	-2.783883	-1.644867	-3.215473
C	2.445598	4.239423	2.209997	C	-1.753126	-2.004867	-2.329185
H	3.389009	4.109481	2.733473	C	-1.576465	-1.397619	-1.078336
C	1.858150	3.144634	1.569874	C	-2.462732	-0.350107	-0.663216
H	2.360929	2.181125	1.577463	C	-3.474580	-0.032345	-1.555279
C	0.642032	-3.292111	-0.886593	C	-3.633866	-0.647137	-2.795251
C	1.859050	-3.143489	-1.570427	C	-2.462583	0.349571	0.663626
H	2.361619	-2.179869	-1.577738	C	-1.576512	1.397409	1.078356
C	2.446851	-4.237977	-2.210744	C	-1.752938	2.004680	2.329229
H	3.390296	-4.107662	-2.734066	H	-1.069527	2.787327	2.635650
C	1.834285	-5.490391	-2.166590	C	-2.783190	1.644316	3.215960
H	2.295750	-6.342172	-2.659782	C	-3.632942	0.646222	2.796142
C	0.629301	-5.650555	-1.476166	H	-2.903757	-2.131194	-4.177337
H	0.152373	-6.626272	-1.429454	H	-1.069487	-2.787183	-2.635936
C	0.037245	-4.559669	-0.840513	C	-3.473950	0.031473	1.556111
H	-0.893892	-4.698902	-0.299595	C	-5.181414	-0.956257	2.619585
C	-0.732529	-2.481395	1.518819	O	-4.469542	-0.901196	1.380351
C	0.096767	-2.393508	2.648857	O	-4.725473	0.124681	3.440121
H	1.061959	-1.899156	2.568098	H	-2.902872	2.130662	4.177838
C	-0.308967	-2.933167	3.870599	C	-5.182432	0.955135	-2.618269
H	0.343524	-2.857136	4.736296	H	-6.253911	0.840455	-2.427779
C	-1.549514	-3.564415	3.980054	H	-4.969731	1.908150	-3.120321
H	-1.865498	-3.985553	4.930861	O	-4.470490	0.899925	-1.379093
C	-2.385069	-3.649052	2.863521	O	-4.726927	-0.126057	-3.438718
H	-3.350230	-4.144129	2.941699	H	-4.969037	-1.909404	3.121508
C	-1.982350	-3.108526	1.640512	H	-6.252878	-0.841181	2.429179
H	-2.648523	-3.162925	0.784737				
Cu	1.445574	0.000193	0.000158				
B	3.454971	0.000410	0.000299				
O	4.267861	-0.390510	-1.071511				

PMe_2CuO^tBu (Modified Ligand)							
P	-0.717762	-0.973422	-1.513214	C	-6.222415	-0.470840	-1.082817
P	-0.788854	0.843181	1.584755	H	-5.230858	-1.842440	1.096324
C	-0.060891	-0.117885	-3.028510	H	-4.573294	-0.450655	1.977724
C	-1.527164	-2.450272	-2.274808	H	-6.328104	-0.578500	1.703526
C	-1.365280	2.197218	2.713219	H	-5.131539	2.048866	-0.776323
C	-0.064817	-0.321584	2.836505	H	-6.281618	1.776658	0.556223
Cu	-2.208574	0.032016	0.076917	H	-4.533229	1.846898	0.882038
C	1.778227	3.633924	-0.090999	H	-7.241258	-0.278097	-0.721372
C	0.777957	2.973627	0.643828	H	-6.087260	0.053034	-2.036132
C	0.667358	1.576767	0.708664	H	-6.118875	-1.545747	-1.270971
C	1.598816	0.759993	-0.011316				

C	2.581650	1.444308	-0.713254				
C	2.674740	2.832757	-0.762309				
C	1.679007	-0.737140	-0.006374				
C	0.797718	-1.635812	-0.688383				
C	1.033372	-3.016618	-0.627046				
H	0.361722	-3.694563	-1.140466				
C	2.117040	-3.579787	0.070869				
C	2.966591	-2.697391	0.701277				
H	1.841510	4.715938	-0.123841				
H	0.067177	3.590842	1.179963				
C	2.749908	-1.322265	0.653478				
C	4.666616	-1.695278	1.747204				
O	3.722230	-0.682714	1.387252				
O	4.078455	-2.968254	1.459023				
H	2.278204	-4.651680	0.106343				
C	4.402600	1.987676	-1.901937				
H	5.370302	1.943622	-1.383882				
H	4.537502	1.943851	-2.986767				
O	3.582658	0.894198	-1.479345				
O	3.730614	3.203856	-1.554195				
H	4.882369	-1.627806	2.817124				
H	5.582528	-1.571967	1.152363				
H	-2.411381	-2.078867	-2.801770				
H	-0.881978	-2.993733	-2.974473				
H	-1.885112	-3.127140	-1.494164				
H	-2.110631	1.756259	3.383099				
H	-0.556079	2.628049	3.313421				
H	-1.865023	2.987038	2.146234				
H	-0.869897	-0.628079	3.512948				
H	0.326077	-1.215146	2.347055				
H	0.733593	0.152923	3.417222				
H	-0.916660	0.219444	-3.623024				
H	0.521618	0.760614	-2.740323				
H	0.564181	-0.781035	-3.636512				
O	-3.894966	-0.309561	-0.634548				
C	-5.143589	-0.011201	-0.077210				
C	-5.340952	-0.763489	1.257766				
C	-5.291165	1.507686	0.163686				

PMe$_2$Bpin (Modified Ligand, LCu-Bpin)							
P	0.029378	0.797409	1.574664	H	5.568081	-1.969438	2.742133
P	0.029573	-0.798058	-1.574559	O	4.478168	-0.883939	1.357830
C	0.723568	-0.330984	2.879404	O	4.710802	-3.185956	1.302292
C	-0.642476	2.150190	2.647448	H	5.567516	1.970583	-2.742139
C	-0.641952	-2.151091	-2.647231	H	6.290069	1.859129	-1.089874
C	0.723370	0.330474	-2.879383	H	-1.433184	1.702491	3.258077
Cu	-1.537902	-0.000372	-0.000052	H	0.111455	2.596929	3.305628
B	-3.544745	-0.000172	-0.000073	H	-1.110241	2.926896	2.036179
O	-4.356303	-1.137221	0.093354	H	-1.432830	-1.703651	-3.257828
O	-4.356048	1.137064	-0.093453	H	0.112066	-2.597642	-3.305438
C	-5.734360	-0.731476	0.285548	H	-1.109448	-2.927907	-2.035895
C	-5.734212	0.731636	-0.285549	H	-0.113765	0.703397	-3.479256
C	-6.642022	-1.720738	-0.447509	H	1.216492	1.187385	-2.416247
C	-6.014663	-0.794326	1.796024	H	1.434317	-0.186311	-3.532999

C	-6.014610	0.794538	-1.796004	H	-0.113432	-0.704187	3.479291
C	-6.641598	1.721108	0.447566	H	1.216931	-1.187725	2.416213
H	-6.543453	-2.713694	0.004643	H	1.434380	0.185982	3.533025
H	-7.695084	-1.420666	-0.377789				
H	-6.373897	-1.806420	-1.503296				
H	-7.060353	-0.563525	2.031190				
H	-5.795848	-1.805858	2.153649				
H	-5.371208	-0.097748	2.342340				
H	-7.060372	0.563982	-2.031090				
H	-5.795581	1.806014	-2.153659				
H	-5.371362	0.097799	-2.342358				
H	-6.542831	2.714040	-0.004597				
H	-7.694735	1.421280	0.377917				
H	-6.373384	1.806734	1.503335				
C	2.727271	-3.610665	-0.123026				
C	1.682159	-2.950363	-0.794296				
C	1.521872	-1.556794	-0.782715				
C	2.445075	-0.747818	-0.043723				
C	3.476260	-1.428056	0.587640				
C	3.619112	-2.813322	0.558864				
C	2.444831	0.747955	0.043725				
C	1.521402	1.556624	0.782770				
C	1.681232	2.950247	0.794347				
H	0.977879	3.562955	1.345311				
C	2.726093	3.610894	0.123026				
C	3.618160	2.813847	-0.558912				
H	2.827898	-4.690215	-0.146618				
H	0.978982	-3.563305	-1.345225				
C	3.475760	1.428533	-0.587688				
C	5.361490	1.965662	-1.668057				
O	4.477800	0.884752	-1.357947				
O	4.709684	3.186838	-1.302408				
H	2.826365	4.690477	0.146616				
C	5.362171	-1.964572	1.668025				
H	6.290780	-1.857733	1.089952				

TS1							
P	-0.559936	0.031091	1.783580	H	1.333053	-0.560639	3.157541
P	-1.519868	0.525620	-1.675167	H	-0.151247	-1.151982	3.943367
C	-0.769814	1.554935	2.823937	H	0.557344	-2.029275	2.558376
C	0.364497	-1.040010	2.983155	H	-0.630731	0.624382	-3.896269
C	-1.124595	1.374803	-3.269265	H	-2.012304	1.752205	-3.789780
C	-2.760229	-0.723791	-2.290296	H	-0.408018	2.180419	-3.090688
Cu	0.427390	0.500717	-0.278005	H	-3.614019	-0.237721	-2.775360
B	1.828649	1.833468	-0.861010	H	-2.251448	-1.366890	-3.014692
O	1.501941	2.950233	-1.640897	H	-3.118639	-1.352442	-1.472715
O	3.210513	1.830440	-0.630760	H	0.227684	1.951225	3.041265
C	2.659561	3.817242	-1.765525	H	-1.320271	2.315523	2.266385
C	3.843942	2.827147	-1.478552	H	-1.292086	1.342154	3.763064
C	2.654200	4.442157	-3.161049	O	5.746616	-1.130060	4.917386
C	2.514230	4.917210	-0.702528	C	5.666788	-1.261978	3.699521
C	4.343258	2.089713	-2.730714	O	4.539617	-1.657499	-0.222919
C	5.028754	3.435979	-0.727905	C	5.676163	-0.098868	2.781585
H	1.771258	5.080710	-3.273982	H	5.722778	0.879448	3.252624
H	3.542318	5.066275	-3.319205	C	5.605748	-0.246133	1.450595

H	2.620386	3.680625	-3.943809	H	5.567311	0.616718	0.788884
H	3.317399	5.660281	-0.768049	O	0.682758	-1.913075	-3.214945
H	1.558793	5.430245	-0.852894	C	5.585070	-1.590025	0.759616
H	2.512072	4.492265	0.305925	C	6.882000	-1.760433	-0.060543
H	4.907405	2.749607	-3.399798	H	6.846215	-2.707870	-0.607723
H	4.999524	1.270194	-2.419439	H	7.760146	-1.753431	0.591429
H	3.509395	1.654951	-3.290533	H	6.964597	-0.946590	-0.787689
H	5.800297	2.673920	-0.569194	C	5.463634	-2.739697	1.735424
H	5.483678	4.251286	-1.303520	H	5.346842	-3.721153	1.278035
H	4.732037	3.824493	0.249329	C	5.524763	-2.595697	3.066528
C	-3.343181	4.064288	-0.468027	H	5.466478	-3.441804	3.746314
C	-2.624242	3.073963	-1.160715	C	0.481414	-2.567683	-2.190126
C	-2.568587	1.735786	-0.745041	C	3.225340	-1.340878	0.257766
C	-3.247347	1.340886	0.451831	H	2.933212	-2.026503	1.066862
C	-3.967342	2.336005	1.097949	H	3.206399	-0.314692	0.636869
C	-4.017387	3.657101	0.662106	C	2.293965	-1.471176	-0.910906
C	-3.308925	-0.040586	1.029831	C	1.269532	-2.352032	-0.957164
C	-2.254862	-0.711179	1.729964	C	-1.162154	-4.157834	-1.038973
C	-2.484894	-1.980423	2.279874	H	-0.911741	-3.743983	-0.067490
H	-1.688235	-2.483316	2.815664	C	-2.135217	-5.154736	-1.122412
C	-3.722378	-2.643843	2.187804	H	-2.614408	-5.516971	-0.216912
C	-4.728166	-1.976856	1.524410	C	-2.495564	-5.680509	-2.364531
H	-3.368477	5.093659	-0.808625	H	-3.247042	-6.463224	-2.427001
H	-2.090318	3.373124	-2.054331	C	-0.933529	-4.186465	-3.446305
C	-4.519941	-0.714310	0.973706	H	-0.462804	-3.785113	-4.337969
C	-6.668948	-1.270509	0.667362	C	-1.891076	-5.193702	-3.527878
O	-5.674923	-0.288867	0.361856	H	-2.169651	-5.599621	-4.496770
O	-6.014065	-2.387204	1.281194	C	-0.550277	-3.662089	-2.200809
H	-3.879214	-3.623495	2.626087	H	1.066719	-2.983541	-0.096731
C	-5.366692	3.463397	2.429243	H	2.545227	-0.900196	-1.799006
H	-6.431950	3.342152	2.185036				
H	-5.238111	3.811675	3.457696				
O	-4.692966	2.212997	2.262356				
O	-4.775348	4.405887	1.529519				
H	-7.154760	-1.596991	-0.257033				
H	-7.401778	-0.846728	1.367501				

Int1							
P	0.379368	0.007353	-1.629190	H	6.282226	1.775857	-3.096273
P	1.357991	0.181746	1.817534	O	5.106760	0.510577	-1.957054
C	1.148924	1.436773	-2.529111	O	6.007838	2.491632	-1.173008
C	-0.772022	-0.597142	-2.952527	H	5.855723	-3.910547	0.555482
C	1.218679	1.132093	3.392676	H	6.450878	-3.300350	-1.036861
C	1.983728	-1.442298	2.471300	H	-1.479636	0.214490	-3.152291
Cu	-0.589152	0.550135	0.476959	H	-0.256331	-0.849501	-3.885695
B	-0.827282	2.563557	0.479349	H	-1.350373	-1.461241	-2.614297
O	-0.409620	3.406726	1.503541	H	0.428533	0.634756	3.965166
O	-1.370771	3.310223	-0.562554	H	2.145935	1.137906	3.976892
C	-0.477764	4.788341	1.047907	H	0.886259	2.150195	3.177734
C	-1.506268	4.698765	-0.139069	H	1.218925	-1.843347	3.142302
C	-0.910280	5.662891	2.224952	H	2.137600	-2.155072	1.658651
C	0.937874	5.181485	0.601360	H	2.921177	-1.313053	3.023248
C	-2.964467	4.893499	0.300861	H	0.373546	2.199199	-2.654509
C	-1.203035	5.601160	-1.335167	H	1.953561	1.869173	-1.931478
H	-0.147308	5.623944	3.009661	H	1.544283	1.141601	-3.507180
H	-1.024637	6.709712	1.918441	O	-5.032343	-2.152821	-4.073060
H	-1.852781	5.320877	2.658345	C	-5.217459	-1.621489	-2.981689
H	0.989841	6.230394	0.288909	O	-4.891242	0.445199	0.519096
H	1.626772	5.039806	1.440104	C	-5.723166	-0.233062	-2.863052

H	1.284002	4.552114	-0.223863	H	-5.905737	0.288872	-3.798759
H	-3.168377	5.932038	0.584754	C	-5.928092	0.343619	-1.669943
H	-3.625044	4.631656	-0.532145	H	-6.283615	1.370147	-1.595204
H	-3.215144	4.246946	1.147465	O	-1.439331	-1.186220	3.289081
H	-1.952612	5.438619	-2.116953	C	-5.707558	-0.355354	-0.347906
H	-1.238209	6.659305	-1.049313	C	-7.052975	-0.463382	0.400484
H	-0.220092	5.389912	-1.762789	H	-6.889448	-0.926830	1.378394
C	4.474352	2.723246	0.763662	H	-7.771030	-1.061882	-0.167910
C	3.393740	2.090586	1.403486	H	-7.463655	0.538658	0.559692
C	2.833098	0.887112	0.951987	C	-5.127205	-1.740827	-0.518186
C	3.358655	0.260905	-0.222835	H	-4.889632	-2.258516	0.408835
C	4.437200	0.900502	-0.818923	C	-4.925299	-2.323095	-1.709790
C	4.981326	2.093767	-0.351916	H	-4.529528	-3.331423	-1.801210
C	2.915351	-1.039420	-0.822441	C	-1.548990	-1.734484	2.181798
C	1.709360	-1.273806	-1.558836	C	-3.555936	0.771074	0.063642
C	1.478941	-2.535942	-2.126417	H	-3.365482	0.322181	-0.917540
H	0.567025	-2.711669	-2.684741	H	-3.508663	1.855949	-0.049959
C	2.386424	-3.604660	-2.019576	C	-2.548105	0.279785	1.075221
C	3.545278	-3.356930	-1.318120	C	-2.017301	-1.023366	0.991966
H	4.890527	3.654357	1.132603	C	-0.990664	-3.867322	0.857845
H	2.987784	2.565398	2.288584	H	-0.971610	-3.309114	-0.072406
C	3.794236	-2.111056	-0.748487	C	-0.719430	-5.236074	0.835804
C	5.576581	-3.422052	-0.383337	H	-0.509806	-5.728732	-0.110131
O	5.007227	-2.140391	-0.102031	C	-0.712505	-5.970015	2.023776
O	4.586195	-4.209738	-1.053893	H	-0.507608	-7.037320	2.004192
H	2.187134	-4.570125	-2.471499	C	-1.223090	-3.956080	3.260067
C	6.221562	1.398272	-2.071945	H	-1.412258	-3.433910	4.192287
H	7.143561	0.868864	-1.791888	C	-0.965640	-5.324706	3.237707
				H	-0.960758	-5.890202	4.166081
				C	-1.247386	-3.209530	2.070998
				H	-2.205590	-1.599510	0.091925
				H	-2.630629	0.709050	2.071147

TS2							
P	1.690936	0.301860	-1.993249	H	7.100868	-1.893449	2.078830
P	0.595645	-0.101775	1.418250	H	0.885130	0.385298	-4.259826
C	2.345693	2.020296	-2.234583	H	2.572475	-0.190253	-4.271885
C	1.609190	-0.262499	-3.754812	H	1.235555	-1.288156	-3.816455
C	-0.618152	0.389926	2.717453	H	-1.386275	-0.389007	2.699051
C	1.206783	-1.707904	2.111518	H	-0.177828	0.447628	3.719253
Cu	-0.208245	0.009344	-0.763597	H	-1.093250	1.338703	2.455652
B	-1.322964	1.697753	-1.083443	H	1.586025	-1.587771	3.132107
O	-1.791753	2.503437	-0.062975	H	0.353731	-2.393056	2.107775
O	-1.288984	2.380664	-2.290545	H	1.991625	-2.123866	1.476750
C	-1.896700	3.872234	-0.556298	H	1.572568	2.597206	-2.751931
C	-1.963465	3.662837	-2.118184	H	2.534382	2.488991	-1.266506
C	-3.135004	4.514157	0.068435	H	3.268908	2.024095	-2.823908
C	-0.633901	4.609578	-0.090149	O	-6.879799	0.916122	3.581008
C	-3.390833	3.490752	-2.656790	C	-6.433716	0.335401	2.594609
C	-1.224468	4.713572	-2.946392	O	-4.357468	-1.038553	-0.677250
H	-3.006406	4.580026	1.153658	C	-5.487899	-0.797342	2.707686
H	-3.285914	5.529817	-0.316157	H	-5.175287	-1.056686	3.715940
H	-4.037050	3.929776	-0.125719	C	-5.021177	-1.444655	1.629235
H	-0.654324	5.667673	-0.373387	H	-4.279795	-2.236219	1.712775
H	-0.569308	4.547948	1.000610	O	-1.892348	-2.275530	0.967961
H	0.269699	4.152650	-0.504084	C	-5.471855	-1.139628	0.218316
H	-3.954608	4.428911	-2.613220	C	-6.274761	-2.344790	-0.321016
H	-3.339229	3.169043	-3.701906	H	-6.563110	-2.155674	-1.360254
H	-3.943623	2.729539	-2.097869	H	-7.174196	-2.515891	0.277495

H	-1.292243	4.457276	-4.008864	H	-5.645268	-3.239502	-0.295717
H	-1.671537	5.705256	-2.810125	C	-6.336434	0.098996	0.141319
H	-0.166624	4.771916	-2.679304	H	-6.599956	0.416574	-0.866949
C	2.847470	3.211191	2.521802	C	-6.793643	0.753784	1.217657
C	1.855555	2.221887	2.397340	H	-7.442891	1.622154	1.138949
C	2.012284	1.071824	1.609628	C	-1.489175	-2.517175	-0.198143
C	3.228419	0.878937	0.878670	C	-3.359345	-0.070812	-0.322715
C	4.192537	1.865112	1.036256	H	-3.775241	0.938905	-0.423083
C	4.016530	2.999009	1.825138	H	-3.039400	-0.217703	0.708859
C	3.596186	-0.312457	0.045567	C	-2.193458	-0.282012	-1.282955
C	3.097710	-0.639003	-1.256838	C	-1.505466	-1.582242	-1.286332
C	3.603410	-1.758058	-1.933935	C	-0.164639	-4.260850	-1.573485
H	3.216998	-2.009658	-2.914287	H	0.130702	-3.500627	-2.289778
C	4.600726	-2.592583	-1.400375	C	0.278795	-5.571429	-1.753243
C	5.075799	-2.258353	-0.151751	H	0.900647	-5.817138	-2.610889
H	2.701797	4.090643	3.139523	C	-0.070386	-6.564915	-0.835669
H	0.932995	2.367226	2.946676	H	0.271744	-7.586793	-0.978920
C	4.587941	-1.150147	0.536651	C	-1.291021	-4.920454	0.451008
C	6.075373	-2.195839	1.846761	H	-1.893038	-4.642603	1.309675
O	5.248628	-1.033278	1.736209	C	-0.857946	-6.232749	0.269214
O	6.053324	-2.874031	0.585350	H	-1.133983	-6.998210	0.990553
H	4.977625	-3.451388	-1.944535	C	-0.961650	-3.914216	-0.470352
C	6.103325	3.027146	1.034335	H	-1.252578	-1.967040	-2.268179
H	6.866695	2.658666	1.734168	H	-2.455747	0.017585	-2.298521
H	6.553117	3.645363	0.252827				
O	5.428941	1.917969	0.434354				
O	5.131161	3.796985	1.750173				
H	5.675262	-2.858632	2.625894				

Int2							
P	-1.726988	0.170229	-1.926334	H	-7.755730	1.972123	1.421556
P	-1.395263	-0.275715	1.645899	H	-0.564345	0.486583	-4.015633
C	-2.434488	-1.393762	-2.640381	H	-2.187282	1.213899	-4.150459
C	-1.322173	1.078911	-3.491491	H	-0.877473	2.050890	-3.261475
C	-0.470624	-1.163373	2.976637	H	0.321953	-0.474494	3.285734
C	-2.015165	1.194818	2.587153	H	-1.090502	-1.417656	3.843794
Cu	-0.139664	0.156774	-0.287279	H	-0.000086	-2.065074	2.575064
B	2.522777	-1.632181	0.154734	H	-2.559079	0.904211	3.492673
O	1.306643	-2.198518	-0.199023	H	-1.126676	1.779408	2.847305
O	3.556579	-2.534750	0.028116	H	-2.661658	1.807528	1.954954
C	1.571277	-3.489679	-0.815708	H	-1.649103	-1.891646	-3.219601
C	2.993136	-3.846342	-0.233357	H	-2.749916	-2.067305	-1.841318
C	0.449886	-4.454325	-0.434801	H	-3.287621	-1.188241	-3.295809
C	1.577973	-3.260773	-2.333639	O	9.355174	1.011793	1.805166
C	2.935894	-4.580070	1.114217	C	8.352459	0.874987	1.109869
C	3.920327	-4.591367	-1.193144	O	4.628826	0.352238	-0.562495
H	-0.499537	-4.098360	-0.848073	C	7.526265	2.032290	0.690969
H	0.641983	-5.455279	-0.838551	H	7.832181	3.000513	1.078289
H	0.334650	-4.533797	0.648650	C	6.459247	1.885074	-0.106842
H	1.709678	-4.197263	-2.886131	H	5.849700	2.739456	-0.397389
H	0.620152	-2.819301	-2.627042	O	1.277631	1.892375	2.148688
H	2.371835	-2.566540	-2.625834	C	6.035465	0.560784	-0.702222
H	2.582671	-5.610888	1.003314	C	6.262549	0.606907	-2.230286
H	3.941505	-4.605524	1.545264	H	5.923202	-0.333431	-2.676625
H	2.281184	-4.062180	1.822449	H	7.319947	0.758146	-2.465109
H	4.888449	-4.758930	-0.709830	H	5.675938	1.424407	-2.661877
H	3.503989	-5.569234	-1.461870	C	6.814070	-0.602921	-0.128344
H	4.095692	-4.022594	-2.109129	H	6.449823	-1.585800	-0.421910
C	-4.000849	-3.513176	1.511624	C	7.884658	-0.459219	0.665232

C	-2.961294	-2.619865	1.829444	H	8.450492	-1.309423	1.037316
C	-2.879817	-1.321443	1.305934	C	1.357992	1.979674	0.900309
C	-3.884733	-0.864050	0.393090	C	4.133003	0.271088	0.783710
C	-4.904236	-1.762253	0.114541	H	4.734242	-0.450373	1.354813
C	-4.966718	-3.048291	0.646913	H	4.214595	1.251453	1.272431
C	-3.989280	0.512236	-0.193319	C	2.665552	-0.172280	0.720938
C	-3.195724	1.068399	-1.249089	C	1.772219	0.883658	0.048826
C	-3.464592	2.366252	-1.707786	C	1.039729	3.594669	-1.100991
H	-2.850322	2.794435	-2.490140	H	1.429553	2.849885	-1.787295
C	-4.500055	3.165331	-1.192871	C	0.656459	4.840796	-1.599614
C	-5.265090	2.607401	-0.193237	H	0.753191	5.049088	-2.662939
H	-4.043395	-4.512418	1.931270	C	0.161911	5.823159	-0.737276
H	-2.198385	-2.962941	2.518532	H	-0.133134	6.794787	-1.125977
C	-5.012472	1.322026	0.279873	C	0.448741	4.304008	1.123309
C	-6.687422	2.200156	1.482286	H	0.397274	4.078441	2.183238
O	-5.931210	1.007458	1.253420	C	0.062067	5.549384	0.628455
O	-6.343549	3.143418	0.461080	H	-0.311263	6.310281	1.309949
H	-4.686657	4.166111	-1.565991	C	0.935669	3.302950	0.269883
C	-6.818329	-2.706705	-0.550440	H	2.152697	1.179587	-0.928268
H	-7.697275	-2.418049	0.043246	H	2.335083	-0.222389	1.771842
H	-7.115326	-3.099977	-1.526217				
O	-5.970728	-1.569667	-0.733434				
O	-6.070346	-3.702015	0.156791				
H	-6.429841	2.615564	2.466025				

TS3							
P	-2.099198	-0.701323	-2.210485	H	-6.222573	2.625623	2.126180
P	-1.454027	-0.615894	1.365285	H	-7.695045	1.873650	1.397326
C	-2.784900	-2.409388	-2.459566	H	-1.194651	-0.873923	-4.441675
C	-1.900630	-0.176903	-3.977577	H	-2.841924	-0.197773	-4.538032
C	-0.408127	-1.418517	2.657381	H	-1.460864	0.822137	-4.036169
C	-1.979327	0.928833	2.242811	H	0.485076	-0.798694	2.785710
Cu	-0.423960	-0.267204	-0.722167	H	-0.929180	-1.515057	3.616881
B	3.086383	0.124604	1.353543	H	-0.072637	-2.405466	2.325151
O	2.873347	-0.440475	2.606241	H	-2.442998	0.705122	3.209731
O	4.183022	0.968190	1.355384	H	-1.076691	1.531170	2.387313
C	3.924962	0.001002	3.496813	H	-2.680796	1.495254	1.625325
C	4.529994	1.248252	2.734142	H	-2.046127	-2.988921	-3.023958
C	3.309464	0.315422	4.861015	H	-2.943942	-2.902444	-1.499232
C	4.906541	-1.174030	3.626765	H	-3.727204	-2.389044	-3.017309
C	3.871057	2.583508	3.113257	O	7.835938	-3.876425	-2.124395
C	6.051100	1.385008	2.829899	C	7.120216	-2.879418	-2.084752
H	2.884662	-0.598191	5.291496	O	4.189710	-0.066169	-1.547859
H	4.065981	0.695991	5.557636	C	7.171876	-1.919936	-0.957206
H	2.509501	1.054431	4.776693	H	7.853945	-2.171951	-0.149168
H	5.717283	-0.958251	4.331531	C	6.406779	-0.818815	-0.932007
H	4.361504	-2.052014	3.988686	H	6.417483	-0.131650	-0.088578
H	5.346798	-1.429632	2.657642	O	1.325947	1.696916	1.317374
H	4.121404	2.880101	4.138404	C	5.478957	-0.416122	-2.056736
H	4.239636	3.358794	2.433228	C	6.001042	0.894005	-2.686691
H	2.786512	2.533501	2.998015	H	5.322876	1.211796	-3.485385
H	6.377580	2.239772	2.228175	H	7.005570	0.759230	-3.098234
H	6.365544	1.562671	3.865218	H	6.020309	1.676282	-1.921911
H	6.567681	0.494942	2.461557	C	5.379083	-1.476938	-3.129215
C	-4.131099	-3.760469	1.883053	H	4.654477	-1.273771	-3.916808
C	-3.040535	-2.876395	1.973544	C	6.137192	-2.582057	-3.154450
C	-2.978213	-1.661373	1.275627	H	6.067279	-3.317151	-3.952108
C	-4.058949	-1.287755	0.414045	C	1.159472	1.669736	0.065105
C	-5.127180	-2.171195	0.362441	C	3.440327	-1.109509	-0.918301

C	-5.169955	-3.372631	1.066188	H	2.931469	-1.705248	-1.694277
C	-4.183665	-0.013081	-0.364567	H	4.113671	-1.786955	-0.374052
C	-3.504709	0.320425	-1.581207	C	2.413323	-0.493270	0.041035
C	-3.801801	1.525364	-2.234750	C	1.492824	0.502204	-0.695883
H	-3.271256	1.787303	-3.142134	C	0.347628	3.005527	-1.977141
C	-4.758348	2.439946	-1.761811	H	0.570503	2.163851	-2.625381
C	-5.408704	2.099097	-0.596913	C	-0.160350	4.180539	-2.530573
H	-4.154828	-4.694758	2.433226	H	-0.325073	4.244685	-3.603793
H	-2.222209	-3.159727	2.624532	C	-0.448845	5.276666	-1.712373
C	-5.127029	0.907910	0.068689	H	-0.843143	6.193016	-2.144576
C	-6.623236	2.054724	1.277329	C	0.293728	4.012753	0.212233
O	-5.936260	0.803654	1.175071	H	0.494072	3.931690	1.275214
O	-6.396044	2.781267	0.064302	C	-0.218018	5.187852	-0.338181
H	-4.971566	3.364032	-2.287228	H	-0.431003	6.038242	0.305189
C	-7.119513	-3.124426	0.006491	C	0.579930	2.902082	-0.594907
H	-7.941279	-2.733440	0.622424	H	1.764561	0.678829	-1.735802
H	-7.502626	-3.635726	-0.880702	H	1.839210	-1.360092	0.410657
O	-6.268158	-2.047246	-0.395256				
O	-6.334983	-4.038445	0.781406				

Int3							
P	-1.468290	-0.347227	-2.020342	O	-5.889541	-1.725984	-1.142755
P	-1.805329	0.400885	1.530123	O	-6.188173	-3.494410	0.320674
C	-2.089733	-2.062205	-2.371392	H	-7.293064	3.035242	0.262683
C	-0.705420	0.062182	-3.656967	H	-7.905917	1.896216	-0.998983
C	-1.230577	0.028012	3.245530	H	0.117490	-0.643449	-3.810842
C	-2.632973	2.040594	1.796161	H	-1.408166	-0.023903	-4.493006
Cu	-0.236628	0.156264	-0.143143	H	-0.272982	1.066218	-3.639790
B	2.469663	-0.848370	0.412282	H	-0.507673	0.806528	3.509361
O	1.172957	-1.577927	0.576230	H	-2.044016	0.028378	3.979459
O	3.497775	-1.792455	0.741642	H	-0.703000	-0.928261	3.267583
C	1.460852	-2.962368	0.810885	H	-3.403792	1.981185	2.572080
C	2.909427	-2.893955	1.439011	H	-1.865816	2.761413	2.095957
C	0.389795	-3.556074	1.728912	H	-3.085517	2.391606	0.866361
C	1.446178	-3.703970	-0.538295	H	-1.226987	-2.693250	-2.609024
C	2.885551	-2.573763	2.945705	H	-2.579065	-2.475334	-1.487231
C	3.771999	-4.135724	1.195214	H	-2.789983	-2.075469	-3.213519
H	-0.587832	-3.520391	1.233793	O	9.006544	-0.930675	-2.746640
H	0.607972	-4.604173	1.967991	C	8.089369	-0.390901	-2.132356
H	0.315150	-2.999184	2.666489	O	4.575647	1.114289	-0.593415
H	1.603288	-4.782188	-0.419039	C	7.389575	-1.060179	-1.013032
H	0.470968	-3.553421	-1.015404	H	7.695996	-2.081883	-0.803626
H	2.219104	-3.312273	-1.205858	C	6.411739	-0.454027	-0.322607
H	2.528843	-3.420930	3.543969	H	5.853015	-0.969866	0.455886
H	3.904926	-2.334130	3.266210	O	2.363015	0.343234	1.419370
H	2.257407	-1.702434	3.149585	C	5.998202	0.982281	-0.557844
H	4.752786	-3.998328	1.664179	C	6.418754	1.826060	0.667589
H	3.315375	-5.033747	1.629318	H	6.093923	2.862183	0.526131
H	3.933912	-4.304836	0.127778	H	7.503454	1.803168	0.806425
C	-4.384424	-2.838004	1.889074	H	5.925747	1.430136	1.560606
C	-3.400918	-1.854527	2.098851	C	6.631535	1.570821	-1.798472
C	-3.203539	-0.771276	1.230538	H	6.270827	2.560085	-2.077262
C	-4.019019	-0.646478	0.059596	C	7.598077	0.960078	-2.497851
C	-4.992020	-1.621520	-0.106311	H	8.064829	1.416292	-3.367192
C	-5.173798	-2.688457	0.770526	C	1.799761	1.341743	0.760549
C	-3.985738	0.472300	-0.938016	C	3.873263	0.334575	-1.568261
C	-2.998495	0.689275	-1.952546	H	3.796436	0.917594	-2.502373
C	-3.142641	1.763939	-2.841858	H	4.431569	-0.583974	-1.793177
H	-2.392016	1.930908	-3.605137	C	2.495893	-0.050868	-1.020598

C	-4.231979	2.652376	-2.803422	C	1.651071	1.152845	-0.613913
C	-5.183859	2.413995	-1.836992	C	0.809665	3.659212	0.978217
H	-4.516704	-3.667121	2.575364	H	0.512921	3.629565	-0.065877
H	-2.777841	-1.954472	2.979410	C	0.531098	4.791055	1.741459
C	-5.058225	1.353170	-0.942775	H	0.026280	5.638074	1.283728
C	-7.017115	2.369599	-0.559996	C	0.902255	4.841490	3.089634
O	-6.125893	1.359442	-0.077090	H	0.686661	5.726420	3.682634
O	-6.328474	3.118398	-1.568473	C	1.835668	2.615446	2.901327
H	-4.320854	3.476462	-3.502626	H	2.355592	1.767471	3.332939
C	-6.760691	-2.807945	-0.797896	C	1.556848	3.750598	3.664238
H	-7.746416	-2.411051	-0.518623	H	1.857190	3.784242	4.708448
H	-6.840301	-3.495102	-1.644882	C	1.463175	2.553060	1.548616
				H	1.447798	1.995685	-1.273041
				H	2.014295	-0.656650	-1.802586

TS4							
P	-1.477792	-0.318922	-1.901300	O	-6.562764	-3.369967	-0.278755
P	-1.910023	-0.177104	1.718258	H	-7.150825	3.105747	0.683159
C	-2.214159	-1.840462	-2.671965	H	-7.758402	2.251645	-0.788862
C	-0.520175	0.354759	-3.331447	H	0.244579	-0.388169	-3.579126
C	-1.602527	-0.736069	3.456408	H	-1.139745	0.536974	-4.216265
C	-2.500090	1.551039	2.029879	H	0.001883	1.268990	-3.038056
Cu	-0.358464	-0.590173	0.113671	H	-0.825627	-0.079756	3.861842
B	2.508747	-0.642153	0.182413	H	-2.494306	-0.669344	4.089409
O	1.354649	-1.629314	-0.043889	H	-1.211314	-1.756588	3.477478
O	3.543048	-1.455908	0.762257	H	-3.385883	1.556291	2.674134
C	1.780790	-2.952672	0.338327	H	-1.688285	2.101429	2.514603
C	2.982997	-2.645438	1.313414	H	-2.731235	2.053015	1.089466
C	0.617089	-3.715102	0.974132	H	-1.395317	-2.517755	-2.937461
C	2.230718	-3.674839	-0.943201	H	-2.861428	-2.351459	-1.956060
C	2.521933	-2.364872	2.757614	H	-2.790772	-1.600111	-3.571506
C	4.069029	-3.726622	1.333275	O	9.511979	-0.811224	-2.086457
H	-0.177918	-3.874958	0.235748	C	8.536144	-0.278891	-1.562538
H	0.942775	-4.699424	1.331781	O	4.878096	1.198509	-0.381218
H	0.192655	-3.166393	1.819189	C	7.721903	-0.965900	-0.534881
H	2.517852	-4.714619	-0.749945	H	8.005703	-1.991156	-0.310909
H	1.402981	-3.677929	-1.661612	C	6.677503	-0.368038	0.058075
H	3.078827	-3.158957	-1.402214	H	6.038748	-0.889755	0.767210
H	2.167837	-3.271951	3.262805	O	1.902209	0.415386	1.131162
H	3.374003	-1.970307	3.320670	C	6.287891	1.071065	-0.196431
H	1.732446	-1.609397	2.779173	C	6.572352	1.894927	1.080812
H	4.854691	-3.442031	2.041920	H	6.253951	2.930676	0.923869
H	3.666745	-4.696325	1.651368	H	7.636955	1.876825	1.331414
H	4.531961	-3.844114	0.350377	H	5.993395	1.480382	1.911700
C	-4.807842	-3.132108	1.454391	C	7.051996	1.680448	-1.350251
C	-3.754989	-2.291598	1.856600	H	6.724700	2.675655	-1.648490
C	-3.409071	-1.115317	1.175474	C	8.088505	1.080329	-1.952181
C	-4.139700	-0.735081	0.002780	H	8.646872	1.551624	-2.757081
C	-5.181249	-1.579348	-0.356985	C	1.666213	1.539450	0.396625
C	-5.509446	-2.743009	0.334779	C	4.305190	0.475302	-1.475521
C	-3.968909	0.523359	-0.794523	H	4.443714	1.055164	-2.404107
C	-2.911893	0.827692	-1.710860	H	4.818178	-0.487548	-1.603780
C	-2.921610	2.048275	-2.401084	C	2.823274	0.220172	-1.181298
H	-2.115189	2.281155	-3.085873	C	2.055418	1.489747	-0.903040
C	-3.941842	3.004736	-2.255021	C	0.480728	3.765217	0.463720
C	-4.965404	2.682602	-1.391531	H	0.419940	3.760395	-0.620935
H	-5.057311	-4.035069	2.000553	C	-0.039825	4.841556	1.178731
H	-3.197873	-2.580390	2.740154	H	-0.497756	5.670675	0.644787
C	-4.971786	1.477221	-0.693312	C	0.021639	4.858234	2.576050

C	-6.869230	2.571309	-0.228295	H	-0.383571	5.699447	3.132358
O	-6.082642	1.425844	0.114541	C	1.130263	2.703693	2.533097
O	-6.066989	3.424328	-1.052753	H	1.602615	1.872928	3.046103
H	-3.925963	3.943096	-2.798206	C	0.611701	3.785090	3.247819
C	-7.003970	-2.472362	-1.303966	H	0.675267	3.792211	4.333527
H	-7.966671	-2.029993	-1.013533	C	1.076690	2.677802	1.127959
H	-7.090486	-3.014352	-2.249828	H	2.015498	2.347934	-1.569505
O	-6.022629	-1.438862	-1.435086	H	2.432901	-0.316340	-2.062542

Int4							
P	1.499755	0.082611	1.708574	H	7.503583	2.428640	-1.314890
P	1.968960	-0.595068	-1.820008	H	7.999142	1.833798	0.318035
C	2.064407	-1.380313	2.701828	H	-0.180660	0.435032	3.391334
C	0.636395	1.060921	3.018619	H	1.292801	1.328455	3.853929
C	1.688641	-1.473715	-3.429768	H	0.181054	1.955962	2.588672
C	2.692922	0.989982	-2.454179	H	0.992741	-0.860483	-4.011460
Cu	0.352267	-0.394928	-0.243518	H	2.613703	-1.603267	-4.002182
B	-2.411151	-0.431384	0.043213	H	1.213695	-2.445972	-3.275645
O	-1.391325	-1.504476	0.361945	H	3.566588	0.806360	-3.088739
O	-3.363032	-1.094155	-0.820052	H	1.920420	1.505924	-3.032874
C	-1.902663	-2.773534	-0.085973	H	2.974826	1.640076	-1.625007
C	-2.859757	-2.350195	-1.264104	H	1.171726	-1.912453	3.047107
C	-0.740158	-3.682518	-0.486706	H	2.647640	-2.061648	2.078774
C	-2.662607	-3.408840	1.091691	H	2.664102	-1.077835	3.567008
C	-2.118593	-2.149434	-2.600497	O	-9.612239	-1.470186	1.452584
C	-4.039332	-3.303617	-1.488916	C	-8.667683	-0.787372	1.063586
H	-0.127253	-3.919007	0.390861	O	-5.114883	1.157494	0.348527
H	-1.106216	-4.627846	-0.905133	C	-7.714409	-1.273399	0.040504
H	-0.095899	-3.202758	-1.228764	H	-7.871394	-2.290215	-0.310771
H	-3.028565	-4.413175	0.850337	C	-6.703166	-0.511651	-0.402658
H	-1.986766	-3.488288	1.950321	H	-5.968760	-0.882439	-1.113222
H	-3.516297	-2.792317	1.387375	O	-1.533905	0.653278	-0.652812
H	-1.781345	-3.098137	-3.036715	C	-6.485801	0.920848	0.029468
H	-2.804943	-1.672099	-3.307708	C	-6.752566	1.847820	-1.178935
H	-1.256795	-1.488013	-2.476793	H	-6.559440	2.886356	-0.891056
H	-4.643616	-2.950063	-2.332146	H	-7.785595	1.754203	-1.526274
H	-3.695714	-4.317802	-1.727748	H	-6.068171	1.584303	-1.991097
H	-4.686339	-3.351808	-0.609645	C	-7.397219	1.325489	1.166210
C	4.609431	-3.642109	-0.864522	H	-7.205123	2.312140	1.585889
C	3.630085	-2.824350	-1.454738	C	-8.402917	0.562798	1.617487
C	3.386444	-1.502617	-1.051493	H	-9.068316	0.888165	2.413150
C	4.151225	-0.939097	0.021596	C	-1.704925	1.852513	0.005078
C	5.120884	-1.768353	0.568947	C	-4.569285	0.407724	1.437742
C	5.347555	-3.077571	0.151878	H	-4.896814	0.863081	2.387893
C	4.081122	0.464527	0.542928	H	-4.945998	-0.623983	1.417610
C	3.044049	1.030030	1.352014	C	-3.041594	0.407378	1.317529
C	3.151844	2.358157	1.789040	C	-2.483333	1.791930	1.102553
H	2.358412	2.788276	2.387952	C	-0.805653	4.196329	0.138937
C	4.252321	3.179048	1.486579	H	-1.069777	4.227140	1.192565
C	5.253147	2.609516	0.730983	C	-0.221669	5.310487	-0.459542
H	4.777730	-4.660534	-1.196656	H	-0.038908	6.204426	0.131846
H	3.046777	-3.252276	-2.261160	C	0.132617	5.282067	-1.811836
C	5.163079	1.293956	0.282744	H	0.588735	6.151799	-2.277391
C	7.154948	2.111905	-0.328164	C	-0.690544	3.008235	-1.959909
O	6.273528	0.993572	-0.470860	H	-0.897048	2.114429	-2.539192
O	6.417902	3.176960	0.281637	C	-0.107089	4.126046	-2.557475
H	4.310287	4.204987	1.832687	H	0.153581	4.097316	-3.613088
C	6.866266	-2.581046	1.712869	C	-1.055886	3.026299	-0.601740
H	7.860676	-2.293309	1.345065	H	-2.766414	2.666099	1.682636

H	6.912916	-2.909087	2.754998	H	-2.670752	-0.022418	2.265948
O	5.974709	-1.467273	1.603442				
O	6.346476	-3.641299	0.902201				

Int5

P	2.565546	-0.054727	-1.976929	C	-3.353455	-0.573552	1.650976
P	1.986609	0.923188	1.433721	H	-2.851619	-1.327789	2.286298
C	4.296371	0.450879	-2.435864	C	-2.337642	0.373736	1.042087
C	1.967334	-0.609801	-3.641748	H	-2.133662	1.252140	1.655776
C	1.753551	2.330656	2.615821	C	-1.607052	-2.110660	-0.699886
C	1.258434	-0.465036	2.432928	H	-1.485252	-2.336411	0.355624
Cu	1.332941	1.416907	-0.770295	C	-1.624795	-3.152480	-1.632748
C	6.077733	1.408216	2.003184	H	-1.526784	-4.180824	-1.292694
C	4.677787	1.537202	2.034976	C	-1.755982	-2.874922	-2.995246
C	3.807103	0.597849	1.463132	H	-1.777354	-3.684068	-3.721224
C	4.346790	-0.547397	0.793571	C	-1.802712	-0.506169	-2.487575
C	5.730290	-0.655572	0.799772	H	-1.843681	0.529046	-2.811891
C	6.576385	0.287250	1.377197	C	-1.846320	-1.543089	-3.418450
C	3.568028	-1.669667	0.177063	H	-1.945620	-1.315537	-4.477504
C	2.819383	-1.634604	-1.043804	C	-1.705341	-0.772664	-1.110890
C	2.181636	-2.799003	-1.496134	C	-5.797309	-3.115808	0.211929
H	1.595077	-2.766936	-2.405663	C	-6.035597	-1.879381	-0.753042
C	2.251670	-4.028337	-0.818425	B	-4.358274	-1.398807	0.738585
C	2.997174	-4.049429	0.339125	O	-4.901274	-2.569410	1.214908
H	6.729344	2.150306	2.451443	O	-4.897163	-1.019406	-0.466984
H	4.268562	2.409629	2.529948	C	-7.048427	-3.629701	0.927765
C	3.631999	-2.903907	0.809971	H	-7.779497	-4.022496	0.211748
C	4.177993	-4.612651	2.147868	H	-6.769248	-4.442865	1.605735
O	4.274839	-3.195155	1.992789	H	-7.526612	-2.847049	1.521151
O	3.219955	-5.095872	1.200293	C	-5.064408	-4.289123	-0.450632
H	1.745910	-4.912675	-1.190076	H	-4.793505	-5.016568	0.321703
C	7.834024	-1.396295	0.624911	H	-5.696862	-4.793066	-1.189534
H	8.133564	-2.131043	1.386082	H	-4.146179	-3.958885	-0.942201
H	8.491598	-1.446237	-0.247392	C	-6.020016	-2.209242	-2.245785
O	6.486583	-1.649674	0.220169	H	-6.833418	-2.897438	-2.503987
O	7.885921	-0.080756	1.185916	H	-6.159181	-1.289830	-2.824028
H	3.837459	-4.845746	3.160667	H	-5.069904	-2.653382	-2.550166
H	5.156011	-5.072795	1.946301	C	-7.292940	-1.068700	-0.406937
H	1.973743	0.274718	-4.287808	H	-7.321028	-0.170781	-1.033705
H	2.605576	-1.381007	-4.087936	H	-8.208129	-1.640004	-0.595155
H	0.936620	-0.967901	-3.583491	H	-7.281476	-0.748650	0.639013
H	0.673393	2.441351	2.760266	O	1.050157	3.403637	-1.026967
H	2.226049	2.169288	3.591616	Li	-0.656287	3.239689	-0.518786
H	2.118380	3.260244	2.170592	C	1.852677	4.324785	-1.711145
H	1.689671	-0.522878	3.438744	C	1.246602	5.734704	-1.541337
H	0.179345	-0.289864	2.498073	H	1.840202	6.505888	-2.049835
H	1.411946	-1.416707	1.919705	H	0.229516	5.761456	-1.951989
H	4.231949	1.282076	-3.145840	H	1.186039	5.990271	-0.476976
H	4.839542	0.799219	-1.555317	C	3.283514	4.321018	-1.132520
H	4.847620	-0.376040	-2.897120	H	3.944287	5.029804	-1.649357
O	-1.948232	4.476002	0.314405	H	3.256111	4.590177	-0.069651
C	-3.003752	3.896274	0.637958	H	3.720168	3.318558	-1.213688
O	-5.374312	0.900699	2.137125	C	1.902258	3.975201	-3.214825
C	-3.509942	3.982890	2.020921	H	0.888233	3.979835	-3.632811
H	-3.005612	4.686296	2.677990	H	2.517006	4.681129	-3.789119
C	-4.472058	3.154936	2.457723	H	2.313708	2.967725	-3.351850
H	-4.801033	3.176057	3.495457				
O	-0.916442	1.398607	-0.530536				
C	-5.213657	2.200458	1.554078				

C -6.672797 2.717631 1.427638				
H -7.247280 2.030184 0.799759				
H -6.697857 3.716958 0.984476				
H -7.134255 2.745900 2.419701				
C -4.635157 2.153072 0.160435				
H -5.051158 1.388686 -0.488153				
C -3.716069 3.026746 -0.290176				
H -3.342613 2.982604 -1.308979				
C -1.652271 0.368602 -0.141251				
C -4.244788 0.189303 2.673140				
H -4.709473 -0.531922 3.352342				
H -3.623474 0.870913 3.271571				

Int5’							
P	-2.262391	-1.231506	1.519455	C	2.784541	4.318375	-0.889605
P	-2.211511	0.781547	-1.487896	H	3.365398	4.887204	-1.614170
C	-2.992936	-0.393550	3.011352	O	0.838773	-1.205893	0.187825
C	-1.510083	-2.726927	2.305690	C	3.579971	3.363809	-0.030094
C	-1.646967	2.238276	-2.478615	C	4.477979	4.210531	0.908085
C	-2.969301	-0.245532	-2.836689	H	5.083613	3.545831	1.530396
Cu	-0.799747	-0.309839	-0.145510	H	3.871283	4.853595	1.551691
C	-4.759783	3.378438	0.493995	H	5.150854	4.829709	0.305898
C	-3.740874	2.800833	-0.282689	C	2.699624	2.491850	0.836658
C	-3.645935	1.420151	-0.511312	H	3.210971	1.685022	1.354526
C	-4.595125	0.534113	0.092062	C	1.384601	2.715343	1.008007
C	-5.601868	1.140797	0.830037	H	0.781931	2.094185	1.664702
C	-5.690272	2.515569	1.030039	C	2.043675	-1.110108	-0.367652
C	-4.634257	-0.957513	-0.031425	C	4.043681	1.805597	-1.907570
C	-3.733174	-1.872834	0.601014	H	4.817238	1.922933	-2.673630
C	-3.909989	-3.252719	0.426468	H	3.106364	2.219721	-2.304289
H	-3.218593	-3.942799	0.895374	C	3.879364	0.297487	-1.583249
C	-4.957935	-3.800109	-0.335549	H	4.028047	-0.187600	-2.565039
C	-5.831908	-2.904250	-0.911564	C	2.502067	-0.007162	-1.023280
H	-4.806154	4.448359	0.663422	H	1.765084	0.786152	-1.138526
H	-2.989854	3.464773	-0.690432	C	3.585119	-2.936310	-1.225797
C	-5.669790	-1.529607	-0.754929	H	3.594843	-2.453875	-2.198446
C	-7.549643	-1.888121	-1.914079	C	4.266170	-4.144628	-1.047922
O	-6.650645	-0.872179	-1.460430	H	4.813466	-4.581571	-1.880006
O	-6.918576	-3.157274	-1.710380	C	4.234814	-4.793977	0.187020
H	-5.074041	-4.871228	-0.459159	H	4.763501	-5.733711	0.326862
C	-7.464828	1.563094	1.996386	C	2.811739	-3.035993	1.053263
H	-8.396450	1.586617	1.413553	H	2.224344	-2.607116	1.858658
H	-7.671666	1.400861	3.058032	C	3.503317	-4.230943	1.239318
O	-6.622116	0.514661	1.509945	H	3.466021	-4.733243	2.203572
O	-6.767347	2.802203	1.830855	C	2.853219	-2.358560	-0.177660
H	-7.749234	-1.747984	-2.980032	C	7.293831	-0.714152	-0.207477
H	-8.479584	-1.843966	-1.329992	C	6.527868	-0.292092	1.117467
H	-0.723146	-2.364887	2.975022	B	5.131519	-0.103787	-0.692073
H	-2.224585	-3.324450	2.882870	O	6.369193	-0.315879	-1.253378
H	-1.021793	-3.344290	1.547487	O	5.148062	-0.183705	0.678443
H	-0.883620	1.874137	-3.174162	C	8.618938	0.012729	-0.448266
H	-2.465800	2.691378	-3.048684	H	9.346698	-0.222179	0.337266
H	-1.189039	2.987792	-1.830323	H	9.039768	-0.310324	-1.406102
H	-3.787002	0.287650	-3.333346	H	8.485188	1.096124	-0.488740
H	-2.184347	-0.466512	-3.567918	C	7.505114	-2.227052	-0.349196
H	-3.341903	-1.191937	-2.441743	H	7.890628	-2.435810	-1.352744
H	-2.170896	-0.112377	3.678602	H	8.231233	-2.601574	0.380843
H	-3.516346	0.517987	2.714817	H	6.569371	-2.778365	-0.229098
H	-3.685283	-1.049438	3.550362	C	6.585811	-1.315750	2.252463

O	-0.557857	3.887865	0.307072	H	7.617395	-1.473114	2.588983
C	0.664191	3.726756	0.219719	H	6.004735	-0.946750	3.104215
O	4.515474	2.615031	-0.811738	H	6.161121	-2.275266	1.949922
C	1.466681	4.515190	-0.745188	C	6.929791	1.093840	1.641526
H	0.920130	5.242472	-1.340418	H	6.262545	1.365823	2.466480
				H	7.958240	1.104334	2.018541
				H	6.828621	1.853397	0.861390

Int5''							
P	-1.916579	0.366415	-1.645477	H	-6.132915	1.421142	1.666618
P	-3.672845	1.375244	1.411365	H	-5.466308	2.994371	1.148290
C	-1.074128	-1.257613	-1.354718	H	-5.644892	1.682013	-0.029641
C	-1.167617	0.833793	-3.268764	H	0.000047	-1.046220	-1.357374
C	-3.562484	1.864897	3.190057	H	-1.354261	-1.677374	-0.386599
C	-5.404592	1.909441	1.010528	H	-1.305562	-1.978569	-2.145879
Cu	-2.034335	2.035483	0.001450	O	-0.744069	0.413024	4.567609
B	5.792139	0.284781	0.158799	C	0.224506	-0.086967	3.986786
O	6.201079	0.702626	-1.082366	O	3.718114	-1.201169	2.087524
O	6.729969	-0.500316	0.790708	C	1.370501	-0.643640	4.742255
C	7.593884	0.336837	-1.255683	H	1.337886	-0.517513	5.821303
C	7.813267	-0.768118	-0.139585	C	2.387099	-1.263101	4.123222
C	7.784575	-0.145797	-2.694519	H	3.229711	-1.663161	4.684962
C	8.410131	1.617043	-1.023424	O	1.605816	1.226042	-1.545895
C	7.625953	-2.204381	-0.644846	C	2.431231	-1.508872	2.631151
C	9.132327	-0.663408	0.629223	C	2.267083	-3.026851	2.380018
H	7.585132	0.681489	-3.383416	H	2.305383	-3.225428	1.304566
H	8.812570	-0.487818	-2.863522	H	1.314559	-3.388049	2.778347
H	7.095543	-0.956629	-2.940301	H	3.089584	-3.565945	2.860824
H	9.481577	1.452512	-1.181836	C	1.328672	-0.779256	1.898243
H	8.070795	2.383717	-1.726947	H	1.388311	-0.811731	0.815780
H	8.265163	2.005318	-0.010197	C	0.313634	-0.155157	2.518464
H	8.443085	-2.505791	-1.309630	H	-0.480200	0.332389	1.958471
H	7.614022	-2.882307	0.214971	C	2.707823	0.700025	-1.027820
H	6.679990	-2.318717	-1.179551	C	4.204342	0.144097	2.248607
H	9.164946	-1.439028	1.401477	H	3.465859	0.736546	2.802883
H	9.989311	-0.813866	-0.037564	H	5.116912	0.080608	2.854807
H	9.240476	0.305473	1.122515	C	4.513348	0.841996	0.905417
C	-3.320371	-2.547508	2.706111	C	3.268906	1.191420	0.119235
C	-3.362190	-1.145025	2.613949	C	3.708440	-1.646292	-1.219203
C	-3.741996	-0.470692	1.443971	H	3.754542	-1.718919	-0.137322
C	-4.081361	-1.217133	0.270075	C	4.103156	-2.735025	-2.004693
C	-4.052907	-2.597509	0.405120	H	4.444889	-3.647855	-1.521392
C	-3.683260	-3.250360	1.578574	C	4.050277	-2.660331	-3.397185
C	-4.520642	-0.662608	-1.050477	H	4.356680	-3.507634	-4.005976
C	-3.681601	-0.035448	-2.026855	C	3.172088	-0.411580	-3.218362
C	-4.232571	0.381996	-3.247204	H	2.790239	0.491888	-3.682736
H	-3.596412	0.860577	-3.982362	C	3.586303	-1.486712	-4.001062
C	-5.587395	0.201782	-3.579821	H	3.540110	-1.415246	-5.085542
C	-6.372694	-0.425617	-2.638114	C	3.237455	-0.465195	-1.814671
H	-3.018891	-3.046957	3.620117	H	2.706946	2.021854	0.558538
H	-3.063296	-0.580860	3.488803	H	4.926941	1.816104	1.244120
C	-5.845627	-0.844669	-1.418555	C	-0.872044	4.795919	0.221243
C	-7.981639	-1.502022	-1.525289	C	-0.756317	4.770281	1.756395
O	-6.845403	-1.408668	-0.660948	H	-1.554767	4.153586	2.185377
O	-7.712759	-0.714399	-2.690675	H	0.202637	4.333105	2.058008
H	-5.989768	0.532674	-4.530950	H	-0.827972	5.775965	2.189440
C	-4.261762	-4.804800	0.078003	C	0.277772	5.634947	-0.370803
H	-5.269878	-5.233406	0.158193	H	1.249612	5.212077	-0.082914
H	-3.600476	-5.463951	-0.492264	H	0.215609	5.643191	-1.465909
O	-4.333878	-3.528909	-0.566168	H	0.251614	6.673891	-0.020019
O	-3.722106	-4.607319	1.389971	C	-2.220495	5.410826	-0.197278
H	-8.862168	-1.105044	-1.012988	H	-2.306997	5.421780	-1.289989
H	-8.135084	-2.549822	-1.819003	H	-3.047377	4.809458	0.201506
H	-0.087307	0.889707	-3.086652	H	-2.335536	6.438328	0.170479
H	-1.366299	0.092896	-4.051138	O	-0.765748	3.480612	-0.296541

H	-1.520112	1.816869	-3.594668	Li	0.634232	2.571585	-0.942576
H	-3.642868	2.956379	3.230448				
H	-4.372419	1.428992	3.785739				
H	-2.597861	1.576519	3.618999				

TS-CC							
P	-2.692298	-0.172126	2.090425	H	4.595798	-1.465678	-3.237426
P	-2.367601	1.114733	-1.289888	H	3.170126	-0.449879	-3.551474
C	-4.470441	-0.048048	2.612944	C	3.398557	-1.021796	-1.460334
C	-1.916404	-0.678296	3.693429	H	2.854716	-1.967131	-1.641001
C	-2.437115	2.640664	-2.333764	C	2.445385	0.094272	-1.078332
C	-1.423921	-0.026860	-2.407720	H	1.931217	0.520774	-1.938048
Cu	-1.811879	1.554422	0.962117	C	2.092988	-1.828570	1.422927
C	-6.491967	0.831503	-1.747422	H	2.048718	-2.398344	0.500021
C	-5.145025	1.233946	-1.796612	C	2.237064	-2.503378	2.639018
C	-4.093054	0.450309	-1.301377	H	2.319067	-3.587795	2.647155
C	-4.379132	-0.815989	-0.696177	C	2.272629	-1.789731	3.839068
C	-5.714182	-1.192960	-0.684548	H	2.395093	-2.312493	4.784285
C	-6.744277	-0.400552	-1.185758	C	1.966134	0.271112	2.604073
C	-3.374340	-1.797324	-0.171001	H	1.828309	1.347448	2.582934
C	-2.626881	-1.698307	1.047222	C	2.138108	-0.396861	3.815467
C	-1.746264	-2.728983	1.406148	H	2.160979	0.167336	4.744710
H	-1.159441	-2.640991	2.312077	C	1.963173	-0.431605	1.387085
C	-1.566904	-3.889413	0.632723	C	6.363748	-2.616274	0.313523
C	-2.314592	-3.980730	-0.520016	C	6.473216	-1.119005	0.811564
H	-7.288487	1.455986	-2.136938	B	4.626649	-1.369145	-0.518778
H	-4.925955	2.194710	-2.247015	O	5.335590	-2.532309	-0.711013
C	-3.188239	-2.964583	-0.898178	O	5.169996	-0.574416	0.459523
C	-3.359915	-4.631482	-2.386116	C	7.631474	-3.181109	-0.327698
O	-3.781531	-3.296300	-2.093976	H	8.454046	-3.217824	0.395968
O	-2.329338	-4.983150	-1.455737	H	7.441348	-4.201904	-0.675233
H	-0.877509	-4.671437	0.931032	H	7.948726	-2.586760	-1.187540
C	-7.628465	-2.339513	-0.520497	C	5.844037	-3.584568	1.384502
H	-7.803409	-3.072414	-1.320811	H	5.619705	-4.546334	0.911665
H	-8.236533	-2.567133	0.359263	H	6.587447	-3.753989	2.171001
O	-6.244708	-2.348535	-0.158167	H	4.926166	-3.210723	1.847086
O	-7.950202	-1.028213	-0.995843	C	6.674695	-0.947406	2.316627
H	-2.960779	-4.672968	-3.403917	H	7.621561	-1.396765	2.638153
H	-4.208547	-5.319276	-2.268905	H	6.706250	0.119114	2.561955
H	-2.093027	0.138241	4.401634	H	5.859173	-1.397520	2.886946
H	-2.347386	-1.600604	4.098607	C	7.511977	-0.292066	0.040325
H	-0.834436	-0.789646	3.584857	H	7.420235	0.757683	0.337439
H	-1.411166	3.015164	-2.428376	H	8.534034	-0.620047	0.257940
H	-2.852087	2.465910	-3.333035	H	7.342362	-0.349133	-1.039179
H	-3.011347	3.418867	-1.823284	O	-1.091152	3.354043	1.129408
H	-1.910228	-0.137732	-3.382990	Li	0.323080	3.100882	0.008072
H	-0.423634	0.396076	-2.540089	C	-1.516388	4.349730	2.028026
H	-1.314822	-1.008283	-1.941699	C	-0.669189	5.615456	1.785618
H	-4.545901	0.732149	3.378067	H	-0.969168	6.443454	2.440544
H	-5.097144	0.242225	1.767465	H	0.391181	5.403128	1.972323
H	-4.834966	-0.994961	3.025599	H	-0.769005	5.942031	0.744328
O	0.966311	4.075975	-1.533067	C	-3.004196	4.677117	1.784915
C	2.030776	3.434002	-1.798531	H	-3.373187	5.461240	2.459108
O	4.877194	0.466111	-2.793700	H	-3.148633	5.013948	0.751592
C	2.318297	3.049276	-3.201113	H	-3.615617	3.777901	1.937236
H	1.637965	3.436998	-3.956006	C	-1.323189	3.876393	3.483532
C	3.337668	2.233865	-3.517830	H	-0.271471	3.619919	3.656933
H	3.540608	1.951673	-4.549860	H	-1.618151	4.644362	4.210599
O	0.867618	1.273009	0.168979	H	-1.924827	2.977600	3.669894
C	4.338285	1.761671	-2.484876				
C	5.587322	2.677483	-2.582151				
H	6.336398	2.349521	-1.854194				
H	5.319779	3.717040	-2.381208				
H	6.021923	2.595334	-3.583771				
C	3.775609	1.891778	-1.076059				

H	4.439079	1.525001	-0.300070	
C	2.925098	2.963062	-0.799815	
H	2.736046	3.255614	0.230208	
C	1.721931	0.320400	0.110383	
C	3.989425	-0.645410	-2.841127	

TS-CC’

P	2.225217	0.451172	1.781970	H	-0.658548	-4.642359	-0.662065
P	2.408026	-1.142793	-1.503674	C	-2.672918	-3.980002	-0.503615
C	3.354951	-0.337930	3.028307	H	-3.088581	-4.726905	-1.178389
C	1.251351	1.582383	2.875752	O	-0.766996	0.371229	0.071862
C	2.035125	-2.697824	-2.434283	C	-3.669824	-3.064680	0.170231
C	2.709618	0.037878	-2.906893	C	-4.370697	-3.872560	1.293619
Cu	0.997978	-0.751035	0.257521	H	-5.114754	-3.240111	1.788428
C	5.886362	-3.029016	-0.226606	H	-3.640963	-4.215335	2.030652
C	4.650686	-2.714766	-0.819850	H	-4.886463	-4.734142	0.856817
C	4.092043	-1.429186	-0.791766	C	-2.982981	-1.872873	0.825732
C	4.784966	-0.373485	-0.116828	H	-3.670310	-1.188197	1.311764
C	6.011008	-0.711513	0.437657	C	-1.694248	-2.037270	1.334084
C	6.550511	-1.994292	0.393766	H	-1.270177	-1.284515	1.990309
C	4.355436	1.059702	-0.024065	C	-1.963717	0.498516	-0.363197
C	3.325618	1.586788	0.820000	C	-4.451876	-2.000587	-1.893510
C	3.076394	2.966642	0.826487	H	-5.372608	-2.038282	-2.484033
H	2.285602	3.363402	1.451252	H	-3.657887	-2.512146	-2.460441
C	3.807219	3.883621	0.050162	C	-4.041538	-0.532386	-1.623062
C	4.811440	3.360894	-0.733912	H	-4.005881	-0.063404	-2.623691
H	6.295627	-4.032740	-0.259420	C	-2.671511	-0.557912	-0.972183
H	4.117765	-3.517131	-1.315033	H	-2.012349	-1.324957	-1.376638
C	5.071252	1.993010	-0.759651	C	-3.183240	2.594872	-1.108112
C	6.598182	3.023612	-2.029926	H	-3.243177	2.186110	-2.112759
O	6.097301	1.741567	-1.642025	C	-3.667839	3.881678	-0.854642
O	5.662343	4.013885	-1.590690	H	-4.107642	4.461196	-1.662770
H	3.592656	4.946419	0.071446	C	-3.580459	4.424205	0.428589
C	8.033443	-0.658075	1.400203	H	-3.960756	5.422913	0.627959
H	8.865240	-0.288377	0.784616	C	-2.481313	2.407299	1.191432
H	8.278196	-0.595694	2.464766	H	-2.005532	1.831172	1.979207
O	6.860411	0.116382	1.135272	C	-2.987240	3.678586	1.453400
O	7.752725	-2.019031	1.055149	H	-2.913643	4.095329	2.455230
H	6.687589	3.065165	-3.119028	C	-2.587279	1.840017	-0.088311
H	7.570472	3.199836	-1.548289	C	-7.360258	1.006664	-0.569619
H	0.703559	0.948331	3.581145	C	-6.744327	0.600785	0.832027
H	1.872439	2.285259	3.442201	B	-5.260446	0.119427	-0.845835
H	0.513256	2.124214	2.278910	O	-6.431884	0.402457	-1.509827
H	1.120347	-2.513667	-3.007648	O	-5.350426	0.345912	0.505205
H	2.830184	-3.000833	-3.124686	C	-8.754857	0.445580	-0.850138
H	1.820316	-3.499178	-1.722571	H	-9.487566	0.839789	-0.136371
H	3.496712	-0.316805	-3.581244	H	-9.071032	0.740427	-1.856240
H	1.773849	0.145203	-3.466171	H	-8.769857	-0.645500	-0.799040
H	2.983845	1.020533	-2.517244	C	-7.342257	2.517231	-0.837588
H	2.737008	-0.922206	3.718695	H	-7.618518	2.693266	-1.882453
H	4.043327	-1.024975	2.531651	H	-8.056809	3.048691	-0.199598
H	3.927909	0.405200	3.593740	H	-6.346320	2.938051	-0.674555
O	0.477397	-2.805299	0.748782	C	-6.794156	1.690935	1.902405
C	-0.789880	-2.913107	0.680576	H	-7.830389	1.953729	2.145852
O	-4.756421	-2.725933	-0.706089	H	-6.314275	1.326257	2.816505
C	-1.363086	-3.945696	-0.212928	H	-6.265933	2.592494	1.584539
				C	-7.306374	-0.714400	1.390562
				H	-6.730534	-0.996145	2.278290
				H	-8.357224	-0.615497	1.683252

				H	-7.213918	-1.526079	0.662818

TS-CC’’

P	1.885903	0.303317	1.187279	H	5.774430	0.999444	-0.931761
P	3.688631	0.266659	-2.005637	H	0.095901	-1.286134	1.655913
C	1.187381	-1.334857	1.732333	H	1.540342	-2.136159	1.079371
C	1.133439	1.420314	2.455812	H	1.461236	-1.565128	2.767610
C	3.429050	0.100900	-3.829828	O	0.363266	0.801092	-2.433839
C	5.445625	0.871179	-1.965183	C	-0.818671	0.358890	-2.426441
Cu	1.961991	1.282218	-0.938108	O	-4.938430	-0.628478	-2.548414
B	-6.013205	-0.176381	0.017755	C	-1.654172	0.549909	-3.633757
O	-7.386704	-0.264274	0.068700	H	-1.198409	1.106799	-4.448739
O	-5.402733	-1.308391	0.512658	C	-2.909081	0.079562	-3.707587
C	-7.738892	-1.645395	0.347615	H	-3.516753	0.220019	-4.599845
C	-6.431796	-2.192533	1.045794	O	-1.956459	2.392343	0.295274
C	-8.996858	-1.658333	1.215041	C	-3.513855	-0.777641	-2.620441
C	-8.027107	-2.305430	-1.009482	C	-3.359737	-2.265238	-3.048796
C	-6.439841	-2.024954	2.570346	H	-3.806719	-2.907637	-2.283318
C	-6.054355	-3.628791	0.683679	H	-2.306580	-2.527988	-3.171797
H	-9.829700	-1.221822	0.654424	H	-3.892481	-2.427649	-3.991303
H	-9.274282	-2.682619	1.490034	C	-2.765331	-0.619643	-1.305420
H	-8.864795	-1.073941	2.128405	H	-3.210203	-1.137469	-0.463458
H	-8.344270	-3.347585	-0.894368	C	-1.433377	-0.281432	-1.300025
H	-8.834339	-1.755578	-1.504072	H	-0.844706	-0.356558	-0.392114
H	-7.148828	-2.273229	-1.662026	C	-3.136270	1.895909	0.498504
H	-7.167755	-2.694776	3.040308	C	-5.506614	0.646761	-2.237009
H	-5.448211	-2.271685	2.963730	H	-5.075313	1.426875	-2.881756
H	-6.672631	-0.996495	2.860352	H	-6.564467	0.536532	-2.493121
H	-5.115609	-3.897444	1.179483	C	-5.342109	1.040577	-0.744348
H	-6.825672	-4.332307	1.017991	C	-3.893690	1.455637	-0.579504
H	-5.915234	-3.749473	-0.392921	C	-4.065042	0.768501	2.605778
C	3.494931	-3.885385	-1.829710	H	-4.172632	-0.166102	2.069032
C	3.494410	-2.542441	-2.248607	C	-4.395979	0.834258	3.962874
C	3.814710	-1.475979	-1.397228	H	-4.765524	-0.054550	4.468981
C	4.144319	-1.737157	-0.028918	C	-4.245686	2.026762	4.670650
C	4.159063	-3.072664	0.345292	H	-4.508959	2.076764	5.724280
C	3.841535	-4.118976	-0.517503	C	-3.396524	3.084568	2.667688
C	4.544613	-0.719588	0.996082	H	-2.986701	3.953092	2.161119
C	3.668568	0.186793	1.674860	C	-3.743960	3.155059	4.014731
C	4.188134	1.055242	2.644843	H	-3.617449	4.089056	4.557020
H	3.526009	1.749198	3.148374	C	-3.568098	1.894717	1.936527
C	5.546785	1.074936	3.008715	H	-3.450116	1.802083	-1.511217
C	6.368549	0.177302	2.363955	H	-6.017408	1.903325	-0.597824
H	3.241274	-4.694056	-2.506343	C	1.982548	4.340055	-1.039662
H	3.232281	-2.341522	-3.280385	C	2.193909	4.353724	-2.567510
C	5.873013	-0.690795	1.394196	H	2.795496	3.488630	-2.871883
C	8.038894	-1.156252	1.714855	H	1.228495	4.283533	-3.082218
O	6.905203	-1.451840	0.894096	H	2.703054	5.265851	-2.905802
O	7.720723	-0.009246	2.510038	C	1.115628	5.551555	-0.635707
H	5.925600	1.757795	3.761261	H	0.141255	5.506102	-1.140328
C	4.448382	-5.002569	1.443065	H	0.939740	5.543834	0.446768
H	5.481739	-5.370856	1.508067	H	1.586427	6.506891	-0.900812
H	3.822126	-5.455649	2.216879	C	3.346696	4.432010	-0.326746
O	4.436622	-3.580227	1.593453	H	3.204141	4.434952	0.760451
O	3.911339	-5.314349	0.152670	H	3.961725	3.560655	-0.582538
H	8.899606	-0.930862	1.078891	H	3.899152	5.339202	-0.604636
H	8.249327	-2.011205	2.372824	O	1.313239	3.165504	-0.647253
H	0.049995	1.418463	2.291234	Li	-0.404252	2.741445	-0.434811
H	1.338284	1.109409	3.486302				

H	1.479905	2.444210	2.292410				
H	3.506157	1.107487	-4.253683				
H	4.164341	-0.547541	-4.318922				
H	2.414468	-0.255288	-4.024691				
H	6.125403	0.182200	-2.478078				
H	5.483517	1.849021	-2.457402				

Int6							
P	2.553249	0.034594	-2.027902	C	-1.927967	2.333594	1.260280
P	2.724350	1.034012	1.479606	H	-1.691224	2.749982	0.282272
C	4.176331	-0.104574	-2.920319	C	-1.846255	-0.166553	-0.390398
C	1.363300	-0.146221	-3.429721	C	-3.983947	-1.076259	2.733564
C	3.023755	2.279300	2.813127	H	-4.736454	-1.813261	3.028848
C	1.669466	-0.189647	2.386084	H	-3.089222	-1.221428	3.362129
Cu	2.195348	1.795847	-0.663291	C	-3.618376	-1.215129	1.241043
C	6.815608	0.335451	1.420097	H	-3.216567	-2.231282	1.108574
C	5.524145	0.829727	1.677230	C	-2.508346	-0.170435	0.975727
C	4.356531	0.198691	1.223998	H	-1.686543	-0.360479	1.677867
C	4.459335	-1.003819	0.452087	C	-2.913543	-1.943304	-1.874047
C	5.746110	-1.472801	0.230489	H	-2.763160	-2.684854	-1.095236
C	6.892774	-0.829112	0.689106	C	-3.437062	-2.338313	-3.108390
C	3.317924	-1.849715	-0.024786	H	-3.692157	-3.381693	-3.275301
C	2.427104	-1.562354	-1.106818	C	-3.631293	-1.398548	-4.122451
C	1.407244	-2.469766	-1.426244	H	-4.046450	-1.705158	-5.078866
H	0.715082	-2.239535	-2.226103	C	-2.726666	0.325760	-2.685369
C	1.221278	-3.684324	-0.743253	H	-2.430386	1.358127	-2.523440
C	2.108198	-3.959752	0.274068	C	-3.278663	-0.063260	-3.904997
H	7.702797	0.841890	1.784118	H	-3.422985	0.673313	-4.691097
H	5.444049	1.739747	2.259720	C	-2.554247	-0.606820	-1.651136
C	3.121018	-3.066415	0.612486	C	-7.087288	-1.768669	-0.221861
C	3.336232	-4.891604	1.890874	C	-6.872767	-0.226079	-0.507670
O	3.830938	-3.566554	1.679354	B	-4.995346	-1.109929	0.459547
O	2.148579	-5.049159	1.105641	O	-5.938784	-2.100095	0.605486
H	0.422878	-4.369362	-1.006497	O	-5.440325	-0.061887	-0.304632
C	7.520515	-2.729634	-0.302262	C	-8.353200	-2.110487	0.565071
H	7.726226	-3.572980	0.371786	H	-9.252196	-1.833048	0.002556
H	7.997516	-2.880310	-1.274764	H	-8.388452	-3.189400	0.748460
O	6.107627	-2.593927	-0.481262	H	-8.376032	-1.603410	1.532239
O	8.008591	-1.518901	0.285280	C	-7.000502	-2.648347	-1.476154
H	3.093340	-5.025560	2.948696	H	-6.979307	-3.699435	-1.169909
H	4.089357	-5.622096	1.564102	H	-7.864653	-2.499894	-2.132693
H	1.506902	0.716078	-4.088851	H	-6.090038	-2.442940	-2.046037
H	1.520521	-1.062645	-4.009189	C	-7.207511	0.224804	-1.928854
H	0.340408	-0.111913	-3.046695	H	-8.270399	0.071529	-2.149522
H	2.037597	2.698764	3.052099	H	-6.992280	1.293365	-2.031451
H	3.472971	1.841742	3.711887	H	-6.611566	-0.309556	-2.672158
H	3.655421	3.095179	2.448714	C	-7.562855	0.688090	0.514374
H	2.202206	-0.624587	3.238643	H	-7.249396	1.721215	0.333462
H	0.784373	0.346240	2.741942	H	-8.653727	0.641518	0.427698
H	1.339615	-0.982052	1.712985	H	-7.274477	0.428131	1.537125
H	4.270869	0.747503	-3.602222	O	1.701739	3.684002	-0.741992
H	5.002205	-0.063969	-2.206607	Li	0.716098	3.775249	0.743068
H	4.240974	-1.035572	-3.493530	C	1.867440	4.544122	-1.852048
O	-0.063407	3.419571	2.297948	C	1.441489	5.963641	-1.425850
C	-1.118970	2.632606	2.325486	H	1.558811	6.691192	-2.238437
O	-4.564445	0.194383	2.976016	H	0.386533	5.970888	-1.120950
C	-1.452015	2.003801	3.629961	H	2.047965	6.300970	-0.576399
H	-0.721097	2.125022	4.427688	C	3.346876	4.561003	-2.281232
C	-2.606020	1.350557	3.830116	H	3.520415	5.228147	-3.135288

H	-2.870244	0.922826	4.795895	H	3.976669	4.893322	-1.447892
O	-0.699862	0.256170	-0.518592	H	3.667036	3.549869	-2.563316
C	-3.676710	1.310823	2.754060	C	0.984701	4.074736	-3.024144
C	-4.587714	2.539730	2.906567	H	-0.067131	4.047375	-2.716547
H	-5.397672	2.494010	2.170101	H	1.074049	4.735845	-3.895703
H	-4.012786	3.455483	2.751372	H	1.271301	3.061721	-3.329884
H	-5.030221	2.553535	3.907876				
C	-3.024800	1.302676	1.335689				
H	-3.821039	1.535296	0.621246				

Int6’							
P	3.695520	-2.058201	-1.348719	C	-7.337745	-0.370597	1.892298
P	1.958784	1.050704	-0.569484	C	-5.587342	-1.136713	3.692223
C	5.325468	-1.710834	-2.172233	C	-5.874113	1.314880	3.234482
C	3.576192	-3.887250	-1.604058	C	-7.680518	-1.862559	1.774135
C	1.139543	2.534054	-1.311906	C	-8.540677	0.407739	2.422408
C	1.338064	1.154580	1.174791	H	-4.610912	-0.879990	4.115478
Cu	1.722178	-0.903999	-1.698016	H	-6.317356	-1.160255	4.509933
C	5.561068	3.051639	-1.136693	H	-5.513441	-2.136989	3.259778
C	4.207067	2.671431	-1.111130	H	-6.521542	1.435036	4.109899
C	3.746116	1.520000	-0.456945	H	-4.838901	1.494210	3.540880
C	4.680924	0.666393	0.212689	H	-6.143842	2.076478	2.495783
C	6.004652	1.081651	0.187115	H	-7.945995	-2.295983	2.744452
C	6.441346	2.231148	-0.467129	H	-8.535589	-1.976927	1.100317
C	4.363144	-0.572062	0.995639	H	-6.844974	-2.428206	1.350186
C	4.026523	-1.856231	0.457850	H	-9.420709	0.182793	1.811129
C	3.819975	-2.938690	1.324578	H	-8.765404	0.122849	3.456930
H	3.553388	-3.906059	0.916138	H	-8.373066	1.486742	2.386615
C	3.941233	-2.836613	2.721978	O	0.317941	-1.782413	-2.640835
C	4.286040	-1.599551	3.219191	C	-0.949273	-1.745241	-2.252627
H	5.892686	3.946735	-1.651382	O	-5.114865	-2.181582	-1.446023
H	3.500032	3.313852	-1.622023	C	-1.931777	-2.239840	-3.249839
C	4.493615	-0.510904	2.375245	H	-1.557724	-2.405591	-4.258351
C	4.926423	0.134763	4.477460	C	-3.203583	-2.505888	-2.918295
O	4.808294	0.595711	3.127918	H	-3.914000	-2.912873	-3.636298
O	4.462264	-1.219093	4.524475	O	-2.175634	1.048153	0.716316
H	3.774517	-3.687996	3.372492	C	-3.682952	-2.381677	-1.480480
C	8.200640	1.313631	0.560464	C	-3.482554	-3.722679	-0.760293
H	8.484325	1.756265	1.525318	H	-3.900504	-3.664583	0.251644
H	9.034271	0.754413	0.126168	H	-2.416470	-3.954525	-0.693913
O	7.084633	0.437878	0.744829	H	-3.994703	-4.524673	-1.302096
O	7.802021	2.351661	-0.342103	C	-2.909981	-1.248724	-0.745483
H	4.305143	0.755461	5.130003	H	-3.080109	-1.380886	0.328271
H	5.979766	0.171214	4.786993	C	-1.425466	-1.334085	-1.042870
H	3.520003	-4.051908	-2.685080	H	-0.735518	-0.964715	-0.289617
H	4.432212	-4.440038	-1.201075	C	-2.826288	1.269050	-0.297129
H	2.647167	-4.268817	-1.172290	C	-5.542470	-0.926383	-1.948202
H	0.061152	2.359517	-1.255675	H	-5.192908	-0.785404	-2.984921
H	1.373347	3.461505	-0.777737	H	-6.636153	-0.958325	-1.952639
H	1.407319	2.638582	-2.367154	C	-5.023465	0.210299	-1.051272
H	1.628185	2.102212	1.641692	C	-3.467423	0.156481	-1.122088
H	0.245517	1.077243	1.153376	C	-3.423459	3.072863	-2.027787
H	1.734895	0.326071	1.765954	H	-3.618037	2.313438	-2.777913
H	5.229329	-1.969057	-3.232478	C	-3.560607	4.419183	-2.369944
H	5.569264	-0.648896	-2.101039	H	-3.865398	4.690729	-3.377048
H	6.137972	-2.298501	-1.731326	C	-3.307824	5.412934	-1.422607
B	-5.677779	0.047688	0.380485	H	-3.421838	6.460584	-1.688853
O	-5.008718	-0.162797	1.557649	C	-2.758456	3.713910	0.205681
O	-7.044004	0.093834	0.544674	H	-2.439896	3.415591	1.199295

C	-5.976088	-0.097462	2.642082	C	-2.906354	5.056518	-0.131363
				H	-2.709502	5.827086	0.609619
				C	-3.020898	2.703313	-0.734464
				H	-3.188737	0.336299	-2.170812
				H	-5.383875	1.160563	-1.473594

Int6’’							
P	2.183355	0.116750	1.416774	H	5.710942	1.856469	-0.759790
P	3.684139	0.892712	-1.769994	H	0.738022	-1.808993	1.232847
C	1.819147	-1.702503	1.371000	H	2.311132	-2.165194	0.514007
C	1.318782	0.561936	2.998136	H	2.130146	-2.210010	2.290812
C	3.257478	0.941343	-3.568153	O	0.423656	0.196165	-1.846284
C	5.270508	1.865388	-1.759978	C	-0.630748	-0.541637	-1.576412
Cu	1.799885	1.306535	-0.473962	O	-4.276411	-2.749543	-1.446258
B	-6.053909	-0.992589	-0.124504	C	-1.027103	-1.550417	-2.592314
O	-7.353985	-1.419895	-0.263869	H	-0.400782	-1.612322	-3.479774
O	-5.579091	-1.175552	1.154612	C	-2.093174	-2.347483	-2.427975
C	-7.684424	-2.221768	0.904761	H	-2.364792	-3.102476	-3.163963
C	-6.672995	-1.672581	1.983793	O	-2.597834	2.082293	-1.552342
C	-9.162572	-2.016527	1.230566	C	-2.925541	-2.320435	-1.157329
C	-7.423209	-3.684603	0.516670	C	-2.406560	-3.386276	-0.181170
C	-7.218369	-0.480214	2.779893	H	-3.038764	-3.405668	0.713782
C	-6.101830	-2.720342	2.938216	H	-1.377792	-3.161884	0.110975
H	-9.773610	-2.397883	0.406299	H	-2.440504	-4.374032	-0.651884
H	-9.442941	-2.561696	2.139261	C	-2.860224	-0.904555	-0.502422
H	-9.405222	-0.960279	1.367445	H	-3.268194	-0.988628	0.508616
H	-7.674678	-4.371054	1.332185	C	-1.447054	-0.386055	-0.482229
H	-8.046817	-3.937071	-0.346933	H	-1.186579	0.361761	0.265869
H	-6.377412	-3.838644	0.232225	C	-3.603213	1.547830	-1.060979
H	-8.013232	-0.788238	3.467332	C	-5.061259	-1.838362	-2.205454
H	-6.407668	-0.044333	3.373264	H	-4.588092	-1.633273	-3.179515
H	-7.613388	0.296095	2.117827	H	-6.014135	-2.343582	-2.383471
H	-5.379843	-2.247215	3.611803	C	-5.249672	-0.522733	-1.410358
H	-6.894748	-3.164428	3.551143	C	-3.818472	0.067193	-1.333248
H	-5.587802	-3.518781	2.398825	C	-5.201825	2.025240	0.863326
C	4.466243	-3.161947	-2.291936	H	-5.069165	1.019200	1.242154
C	4.115596	-1.812644	-2.476136	C	-5.998954	2.931036	1.566187
C	4.257591	-0.840953	-1.475612	H	-6.489858	2.615417	2.482644
C	4.761965	-1.219518	-0.190782	C	-6.163480	4.236139	1.099866
C	5.124602	-2.551595	-0.050692	H	-6.795550	4.933366	1.643528
C	4.980551	-3.499839	-1.059647	C	-4.685792	3.757281	-0.750209
C	4.987314	-0.315940	0.984044	H	-4.152720	4.071795	-1.641352
C	3.981584	0.239746	1.840371	C	-5.506497	4.647152	-0.063850
C	4.367128	1.007498	2.949439	H	-5.626861	5.663508	-0.428677
H	3.605846	1.433561	3.592023	C	-4.537675	2.431395	-0.302933
C	5.709419	1.259461	3.284192	H	-3.412552	-0.000204	-2.351824
C	6.661273	0.701604	2.460030	H	-5.899444	0.135741	-2.005640
H	4.341448	-3.896632	-3.079914	C	0.831426	4.231621	-0.387596
H	3.715236	-1.527575	-3.441333	C	2.213338	4.742624	-0.848879
C	6.300937	-0.062446	1.353366	H	3.004363	4.110887	-0.426004
C	8.530243	-0.102379	1.541586	H	2.283678	4.689618	-1.942210
O	7.438880	-0.475629	0.697349	H	2.397781	5.780258	-0.540300
O	8.029201	0.792489	2.540343	C	-0.268915	5.123811	-1.001580
H	5.979581	1.856337	4.148443	H	-0.217162	5.078495	-2.096676
C	5.954777	-4.495247	0.686139	H	-1.258637	4.766827	-0.693613
H	7.047744	-4.598843	0.627402	H	-0.169784	6.174037	-0.696974
H	5.533611	-5.203307	1.405645	C	0.748342	4.309316	1.152448
O	5.620920	-3.161967	1.079576	H	-0.227948	3.944347	1.493112
O	5.383481	-4.731577	-0.604623	H	1.524516	3.673892	1.597313

H	9.293349	0.406913	0.946477	H	0.885946	5.332741	1.526334
H	8.942904	-0.998300	2.027125	O	0.634924	2.914190	-0.818174
H	0.260966	0.317498	2.853995	Li	-0.608356	1.844040	-1.547042
H	1.695456	0.010051	3.866954				
H	1.381392	1.636864	3.187745				
H	3.068379	1.990988	-3.817447				
H	4.047813	0.558020	-4.223597				
H	2.324065	0.393330	-3.722088				
H	5.999185	1.468866	-2.475657				
H	5.036636	2.903561	-2.019523				
